# *peri*-Acenoacene Ribbons with Zigzag BN-Doped Peripheries

**DOI:** 10.1021/jacs.2c06803

**Published:** 2022-11-17

**Authors:** Marco Franceschini, Martina Crosta, Rúben R. Ferreira, Daniele Poletto, Nicola Demitri, J. Patrick Zobel, Leticia González, Davide Bonifazi

**Affiliations:** † Institute of Organic Chemistry, Faculty of Chemistry, University of Vienna, Währinger Straße 38, 1090, Vienna, Austria; ‡ Elettra − Sincrotrone Trieste, S.S. 14 Km 163.5 in Area Science Park, 34149 Basovizza, Trieste, Italy; § Institute of Theoretical Chemistry, Faculty of Chemistry, University of Vienna, Währinger Straße 17, 1090, Vienna, Austria

## Abstract

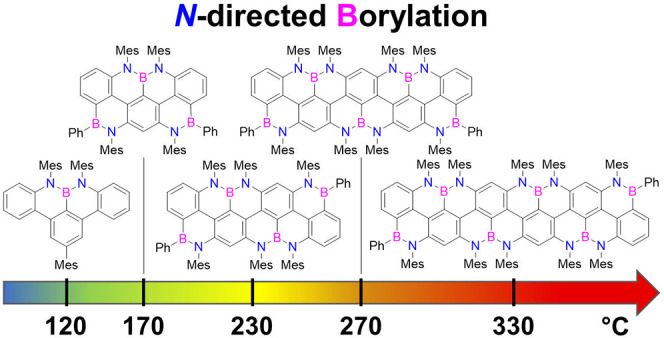

Here, we report the synthesis of BN-doped graphenoid nanoribbons, in which peripheral carbon atoms at the zigzag edges have been selectively replaced by boron and nitrogen atoms as BN and NBN motifs. This includes high-yielding ring closure key steps that, through *N*-directed borylation reaction using solely BBr_3_, allow the planarization of *meta*-oligoarylenyl precursors, through the formation of B–N and B–C bonds, to give ter-, quater-, quinque-, and sexi-arylenyl nanoribbons. X-ray single-crystal diffraction studies confirmed the formation of the BN and NBN motifs and the zigzag-edged topology of the regularly doped ribbons. Steady-state absorption and emission investigations at room temperature showed a systematic bathochromic shift of the UV–vis absorption and emission envelopes upon elongation of the oligoarylenyl backbone, with the nanoribbon emission featuring a TADF component. All derivatives displayed phosphorescence at 77 K. Electrochemical studies showed that the π-extension of the *peri*-acenoacene framework provokes a lowering of the first oxidative event (from 0.83 to 0.40 V), making these nanoribbons optimal candidates to engineer *p*-type organic semiconductors.

## Introduction

**Figure 1:**
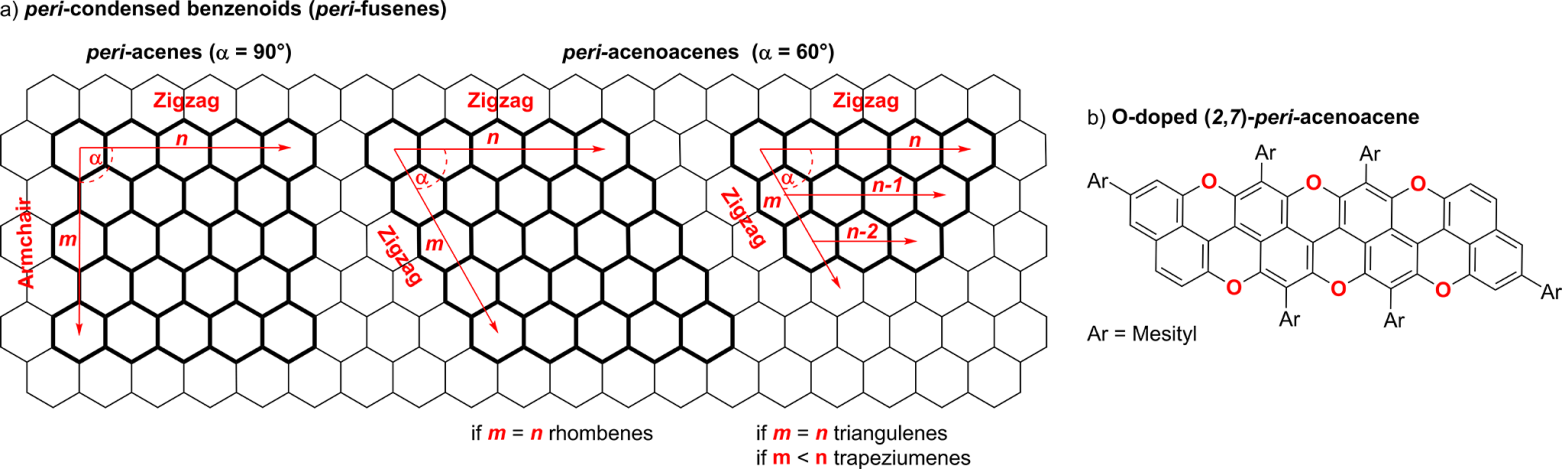
(a) (*m*,*n*)-*peri*-Acenes and (*m*,*n*)-*peri*-acenoacenes nanographenes: armchair/zigzag (left) and zigzag/zigzag (right) topologies; (b) example of an O-doped (2,7)-*peri*-acenoacene derivative reported by our group.^1^

The replacement of C(sp^2^) atoms with heteroatoms in polycyclic aromatic hydrocarbons (PAHs) is emerging as an attractive approach to prepare hybrid graphenoid structures that, if constituted only by C(sp^2^), could be of difficult synthesis following classical in-solution approaches, or chemically unstable under ambient conditions.

(*m*,*n*)-*peri*-Acenes exhibit orthogonal zigzag and armchair edges and derive from the fusion of an *m* number of [*n*]acenes at their *peri*-position.^2−10^ Instead, (*m*,*n*)-*peri*-acenoacenes are nanographenes with two zigzag peripheries with a 60° angle (Figure 1a) and are the rarest examples.^11−14^ When the annulated [*n*]acenes display different *n*, one could refer to either *n*-*peri*-acenoacenes (i.e., triangulenes) or (*m*, *n*, *n*-1, *n*-2, . . ., *n*-*m*)-*peri*-acenoacenes (i.e., also known as benzo[*c*]acenoacenes or trapeziumenes). Generally, the substitution of the carbon edges with heteroatoms has provided a viable approach to prepare zigzag nanographenes, and noticeable examples include O-,^1−17^ BO-,^18,19^ OBO-,^20−23^ B/N-,^24−30^ NBN-,^22,31−35^ and BNB-doped^36,37^ structures. For example, a few years ago, our group reported the first stable (2,7)-*peri*-acenoacene congener, in which six carbon atoms at the zigzag edges have been replaced by O atoms (Figure 1b). The presence of the O atoms provides air-stable PAHs, which can be used as *p*-type semiconductors.^1^

**Figure 2:**
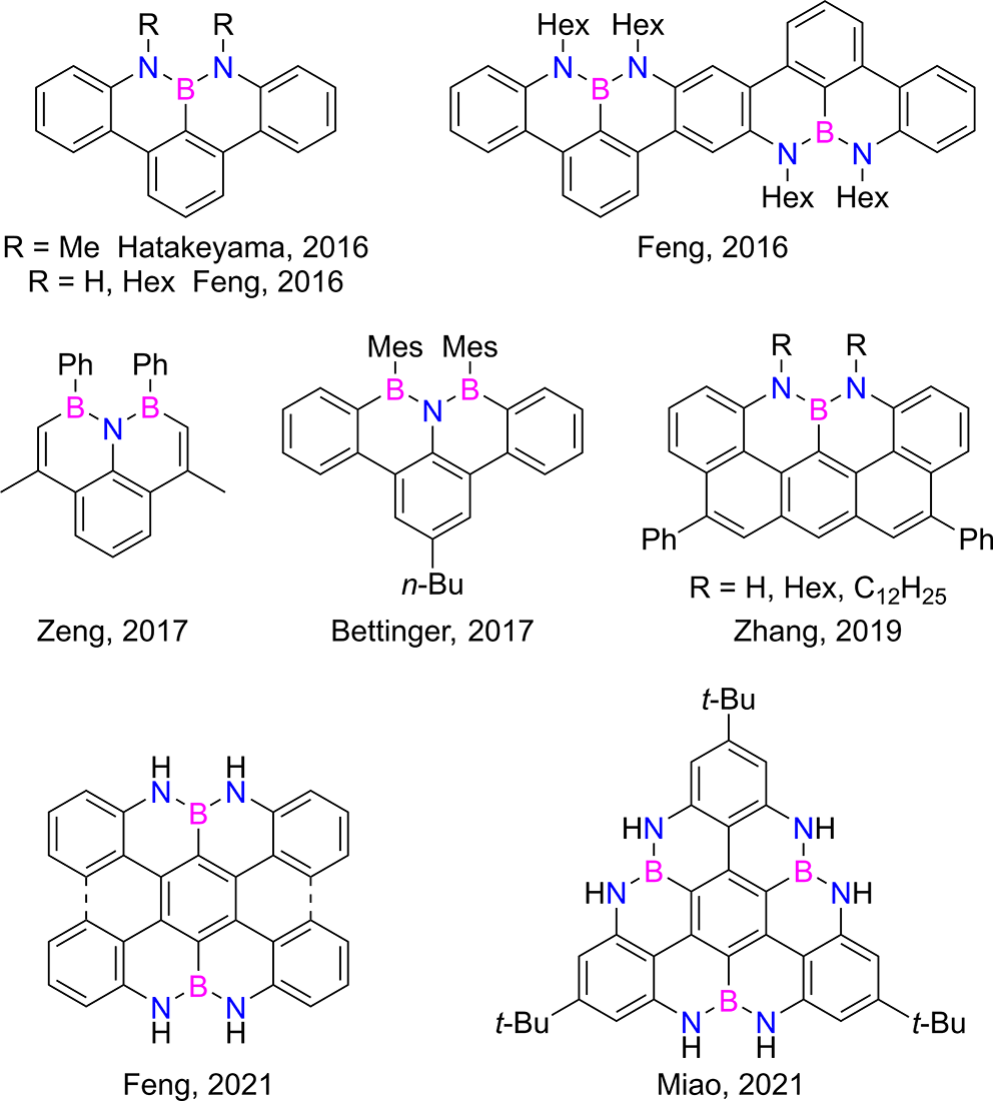
Representative BN-doped molecular graphenoids featuring zigzag peripheries.

The use of isostructural and isoelectronic B–N couples to substitute C=C bonds has attracted increasing interest in the organic chemistry community as a versatile route to prepare isostructural PAHs.^38−40^ For instance, small PAHs featuring zigzag peripheries could be synthesized in solution when NBN motifs are inserted at the peripheral zigzag positions. Seminal examples (Figure 2) include NBN-benzo[*fg*]tetracenes also known as NBN-dibenzophenalenes,^22,31^ NBN-dibenzoheptazethrene,^31^ BNB-benzo[*fg*]tetracene,^36^ BNB-embedded phenalenyls,^37^ NBN-anthratetracenes,^32^ NBN-*bis*- and *peri*-tetracene,^33^ and B_3_N_6_-[4]triangulene.^34^ Exceptional cases also include NBN-decorated nanoribbons prepared by on-surface synthesis.^41^ In particular, BN-doped PAHs containing NBN patterns revealed to be unique frameworks to access isoelectronic analogs of all-C derivatives when oxidized to either mono- or dicationic species. For instance, by chemical and electrochemical oxidation of NBN-dibenzophenalenes and NBN-*bis*- and *peri*-tetracenes, it has been shown that isoelectronic congeners of dibenzophenalenes^31^ and *bis*- and *peri*-tetracenes^33^ could be easily accessed.

However, to the best of our knowledge, only the shortest terms of what can be considered ribbon-like structures have been prepared to date in solution (Figure 2), and monodispersed molecular structures with regular BN-doping patterns remain rather uncommon. It is with this challenge in mind that, in this paper, we report on the preparation of BN-doped molecular ribbons featuring *peri*-acenoacene topologies. In particular, we focus on the synthesis, structural and optoelectronic characterization of BN-doped (2,4,3)-, (2,5)-, (2,7,6)-, (2,7)-*peri*-acenoacene derivatives with *meta*-linked ter-, quater-, quinque-, and sexi-arylenyl skeletons, respectively. These molecules show doping ratios (ρ , where *n*(B), *n*(N), and *n*(C) are the numbers of boron, nitrogen, and carbon atoms) in the range between 28% and 31% that, to the best of our knowledge, are the highest doping values reported so far for discrete BN-doped graphenoid-type molecules.

## Results and Discussion

### The Planarization Approach

**Scheme 1:**
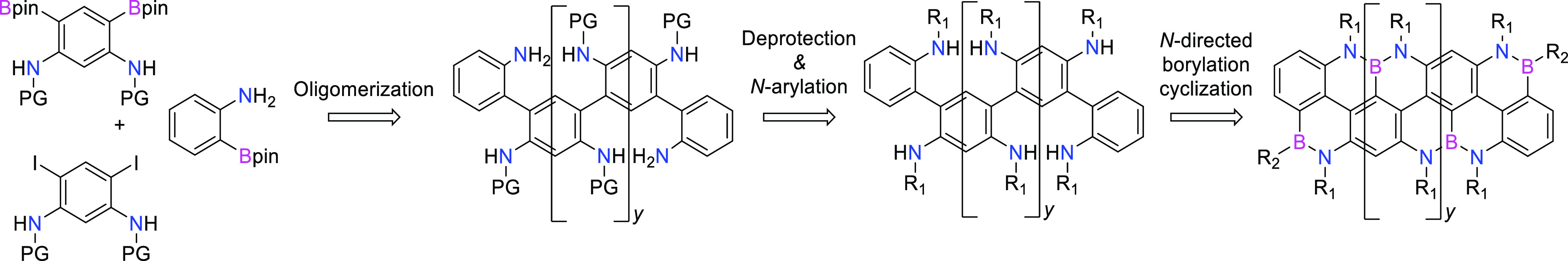
Envisaged Synthetic Strategy towards BN-Doped Zigzag Graphenoid Nanoribbons Using *N*-Directed Borylation As the Planarization Reaction (PG = Protecting Group)

Generally, heteroatom-doped graphene substructures are obtained through bottom-up synthesis of precursor scaffolds, featuring the aromatic heterocycles in defined positions, and successively planarized through C–C bond formation reactions.^41−43^ Building on early works describing the synthesis of 9,10-azaboraphenanthrenes^44^ and NBN-dibenzophenalenes^22,31^ through *N*-directed borylation of the relevant amino-bearing precursors, we envisioned the BN-doped zigzag nanoribbons proposed in this work as arising by *meta*-oligoarylenes, with 1,5-diaminoaryl and 1-aminoaryl moieties being the key monomeric and terminating units (Scheme 1). At the synthetic planning level, we contemplated the addition/elimination and electrophilic aromatic substitution reactions to form B–N and B–C bonds, respectively, as the planarization reactions. As we anticipated the potential insolubility of the BN-doped nanoribbons in common organic solvents, each amino group was equipped with a solubilizing mesityl (Mes) moiety.

**Scheme 2:**
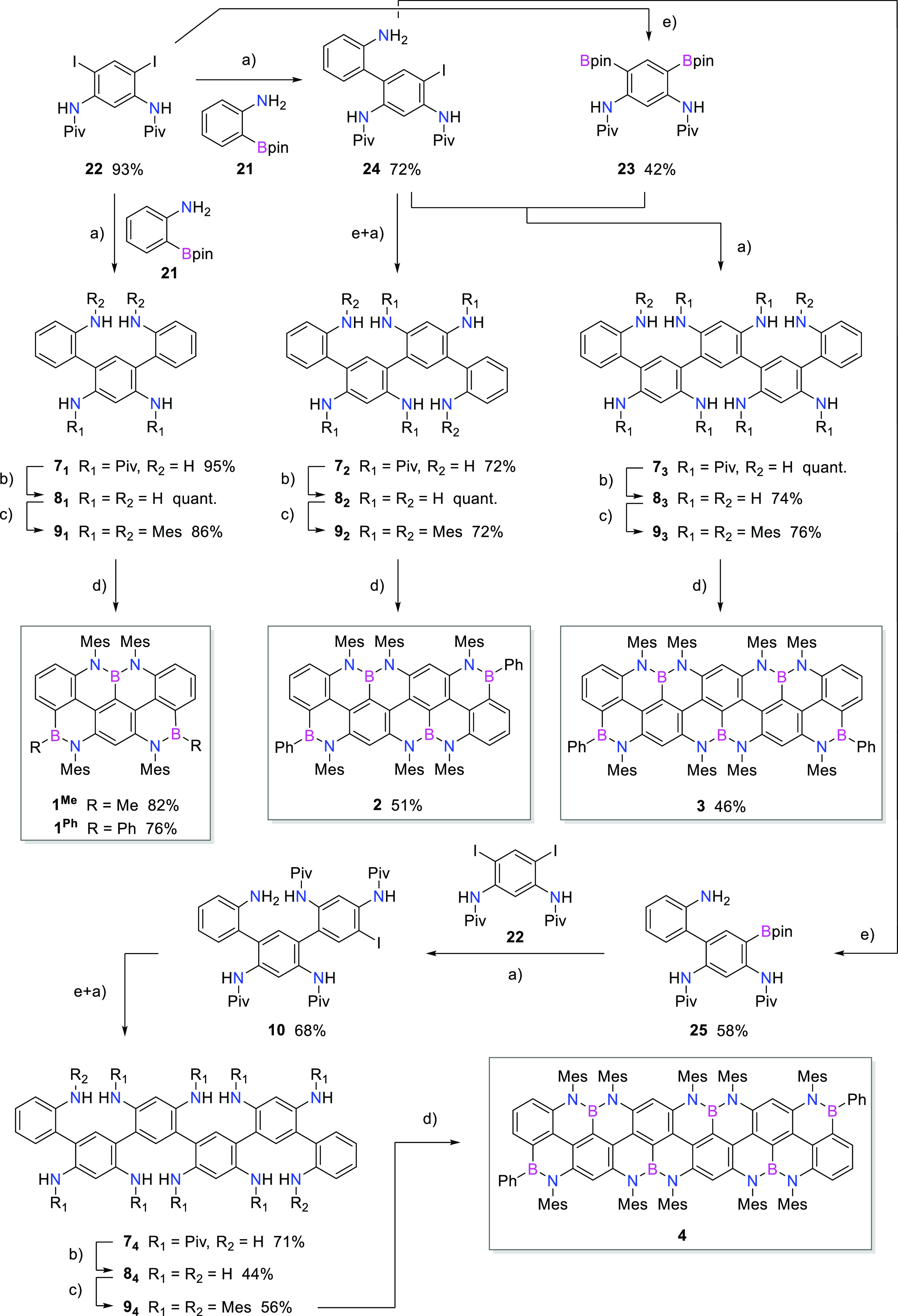
Synthesis of BN-Doped Zigzag Graphenoid Nanoribbons Reagents and conditions: (a) Na_2_CO_3_, [Pd(PPh_3_)_4_], toluene/EtOH/H_2_O, 95 °C; (b) 6 M aq. HCl, dioxane, 100–120 °C; (c) MesBr, *t*-BuONa, [Pd_2_(dba)_3_], *rac*-BINAP, toluene, 100–110 °C; (d) BBr_3_, *o*-DCB or TCB, 170–330 °C; (e) B_2_pin_2_, KOAc, [Pd(dppf)Cl_2_], DMF, 60–95 °C.

Specifically, four zigzag nanoribbons (Scheme 2) bearing *meta*-linked ter-, quater-, quinque-, and sexi-arylenyl skeletons (**1**, **2**, **3**, and **4**), respectively, along with molecular references **5** and **6** (Tables 1 and 2), have been conceived. All *meta*-oligoarylenes were synthesized by sequential Suzuki-type cross-coupling reactions,^45^ whereas multiple Buchwald–Hartwig *N*-arylations^46^ were used to equip each amino group with a solubilizing Mes moiety. It should be noted that all *meta*-oligoarylenes intermediates **7**_*y*_–**9**_*y*_ and **10** (Scheme 2) were prepared and used as atropoisomeric mixtures.

**Table 1:**
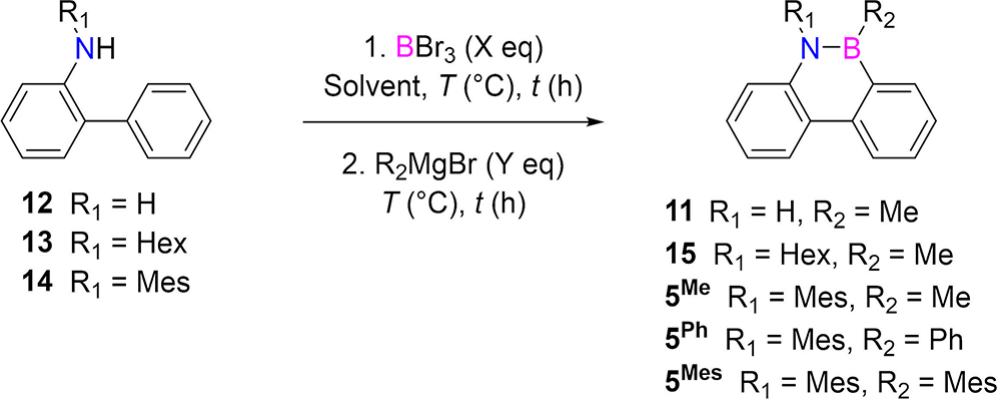

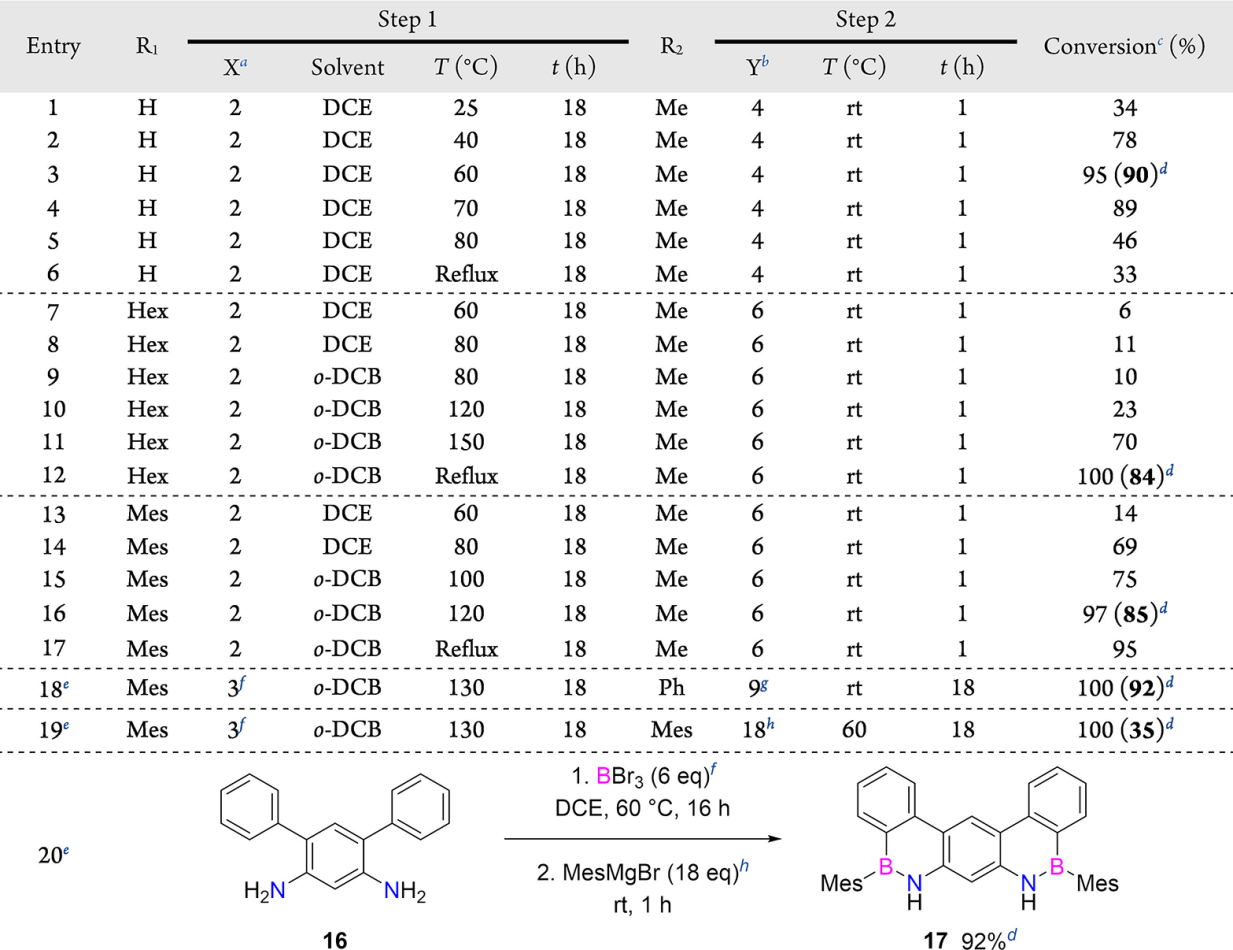
Optimization of the *N*-Directed BBr_3_-Based Borylation Reaction To Prepare *N*- and *B*-Substituted Phenanthrenes

a BBr_3_ 1 M in hexane solution, unless otherwise specified.

b MeMgBr 3 M in diethyl ether solution, unless otherwise specified.

c Conversions were calculated by ^1^H NMR spectroscopy.

d Isolated yield.

e Reaction performed in the glovebox.

f BBr_3_ neat.

g PhMgBr 16% solution in THF.

h MesMgBr solid obtained from a commercial solution. rt = room temperature.

**Table 2:**
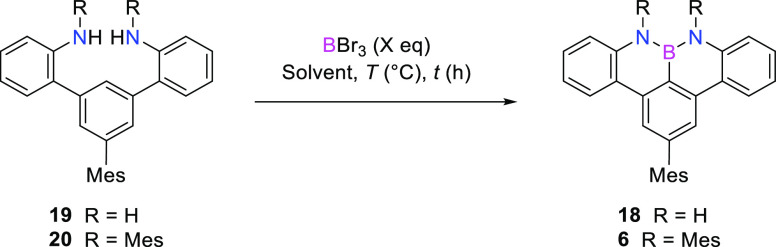
Optimization of the *N*-Directed BBr_3_-Based Borylation Reaction To Prepare *N*-Substituted NBN-Doped Dibenzophenalenes

Entry	R	X^a^	Solvent	*T* (°C)	*t* (h)	Conversion^b^ (%)
1	H	2	DCE	60	18	43
2	H	2	DCE	80	18	60 (21)^c^
3	H	4	DCE	80	18	37
4^d^	H	4^e^	DCE	80	18	100 (**60**)^c^
5^d^	H	6^e^	DCE	80	18	100 (52)^c^
6^d^	H	4^e^	*o*-DCB	120	18	100 (34)^c^
						
7	Mes	2	*o*-DCB	120	18	100 (56)^c^
8^d^	Mes	4^e^	*o*-DCB	120	18	100 (**85**)^c^
9	Mes	2	*o*-DCB	Reflux	18	100 (56)^c^

a BBr_3_ 1 M in hexane solution, unless otherwise specified.

b Conversions were calculated by ^1^H NMR spectroscopy.

c Isolated yield.

d Reaction performed in the glovebox.

e BBr_3_ neat.

### Development and Optimization of a Single Component Borylation Reaction

Our studies began by investigating the optimal conditions for the single-step *N*-directed borylation to prepare model BN-phenanthrene **11** (Table 1). Inspired by literature protocols reporting the use of BBr_3_ as a direct borylating agent in electrophilic aromatic substitutions of activated polycyclic aromatic hydrocarbons,^100,22,26,47−51^ we conjectured that BBr_3_ could act as both electrophile and Lewis acid, self-promoting the *N*-directed borylation reaction without the need of an additional Lewis acid or base (i.e., AlCl_3_, Et_3_N), as used so far for preparing the BN-doped PAHs.^31−34,44,52^ Thus, commercially available 2-aminobiphenyl **12** was reacted overnight with 2 equiv of BBr_3_ (1 M solution in heptane) in 1,2-dichloroethane (DCE) at various temperatures (Table 1, entries 1–6). As one can expect, the borylation reaction exhibits a significant temperature dependence, with 95% conversion (90% yield after purification) achieved at 60 °C (entry 3). However, when a solubilizing *N*-hexyl and *N*-mesityl moiety is present (Table 1, entries 7–12 and 13–17), no satisfactory conversions could be obtained at 60 °C. When increasing the reaction temperatures to 180 and 120 °C in 1,2-dichlorobenzene (*o*-DCB) for molecules **13** and **14**, respectively (entries 12 and 16), the borylation reaction occurred satisfactorily, and BN-derivatives **15** and **5**^**Me**^ were isolated in 84% and 85% yield, respectively.

With the optimized borylation conditions in hand, different organometallic reagents were investigated for the *B*-alkylation/arylation (Table 1, entries 18–19). While an excellent 92% yield could be obtained for *B*-Ph derivative **5**^**Ph**^, sterically encumbered derivative *B*-Mes **5**^**Mes**^ was isolated in a mediocre yield (35%), despite the use of a large excess of MesMgBr (18 equiv). Although the Mes group provided stability toward parasitic protiodeborylation reaction,^53^ the low yield for this arylation reaction with MesMgBr appeared to be inadequate for preparing our ribbons in high yields. To assess whether multiple borylations are accessible with our strategy, diamine substrate **16** (Table 1, entry 20) was also treated with 6 equiv of BBr_3_ at 60 °C, followed by the addition of MesMgBr (18 equiv). Pleasingly, the approach gave access to the desired diborylated compound **17** in 92% yield.

Our attention was then directed toward the reaction optimization to prepare the model NBN-dibenzophenalene **18** (Table 2). Tuning (entries 1–6) the temperature to 80 °C and the amount of neat BBr_3_ to 4 equiv, the borylation of **19** in DCE gave NBN-dibenzophenalene derivative **18** in 60% yield (entry 4). Higher equivalents of BBr_3_ and higher temperatures afforded the product in lower yields (entries 5–6). Borylation of *N*-mesityl **20** was at last considered (Table 2, entries 7–9). Building on the reaction conditions to prepare reference molecule **5**^**Me**^, a temperature of 120 °C was selected for the borylation of **20**. Similarly to the case of derivative **18**, 4 equiv of neat BBr_3_ gave NBN-doped dibenzophenalene **6** in a very high yield (85%, Table 2, entry 8).

### Synthesis of the BNC Ribbons

Having optimized the borylation conditions, we focused our efforts on the synthesis (Scheme 2) of the BN-doped nanoribbons, following the synthetic approach presented in Scheme 1. Pivaloyl (Piv) -protected *meta*-oligoarylene precursors (**7**_***y***_, *y* = 1–4) were achieved through sequential Suzuki–Miyaura cross-coupling reactions, following two synthetic strategies: convergent and linear approaches for the odd- and even-numbered *meta*-oligoarylenyl derivatives, respectively. All *meta*-oligoarylenes were prepared starting from two modules: commercial aniline boron pinacolate (Bpin) **21** and diiodobispivalamide **22** (prepared in two steps in 90% overall yield). Molecule **7**_**1**_ was prepared in 95% yield by reacting boronic ester **21** with diiodobispivalamide **22**, under typical Suzuki–Miyaura cross-coupling conditions. Quinquearylene **7**_**3**_ was instead prepared in quantitative yield, through Suzuki–Miyaura cross-coupling between bis(boronpinacolato)bispivalamide **23** (obtained from diiodobispivalamide **22** in 42% yield) and iodobiarylene **24** (synthesized from commercial boronic ester **21** and diiodobispivalamide **22** in 72% yield). Quaterarylene **7**_**2**_ was obtained in 72% yield, by dimerization reaction of iodobiarylene **24**, performed through a Miyaura borylation reaction followed by a Suzuki–Miyaura cross-coupling, with no isolation of the boronic ester intermediate. Similarly, the preparation of sexiarylene **7**_**4**_ required the dimerization of iodoterarylene **10**, which was prepared through a Suzuki–Miyaura cross-coupling reaction between diiodobispivalamide **22** and boron pinacolato **25** (obtained through Miyaura borylation from iodobiarylene **24**). All Piv-protected oligoarylenes underwent acid-catalyzed hydrolysis to obtain the respective polyaminoarylenes (**8**_***y***_, *y* = 1–4), in yields ranging from 44% to quantitative. Mesitylation of the polyaminoarylenes through Buchwald–Hartwig reaction^46^ with MesBr gave terarylene **9**_**1**_, quaterarylene **9**_**2**_, quinquearylene **9**_**3**_, and sexiarylene **9**_**4**_ in 56–86%, with an average yield per each newly formed C–N bond of 94–97%. Final planarization of the *meta*-oligoarylenes was performed through *N*-directed borylation with BBr_3_, exploiting the conditions developed for preparing NBN-dibenzophenalene derivative **6**. Terarylene **9**_**1**_ was planarized with 15 equiv of BBr_3_ in *o*-DCB at 170 °C. Terminal *B*-alkylation was performed with MeMgBr to give final BN-doped *peri*-acenoacene **1**^**Me**^ in 82% yield over two steps (62% overall yield over seven steps). While the final molecule stability allowed isolation and growth of single crystals suitable for X-ray diffraction experiments (see Supporting Information (SI), section 4), in-solution ^1^H NMR characterization showed the occurrence of decomposition. It is suggested that molecule **1**^**Me**^ is extremely susceptible to O_2_, likely undergoing oxidation at the Me-B sites, forming Me–O–B functions that could further hydrolyze into the corresponding borinic acid-type derivative.

Thus, *B*-substituted phenyl derivative **1**^**Ph**^ was prepared (76% yield over two steps, 58% overall yield over seven steps) using PhMgBr. ^1^H NMR investigations showed that the molecule is stable under atmospheric conditions, and thus a decision was made to install the Ph substituents on the terminal *B*-centers for all BN-doped *peri*-acenoacenes.

**Figure 3:**
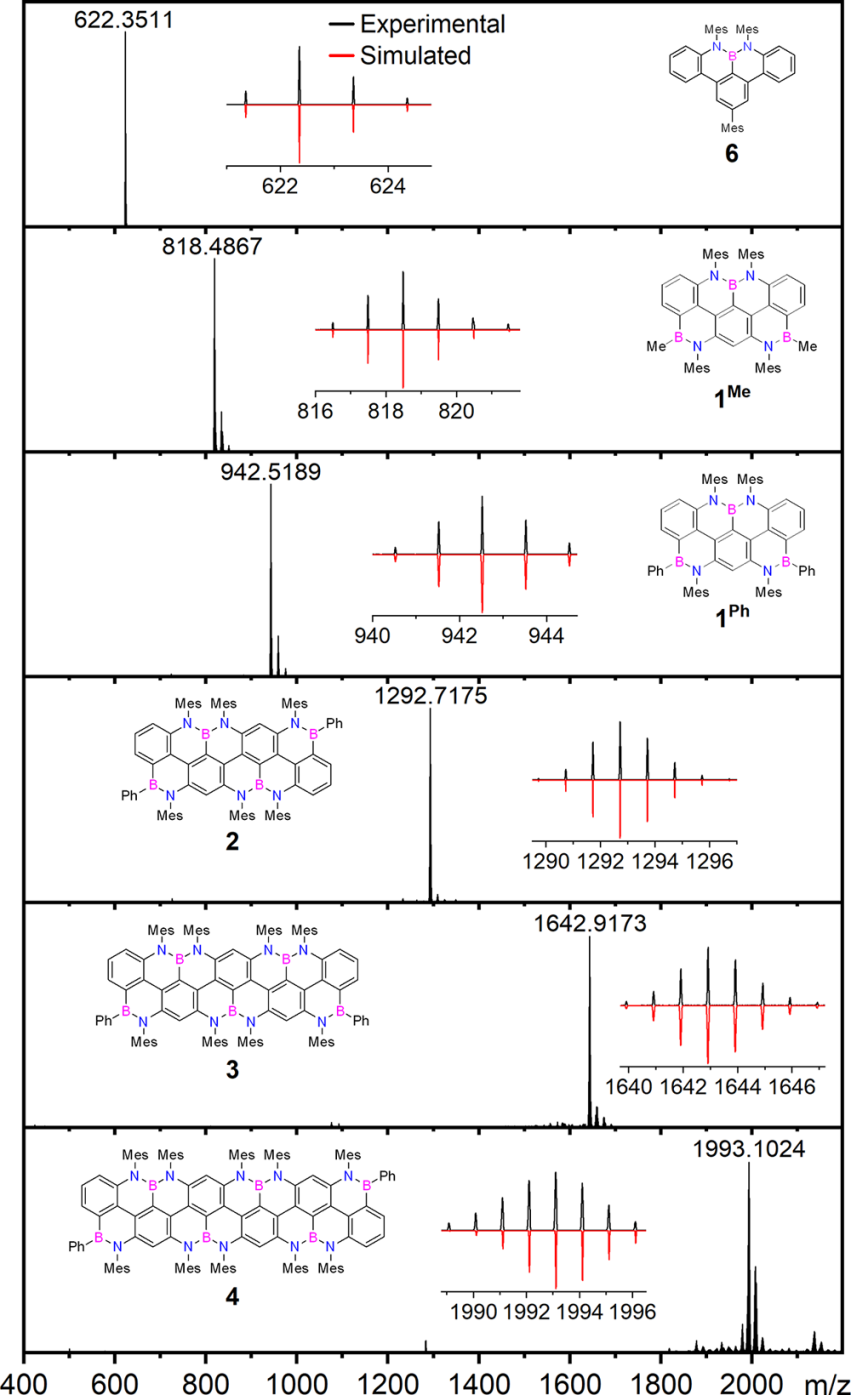
HR LD MS spectra for the BN-doped nanoribbons **1**^**Me**^, **1**^**Ph**^, **2**, and **3** and HR MALDI MS (Matrix: DCTB) spectra for **6** and **4**. Inset: experimental (black) and simulated (red) isotopic pattern of the M^+^ peak. Satellite high-mass peaks correspond to the [M+O]^+^ and [M+2O]^+^ adducts, presumably formed during the ionization.

To our surprise, full borylation of quaterarylene **9**_**2**_ at 170 and 200 °C gave, respectively, no product and an 8% yield of **2**. However, when the reaction mixture containing quaterarylene **9**_**2**_ was heated at 230 °C (in Schlenk tube with PTFE screwcap charged with 30 equiv of BBr_3_), BN-quaterarylene **2** could be isolated in 51% yield after reaction with PhMgBr. Taken all together, these observations suggested that, upon elongation of the *meta*-oligoaminoarylene precursor, a progressive increase in the temperature (around 40–50 °C per additional boron atom) was necessary to achieve full planarization. Thus, when reacting quinquearylene **9**_**3**_ with BBr_3_ at 270 °C, BN-quinquearylene **3** could be isolated with a 46% yield after the addition of PhMgBr. Finally, full borylation-induced planarization of BN-sexiarylene **4** could be obtained only at 330 °C (60 °C higher when compared to the case of BN-quinquearylene **3**). Notably, all nanoribbons displayed propensity to aggregate and limited solubility in classical organic solvents. While terarylene **1**^**Ph**^ could be spectroscopically characterized by ^1^H NMR in CDCl_3_ (in CD_2_Cl_2_ extensive broadening of the hydrogen resonance was observed), NMR spectra for quaterarylene **2** could be only obtained in a 1:1 mixture of CD_2_Cl_2_ and CS_2_ (solubility in CD_2_Cl_2_ <1 mg/mL). Moreover, NMR characterization of quinquearylene **3** was performed with a 1:1 mixture of C_6_D_6_ and CS_2_. The new BN-*peri*-acenoacenes were unambiguously identified by HRMS (Figure 3) through the detection of the peak corresponding to the molecular ions, at *m*/*z* 942.5189 (M^+^, C_66_H_61_B_3_N_4_, calc.: 942.5198), 1292.7175 (M^+^, C_90_H_84_B_4_N_6_, calc.: 1292.7168), 1642.9173 (M^+^, C_114_H_107_B_5_N_8_, calc.: 1642.9139), and 1993.1024 (M^+^, C_138_H_130_B_6_N_10_, calc.: 1993.1103) for molecules **1**^**Ph**^, **2**, **3**, and **4**, respectively. All nanoribbons displayed sufficient thermal and chemical stability to be isolated and fully characterized with one-dimensional and two-dimensional ^1^H, ^13^C, and ^11^B NMR spectroscopies, and single-crystal X-ray diffraction analysis, except for derivative **4**, which displayed high O_2_-susceptibility and insolubility in organic solvents, that prevented us from performing a full NMR characterization. While the ^1^H and ^13^C resonances had no signals characterizing the effectiveness of the planarization reaction, with ^11^B NMR we could fingerprint the BN and NBN moieties at *ca*. 39 and 27 ppm, respectively. In the case of terarylene **1**^**Ph**^, the ^11^B resonances appeared as two distinct peaks at 39.0 and 26.6 ppm for, respectively, the BN and NBN motifs. In the extended frameworks quaterarylene **2** and quinquearylene **3**, the two resonances instead overlap, progressively displaying the BN-centered peak as a shoulder (at 39.1 ppm for **2** and 38.3 ppm for **3**) to the highest-intensity NBN-centered resonance (at 27.6 ppm for **2** and 27.9 ppm for **3**).

### X-ray Characterization

**Figure 4:**
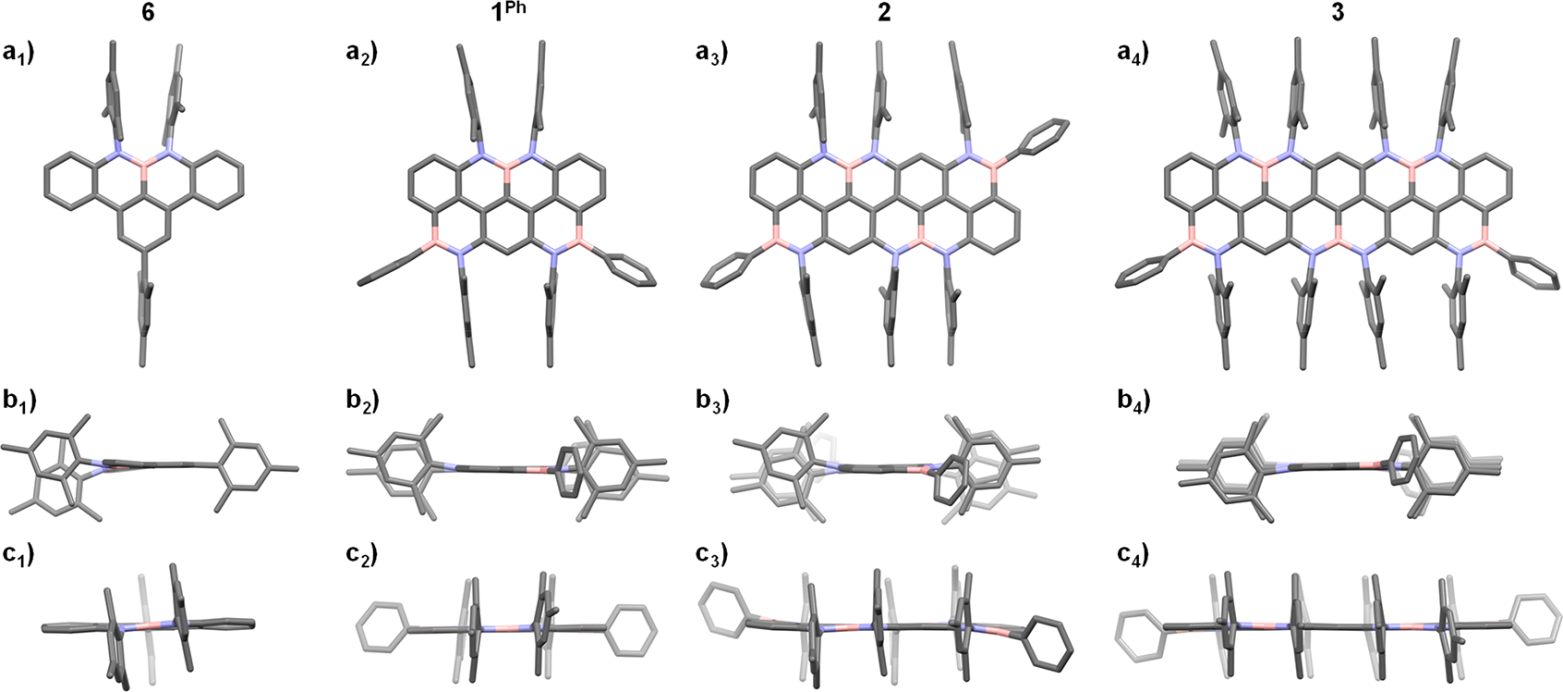
Single-crystal X-ray structures for **6**, **1**^**Ph**^, **2**, and **3**. (a) Top view; (b) short-side view; (c) long-side view. Space groups: *P*2_1_/*n* (**6**), *P*1 (**1**^**Ph**^), *P*1 (**2**), *P*1 (**3**). Crystallization solvents: CHCl_3_/MeOH (**6**), toluene/MeOH (**1**^**Ph**^), CH_2_Cl_2_/CH_3_CN (**2**), toluene/MeOH (**3**). Hydrogen atoms and solvent molecules are omitted for clarity. For compound **1**^**Ph**^, a single, crystallographically independent, molecule representative of the crystal is shown. Atom colors: gray, C; pink, B; blue, N.

Systematic X-ray diffraction investigations of single crystals of compounds **5**^**Me**^, **5**^**Ph**^, **5**^**Mes**^, **6**, **1**^**Me**^, **1**^**Ph**^, **2**, and **3** confirmed, upon borylation, the formation of BN and NBN motifs and the progressive π-extension of the polyaromatic framework along the ribbon series (Figure 4). All derivatives were crystallized by the vapor diffusion method, using a low-temperature boiling liquid antisolvent to generate the vapors, except for **5**^**Ph**^, whose single crystals were grown by thermal crystallization with the aid of a Technobis Crystal16 instrument. X-ray diffraction analysis of single crystals of **6** (solvent: CHCl_3_, antisolvent: MeOH) showed a nearly planar structure of the NBN-dibenzophenalene scaffold (maximum deviation from planarity: 6.0°), with each azaborine ring depicting a slight distortion, likely originated by repulsion between the *peri*-*N*-mesityl groups (Figure 4). The B–N bond lengths of 1.438 and 1.448 Å denote a partial double bond character, as previously observed with other peripherally doped NBN-containing PAHs.^22,31,32,54,55^ X-ray analysis of terarylene **1**^**Ph**^ (solvent: toluene, antisolvent: MeOH) showed two crystallographically independent molecules, both displaying a planar polyaromatic core (maximum deviation from planarity: 1.7°–2.8°), in the asymmetric unit, and B–N bond lengths for the NBN unit (1.438–1.450 Å) consistent with those measured for derivative **6**. The peripheral azaborine-like B–N bond lengths fall within the 1.413–1.421 Å range, suggesting a distinct double bond character that is peculiar to 9,10-azaboraphenanthrenes^56−59^ (see SI, section 4, for **5**^**Me**^, **5**^**Ph**^, and **5**^**Mes**^). Molecule **2** (solvent: CH_2_Cl_2_, antisolvent: CH_3_CN), possessing an inversion center, shows an S-shaped bending of the ribbon structure (maximum deviation from planarity: 6.4°) driven by the out-of-plane *B*-phenyl rings (dihedral angle: 69.4°). The B–N bond lengths for the NBN fragments (1.443 and 1.452 Å) and the terminal BN couples (1.413 Å) are in line with the aforementioned values for the smaller derivatives. Finally, BN-quinquearylene **3** (solvent: CH_2_Cl_2_, antisolvent: CH_3_CN) possesses an essentially planar backbone (maximum deviation from planarity: 1.9°) and slightly shorter B–N bonds (1.427–1.450 Å for NBN moieties, 1.408 and 1.414 Å for terminal BN bonds) than the smaller nanoribbons. Notably, all compounds bear Mes groups that are oriented nearly perpendicularly to the π-conjugated BN-doped *peri*-acenoacene core. The solid-state organization is generally governed by the interdigitation of the bulky hydrophobic Mes groups, and no significant π–π stacking or BN-driven dipole–dipole interactions could be detected.

### UV–vis Absorption and Emission Spectroscopic Investigations

**Figure 5:**
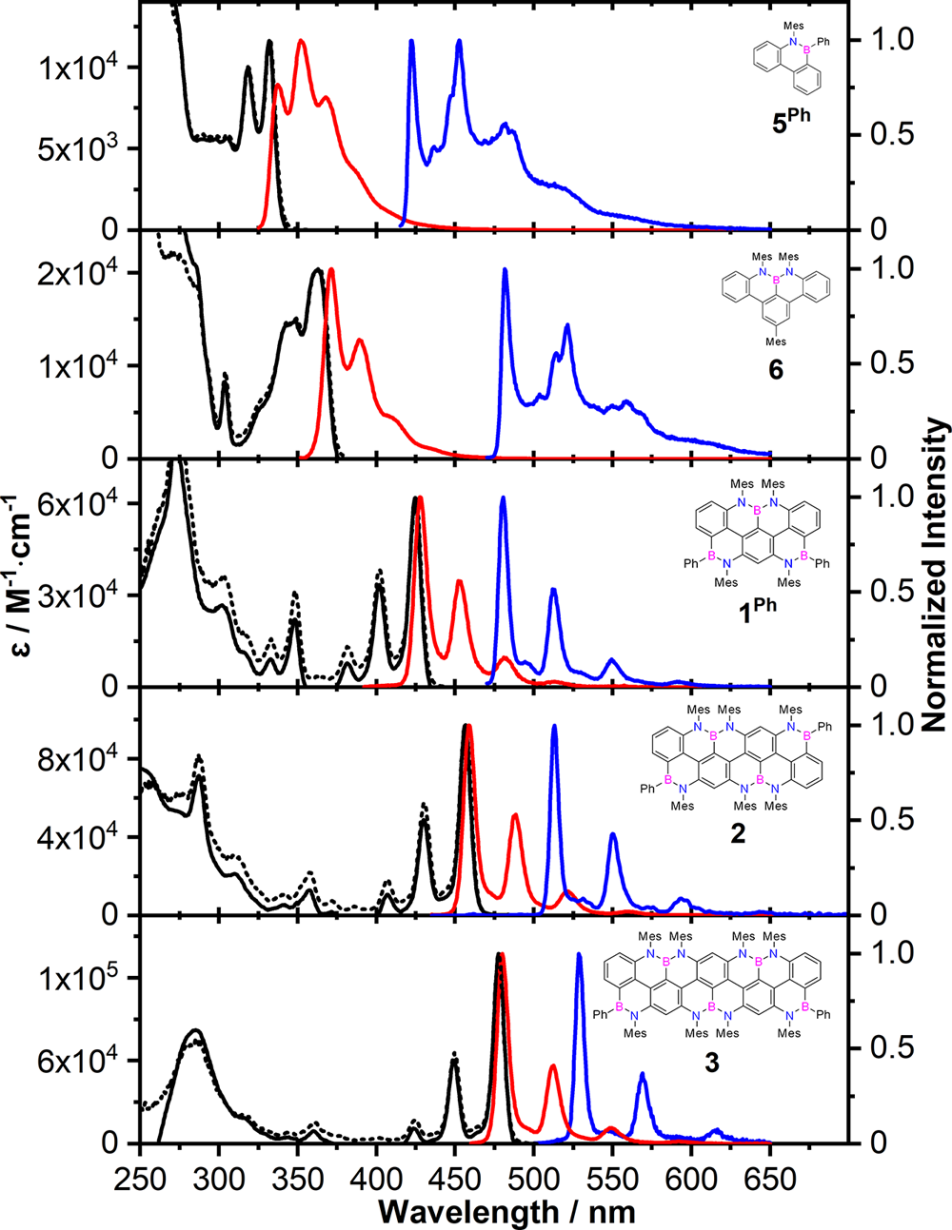
Absorption (black), normalized fluorescence (red), and excitation (dotted) spectra of **5**^**Ph**^, **6**, **1**^**Ph**^, **2**, and **3** in 2-MeTHF at rt. Normalized phosphorescence (blue) at 77 K.

UV–vis absorption and emission properties (Figure 5 and Table 3) of BN-doped *peri*-acenoacenes **1**^**Ph**^, **2**, **3**, and their reference molecules **5**^**Ph**^ and **6** were studied in 2-methyltetrahydrofuran (2-MeTHF). All compounds were measured immediately after their isolation and structural characterization. The spectral envelopes of ribbons **1**^**Ph**^, **2**, and **3** are similar to that of reference **6**, displaying pronounced vibronic substructures. As revealed by TD-DFT calculations, the absorption bands are characterized by π → π* transitions (see SI, section 5). As evidenced in Figure 5, the absorption maximum λ_max_ bathochromically shifts upon increasing the framework length, from the shortest nanoribbon up to the longest, with the lowest-energy electronic transition centered at 425, 457, and 478 nm for **1**^**Ph**^, **2**, and **3**, respectively. Reference molecules **5**^**Ph**^ and **6** exhibited similar electronic transitions, centered at 332 and 363 nm, respectively. Notably, NBN-doped phenalene **6** possesses an energy transition 0.5 eV higher (363 nm) than that of **1**^**Ph**^ (425 nm), suggesting that the double peripheral BN-fusion strongly contributes to lowering the transition energy.

**Table 3:** Summary of Computational and Photophysical Properties of BN-Doped *peri*-Acenoacene **1**^**Ph**^, **2**, and **3** and Their Reference Molecules **5**^**Ph**^ and **6** in 2-MeTHF

	Absorbance			Fluorescence				Phosphorescence^a^				Energy			
	*ε*	λ_max_	λ_em_			τ_PF_	τ_PF_^d^	τ_DF_^d^	λ_em_	τ_PH_	*ΔE*_ST_^e^	*E*_g_^00^^f^	*E*_g_^opt^^g^	*E*_S_0_ → S_1__^T^^h^	*E*_g_^HL^^i^
	(M^–1^·cm^–1^)	(nm)	(nm)	Φ_PF_^b^	Φ_PF_^c^^,^^d^	(ns)	(ns)	(μs)	(nm)	(s)	(eV)	(eV)	(eV)	(eV)	(eV)
**5**^**Ph**^	11614	332	338	0.48	0.56	3.6 (rt)	3.7 (rt)	nd	418	6.0	0.74	3.71	3.73	4.21/3.92	4.75
						3.7 (77 K)									
6	20309	363	372	0.55	0.74	4.4 (rt)	4.3 (rt)	nd	477	6.2	0.78	3.38	3.42	3.60/3.38	3.95
						4.6 (77 K)									
**1**^**Ph**^	61354	425	428	0.71	0.91	3.6 (rt)	3.2 (rt)	138.3	472	2.2	0.27	2.90	2.92	3.14/2.94	3.54
						3.3 (77 K)	3.2 (77 K)								
**2**	97157	457	459	0.84	1	3.0 (rt)	2.6 (rt)	639.7	508	3.5	0.25	2.69	2.71	2.87/2.69	3.25
						2.9 (77 K)	2.4 (77 K)								
**3**	137101	478	480	0.86	0.91	2.0 (rt)	2.1 (rt)	387.4	523	3.1	0.21	2.58	2.59	2.70/2.53	3.07
						1.8 (77 K)	1.9 (77 K)								

a At 77 K.

b Absolute quantum yield.

c Relative quantum yield.

d Degassed solution.

e Δ*E*_ST_ = *E*_S_ – *E*_T_, singlet (*E*_S_) and triplet (*E*_T_) energies from the fluorescence and phosphorescence spectra at 77 K.

f Energy of the 0–0 transition obtained from the crossing point between the excitation and emission spectra.

g Optical bandgap: 1240/λ_max_.

h Calculated energy for the S_0→_S_1_ transition, from TD-DFT, at the B3LYP/6-311G*/CPCM optimized S_0_/S_1_ geometry in 2-MeTHF.

i Calculated HOMO–LUMO gap value at the B3LYP/6-311+G** optimized geometry in CH_2_Cl_2_. nd = not detected. rt = room temperature.

Consistently, the steady-state emission spectra of air-equilibrated solutions of the BN-ribbons in 2-MeTHF (Figure 5) reflect the same trend as that observed with UV–vis absorption investigations, with envelopes displaying strong vibronic structures and mirroring the absorption profiles. Specifically, the main emission peak of quinquearylenyl derivative **3** is centered at 480 nm (Φ_PF_ = 0.86, τ_PF_ = 2.0 ns) and is significantly red-shifted with respect to that of the quaterarylenyl (**2**) and terarylenyl (**1**^**Ph**^) derivatives (λ_em_ = 459 and 428 nm, τ_PF_ = 3.0 and 3.6 ns, respectively). Notably, all derivatives display high fluorescence quantum yields (Φ_PF_ = 0.71, 0.84, and 0.86 for **1**^**Ph**^, **2**, and **3**, respectively) and very small Stokes shifts, suggesting reduced vibrational changes or solvent reorganization upon excitation. Taken all together, these findings indicate that upon elongation of the BN-doped *peri*-acenoacene scaffold, the optical band gaps (*E*_g_^00^) of **5**^**Ph**^ to **6**, **1**^**Ph**^, **2**, and **3** progressively shrink from 3.71 to 3.38, 2.90, 2.69, and 2.58 eV, respectively. Considering the small Stokes shifts of the molecules studied in this paper, we use *E*_g_^00^ as a reference value when discussing the optoelectronic properties.

When cooled to 77 K, two independent emissions with different lifetimes were observed and assigned to fluorescence and phosphorescence. The phosphorescence emission (Figure 5) shows a tetra-peak emission profile for all derivatives, with the envelopes being progressively red-shifted when passing from *peri*-acenoacene **1**^**Ph**^ to **2** and **3** (λ_em_ = 472, 508, and 523 nm, τ_PH_ = 2.2, 3.5, and 3.1 s, respectively). Notably, the introduction of the lateral BN bonds had almost no effect on the phosphorescent energy transition for BN-doped terarylene **1**^**Ph**^, with the latter displaying an almost identical energy transition centered around 472 nm compared to 477 nm for NBN-dibenzophenalene **6**. On the other hand, the phosphorescence lifetime (τ_PH_) of molecule **6** is almost three times longer than that of **1**^**Ph**^, likely suggesting that the extension of the framework impacts the nonradiative deactivation pathways of the T_1_ excited states. As expected, no major changes in the fluorescence emission properties were observed at 77 K, with only a slight narrowing of the peaks being detected, caused by further inhibition of thermal deactivation pathways.

**Figure 6:**
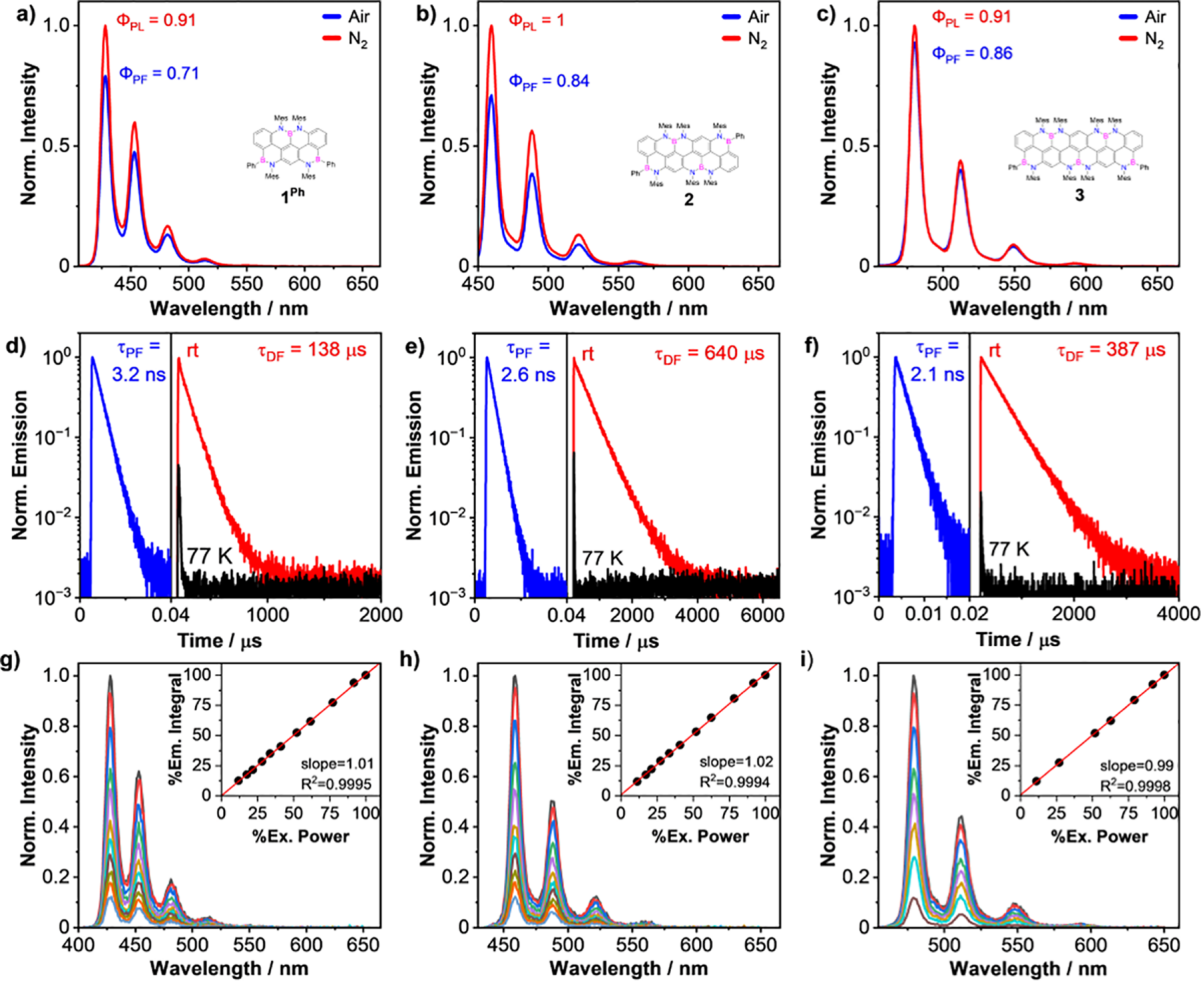
Steady-state emission profiles (a–c) of **1**^**Ph**^, **2**, and **3**, measured in 2-MeTHF at rt in air-equilibrated (blue traces) and in degassed (red traces) solutions. The quantum yield of the degassed solutions was estimated from the relative emission intensity and the previously determined absolute quantum yield for the air-equilibrated solutions. Fluorescence decays (d–f) of degassed solutions in 2-MeTHF, highlighting the PF component at rt (blue traces) and the delayed emission at rt (red traces) and 77 K (black traces). (g–i) Gated (200 μs) fluorescence emission spectra of **1**^**Ph**^, **2**, and **3**, measured in degassed 2-MeTHF at rt, attenuated with a series of neutral density filters (OD 0.03–1). Inset: Linear dependence of the integrated delayed fluorescence on the excitation power.

Based on the low-temperature emission profiles, we calculated the energy difference between the S_1_ and T_1_ excited states (Δ*E*_ST_), obtaining the values of 0.27, 0.25, and 0.21 eV for the *peri*-acenoacenes **1**^**Ph**^, **2**, and **3**, respectively. On the other hand, reference molecules **5**^**Ph**^ and **6** display a relatively high singlet–triplet band gap of 0.74 and 0.78 eV, respectively. Thus, considering the Δ*E*_ST_ value of 0.3 eV as the threshold at which one could observe thermally activated delayed fluorescence (TADF),^60^ we hypothesized that our BN-*peri*-acenoacenes could also possess a delayed emissive component. Being a phenomenon involving the formation of a triplet state, with a lifetime usually in the range of μs–ms, TADF is typically heavily quenched by triplet O_2_ (triplet–triplet annihilation, TTA). For this reason, it is safe to assume that delayed fluorescence (DF) is totally quenched in air-equilibrated solutions and only prompt fluorescence (PF) is observed. To evaluate the presence of such a delayed component, we measured the emission properties of solutions containing the BN-derivatives in O_2_-free conditions (Figure 6).

A strong long-living delayed fluorescence component was detected for *peri*-acenoacenes **1**^**Ph**^, **2**, and **3**, with τ_DF_ values of 138.3, 639.7, and 387.4 μs, respectively. Notably, all short-living fluorescence components have very similar τ_PF_ values to those measured in the presence of O_2_, indicating that no significant O_2_-quenching occurs at rt. As expected, both reference molecules **5**^**Ph**^ and **6** displayed no delayed fluorescence.

Interestingly, under O_2_-free conditions, the quaterarylene derivative **2** exhibited a quantitative fluorescent emission (Φ_PL_ = 1), suggesting that the deactivation of the excited states does not involve any radiationless pathway. Similarly, all BN-*peri*-acenoacenes experienced a considerable increase in their photoluminescent (PL) quantum yield. Given that molecules **5**^**Ph**^ and **6** displayed no TADF emission, the gain in fluorescent emission upon O_2_ removal cannot be attributed to the recovery of energy from the triplet state (Δ*E*_ST_ > 0.7 eV). Considering that the quenching of excited singlet states of aromatics can be favored by an O_2_-catalyzed intersystem crossing, we tentatively explain the increase of PL quantum yield under O_2_-free conditions as a consequence of an inefficient intersystem crossing.^61−63^

Due to technical limitations of our time-resolved fluorescence spectrometer (i.e., our nanopulsed (55 ps) light source is too weak (0.11 mW) to obtain any noticeable TADF emission above the detector background noise (in MCS mode), and our μflash lamp has a pulse width (2.5 μs) that overlaps with the PF emission), we could not experimentally estimate the kinetics of intersystem crossing, reversed intersystem crossing, and all radiationless pathways. Nevertheless, we confirmed the presence of TADF by performing low-temperature measurements under O_2_-free conditions. As expected, the delayed emission decays were completely quenched at low-temperature (Figure 6d–f), giving yield to the phosphorescent emission previously observed (see SI, section 3.1). Quenching of the TADF was sometimes noticed with aged samples, which featured extensive aggregation when redissolved in solution. The delayed emission could be restored upon heating the solution (see SI, Figures S196–199). To unequivocally assign the delayed fluorescence to a TADF process, we investigated the dependence of the delayed emission component against the excitation power.^64,65^ The excitation power was attenuated by placing a series of neutral density filters (OD 0.03–1) between the excitation monochromator and the sample holder, and the delayed emission was measured by gating the detector (200 μs delay) to exclude the prompt fluorescence component. Using the total integrated delayed emission, recorded without attenuation, as a reference for the emission with 100% of irradiation power, we plotted the relative percentage of the integrated emission recorded at different attenuation levels as a function of the corresponding filter transmittance at the given excitation wavelength (Figure 6g–i). A linear proportionality with a slope close to 1 was found for all solutions containing the nanoribbons, suggesting that the observed delayed fluorescence exclusively originates from an intramolecular single photon intersystem process, consistent with TADF. A mechanism relying on the collision of two triplet excited molecules (i.e., TTA) would display a quadratic dependence, which has not been observed here.^66^

### Electrochemical Investigations

**Figure 7:**
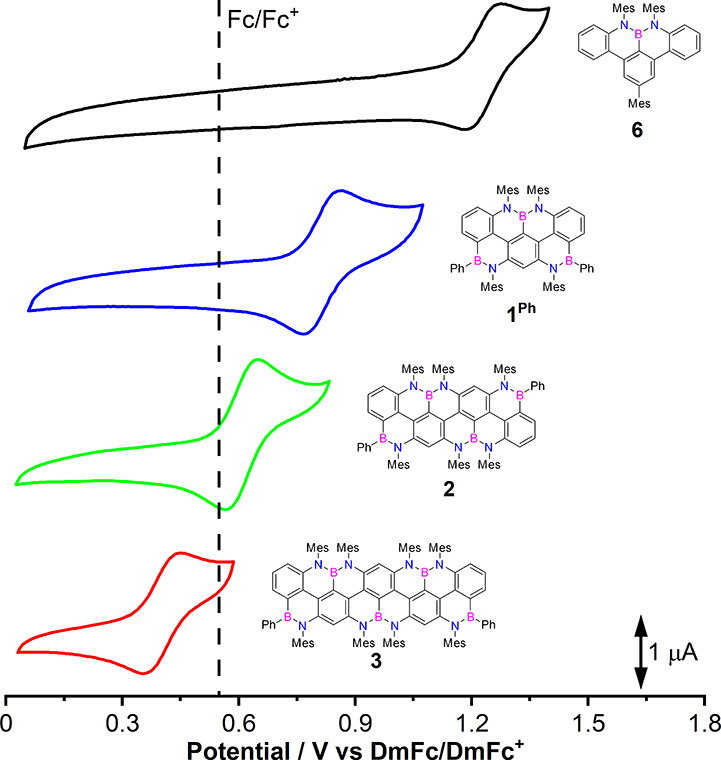
Cyclic voltammetry of molecules **6**, **1**^**Ph**^, **2**, and **3** (0.2 mM). Scan rate: 60 mV/s. Solvent: *o*-DCB/CH_3_CN (4:1) for **6**, **2**, and **3**; TCE for **1**^**Ph**^. Supporting electrolyte: TBAPF_6_. Working electrode: 7 mm^2^ platinum disk. Counter electrode: Platinum wire. DmFc is used as an internal reference standard. The *E*_1/2_^ox^ for the Fc/Fc^+^ (—) couple is shown for comparison purposes.

Next, we examined the redox properties of all derivatives by cyclic voltammetry (CV) measurements using the redox couple Decamethylferrocene/Decamethylferrocenium (DmFc/DmFc^+^) as internal reference (Table 4, Figure 7). Initial studies in *o*-DCB/CH_3_CN (4:1) displayed a first one-electron reversible oxidation process (*E*_1/2_^ox^) for reference molecule **6** and nanoribbons **2** and **3**, while reference molecule **5**^**Ph**^ and nanoribbon **1**^**Ph**^ only exhibited irreversible oxidation waves. It is hypothesized that in the case of molecule **5**^**Ph**^ and nanoribbon **1**^**Ph**^ chemically unstable radical cations undergoing further decomposition were formed. Building on the experimental procedure developed by Xie et al. for probing the reversible electrooxidation of C_60_,^67^ we performed the CV studies of these molecules in 1,1,2,2-tetrachloroethane (TCE). Interestingly, we observed a one-electron reversible oxidation process for molecule **1**^**Ph**^ (*E*_1/2_^ox^ = 0.83 V vs DmFc/DmFc^+^), while molecule **5**^**Ph**^ still displayed irreversible oxidation (*E*_onset_^ox^ = 1.46 V vs DmFc/DmFc^+^) under our experimental conditions (Figure S202).

**Table 4:** Oxidation Potentials and the Associated Frontier Molecular Orbital Energies

Molecule	*E*_1/2_^ox^^a^(V vs DmFc/DmFc^+^)	*E*_HOMO_^b^ (eV)	*E*_LUMO_^c^ (eV)
**5**^**Ph**^	1.46^d^	–5.72	–2.01
**6**	1.23 (60.4 mV)	–5.49	–2.11
**1**^**Ph**^	0.83 (70.5 mV)	–5.09	–2.19
**2**	0.62 (65.5 mV)	–4.88	–2.19
**3**	0.40 (70.5 mV)	–4.66	–2.08

a Half-wave potentials were calculated as *E*_1/2_ = (*E*_pa_ + *E*_pc_)/2 by considering anodic (*E*_pa_) and cathodic (*E*_pc_) peak potentials, unless otherwise specified. Values in parentheses are referred to the peak separation ((*E*_pa_ – *E*_pc_) of each reversible process.

b Calculated using the formula *E*_HOMO_ = −(*E*_1/2_^ox^ - 0.54)- 4.8.

c Calculated using the optical bandgap: *E*_LUMO_ = *E*_HOMO_ + *E*_g_^00^.

d Potential taken from the onset of an irreversible peak.

Upon lateral BN-type extension, as in the case of terarylenyl derivative **1**^**Ph**^, the oxidation potential significantly decreases to 0.83 V. By increasing the ribbon length to the quaterarylenyl (**2**) and quinquearylenyl (**3**) derivatives, the *E*_1/2_^ox^ further reduces to 0.62 and 0.40 V, respectively. Notably, the elongation of the nanoribbon of one *meta*-arylenyl unit accounts for a negative shift of the oxidation potential of *ca*. 0.20 V. Following this trend, we can expect a very low oxidation potential of *ca*. 0.20 V for nanoribbon **4**, which could explain its high susceptibility toward O_2_. Upon increasing the potential above the first oxidation wave, a second oxidation event was observed, although, under our experimental conditions, it was not reversible (Figures S207–210). No reduction processes were observed. Together with the photophysical data (*E*_g_^00^, see also Table 3), electrochemical data allowed us to estimate the energies of the HOMO and LUMO frontier orbitals, spanning from −5.72 (HOMO) and −2.01 eV (LUMO) for **5**^**Ph**^ to −4.66 (HOMO) and −2.08 eV (LUMO) for **3** (Figure 10). The extension of the polyaromatic framework along the ter-, quater-, quinque-, and sexi-arylenyl series provokes a rise in the HOMO energy level, progressively making the nanoribbon a stronger electron donor (i.e., *p*-type semiconductor).

**Figure 8:**
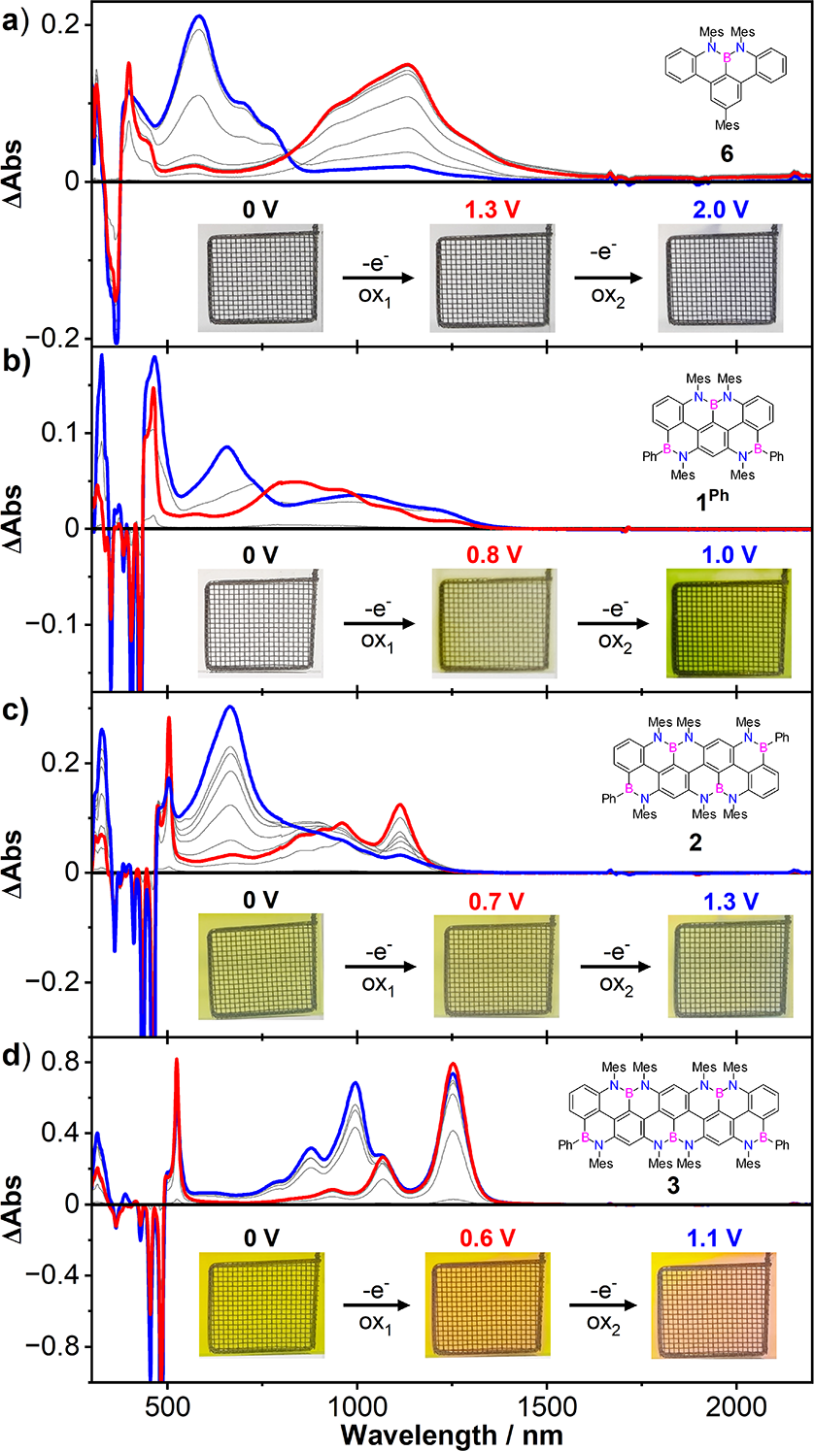
UV–vis–NIR absorption spectra measured during the electrochemical oxidation of **6** (a), **1**^**Ph**^ (b), **2** (c), and **3** (d). **1**^**Ph**^ was measured in TCE, while **6**, **2**, and **3** were measured in a 4:1 mixture of *o*-DCB/CH_3_CN. Potentials vs Ag/AgCl.

To shed further light on the optoelectronic properties of the oxidized species, we performed *in situ* spectroelectrochemical (SEC) measurements of all nanoribbons **1**^**Ph**^, **2**, and **3** and reference molecule **6**. Upon increasing the potential from 0 V to the experimentally determined oxidation potentials (*E*_1/2_^ox^, Table 4), a gradual appearance of a new set of bands in the UV–vis–NIR region was observed, accompanied by the concomitant decrease of the initial absorption bands centered at 363, 425, 457, and 478 nm for **6**, **1**^**Ph**^, **2**, and **3**, respectively (Figure 8a–d).

Notably, a new sharp absorption peak with a small red shift (0.20–0.25 eV) was observed in the UV–vis region for all molecules, centered at 398, 462, 503, and 524 nm, for **6**, **1**^**Ph**^, **2**, and **3**, respectively. At the same time, a large bathochromic shift (850–1250 nm) into the NIR region was observed, with broad absorption bands resembling the neutral molecule, consistent with the formation of a monoradical cationic species. The optical band gap of the newly formed species was inferred from the onset of the lowest energy absorption band and found to be 0.94, 1.04, 0.94, and 0.95 eV for **6**, **1**^**Ph**^, **2**, and **3**, respectively, considerably lower than that of the neutral molecules (Table 3). One can notice that the cation of molecule **1**^**Ph**^ is isoelectronic to the all-C benzo[*c*]-anthanthrenyl radical derivatives recently synthesized by Wu and co-workers.^13^ When comparing the UV–vis–NIR absorption profiles, it is possible to see that all three species have the lowest electronic transitions in the NIR region; however, a direct comparison could not be made, since the benzo[*c*]-anthanthrenyl radicals feature TIPSe and 3,5-di-*tert*-butylphenyl groups that significantly change their absorption spectra.

Further increasing the potential toward the second oxidation event led to the appearance of a new set of absorption bands at higher energy, with clear isosbestic points centered at 755, 815, 936, and 1073 nm for **6**, **1**^**Ph**^, **2**, and **3**, respectively. The existence of isosbestic points for all molecules suggests that the monoradical cation species interconverts into a newly further oxidized species. Notably, for quinquearylenyl derivative **3**, both radical cation and dication species exhibit similar spectral envelopes to that of the neutral species, with the absorption bands being broader most likely due to the charge-transfer nature of the electronic transitions in the oxidized species. However, given the irreversible nature of the second oxidation, any discussion about the electronic properties of the dication species and their comparison with all-C congeners would be highly speculative.

### Theoretical Investigations

**Figure 9:**
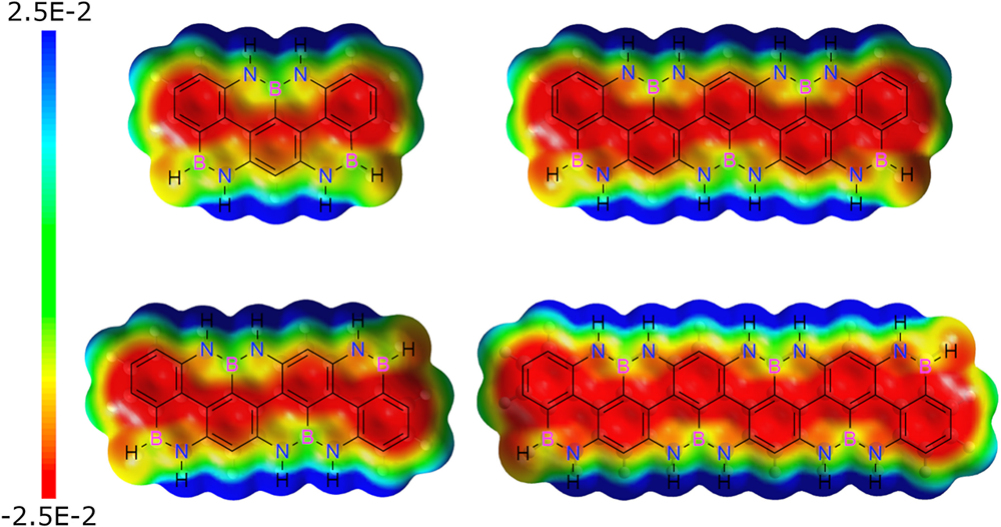
ESP mapped on the van der Waals surface up to an electron density of 0.001 electron·bohr^–3^.

To appraise the effect of the BN doping on the aromatic π-surface, we further determined the charge distribution of the BN-nanoribbons in the form of an electrostatic potential (ESP) map (Figure 9) calculated with Gaussian 16 at the B3LYP/6-311+G** level of theory (see SI, section 5).^68^ To optimize the computational time, all calculations were performed with ribbons in which the *N*-Mes and the *B*-Ph moieties were substituted by H atoms. As previously observed with the hexa-*peri*-hexabenzoborazinocoronene,^43^ the presence of the BN bonds induces a great charge polarization of the π-surface, with positive and negative partial charges located on B and N atoms, respectively. At the same time, the π-surface containing the *meta*-oligoarylenyl framework displays partial negative charges due to its quadrupolar nature. These results are consistent with the expected ambipolar character of the molecule.^69^

**Figure 10:**
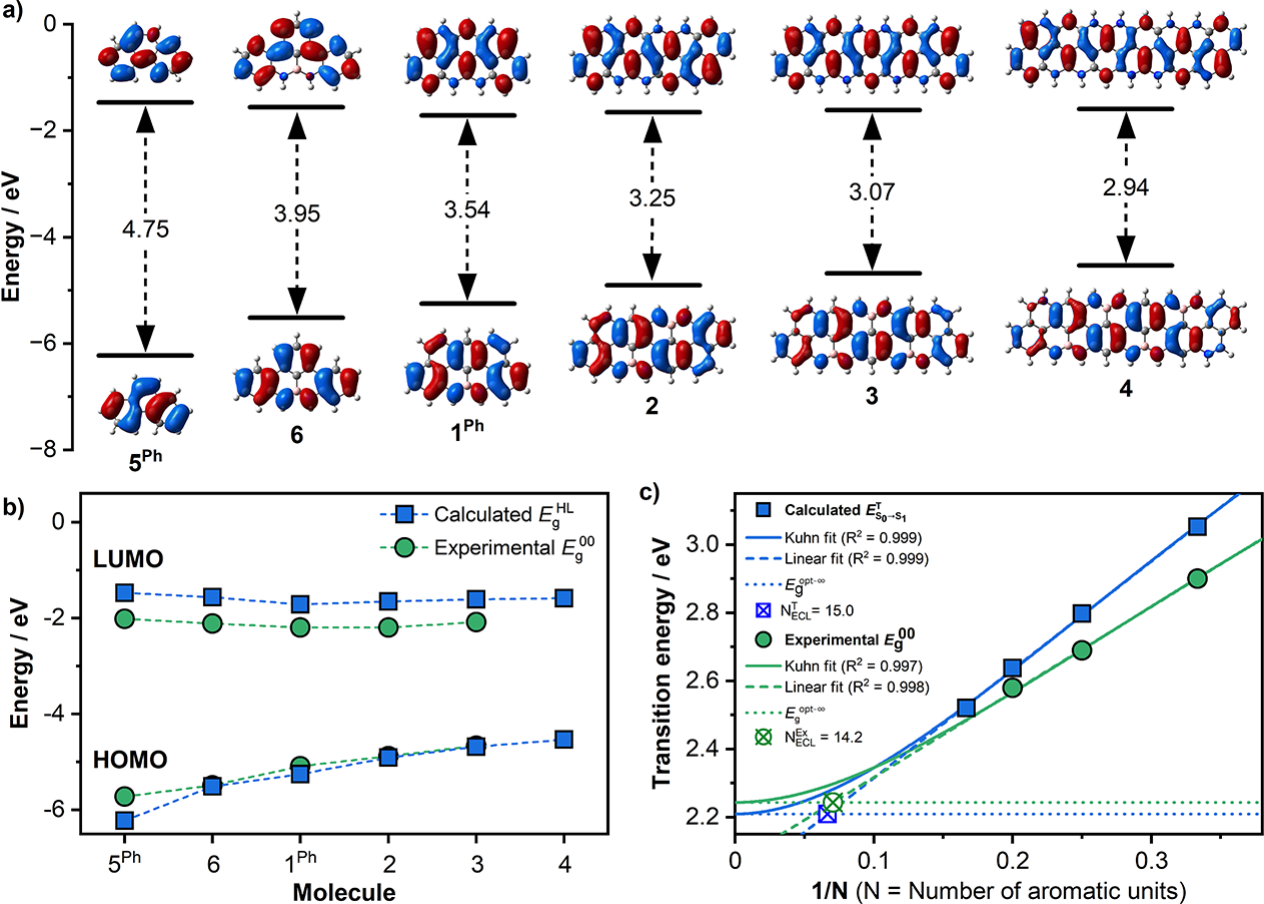
(a) Calculated frontier orbital energy levels for molecules **5**^**Ph**^, **6**, **1**^**Ph**^, **2**, **3**, and **4** (with Mes groups substituted by H atoms) together with their HOMO and LUMO profiles at B3LYP/6-311+G** level of theory (Gaussian16); (b) comparison between the HOMO and LUMO energy levels as estimated experimentally from the CV and photophysical data (green circles, Table 4) and theoretically (blue squares); (c) calculated absorption maxima (TD-DFT, circle) and experimental optical bandgaps (square) as a function of 1/*N*, with *N* being the number of *meta*-linked aryls within the framework. Solid lines are fits using the Kuhn equation and dashed lines using a linear equation. Dotted lines represent the *y*-intercept (*E*_*g*_^opt-∞^) of the Kuhn fit. *N*_ECL_^Ex^ and *N*_ECL_^T^ are determined from the interception of the linear fit with the *y*-intercept of the Kuhn fit.

To shed further light on the structure–property relationship, we also calculated the HOMO and LUMO orbitals for all nanoribbons (Figure 10). It transpires that both orbitals are homogeneously distributed on the π-surface, with the LUMOs preferentially located on the B atoms and the B-deactivated positions, whereas the HOMOs on the N atoms and N-activated aromatic positions. Notably, the HOMOs are never located on the B atoms of the NBN motifs (Figure 10a). Taken all together, these results suggest that the one-electron oxidation events for all ribbons are likely to be confined to the N atoms and N-activated aryl rings. Furthermore, Time-Dependent (TD) calculations of the theoretical UV–vis absorption spectra (S_0_ → S_1_) remarkably trace the experimental profiles, unambiguously confirming the bathochromic shift upon increasing the nanoribbon length from **1**^**Ph**^ to **3**. As one can notice from the values calculated by TD-DFT, the theoretical optical gaps are only slightly blue-shifted compared to those measured experimentally, showing very good accuracy. To estimate the extent to which one can modulate the optical absorption of these BN-doped ribbons by structural extension (Figure 10c), we have attempted to assess the effective conjugation length (ECL) using the Kuhn fit.^70,71^ Although we have only a few experimental *E*_g_^00^ values, we estimated the *N*_ECL_^Ex^ of our BN-nanoribbons to be around 14, in good agreement with that obtained from the calculated TD-DFT spectra (*N*_ECL_^T^ = 15). Interestingly, the *E*_*g*_^opt-∞^ is estimated to fall in the 2.2–2.3 eV interval, suggesting that a further shrinking of the optical band gap of 0.3–0.4 eV is still achievable with these BN-doped *peri*-acenoacene architectures.

To investigate the origin of the TADF, we have performed theoretical investigations to calculate the excited-state properties with TD-DFT (Table 5). These calculations, together with the UV–vis absorption spectra and the fact that the HOMO and the LUMO are not spatially segregated (Figure 10a), show no S_1_/T_1_ charge-transfer states. Moreover, by analyzing the spin–orbit couplings of higher-excited states (up to S_10_/T_10_) with either S_1_ or T_1_, we did not find any state pairs with large spin–orbit couplings (all below 0.1 cm^–1^, see Tables S43–S46), not suggesting any additional states besides the S_1_ and T_1_ to take part in the TADF mechanism in our molecules.^72^ This situation might change when nuclear vibrations displace the molecules from their planar S_1_/T_1_ geometries, increasing considerably the previously symmetry-forbidden spin-orbit couplings.^73^

**Table 5:** S_1_ and T_1_ Excitation Energies, Δ*E*_*S*_1_ – *T*_1__ Energy Gaps, for the B3LYP/6-311G*/CPCM(2-MeTHF) Optimized S_0_, S_1_, and T_1_ Geometries of Molecules **5**^**Ph**^, **6**, **1**^**Ph**^, **2**, and **3** (with Mes Groups Substituted by H Atoms)

	Optimized S_0_			Optimized S_1_			Optimized T_1_		
	*E*_*S*_1__ (eV)	*E*_*T*_1__ (eV)	Δ*E*_*S*_1_ – *T*_1__ (eV)	*E*_*S*_1__ (eV)	*E*_*T*_1__ (eV)	Δ*E*_*S*_1_ – *T*_1__ (eV)	*E*_*S*_1__ (eV)	*E*_*T*_1__ (eV)	Δ*E*_*S*_1_ – *T*_1__ (eV)
**5**^**Ph**^	4.21	3.38	0.83	3.92	3.08	0.84	3.91	2.71	1.21
**6**	3.60	2.83	0.77	3.38	2.53	0.85	3.33	2.36	0.97
**1**^**Ph**^	3.14	2.58	0.56	2.94	2.34	0.60	2.93	2.32	0.61
**2**	2.87	2.39	0.48	2.69	2.18	0.51	2.69	2.16	0.53
**3**	2.70	2.29	0.41	2.53	2.11	0.42	2.53	2.10	0.44

Considering that TADF requires small singlet–triplet Δ*E*_ST_ gaps and high S_1_ radiative rates (i.e., the fluorescence rate from the S_1_ must outcompete the radiative and nonradiative relaxation from the T_1_) to happen, it is apparent that in our case it is the extension of the π-conjugated backbone of these nanoribbons (as confirmed by both experimental and theoretical observations, Table 3 and 5, respectively) that causes both shrinking of the Δ*E*_ST_ and shortening of the τ_PF_.

This is in full resonance with our experimental observations, for which only TADF for molecules **1**^**Ph**^, **2**, and **3** (Δ*E*_ST_ < 0.3 eV) has been detected, whereas no delayed emissive components were found for reference derivatives **5**^**Ph**^ or **6** (Δ*E*_ST_ > 0.7 eV) (Table 3). TADF induced by extension of the π-conjugation has been already observed for polymer structures,^65,74,75^ and although our systems are not polymeric, we propose that the same π-conjugation effect gives rise to the TADF properties of our BN-doped *peri*-acenoacenes. Considering the estimated *N*_ECL_^T^ value of 15, we think that there is still room to reduce the Δ*E*_ST_ gap. Thus, future studies will be attempted in our group to extend the π-conjugation of these architectures and further investigate the effect on the TADF properties.

## Conclusion

In this work, the first series of regularly BN-doped molecular ribbons, featuring *peri*-acenoacene topologies, was developed *via* borylation-induced planarization of amino-bearing *meta*-oligoarylenyl precursors, through the formation of B–C and B–N bonds with BBr_3_. The protocol was optimized using BN-phenanthrenes and NBN-dibenzophenalenes as chemical models, achieving 93–97% average yield per B–N/B–C bond formed. For the borylation of the *meta*-oligoaminoarylenes (prepared by sequential Suzuki-type cross-coupling reactions), a substantial increase in the reaction temperature (*ca*. 50 °C per additional ring) proved necessary upon increasing the length of the oligoarylenyl framework, spanning from 170 to 230, 270, and 330 °C for achieving ter-, quater-, quinque-, and sexi-arylenyl nanoribbons, respectively. The resulting zigzag-edged ribbons were analyzed by NMR, HRMS, and single-crystal X-ray diffraction, except for the sexi-arylenyl derivative that, displaying high susceptibility toward O_2_ and insolubility in organic solvents, was characterized solely by HR MALDI MS. To the best of our knowledge, these molecules possess the highest doping ratio (∼30%) reported so far for discrete BN-doped graphenoid-type molecules. Photophysical investigations showed a progressive bathochromic shift of the UV–vis absorption and emission spectra upon extension of the polyaromatic framework in the ribbon series, with the optical band gap shrinking from 2.90 to 2.69 and 2.58 eV for the ter-, quater-, and quinque-arylenyl BN-ribbons, respectively. All derivatives displayed high fluorescence quantum yields in solution (between 71% and 86%) and phosphorescence emission at 77 K. O_2_-free measurements revealed the existence of a TADF emission at room temperature for the ter-, quater-, and quinque-arylenyl derivatives, with the quaterarylenyl ribbon featuring a quantitative fluorescence yield. Supported by both experimental and theoretical investigations, the presence of the TADF component is likely to be originated from the extension of the π-conjugation, which causes both shrinking of the Δ*E*_ST_ and shortening of the τ_PF_. Electrochemical studies showed that π-extension of the *peri*-acenoacene framework provokes a lowering of the first oxidative event (from 0.83 to 0.40 V) and a rise in the HOMO energy level, progressively making the nanoribbon a stronger electron donor. Preliminary attempts to assess the ECL showed a projected effective conjugation length of around 14, which would give an *E*_*g*_^opt-∞^ of 2.2–2.3 eV. Taken together, these findings suggest that the nanoribbons prepared in this work can be optimal candidates to engineer *p*-type organic semiconductors, with optical band gaps falling in the blue and green region of the visible spectrum. Future studies in this field from our group will focus on the development of graphenoid nanoribbons displaying higher doping ratios, different doping patterns, topologies, and further π-extensions to study the effects of these structural parameters on the molecular optoelectronic properties, with a particular focus on the delayed emission characteristics.

## Supporting Information Available

Synthetic procedures, NMR and HRMS spectra, photophysical and electrochemical characterizations, crystallographic data, computational studies. (PDF)
