# Supplementary material for: peri-Acenoacene Ribbons with Zigzag BN-Doped Peripheries
Source: J Am Chem Soc. 2022 Nov 17;144(47):21470–84. doi: 10.1021/jacs.2c06803 (PMC9716572; doi:10.1021/jacs.2c06803)
Supplement: Supplementary file 1 — ja2c06803_si_001.pdf [file ja2c06803_si_001.pdf]

Supporting Information

Peri-acenoacene ribbons with zig-zag BN-doped peripheries

Marco Franceschini,‡[a] Martina Crosta,‡[a] Rúben R. Ferreira,‡[a] Daniele Poletto,[a] Nicola Demitri,[b] J. Patrick Zobel,[c] Leticia González[c] and Davide Bonifazi*[a]

[a] Institute of Organic Chemistry, Faculty of Chemistry, University of Vienna, Währinger Strasse 38, 1090, Vienna, Austria; email: davide.bonifazi@univie.ac.at.

[b] Elettra – Sincrotrone Trieste, S.S. 14 Km 163.5 in Area Science Park, 34149 Basovizza, Trieste, Italy.

[c] Institute of Theoretical Chemistry, Faculty of Chemistry, University of Vienna, Währinger Straße 17, 1090, Vienna, Austria.

‡ These authors contributed equally.

Table of contents

1. General remarks 2
   1.1. Instrumentation 2
   1.2. Materials and methods 7
2. Synthetic procedures 8
2. NMR and HRMS spectra 36
3. Optoelectronic characterization 100
   3.1. Photophysical properties 100
   3.2. Electrochemical properties 139
4. Crystallographic data 147
5. Computational studies 155
6. References 196

1. General remarks

1.1. Instrumentation

Thin layer chromatography (TLC) was conducted on pre-coated aluminum sheets with 0.20 mm Merck Millipore Silica gel 60 with fluorescent indicator F254. TLC plates were visualized by exposure to ultraviolet light (254 or 366 nm).

Column chromatography was carried out using Merck Gerduran silica gel 60 (particle size 40-63 µm).

Preparative high-performance liquid chromatography (HPLC) was performed on an Agilent 1260 Infinity Preparative LC System, equipped with a reverse phase column (Varian Pursuit C18, 5 µm, 250 X 21.2 mm). Eluent system: water added with 0.1% TFA (solvent A), CH_3CN (solvent B) in all cases. Elution with isocratic mixture for 3 min (90% solvent A, 10 % solvent B), then gradient elution to reach 100% of solvent B in 30 min. Flow rate: 17 mL/min.

Melting points (mp) were measured on a Leica Galen III microscope equipped with a heating block and a Hg thermometer (T_{max} = 200 °C) on a microscope slide, or a DigiMelt MPA 160 (T_{max} = 260 °C) on open capillary tubes, under air, and are uncorrected. According to the limitations of the apparatus, the compounds which did not melt or decompose (dec) up to 200 or 260 °C are presented as "> 200 °C" or "> 260 °C".

Nuclear magnetic resonance (NMR) characterizations were performed at the NMR centre of the University of Vienna. NMR spectra were recorded on Bruker spectrometer AV III HD 700, AV III 600 or AV NEO 400. ^{1}H NMR spectra were obtained at 700, 600 or 400 MHz, ^{13}C NMR spectra at 176, 151 or 101 MHz, ^{11}B NMR spectra at 193 MHz in quartz NMR tube. All spectra were obtained at room temperature. Carbon spectra were acquired with a complete decoupling for the proton. Proton and carbon chemical shifts are reported in parts per million (ppm, δ scale) according to tetramethylsilane ($\delta_H = \delta_C = 0$ ppm) using the solvent residual signal as an internal reference ($CDCl_3$: δ_H = 7.26 ppm, δ_C = 77.16 ppm; DMSO-*d_6*: δ_H = 2.50 ppm, δ_C = 39.52 ppm; C_6D_6: δ_H = 7.16 ppm, δ_C = 128.06 ppm; CD_2Cl_2: δ_H = 5.32 ppm, δ_C = 54.00 ppm). Boron chemical shifts are reported in ppm, referenced to the external standard boron signal of $BF_3 \cdot Et_2O$ (δ_B = 0 ppm). Coupling constants (*J*) are given in Hz. Resonance multiplicity is described as s (singlet), d (doublet), dd (doublet of doublets), ddd (doublet of doublets of doublets), t (triplet), td (triplet of doublets), q (quartet), p (pentet), m (multiplet) and bs (broad signal).

Infrared spectra (IR) were recorded on a Bruker Alpha FT-IR spectrometer in ATR mode. Selected absorption bands are reported in wavenumbers (cm^{-1}).

High-resolution mass spectrometry (HRMS) analyses were performed at the Mass Spectrometry Centre of the University of Vienna. ESI mass spectra were obtained on a Bruker maXis UHR ESI-Qq-

TOF mass spectrometer in the positive ion mode, GC mass spectra on an Agilent 7200B GC/Q-TOF mass spectrometer, LD and MALDI mass spectra on a Bruker Autoflex Speed LD-timsTOF or MALDI-timsTOF (matrix: 2-[(2E)-3-(4-tert-butylphenyl)-2-methylprop-2-enylidene]malononitrile (DCTB)) mass spectrometer.

Ultraviolet-Visible (UV-Vis) **absorption spectroscopy** was recorded on Agilent Cary 5000 UV-Vis-NIR Spectrophotometer running in double beam mode with a matched pair of quartz absorbance cuvettes (1 x 1 cm). All absorption measurements were performed at 21 °C unless specified otherwise. The absorbance of oxygen-free solutions was measured using a special built quartz cuvette (see Figure S192). The molar attenuation coefficient (ε) was determined by dissolving a known amount of compound (typically 1 – 2 mg) in 2-MeTHF and diluting the resulting stock solution to achieve 5 solutions with an appropriate concentration for measurements (absorbance < 0.5). The plot of absorbance versus concentration was fitted with a linear function and the molar attenuation coefficient obtained from the slope.

UV-Vis emission spectroscopy: The photoluminescence (PL) excitation and emission spectra, absolute quantum yield, and decay curves were recorded on a FLS1000 photoluminescence spectrometer (Edinburgh Instruments, UK). The spectrometer was equipped with excitation and emission double grating Czerny-Turner monochromators, a photomultiplier detector with extended near-infrared sensitivity (PMT-980), fitted with a gating circuit and thermoelectrically cooled to -20 °C with a fan-assisted Peltier element, and a High Speed PMT detector with a response width < 180 ps operating at 0 °C. All samples were prepared in air-equilibrated extra dry 99+% 2-methyltetrahydrofuran (Thermo Fisher, stabilizer free), unless otherwise stated. The maximum absorbance of all solutions was adjusted to < 0.1 to avoid inner filter effect. For oxygen-free measurements, the solutions where degassed by performing multiple cycles of freeze-pump-thaw (typically 5), and the headspace was filled with nitrogen (≥ 99.999%). Low temperature (77 K) measurements were carried out using an Optistat DN liquid nitrogen cryostat (Oxford Instruments, UK) fitted in the FLS1000 spectrometer sample chamber. The samples were placed in a special built cryogenic 1x1 cm quartz cuvette (see Figure S192) and held in a static nitrogen atmosphere. Glassy matrix was achieved by slowly lowering the temperature (20 °C/min). For steady-state measurements, the samples were excited using a 450 W ozone-free continuous Xenon arc lamp. Time-resolved measurements in the ns range were performed by irradiating the samples with a suitable nano-pulsed LED (EPLED-295) or laser (EPL-405) and acquired using the High Speed PMT detector in Time-Correlated Single Photon Counting (TCSPC) mode. For decays in the µs - ms range, a 60 W microsecond pulsed Xenon flash lamp was used as light source and the measurement performed using the PMT-980 detector in Multi-Channel Scaling (MCS) mode. For decays over 10 s, the 450 W continuous Xenon arc lamp was used in conjunction with the software-controlled excitation shutter to create a 0.01 s light pulse, using the PMT-980 detector in kinetic scan mode (0.02 s time resolution). The tail portion of the decay curves were fitted using the FAST software (Edinburgh Instruments, UK), following a single exponential model with y-offset (background-offset):

$$I(t) = A + B \cdot e^{\frac{-t}{\tau}} \tag{1}$$

where A is the y-offset, B the pre-exponential factor, and τ the lifetime. For decays close to the pulse width of the light source, the instrument response function (IRF) was measured using a Ludox® solution (at room temperature) or using the sample itself (at low temperature). In both cases the count rate was adjusted using a computer-controlled neutral density filter wheel in order to match the count rate of the sample emission. In these cases, the decay lifetime was obtained by performing a reconvolution fit using the FAST software. Absolute quantum yields were measured using an integrating sphere (internal diameter 120 mm) fitted on the FLS1000 sample chamber. The samples and blank reference (solvent) were placed in a 1 x 1 cm fluorescence quartz cuvette and the calculations were done using the "direct excitation" method following the equation:

$$\Phi = \frac{E_B - E_A}{S_A - S_B} \tag{2}$$

where E_B and E_A correspond to the integrated fluorescence emission of the sample and blank reference (solvent), respectively. S_A and S_B refer to the integrated excitation scatter region of the reference and the sample, respectively. For measuring the scatter region, and avoid detector saturation, a neutral density filter (OD = 1) was placed between the integrating sphere exit and the detector in order to attenuate the signal. A fixed excitation bandwidth of 3 nm was used to ensure the determination of the sample absorption with high accuracy (step = 0.1 nm), while the emission bandwidth was chosen in order to obtain a strong sample emission signal (peak emission > 10^4 cps). The quantum yield of oxygen-free solutions was determined using the relative method, following the equation:

$$\Phi_F = \Phi_R \cdot \frac{I_F}{I_R} \cdot \frac{A_R}{A_F} \tag{3}$$

where ϕ_R is the quantum yield of the air-equilibrated solution, determined using the absolute method, I_F and I_R are the integrated emissions of the degassed and air-equilibrated solutions, respectively, and A_R and A_F are the absorbance of the solution before and after degassing, respectively. The optical bandgap E_g^{00}, was calculated using the intercept of the excitation and emission spectra (λ_{00}) following equation:

$$E_g^{00}(\mathrm{eV}) = 1240/\lambda_{00} \tag{4}$$

Alternatively, the optical bandgap was also calculated using the absorbance maximum λ_{max}:

$$E_g^{opt}(\mathrm{eV}) = 1240/\lambda_{max} \tag{5}$$

Electrochemical analysis: Cyclic voltammetry experiments were performed at room temperature in a mixture of freshly distilled *o*-DCB/CH_3CN 4:1 or in TCE, using an Autolab PGSTAT204 potentiostat (Metrohm, DE). A conventional three-electrode electrochemical cell connected to an argon source and an oil bubbler was used. Dry argon gas was bubbled through the sample solution for at least 15 min prior to each measurement and the headspace was continuously flushed throughout the experiment. A pre-bubbler filled with solvent was used in order to prevent evaporation. Platinum

disk (3 mm diameter) was used as a working electrode, Pt wire as auxiliary electrode, and an Ag/AgCl electrode as reference.

The platinum working electrode was polished on a pad using alumina slurry and washed with deionized water before each experiment; the Pt wire was flame-cleaned. Tetrabutylammonium hexafluorophosphate (Alfa Aesar, $TBAPF_6$) was twice recrystallized from absolute ethanol prior to use and it was added to the solution as a supporting electrolyte at a concentration of 0.1 M. Decamethylferocene (Sigma Aldrich) or Ferrocene (Sigma Aldrich) was used as an internal reference. The formal redox potentials (half-wave potentials) were calculated using the formula:

$$E_{1/2} = \frac{E_{\mathrm{pa}} + E_{\mathrm{pc}}}{2} \quad (6)$$

where E_{pa} is the peak anodic potential and E_{pc} is the peak cathodic potential. The energy of the HOMO was estimated using the following equation:

$$E_{\mathrm{HOMO}}(\mathrm{eV}) = -\left(E^{\mathrm{ox}}_{1/2}\right) - 4.8 \quad (7)$$

where E_{ox} is the half-wave oxidation potential (vs Fc/Fc^+) and 4.8 is the HOMO energy of ferrocene in vacuum. To reference the oxidation potentials vs Fc/Fc^+, we experimentally determined the potential of the $DmFc/DmFc^+$ redox couple to be -0.54 V vs Fc/Fc^+.

Spectroelectrochemical characterization was performed using a thin layer quartz cuvette (path length of 1 mm) equipped with an optically transparent platinum minigrid working electrode, platinum wire auxiliary electrode, and an Ag/AgCl reference electrode.

X-ray measurements of **5^{Me}**, **5^{Ph}** **5^{Mes}**, **6**, **1^{Me}**, **1^{Ph}**, and **2** were performed at the Centre for X-ray Structure Analysis of the University of Vienna. X-ray intensity data were measured at 100 K on a STOE Stadivari diffractometer equipped with dual radiation source Mo and Cu Kα, and a Dectris EIGER2 R 500K detector. The structures were solved *ab initio* and refined by full-matrix least-squares techniques. Hydrogen atoms were inserted at calculated positions using AFIX instructions, while all other atoms were refined with anisotropic displacement parameters. **5^{Mes}** was refined with isotropic displacement parameters because of the molecular symmetry (B and N are interchangeable) and subsequent disorder in the crystal lattice. The following softwares were used: STOE software package for collecting crystal data and image processing, STOE LANA for scaling and absorption correction,[1] SHELXT-2018/2 for structure solution,[2] SHELXL-2018/3 for structure refinement,[3] SHELXLE version 1378 and OLEX2-1.5 as graphical user interfaces.[4,5] Data collections for **3** were performed at the XRD2 beamline of the Elettra Synchrotron, Trieste (Italy).[6] The crystals were dipped in NHV oil (Jena Bioscience, Jena, Germany) and mounted on the goniometer head with kapton loops (MiTeGen, Ithaca, USA). Complete datasets were collected at 100 K (nitrogen stream supplied through an Oxford Cryostream 700) through the rotating crystal method. Data were acquired using monochromatic wavelength of 0.620 Å on Pilatus hybrid-pixel area detectors (DECTRIS Ltd., Baden-Daettwil, Switzerland). The diffraction data were indexed, integrated and scaled using XDS.[7] Two different datasets, collected from random orientations of the same crystal, were merged to obtain complete sets of data, using CCP4-Aimless code.[8,9] Semi-empirical absorption corrections and scaling were performed on the datasets, exploiting multiple measures

of symmetry-related reflections, using SADABS.[10] The structures were solved by the dual space algorithm implemented in the SHELXT code.[2] Fourier analysis and refinement were performed by the full-matrix least-squares methods based on F^2 implemented in SHELXL (Version 2018/3).[3] The Coot program was used for modelling.[11] Anisotropic thermal motion refinement was used for all atoms with full occupancies. Geometry and thermal motion parameters restraints (SIMU, DFIX, SADI and FLAT) were used on disordered solvent molecules. Hydrogen atoms were included at calculated positions with isotropic $U_{factors}$ = 1.2 x U_{eq} or $U_{factors}$ = 1.5 x U_{eq} for methyl groups (U_{eq} being the equivalent isotropic thermal factor of the bonded non-hydrogen atom). Electron density in packing voids filled with heavily disordered solvent molecules could not be modeled and was removed with Platon SQUEEZE[12] routine (68 e-/cell squeezed, corresponding to ~1 toluene molecule/cell in 275 $Å^3$ volume voids). All the samples tested showed severe anisotropic diffraction and spot splitting due to the growth of crystals as stacked thin plates. The model was fully refined as a 2-component non-merohedral twin. Crystal showed two domains related by a 180° rotation around [1 0 1] reciprocal lattice direction (twin fraction refined to 39%). Crystal data, data collection parameters and structure refinement details are given in Tables S1–S8. Structures have been deposited in the Cambridge Structural Database (CSD) with the following deposition numbers: $\mathbf{5^{Me}}$ (2165184), $\mathbf{5^{Ph}}$ (2181763), $\mathbf{5^{Mes}}$ (2165204), **6** (2165185), $\mathbf{1^{Me}}$ (2165187), $\mathbf{1^{Ph}}$ (2165201), **2** (2165203), **3** (2173120). These data are provided free of charge by the joint Cambridge Crystallographic Data Centre and Fachinformationszentrum Karlsruhe Access Structures service via www.ccdc.cam.ac.uk/structures.

Quantum chemical calculations were performed on the Vienna Scientific Cluster (VSC), by using the program package "Gaussian16, Revision A.03".[13] In order to reduce the elapsed time of the calculations, all the mesityl- and phenyl- substituents connected to the main sp^2 chain of each molecule were replaced with hydrogen atoms. The geometry of the input molecules was pre-optimized with the method UFF by using Avogadro. The geometry optimizations were carried out using the hybrid functional B3LYP, in combination with the basis set 6-311+G**. The solvent effect (CH_2Cl_2) was included in the calculation by using the implicit polarizable continuum model (pcm). The energy of the first 64 electronically excited singlet state was calculated using the TD-DFT method, adopting the same functional and basis set used for the geometry optimization. Even in this case, the solvent effect was taken into account by introducing the pcm in the calculation.

To investigate excitation energies, energy gaps between S_1 and T_1 and spin-orbit couplings, we have optimized the geometries of S_0, S_1 and T_1 states. Ground-state geometries have been optimized at the DFT level of theory using the B3LYP functional[14–16] and 6-311G* basis set[17] using the ORCA5.0 program package.[18] For SCF calculations, the resolution-of-the-identity (RIJCOSX) approximation[19] and SARC-J auxiliary basis sets were used.[20] Solvent effects for 2-MeTHF have been simulated using a conductor-like polarizable continuum model (CPCM) with the dielectric constant of 6.97 and a refraction index of 1.407. The first excited singlet and triplet states have been, instead, optimized at the TD-DFT level of theory in the Tamm-Dancoff approximation[21] using the same functional, basis set and solvent model as for the ground state. At both ground state and excited state minima, frequency calculations were performed, and no imaginary frequencies were found, confirming the identity of true minima. At the optimized geometries of the ground state and the lowest triplet

state, the ten lowest excited singlet and triplet states have been calculated including spin-orbit couplings using the mean-field/effective potential approach as implemented in ORCA.

The differences in the computational protocol to the above reported calculations have been introduced for technical reasons. The ORCA program was used to calculate spin-orbit couplings (not available in Gaussian). The ORCA program features only the C-PCM version of the CPM solvent model, which is an approximation of the PCM model. This approximation is justified, except for very non-polar solvents (dielectric constant ε<5,), i.e., also for 2-MeTHF (ε=6.97). Finally, the smaller 6-311G* basis set was used in place of the 6-311+G** basis set, since excited-state geometry optimizations for some of the molecules did not converge using the 6-311+G** basis set.

1.2. Materials and methods

Chemicals were purchased from Sigma Aldrich, Acros Organics, TCI, Alfa Aesar, Fluorochem, Thermo Fisher Scientific and BLDpharm and used without further purification. Mesityl bromide was distilled over CaH_2 and stored in an argon-filled glove box. Anhydrous 1,2-dichloroethane (DCE) and 1,2-dichlorobenzene (*o*-DCB) were purchased from Acros Organics, anhydrous 1,2,4-trichlorobenzene (TCB) was purchased from Sigma Aldrich and were used as received. Anhydrous toluene and tetrahydrofuran (THF) were dried on a MBraun SPS-800 solvent purification system, degassed and stored over activated 4 Å molecular sieves. Deuterated solvents were purchased from Eurisotop. Anhydrous conditions were achieved by drying glassware in oven at 120 °C for at least 12 h and by flaming the reaction vessels with a heat gun under vacuum and purging with argon. The inert atmosphere was maintained using argon-filled balloons equipped with a syringe and needle that was used to penetrate the silicon septa used to close the flask's necks. Addition of liquid reagents was performed using argon-purged plastic or glass syringes. Alternative to the use of Schlenk line techniques, inert conditions were achieved by using an argon-filled MBraun LabStar glove box when stated. Degassing of solutions was performed by bubbling argon or *freeze-pump-thaw* procedure: solutions were frozen in liquid nitrogen and kept under vacuum for 10–15 min before thawing. 0 °C baths were prepared using ice/water. High temperature (> 200 °C) reactions were carried out in sand baths. When heating in closed flask, the reactions were performed in Schlenk tubes filled with argon and opportunely closed with oven-dried glass stoppers and Glindemann PFTE sealing rings for guaranteeing gas-tight joints, or in Schlenk tubes with PTFE screwcap when stated. For the synthesis of **4**, a Schlenk pressure tube with PTFE screwcaps, sealed with a FFKM O-ring, was employed. The reaction vessel was heated on a sand bath using a heating mantle, and the reaction mixture was stirred using a glass-coated magnetic stir bar.

2. Synthetic procedures

6-Methyl-5,6-dihydrodibenzo[*c*,*e*][1,2]azaborinine (11)

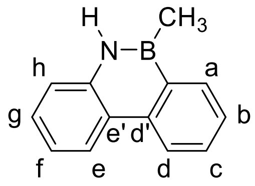

To an oven- and flame-dry 25 mL Schlenk tube, BBr_3 (1 M in hexanes, 1.0 mL, 1.0 mmol) was added dropwise to a stirring solution of dry 2-aminobiphenyl (**12**, 85 mg, 0.5 mmol) in dry DCE (5 mL) at 0 °C. The white suspension was stirred at 60 °C for 18 h under argon atmosphere. After allowing the yellow-green solution to reach room temperature, five *freeze-pump-thaw* cycles were performed. MeMgBr (3 M in diethyl ether, 0.67 mL, 2.0 mmol) was added dropwise at 0 °C and the pale-yellow solution was stirred at room temperature for 1 h. The mixture was quenched by dropwise addition of water (30 mL) and extracted with CH_2Cl_2 (3 x 50 mL). The combined organic layers were dried over Na_2SO_4, and the solvents evaporated *in vacuo*. The resulting orange solid was purified by silica gel column chromatography (petroleum ether/EtOAc 99:1) affording **11** as a white solid (87 mg, 90% yield over two steps). The solid was further purified by precipitation in MeOH/H_2O to provide the analytically pure product.

mp 101–103 °C. ^{1}H NMR (700 MHz, $CDCl_3$) δ 8.43 (dd, *J* = 8.3, 1.1, 1H, H_a), 8.39 (dd, *J* = 8.2, 1.3, 1H, H_e), 8.16 (dd, *J* = 7.5, 1.6, 1H, H_d), 7.73 (ddd, *J* = 8.3, 7.2, 1.6, 1H, H_b), 7.52 (ddd, *J* = 7.5, 7.2, 1.1, 1H, H_c), 7.47 (bs, 1H, N-H), 7.40 (ddd, *J* = 8.0, 7.1, 1.3, 1H, H_g), 7.23 (ddd, *J* = 8.2, 7.1, 1.3, 1H, H_f), 7.20 (dd, *J* = 8.0, 1.3, 1H, H_h), 1.01 (s, 3H, B-CH_3). ^{13}C NMR (176 MHz, $CDCl_3$) δ 139.1 (C-N), 138.2 ($C_{d'}$), 134.7 (C_d), 130.9 (C_b), 128.0 (C_g), 126.1 (C_c), 124.0 (C_e), 123.5 ($C_{e'}$), 122.2 (C_a), 121.3 (C_f), 118.8 (C_h) (two signals are missing due to ^{11}B-induced quadrupolar relaxation). ^{11}B NMR (193 MHz, $CDCl_3$) δ 39.5. IR (cm^{-1}): 3380, 3369, 1602, 1557, 1513, 1488, 1436, 1421, 1330, 1313, 1279, 1231, 1188, 1159, 1138, 1046, 1005, 941, 883, 865, 820, 799, 755, 723, 697, 682, 617, 565, 530, 515, 487, 430. HRMS (GC/Q-TOF): *m/z* calcd for $C_{13}H_{12}BN^+$: 193.1058 [M]$^+$; found: 193.1055.

N-hexyl-[1,1'-biphenyl]-2-amine (13)

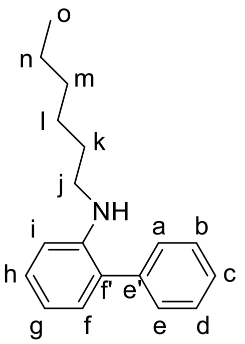

To a 25 mL round-bottom flask, hexanal (0.12 mL, 1.0 mmol), $NaBH_3CN$ (69 mg, 1.1 mmol) and acetic acid (0.5 mL, 8.7 mmol) were successively added to a stirring solution of 2-aminobiphenyl (**12**, 169 mg, 1.0 mmol) in CH_3CN (4 mL) at 0 °C. The yellow solution was stirred at room temperature for 18 h. After solvent removal *in vacuo*, the mixture was diluted with sat. aq. $NaHCO_3$ (30 mL) and extracted with EtOAc (3 x 40 mL). The combined organic layers were dried over $MgSO_4$, and the solvent evaporated *in vacuo*. The resulting yellow oil was purified by silica gel column chromatography (petroleum ether/EtOAc 99:1) affording **13** as a yellow oil (211 mg, 83% yield). The product was further purified by distillation over CaH_2 (0.05 mbar, 170 °C) for subsequent borylation reaction (pale yellow liquid, density: 0.958 g/mL).

^1H NMR (600 MHz, $CDCl_3$) δ 7.49–7.41 (m, 4H, H_a, H_b, H_d, H_e), 7.39–7.33 (m, 1H, H_c), 7.28–7.21 (ddd, *J* = 8.0, 7.4, 1.6, 1H, H_h), 7.10 (dd, *J* = 7.4, 1.6, 1H, H_f), 6.76 (dd, *J* = 7.4, 7.4, 1H, H_g), 6.71 (d, *J* = 8.0, 1H, H_i), 3.90 (bs, 1H, N-H), 3.10 (td, *J* = 7.0, 4.3, 2H, H_j), 1.55 (p, *J* = 7.0, 2H, H_k), 1.31 (m, 6H, H_l, H_m, H_n), 0.89 (t, *J* = 6.7, 3H, H_o). ^{13}C NMR (151 MHz, $CDCl_3$) δ 145.4 (C-N), 139.7 ($C_{e'}$), 130.3 (C_f), 129.5 (C_a, C_e), 129.0 (C_h), 128.8 (C_b, C_d), 127.6 ($C_{f'}$), 127.2 (C_c), 116.7 (C_g), 110.4 (C_i), 44.1 (C_j), 31.6 (C_m), 29.4 (C_k), 26.9 (C_l), 22.7 (C_n), 14.1 (C_o). IR (cm^{-1}): 3422, 3058, 3031, 2955, 2925, 2855, 1603, 1581, 1509, 1490, 1461, 1436, 1377, 1317, 1283, 1193, 1161, 1144, 1072, 1053, 1034, 1008, 994, 930, 916, 842, 769, 735, 701, 615, 562, 486, 430. HRMS (ESI) *m/z* calcd for $C_{18}H_{24}N^+$: 254.1903 $[M+H]^+$; found: 254.1902.

5-Hexyl-6-methyl-5,6-dihydrodibenzo[*c*,*e*][1,2]azaborinine (15)

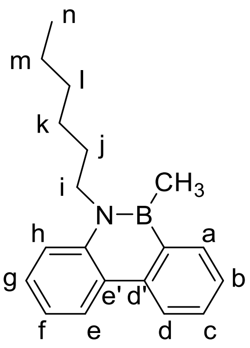

To a flame-dry 25 mL Schlenk tube, BBr_3 (1 M in hexanes, 1.0 mL, 1.0 mmol) was added dropwise to a stirring solution of distilled **13** (0.13 mL, 0.5 mmol) in dry *o*-DCB (5 mL) at 0 °C. The Schlenk tube was equipped with an oven-dry condenser and the yellow solution refluxed at 190 °C for 18 h under argon atmosphere. After allowing the black suspension to reach room temperature, five *freeze-pump-thaw* cycles were performed. MeMgBr (3 M in diethyl ether, 1.0 mL, 3.0 mmol) was added dropwise at 0 °C and the dark brown suspension was stirred at room temperature for 2 h. The mixture was quenched by dropwise addition of water (20 mL) and extracted with CH_2Cl_2 (3 x 20 mL). The combined organic layers were dried over Na_2SO_4, the solvents evaporated *in vacuo*. The resulting brown oil was purified by silica gel column chromatography (petroleum ether/EtOAc 99:1) affording **15** as a white solid (116 mg, 84% yield over two steps).

mp 62–63 °C. ^1H NMR (600 MHz, $CDCl_3$) δ 8.48 (dd, *J* = 8.1, 1.5, 1H, H_e), 8.43 (dd, *J* = 8.3, 0.9, 1H, H_a), 8.21 (dd, *J* = 7.6, 1.5, 1H, H_d), 7.70 (ddd, *J* = 8.3, 7.1, 1.5, 1H, H_b), 7.54 (dd, *J* = 8.6, 1.2, 1H, H_h), 7.49

(m, 2H, H-4, H_g), 7.25 (ddd, *J* = 8.1, 6.9, 1.3, 1H, H_f), 4.15 – 4.06 (m, 2H, H_i), 1.83 – 1.75 (m, 2H, H_j), 1.51 – 1.44 (m, 2H, H_k), 1.43 – 1.32 (m, 4H, H_l, H_m), 1.13 (s, 3H, B-CH_3), 0.98 – 0.89 (m, 3H, H_n). ^{13}C NMR (151 MHz, $CDCl_3$) δ 140.6 (C-N), 137.8 ($C_{d'}$), 134.7 (C_d), 130.7 (C_b), 128.1 (C_g), 126.0 (C_c), 124.7 ($C_{e'}$), 124.5 (C_e), 122.0 (C_a), 120.7 (C_f), 115.7 (C_h), 47.8 (C_i), 31.8 (C_l), 29.7 (C_j), 27.1 (C_k), 22.9 (C_m), 14.2 (C_n) (two signals are missing due to ^{11}B-induced quadrupolar relaxation). ^{11}B NMR (193 MHz, $CDCl_3$) δ 40.3. IR (cm^{-1}): 3061, 3027, 2950, 2921, 2850, 1693, 1602, 1578, 1556, 1488, 1466, 1435, 1372, 1323, 1312, 1286, 1243, 1208, 1192, 1167, 1151, 1139, 1119, 1066, 1027, 1002, 937, 879, 860, 783, 752, 739, 720, 617, 566, 542, 524, 435. HRMS (GC/Q-TOF) *m/z* calcd for $[C_{19}H_{24}BN]^+$: 277.1996 $[M]^+$, found: 277.1996.

N-mesityl-[1,1'-biphenyl]-2-amine (14)

Procedure adapted from the literature.[22]

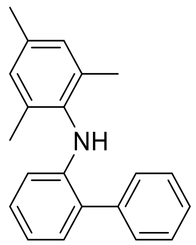

In the glove-box, to a 250 mL Schlenk tube charged with $[Pd_2(dba)_3]$ (28 mg, 0.26 mol%), *rac*-BINAP (55 mg, 0.74 mol%), *t*-BuONa (1.72 g, 17.9 mmol), 2-aminobiphenyl (**12**, 2.52 g, 14.9 mmol), 2-bromomesitylene (1.82 mL, 11.9 mmol) and toluene (24 mL) were added. The mixture was stirred at 110 °C for 20 h in closed flask. After allowing it to reach room temperature, the orange suspension was diluted with sat. aq. NH_4Cl (200 mL) and extracted with EtOAc (2 x 450 mL). The combined organic layers were washed with brine (250 mL), dried over $MgSO_4$, and the solvents evaporated *in vacuo*. The resulting orange oil was purified by silica gel column chromatography (petroleum ether/CH_2Cl_2 9:1) affording **14** as a white crystalline solid (1.85 g, 54% yield). Spectral properties are in agreement with those reported in the literature.[23]

^{1}H NMR (600 MHz, $CDCl_3$) δ 7.60–7.54 (m, 2H), 7.48 (ddd, *J* = 8.6, 6.8, 2H), 7.40–7.36 (m, 1H), 7.17 (dd, *J* = 7.4, 1.6, 1H), 7.08 (ddd, *J* = 8.4, 7.4, 1.6, 1H), 6.92 (s, 2H), 6.79 (td, *J* = 7.4, 1.2, 1H), 6.21 (dd, *J* = 8.4, 1.2, 1H), 5.20 (bs, 1H), 2.29 (s, 3H), 2.14 (s, 6H). ^{13}C NMR (151 MHz, $CDCl_3$) δ 143.6, 139.7, 136.3, 135.6, 135.4, 130.4, 129.6, 129.3, 129.1, 128.7, 127.5, 127.4, 117.6, 111.5, 21.1, 18.4. HRMS (ESI) *m/z* calcd for $[C_{21}H_{22}N]^+$: 288.1747 $[M+H]^+$; found: 288.1739.

5-Mesityl-6-methyl-5,6-dihydrodibenzo[*c*,*e*][1,2]azaborinine (5^{Me})

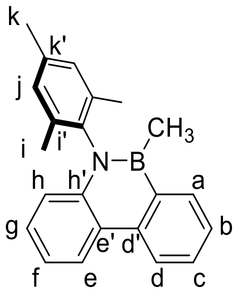

To a flame-dry 50 mL Schlenk tube, BBr_3 (1 M in hexanes, 1.0 mL, 1.0 mmol) was added dropwise to a stirring solution of dry **14** (144 mg, 0.5 mmol) in dry *o*-DCB (5 mL) at 0 °C. The green solution was stirred at 120 °C for 18 h under argon atmosphere. After allowing the blue suspension to reach room temperature, three *freeze-pump-thaw* cycles were performed. MeMgBr (3 M in diethyl ether, 1.0 mL, 3.0 mmol) was added dropwise at 0 °C and the brown solution was stirred at room temperature for 2 h. The mixture was quenched by dropwise addition of water (20 mL), acidified with sat. aq. NH_4Cl (10 mL) and extracted with CH_2Cl_2 (3 x 50 mL). The combined organic layers were dried over Na_2SO_4, and the solvents evaporated *in vacuo*. The resulting brown solid was purified by silica gel column chromatography (heptane) affording **5^{Me}** as a white solid (132 mg, 84% yield over two steps). The solid was further purified by liquid-liquid diffusion in layered $CHCl_3$/MeOH to provide the analytically pure product.

mp 151–152 °C. ^{1}H NMR (700 MHz, $CDCl_3$) δ 8.52 (dd, *J* = 8.4, 1.0, 1H, H_a), 8.51–8.49 (m, 1H, H_e), 8.26 (dd, *J* = 7.4, 1.5, 1H, H_d), 7.78 (ddd, *J* = 8.4, 7.1, 1.5, 1H, H_b), 7.56 (ddd, *J* = 7.4, 7.1, 1.0, 1H, H_c), 7.26–7.23 (m, 2H, H_f, H_g), 7.07 (s, 2H, H_j), 6.75–6.68 (m, 1H, H_h), 2.41 (s, 3H, H_k), 1.88 (s, 6H, H_i), 0.66 (s, 3H, B-CH_3). ^{13}C NMR (176 MHz, $CDCl_3$) δ 140.4 ($C_{h'}$), 140.0 (C-N), 138.1 ($C_{d'}$), 136.5 ($C_{k'}$), 135.09 ($C_{i'}$), 135.09 (C_d), 131.1 (C_b), 129.6 (C_j), 128.2 (C_g), 126.2 (C_c), 124.14 (C_e), 124.12 ($C_{e'}$), 122.2 (C_a), 121.3 (C_f), 116.7 (C_h), 21.2 (C_k), 17.8 (C_i) (two signals are missing due to ^{11}B-induced quadrupolar relaxation). ^{11}B NMR (193 MHz, $CDCl_3$) δ 40.2. IR (cm^{-1}): 2967, 2939, 2914, 1602, 1578, 1555, 1483, 1444, 1433, 1363, 1347, 1324, 1312, 1281, 1240, 1191, 1166, 1127, 1051, 1030, 1008, 950, 936, 877, 858, 785, 750, 735, 722, 661, 615, 606, 580, 570, 531, 519, 490, 431, 423, 410. HRMS (GC/Q-TOF) *m/z* calcd for $[C_{22}H_{22}BN]^+$: 311.1840 $[M]^+$; found: 311.1834. Crystal suitable for X-ray diffraction was obtained by vapor diffusion of MeOH to a heptane solution of **5^{Me}** (CCDC #2165184 – see Table S1).

5-Mesityl-6-phenyl-5,6-dihydrodibenzo[*c*,*e*][1,2]azaborinine (5^{Ph})

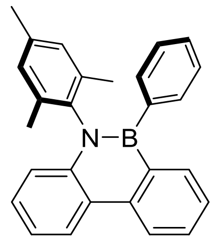

In the glove box, to a 25 mL Schlenk tube, BBr_3 (71 µL, 0.75 mmol) was added dropwise to a stirring solution of **14** (72 mg, 0.25 mmol) in *o*-DCB (2.5 mL). The yellow solution was stirred at 130 °C for 18 h in closed flask. After allowing the blue solution to reach room temperature, all volatiles were removed *in vacuo*. THF (1 mL) and PhMgBr (16% in THF, 2.45 mL, 2.25 mmol) were added and the yellow solution was stirred at room temperature for 18 h. The mixture was quenched by dropwise addition of MeOH (5 mL) at 0 °C. The solvents were evaporated *in vacuo*, the mixture was suspended in 1 M aq. HCl (15 mL) and extracted with CH_2Cl_2 (4 x 15 mL). The combined organic layers were dried over Na_2SO_4, and the solvents evaporated *in vacuo*. The resulting green solid was purified by silica gel column chromatography (heptane to heptane/CH_2Cl_2 9:1) affording **5^{Ph}** as a white solid (86 mg, 92% yield over two steps). Spectral properties are in agreement with those reported in the literature.[24]

^{1}H NMR (600 MHz, $CDCl_3$) δ 8.61–8.55 (m, 2H), 7.90 (dd, *J* = 7.7, 1.7, 1H), 7.78 (ddd, *J* = 8.5, 7.1, 1.5, 1H), 7.46 (t, *J* = 7.4, 1H), 7.35–7.27 (m, 4H), 7.25–7.17 (m, 3H), 6.86 (s, 2H), 6.83–6.77 (m, 1H), 2.28 (s, 3H), 1.89 (s, 6H). ^{13}C NMR (151 MHz, $CDCl_3$) δ 140.2, 139.4, 139.2, 137.6, 136.5, 135.1, 132.6, 131.3, 129.3, 128.4, 127.3, 126.9, 126.2, 124.6, 124.2, 122.01, 121.98, 117.3, 21.1, 18.2 (two signals are missing due to ^{11}B-induced quadrupolar relaxation). ^{11}B NMR (193 MHz, $CDCl_3$) δ 38.3. HRMS (LD-timsTOF) *m/z* calcd for $[C_{27}H_{24}BN]^+$: 373.2001 $[M]^+$; found: 373.1992. Crystal suitable for X-ray diffraction was obtained by thermal crystallization of a CH_3CN solution of **5^{Ph}** using a Technobis Crystal16 crystallization system (CCDC #2181763 – see Table S2).

5,6-Dimesityl-5,6-dihydrodibenzo[*c*,*e*][1,2]azaborinine (5^{Mes})

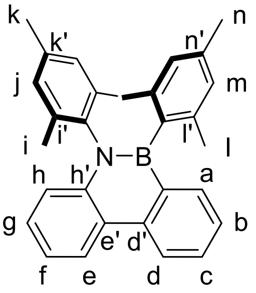

In the glove box, to a 10 mL Schlenk tube, BBr_3 (71 µL, 0.75 mmol) was added dropwise to a stirring solution of **14** (72 mg, 0.25 mmol) in *o*-DCB (2.5 mL). The yellow solution was stirred at 130 °C for 18 h in closed flask. After allowing the blue solution to reach room temperature, all volatiles were removed *in vacuo*. THF (2 mL) and MesMgBr (1.0 g, 4.5 mmol) were added and the yellow solution was stirred at 60 °C for 18 h. The mixture was quenched by dropwise addition of MeOH (5 mL) at 0 °C. The solvents were evaporated *in vacuo*, the mixture was suspended in 1 M aq. HCl (15 mL) and extracted with CH_2Cl_2 (4 x 15 mL). The combined organic layers were dried over Na_2SO_4, and the solvents evaporated *in vacuo*. The resulting yellow oil was purified by silica gel column chromatography (heptane to heptane/CH_2Cl_2 9:1) and liquid-liquid diffusion in layered CH_2Cl_2/MeOH affording **5^{Mes}** as a white crystalline solid (36 mg, 35% yield over two steps).

mp 214–216 °C. ^{1}H NMR (600 MHz, $CDCl_3$) δ 8.53 (dd, *J* = 7.7, 1.9, 1H, H_e), 8.51 (d, *J* = 8.2, 1H, H_a), 7.74 (ddd, *J* = 8.2, 7.2, 1.5, 1H, H_b), 7.59 (dd, *J* = 7.6, 1.5, 1H, H_d), 7.38 (dd, *J* = 7.6, 7.2, 1H, H_c), 7.32–

7.27 (m, 2H, H_f, H_g), 6.82 (s, 2H, H_j), 6.75 (dd, *J* = 8.0, 1.6, 1H, H_h), 6.70 (s, 2H, H_m), 2.27 (s, 3H, H_k), 2.26 (s, 3H, H_n), 1.93 (s, 6H, H_l), 1.84 (s, 6H, H_i). ^{13}C NMR (176 MHz, $CDCl_3$) δ 141.3 ($C_{h'}$), 140.2 ($C_{l'}$), 139.5 (C-N), 138.0 ($C_{d'}$), 136.7 ($C_{n'}$), 136.6 (C_d), 136.2 ($C_{k'}$), 135.6 ($C_{i'}$), 131.3 (C_b), 129.4 (C_j), 128.1 (C_g), 127.2 (C_m), 126.5 (C_c), 124.7 ($C_{e'}$), 124.3 (C_e), 122.0 (C_a), 121.8 (C_f), 118.2 (C_h), 23.5 (C_l), 21.3 (C_n), 21.1 (C_k), 18.8 (C_i) (two signals are missing due to ^{11}B-induced quadrupolar relaxation). ^{11}B NMR (193 MHz, $CDCl_3$) δ 41.0. IR (cm^{-1}): 3062, 2960, 2920, 2856, 1604, 1578, 1551, 1482, 1443, 1425, 1373, 1356, 1342, 1319, 1301, 1295, 1271, 1240, 1199, 1166, 1105, 1083, 1030, 993, 961, 922, 849, 802, 786, 752, 725, 659, 623, 594, 564, 524, 434. HRMS (LD-timsTOF) *m/z* calcd for $[C_{30}H_{30}BN]^+$: 415.2471 $[M]^+$; found: 415.2462. Crystal suitable for X-ray diffraction was obtained by vapor diffusion of MeOH to a $CHCl_3$ solution of **5^{Mes}** (CCDC #2165204 – see Table S3).

***N*,*N*'-(1,3-phenylene)bispivalamide (26)**

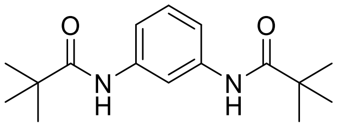

To a 250 mL round-bottom flask charged with *m*-phenylenediamine (4.00 g, 37.0 mmol), THF (100 mL) and Et_3N (11.3 mL, 81.4 mmol) were added. The solution was cooled to 0 °C and then pivaloyl chloride (10.0 mL, 81.4 mmol) was added dropwise. The white suspension was stirred at room temperature for 18 h. The mixture was poured in water (1 L) and filtered, affording **26** as a white solid (9.96 g, 97% yield). Spectral properties are in agreement with those reported in the literature.[25] ^{1}H NMR (400 MHz, $CDCl_3$) δ 7.91 (t, *J* = 1.9, 1H), 7.34 (bs, 2H), 7.32–7.28 (m, 2H), 7.28–7.22 (m, 1H), 1.30 (s, 18H). ^{13}C NMR (101 MHz, $CDCl_3$) δ 176.9, 138.8, 129.6, 115.5, 111.2, 39.8, 27.7. HRMS (ESI) *m/z* calcd for $[C_{16}H_{24}N_2O_2Na]^+$: 299.1730 $[M+Na]^+$; found: 299.1729.

***N*,*N*'-(4,6-diiodo-1,3-phenylene)bispivalamide (22)**

Procedure adapted from the literature.[25]

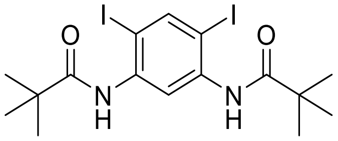

To a 500 mL round-bottom flask charged with **26** (5.00 g, 18.1 mmol), $CHCl_3$ (150 mL) was added. The solution was heated to 60 °C and iodine monochloride (6.47 g, 39.8 mmol) was added portion-wise. The brown solution was stirred at 60 °C for 24 h. After allowing it to reach room temperature, the mixture was washed with a 1:1 $NaHCO_3/Na_2S_2O_3$ aq. sol. (200 mL, prepared mixing 100 mL of sat. aq. $NaHCO_3$ and 100 mL of 0.5 M aq. sol. $Na_2S_2O_3$). The organic layer was separated and dried over Na_2SO_4, and the solvent evaporated *in vacuo*. The crude product was purified by silica gel column chromatography (CH_2Cl_2 to CH_2Cl_2/EtOAc 95:5) and boiled in heptane affording **22** as a white solid (8.90 g, 93% yield). Spectral properties are in agreement with those reported in the literature.[25]

^{1}H NMR (600 MHz, $CDCl_3$) δ 9.25 (s, 1H), 8.10 (s, 1H), 7.69 (bs, 2H), 1.34 (s, 18H). ^{13}C NMR (151 MHz, $CDCl_3$) δ 176.5, 146.0, 139.5, 114.9, 83.9, 40.4, 27.8. HRMS (ESI) *m/z* calcd for $[C_{16}H_{23}N_2O_2I_2]^+$: 528.9844 $[M+H]^+$; found: 528.9842.

N,N'-([1,1':3',1''-terphenyl]-4',6'-diyl)bispivalamide (27)

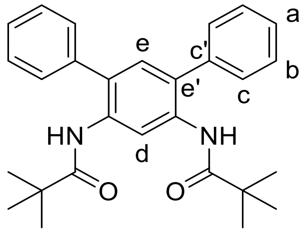

To a 100 mL round-bottom flask charged with diiodobispivalamide **22** (2.11 g, 4.00 mmol), phenylboronic acid (1.46 g, 12.0 mmol) and Na_2CO_3 (2.12 mg, 20.0 mmol), toluene (30 mL), EtOH (10 mL) and water (10 mL) were added. The mixture was degassed by bubbling argon for 10 min, then $[Pd(PPh_3)_4]$ (116 mg, 2.5 mol%) was added and 10 min of argon purging were performed again. The flask was equipped with a condenser and the brown suspension refluxed at 95 °C for 16 h under argon atmosphere. After allowing it to reach room temperature, the mixture was diluted with water (50 mL) and brine (250 mL) and extracted with CH_2Cl_2 (3 × 100 mL). The combined organic layers were dried over Na_2SO_4 and the solvents evaporated in vacuo. The resulting brown solid was purified by silica gel column chromatography (CH_2Cl_2 to CH_2Cl_2/EtOAc 7:3) affording **27** as an off-white solid (1.72 g, quantitative yield).
mp > 200 °C. ^{1}H NMR (700 MHz, DMSO-d_6) δ 8.84 (bs, 2H, N-H), 7.46 (s, 1H, H_d), 7.45–7.40 (m, 8H, H_b, H_c), 7.37–7.33 (m, 2H, H_a), 7.25 (s, 1H, H_e), 1.07 (s, 18H, $-CH_3$). ^{13}C NMR (176 MHz, DMSO-d_6) δ 176.4 (-C=O), 138.4 ($C_{c'}$), 135.4 (C-N), 134.6 ($C_{e'}$), 131.1 (C_e), 128.9 (C_b or C_c), 128.2 (C_b or C_c), 127.2 (C_a), 126.5 (C_d), 38.6 ($-C(CH_3)_3$), 27.1 ($-CH_3$). IR (cm^{-1}): 3434, 3290, 3057, 3027, 2957, 2929, 2906, 2869, 1653, 1584, 1571, 1521, 1490, 1443, 1399, 1366, 1322, 1291, 1226, 1200, 1171, 1074, 1029, 1012, 928, 891, 872, 824, 782, 759, 729, 699, 650, 619, 590, 557, 541, 522, 509, 490, 454, 405. HRMS (ESI) *m/z* calcd for $[C_{28}H_{33}N_2O_2]^+$: 429.2537 $[M+H]^+$; found: 429.2536.

[1,1':3',1''-Terphenyl]-4',6'-diamine (16)

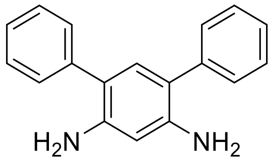

To a 100 mL round-bottom flask charged **27** (1.5 g, 3.5 mmol), dioxane (20 mL) and 6 M aq. HCl (10 mL) were added. The flask was equipped with a condenser and the suspension refluxed at 120 °C for 16 h. After allowing it to reach room temperature, the mixture was neutralized with conc. aq. NH_3 (pH checked with pH paper) and extracted with CH_2Cl_2 (3 x 100 mL). The combined organic layers were dried over Na_2SO_4 and the solvents evaporated *in vacuo.* The crude product was purified

by silica gel column chromatography (CH_2Cl_2 to CH_2Cl_2/EtOAc 9:1) affording **16** as a white solid (800 mg, 88% yield). Spectral properties are in agreement with those reported in the literature.[26]

^{1}H NMR (400 MHz, $CDCl_3$) δ 7.55–7.44 (m, 4H), 7.44–7.37 (m, 4H), 7.35–7.27 (m, 2H), 6.97 (s, 1H), 6.20 (s, 1H), 3.76 (bs, 4H). ^{13}C NMR (101 MHz, $CDCl_3$) δ 144.0, 139.6, 132.7, 129.3, 128.8, 126.7, 119.4, 102.2. HRMS (ESI) *m/z* calcd for $[C_{18}H_{17}N_2]^+$: 261.1386 [M+H]$^+$; found: 261.1383.

5,9-Dimesityl-6H,10H-6,10-diaza-5,9-dibora-dibenz[*a,j*]anthracene (17)

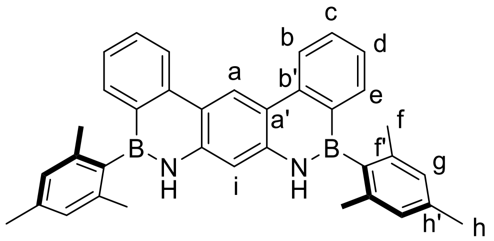

In the glove box, to a 10 mL Schlenk tube, BBr_3 (86 µL, 0.90 mmol) was added dropwise to a stirring solution of **16** (39 mg, 0.15 mmol) in DCE (1.5 mL). The solution was stirred at 60 °C for 16 h in closed flask. After allowing the blue suspension to reach room temperature, three *freeze-pump-thaw* cycles were performed. In the glove box, MesMgBr (603 mg, 2.70 mmol, obtained evaporating the solvent from a 1 M solution in Et_2O) and THF (2 mL) were added and the mixture was stirred at room temperature for 1 h. After adding sat. aq. NH_4Cl (4 mL) and water (30 mL), the mixture was extracted with CH_2Cl_2 (3 x 20 mL). The combined organic layers were dried over Na_2SO_4 and the solvent evaporated *in vacuo*. The resulting yellow oil was purified by silica gel column chromatography (heptane/CH_2Cl_2 8:2) and recrystallization from boiling CH_2Cl_2/MeOH affording **17** as a white solid (71 mg, 92% yield over two steps).

mp 186–188 °C. ^{1}H NMR (600 MHz, $CDCl_3$) *δ* 9.60 (s, 1H, H_a), 8.76–8.68 (m, 2H, H_b), 7.85–7.79 (m, 2H, H_c), 7.79–7.75 (m, 2H, H_e), 7.67 (bs, 2H, N-H), 7.45–7.39 (m, 2H, H_d), 7.01 (s, 1H, H_i), 6.96 (s, 4H, H_g), 2.39 (s, 6H, H_h), 2.18 (s, 12H, H_f). ^{13}C NMR (151 MHz, $CDCl_3$) *δ* 140.5 ($C_{f'}$), 139.4 (C_a or C_b), 139.0 (C_a or C_b), 137.9 ($C_{h'}$), 136.6 (C_e), 131.6 (C_c), 127.3 (C_g), 126.0 (C_d), 121.9 (C_b), 119.9 (C_a), 119.1 (C-N), 107.2 (C_i), 22.9 (C_f), 21.4 (C_h) (two signals are missing due to ^{11}B-induced quadrupolar relaxation). ^{11}B NMR (193 MHz, $CDCl_3$) *δ* 39.81. IR (cm^{-1}): 3524, 3373, 3290, 2909, 2332, 2116, 2064, 1802, 1625, 1605, 1565, 1556, 1523, 1474, 1448, 1435, 1423, 1375, 1338, 1312, 1294, 1245, 1232, 1194, 1161, 1080, 1012, 953, 899, 878, 847, 770, 752, 731, 657, 640, 618, 579, 510, 480, 468, 460, 442, 428, 415. HRMS (LD-timsTOF) *m/z* calcd for $[C_{36}H_{34}B_2N_2]^+$: 516.2915 [M]$^+$; found: 516.2916.

3',5'-Dibromo-2,4,6-trimethyl-1,1'-biphenyl (28)

Procedure adapted from the literature.[27]

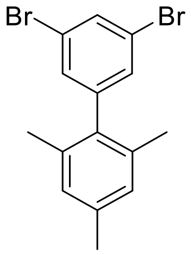

To a 500 mL three-neck round-bottom flask charged with 1,3,5-tribromobenzene (10.2 g, 32.5 mmol), 2,4,6-trimethylphenylboronic acid (4.44 g, 27.1 mmol), Na_2CO_3 (5.72 g, 54.0 mmol), toluene (162 mL), EtOH (54 mL) and water (27 mL) were added. The mixture was degassed by two *freeze-pump-thaw* cycles, then [Pd(PPh_3)$_4$] (625 mg, 2.0 mol%) was added under argon flow. The flask was equipped with a condenser and the mixture refluxed at 95 °C for 18 h under argon atmosphere. After allowing it to reach room temperature, the mixture was diluted with water (100 mL) and extracted with EtOAc (3 x 200 mL). The combined organic layers were dried over $MgSO_4$, and the solvents evaporated *in vacuo*. The resulting beige solid was purified by silica gel column chromatography (petroleum ether) and recrystallization from boiling CH_2Cl_2/MeOH affording **28** as a white solid (4.66 g, 49% yield). Spectral properties are in agreement with those reported in the literature.[28]
^{1}H NMR (600 MHz, $CDCl_3$) δ 7.67 (t, *J* = 1.8, 1H), 7.27 (d, *J* = 1.8, 2H), 6.92 (s, 2H), 2.32 (s, 3H), 2.01 (s, 6H). ^{13}C NMR (151 MHz, $CDCl_3$) δ 145.0, 137.7, 136.3, 135.7, 132.4, 131.3, 128.4, 123.1, 21.2, 20.8. IR (cm^{-1}): 3056, 2914, 2853, 1734, 1613, 1581, 1544, 1484, 1434, 1400, 1374, 1257, 1093, 1031, 989, 880, 853, 770, 755, 723, 695, 605, 579, 550, 522, 509, 482, 446, 412. HRMS (GC/Q-TOF) *m/z* calcd for $[C_{15}H_{14}Br_2]^+$: 353.9437 [M]$^+$; found: 353.9426.

5'-Mesityl-[1,1':3',1''-terphenyl]-2,2''-diamine (19)

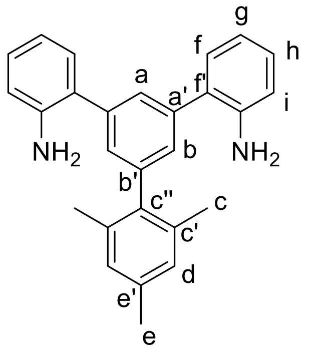

To a 250 mL round-bottom flask charged with **28** (3.54 g, 10 mmol), 2-aminophenylboronic acid pinacol ester **21** (6.57 g, 30 mmol) and Na_2CO_3 (5.30 g, 50 mmol), toluene (75 mL), EtOH (25 mL) and water (25 mL) were added. The mixture was degassed by bubbling argon for 10 min, then [Pd(PPh_3)$_4$]

(289 mg, 2.5 mol%) was added and 10 min of argon purging were performed again. The flask was equipped with a condenser and the mixture refluxed at 95 °C for 17 h under argon atmosphere. After allowing it to reach room temperature, the yellow mixture was diluted with water/brine (150 mL) and extracted with EtOAc (3 x 250 mL). The combined organic layers were dried over $MgSO_4$, filtered over Celite (washing with EtOAc) and the solvents evaporated *in vacuo*. The resulting red oil was purified by silica gel column chromatography (heptane/CH_2Cl_2 1:4 to 5:95). To remove residual unreacted 2-aminophenylboronic acid pinacol ester, the orange solid was dissolved in CH_2Cl_2 (150 mL) and washed with 1 M aq. HCl (3 x 150 mL) and sat. aq. $NaHCO_3$ (150 mL). The organic layer was dried over $MgSO_4$ and the solvent evaporated *in vacuo* affording **19** as a beige fluffy solid (2.73 g, 72% yield).

mp 80–81 °C. ^{1}H NMR (700 MHz, $CDCl_3$) δ 7.58 (t, *J* = 1.6, 1H, H_a), 7.28 (d, *J* = 1.6, 2H, H_b), 7.23 (dd, *J* = 7.8, 1.6, 2H, H_f), 7.17 (ddd, *J* = 8.0, 7.6, 1.6, 2H, H_h), 6.97 (s, 2H, H_d), 6.85 (ddd, *J* = 7.8, 7.6, 1.2, 2H, H_g), 6.79 (dd, *J* = 8.0, 1.2, 2H, H_i), 3.86 (bs, 4H, N-H), 2.35 (s, 3H, H_e), 2.13 (s, 6H, H_c). ^{13}C NMR (151 MHz, $CDCl_3$) δ 143.6 (C-N), 142.2 ($C_{b'}$), 140.3 ($C_{a'}$), 138.5 ($C_{c''}$), 136.8 ($C_{e'}$), 135.9 ($C_{c'}$), 130.7 (C_f), 128.8 (C_b), 128.7 (C_h), 128.3 (C_d), 127.9 (C_a), 127.3 ($C_{f'}$), 118.9 (C_g), 115.9 (C_i), 21.2 (C_c, C_e). IR (cm^{-1}): 3456, 3364, 3023, 2946, 2915, 2853, 1611, 1590, 1493, 1454, 1407, 1375, 1294, 1257, 1227, 1157, 1142, 1082, 1031, 934, 891, 850, 745, 725, 646, 621, 593, 549, 502, 474, 448, 432. HRMS (ESI) *m/z* calcd for $[C_{27}H_{27}N_2]^+$: 379.2169 [M+H]$^+$; found: 379.2164.

2-Mesityl-8H,9H-8,9-diaza-8a-borabenzo[*fg*]tetracene (18)

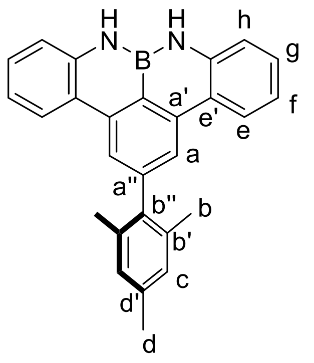

In the glove box, to a 10 mL Schlenk tube, BBr_3 (38 µL, 0.4 mmol) was added to a stirring solution of **19** (38 mg, 0.1 mmol) in DCE (1 mL). The yellow solution was stirred at 80 °C for 18 h in closed flask. After allowing the black solution to reach room temperature, the mixture was quenched by dropwise addition of water (20 mL) and extracted with CH_2Cl_2 (3 x 25 mL). The combined organic layers were dried over Na_2SO_4, and the solvents evaporated *in vacuo*. The resulting yellow powder was purified by silica gel column chromatography (heptane/CH_2Cl_2 7:3) affording **18** as a white powder (23 mg, 60% yield).

mp > 200 °C. ^{1}H NMR (600 MHz, $CDCl_3$) δ 8.19 (dd, *J* = 7.9, 1.5, 2H, H_e), 7.97 (s, 2H, H_a), 7.32 (ddd, *J* = 8.2, 7.1, 1.5, 2H, H_g), 7.09–7.03 (m, 6H, H_f, H_h, H_c), 6.28 (bs, 2H, N-H), 2.40 (s, 3H, H_d), 2.09 (s, 6H, H_b). ^{13}C NMR (151 MHz, $CDCl_3$) δ 144.1 ($C_{a''}$), 140.8 (C-N), 140.0 ($C_{b''}$), 139.3 ($C_{a'}$), 137.0 ($C_{d'}$), 136.3 ($C_{b'}$), 128.6 (C_g), 128.3 (C_c), 124.6 (C_e), 122.5 ($C_{e'}$), 120.23 (C_f or C_h), 120.17 (C_a), 118.5 (C_f or C_h), 21.2 (C_d), 21.0 (C_b) (one signal is missing due to ^{11}B-induced quadrupolar relaxation). ^{11}B NMR (193 MHz,

$CDCl_3$) δ 26.5. IR (cm^{-1}): 3385, 2951, 2912, 2848, 1603, 1588, 1559, 1504, 1454, 1437, 1411, 1376, 1347, 1321, 1256, 1247, 1198, 1163, 1123, 1043, 1005, 935, 873, 851, 824, 786, 748, 732, 663, 634, 617, 583, 546, 525, 488, 450. HRMS (LD-timsTOF) *m/z* calcd for $[C_{27}H_{23}BN_2]^+$: 386.1954 $[M]^+$; found: 386.1952.

N^2,$N^{2''}$,5'-Trimesityl-[1,1':3',1''-terphenyl]-2,2''-diamine (20)

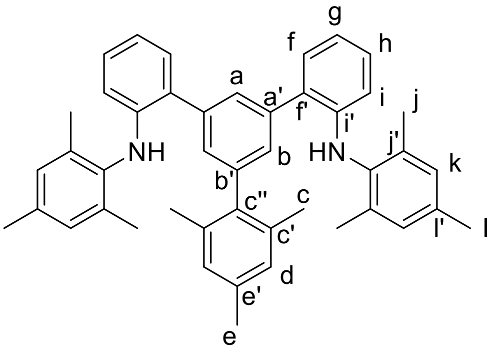

In the glove-box, to a 5 mL microwave vial charged with $[Pd_2(dba)_3]$ (1.2 mg, 0.52 mol%), *rac*-BINAP (2.3 mg, 1.5 mol%), *t*-BuONa (72 mg, 0.75 mmol) and **19** (95 mg, 0.25 mmol), 2-bromomesitylene (77 µL, 0.5 mmol) and toluene (2 mL) were added. The mixture was stirred at 110 °C for 20 h in closed flask. After allowing it to reach room temperature, the orange suspension was diluted with sat. aq. NH_4Cl (15 mL) and extracted with EtOAc (3 x 20 mL). The combined organic layers were washed with brine (20 mL), dried over Na_2SO_4, and the solvents evaporated *in vacuo*. The resulting orange solid was purified by silica gel column chromatography (heptane/CH_2Cl_2 9:1) affording **20** as a white fluffy solid (126 mg, 82% yield).

mp 189–190 °C. ^1H NMR (700 MHz, $CDCl_3$) δ 7.74 (t, *J* = 1.7, 1H, H_a), 7.37 (d, *J* = 1.7, 2H, H_b), 7.25 (dd, *J* = 7.6, 1.6, 2H, H_f), 7.08 (ddd, *J* = 8.4, 7.4, 1.6, 2H, H_h), 6.96 (s, 2H, H_d), 6.91 (s, 4H, H_k), 6.80 (ddd, *J* = 7.6, 7.4, 1.2, 2H, H_g), 6.23 (dd, *J* = 8.4, 1.2, 2H, H_i), 5.30 (bs, 2H, N-H), 2.34 (s, 3H, H_e), 2.29 (s, 6H, H_l), 2.11 (s, 6H, H_c), 2.10 (s, 12H, H_j). ^{13}C NMR (176 MHz, $CDCl_3$) δ 143.6 ($C_{i'}$), 142.5 ($C_{b'}$), 140.4 ($C_{a'}$), 138.5 ($C_{c''}$), 136.9 ($C_{e'}$), 136.1 ($C_{j'}$), 135.82 ($C_{c'}$ or C-N), 135.78 ($C_{c'}$ or C-N), 135.6 ($C_{l'}$), 130.4 (C_f), 129.5 (C_b), 129.3 (C_k), 128.8 (C_h), 128.7 (C_a), 128.4 (C_d), 127.3 ($C_{f'}$), 117.8 (C_g), 111.8 (C_i), 21.2 (C_e), 21.1 (C_c), 21.0 (C_l), 18.4 (C_j). IR (cm^{-1}): 3409, 2998, 2915, 2854, 1600, 1587, 1576, 1497, 1484, 1453, 1434, 1406, 1375, 1310, 1294, 1281, 1256, 1225, 1158, 1033, 1010, 890, 851, 746, 725, 654, 620, 531, 473. HRMS (ESI) *m/z* calcd for $[C_{45}H_{47}N_2]^+$: 615.3734 $[M+H]^+$; found: 615.3737.

2,8,9-Trimesityl-8H,9H-8,9-diaza-8a-borabenzo[*fg*]tetracene (6)

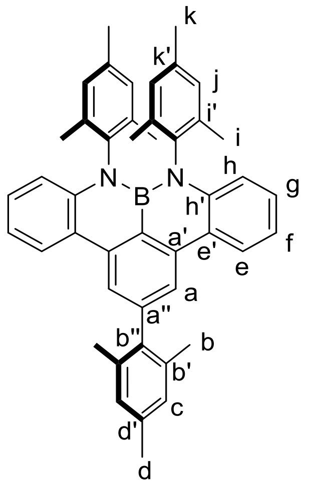

In the glove box, to a 10 mL Schlenk tube, BBr_3 (38 µL, 0.4 mmol) was added to a stirring solution of **20** (62 mg, 0.1 mmol) in *o*-DCB (1 mL). The yellow solution was stirred at 120 °C for 18 h in closed flask. After allowing the black solution to reach room temperature, the mixture was quenched by dropwise addition of water (20 mL) and extracted with CH_2Cl_2 (3 x 25 mL). The combined organic layers were dried over Na_2SO_4, and the solvents evaporated *in vacuo*. The resulting green solid was purified by silica gel column chromatography (heptane/CH_2Cl_2 95:5 to 9:1) affording **6** as a white solid (53 mg, 85% yield).

mp > 200 °C. ^{1}H NMR (600 MHz, $CDCl_3$) δ 8.32 (dd, *J* = 8.1, 1.6, 2H, H_e), 8.13 (s, 2H, H_a), 7.09 (ddd, *J* = 8.5, 6.9, 1.6, 2H, H_g), 7.07 (s, 2H, H_c), 7.05 (ddd, *J* = 8.1, 6.9, 1.4, 2H, H_f), 6.60 (s, 4H, H_j), 6.35 (dd, *J* = 8.5, 1.4, 2H, H_h), 2.42 (s, 3H, H_d), 2.28 (s, 6H, H_k), 2.18 (s, 6H, H_b), 1.84 (s, 12H, H_i). ^{13}C NMR (151 MHz, $CDCl_3$) δ 143.7 ($C_{b''}$), 142.6 ($C_{h'}$), 140.0 ($C_{a''}$), 139.2 ($C_{a'}$), 138.8 (C-N), 137.0 ($C_{d'}$), 136.4 ($C_{b'}$), 136.3 ($C_{i'}$), 135.7 ($C_{k'}$), 128.9 (C_j), 128.6 (C_g), 128.3 (C_c), 124.2 (C_e), 123.3 ($C_{e'}$), 120.40 (C_a), 120.36 (C_f), 117.0 (C_h), 21.3 (C_d), 21.2 (C_b), 21.1 (C_k), 19.0 (C_i) (one signal is missing due to ^{11}B-induced quadrupolar relaxation). ^{11}B NMR (193 MHz, $CDCl_3$) δ 27.1. IR (cm^{-1}): 2998, 2950, 2915, 2853, 1605, 1578, 1541, 1495, 1476, 1443, 1413, 1359, 1338, 1313, 1295, 1277, 1255, 1217, 1172, 1088, 1060, 1028, 1007, 881, 872, 849, 784, 750, 733, 663, 628, 592, 561, 491, 453. HRMS (MALDI-timsTOF, matrix: DCTB) *m/z* calcd for $[C_{45}H_{43}BN_2]^+$: 622.3521 $[M]^+$; found: 622.3511. Crystal suitable for X-ray diffraction was obtained by vapor diffusion of MeOH to a $CHCl_3$ solution of **6** (CCDC #2165185 – see Table S4).

***N*,*N*'-(2,2''-diamino-[1,1':3',1''-terphenyl]-4',6'-diyl)bispivalamide (7_1)**

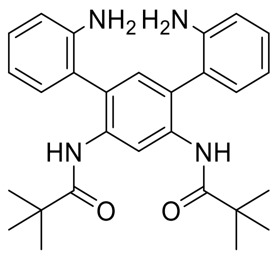

To a 100 mL round-bottom flask charged with diiodobispivalamide **22** (2.11 g, 4.00 mmol), 2-aminophenylboronic acid pinacol ester **21** (2.63 g, 12.0 mmol) and Na_2CO_3 (2.12 g, 20.0 mmol), toluene (30 mL), EtOH (10 mL) and water (10 mL) were added. The mixture was degassed by bubbling argon for 10 min, then $[Pd(PPh_3)_4]$ (116 mg, 2.5 mol%) was added and 10 min of argon purging were performed again. The flask was equipped with a condenser and the mixture refluxed at 95 °C for 16 h under argon atmosphere. After allowing it to reach room temperature, the mixture was diluted with water (50 mL) and brine (250 mL) and extracted with CH_2Cl_2 (2 × 100 mL). The combined organic layers were dried over Na_2SO_4 and the solvent evaporated *in vacuo*. The resulting brown solid was purified by silica gel column chromatography (CH_2Cl_2 to CH_2Cl_2/EtOAc 1:1) and recrystallization from boiling CH_2Cl_2/petroleum ether affording **7_1** as off-white crystals (1.74 g, 95% yield, mixture of atropoisomers).

mp > 200 °C. ^{1}H NMR (700 MHz, DMSO-*d_6*) δ 9.02–8.15 (m, 3H), 7.18–6.66 (m, 9H), 5.05–4.87 (m, 4H), 1.07–0.99 (m, 18H). ^{13}C NMR (176 MHz, DMSO-*d_6*) δ 175.7, 175.5, 145.6, 144.9, 135.2, 134.9, 132.2, 131.8, 131.1, 130.4, 128.8, 128.7, 128.6, 127.9, 124.0, 122.6, 119.0, 117.8, 117.4, 117.1, 115.9, 115.2, 27.0, 26.9. IR (cm^{-1}): 3471, 3399, 3348, 3058, 3031, 2959, 2900, 2866, 1679, 1614, 1584, 1521, 1486, 1461, 1446, 1414, 1366, 1330, 1292, 1266, 1201, 1159, 1137, 1038, 1005, 932, 900, 884, 859, 844, 750, 693, 630, 617, 551, 520, 500, 483, 460, 441. HRMS (ESI) *m/z* calcd for $[C_{28}H_{34}N_4O_2Na]^+$: 481.2574 $[M+Na]^+$; found: 481.2573.

[1,1':3',1''-Terphenyl]-2,2'',4',6'-tetraamine (8_1)

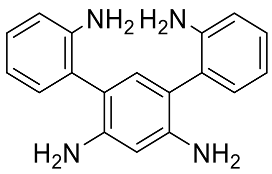

To a 100 mL round-bottom flask charged with **7_1** (1.50 g, 3.27 mmol), dioxane (30 mL) and 6 M aq. HCl (10 mL) were added. The flask was equipped with a condenser and the suspension refluxed at 120 °C for 16 h. After allowing it to reach room temperature, the mixture was neutralized with conc. aq. NH_3 (pH checked with pH paper) and extracted with CH_2Cl_2 (2 x 100 mL). The combined organic layers were dried over Na_2SO_4 and the solvent evaporated *in vacuo*. The crude product was purified by silica gel column chromatography (CH_2Cl_2 to EtOAc) affording **8_1** as an off-white solid (935 mg, quantitative yield, mixture of atropoisomers).

mp 169–171 °C. ^{1}H NMR (600 MHz, DMSO-*d_6*) δ 7.06–6.90 (m, 4H), 6.78–6.70 (m, 2H), 6.63–6.51 (m, 3H), 6.24–6.18 (m, 1H), 4.63 (s, 4H), 4.44 (s, 2H), 4.37 (s, 2H). ^{13}C NMR (151 MHz, DMSO-*d_6*) δ 145.8, 145.5, 145.2, 145.1, 132.9, 132.6, 131.0, 130.9, 127.5, 124.5, 124.3, 116.8, 116.6, 114.9, 114.8, 114.5, 114.2, 101.3, 101.3. IR (cm^{-1}): 3469, 3411, 3377, 3331, 3199, 3057, 3013, 2361, 2229, 2176, 2106, 1910, 1885, 1817, 1616, 1597, 1572, 1510, 1483, 1452, 1418, 1328, 1315, 1297, 1266, 1247, 1230, 1193, 1155, 1138, 1056, 1003, 971, 944, 911, 854, 826, 751, 731, 666, 645, 616, 584, 547, 526, 491, 474, 458, 442, 434, 416. HRMS (ESI) *m/z* calcd for $[C_{18}H_{19}N_4]^+$: 291.1604 $[M+H]^+$; found: 291.1602.

N^2,$N^{2''}$,$N^{4'}$,$N^{6'}$-tetramesityl-[1,1':3',1''-terphenyl]-2,2'',4',6'-tetraamine (9_1)

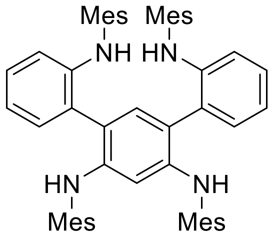

In the glove box, to a 20 mL microwave vial charged with **8_1** (203 mg, 0.70 mmol), toluene (8 mL) was added. *rac*-BINAP (13 mg, 3.0 mol%), *t*-BuONa (404 mg, 4.20 mmol), 2-bromomesitylene (1.72 mL, 11.2 mmol) and [$Pd_2(dba)_3$] (6 mg, 1 mol%) were successively added. The mixture was stirred at 100 °C for 72 h in closed flask. After allowing it to reach room temperature, the mixture was diluted with water (30 mL) and extracted with CH_2Cl_2 (3 x 30 mL). The combined organic layers were dried over Na_2SO_4, and the solvent evaporated *in vacuo*. The resulting brown oil was purified by silica gel column chromatography (heptane to heptane/CH_2Cl_2 7:3) affording **9_1** as a white solid (460 mg, 86% yield, mixture of atropoisomers).

mp > 200 °C. ^{1}H NMR (600 MHz, $CDCl_3$) δ 7.35–7.29 (m, 1H), 7.25–7.22 (m, 1H), 7.15–7.04 (m, 3H), 6.95–6.68 (m, 10H), 6.24–6.18 (m, 2H), 5.48–5.37 (m, 2H), 5.14–4.99 (m, 3H), 2.37–1.94 (m, 36H). ^{13}C NMR (151 MHz, $CDCl_3$) δ 145.7, 145.6, 145.4, 145.3, 136.6, 136.4, 135.8, 135.7, 135.6, 135.6, 135.5, 135.4, 135.0, 134.9, 132.8, 132.2, 131.6, 131.3, 129.1, 129.0, 128.6, 128.6, 128.6, 124.0, 123.9, 117.5, 117.3, 113.5, 113.3, 111.2, 111.1, 95.4, 20.9, 20.9, 20.8, 18.3, 18.2, 18.1, 18.1. IR (cm^{-1}): 3402, 3359, 3012, 2963, 2942, 2916, 2859, 1618, 1597, 1574, 1567, 1487, 1452, 1403, 1375, 1312, 1297, 1281, 1251, 1226, 1178, 1158, 1107, 1035, 1004, 934, 903, 851, 824, 745, 728, 676, 636, 561, 539, 524, 509, 469, 431, 421, 410. HRMS (ESI) *m/z* calcd for $[C_{54}H_{59}N_4]^+$: 763.4734 $[M+H]^+$; found: 763.4716.

Dimethyl-B_3N_4-tetracenoanthracene (1^{Me})

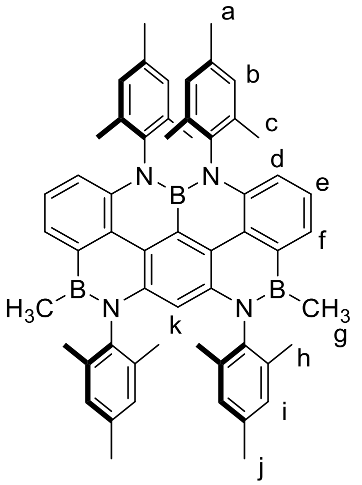

In the glove box, to a 10 mL Schlenk tube, BBr_3 (80 µL, 0.84 mmol) was added dropwise to a stirring solution of **9_1** (53 mg, 0.07 mmol) in *o*-DCB (1.5 mL). The orange solution was stirred at 170 °C for 48 h in closed flask. After allowing the brown solution to reach room temperature, three *freeze-pump-thaw* cycles were performed. In the glove box, MeMgBr (3 M in Et_2O, 0.84 mL, 2.5 mmol) was added and the mixture was stirred at room temperature for 16 h. After adding sat. aq. NH_4Cl (4 mL) and water (30 mL), the mixture was extracted with CH_2Cl_2 (3 x 20 mL). The combined organic layers were dried over Na_2SO_4 and the solvent evaporated *in vacuo*. The resulting brown solid was purified by silica gel column chromatography (heptane to heptane/CH_2Cl_2 9:1, column covered with aluminium foil) affording **1^{Me}** as a pale yellow solid (47 mg, 82% yield over two steps, 93% pure by NMR).

mp > 260 °C. ^{1}H NMR (600 MHz, $CDCl_3$) δ 7.88 (dd, *J* = 7.2, 1.1, 2H, H_f), 7.31 (dd, *J* = 8.2, 7.2, 2H, H_e), 6.86 (s, 4H, H_i), 6.66 (s, 4H, H_b), 6.60 (dd, *J* = 8.2, 1.1, 2H, H_d), 6.35 (s, 1H, H_k), 2.39 (s, 6H, H_j), 2.32 (s, 6H, H_a), 1.92 (s, 12H, H_c), 1.78 (s, 12H, H_h), 0.70 (s, 6H, H_g). ^{13}C NMR (151 MHz, $CDCl_3$) δ 141.4, 139.8, 139.3, 138.0, 136.4, 136.1, 135.6, 134.7, 129.0 (C_b, C_i), 126.7, 126.4 (C_f), 125.9 (C_e), 121.00, 117.1 (C_d), 105.1 (C_k), 21.3 (C_j), 21.2 (C_a), 19.1 (C_c), 17.7 (C_h) (three signals are missing due to ^{11}B-induced quadrupolar relaxation). ^{11}B NMR (193 MHz, $CDCl_3$) δ 41.1, 27.0, 22.3 (impurity). IR (cm^{-1}): 2945, 2915, 2853, 2363, 2326, 1601, 1579, 1551, 1520, 1479, 1455, 1434, 1342, 1325, 1304, 1292, 1277, 1264, 1238, 1214, 1179, 1154, 1107, 1031, 1011, 978, 961, 908, 871, 848, 814, 782, 758, 730, 693, 681, 617, 572, 538, 524, 503, 449. HRMS (LD-timsTOF) *m/z* calcd for $[C_{56}H_{57}B_3N_4]^+$: 818.4882 $[M]^+$; found: 818.4871. Crystal suitable for X-ray diffraction was obtained by vapor diffusion of MeOH to a toluene solution of **1^{Me}** (CCDC #2165187 – see Table S5).

Diphenyl-B_3N_4-tetracenoanthracene (1^{Ph})

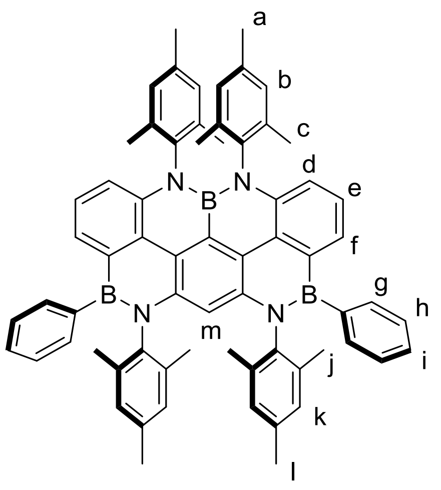

In the glove box, to a 25 mL Schlenk tube, BBr_3 (200 µL, 2.10 mmol) was added dropwise to a stirring solution of **9_1** (107 mg, 0.14 mmol) in *o*-DCB (2 mL). The orange solution was stirred at 170 °C for 24 h in closed flask. After allowing the mixture to reach room temperature, all volatiles were removed *in vacuo*. In the glove box, THF (1 mL) and PhMgBr (16% in THF, 6.3 mL, 6.3 mmol) were added and the brown solution was stirred at room temperature for 16 h. The solution was quenched with water (30 mL), diluted with 0.1 M aq. HCl (30 mL) and extracted with CH_2Cl_2 (3 x 30 mL). The combined

organic layers were dried over Na_2SO_4, and the solvents evaporated *in vacuo*. The resulting brown solid was purified by silica gel column chromatography (heptane to heptane/CH_2Cl_2 75:25) affording **1^{Ph}** as a bright yellow solid (100 mg, 76% yield over two steps).

mp > 260 °C. ^{1}H NMR (600 MHz, $CDCl_3$) δ 7.54 (d, *J* = 7.0, 2H, H_f), 7.37–7.31 (m, 4H, H_g), 7.23 (t, *J* = 7.9, 2H, H_e), 7.21–7.15 (m, 6H, H_h, H_i), 6.67 (s, 4H, H_b), 6.64 (s, 4H, H_k), 6.62 (d, *J* = 8.2, 2H, H_d), 6.55 (s, 1H, H_m), 2.33 (s, 6H, H_a), 2.24 (s, 6H, H_l), 1.96 (s, 12H, H_c), 1.82 (s, 12H, H_j). ^{13}C NMR (151 MHz, $CDCl_3$) δ 141.3, 139.3, 139.2, 137.9, 136.4, 136.0, 135.7, 134.6, 132.6 (C_g), 129.14 (C_f), 129.08 (C_b), 128.8 (C_k), 127.7, 127.2 (C_i), 126.8 (C_h), 126.1 (C_e), 122.0, 117.3 (C_d), 106.2 (C_m), 21.3 (C_l), 21.2 (C_a), 19.2 (C_c), 18.0 (C_j) (three signals are missing due to ^{11}B-induced quadrupolar relaxation). ^{11}B NMR (193 MHz, $CDCl_3$) δ 39.0, 26.6. IR (cm^{-1}): 3002, 2950, 2916, 2853, 1600, 1480, 1455, 1429, 1369, 1344, 1303, 1283, 1265, 1219, 1182, 1157, 1029, 935, 905, 879, 849, 820, 788, 750, 733, 701, 602, 530, 505, 486. HRMS (LD-timsTOF) *m/z* calcd for $[C_{66}H_{61}B_3N_4]^+$: 942.5198 $[M]^+$; found: 942.5189. Crystal suitable for X-ray diffraction was obtained by vapor diffusion of MeOH to a toluene solution of **1^{Ph}** (CCDC #2165201 – see Table S6).

***N*,*N*'-(2'-amino-5-iodo-[1,1'-biphenyl]-2,4-diyl)bispivalamide (24)**

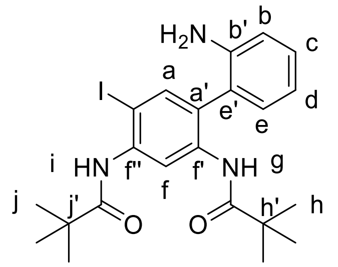

To a 250 mL round-bottom flask charged with diiodobispivalamide **22** (2.11 g, 4.00 mmol), 2-aminophenylboronic acid pinacol ester **21** (0.657 g, 3.00 mmol) and Na_2CO_3 (1.59 g, 15.0 mmol), toluene (60 mL), EtOH (20 mL) and water (20 mL) were added. The mixture was degassed by bubbling argon for 10 min, then $[Pd(PPh_3)_4]$ (87 mg, 1.9 mol%) was added and 10 min of argon purging were performed again. The flask was equipped with a condenser and the yellow suspension refluxed at 95 °C for 16 h under argon atmosphere. After allowing it to reach room temperature, the mixture was diluted with water (300 mL) and extracted with CH_2Cl_2 (2 x 100 mL). The combined organic layers were dried over Na_2SO_4 and the solvents evaporated *in vacuo*. The resulting orange solid was purified by silica gel column chromatography (CH_2Cl_2 to CH_2Cl_2/EtOAc 85:15) and recrystallization from boiling heptane/CH_2Cl_2 affording **24** as an off-white solid (1.07 g, 72% yield).

mp > 200 °C. ^{1}H NMR (700 MHz, $CDCl_3$) δ 9.02 (s, 1H, H_f), 7.78 (bs, 1H, N-H_g), 7.70 (bs, 1H, N-H_i), 7.67 (s, 1H, H_a), 7.22 (ddd, *J* = 8.0, 7.6, 1.6, 1H, H_c), 7.04 (dd, *J* = 7.5, 1.6, 1H, H_e), 6.85 (ddd, *J* = 7.6, 7.5, 1.2, 1H, H_d), 6.80 (dd, *J* = 8.0, 1.2, 1H, H_b), 3.64 (bs, 2H, $-NH_2$), 1.37 (s, 9H, H_j), 1.06 (s, 9H, H_h). ^{13}C NMR (176 MHz, $CDCl_3$) δ 176.64 (C=O), 176.62 (C=O), 143.6 ($C_{b'}$), 139.6 (C_a), 138.6 ($C_{f'}$ or $C_{f''}$), 137.1 ($C_{f'}$ or $C_{f''}$), 131.2 (C_e), 130.0 (C_c), 128.0 ($C_{a'}$), 122.0 ($C_{e'}$), 119.5 (C_d), 116.5 (C_f), 115.7 (C_b), 84.7 (C-I), 40.2 ($C_{j'}$), 39.8 ($C_{h'}$), 27.8 (C_j), 27.3 (C_h). IR (cm^{-1}): 3395, 3353, 3286, 3215, 2956, 2931, 2903, 2868, 1690, 1656, 1605, 1579, 1560, 1482, 1449, 1390, 1365, 1322, 1303, 1284, 1257, 1245, 1205, 1159,

1142, 1046, 1026, 1016, 926, 888, 848, 827, 806, 770, 751, 722, 677, 655, 550, 524, 490, 447. HRMS (ESI) *m/z* calcd for $[C_{22}H_{29}N_3O_2I]^+$: 494.1299 $[M+H]^+$; found: 494.1300.

***N*,*N*',*N*'',*N*'''-(2,2'''-diamino-[1,1':3',1'':3'',1'''-quaterphenyl]-4',4'',6',6''-tetrayl)tetrakispivalamide (7_2)**

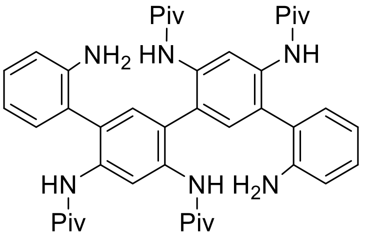

To a 50 mL round-bottom flask charged with iodobiaryl **24** (666 mg, 1.35 mmol), B_2Pin_2 (360 mg, 1.42 mmol), KOAc (397 mg, 4.05 mmol) and $[Pd(dppf)Cl_2]$ (99 mg, 10 mol%), dry DMF (13 mL) was added. The mixture was degassed by bubbling argon for 5 min under sonication. The brown suspension was stirred at 95 °C for 3 h, with the flask sealed with a septum. After allowing it to reach room temperature, the mixture was filtered through Celite (washing with CH_2Cl_2), and the organic solution was concentrated *in vacuo*. The crude product was dissolved in CH_2Cl_2 (50 mL), washed with water (50 mL) and the aqueous layer was extracted with CH_2Cl_2 (2 x 50 mL). The combined organic layers were dried over Na_2SO_4 and the solvent evaporated *in vacuo.*
To the resulting brown solid, iodobiaryl **24** (666 mg, 1.35 mmol), Na_2CO_3 (572 mg, 5.40 mmol), $[Pd(PPh_3)_4]$ (39 mg, 2.5 mol%), toluene (15 mL), EtOH (5 mL) and water (5 mL) were added. The mixture was degassed by bubbling argon for 10 min. The flask was equipped with a condenser and the mixture refluxed at 95 °C for 7 h under argon atmosphere. After allowing it to reach room temperature, the suspension was diluted with water (200 mL) and extracted with CH_2Cl_2 (3 × 100 mL). The combined organic layers were dried over Na_2SO_4 and the solvents evaporated *in vacuo*. The resulting black solid was purified by silica gel column chromatography (CH_2Cl_2 to CH_2Cl_2/EtOAc 3:2) affording $\mathbf{7_2}$ as an off-white solid (710 mg, 72% yield over two steps, mixture of atropoisomers). mp > 200 °C. ^{1}H NMR (600 MHz, $CDCl_3$) δ 8.91–8.67 (m, 2H), 7.92–7.70 (m, 2H), 7.56–7.36 (m, 2H), 7.25–6.76 (m, 10H), 3.72–3.54 (m, 4H), 1.22–0.94 (m, 36H). ^{13}C NMR (151 MHz, $CDCl_3$) δ 177.5, 177.4, 177.0, 176.9, 176.7, 176.84, 176.80, 176.7, 143.9, 143.8, 143.5, 143.3, 136.7, 136.6, 136.5, 136.4, 136.3, 136.2, 136.1, 132.1, 131.9, 131.64, 131.58, 131.4, 131.1, 130.8, 129.9, 129.83, 129.81, 129.78, 127.6, 127.4, 127.3, 127.08, 127.06, 126.8, 126.7, 122.9, 122.8, 122.7, 122.3, 119.7, 119.6, 119.4, 119.3, 118.4, 118.1, 117.91, 117.86, 115.8, 115.7, 39.81, 39.78, 39.76, 39.75, 39.6, 39.5, 27.52, 27.45, 27.4, 27.3. IR (cm^{-1}): 3475, 3429, 3347, 2961, 2869, 1681, 1660, 1640, 1620, 1566, 1513, 1492, 1482, 1446, 1392, 1366, 1312, 1213, 1196, 1163, 1144, 1028, 1008, 940, 924, 903, 870, 829, 749, 603, 571, 528, 482, 449, 423. HRMS (ESI) *m/z* calcd for $[C_{44}H_{57}N_6O_4]^+$: 733.4436 $[M+H]^+$; found: 733.4437.

[1,1':3',1'':3'',1'''-quaterphenyl]-2,2''',4',4'',6',6''-hexaamine (8_2)

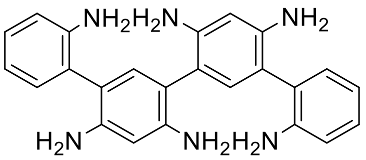

To a 50 mL round-bottom flask charged with $\mathbf{7_2}$ (500 mg, 0.68 mmol), dioxane (10 mL) and 6 M aq. HCl (10 mL) were added. The mixture was heated at 100 °C for 16 h, covering the flask with aluminium foil. After allowing it to reach room temperature, the mixture was neutralized with sat. aq. $NaHCO_3$ (200 mL) and extracted with CH_2Cl_2 (3 x 100 mL). The combined organic layers were dried over Na_2SO_4 and the solvent evaporated *in vacuo*. The crude residue was purified by silica gel column chromatography (CH_2Cl_2 to CH_2Cl_2/MeOH 9:1) affording $\mathbf{8_2}$ as an off-white solid (270 mg, quantitative yield, mixture of atropoisomers).
mp 188–190 °C. ^{1}H NMR (700 MHz, DMSO-d_6) δ 7.04–6.88 (m, 4H), 6.78–6.68 (m, 2H), 6.63–6.50 (m, 4H), 6.23–6.14 (m, 2H), 4.71–4.26 (m, 12H). ^{13}C NMR (176 MHz, DMSO-d_6) δ 145.77, 145.75, 145.7, 145.6, 145.5, 145.4, 145.3, 144.61, 144.56, 133.2, 133.0, 132.8, 131.0, 130.9, 127.4, 124.90, 124.7, 124.58, 124.55, 116.8, 116.7, 116.6, 115.00, 114.9, 114.8, 114.6, 114.4, 114.3, 114.23, 114.18, 113.9, 101.24, 101.20, 101.1. IR (cm^{-1}): 3548, 3431, 3418, 3353, 3342, 3207, 3016, 1620, 1609, 1560, 1505, 1485, 1450, 1422, 1339, 1294, 1267, 1248, 1203, 1180, 1156, 1139, 1039, 1004, 902, 837, 750, 696, 668, 645, 614, 601, 584, 539, 522, 471, 453. HRMS (LD-timsTOF) *m/z* calcd for $[C_{24}H_{24}N_6]^+$: 396.2057 $[M^+]$; found: 396.2051.

***N^2,$N^{2'''}$,$N^{4'}$,$N^{4''}$,$N^{6'}$,$N^{6''}$*-hexamesityl-[1,1':3',1'':3'',1'''-quaterphenyl]-2,2''',4',4'',6',6''-hexaamine (9_2)**

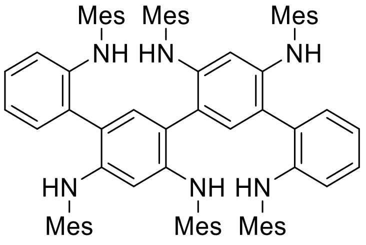

In the glove box, to a 20 mL microwave vial charged with $\mathbf{8_2}$ (198 mg, 0.5 mmol), $[Pd_2(dba)_3]$ (4.6 mg, 1.0 mol%), *rac*-BINAP (9.3 mg, 3.0 mol%), *t*-BuONa (432 mg, 4.5 mmol), 2-bromomesitylene (1.84 mL, 12.0 mmol) and toluene (6 mL) were added. The mixture was stirred at 100 °C for 72 h in closed flask. After allowing it to reach room temperature, the mixture was diluted with water (100 mL) and extracted with CH_2Cl_2 (3 x 50 mL). The combined organic layers were dried over Na_2SO_4, and the solvent evaporated *in vacuo*. The resulting brown oil was purified by silica gel column chromatography (heptane to heptane/CH_2Cl_2 75:25) affording $\mathbf{9_2}$ as a white solid (400 mg, 72% yield, mixture of atropoisomers).
mp > 200 °C. ^{1}H NMR (600 MHz, $CDCl_3$) δ 7.34–7.27 (m, 1H), 7.26–6.64 (m, 19H), 6.28–6.16 (m, 2H), 5.53–4.95 (m, 8H), 2.37–1.87 (m, 54H). ^{13}C NMR (151 MHz, $CDCl_3$) δ 146.4, 146.33, 146.31, 146.2,

145.5, 145.44, 145.38, 145.3, 136.7, 136.5, 136.2, 136.14, 136.09, 136.06, 136.04, 136.01, 135.97, 135.9, 135.5, 135.4, 135.02, 135.00, 134.98, 134.94, 134.91, 133.65, 133.59, 133.4, 133.00, 131.8, 131.6, 131.5, 129.24, 129.20, 129.15, 128.73, 128.70, 128.66, 128.61, 128.58, 128.56, 124.6, 124.5, 124.4, 124.3, 117.6, 117.5, 117.4, 114.2, 114.1, 113.75, 113.71, 113.68, 113.5, 113.4, 111.3, 111.21, 111.18, 95.5, 95.42, 95.35, 21.08, 21.05, 21.04, 20.92, 20.89, 18.5, 18.41, 18.36, 18.33, 18.25, 18.22, 18.20. IR (cm^{-1}): 3402, 3359, 3013, 2964, 2942, 2915, 2852, 1615, 1597, 1563, 1505, 1484, 1452, 1434, 1395, 1375, 1335, 1311, 1279, 1254, 1225, 1184, 1155, 1034, 1010, 903, 850, 834, 749, 662, 628, 562, 534, 516, 470, 423. HRMS (ESI) *m/z* calcd for $[C_{78}H_{85}N_6]^+$: 1105.6830 $[M+H]^+$; found: 1105.6820.

Diphenyl-B_4N_6-pentacenopentacene (2)

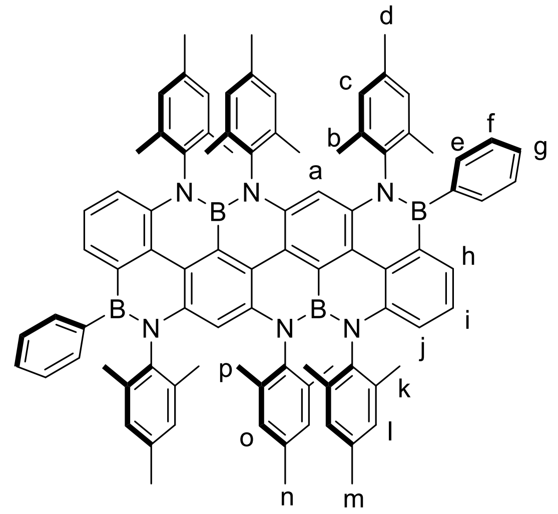

In the glove box, to a 10 mL Schlenk tube with PTFE screwcap, BBr_3 (142 µL, 1.50 mmol) was added dropwise to a stirring solution of **9₂** (55 mg, 0.05 mmol) in TCB (1 mL). The mixture was stirred at 230 °C (sand bath) for 16 h in closed flask. After allowing the mixture to reach room temperature, all volatiles were removed *in vacuo*. In the glove box, THF (1 mL) and PhMgBr (16% in THF, 4.5 mL, 4.5 mmol) were added and the yellow suspension was stirred at 50 °C for 16 h. The solution was quenched with water (40 mL), diluted with 0.1 M aq. HCl (20 mL) and extracted with CH_2Cl_2 (3 x 30 mL). The combined organic layers were dried over Na_2SO_4, and the solvents evaporated *in vacuo*. The resulting yellow solid was purified by silica gel column chromatography (heptane to heptane/CH_2Cl_2 8:2) affording **2** as a bright yellow solid (33 mg, 51% yield over two steps).
mp > 260 °C. ^1H NMR (600 MHz, CD_2Cl_2) δ 7.32 (dd, *J* = 7.2, 1.0, 2H, H_h), 7.28–7.24 (m, 4H, H_e), 7.20–7.12 (m, 6H, H_f, H_g), 7.07 (dd, *J* = 8.1, 7.2, 2H, H_i), 6.64–6.57 (m, 8H, H_c, H_l), 6.45 (dd, *J* = 8.2, 1.0, 2H, H_j), 6.41 (s, 4H, H_o), 6.05 (s, 2H, H_a), 2.31 (s, 6H, H_n), 2.30–2.28 (m, 12H, H_d, H_m), 1.92 (s, 12H, H_k), 1.78 (s, 12H, H_b), 1.77 (s, 12H, H_p). ^{13}C NMR (151 MHz, CD_2Cl_2) δ 141.6, 141.2, 139.8, 139.4, 139.3, 138.1, 136.6, 136.4, 136.2, 136.0, 135.4, 135.00, 132.9 (C_e), 129.7 (C_h), 129.6 (C_l), 129.3 (C_c), 129.1 (C_o), 128.4, 127.6 (C_g), 127.4 (C_f), 126.2 (C_i), 122.5, 121.2, 117.4 (C_j), 106.5 (C_a), 21.70 (C_d or C_m or C_n), 21.66 (C_d or C_m or C_n), 21.57 (C_d or C_m or C_n), 19.3 (C_k), 19.2 (C_p), 18.3 (C_b) (three signals are missing due to ^{11}B-induced quadrupolar relaxation). ^{11}B NMR (193 MHz, $CDCl_3$) δ 39.1, 27.6. IR (cm⁻

[1]): 3003, 2950, 2915, 2853, 1599, 1547, 1480, 1433, 1396, 1372, 1348, 1333, 1302, 1285, 1264, 1223, 1182, 1157, 1030, 871, 846, 813, 779, 754, 735, 701, 605, 449. HRMS (LD-timsTOF) *m/z* calcd for $[C_{90}H_{84}B_4N_6]^+$: 1292.7168 $[M]^+$; found: 1292.7175. Crystal suitable for X-ray diffraction was obtained by vapor diffusion of CH_3CN to a CH_2Cl_2 solution of **2** (CCDC #2165203 – see Table S7).

***N,N'*-(4,6-bis(4,4,5,5-tetramethyl-1,3,2-dioxaborolan-2-yl)-1,3-phenylene)bispivalamide (23)**

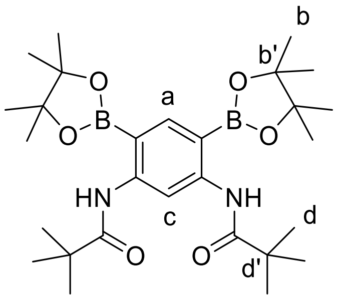

To an oven-dry 50 mL Schlenk tube charged with diiodobispivalamide **22** (528 mg, 1.0 mmol), B_2Pin_2 (1.02 g, 4.0 mmol), $[Pd(dppf)Cl_2]$ (73 mg, 10 mol%) and KOAc (589 mg, 6.0 mmol) under argon, dry DMF (10 mL) was added. The mixture was degassed by bubbling argon for 20 min and stirred at 60 °C for 48 h in closed flask. After allowing it to reach room temperature, the brown suspension was diluted with brine (100 mL) and extracted with CH_2Cl_2 (3 x 100 mL). The combined organic layers were washed with water (2 x 100 mL) and brine (100 mL), then dried over Na_2SO_4 and the solvents evaporated *in vacuo*. The resulting brown solid was purified by silica gel column chromatography (CH_2Cl_2 to CH_2Cl_2/EtOAc 9:1) and recrystallization from boiling CH_2Cl_2/heptane affording **23** as rose needles (222 mg, 42% yield).

mp > 200 °C. ^1H NMR (600 MHz, $CDCl_3$) δ 9.80 (s, 1H, H_c), 9.56 (bs, 2H, N-H), 8.13 (s, 1H, H_a), 1.36 (s, 24H, H_b), 1.30 (s, 18H, H_d). ^{13}C NMR (151 MHz, $CDCl_3$) δ 176.8 (C=O), 149.5 (C-N), 145.1 (C_a), 109.0 (C_c), 84.2 ($C_{b'}$), 40.4 ($C_{d'}$), 27.7 (C_d), 25.1 (C_b) (one signal is missing due to ^{11}B-induced quadrupolar relaxation). ^{11}B NMR (193 MHz, $CDCl_3$) δ 30.2. IR (cm^{-1}): 3369, 2968, 2930, 2907, 2869, 1696, 1619, 1596, 1531, 1476, 1438, 1395, 1371, 1340, 1313, 1276, 1251, 1200, 1169, 1136, 1108, 1088, 1040, 1015, 962, 936, 917, 893, 857, 844, 826, 758, 691, 680, 672, 646, 629, 580, 420. HRMS (ESI) *m/z* calcd for $[C_{28}H_{46}B_2N_2O_6Na]^+$: 551.3444 $[M+Na]^+$; found: 551.3445.

***N,N',N'',N''',N'''',N'''''*-(2,2''''-diamino-[1,1':3',1'':3'',1''':3''',1''''-quinquephenyl]-4',4'',4''',6',6'',6'''-hexayl)hexakispivalamide (7_3)**

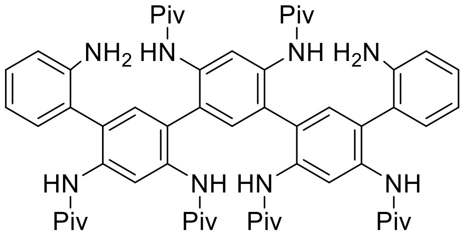

To a 50 mL two-neck round-bottom flask charged with bis(boronpinacolato)bispivalamide **23** (350 mg, 0.66 mmol), iodobiaryl **24** (686 mg, 1.39 mmol), Na_2CO_3 (351 mg, 3.31 mmol), toluene (12 mL), EtOH (4 mL) and water (4 mL) were added. The mixture was degassed by bubbling argon for 20 min, then $[Pd(PPh_3)_4]$ (19 mg, 2.5 mol%) was added under argon flow and 10 min of argon purging were performed again. The flask was equipped with a condenser and the mixture refluxed at 95 °C for 18 h under argon atmosphere. After allowing it to reach room temperature, the yellow suspension was diluted with water (100 mL) and brine (50 mL) and extracted with CH_2Cl_2 (3 x 100 mL). The combined organic layers were dried over Na_2SO_4, and the solvents evaporated *in vacuo*. The resulting yellow solid was purified by silica gel column chromatography (CH_2Cl_2/EtOAc 4:1 to 1:1) affording **7_3** as an off-white solid (664 mg, quantitative yield, mixture of atropoisomers).

mp > 200 °C. ^{1}H NMR (700 MHz, $CDCl_3$) δ 9.08–8.72 (m, 3H), 7.91–7.84 (m, 1H), 7.79–7.66 (m, 1H), 7.59–7.28 (m, 4H), 7.26–7.17 (m, 2H), 7.15–6.96 (m, 5H), 6.89–6.73 (m, 4H), 3.76–3.51 (m, 4H), 1.15–1.00 (m, 54H). ^{13}C NMR (176 MHz, $CDCl_3$) δ 177.3, 177.2, 177.13, 177.06, 176.92, 176.91, 176.88, 176.87, 176.80, 176.77, 176.74, 176.72, 176.69, 176.66, 176.63, 144.02, 143.97, 143.79, 143.76, 143.23, 143.22, 137.0, 136.92, 136.86, 136.84, 136.81, 136.79, 136.75, 136.72, 136.70, 136.68, 136.51, 136.45, 136.2, 132.5, 131.95, 131.90, 131.7, 131.5, 131.35, 131.31, 131.30, 131.28, 131.26, 131.1, 130.8, 130.00, 129.95, 129.86, 127.4, 127.3, 127.2, 127.0, 126.9, 126.8, 126.7, 126.63, 126.56, 126.4, 126.19, 126.16, 125.82, 125.78, 125.7, 125.5, 124.9, 124.6, 122.8, 122.7, 122.4, 122.3, 122.1, 122.0, 119.82, 119.82, 119.7, 119.4, 119.2, 118.8, 118.6, 118.5, 118.3, 118.1, 117.9, 117.7, 117.6, 117.5, 116.7, 116.6, 116.5, 115.78, 115.76, 115.68, 115.66, 39.92, 39.90, 39.85, 39.79, 39.76, 39.7, 39.64, 39.59, 39.52, 39.51, 27.71, 27.70, 27.53, 27.51, 27.48, 27.45, 27.42, 27.37. IR (cm^{-1}): 3425, 3339, 2959, 2929, 2906, 2869, 1696, 1678, 1660, 1618, 1574, 1519, 1480, 1449, 1400, 1383, 1365, 1311, 1290, 1262, 1227, 1199, 1158, 1139, 936, 925, 898, 749, 624, 613, 592, 549, 484. HRMS (ESI) *m/z* calcd for $[C_{60}H_{79}N_8O_6]^+$: 1007.6117 $[M+H]^+$; found: 1007.6118.

[1,1':3',1":3",1"':3"',1""-quinquephenyl]-2,2"",4',4",4"',6',6",6"'-octaamine (8_3)

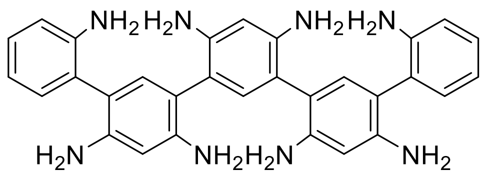

To a 50 mL round-bottom flask charged with **7_3** (650 mg, 0.65 mmol), dioxane (10 mL) and 6 M aq. HCl (10 mL) were added. The flask was equipped with a condenser and the suspension refluxed at 100 °C for 16 h. After allowing it to reach room temperature, the mixture was neutralized with sat. aq. $NaHCO_3$ (200 mL) and extracted with CH_2Cl_2 (4 x 100 mL). The combined organic layers were dried over Na_2SO_4 and the solvent evaporated *in vacuo*. The resulting beige solid was purified by reverse phase preparative HPLC. CH_3CN was evaporated *in vacuo*, the aqueous solution neutralized with sat. aq. $NaHCO_3$ (70 mL) and extracted with CH_2Cl_2 (3 x 100 mL). The combined organic layers were dried over Na_2SO_4 and the solvent evaporated *in vacuo* affording **8_3** as a white solid (240 mg, 74% yield, mixture of atropoisomers).

mp > 200 °C. ^{1}H NMR (600 MHz, DMSO-d_6) δ 7.02–6.87 (m, 4H), 6.74–6.67 (m, 2H), 6.61–6.47 (m, 5H), 6.19–6.10 (m, 3H), 4.66–4.23 (m, 16H). ^{13}C NMR (151 MHz, DMSO-d_6) δ 145.7, 145.6, 145.5, 145.4, 145.3, 145.2, 145.0, 144.9, 144.8, 144.54, 144.50, 133.2, 133.0, 132.9, 132.84, 132.77, 132.7, 130.94, 130.89, 127.3, 124.8, 124.7, 124.6, 116.7, 116.6, 115.2, 114.9, 114.80, 114.75, 114.65, 114.57, 114.5, 114.4, 114.15, 114.09, 113.97, 113.92, 101.20, 101.17, 39.5. IR (cm^{-1}): 3415, 3347, 3188, 2981, 1619, 1610, 1595, 1559, 1502, 1485, 1450, 1421, 1406, 1316, 1293, 1267, 1248, 1201, 1156, 1138, 836, 751, 694, 664, 651, 637, 603, 583, 547, 531, 519, 471. HRMS (ESI) *m/z* calcd for $[C_{30}H_{31}N_8]^+$: 503.2666 [M+H]$^+$; found: 503.2664.

***N*²,*N*²'''',*N*⁴',*N*⁴'',*N*⁴''',*N*⁶',*N*⁶'',*N*⁶'''-octamesityl-[1,1':3',1'':3'',1''':3''',1''''-quinquephenyl]-2,2'''',4',4'',4''',6',6'',6'''-octaamine (9$_3$)**

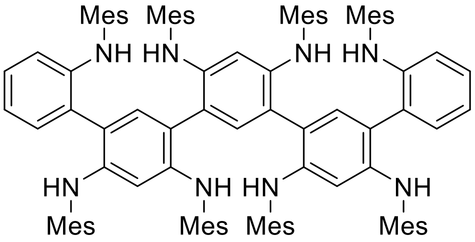

In the glove box, to a 25 mL Schlenk tube with PTFE screwcap charged with [$Pd_2(dba)_3$] (15 mg, 4.0 mol%), *rac*-BINAP (20 mg, 8 mol%), *t*-BuONa (923 mg, 9.6 mmol), **8$_3$** (201 mg, 0.40 mmol), 2-bromomesitylene (1.96 mL, 12.8 mmol) and toluene (12 mL) were added. The mixture was stirred at 110 °C for 24 h in closed flask. After allowing the mixture to reach room temperature, [$Pd_2(dba)_3$] (15 mg, 4.0 mol%), *rac*-BINAP (20 mg, 8 mol%) and toluene (2 mL) were added and the mixture was stirred at 120 °C for additional 48 h in closed flask. After allowing it to reach room temperature, the orange suspension was diluted with water (100 mL) and brine (50 mL), and extracted with CH_2Cl_2 (3 x 150 mL). The combined organic layers were dried over Na_2SO_4, and the solvent evaporated *in vacuo*. The resulting brown oil was purified by silica gel column chromatography (heptane to heptane/CH_2Cl_2 7:3) affording **9$_3$** as a white solid (442 mg, 76% yield, mixture of atropoisomers).
mp > 200 °C. ^{1}H NMR (600 MHz, $CDCl_3$) δ 7.39–7.28 (m, 1H), 7.25–7.00 (m, 6H), 6.96–6.56 (m, 18H), 6.26–6.16 (m, 2H), 5.55–5.21 (m, 6H), 5.13–4.91 (m, 5H), 2.48–1.79 (m, 72H). ^{13}C NMR (151 MHz, $CDCl_3$) δ 146.4, 146.33, 146.27, 146.2, 146.1, 146.0, 145.9, 145.8, 145.6, 145.5, 145.43, 145.41, 145.32, 145.25, 136.6, 136.39, 136.36, 136.31, 136.26, 136.23, 136.20, 136.15, 136.09, 136.03, 135.98, 135.93, 135.8, 135.6, 135.5, 135.44, 135.38, 135.3, 134.93, 134.87, 134.84, 134.78, 134.3, 134.1, 133.7, 133.5, 133.6, 133.1, 133.0, 131.8, 131.7, 131.6, 131.5, 131.4, 129.3, 129.2, 129.1, 128.73, 128.70, 128.67, 128.63, 128.61, 128.57, 128.55, 128.50, 124.7, 124.6, 124.43, 124.40, 124.36, 124.2, 117.6, 117.51, 117.47, 117.4, 117.3, 114.6, 114.5, 114.3, 114.2, 114.1, 113.81, 113.75, 113.5, 113.4, 113.3, 113.2, 113.1, 111.31, 111.25, 111.19, 111.15, 95.41, 95.35, 95.29, 95.25, 21.1, 21.0, 20.9, 18.52, 18.47, 18.44, 18.41, 18.39, 18.35, 18.32, 18.27, 18.24, 18.18. IR (cm^{-1}): 3398, 3001, 2967, 2945, 2914, 2855, 1616, 1596, 1564, 1507, 1483, 1451, 1401, 1394, 1374, 1325, 1311, 1280, 1240, 1226, 1185, 1154, 1034, 1009, 907, 853, 837, 745, 627, 532, 471, 445. HRMS (MALDI-timsTOF, matrix: DCTB) *m/z* calcd for $[C_{102}H_{110}N_8]^+$: 1447.8880 [M]$^+$; found: 1447.8963.

Diphenyl-B_5N_8-heptacenohexacene (3)

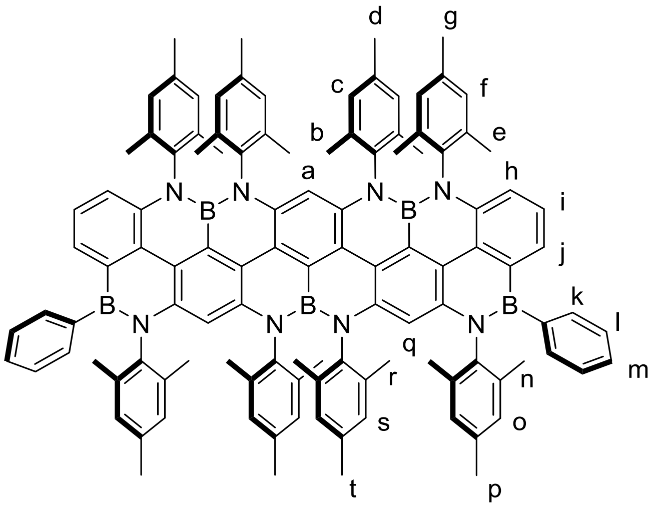

In the glove box, to a 10 mL Schlenk tube with PTFE screwcap, BBr_3 (147 µL, 1.55 mmol) was added dropwise to a stirring solution of **9₃** (50 mg, 0.035 mmol) in TCB (1 mL). The brown suspension was stirred at 270 °C (sand bath) for 20 h in closed flask. After allowing the orange suspension to reach room temperature, all volatiles were removed *in vacuo*. In the glove box, THF (1 mL) and PhMgBr (16% in THF, 5.07 mL, 4.66 mmol) were added and the orange suspension was stirred at 50 °C for 16 h. The solution was concentrated under a flow of argon, quenched with water (70 mL), diluted with 1 M aq. HCl (5 mL) and extracted with CH_2Cl_2 (3 x 100 mL). The combined organic layers were dried over Na_2SO_4, and the solvents evaporated *in vacuo*. The resulting brown solid was purified by silica gel column chromatography (heptane to heptane/CH_2Cl_2 7:3) in the dark and under argon affording **3** as a dark yellow solid (26 mg, 46% yield over two steps). The solid was further purified by sonication in CH_3CN and heptane to provide the analytically pure product.

mp > 260 °C. ^{1}H NMR (600 MHz, C_6D_6/CS_2 1:1) δ 7.56 (dd, *J* = 7.0, 3.2, 2H, H_j), 7.36–7.27 (m, 4H, H_k), 7.08–6.97 (m, 8H, H_l, H_m, H_i), 6.54 (dd, *J* = 8.2, 3.2, 2H, H_h), 6.45 (s, 4H, H_f), 6.42 (s, 4H, H_o), 6.24 (s, 4H, H_c), 6.21 (s, 4H, H_s), 6.02 (s, 2H, H_q), 5.72 (s, 1H, H_a), 2.19 (s, 6H, H_d), 2.16 (s, 6H, H_g), 2.11 (s, 6H, H_t), 2.07 (s, 6H, H_p), 1.84 (s, 12H, H_e), 1.71 (s, 12H, H_n), 1.69 (s, 12H, H_r), 1.64 (s, 12H, H_b). ^{13}C NMR (151 MHz, C_6D_6/CS_2 1:1) δ 141.5, 140.8, 140.7, 139.7, 139.4, 139.3, 139.1, 137.7, 136.5, 136.1, 136.0, 135.7, 135.3, 134.8, 134.7, 134.6, 132.8 (C_k), 129.9 (C_j), 129.2 (C_f), 129.0 (C_o), 128.7 (C_c), 128.6 (C_s), 128.4, 127.6, 127.5 (C_m), 127.2 (C_l), 126.2 (C_i), 122.9, 121.2, 121.0, 117.2 (C_h), 106.3 (C_a), 106.1 (C_q), 21.35 (C_d, C_g, C_p or C_t), 21.33 (C_d, C_g, C_p or C_t), 21.30 (C_d, C_g, C_p or C_t), 21.28 (C_d, C_g, C_p or C_t), 19.1 (C_e), 19.0 (C_b), 18.9 (C_r), 18.10 (C_n) (four signals are missing due to ^{11}B-induced quadrupolar relaxation). ^{11}B NMR (193 MHz, C_6D_6/CS_2 1:1) δ 38.3, 27.9 (two boron signals overlap). IR (cm^{-1}): 3003, 2948, 2916, 2852, 1735, 1598, 1579, 1547, 1480, 1434, 1394, 1372, 1347, 1325, 1301, 1284, 1262, 1222, 1181, 1158, 1119, 1029, 1011, 939, 903, 868, 844, 802, 777, 754, 738, 702, 669, 641, 607, 590, 565, 547, 531, 501, 473, 427. HRMS (LD-timsTOF) *m/z* calcd for $[C_{114}H_{107}B_5N_8]^+$: 1642.9139 $[M]^+$; found: 1642.9173. Crystal suitable for X-ray diffraction was obtained by vapor diffusion of MeOH to a toluene solution of **3** (CCDC #2173120 – see Table S8).

_N,N'_-(2'-amino-5-(4,4,5,5-tetramethyl-1,3,2-dioxaborolan-2-yl)-[1,1'-biphenyl]-2,4-diyl)bispivalamide (25)

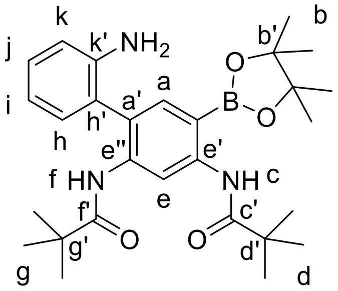

To an oven-dry 100 mL Schlenk tube charged with dry iodobiaryl **24** (1.20 g, 2.43 mmol), B_2Pin_2 (1.30 g, 5.11 mmol), [Pd(dppf)Cl_2] (178 mg, 10 mol%) and KOAc (1.19 g, 12.2 mmol) under argon, dry and degassed DMF (25 mL) was added. The mixture was stirred at 95 °C for 48 h in closed flask. After allowing it to reach room temperature, the crude product was filtered through silica (washing with CH_2Cl_2) and the solvents evaporated *in vacuo*. The resulting brown solid was purified by recrystallization from boiling CH_3CN affording **25** as white crystalline solid (720 mg, 58% yield).
mp > 200 °C. ^{1}H NMR (600 MHz, $CDCl_3$) δ 9.41 (bs, 1H, H_c), 9.38 (d, *J* = 1.6, 1H, H_e), 7.73 (bs, 1H, H_f), 7.64 (s, 1H, H_a), 7.19 (ddd, *J* = 8.0, 7.6, 1.6, 1H, H_j), 7.06 (dd, *J* = 7.4, 1.6, 1H, H_h), 6.83 (ddd, *J* = 7.6, 7.4, 1.1, 1H, H_i), 6.77 (dd, *J* = 8.0, 1.1, 1H, H_k), 3.58 (bs, 2H, NH_2), 1.36 (s, 6H, H_b), 1.35 (s, 6H, H_b), 1.33 (s, 9H, H_d), 1.06 (s, 9H, H_g). ^{13}C NMR (151 MHz, $CDCl_3$) δ 176.9 ($C_{c'}$), 176.4 ($C_{f'}$), 145.5 ($C_{e'}$ or $C_{e''}$), 144.0 ($C_{k'}$), 139.9 ($C_{e'}$ or $C_{e''}$), 138.0 (C_a), 131.5 (C_h), 129.5 (C_j), 123.7 ($C_{a'}$), 123.1 ($C_{h'}$), 119.2 (C_i), 115.4 (C_k), 112.2 (C_e), 84.2 ($C_{b'}$), 40.2 ($C_{d'}$), 39.9 ($C_{g'}$), 27.7 (C_g), 27.4 (C_d), 25.14 (C_b), 25.11 (C_b). ^{11}B NMR (193 MHz, $CDCl_3$) δ 29.1. IR (cm^{-1}): 3407, 3374, 3322, 2976, 2961, 2930, 2868, 1694, 1619, 1589, 1533, 1496, 1476, 1446, 1414, 1380, 1349, 1291, 1256, 1208, 1163, 1136, 1106, 1063, 1025, 961, 922, 898, 859, 844, 827, 770, 758, 747, 704, 671, 631, 579, 532, 502. HRMS (LD-timsTOF) *m/z* calcd for $[C_{28}H_{40}BN_3O_4]^+$: 516.2989 $[M]^+$; found: 516.3009.

_N,N',N'',N'''_-(2''-amino-5-iodo-[1,1':3',1''-terphenyl]-2,4,4',6'-tetrayl)tetrakispivalamide (10)

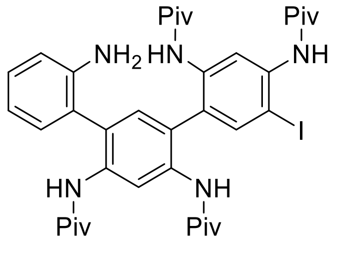

To a 250 mL Schlenk tube charged with **25** (680 mg, 1.38 mmol), diiodobispivalamide **22** (873 mg, 1.65 mmol) and Na_2CO_3 (730 mg, 6.89 mmol), toluene (21 mL), EtOH (7 mL) and water (7 mL) were added. The mixture was degassed by three evacuate-refill cycles, then [Pd$(PPh_3)_4$] (41 mg, 2.5 mol%) was added and three evacuate-refill cycles were performed again. The mixture was stirred at 95 °C for 20 h in closed flask. After allowing it to reach room temperature, the mixture was diluted with water (50 mL) and extracted with CH_2Cl_2 (3 x 100 mL). The combined organic layers were washed

with brine (100 mL), dried over Na_2SO_4 and the solvents evaporated *in vacuo*. The resulting dark yellow residue was purified by silica gel column chromatography (CH_2Cl_2 to CH_2Cl_2/EtOAc 4:1) and recrystallization from boiling heptane/CH_2Cl_2 affording **10** as a white powder (724 g, 68% yield, mixture of atropoisomers).

mp 180–182 °C dec. ^{1}H NMR (600 MHz, $CDCl_3$) δ 9.09–8.72 (m, 2H), 7.92–7.70 (m, 2H), 7.64–7.54 (m, 1H), 7.47–7.28 (m, 2H), 7.22 (ddd, *J* = 8.0, 7.4, 1.6, 1H), 7.11–6.94 (m, 2H), 6.89–6.78 (m, 2H), 3.65 (bs, 1H), 3.57 (bs, 1H), 1.37 (s, 8H), 1.11–1.07 (m, 24H), 1.03 (s, 4H). ^{13}C NMR (151 MHz, $CDCl_3$) δ 177.3, 177.1, 177.0, 176.9, 176.7, 176.6, 143.9, 143.3, 139.5, 139.3, 139.13, 139.11, 137.3, 137.2, 137.1, 136.9, 136.4, 136.1, 131.9, 131.5, 131.4, 130.9, 130.0, 129.9, 127.7, 127.6, 127.3, 126.6, 126.2, 125.0, 122.7, 122.2, 119.8, 119.4, 118.5, 117.7, 116.7, 116.6, 115.8, 115.7, 115.0, 84.21, 84.18, 40.3, 39.84, 39.79, 39.7, 39.6, 27.8, 27.7, 27.53, 27.50, 27.48, 27.4. IR (cm^{-1}): 3393, 3327, 2960, 2905, 2868, 1688, 1664, 1650, 1581, 1557, 1522, 1484, 1459, 1446, 1409, 1383, 1366, 1290, 1206, 1165, 1147, 1015, 941, 925, 890, 871, 780, 762, 586, 561, 483, 425. HRMS (LD-timsTOF) *m/z* calcd for $[C_{38}H_{50}N_5O_4INa]^+$: 790.2777 $[M+Na]^+$; found: 790.2800.

***N*,*N*',*N*'',*N*''',*N*'''',*N*''''',*N*'''''',*N*'''''''-(2,2'''''-diamino-[1,1':3',1'':3'',1''':3''',1'''':3'''',1'''''-sexiphenyl]-4',4'',4''',4'''',6',6'',6''',6''''-octayl)octakispivalamide (7_4)**

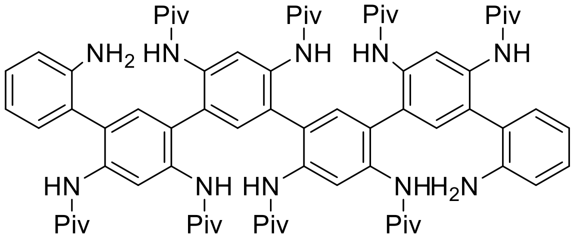

To an oven-dry 50 mL Schlenk tube charged with dry **10** (305 mg, 0.397 mmol), B_2Pin_2 (210 mg, 0.417 mmol), KOAc (117 mg, 1.19 mmol) and $[Pd(dppf)Cl_2]$ (29 mg, 10 mol%), dry and degassed DMF (4 mL) was added. The mixture was stirred at 95 °C for 3 h in closed flask. After allowing it to reach room temperature, the mixture was filtered through Celite (washing with CH_2Cl_2/EtOAc 1:1) and the solvents evaporated *in vacuo*.

To the resulting brown solid, **10** (305 mg, 0.397 mmol), Na_2CO_3 (215 mg, 1.97 mmol), toluene (6 mL), EtOH (2 mL) and water (2 mL) were added. The mixture was degassed by three evacuate-refill cycles, then $[Pd(PPh_3)_4]$ (11 mg, 2.5 mol%) was added and three evacuate-refill cycles were performed again. The mixture stirred at 95 °C for 20 h in closed flask. After allowing it to reach room temperature, the crude product was diluted with water (50 mL) and extracted with CH_2Cl_2 (3 × 100 mL). The combined organic layers were washed with brine (100 mL), dried over Na_2SO_4 and the solvents evaporated *in vacuo*. The resulting brown solid was purified by silica gel column chromatography (CH_2Cl_2/EtOAc 9:1 to 2:3) affording **7_4** as an off-white solid (360 mg, 71% yield over two steps, mixture of atropoisomers).

mp > 200 °C. ^{1}H NMR (600 MHz, $CDCl_3$) δ 9.15–8.65 (m, 4H), 7.99–7.27 (m, 8H), 7.25–6.72 (m, 12H), 4.51–3.48 (m, 4H), 1.16–0.98 (m, 72H). ^{13}C NMR (151 MHz, $CDCl_3$) δ 177.4, 177.21, 177.19, 177.14, 177.08, 177.03, 177.00, 176.97, 176.94, 176.93, 176.89, 176.82, 176.80, 176.79, 176.77, 176.73,

176.70, 176.66, 176.63, 176.61, 176.57, 176.4, 145.3, 144.0, 143.94, 143.89, 143.74, 143.70, 143.34, 143.28, 143.27, 143.24, 143.17, 137.5, 137.2, 137.12, 137.10, 137.04, 136.98, 136.96, 136.93, 136.91, 136.89, 136.87, 136.83, 136.81, 136.45, 136.41, 136.38, 136.36, 136.33, 136.30, 136.25, 136.18, 136.1, 136.03, 135.96, 135.88, 135.85, 135.74, 135.67, 135.65, 135.60, 135.59, 135.2, 132.8, 132.7, 132.62, 132.60, 132.56, 132.53, 132.4, 132.3, 132.22, 132.19, 132.1, 131.99, 131.95, 131.91, 131.8, 131.6, 131.5, 131.45, 131.41, 131.38, 131.32, 131.29, 131.25, 131.19, 131.12, 131.10, 131.07, 131.03, 130.92, 130.88, 130.87, 130.3, 130.09, 130.05, 130.03, 129.99, 129.94, 129.88, 127.6, 127.54, 127.49, 127.47, 127.45, 127.41, 127.39, 127.35, 127.33, 127.2, 127.1, 127.0, 126.91, 126.88, 126.86, 126.82, 126.76, 126.73, 126.72, 126.6, 126.53, 126.49, 126.39, 126.36, 126.3, 126.2, 126.0, 125.9, 125.84, 125.81, 125.78, 125.75, 125.67, 125.60, 125.57, 125.55, 125.52, 125.47, 125.42, 125.41, 125.36, 125.35, 125.33, 125.2, 125.1, 124.3, 124.2, 124.0, 123.8, 123.7, 122.9, 122.75, 122.71, 122.65, 122.58, 122.5, 122.3, 122.11, 122.07, 122.05, 122.02, 121.0, 119.9, 119.83, 119.78, 119.43, 119.39, 119.31, 119.26, 119.2, 118.93, 118.86, 118.7, 118.6, 118.5, 118.2, 118.10, 118.06, 117.7, 117.54, 117.50, 117.45, 117.43, 117.33, 117.31, 116.7, 116.52, 116.47, 116.45, 116.40, 116.3, 116.25, 116.22, 115.9, 115.81, 115.80, 115.75, 115.66, 112.5, 40.04, 40.03, 40.01, 39.97, 39.89, 39.87, 39.85, 39.83, 39.80, 39.78, 39.73, 39.72, 39.69, 39.67, 39.64, 39.61, 39.56, 39.53, 39.52, 39.49, 27.7, 27.6, 27.5, 27.44, 27.42, 27.40, 27.38, 27.36. IR (cm^{-1}): 3424, 3319, 2959, 2906, 2869, 1695, 1678, 1661, 1619, 1573, 1519, 1480, 1452, 1399, 1366, 1308, 1287, 1198, 1158, 1139, 924, 898, 750, 641, 625, 600, 578, 564, 543, 488, 455, 430. HRMS (MALDI-timsTOF, matrix: DCTB) *m/z* calcd for $[C_{76}H_{100}N_{10}O_8]^+$: 1281.7798 $[M]^+$; found: 1281.7789.

[1,1':3',1'':3'',1''':3''',1'''':3'''',1'''''-sexiphenyl]-2,2''''',4',4'',4''',4'''',6',6'',6''',6''''-decaamine (8_4)

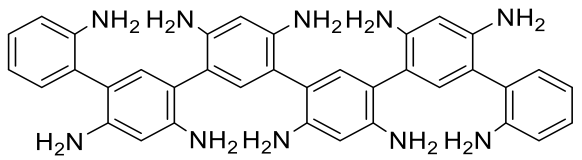

To a 50 mL round-bottom flask charged with **7_4** (330 mg, 0.257 mmol), dioxane (10 mL) and 6 M aq. HCl (10 mL) were added. The flask was equipped with a condenser and the suspension refluxed at 105 °C for 16 h under argon atmosphere. After allowing it to reach room temperature, the mixture was basified with sat. aq. $NaHCO_3$ (until pH 9-10, checked with pH paper) and extracted with EtOAc (3 x 50 mL). The combined organic layers were washed with brine (70 mL), dried over Na_2SO_4 and the solvent evaporated *in vacuo*. The resulting brown solid was purified by reverse phase preparative HPLC. CH_3CN was evaporated *in vacuo*, the aqueous solution neutralized with sat. aq. Na_2CO_3 (25 mL) and extracted with CH_2Cl_2 (3 x 50 mL). The combined organic layers were dried over Na_2SO_4 and the solvent evaporated *in vacuo* affording **8_4** as a brown powder (120 mg, 44% yield, mixture of atropoisomers).

mp > 200 °C. ^{1}H NMR (600 MHz, DMSO-*d6*) δ 7.04–6.84 (m, 4H), 6.75–6.66 (m, 2H), 6.64–6.47 (m, 6H), 6.21–6.06 (m, 4H), 4.42 (bs, 20H). ^{13}C NMR (151 MHz, DMSO-*d6*) δ 145.7, 145.55, 145.47, 145.44, 145.41, 145.36, 145.32, 145.25, 145.18, 145.12, 145.09, 145.06, 145.0, 144.9, 144.80, 144.77, 144.72, 144.69, 144.5, 133.6, 133.36, 133.34, 133.26, 133.17, 133.13, 133.05, 132.97,

132.95, 132.85, 132.77, 132.73, 132.71, 132.68, 130.9, 127.3, 124.8, 124.71, 124.67, 124.66, 124.64, 124.60, 116.73, 116.65, 116.61, 116.60, 115.3, 115.2, 114.93, 114.88, 114.84, 114.81, 114.75, 114.74, 114.62, 114.49, 114.48, 114.39, 114.37, 114.2, 114.09, 114.05, 114.02, 113.99, 113.9, 101.2. IR (cm^{-1}): 3444, 3345, 3200, 1619, 1610, 1561, 1501, 1487, 1451, 1422, 1408, 1316, 1292, 1268, 1249, 1203, 1158, 1137, 838, 753, 720, 703, 680, 667, 655, 637, 611, 600, 584, 541, 526, 476, 464, 454. HRMS (ESI) *m/z* calcd for $[C_{36}H_{37}N_{10}]^+$: 609.3197 $[M+H]^+$; found: 609.3187.

N^{2},$N^{2'''''}$,$N^{4'}$,$N^{4''}$,$N^{4'''}$,$N^{4''''}$,$N^{6'}$,$N^{6''}$,$N^{6'''}$,$N^{6''''}$-octamesityl-[1,1':3',1'':3'',1''':3''',1'''':3'''',1'''''-sexiphenyl]-2,2''''',4',4'',4''',4'''',6',6'',6''',6''''-decaamine (9$_4$)

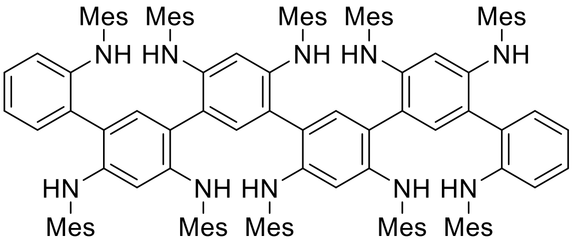

In the glove box, to a 25 mL Schlenk tube with PTFE screwcap charged with $[Pd_2(dba)_3]$ (24.7 mg, 15.0 mol%), *rac*-BINAP (33.6 mg, 30 mol%), *t*-BuONa (415 mg, 4.3 mmol), **8$_4$** (110 mg, 0.18 mmol), 2-bromomesitylene (1.1 mL, 7.2 mmol) and toluene (10 mL) were added. The mixture was stirred at 110 °C for 48 h in closed flask. After allowing the mixture to reach room temperature, $[Pd_2(dba)_3]$ (8.2 mg, 5.0 mol%), *rac*-BINAP (11.2 mg, 10 mol%) and *t*-BuONa (138 mg, 1.4 mmol) were added and the mixture was stirred at 110 °C for additional 24 h in closed flask. After allowing it to reach room temperature, the orange suspension was diluted with water (50 mL) and extracted with CH_2Cl_2 (3 x 30 mL). The combined organic layers were dried over Na_2SO_4, and the solvent evaporated *in vacuo*. The resulting brown oil was purified by silica gel column chromatography (heptane to heptane/CH_2Cl_2 3:2) affording **9$_4$** as a white solid (181 mg, 56% yield, mixture of atropoisomers).
mp 186–188 °C dec. ^{1}H NMR (600 MHz, $CDCl_3$) δ 7.38–7.26 (m, 2H), 7.25–6.47 (m, 30H), 6.28–6.15 (m, 2H), 5.50–4.90 (m, 14H), 2.41–1.85 (m, 88H), 1.36 (s, 2H). ^{13}C NMR (151 MHz, $CDCl_3$) δ 146.3, 146.1, 145.9, 145.7, 145.5, 145.4, 145.3, 136.9, 136.6, 136.43, 136.35, 136.2, 136.13, 136.09, 136.03, 135.98, 135.40, 135.37, 135.3, 134.9, 134.8, 134.73, 134.68, 134.3, 134.1, 133.5, 132.9, 132.3, 131.8, 131.7, 131.6, 129.4, 129.1, 128.8, 128.71, 128.67, 128.62, 128.59, 124.6, 124.5, 124.3, 117.6, 117.4, 117.2, 114.6, 114.3, 114.1, 113.34, 113.29, 111.25, 111.18, 95.3, 31.1, 29.9, 23.8, 21.11, 21.05, 21.03, 20.96, 20.92, 20.88, 20.7, 18.9, 18.50, 18.47, 18.43, 18.40, 18.38, 18.34, 18.28, 18.22, 18.19, 18.16. IR (cm^{-1}): 3399, 3012, 2916, 2856, 1616, 1598, 1563, 1483, 1454, 1401, 1375, 1313, 1279, 1235, 1186, 1036, 1010, 910, 852, 747, 630, 531, 473. HRMS (MALDI-timsTOF, matrix: DCTB) *m/z* calcd for $[C_{126}H_{136}N_{10}]^+$: 1790.0976 $[M]^+$; found: 1790.1016.

Diphenyl-B_6N_{10}-octacenooctacene (4)

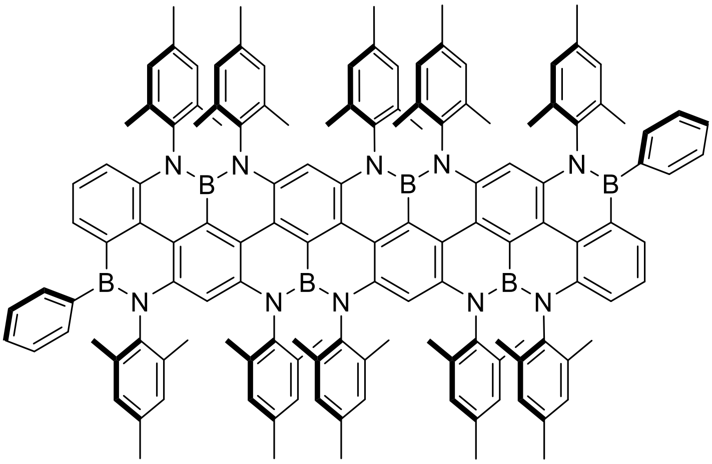

In the glovebox, to a 15 mL flame dried thick-walled Schlenk tube, with PTFE screwcaps, equipped with a glass-coated magnetic stirbar, BBr_3 (142 µL, 1.5 mmol) was added dropwise to stirring solution of **9_4** (45 mg, 0.025 mmol) in TCB (1.5 mL). The mixture was stirred at 330 °C (sand bath) for 16 h in closed flask. After allowing the orange suspension to reach room temperature, all volatiles were removed *in vacuo*. In the glove box, THF (1 mL) and PhMgBr (16% in THF, 4.5 mL, 4.5 mmol) were added and the brown solution was stirred at 50 °C for 16 h. The brown solution was diluted with CH_2Cl_2 and filtered through a Celite plug (washing with CH_2Cl_2). The solution was concentrated to about 200 mL of total volume and filtered through a silica gel plug (CH_2Cl_2). The eluted solution was concentrated *in vacuo* and the crude product was transferred to a plastic Falcon tube. CH_3CN (10 mL) was added and the brown suspension was sonicated for 10 min. After centrifugation (5000 rpm for 20 min), the supernatant was removed and CH_2Cl_2 (10 mL) was added. The suspension was sonicated for 10 min and, after centrifugation (5000 rpm for 20 min), the supernatant was removed and the solvent evaporated *in vacuo*. The crude product was filtered through a silica gel plug (heptane/CH_2Cl_2 1:1 to CH_2Cl_2) under argon and red-light conditions. The solid obtained was transferred to a plastic Falcon tube, resuspended in CH_3CN (10 mL) and sonicated for 10 min. After centrifugation (5000 rpm for 20 min) the supernatant was removed, obtaining a brown solid (5 mg). HRMS (MALDI-timsTOF, matrix: DCTB) *m/z* calcd for $[C_{138}H_{130}B_6N_{10}]^+$: 1993.1103 $[M]^+$; found: 1993.1024.

2. NMR and HRMS spectra

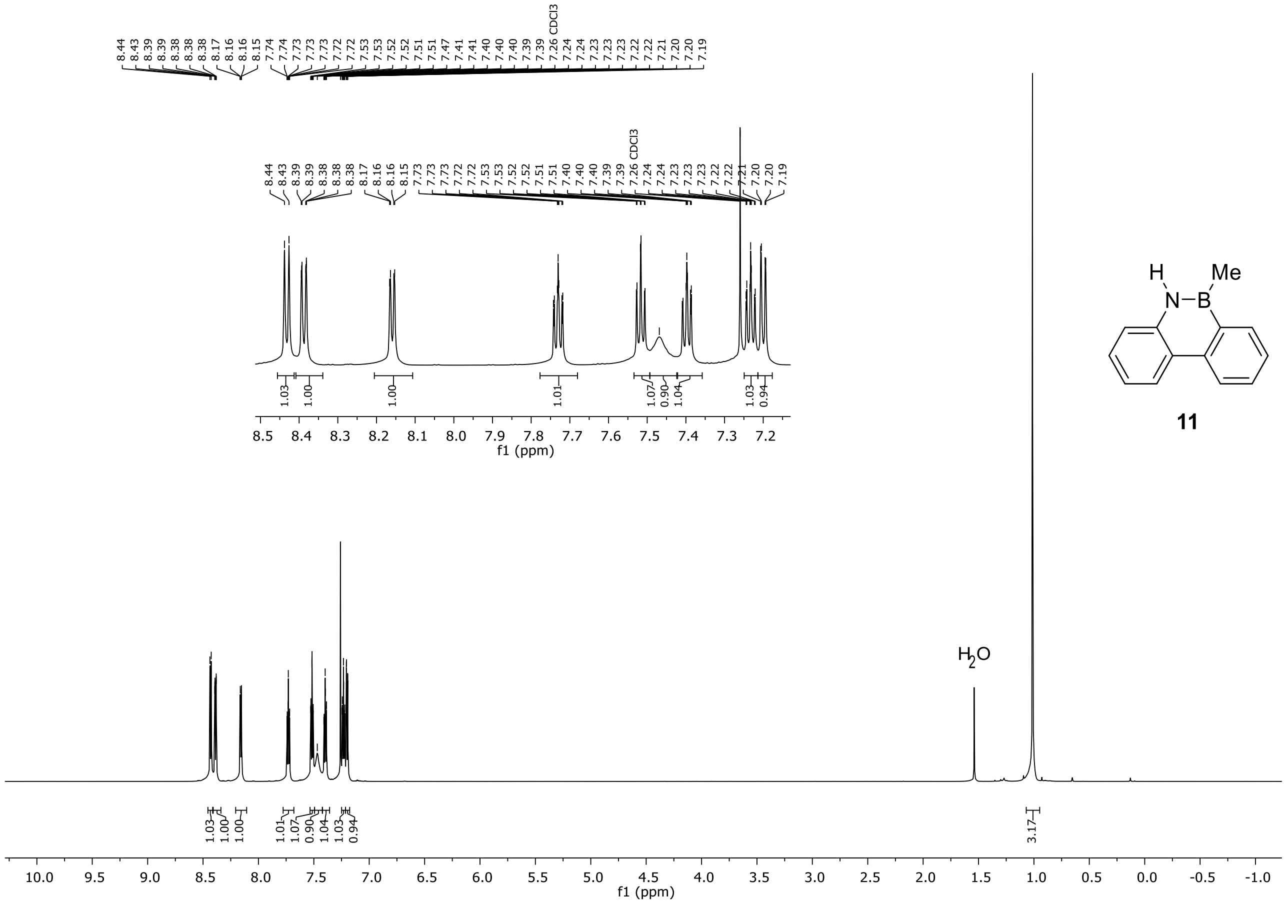

Figure S1. ^{1}H NMR (700 MHz, $CDCl_3$) spectrum of **11**.

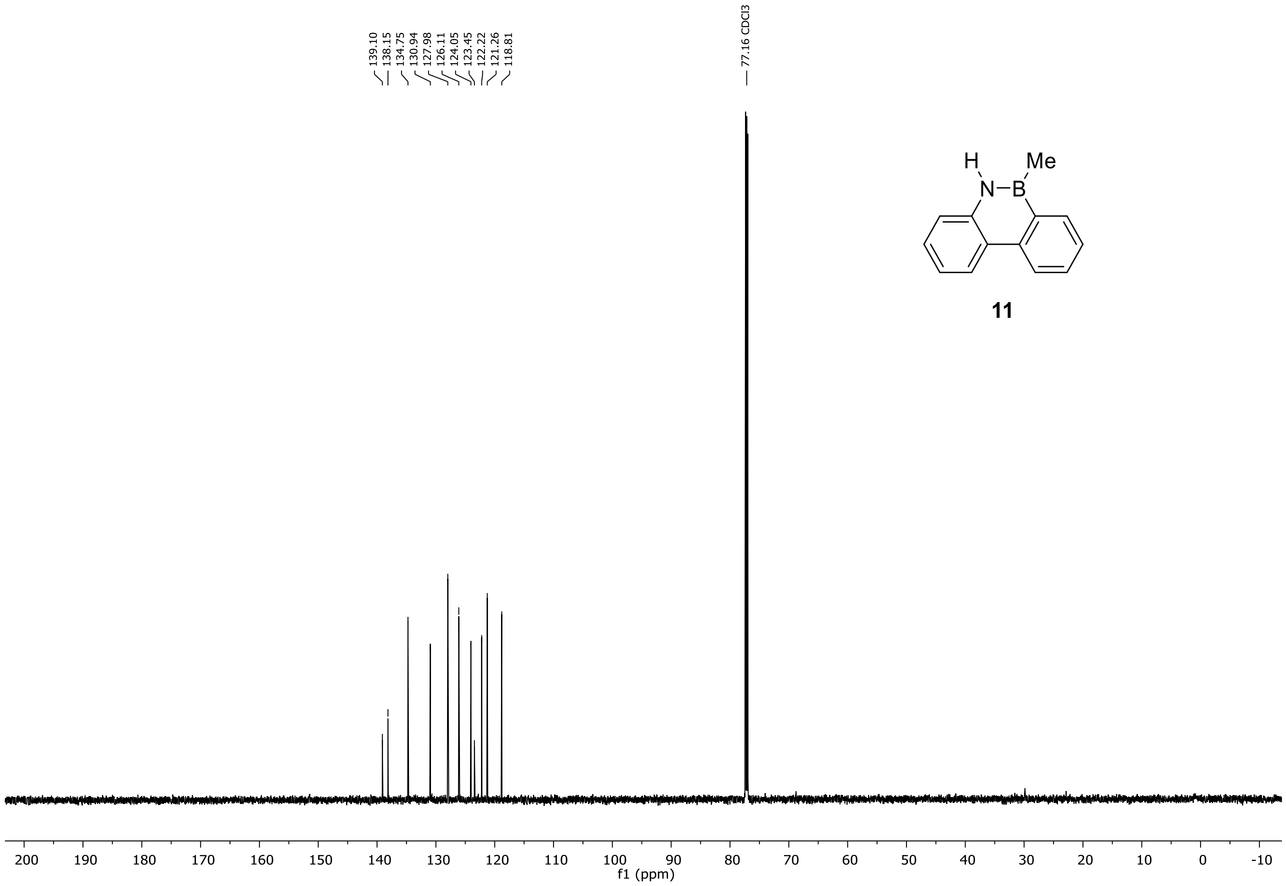

Figure S2. ^{13}C NMR (176 MHz, $CDCl_3$) spectrum of **11**.

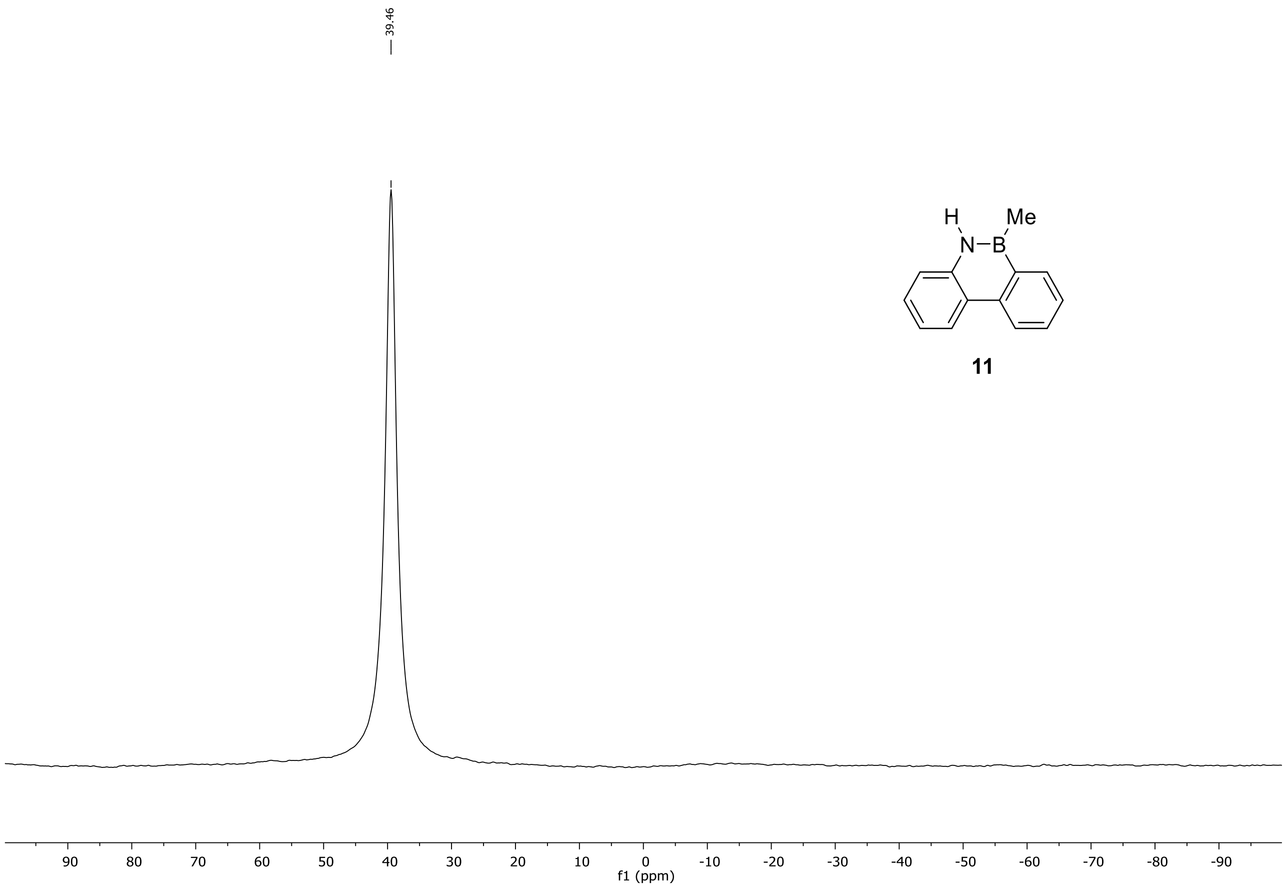

Figure S3. ^{11}B NMR (193 MHz, $CDCl_3$) spectrum of **11**.

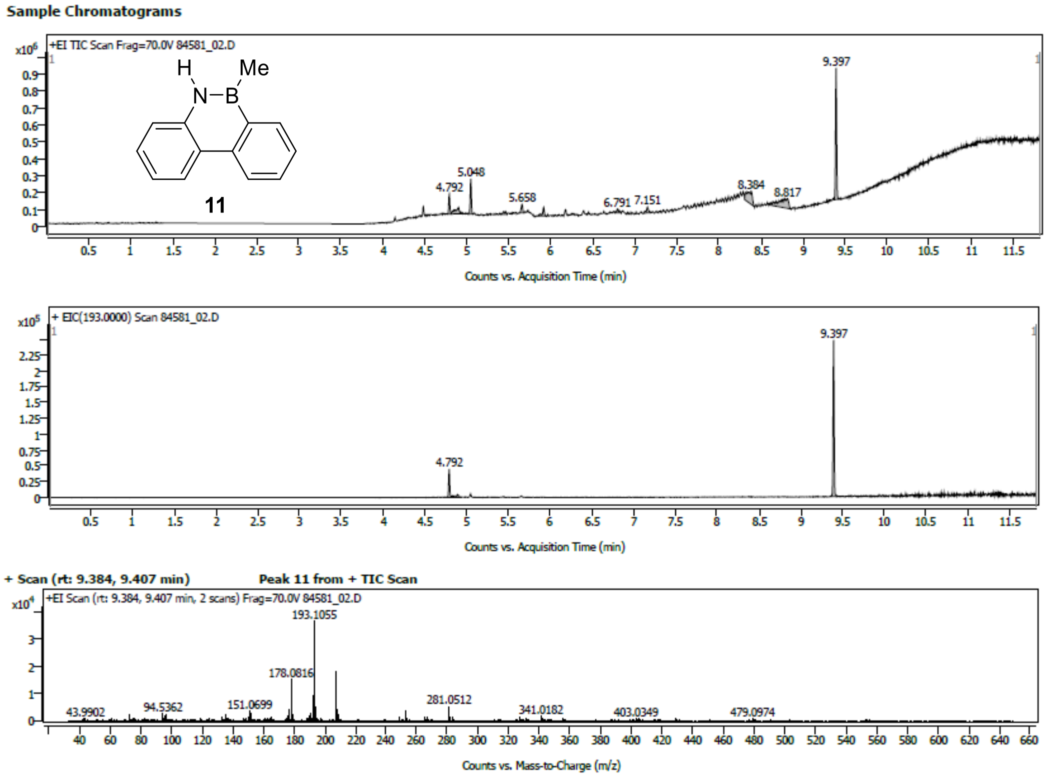

Figure S4. HRMS (GC/Q-TOF) spectrum of **11**.

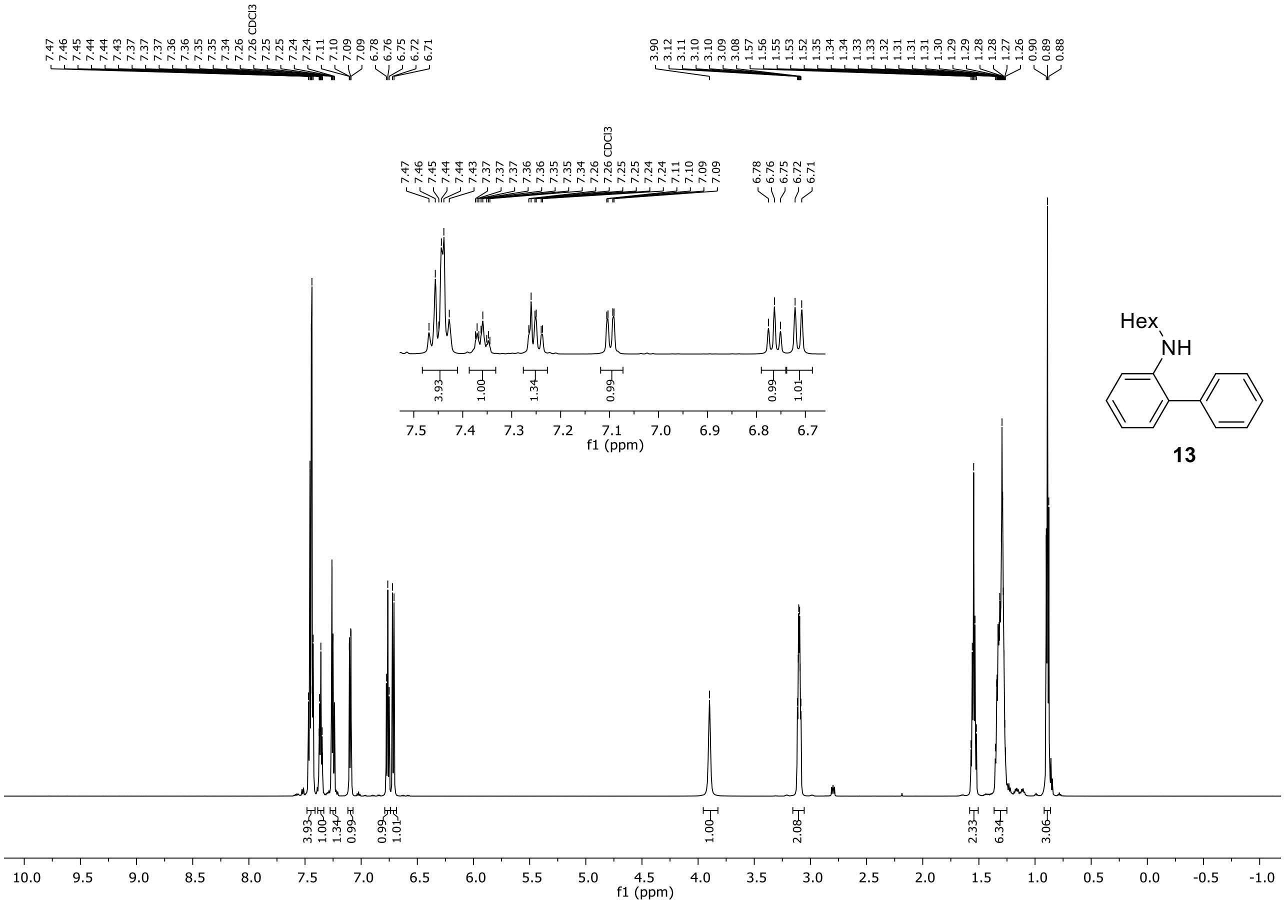

Figure S5. ^{1}H NMR (600 MHz, $CDCl_3$) spectrum of **13**.

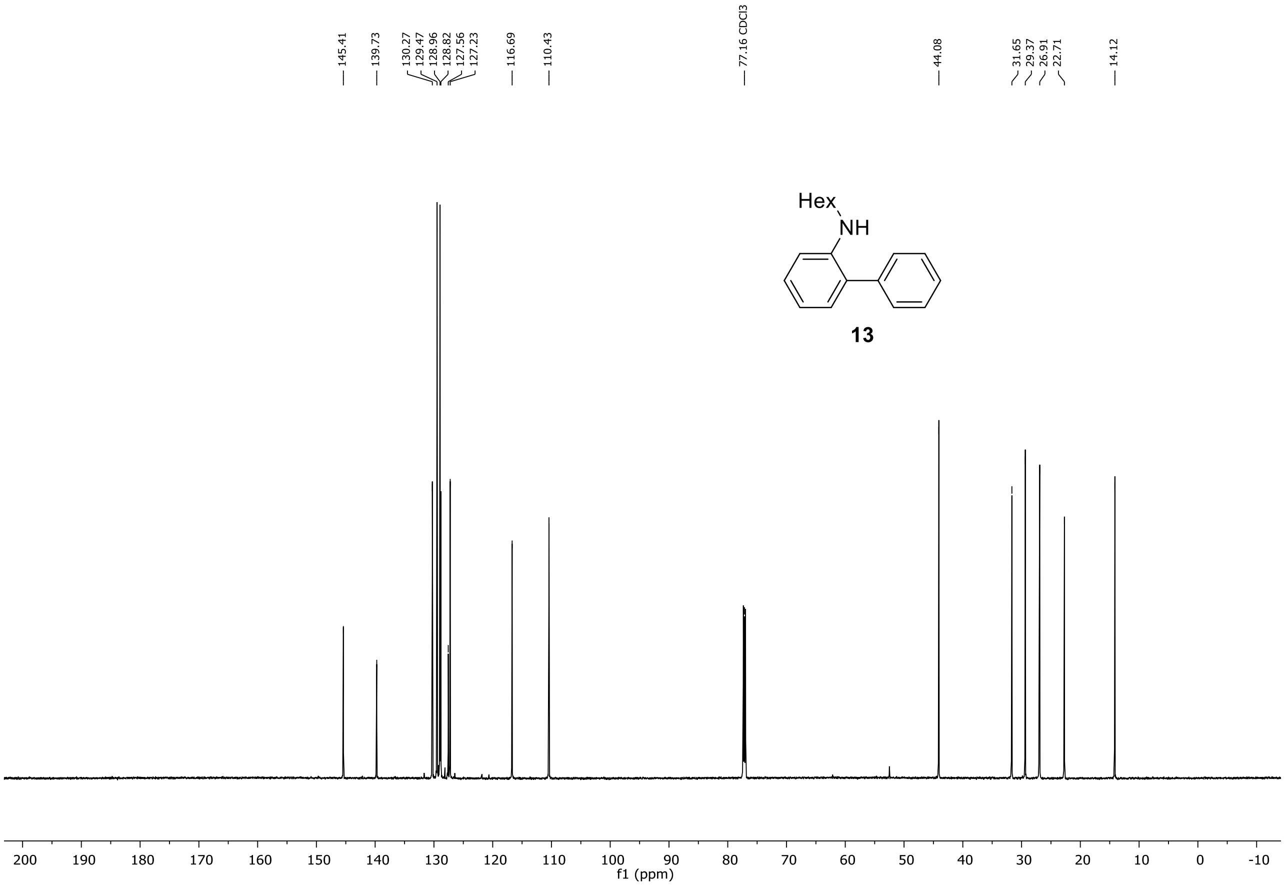

Figure S6. ^{13}C NMR (151 MHz, $CDCl_3$) spectrum of **13**.

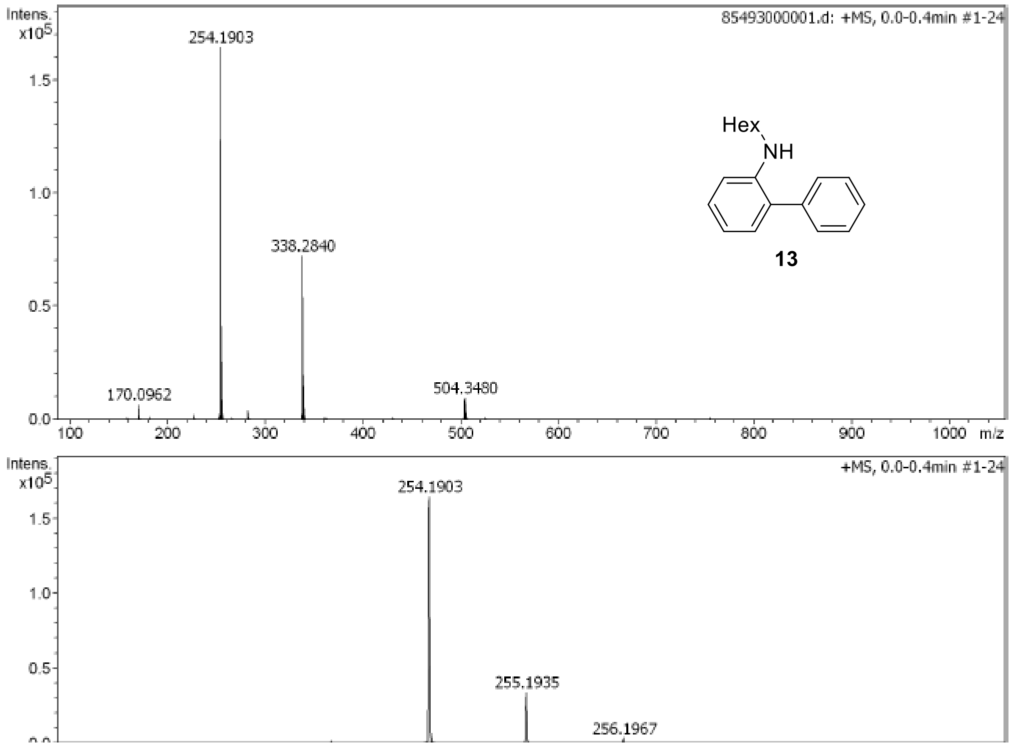

Figure S7. HRMS (ESI) spectrum of **13**.

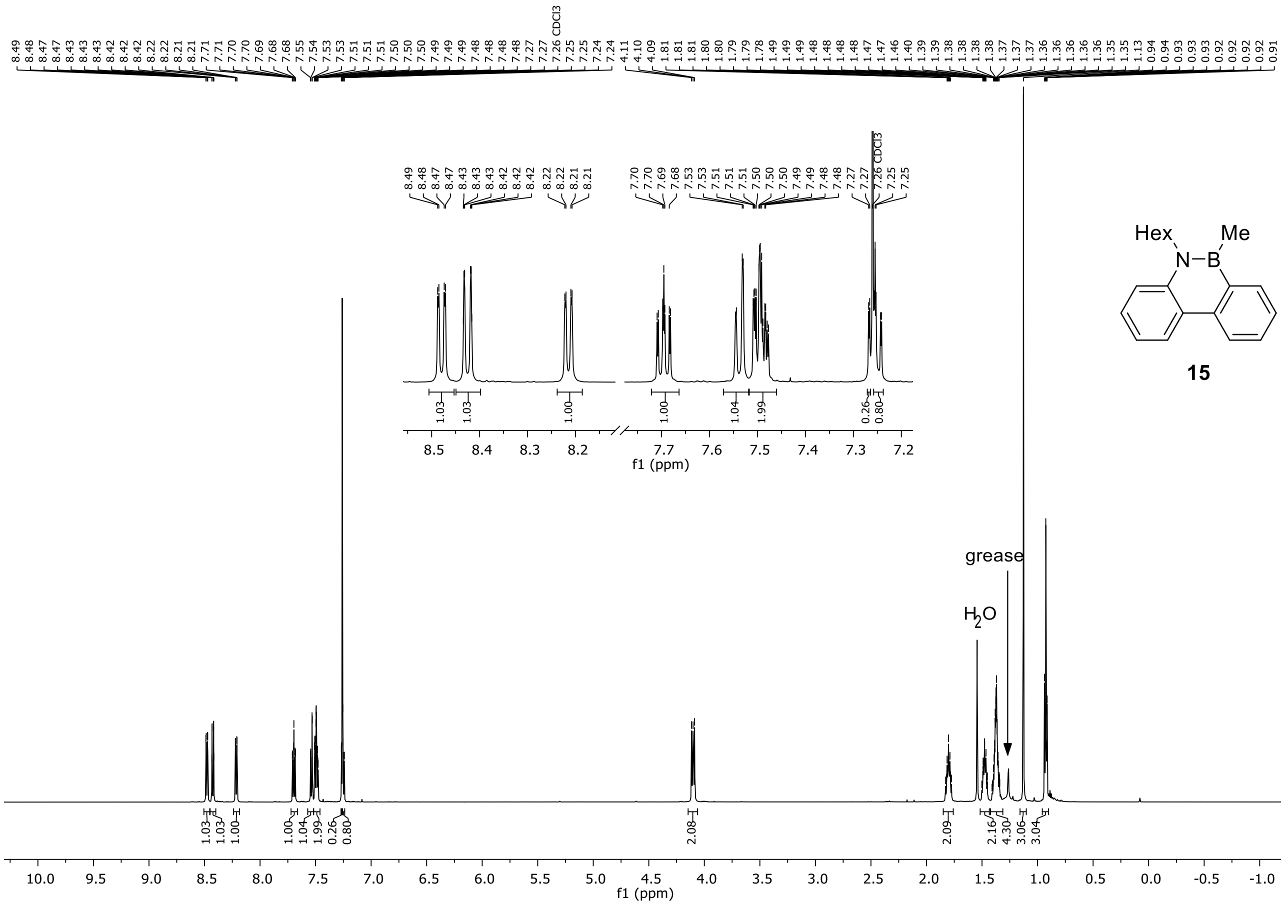

Figure S8. ^{1}H NMR (600 MHz, $CDCl_3$) spectrum of **15**.

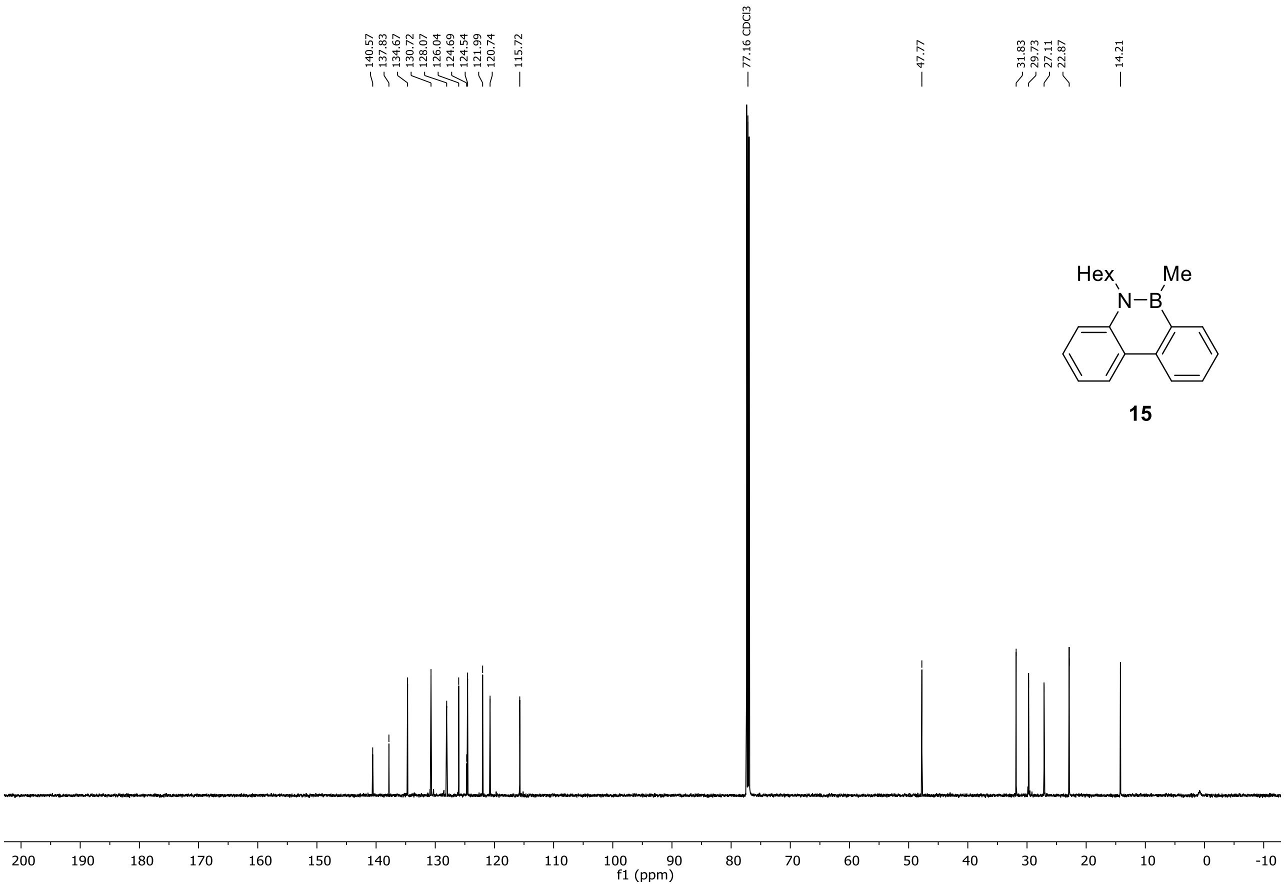

Figure S9. ^{13}C NMR (151 MHz, $CDCl_3$) spectrum of **15**.

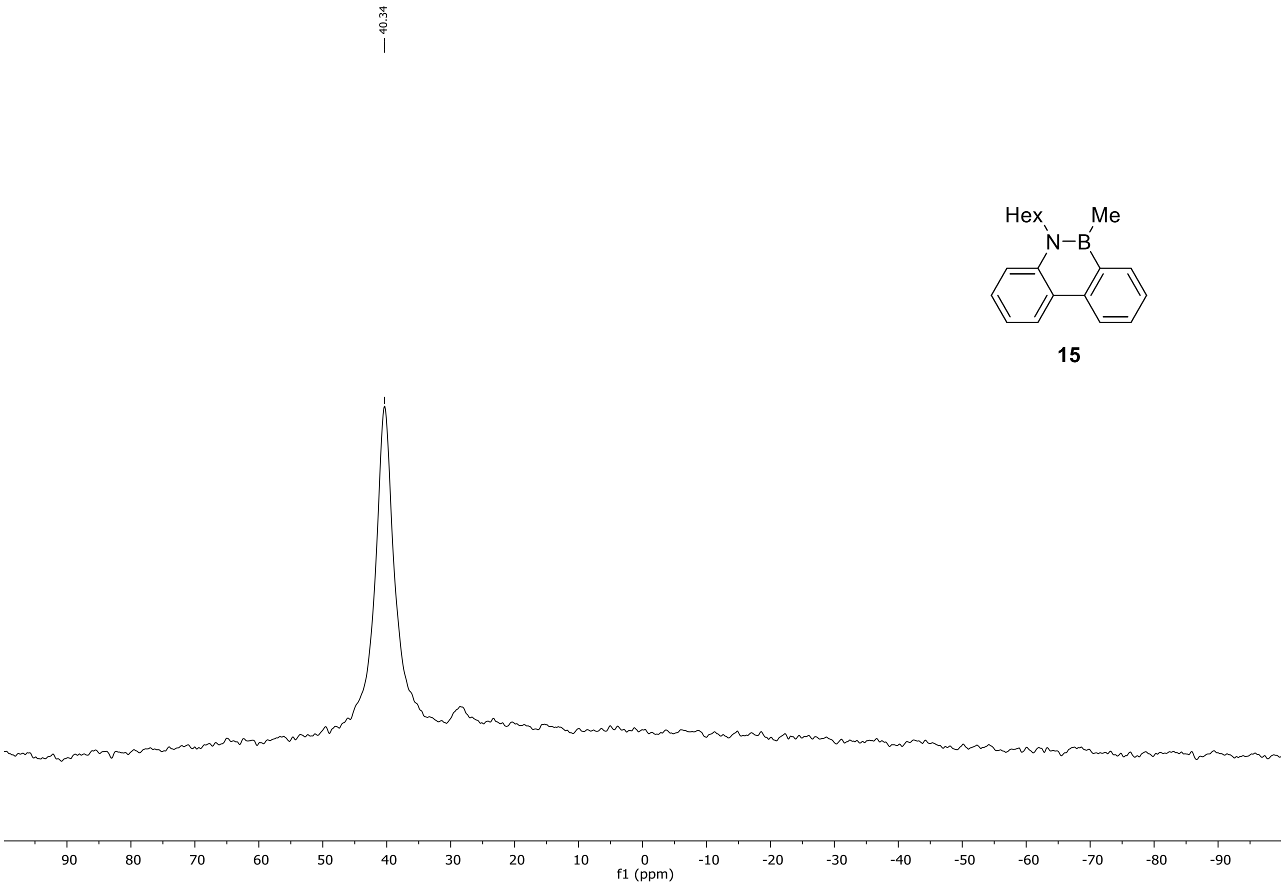

Figure S10. ^{11}B NMR (193 MHz, $CDCl_3$) spectrum of **15**.

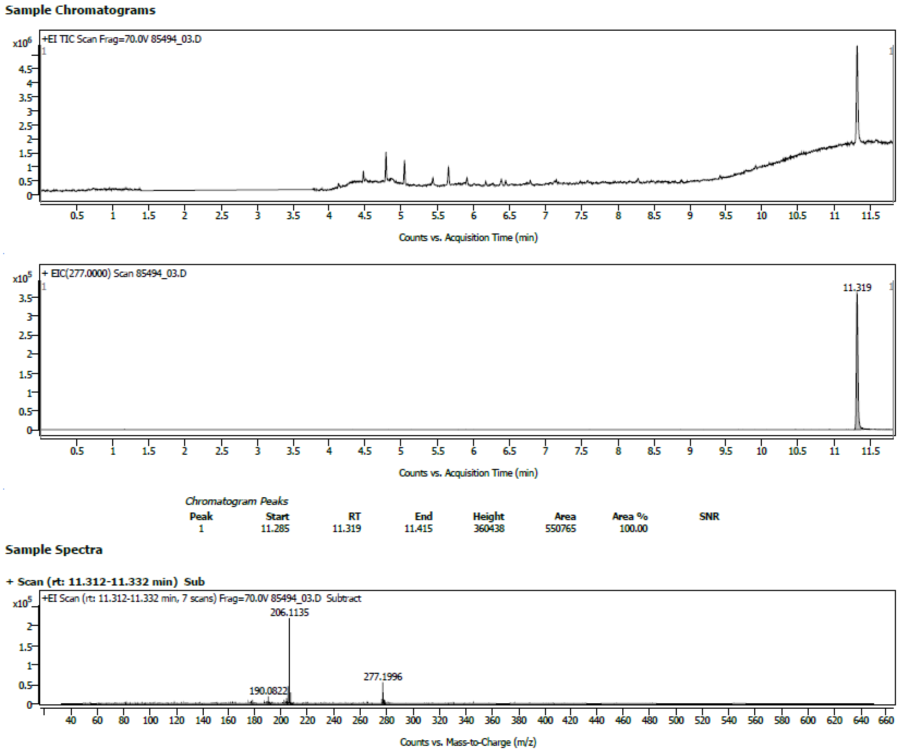

Peak	Start	RT	End	Height	Area	Area %	SNR
1	11.285	11.319	11.415	360438	550765	100.00	

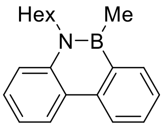

15

Figure S11. HRMS (GC/Q-TOF) spectrum of **15**.

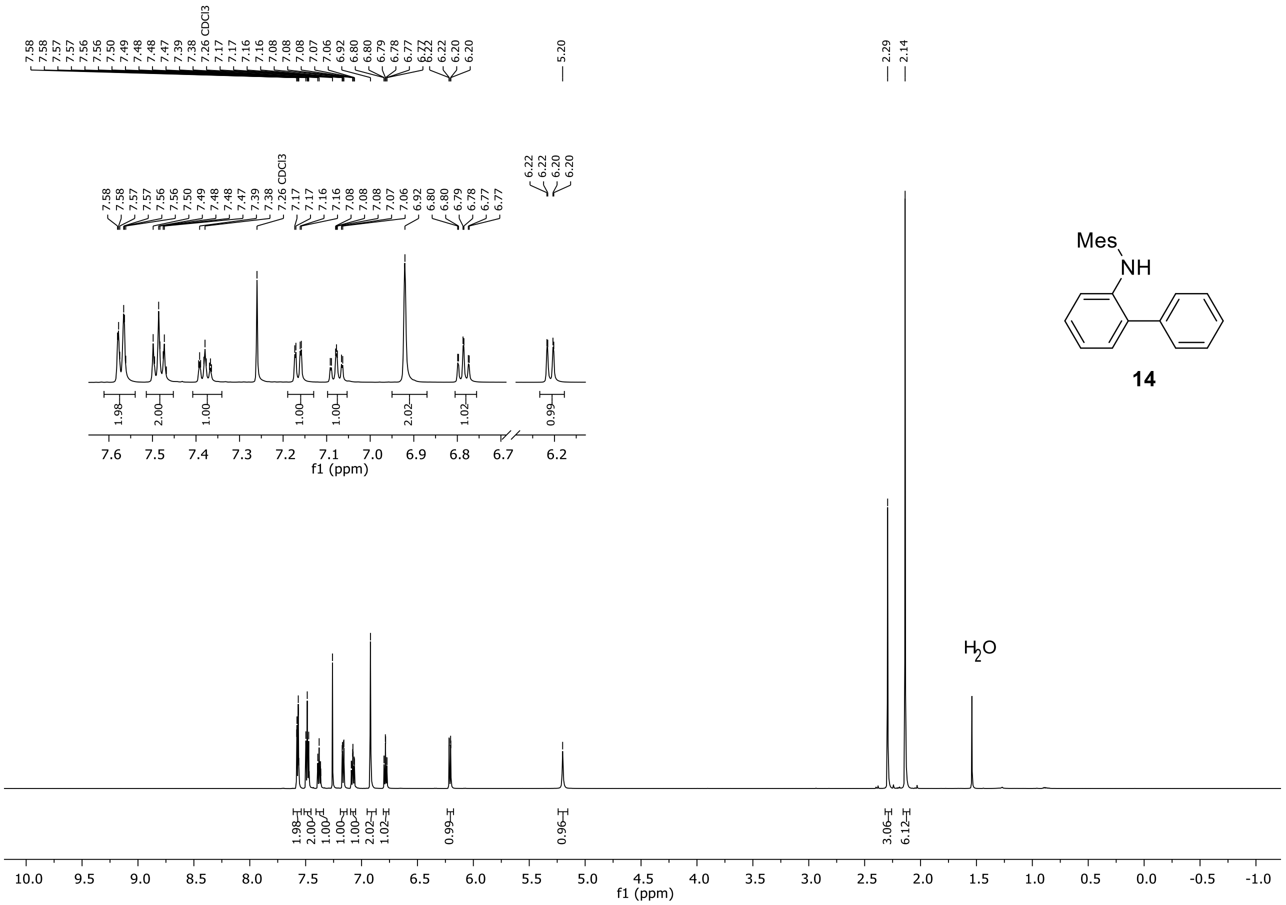

Figure S12. ^{1}H NMR (600 MHz, $CDCl_3$) spectrum of **14**.

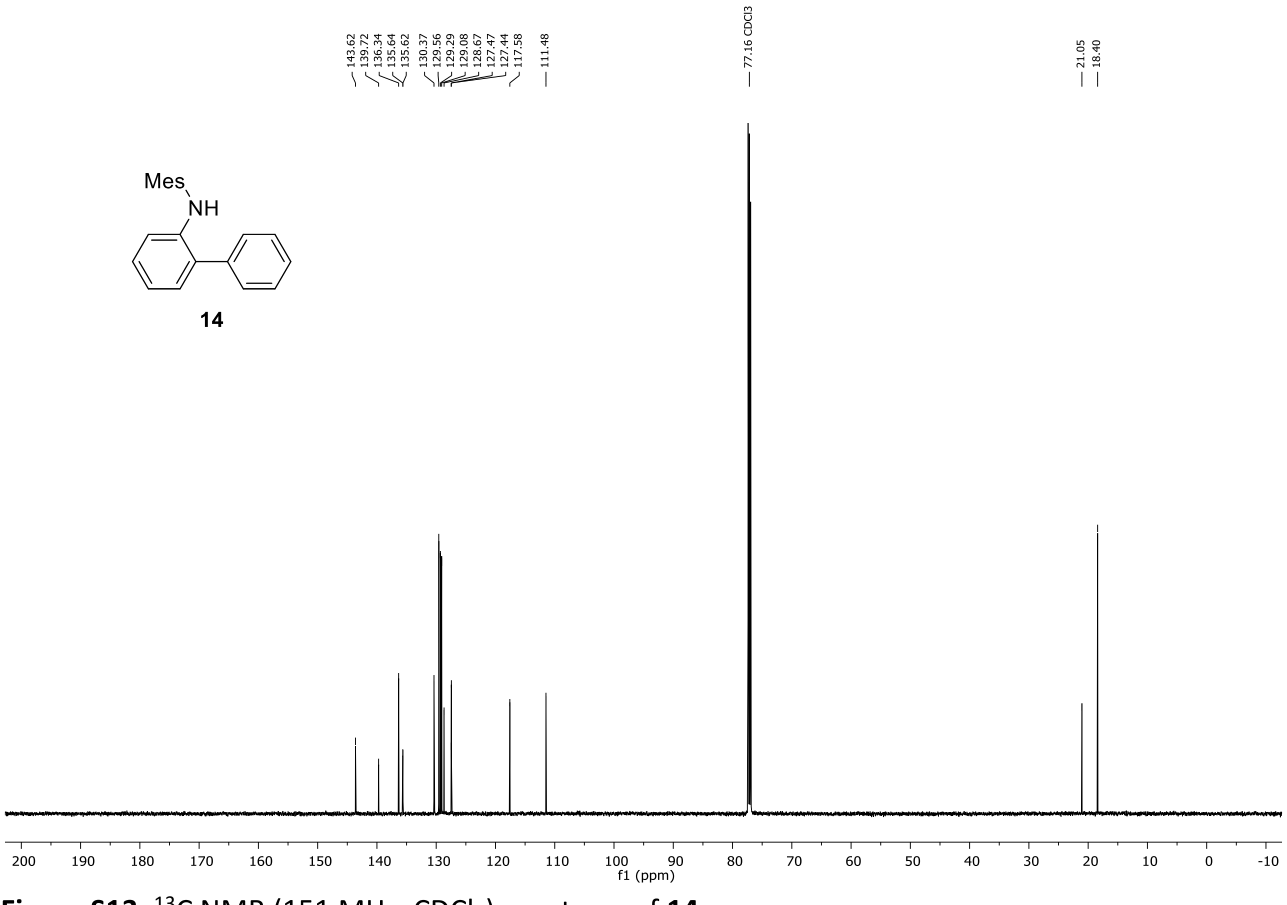

Figure S13. ^{13}C NMR (151 MHz, $CDCl_3$) spectrum of **14**.

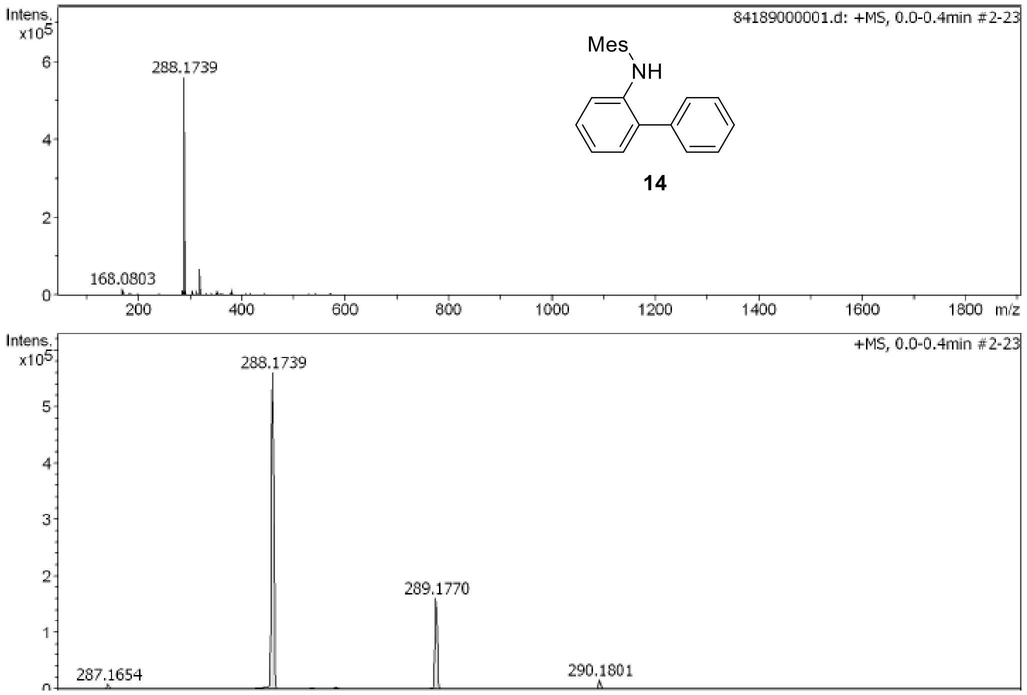

Figure S14. HRMS (ESI) spectrum of **14**.

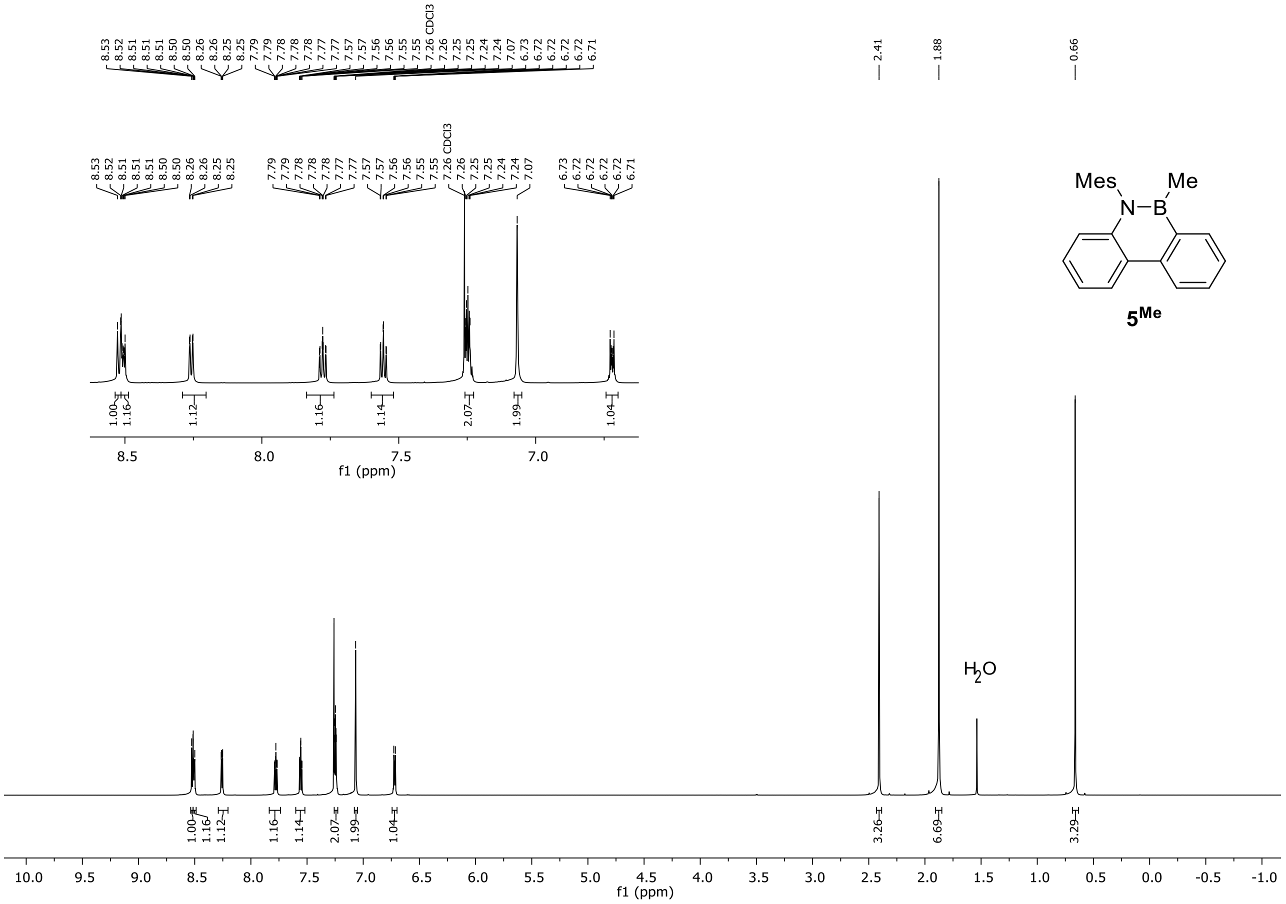

Figure S15. ^{1}H NMR (700 MHz, $CDCl_3$) spectrum of **5^{Me}**.

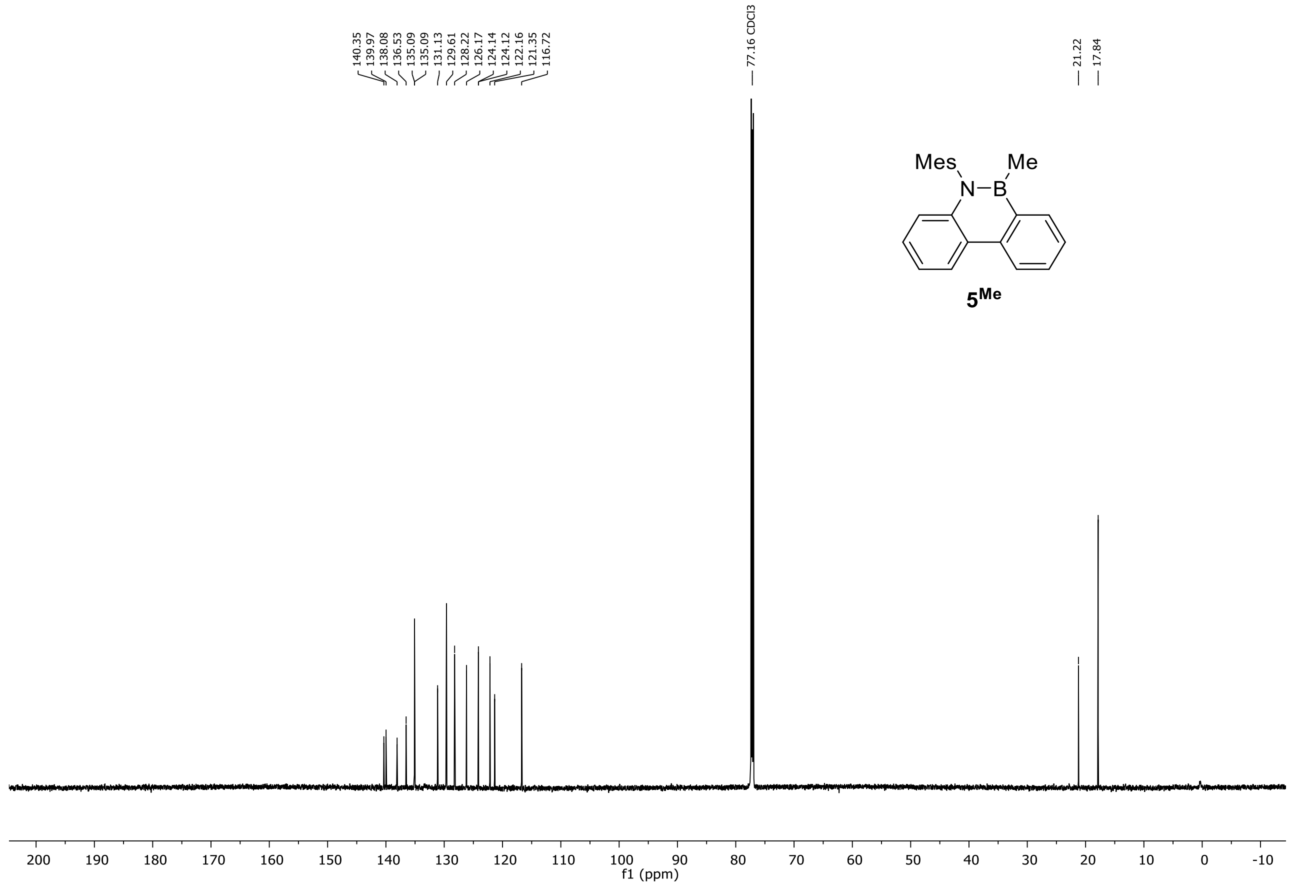

Figure S16. ^{13}C NMR (176 MHz, $CDCl_3$) spectrum of $\mathbf{5^{Me}}$.

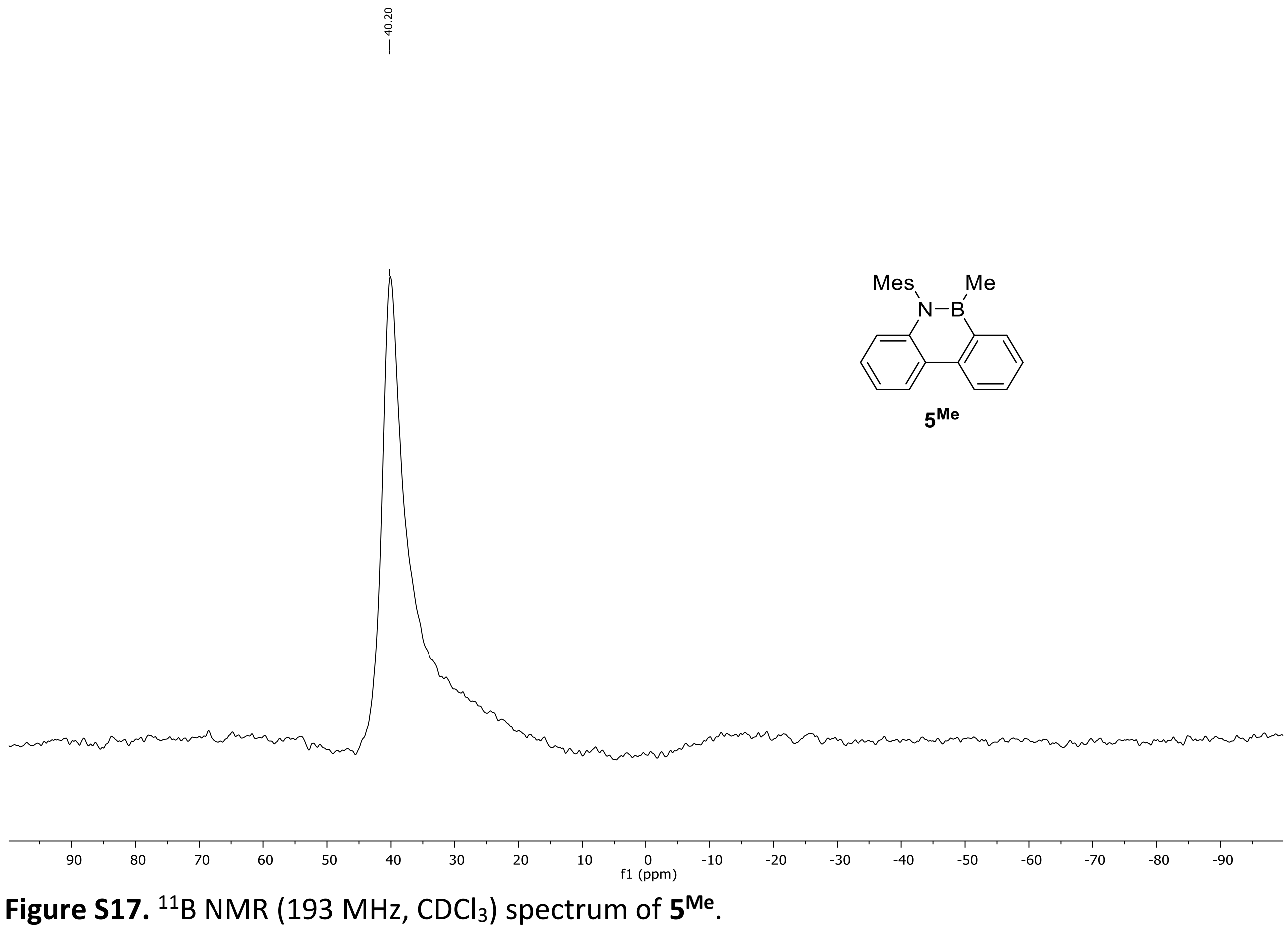

Figure S17. ^{11}B NMR (193 MHz, $CDCl_3$) spectrum of $\mathbf{5^{Me}}$.

Sample Chromatograms

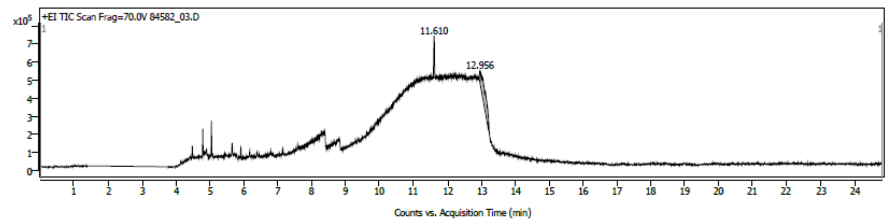

Chromatogram Peaks

Peak	Start	RT	End	Height	Area	Area %	SNR
1	11.580	11.610	11.648	231133	372364	25.48	
2	12.943	12.956	13.219	60921	1461332	100.00	

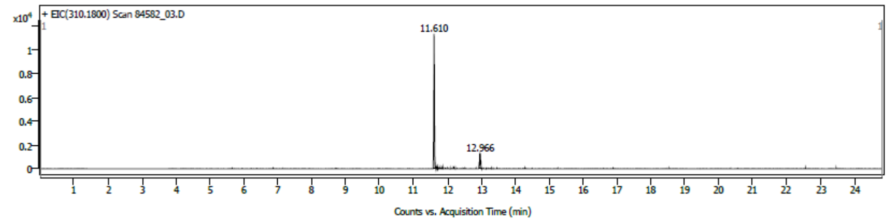

Chromatogram Peaks

Peak	Start	RT	End	Height	Area	Area %	SNR
1	11.583	11.610	11.650	11346	15752	100.00	
2	12.949	12.966	13.003	1306	2450	15.55	

Sample Spectra

+ Scan (rt: 11.580-11.647 min) Peak 1 from + TIC Scan

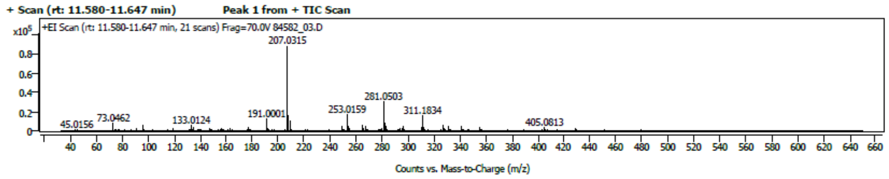

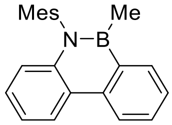

5^{Me}

Figure S18. HRMS (GC/Q-TOF) spectrum of **5^{Me}**.

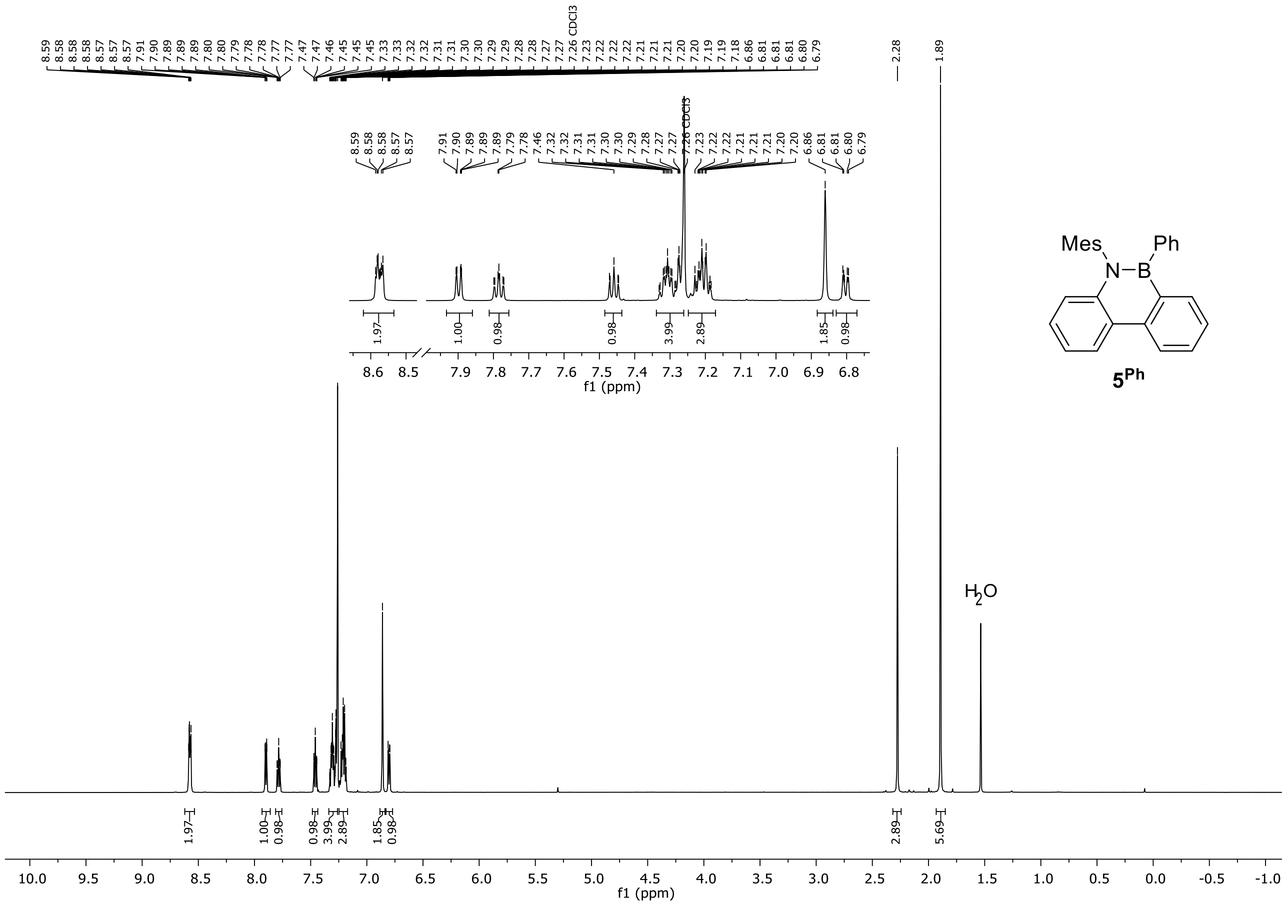

5^{Ph}

Figure S19. ^{1}H NMR (600 MHz, $CDCl_3$) spectrum of **5^{Ph}**.

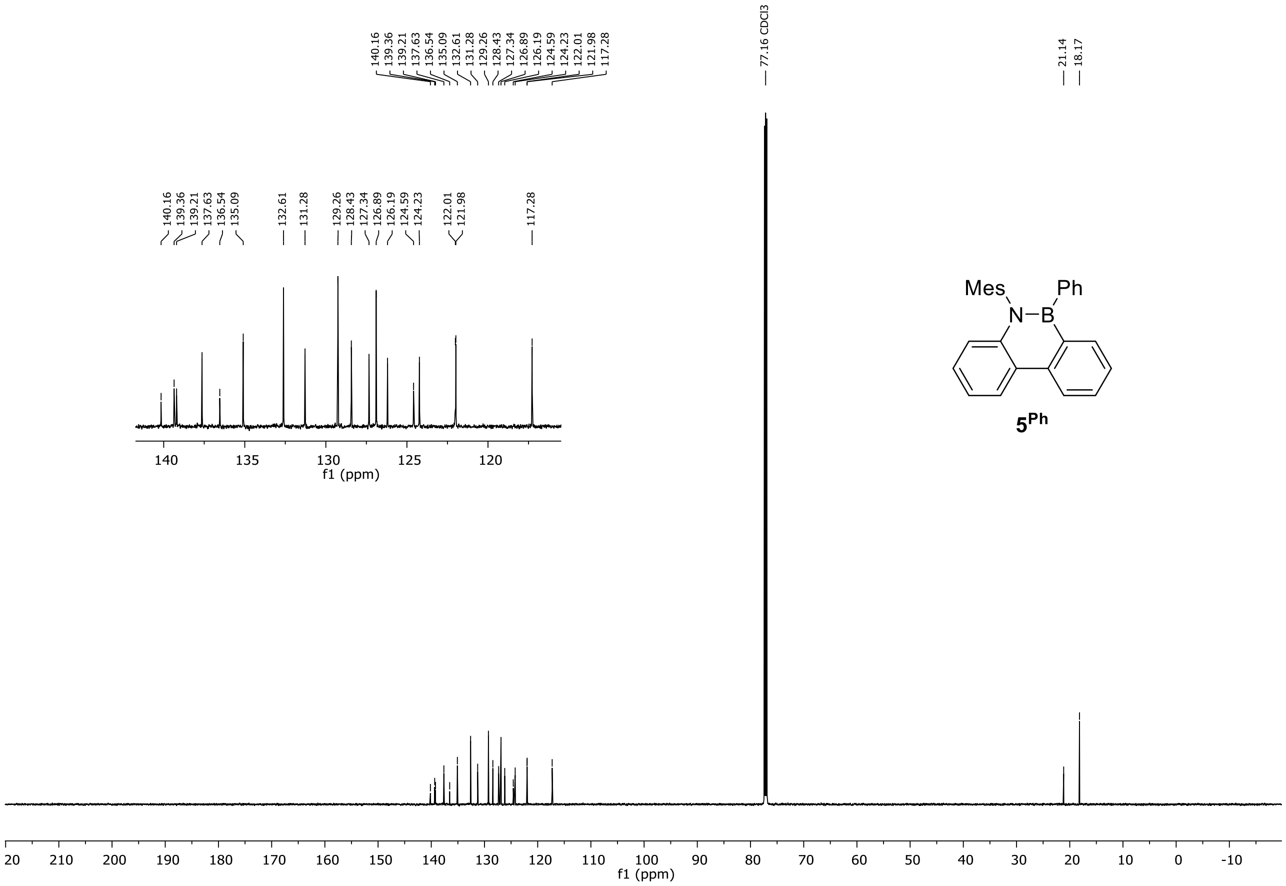

Figure S20. ^{13}C NMR (151 MHz, $CDCl_3$) spectrum of $\mathbf{5^{Ph}}$.

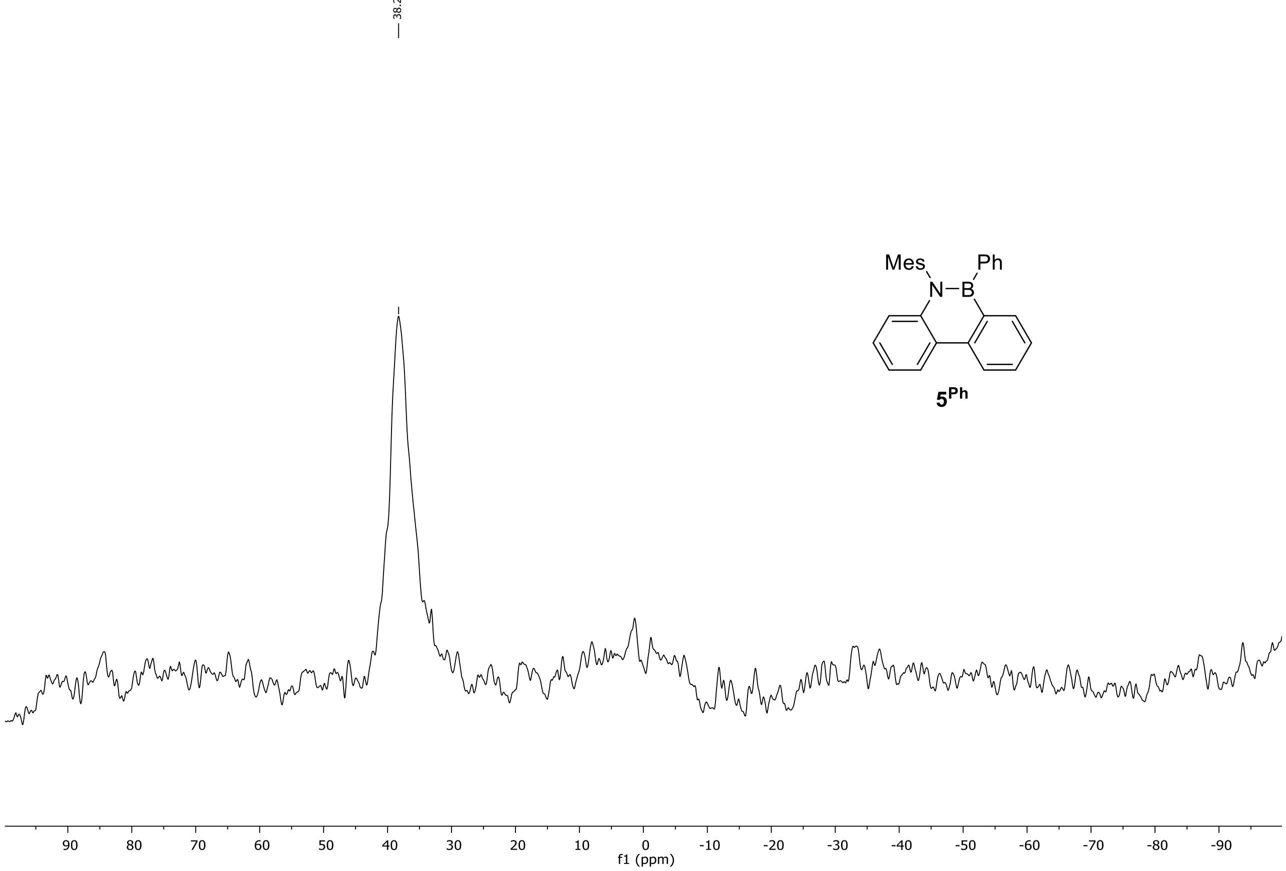

Figure S21. ^{11}B NMR (193 MHz, $CDCl_3$) spectrum of $\mathbf{5^{Ph}}$.

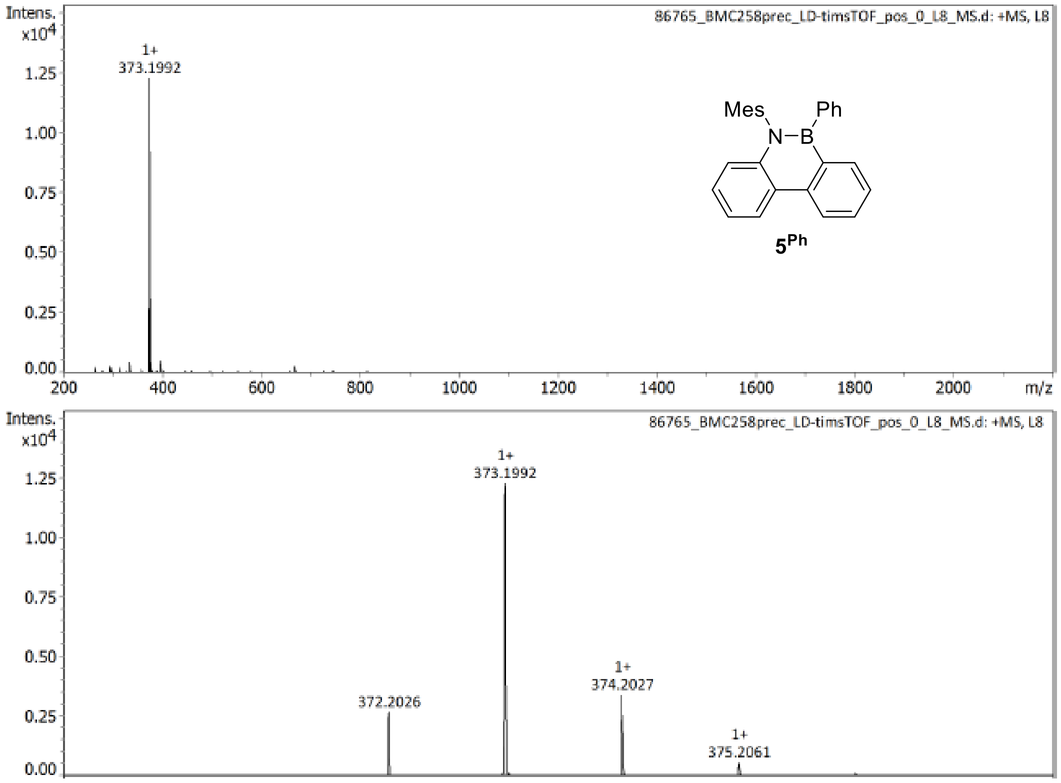

Figure S22. HRMS (LD-timsTOF) spectrum of $\mathbf{5^{Ph}}$.

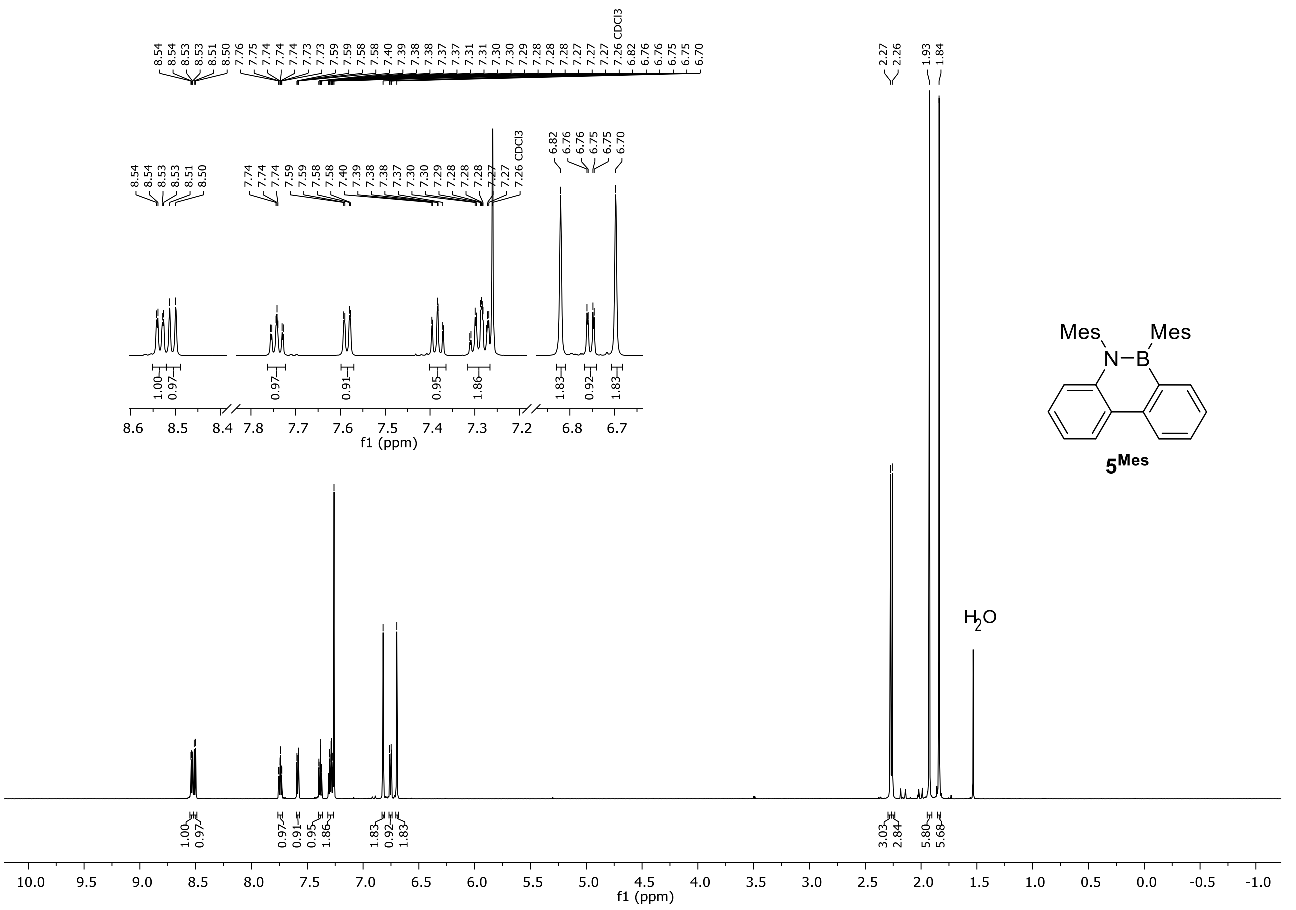

Figure S23. ^{1}H NMR (600 MHz, CDCl$_3$) spectrum of $\mathbf{5^{Mes}}$.

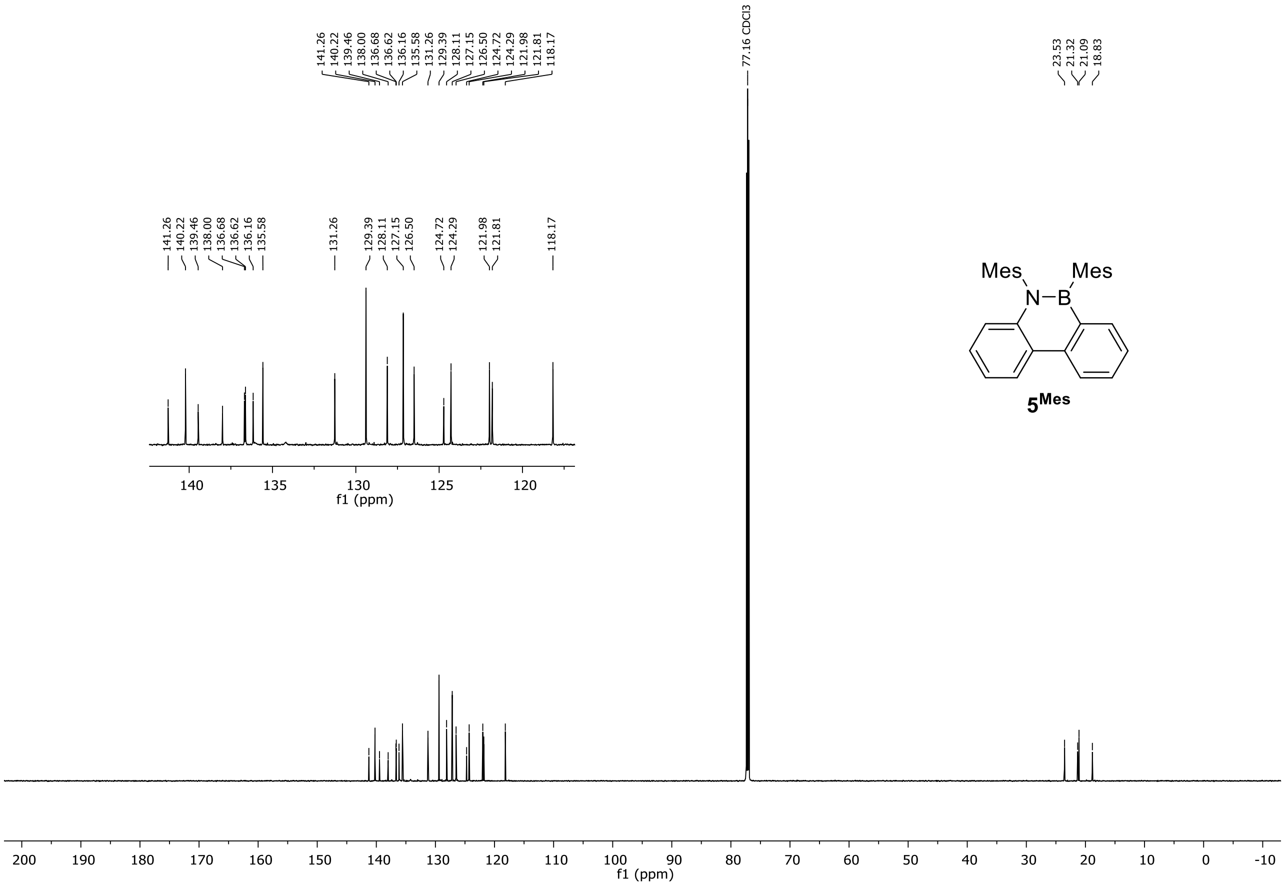

Figure S24. ^{13}C NMR (176 MHz, $CDCl_3$) spectrum of **5^Mes^**.

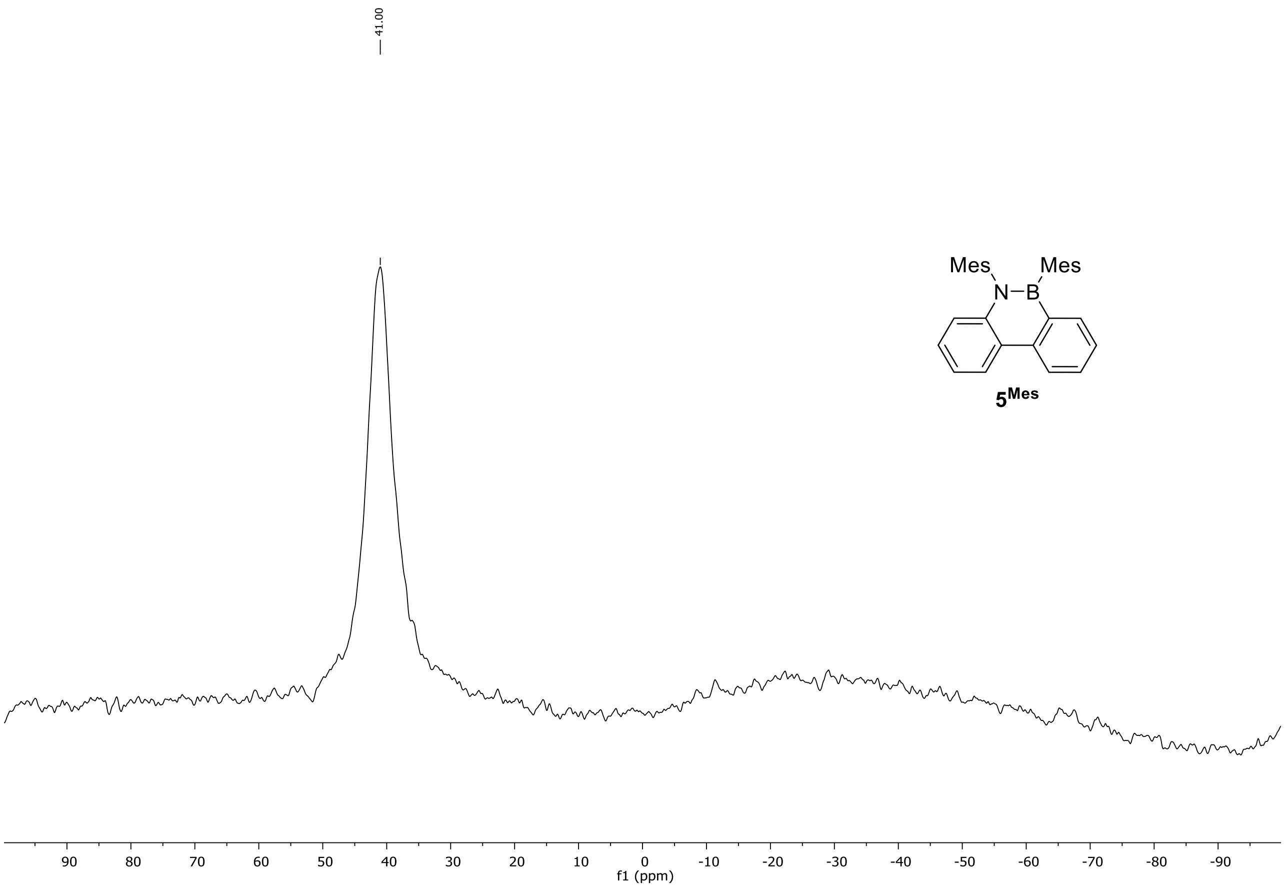

Figure S25. ^{11}B NMR (193 MHz, $CDCl_3$) spectrum of **5^Mes^**.

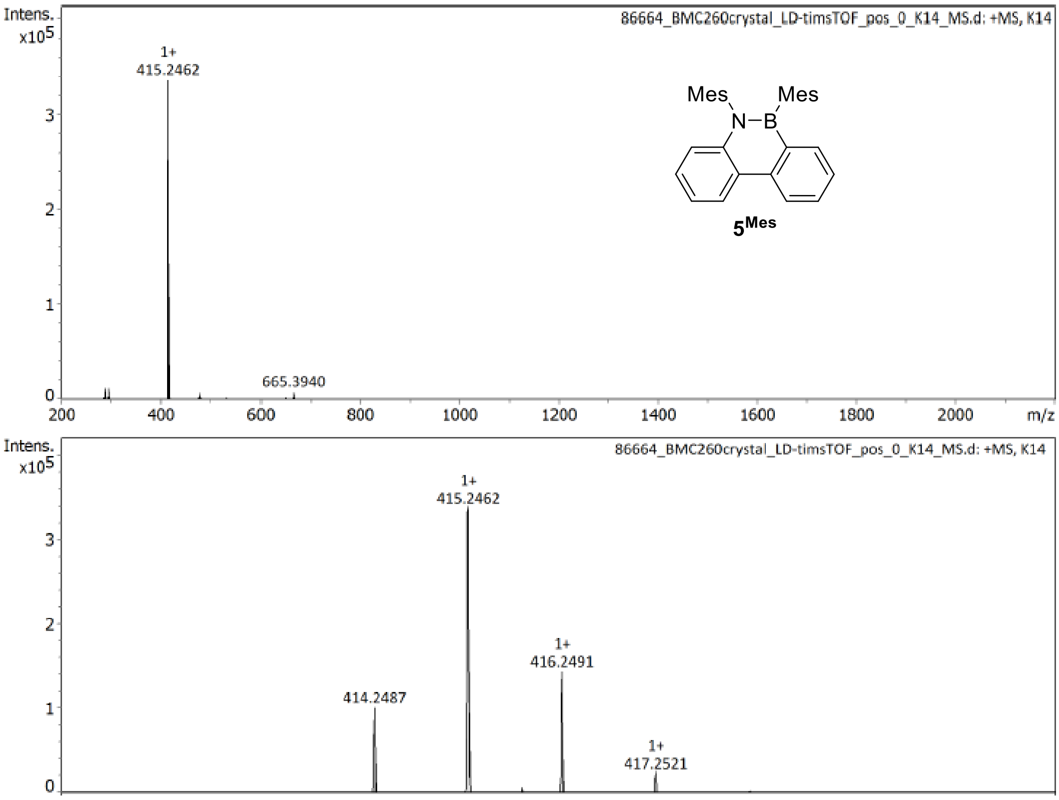

Figure S26. HRMS (LD-timsTOF) spectrum of $\mathbf{5^{Mes}}$.

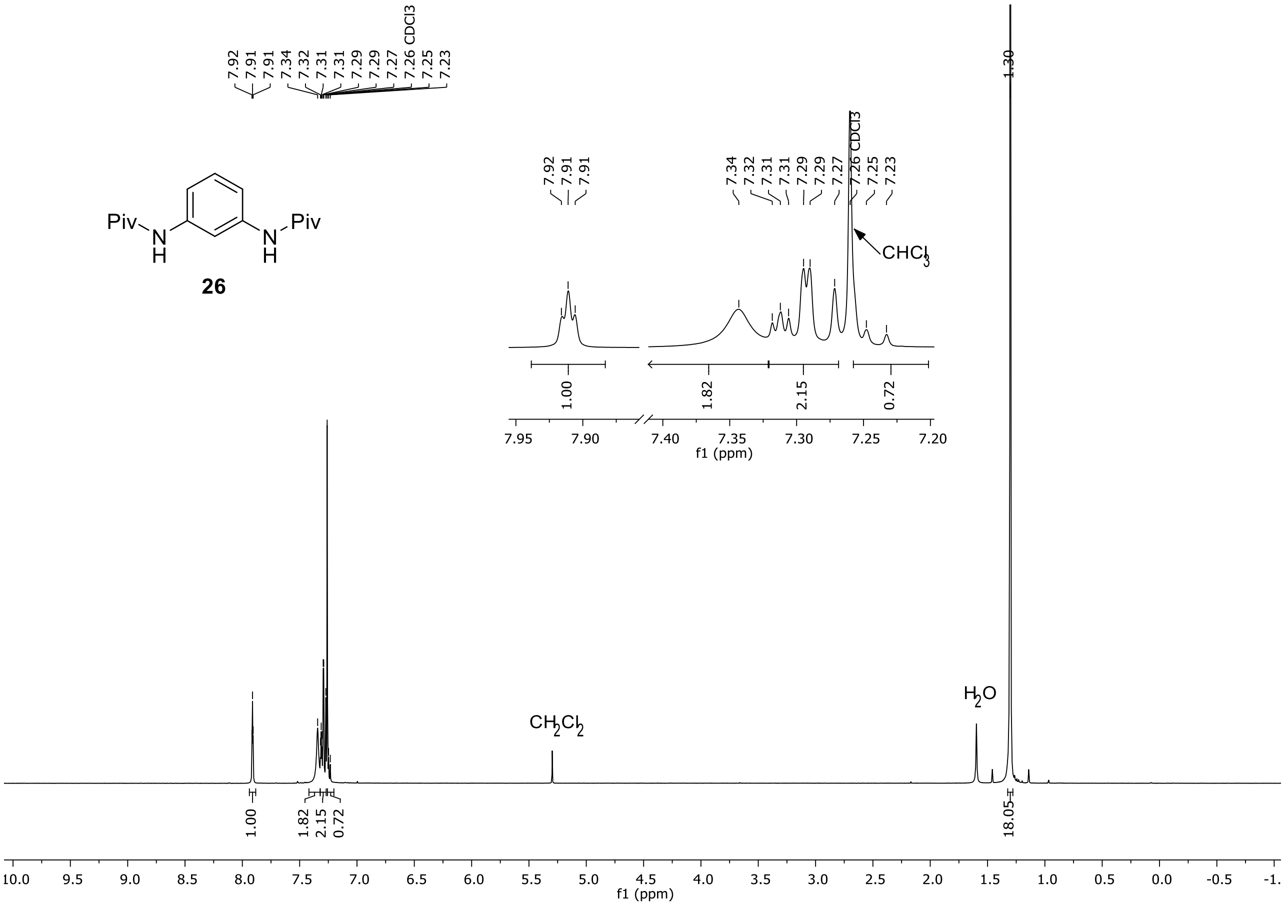

Figure S27. ^{1}H NMR (400 MHz, $CDCl_3$) spectrum of **26**.

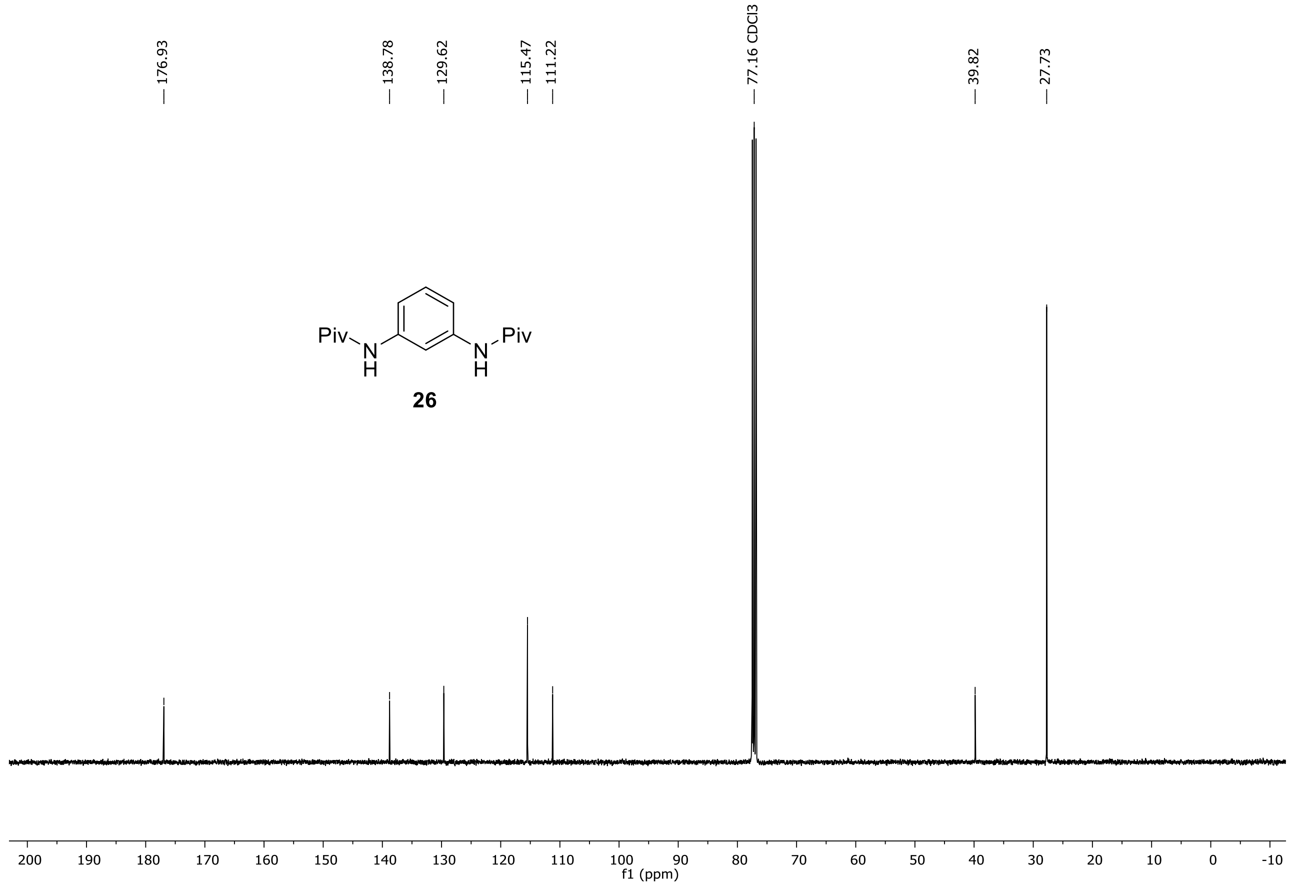

Figure S28. ^{13}C NMR (101 MHz, $CDCl_3$) spectrum of **26**.

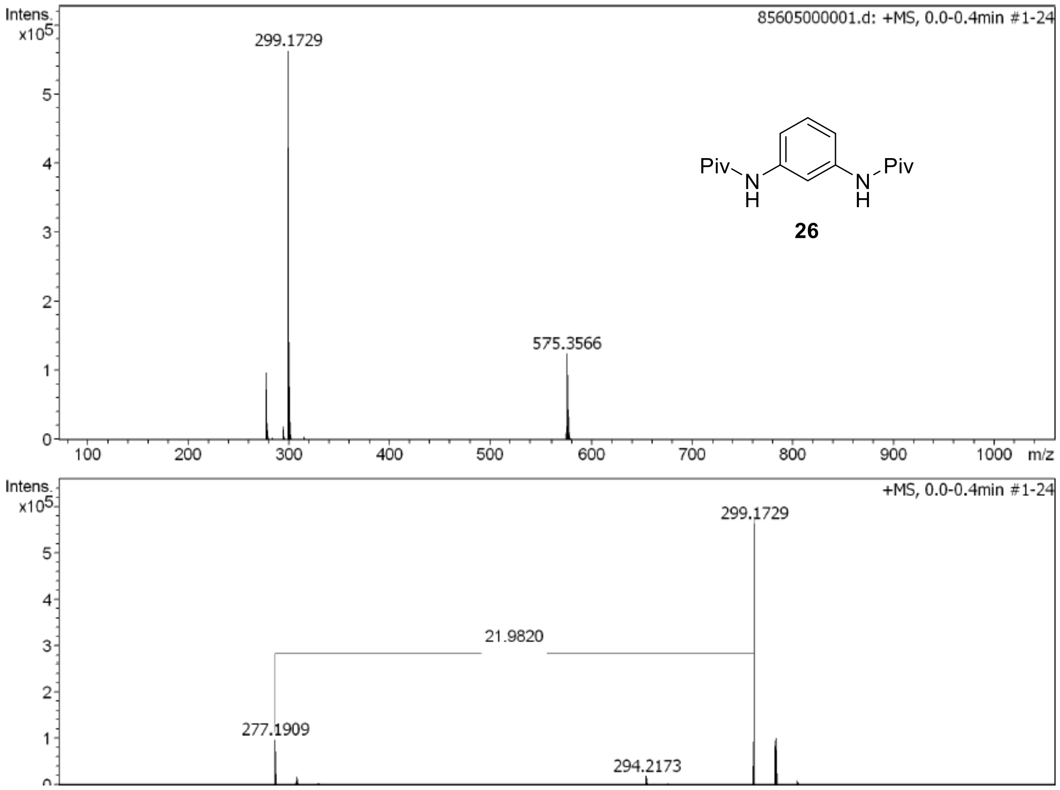

Figure S29. HRMS (ESI) spectrum of **26**.

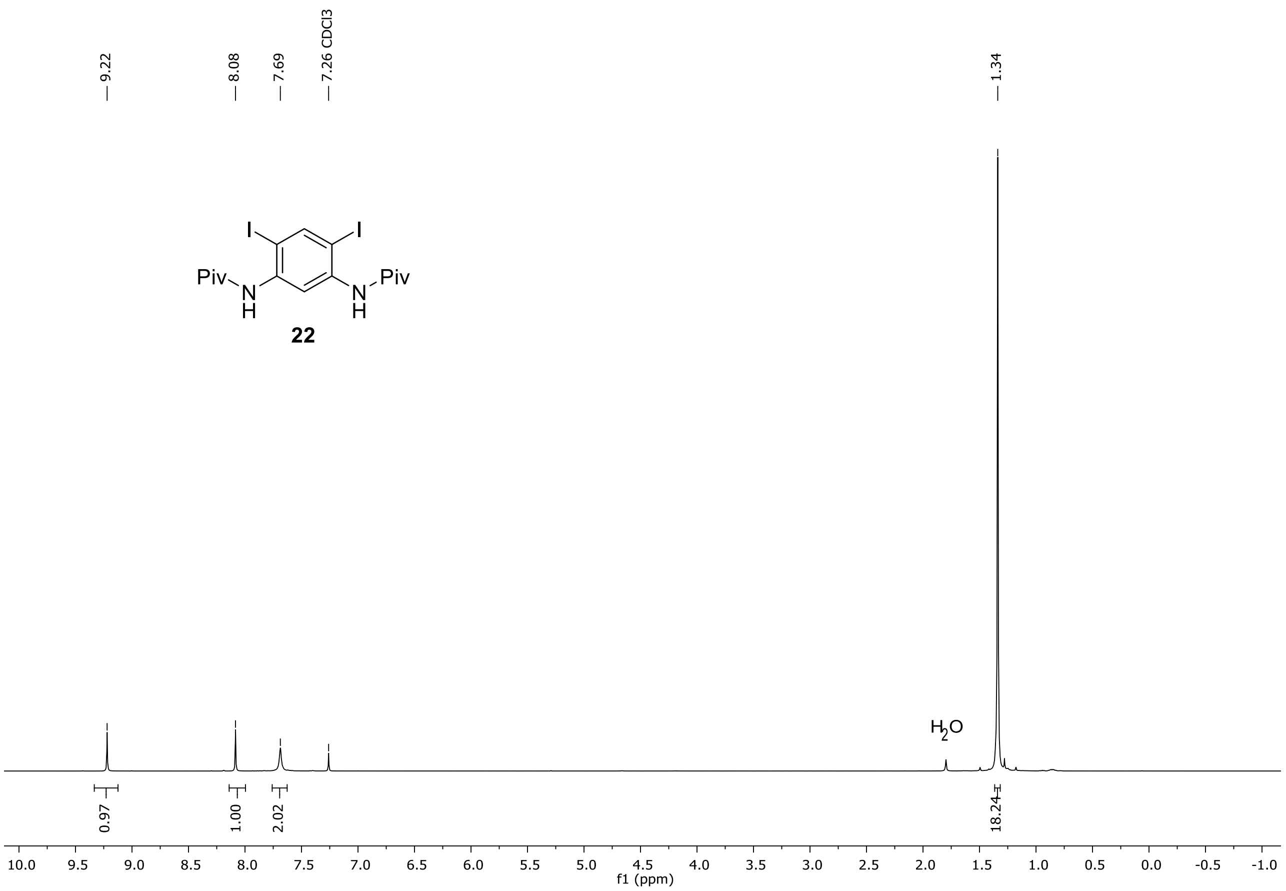

Figure S30. ^{1}H NMR (600 MHz, $CDCl_3$) spectrum of **22**.

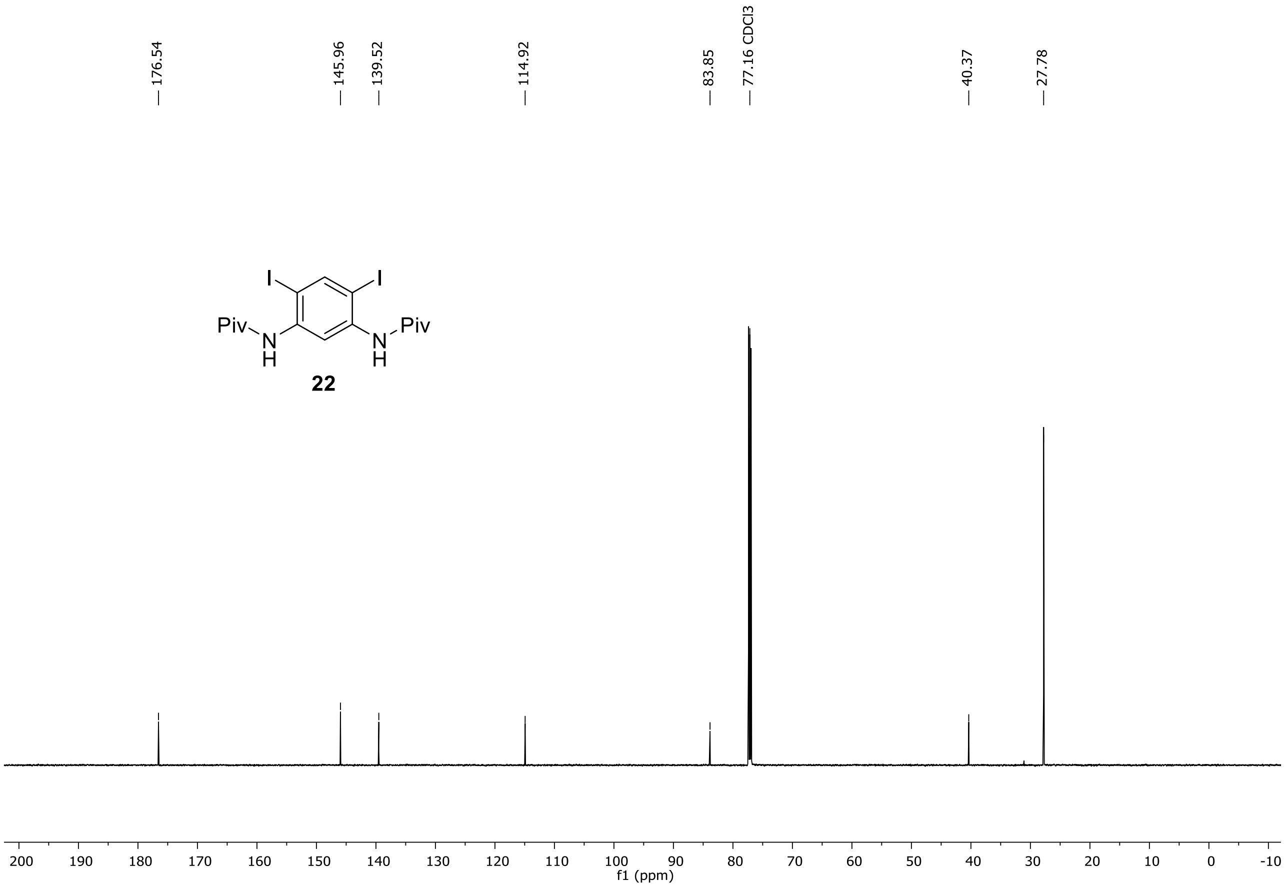

Figure S31. ^{13}C NMR (151 MHz, $CDCl_3$) spectrum of **22**.

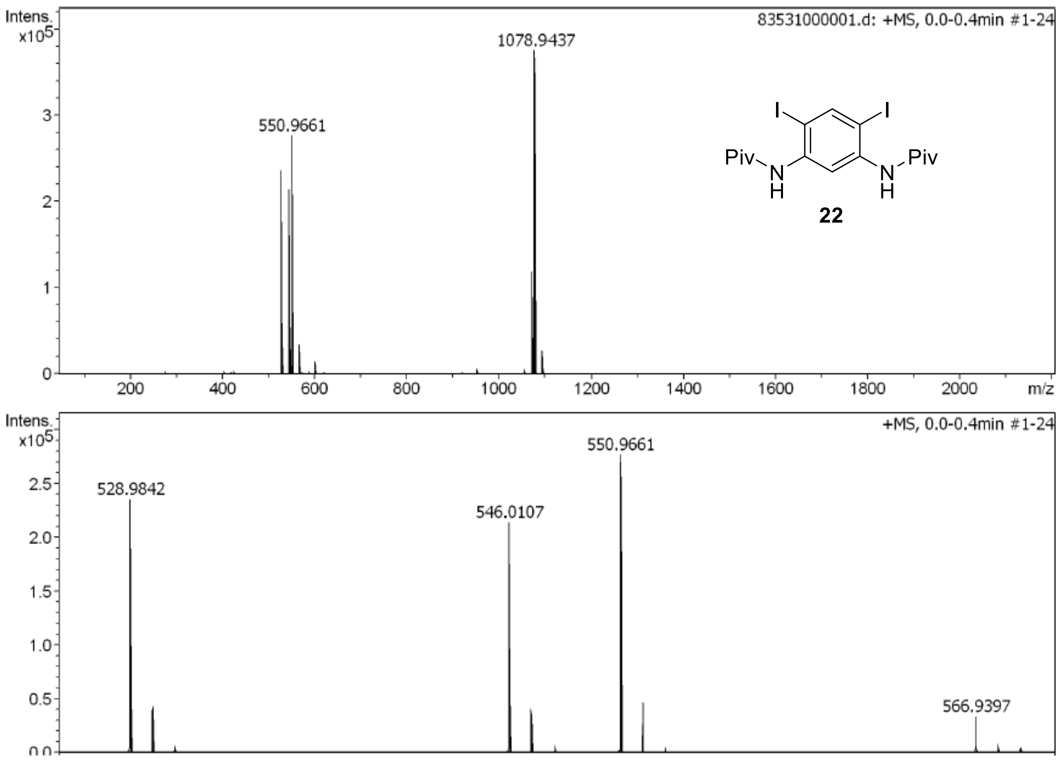

Figure S32. HRMS (ESI) spectrum of **22**.

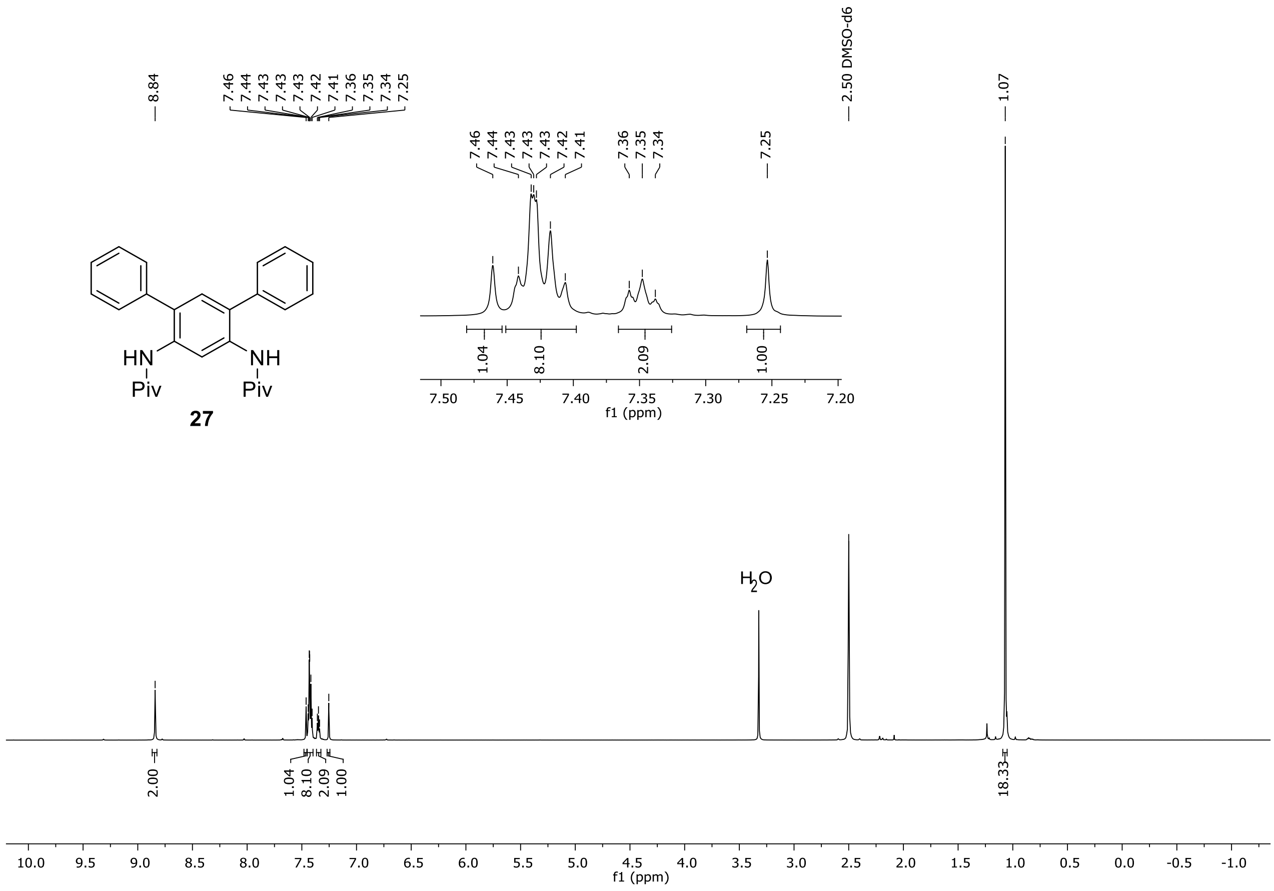

Figure S33. ^{1}H NMR (700 MHz, DMSO-d_6) spectrum of **27**.

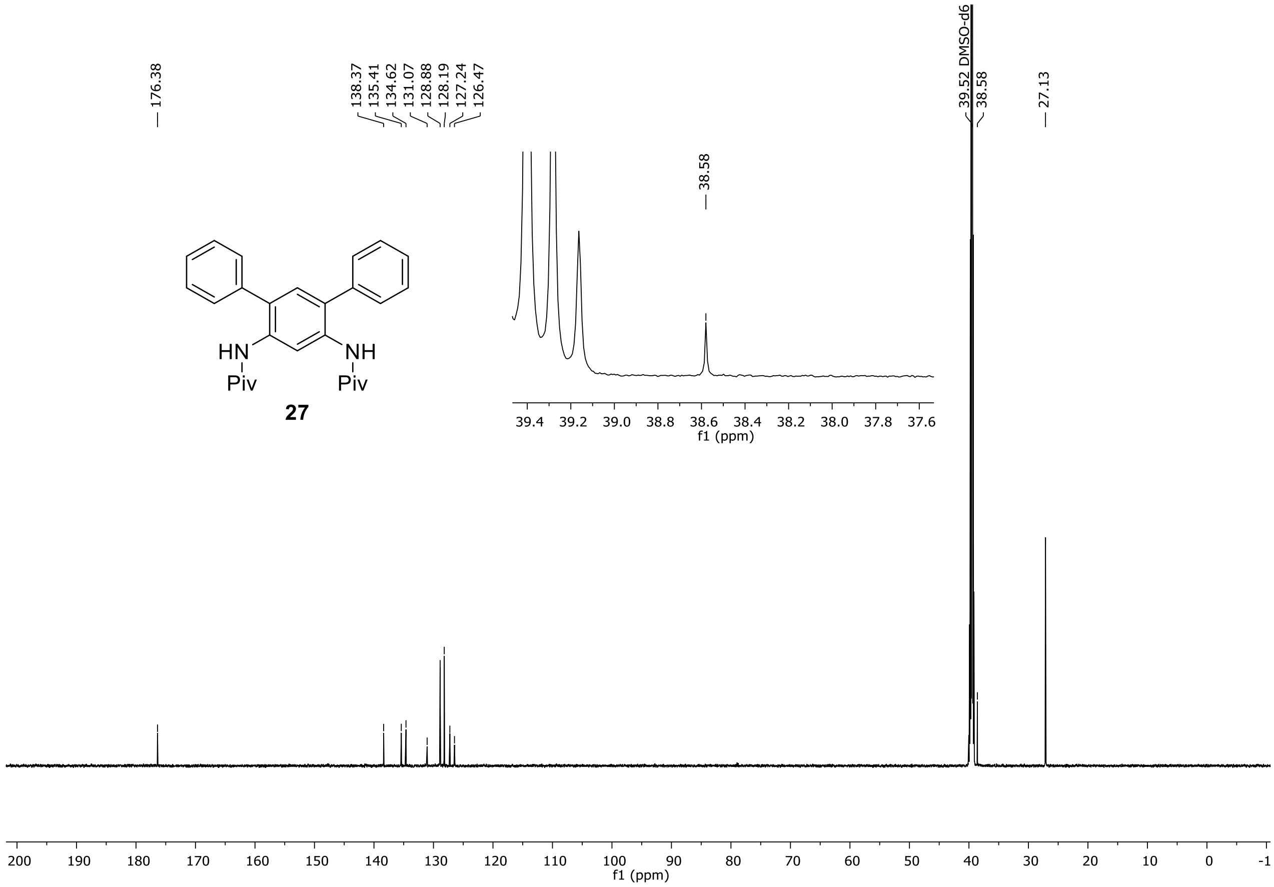

Figure S34. ^{13}C NMR (176 MHz, DMSO-d_6) spectrum of **27**.

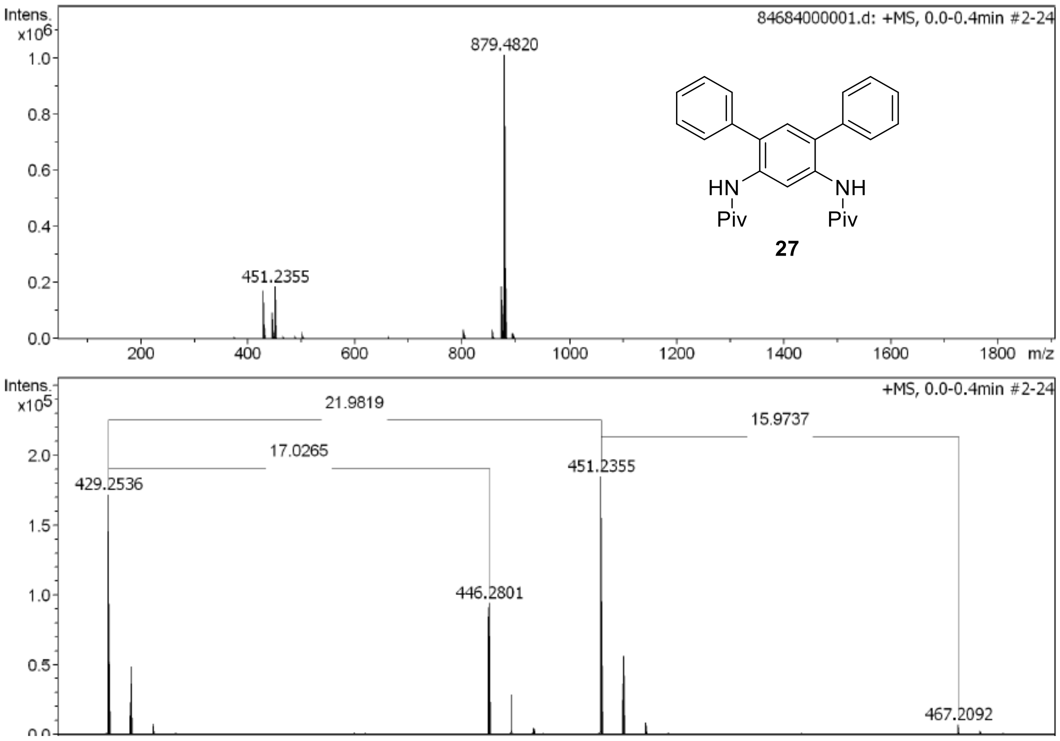

Figure S35. HRMS (ESI) spectrum of **27**.

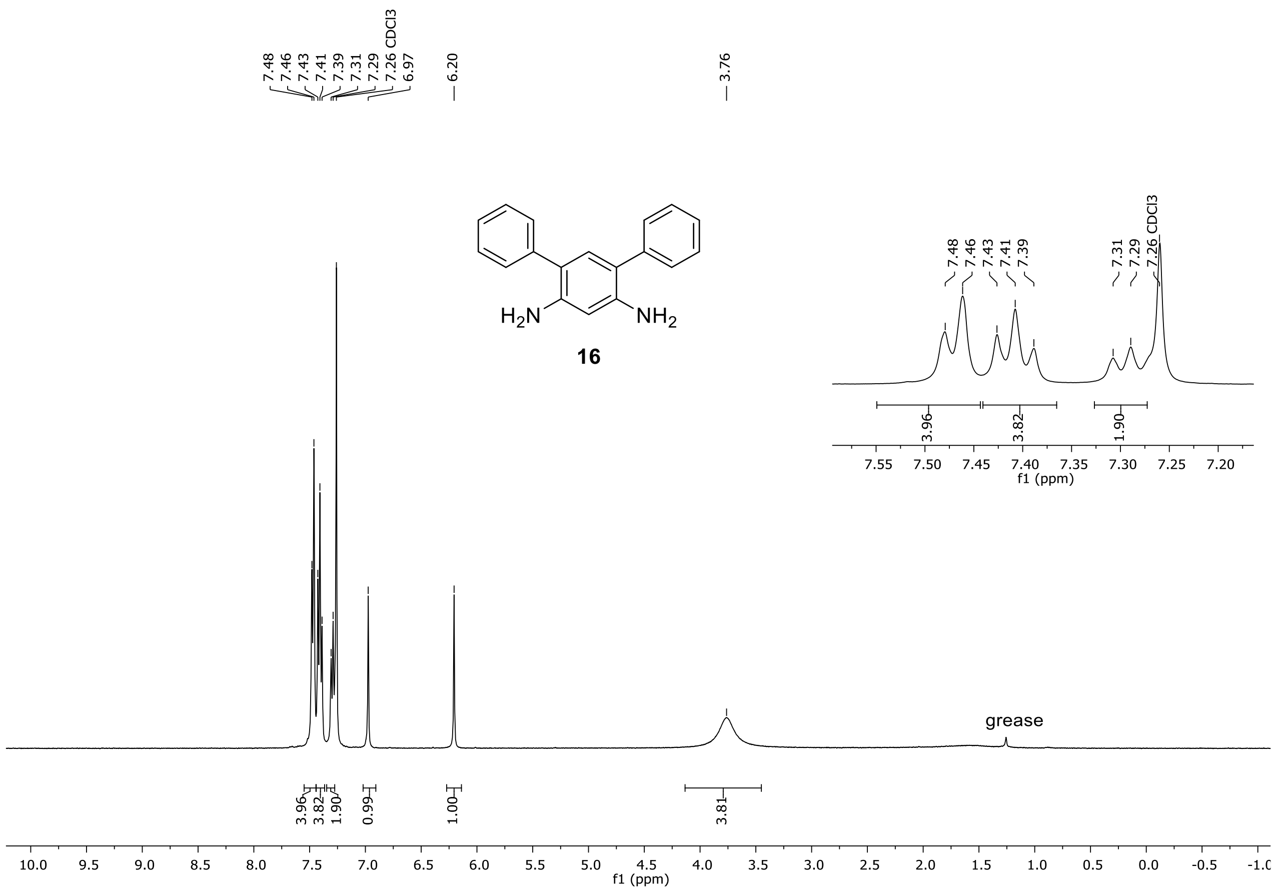

Figure S36. ^{1}H NMR (400 MHz, $CDCl_3$) spectrum of **16**.

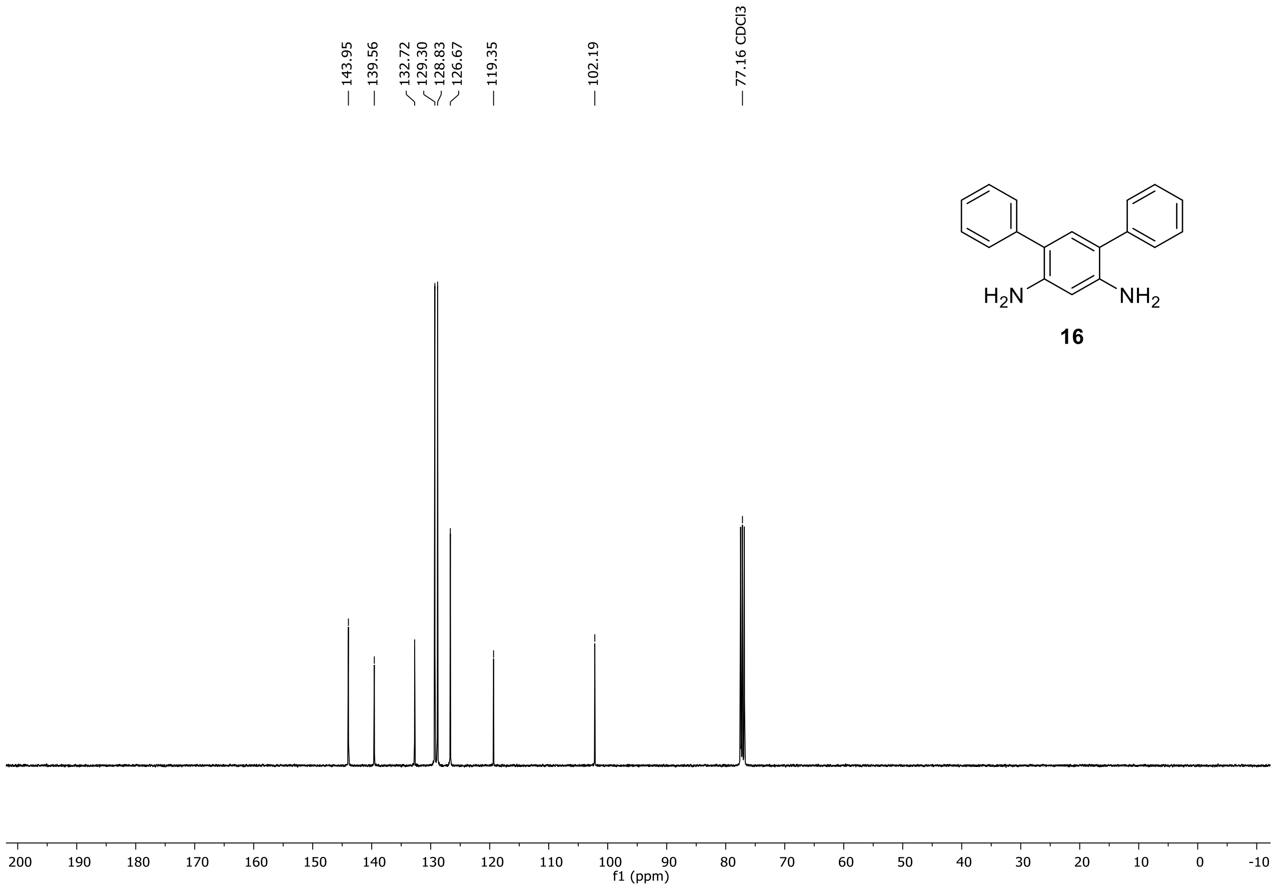

Figure S37. ^{13}C NMR (101 MHz, $CDCl_3$) spectrum of **16**.

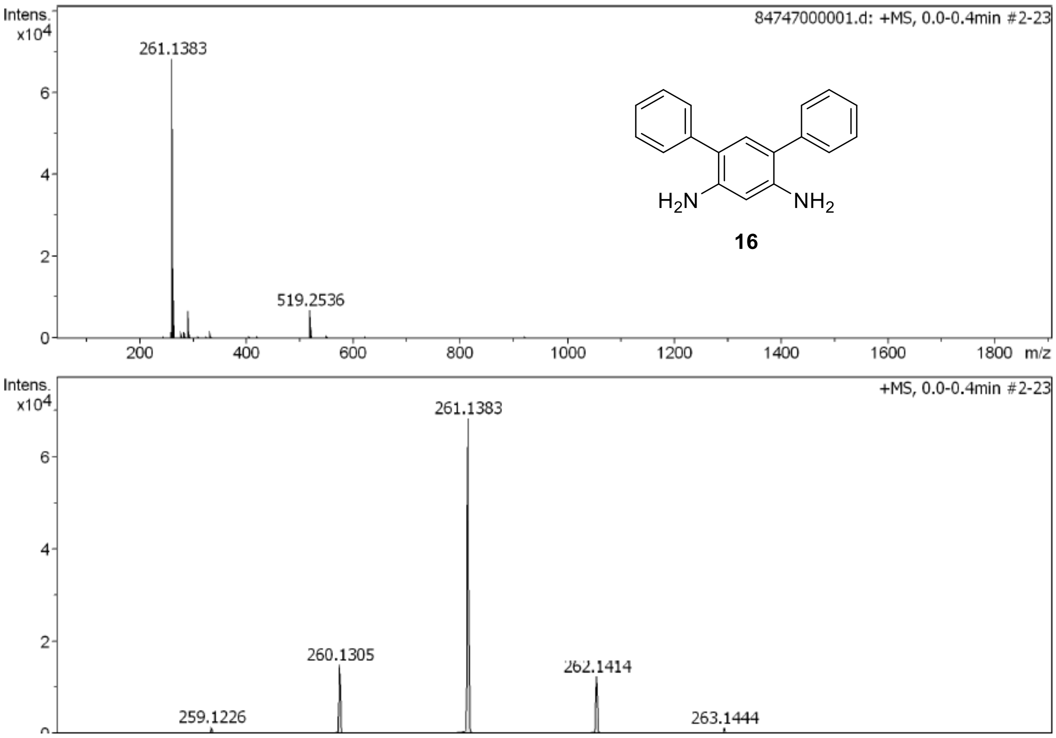

Figure S38. HRMS (ESI) spectrum of **16**.

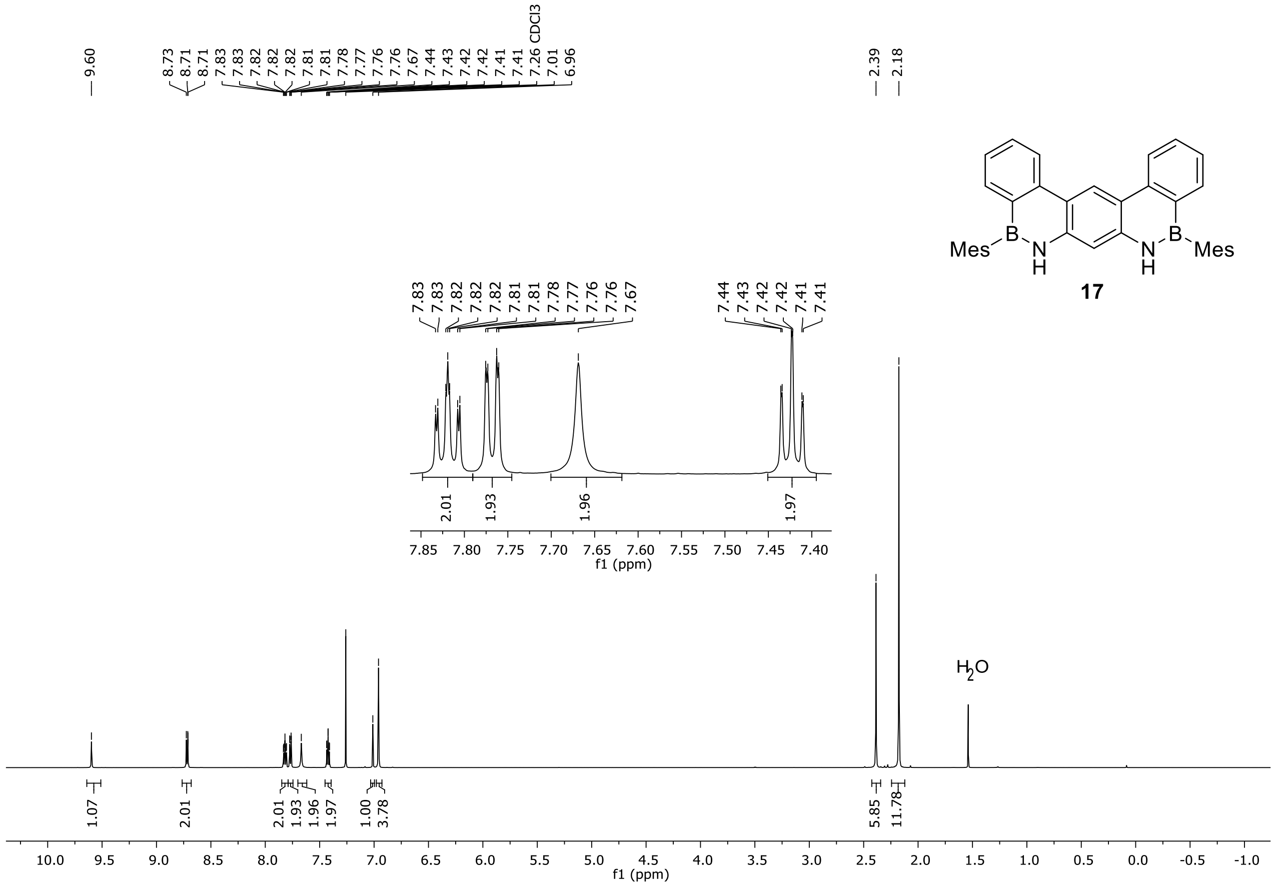

Figure S39. ^{1}H NMR (600 MHz, $CDCl_3$) spectrum of **17**.

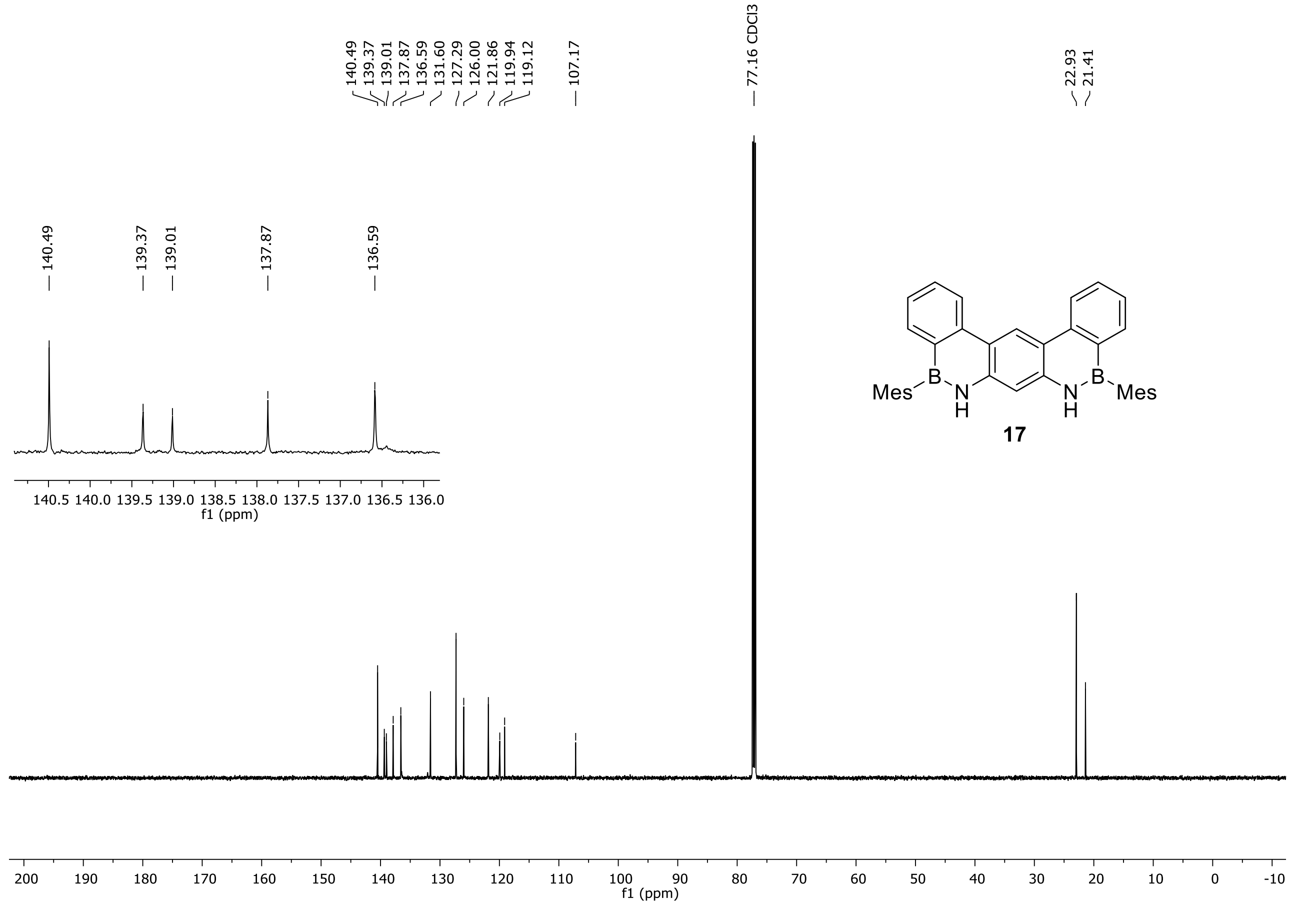

Figure S40. ^{13}C NMR (151 MHz, $CDCl_3$) spectrum of **17**.

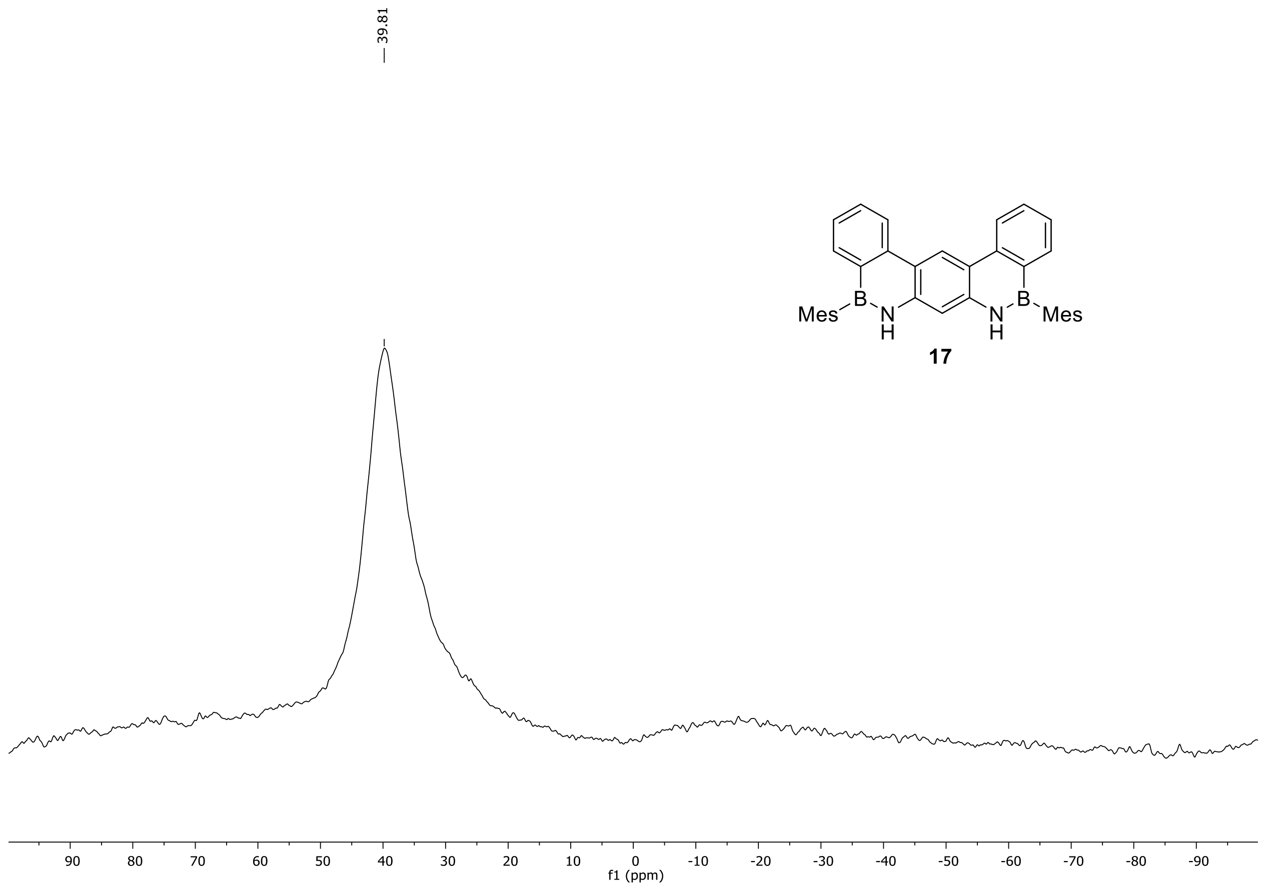

Figure S41. ^{11}B NMR (193 MHz, $CDCl_3$) spectrum of **17**.

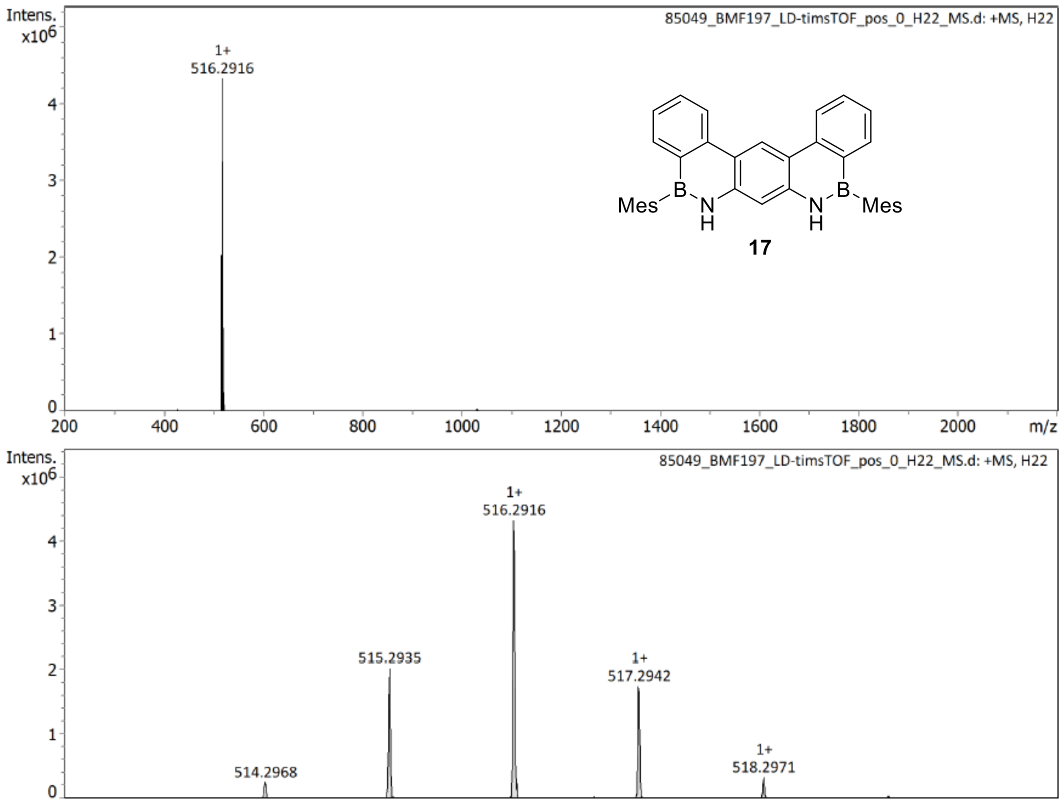

Figure S42. HRMS (LD-timsTOF) spectrum of **17**.

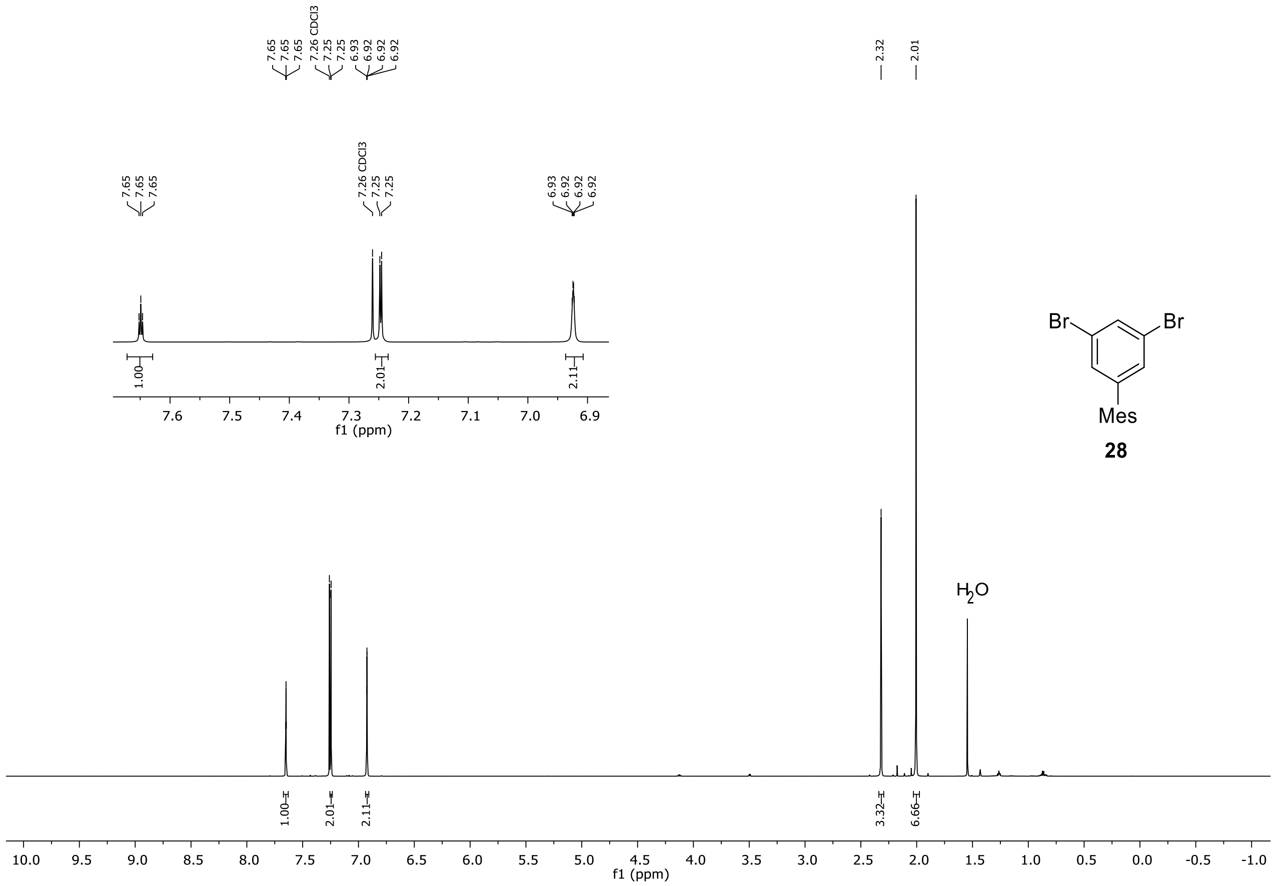

Figure S43. ^{1}H NMR (600 MHz, $CDCl_3$) spectrum of **28**.

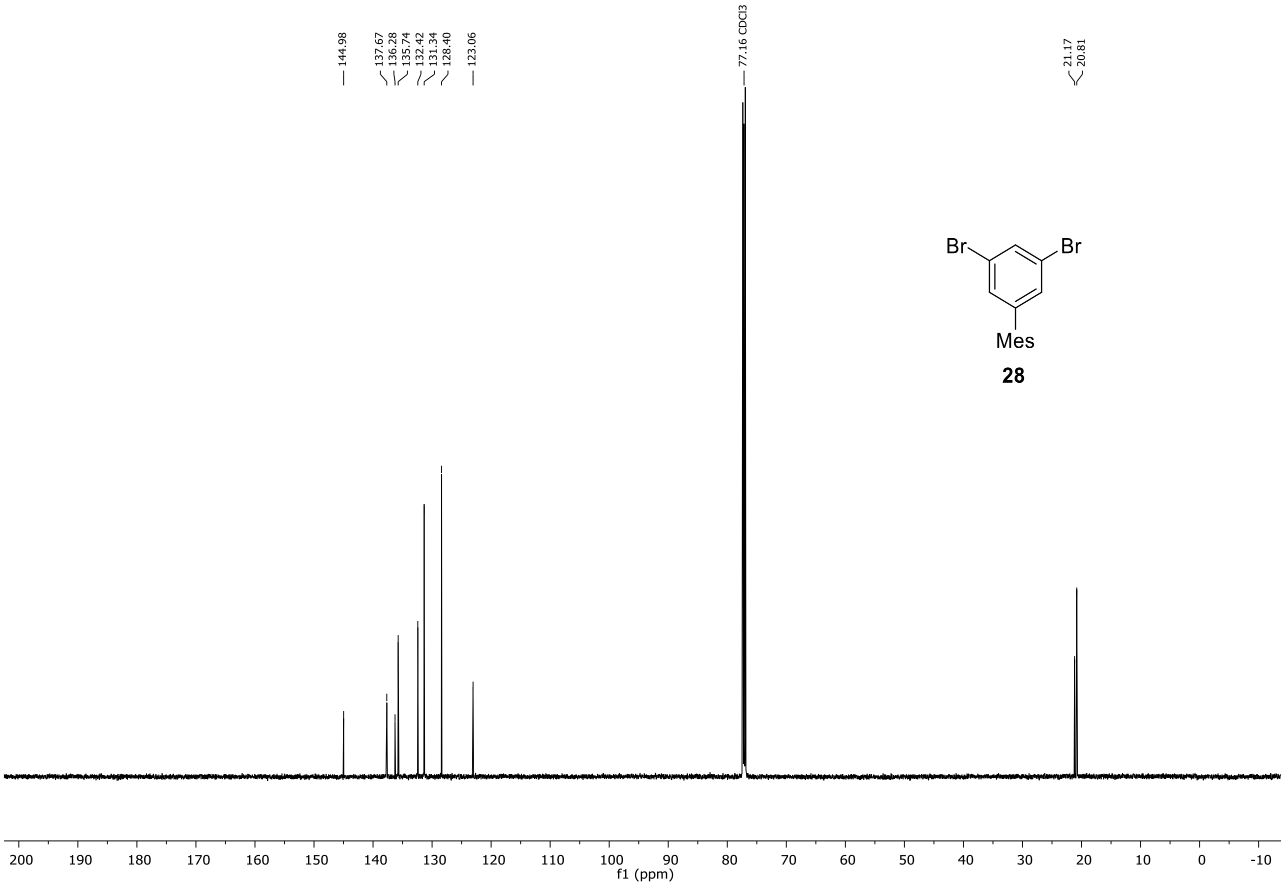

Figure S44. ^{13}C NMR (151 MHz, $CDCl_3$) spectrum of **28**.

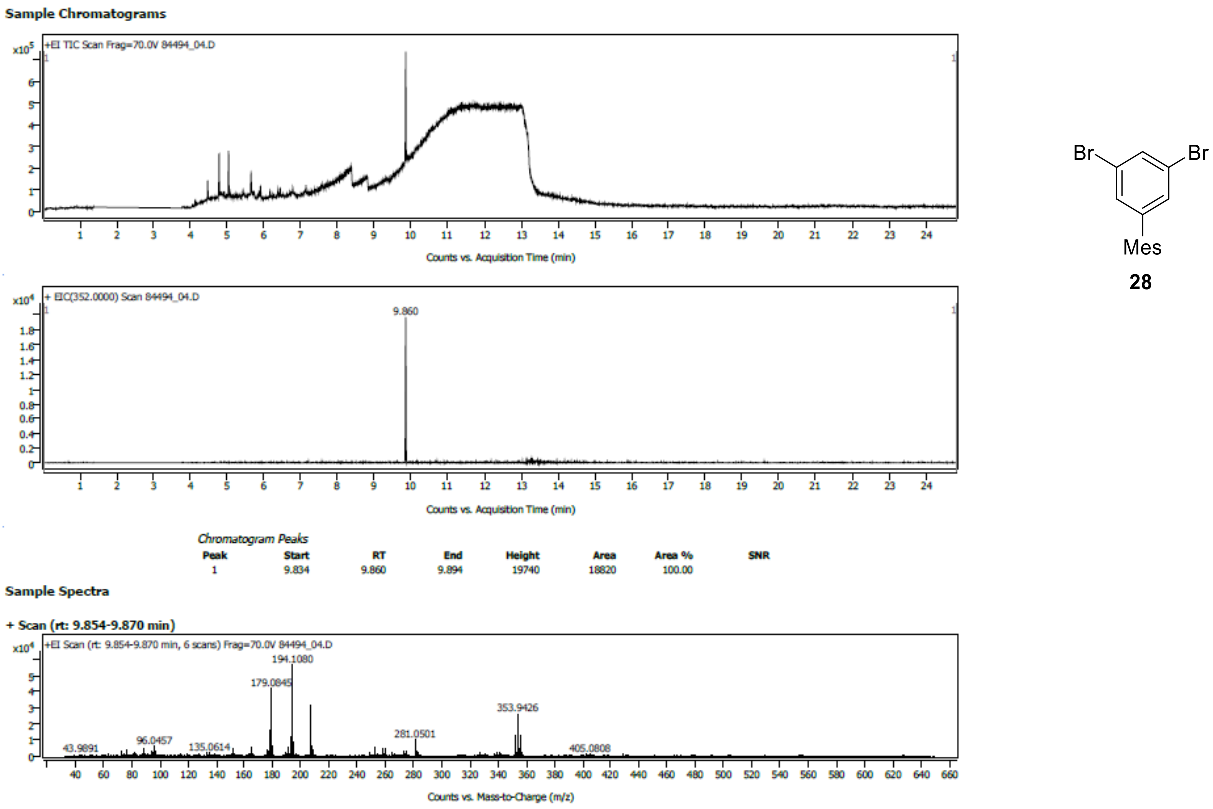

Figure S45. HRMS (GC/Q-TOF) spectrum of **28**.

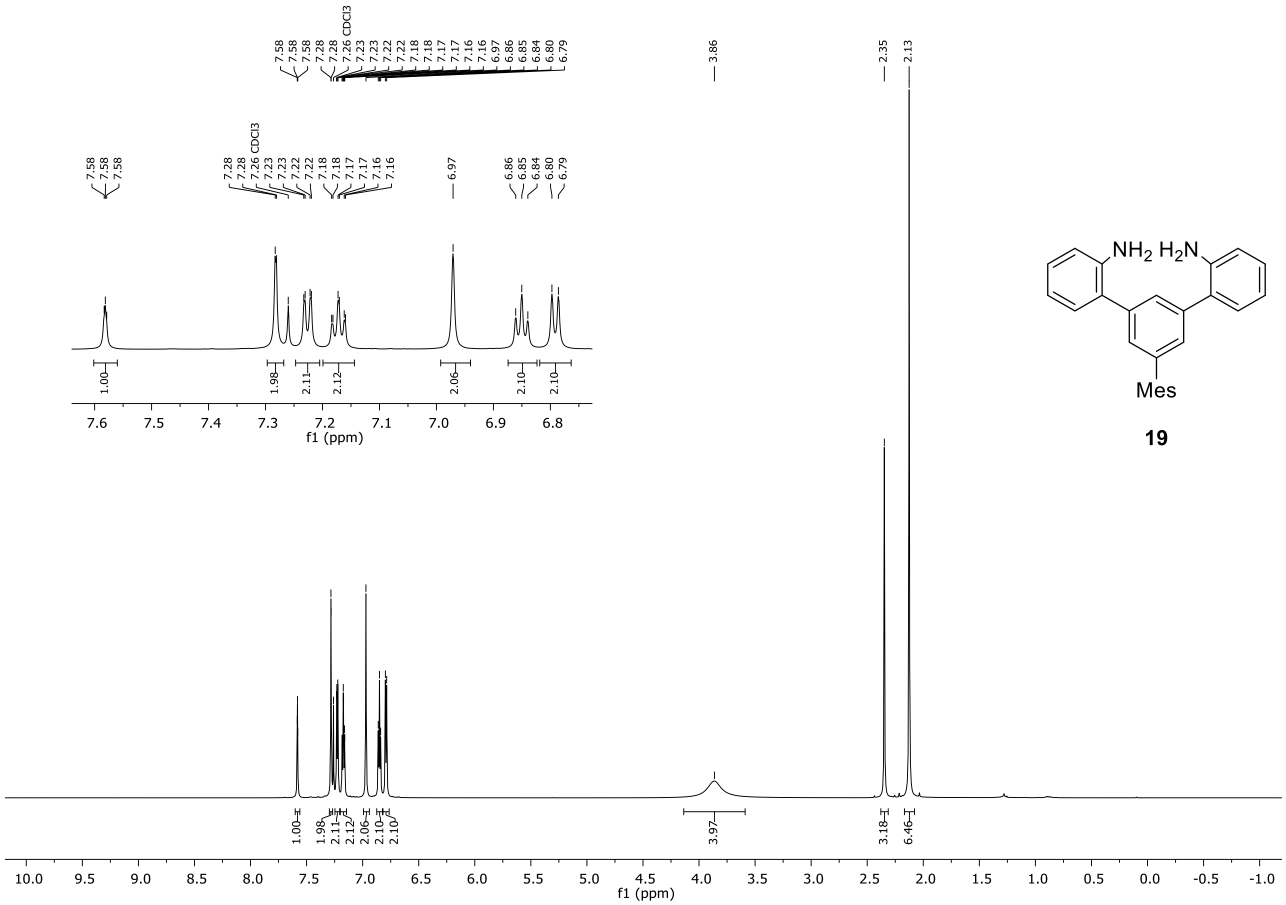

Figure S46. ^{1}H NMR (700 MHz, $CDCl_3$) spectrum of **19**.

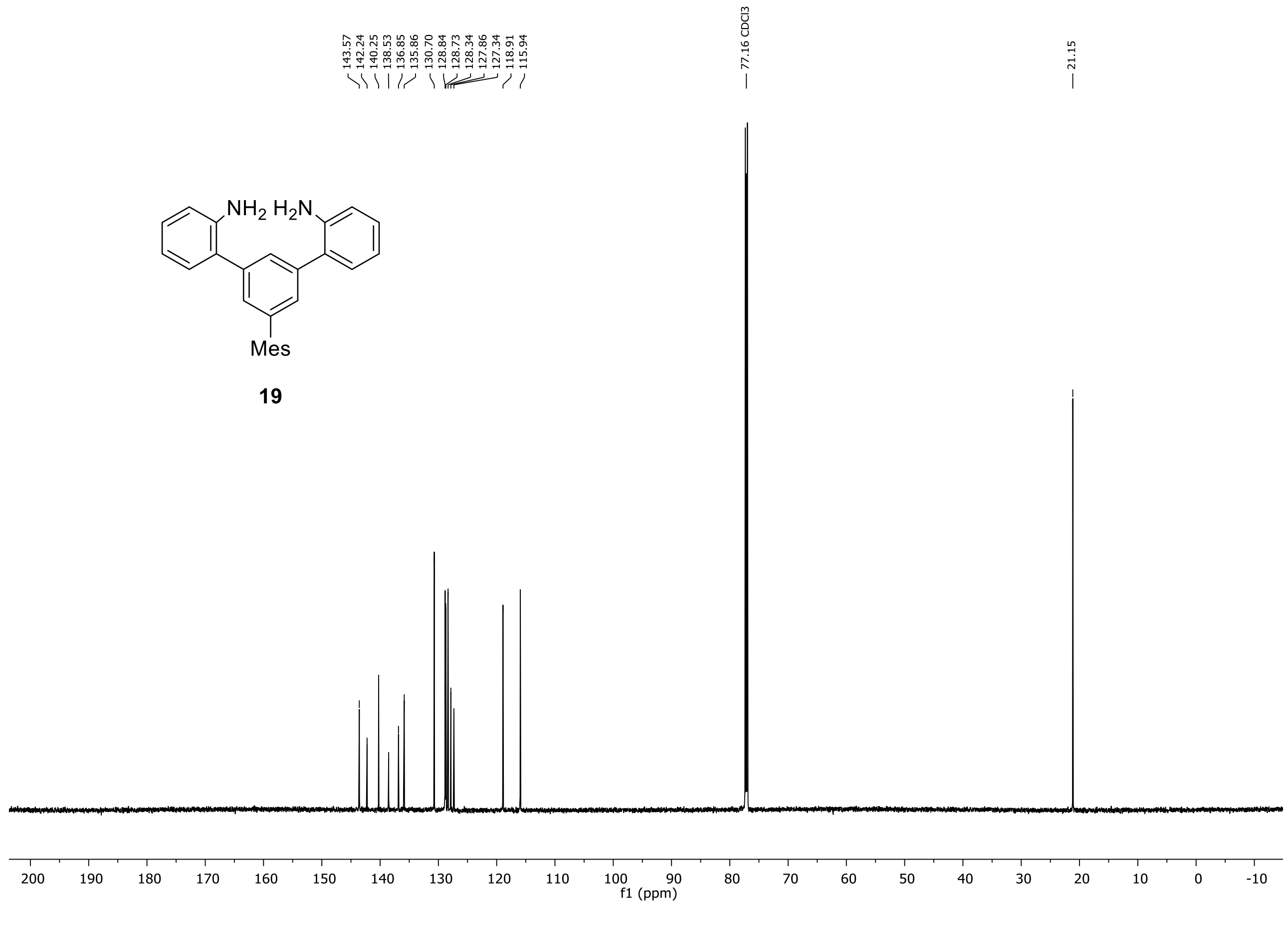

Figure S47. ^{13}C NMR (151 MHz, $CDCl_3$) spectrum of **19**.

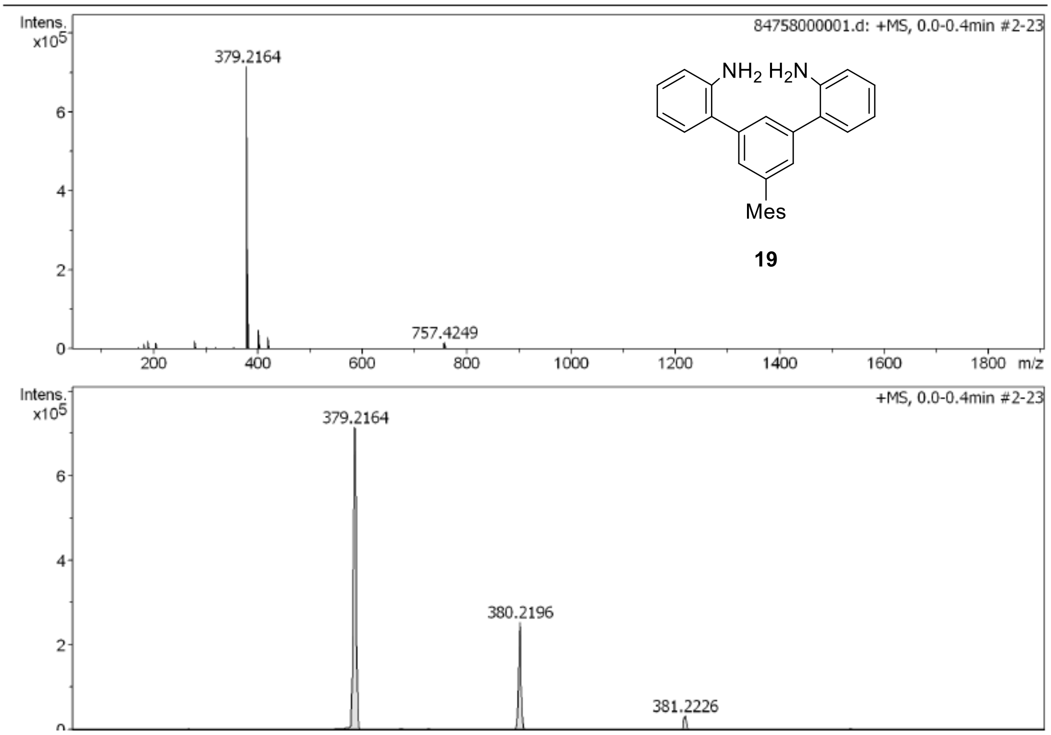

Figure S48. HRMS (ESI) spectrum of **19**.

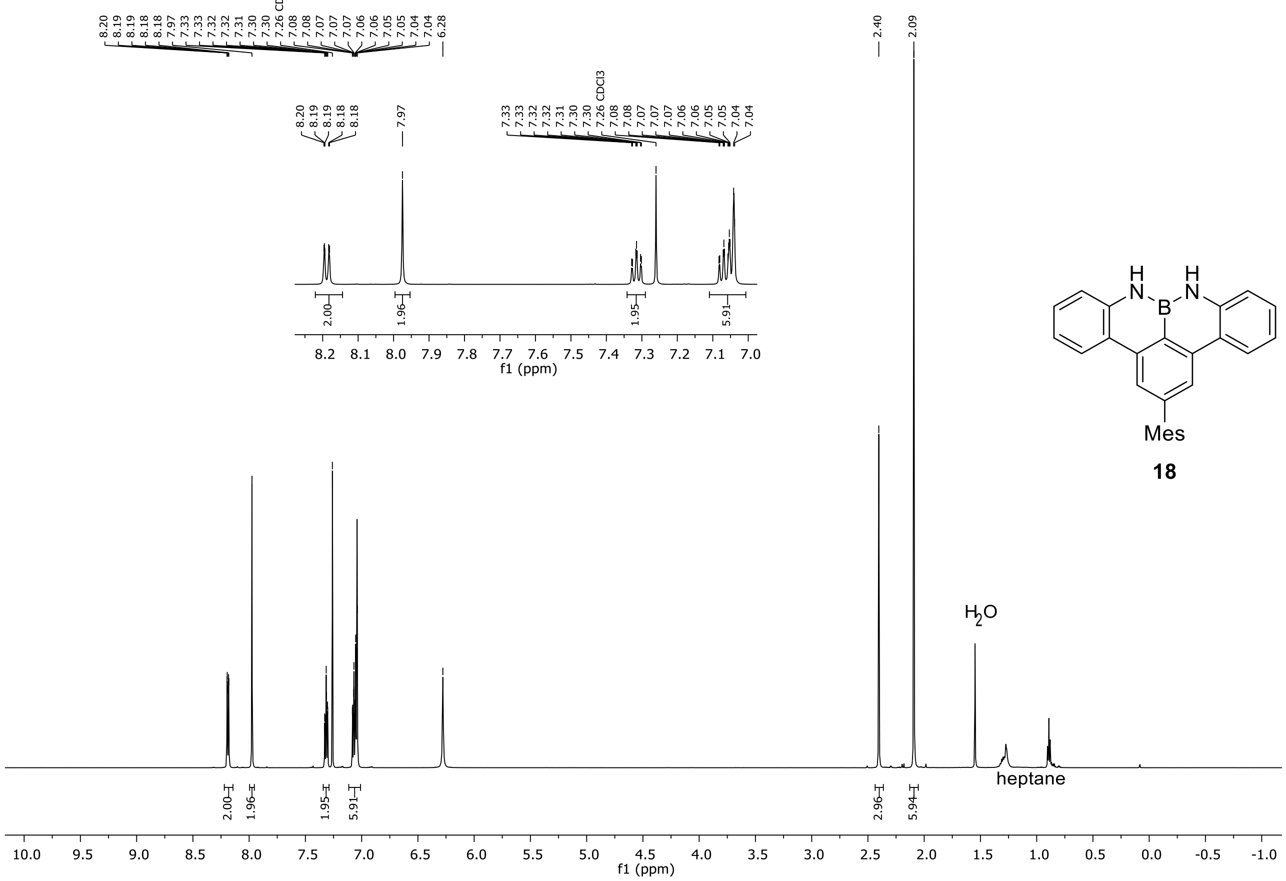

Figure S49. ^{1}H NMR (600 MHz, $CDCl_3$) spectrum of **18**.

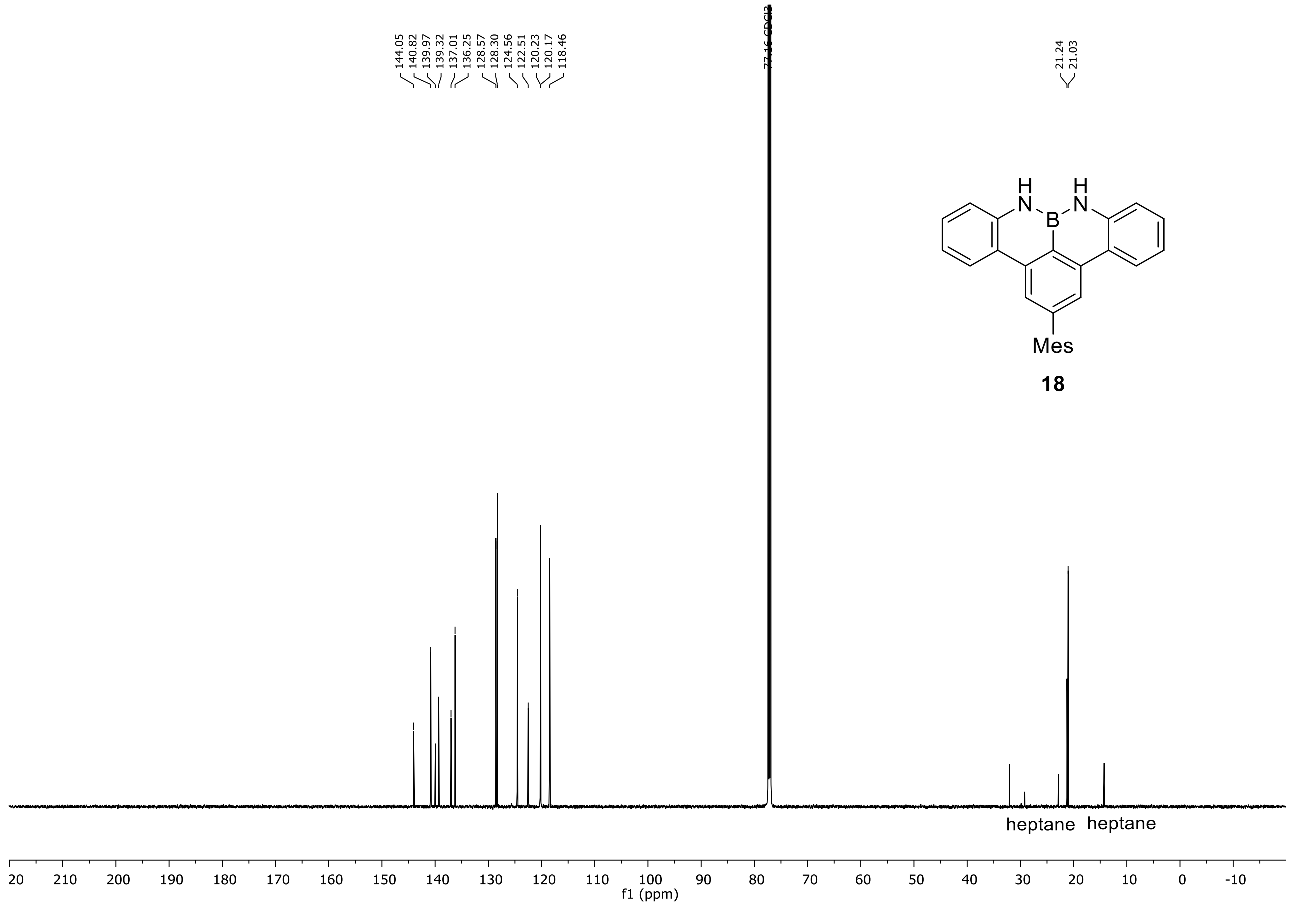

Figure S50. ^{13}C NMR (151 MHz, $CDCl_3$) spectrum of **18**.

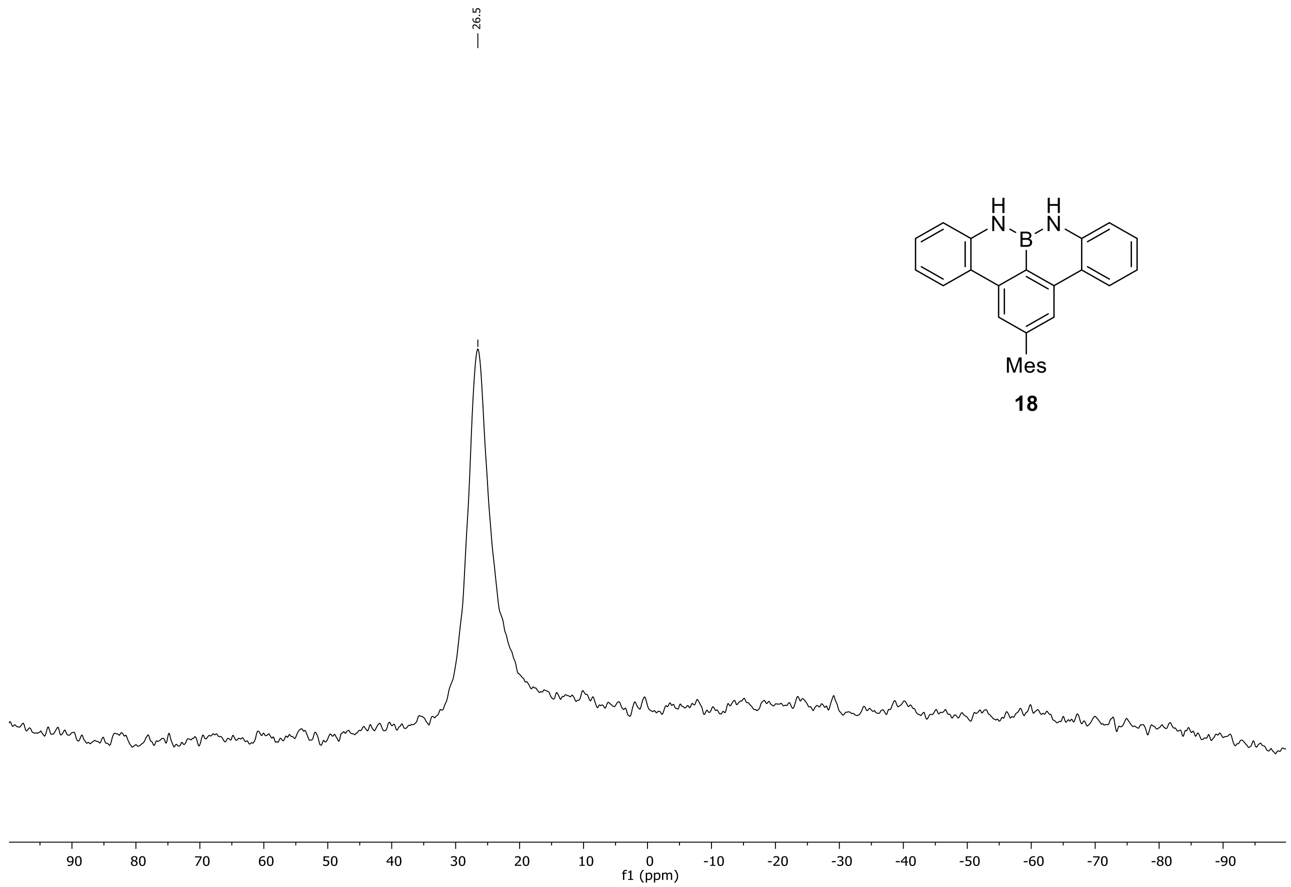

Figure S51. ^{11}B NMR (193 MHz, $CDCl_3$) spectrum of **18**.

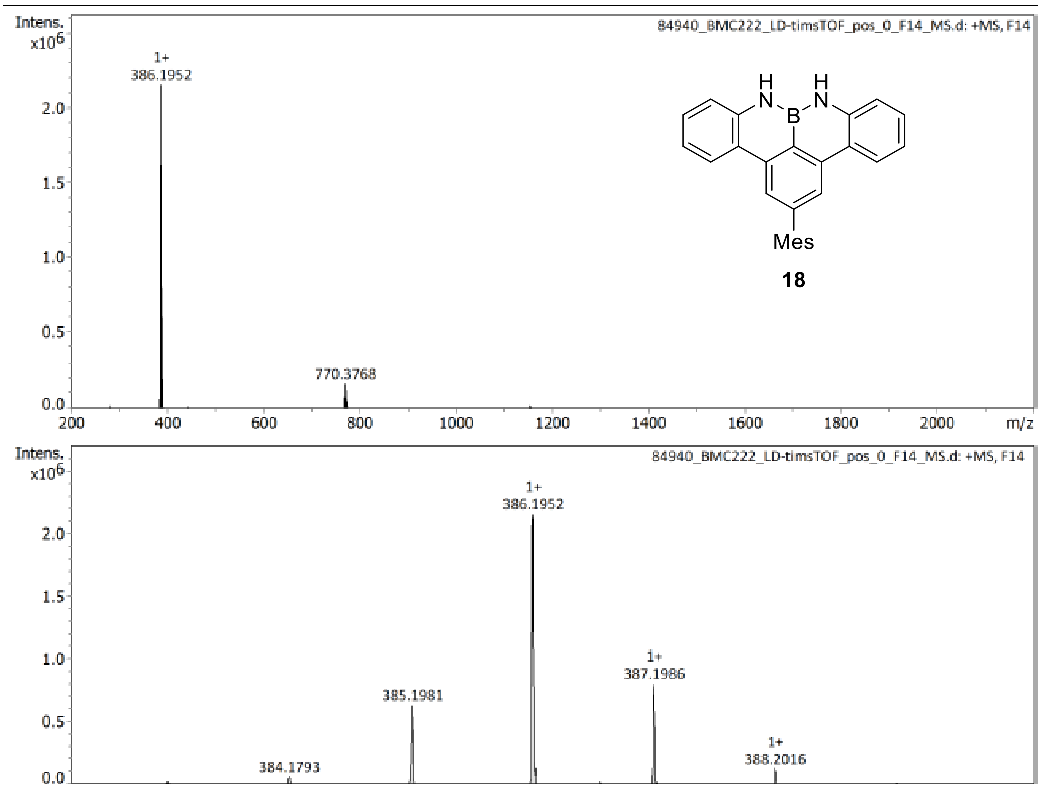

Figure S52. HRMS (LD-timsTOF) spectrum of **18**.

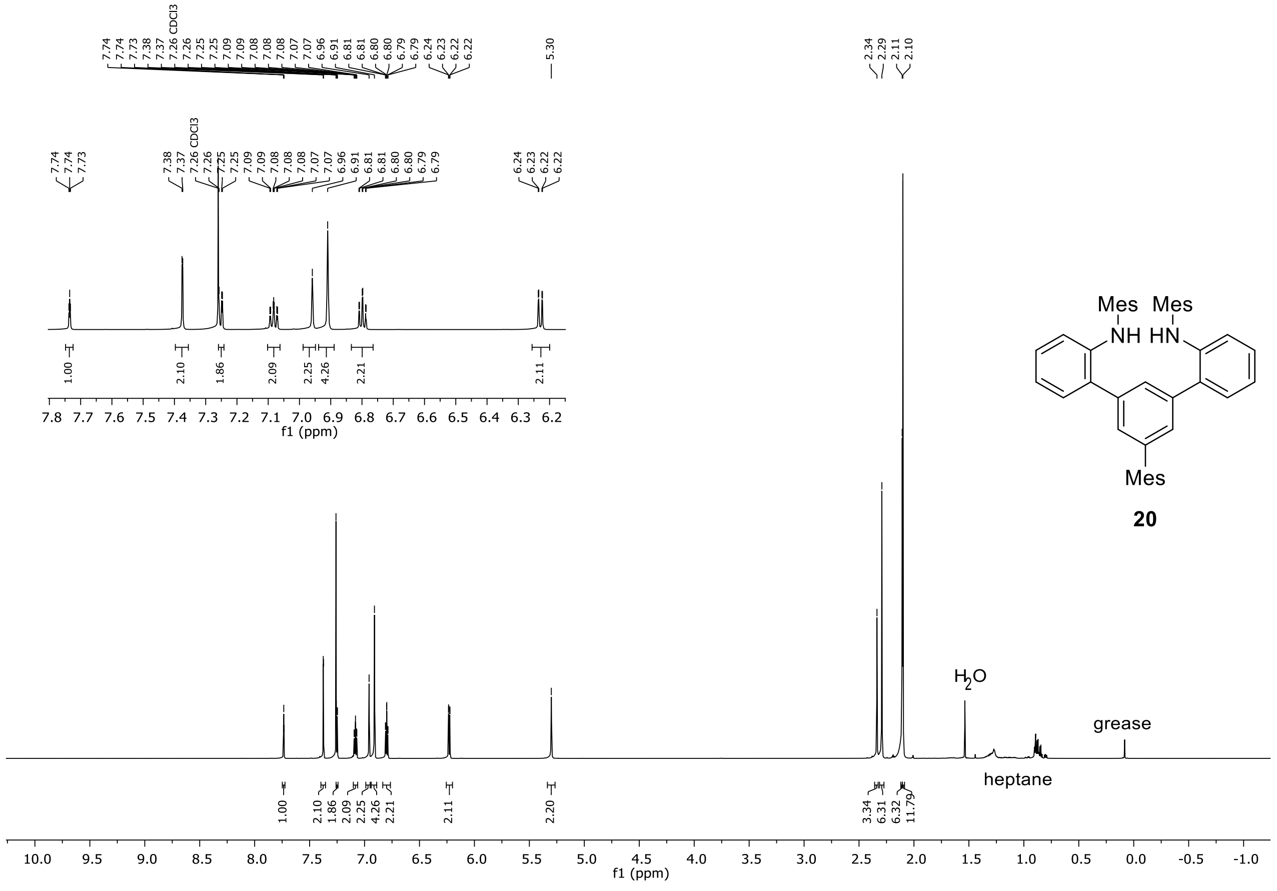

Figure S53. ^{1}H NMR (700 MHz, $CDCl_3$) spectrum of **20**.

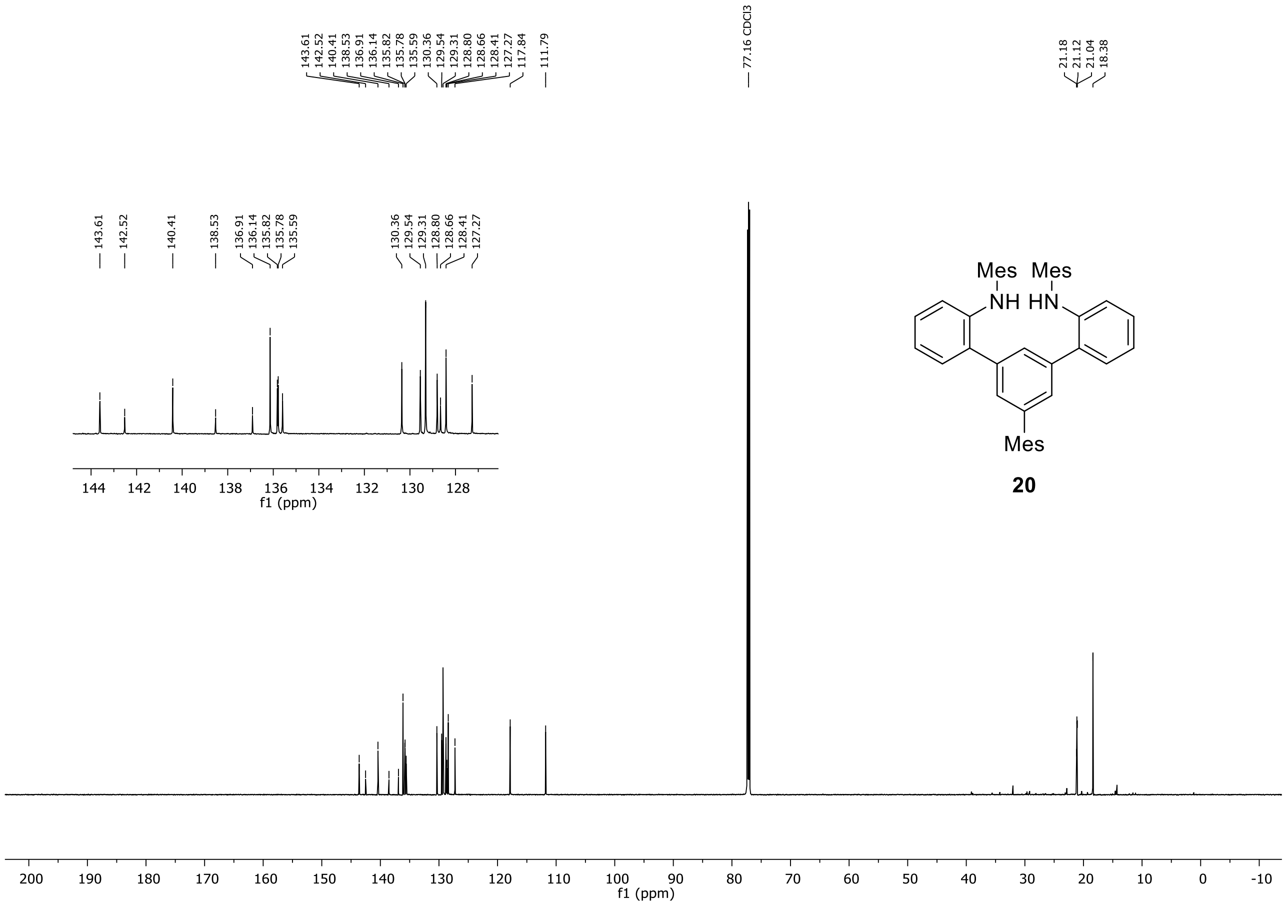

Figure S54. ^{13}C NMR (176 MHz, $CDCl_3$) spectrum of **20**.

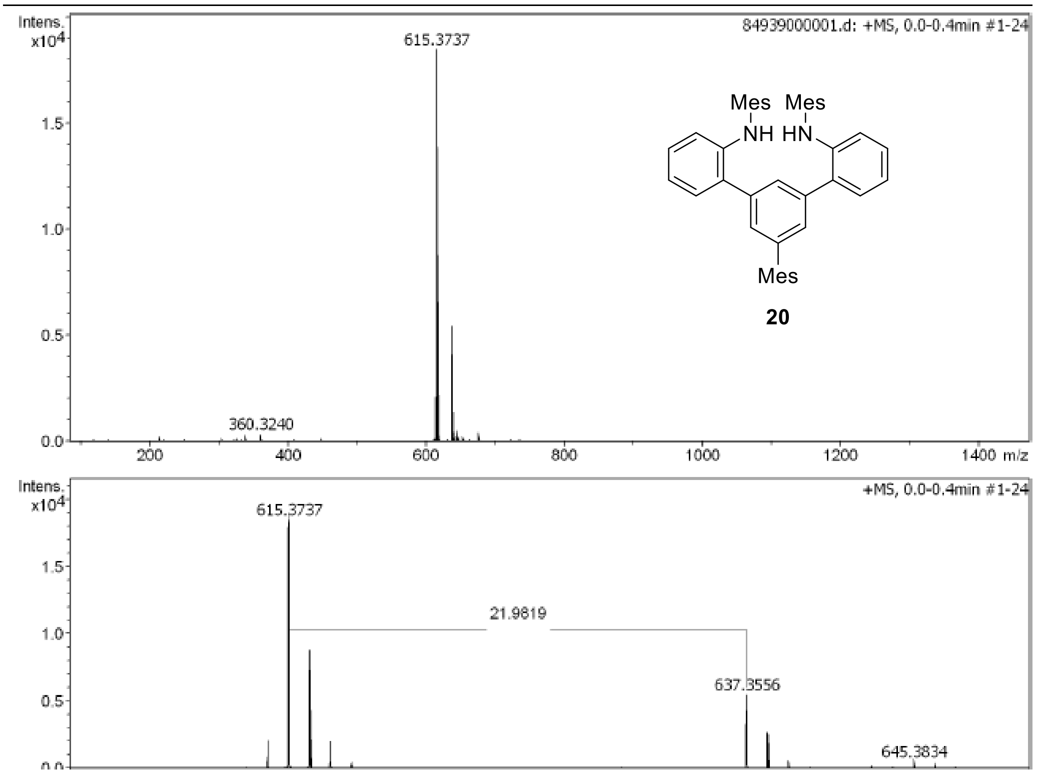

Figure S55. HRMS (ESI) spectrum of **20**.

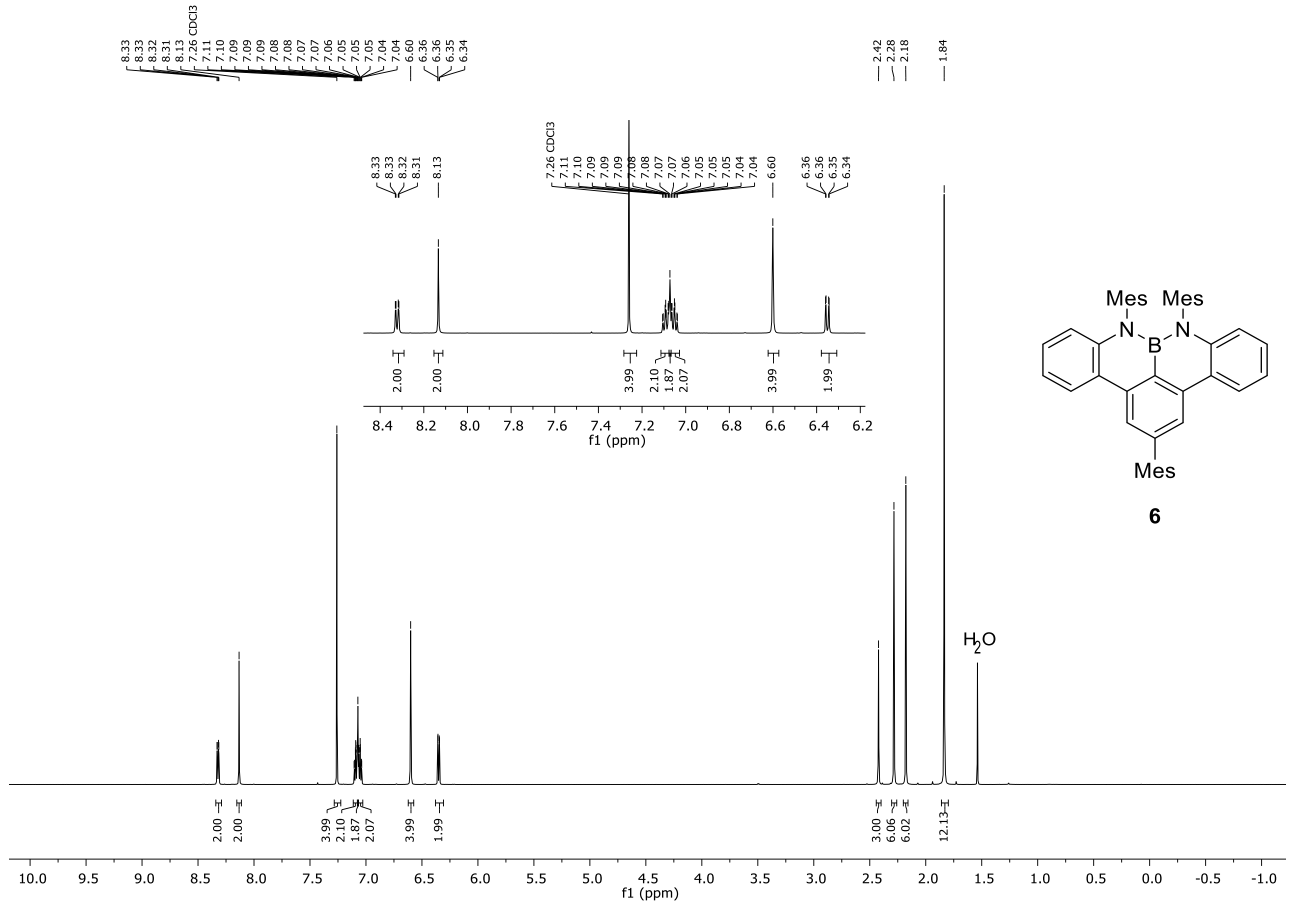

Figure S56. ^{1}H NMR (600 MHz, $CDCl_3$) spectrum of **6**.

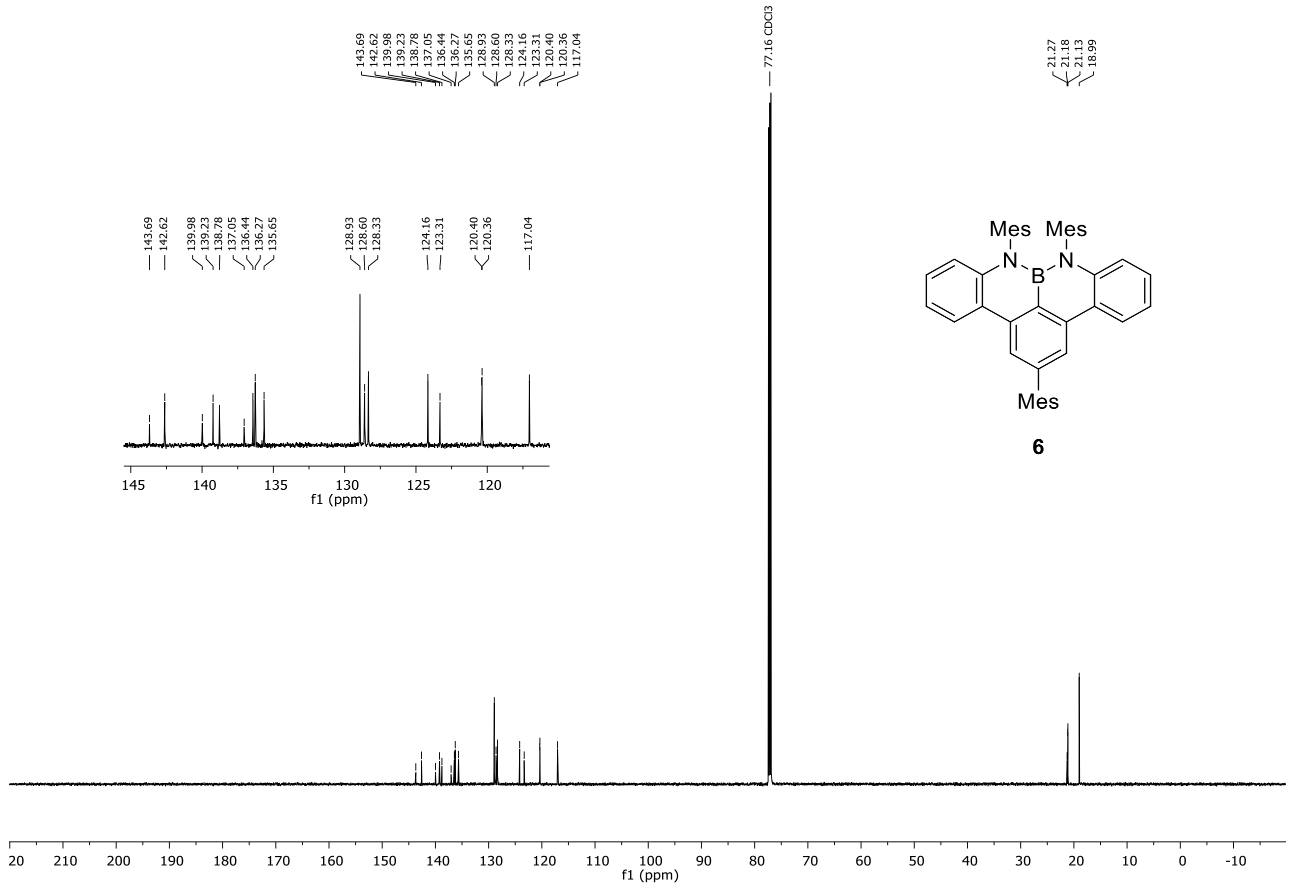

Figure S57. ^{13}C NMR (151 MHz, $CDCl_3$) spectrum of **6**.

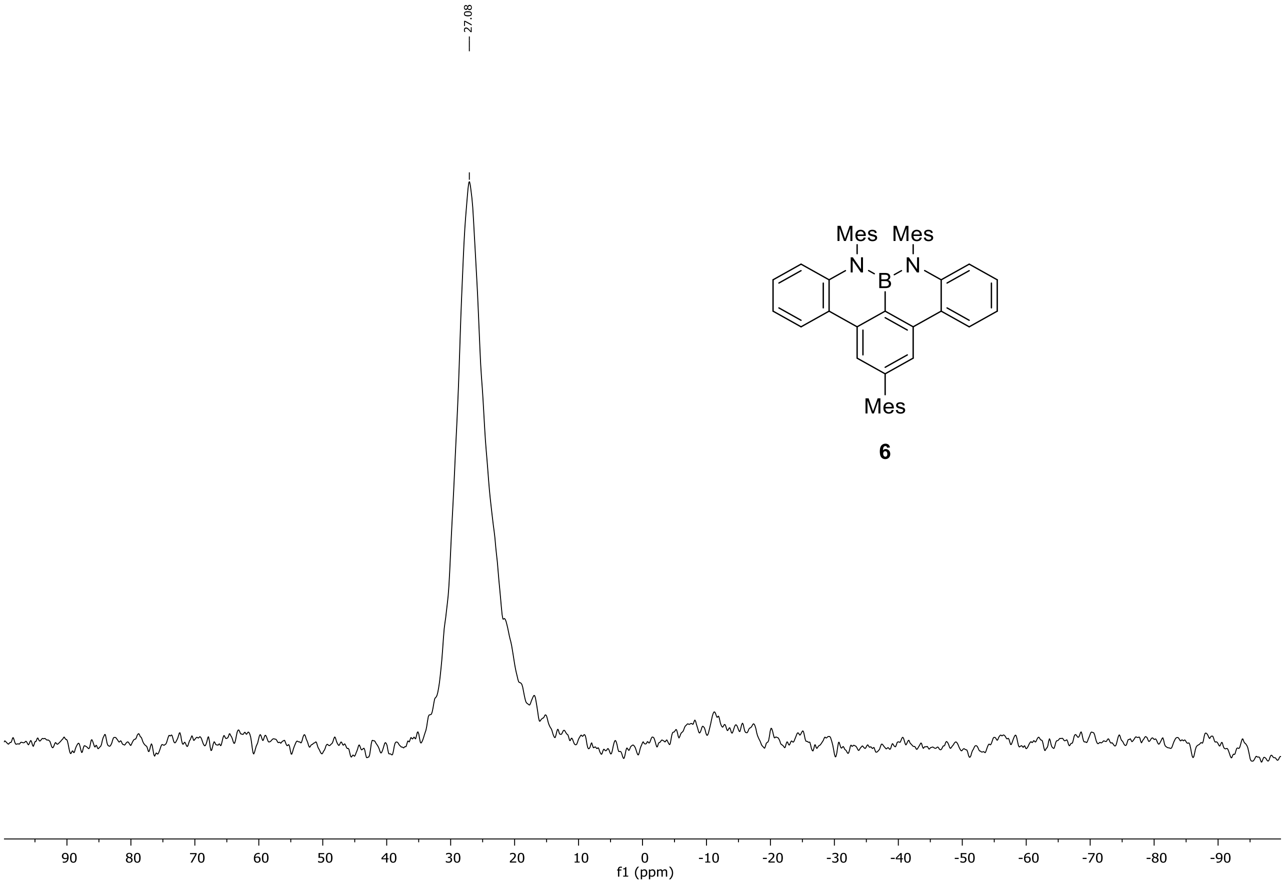

Figure S58. ^{11}B NMR (193 MHz, $CDCl_3$) spectrum of **6**.

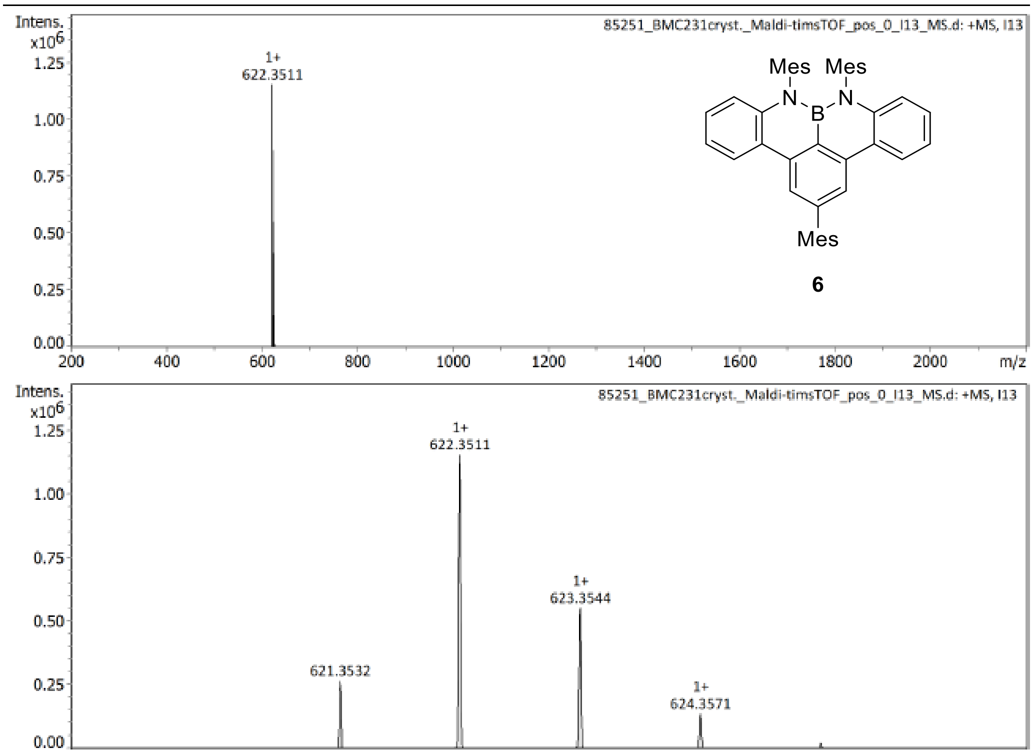

Figure S59. HRMS (MALDI-timsTOF, matrix: DCTB) spectrum of **6**.

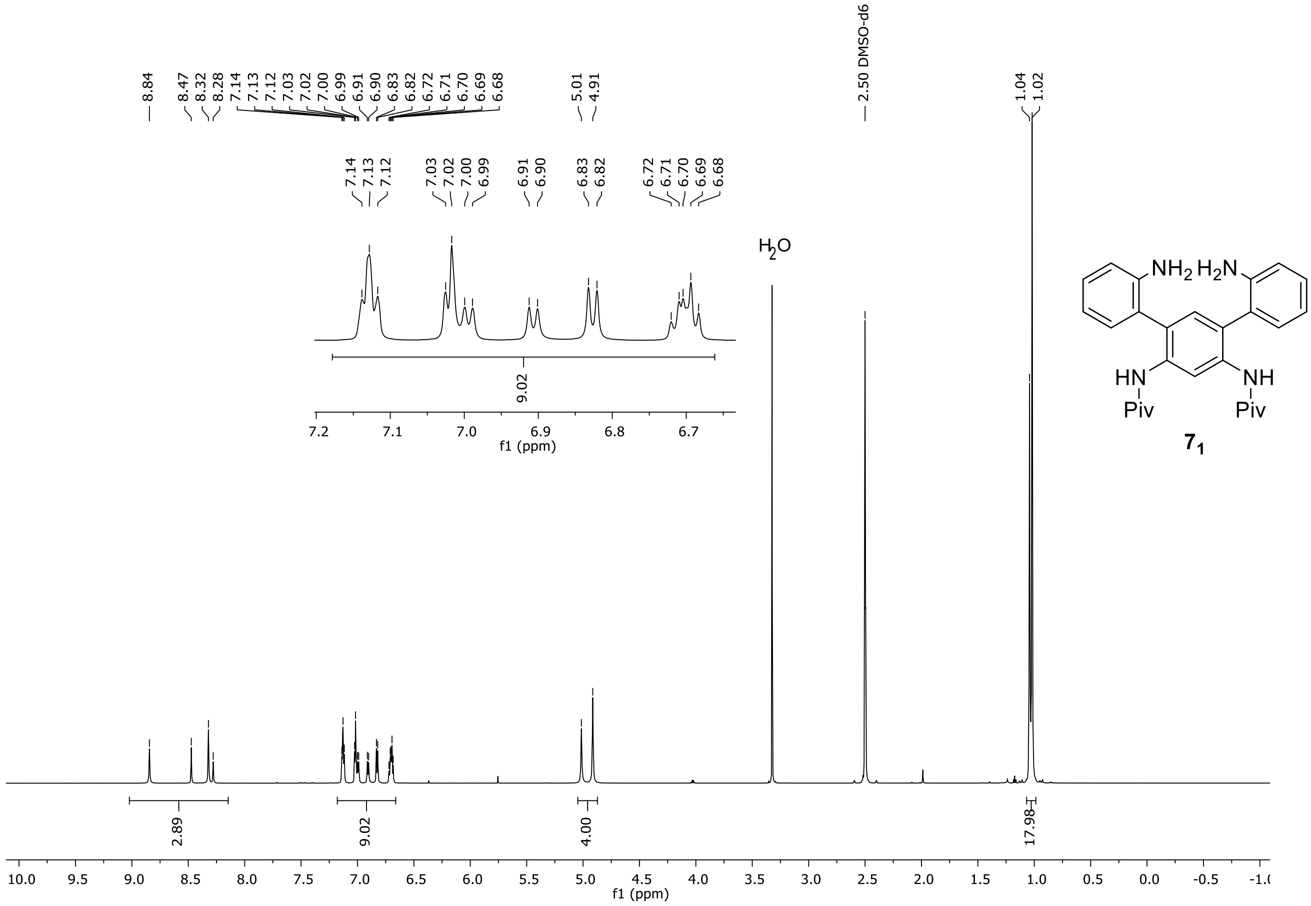

Figure S60. ^{1}H NMR (700 MHz, DMSO-d_6) spectrum of $\mathbf{7_1}$.

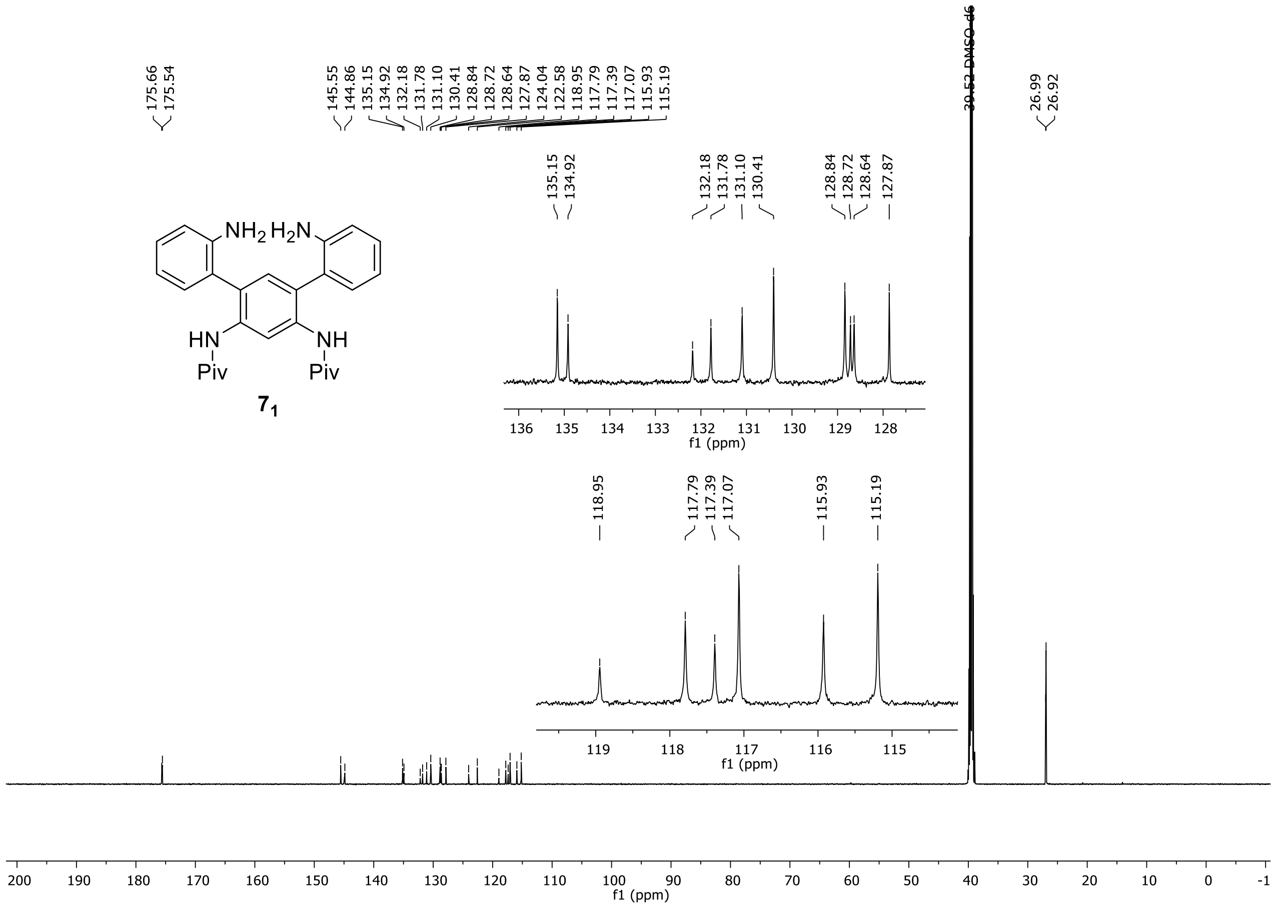

Figure S61. ^{13}C NMR (176 MHz, DMSO-d_6) spectrum of $\mathbf{7_1}$.

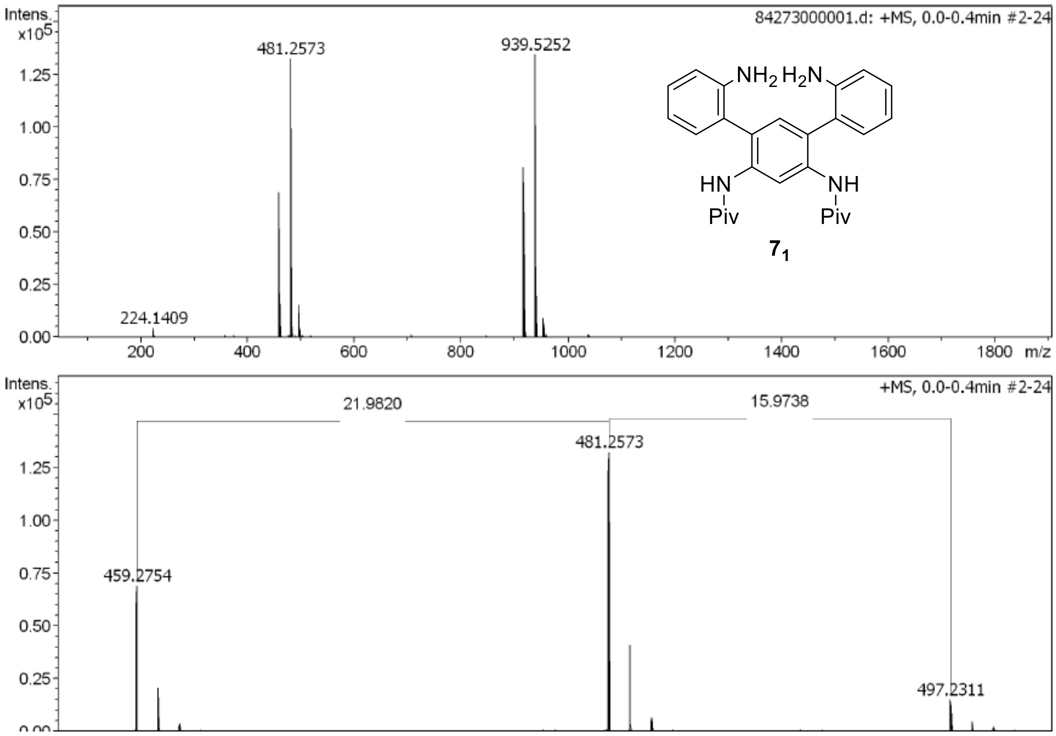

Figure S62. HRMS (ESI) spectrum of $\mathbf{7_1}$.

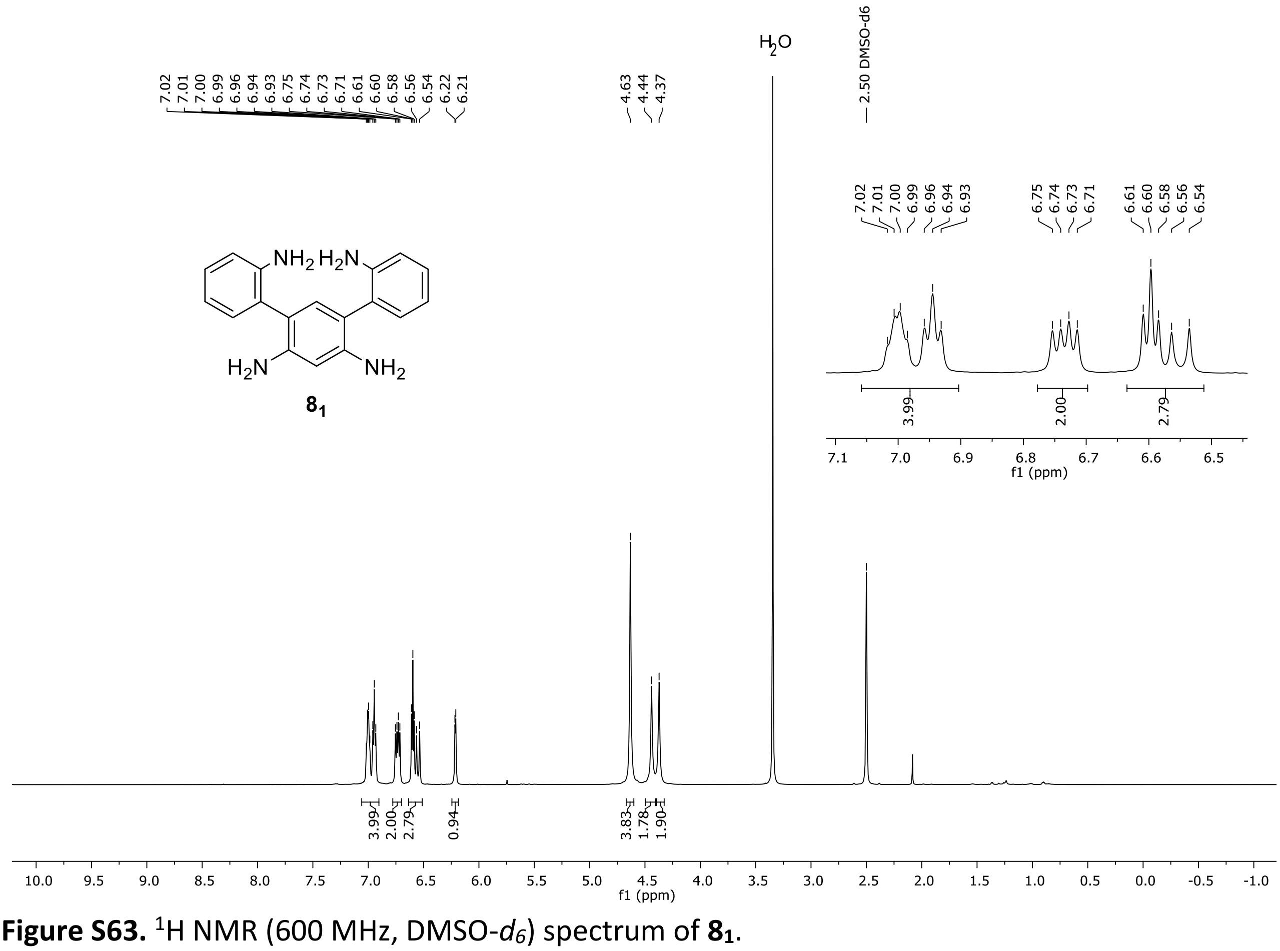

Figure S63. ^{1}H NMR (600 MHz, DMSO-d_6) spectrum of $\mathbf{8_1}$.

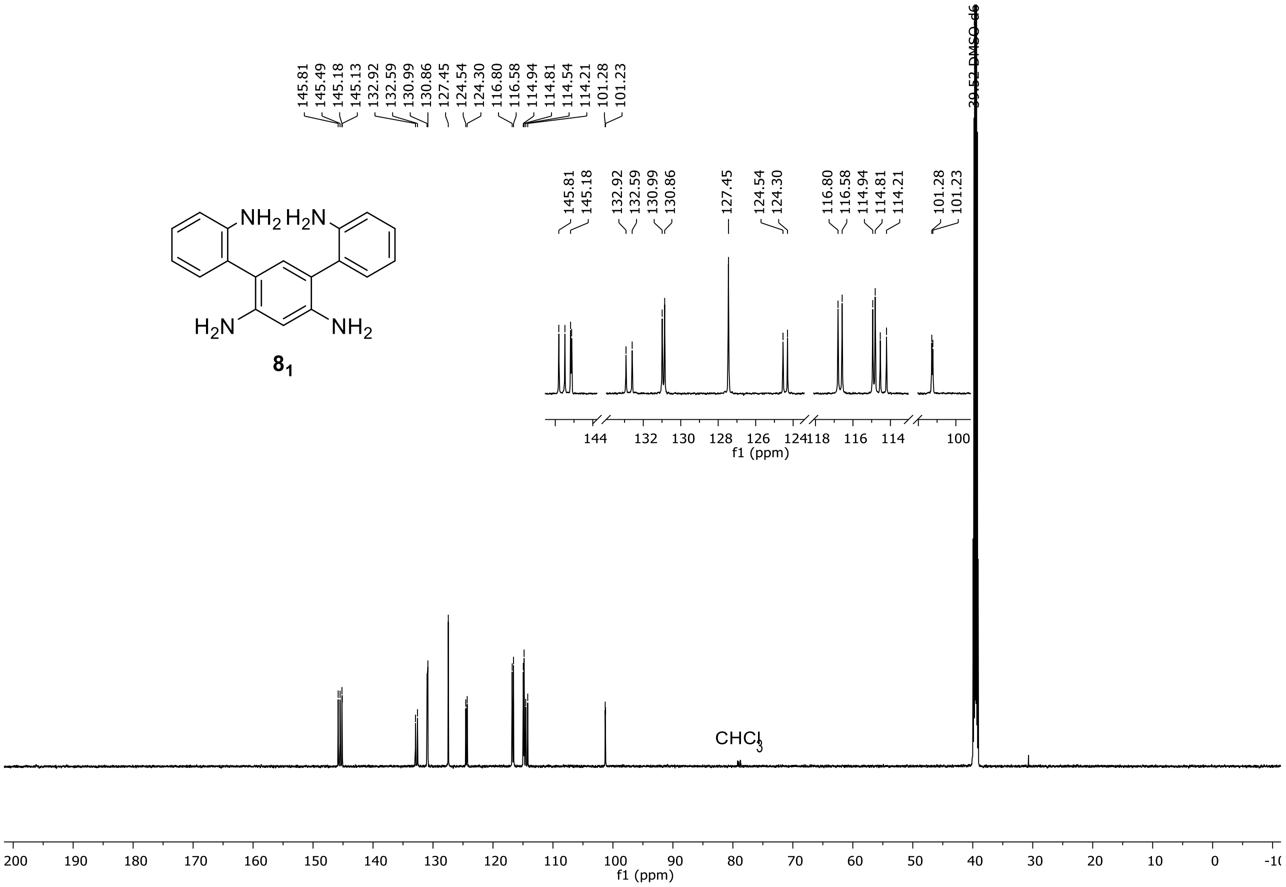

Figure S64. ^{13}C NMR (151 MHz, DMSO-d_6) spectrum of $\mathbf{8_1}$.

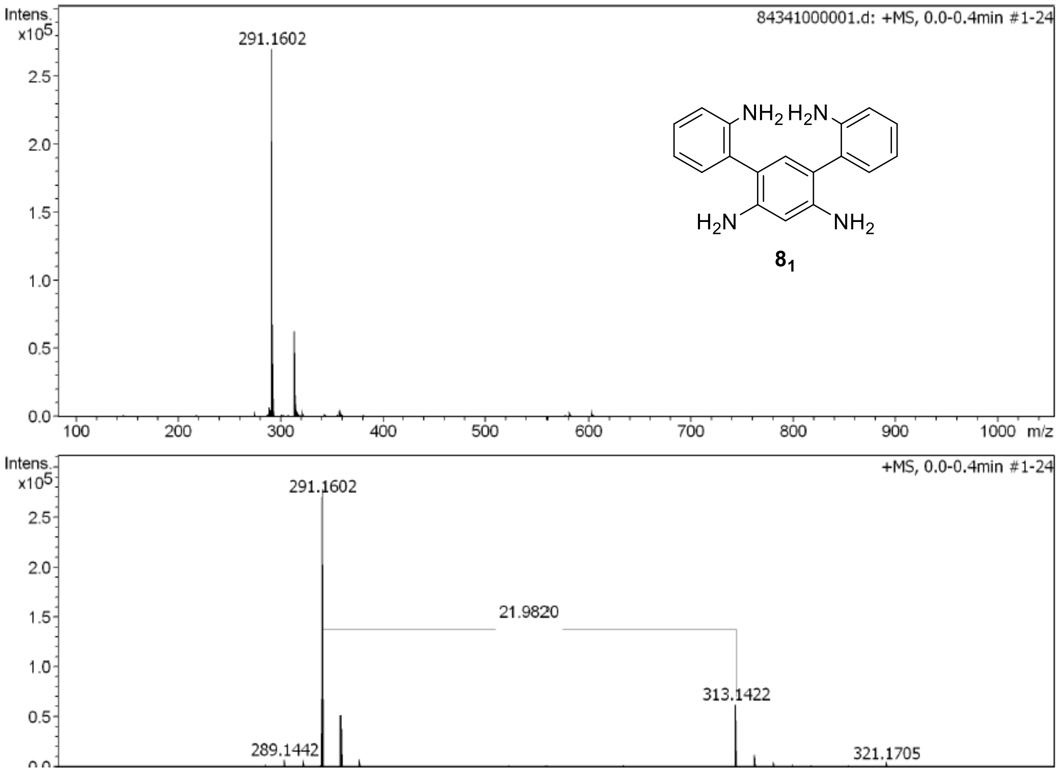

Figure S65. HRMS (ESI) spectrum of $\mathbf{8_1}$.

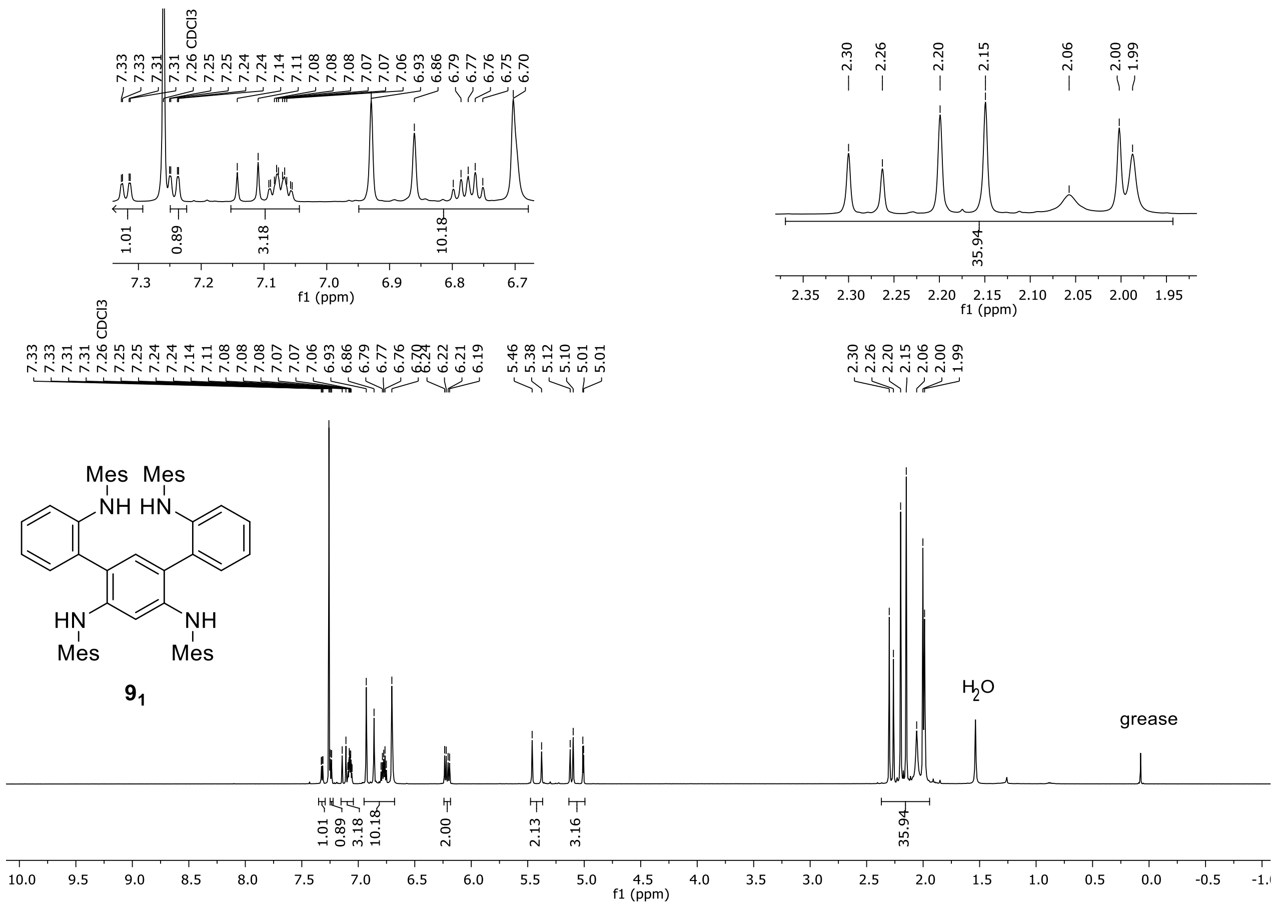

Figure S66. ^1H NMR (600 MHz, $CDCl_3$) spectrum of $\mathbf{9_1}$.

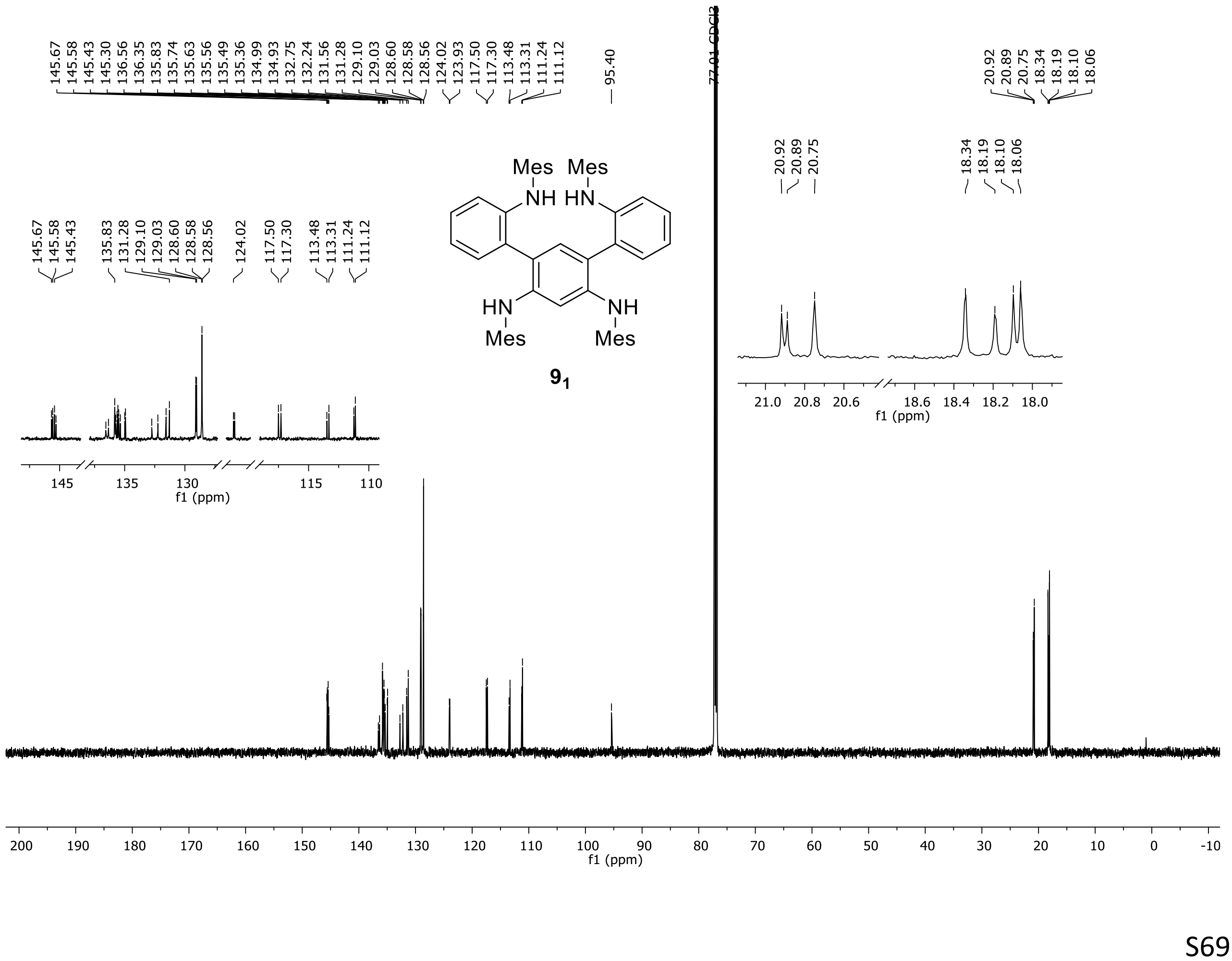

Figure S67. ^{13}C NMR (151 MHz, $CDCl_3$) spectrum of **9_1**.

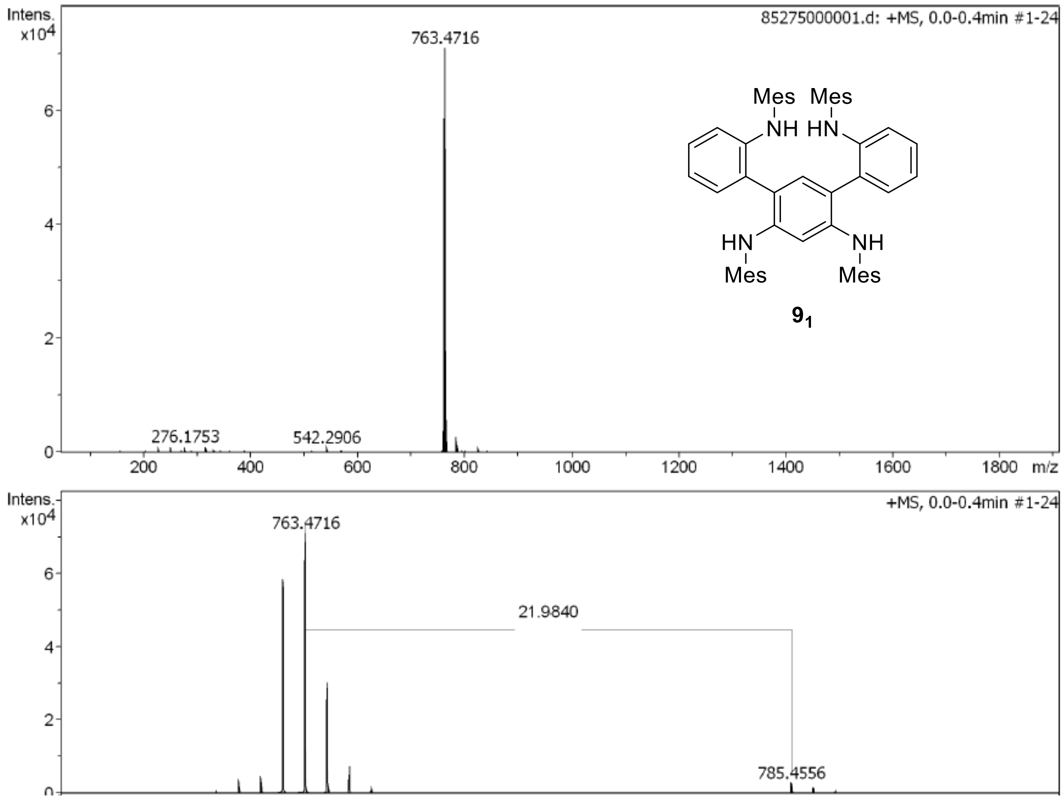

Figure S68. HRMS (ESI) spectrum of **9_1**.

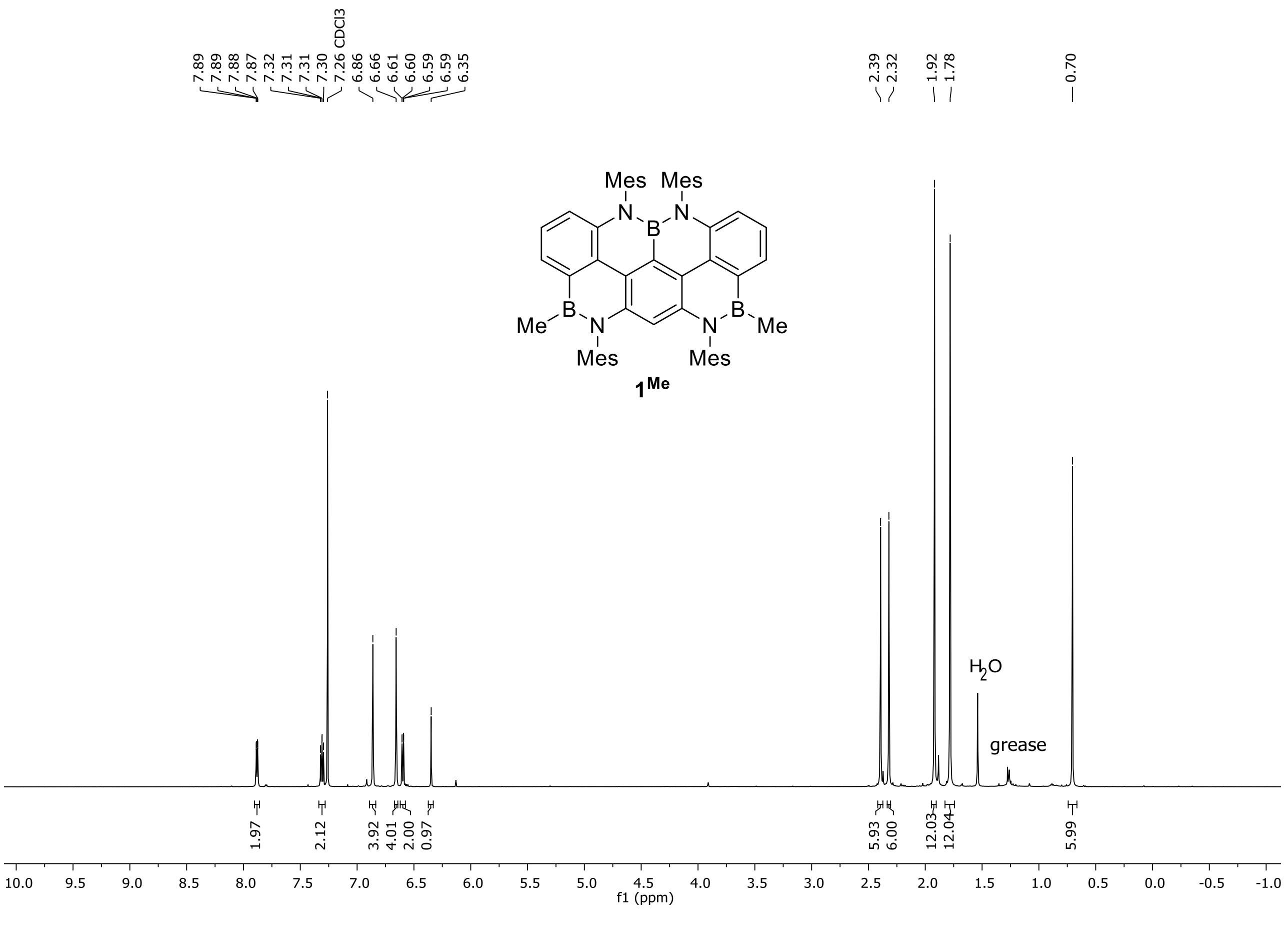

Figure S69. ^{1}H NMR (600 MHz, $CDCl_3$) spectrum of $\mathbf{1^{Me}}$.

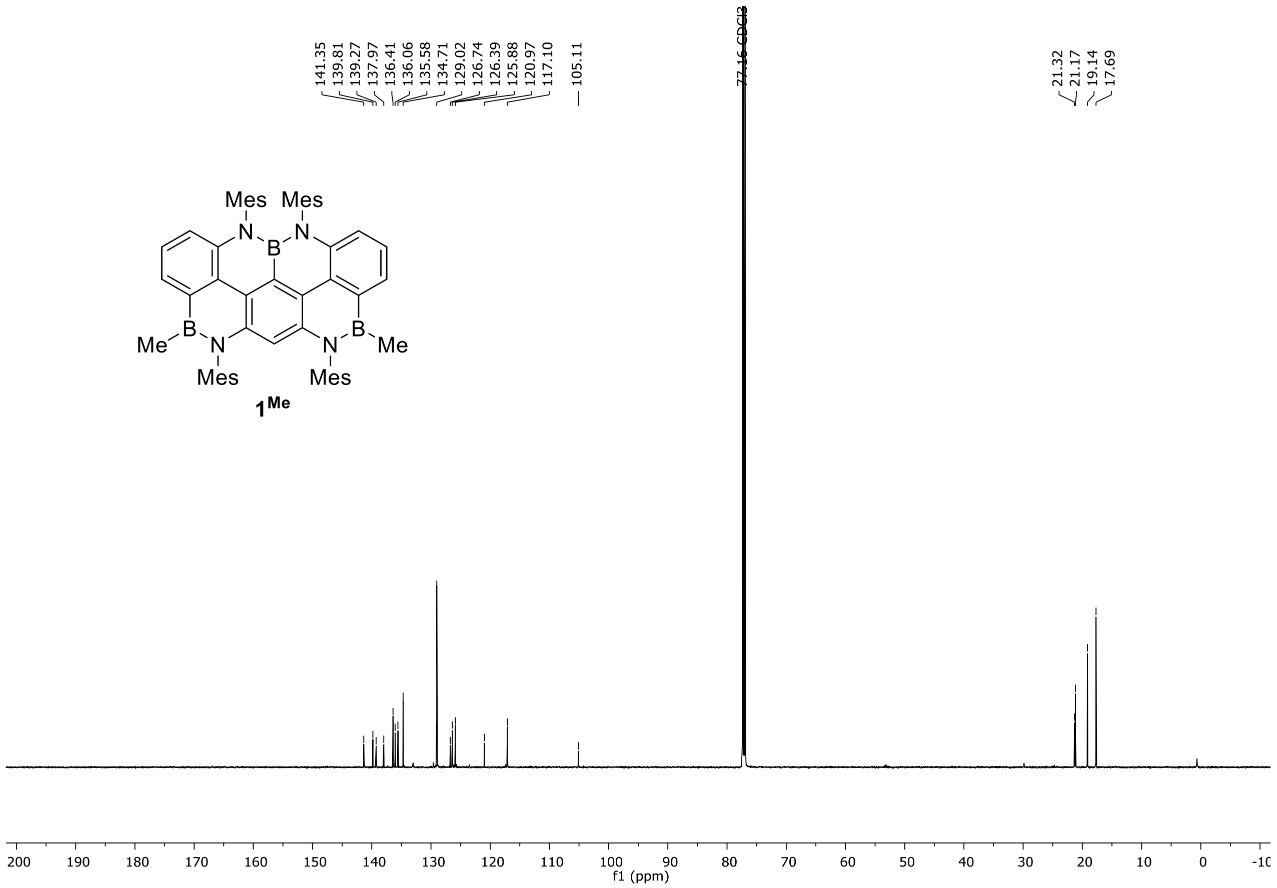

Figure S70. ^{13}C NMR (151 MHz, $CDCl_3$) spectrum of $\mathbf{1^{Me}}$.

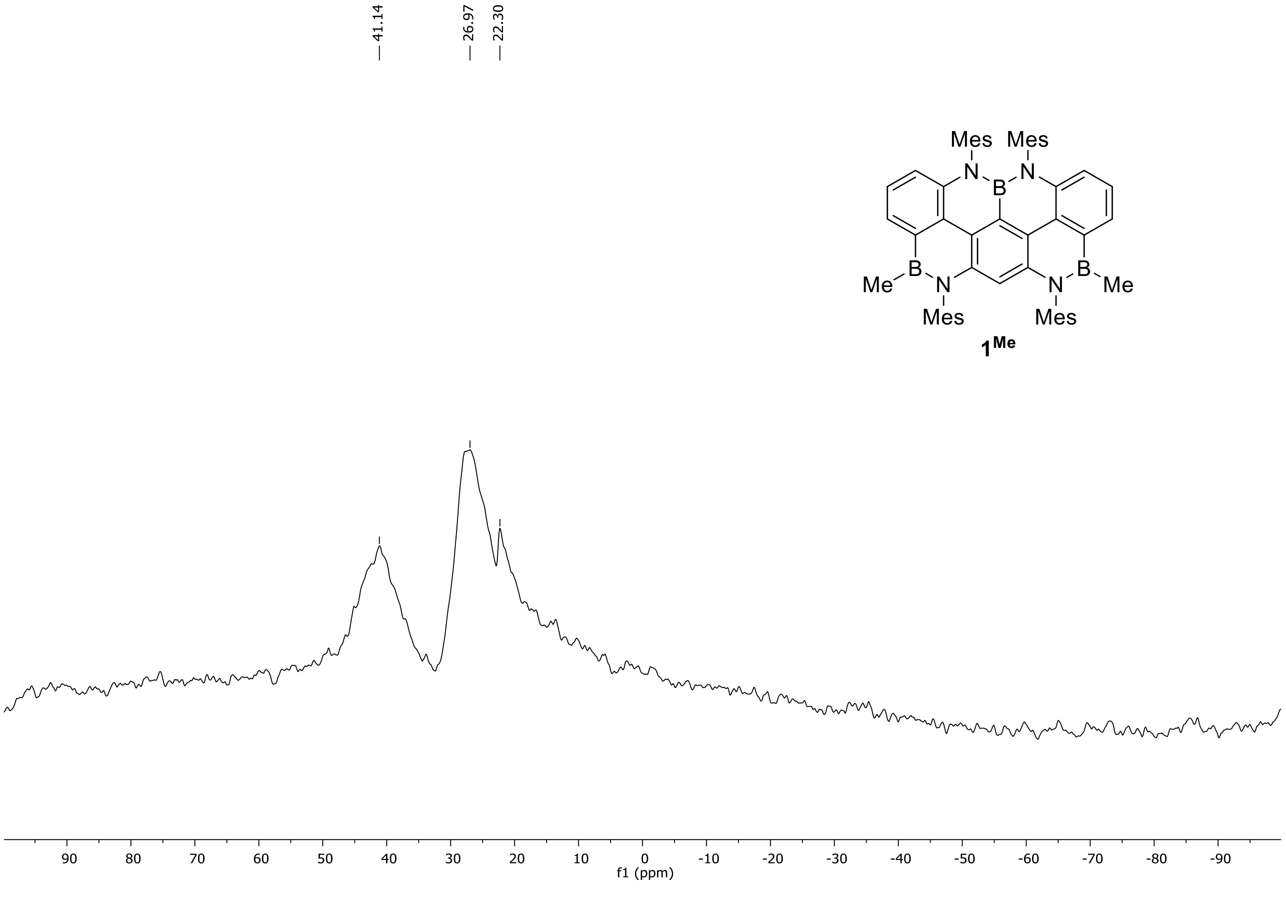

Figure S71. ^{11}B NMR (193 MHz, CDCl$_3$) spectrum of **1^{Me}**.

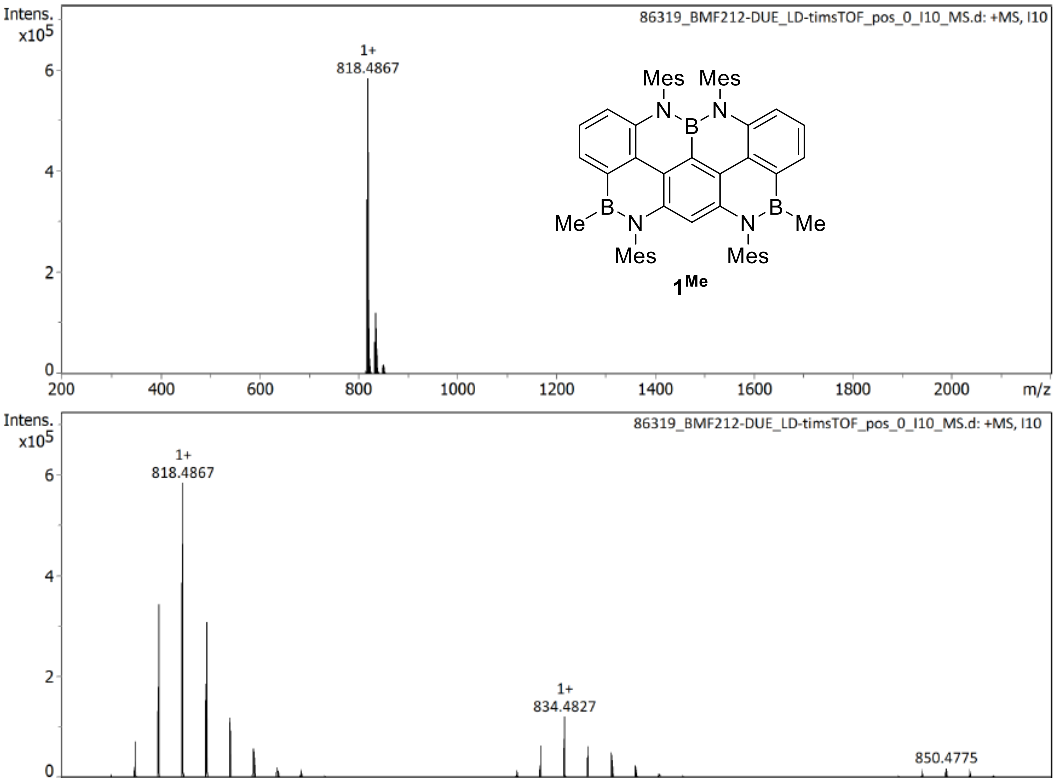

Figure S72. HRMS (LD-timsTOF) spectrum of **1^{Me}**.

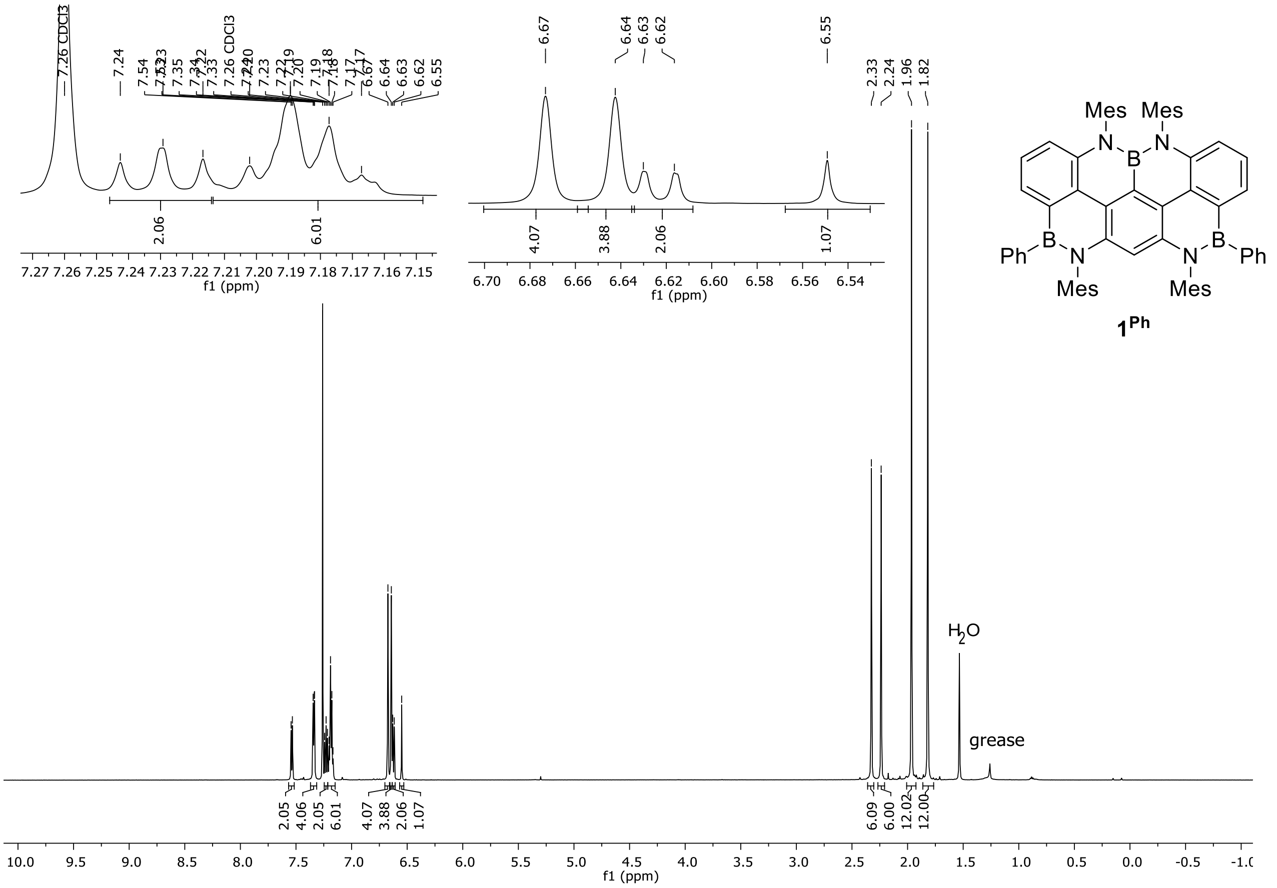

Figure S73. ^{1}H NMR (600 MHz, CDCl$_3$) spectrum of **1^{Ph}**.

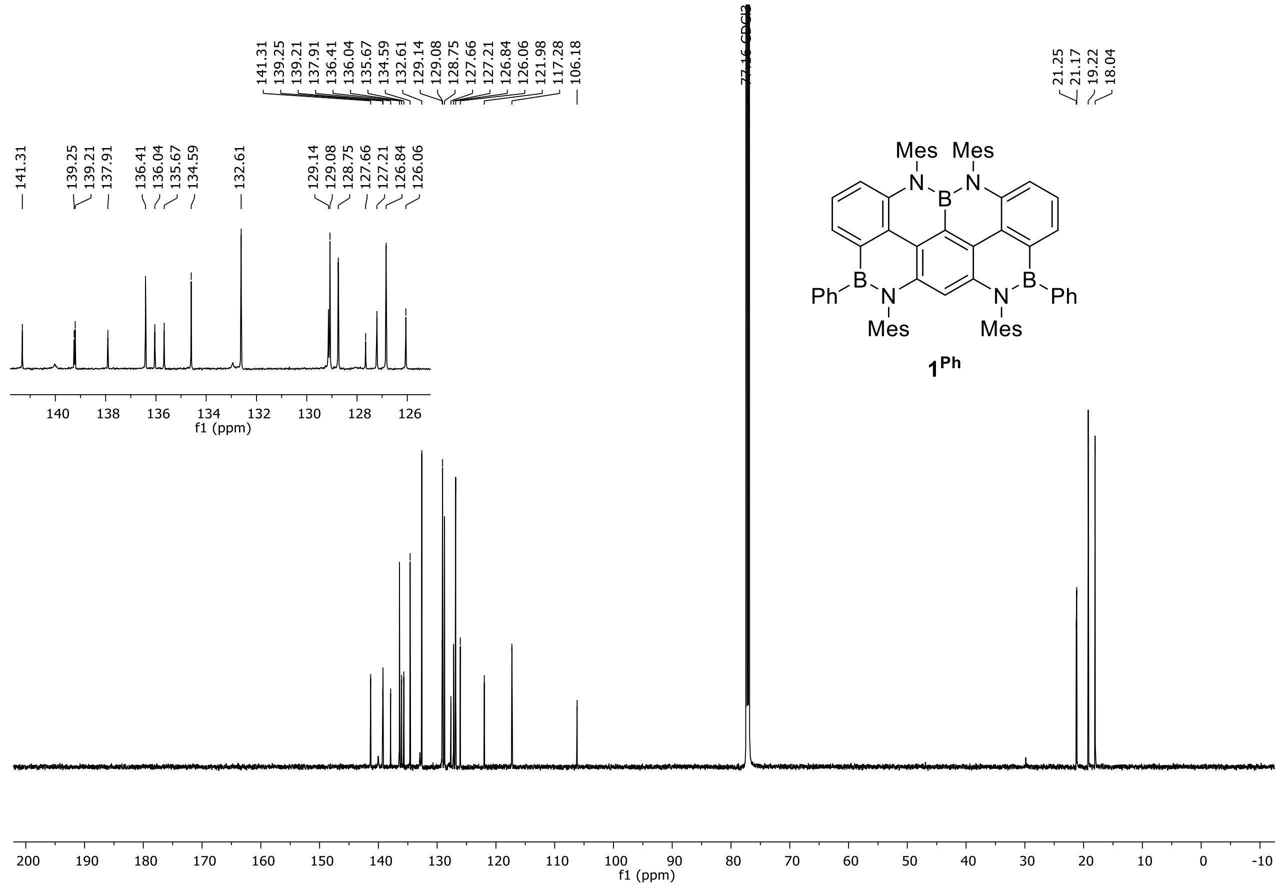

Figure S74. ^{13}C NMR (151 MHz, CDCl$_3$) spectrum of $\mathbf{1^{Ph}}$.

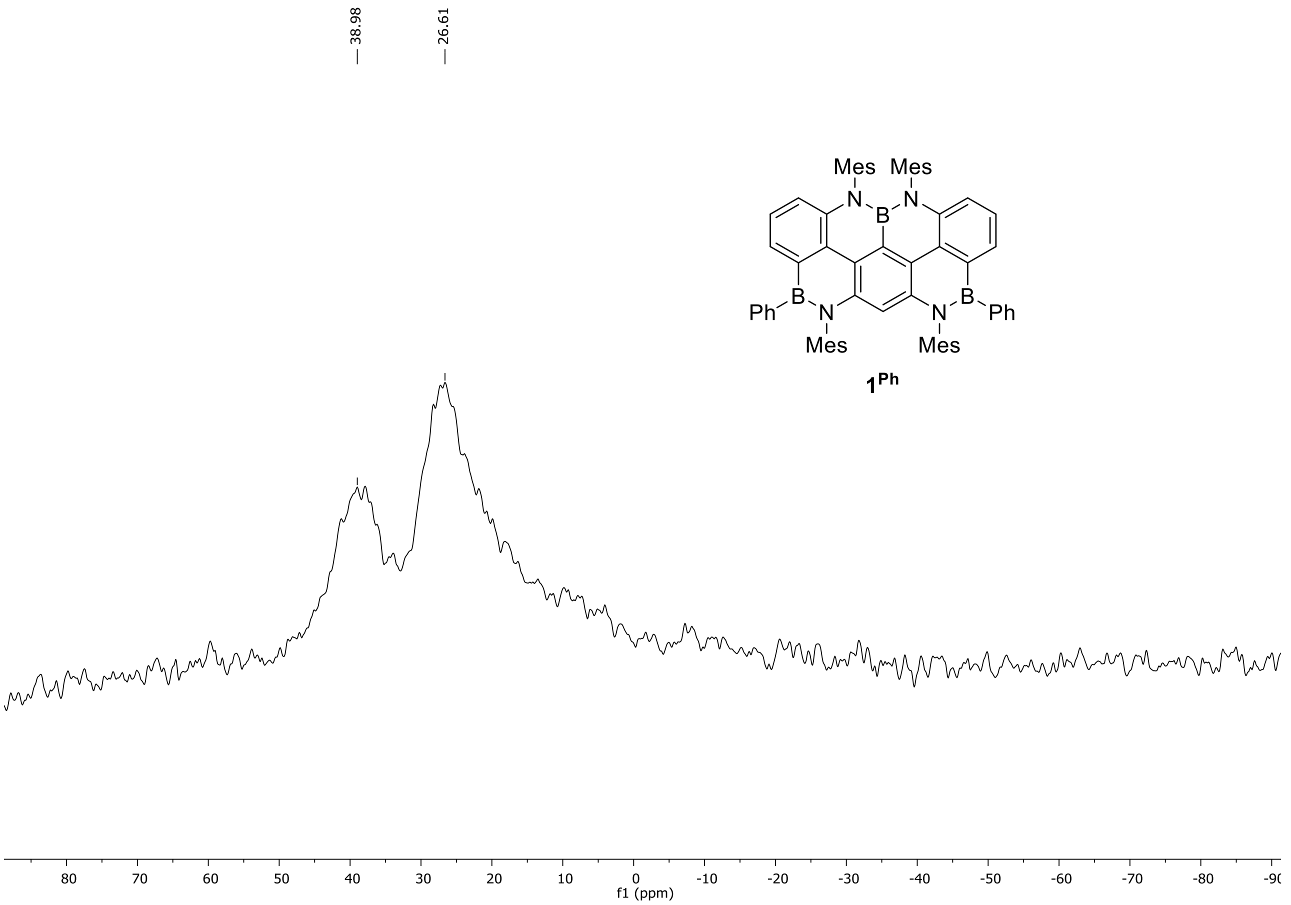

Figure S75. ^{11}B NMR (193 MHz, CDCl$_3$) spectrum of $\mathbf{1^{Ph}}$.

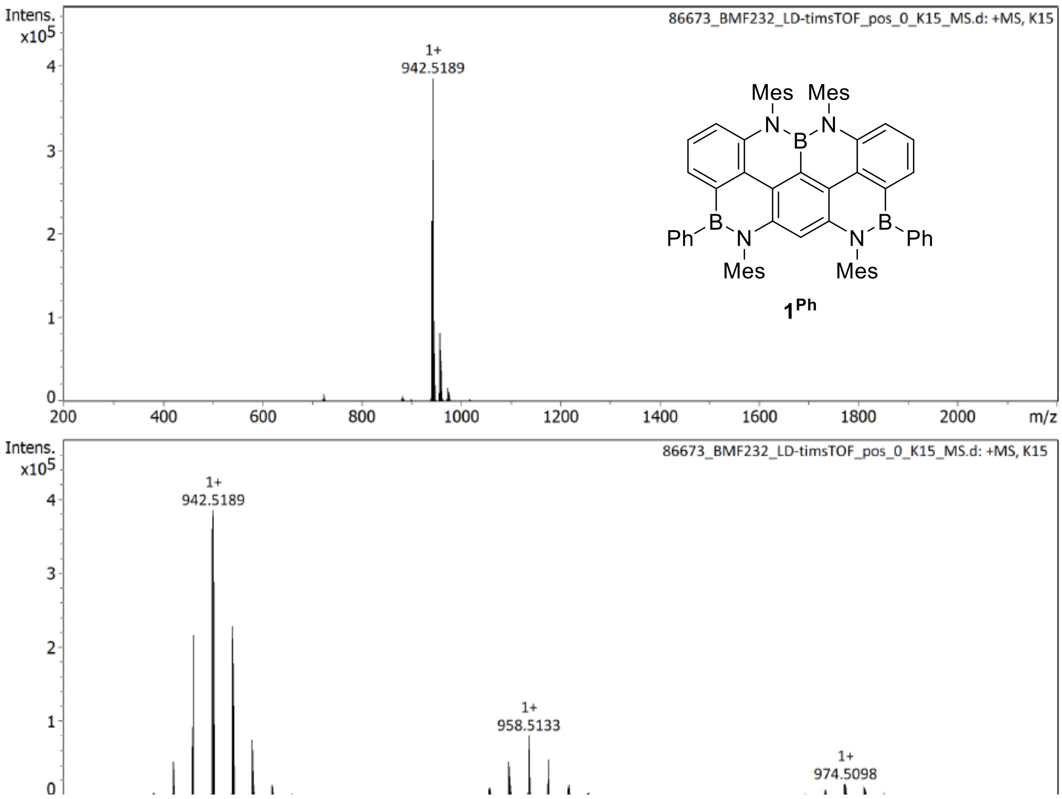

Figure S76. HRMS (LD-timsTOF) spectrum of **1^{Ph}**.

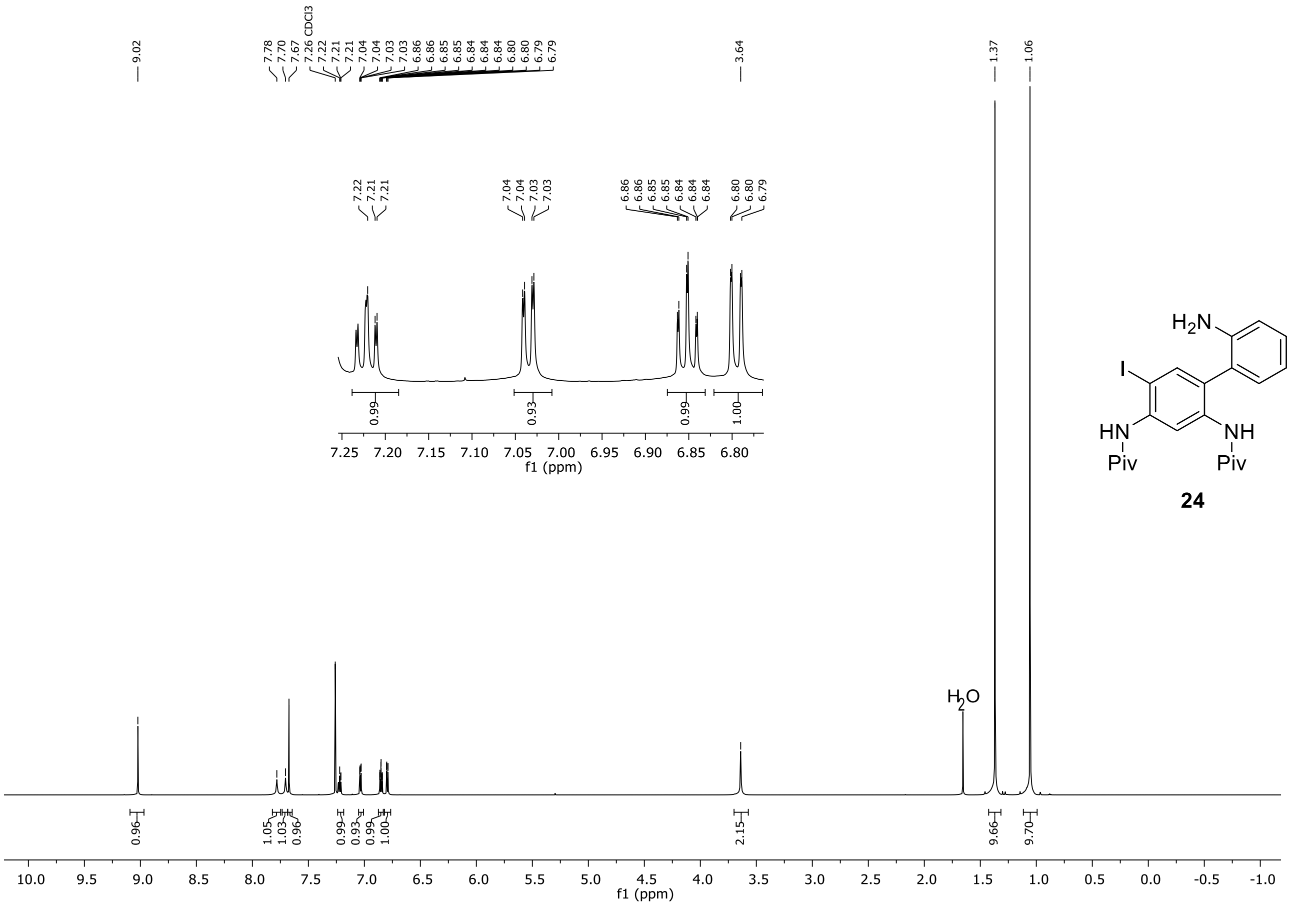

Figure S77. ^{1}H NMR (700 MHz, $CDCl_3$) spectrum of **24**.

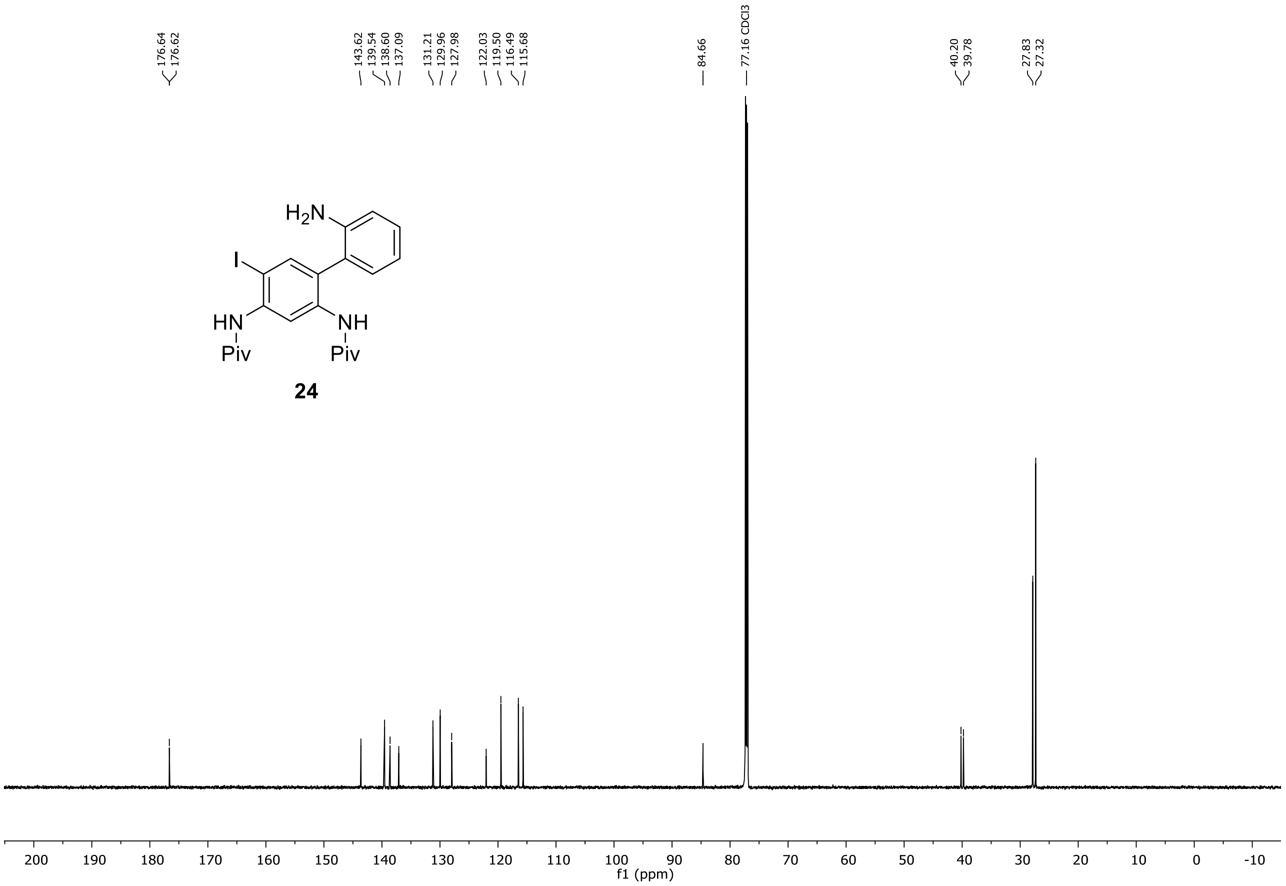

Figure S78. ^{13}C NMR (176 MHz, $CDCl_3$) spectrum of **24**.

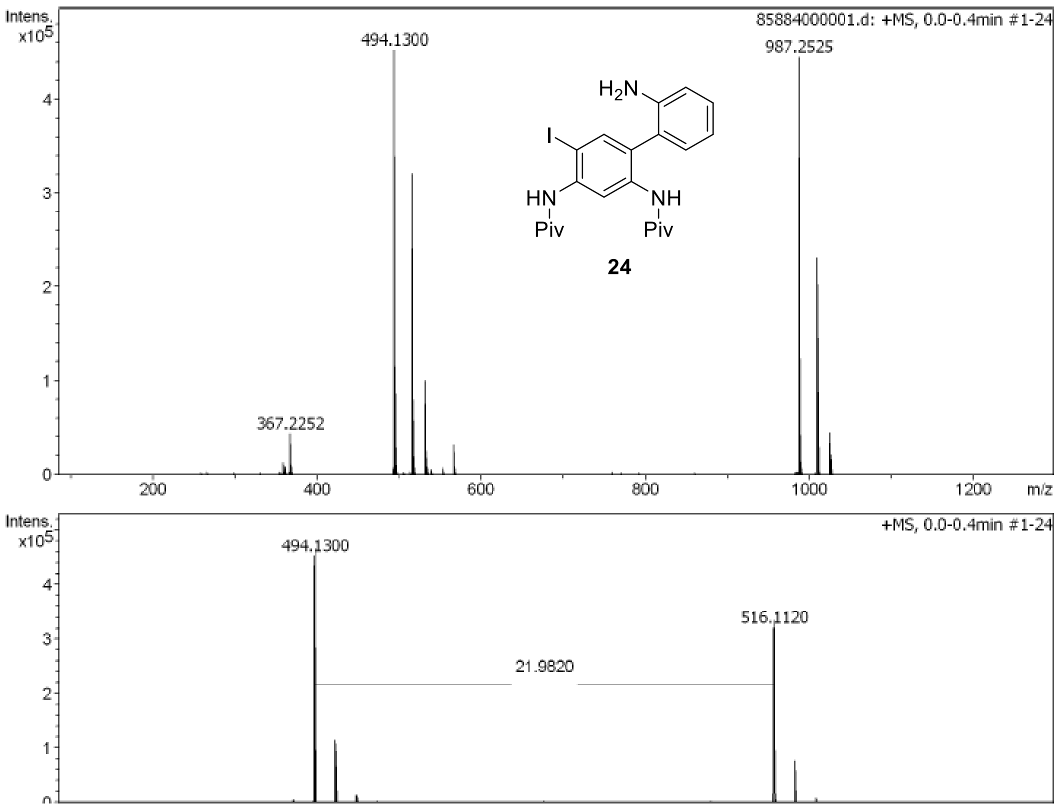

Figure S79. HRMS (ESI) spectrum of **24**.

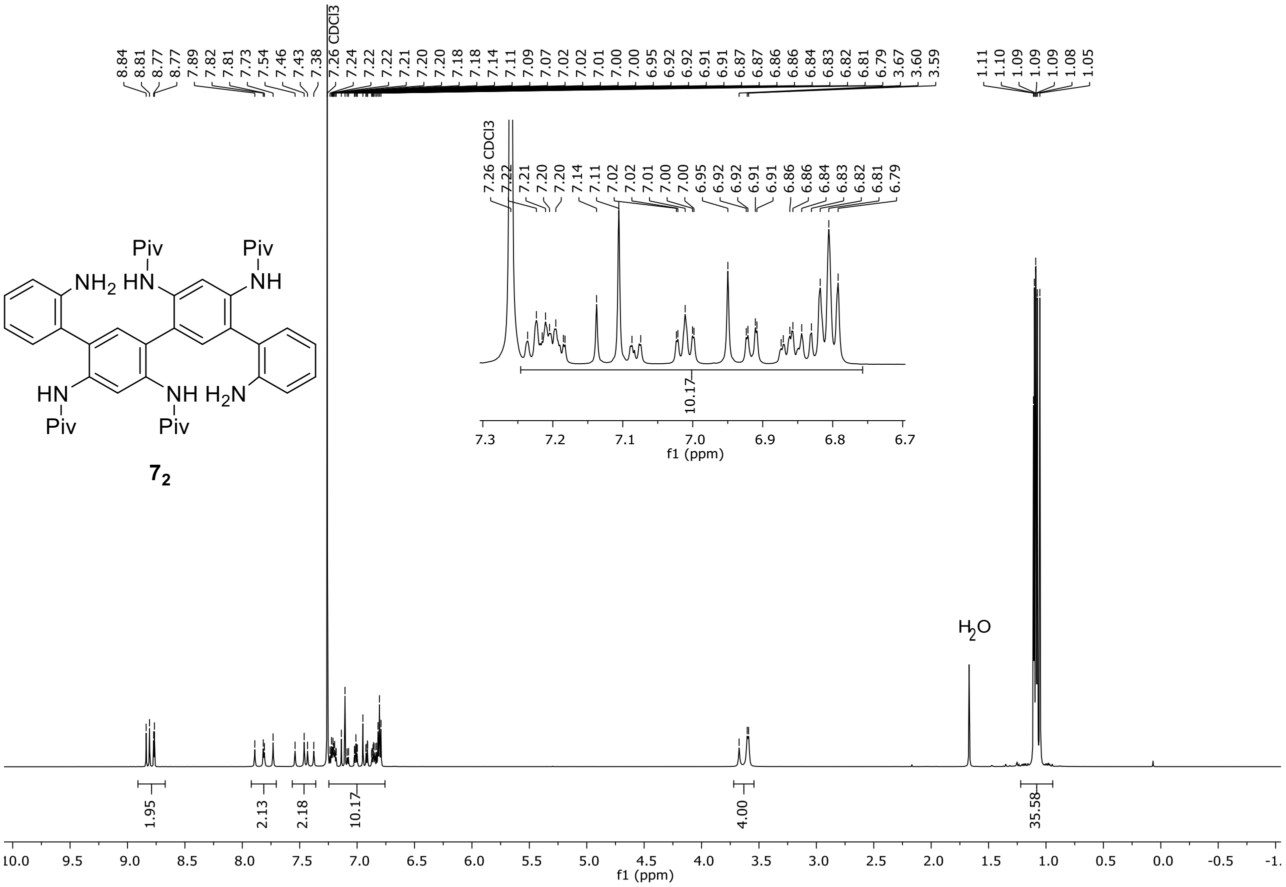

Figure S80. ^{1}H NMR (600 MHz, $CDCl_3$) spectrum of **7_2**.

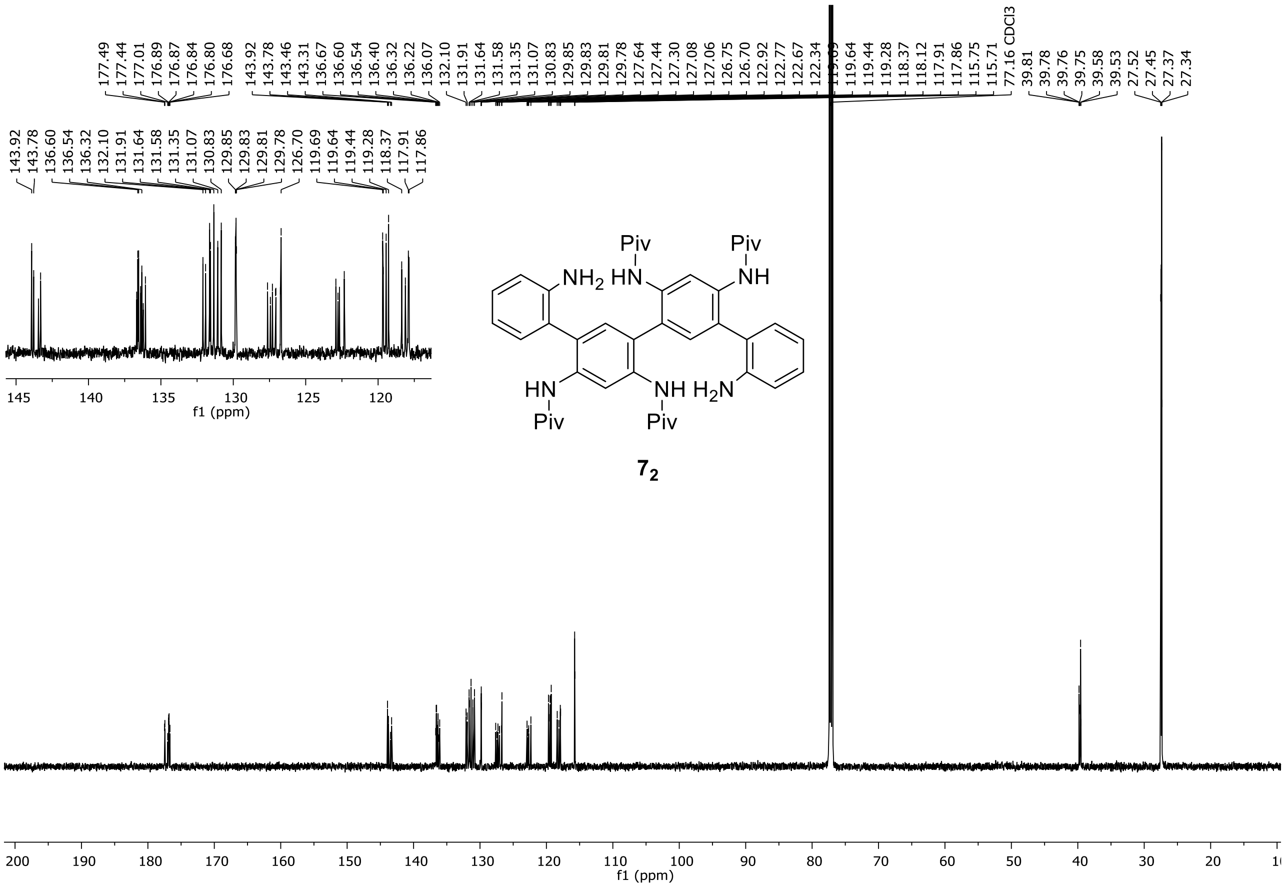

Figure S81. ^{13}C NMR (151 MHz, $CDCl_3$) spectrum of **7_2**.

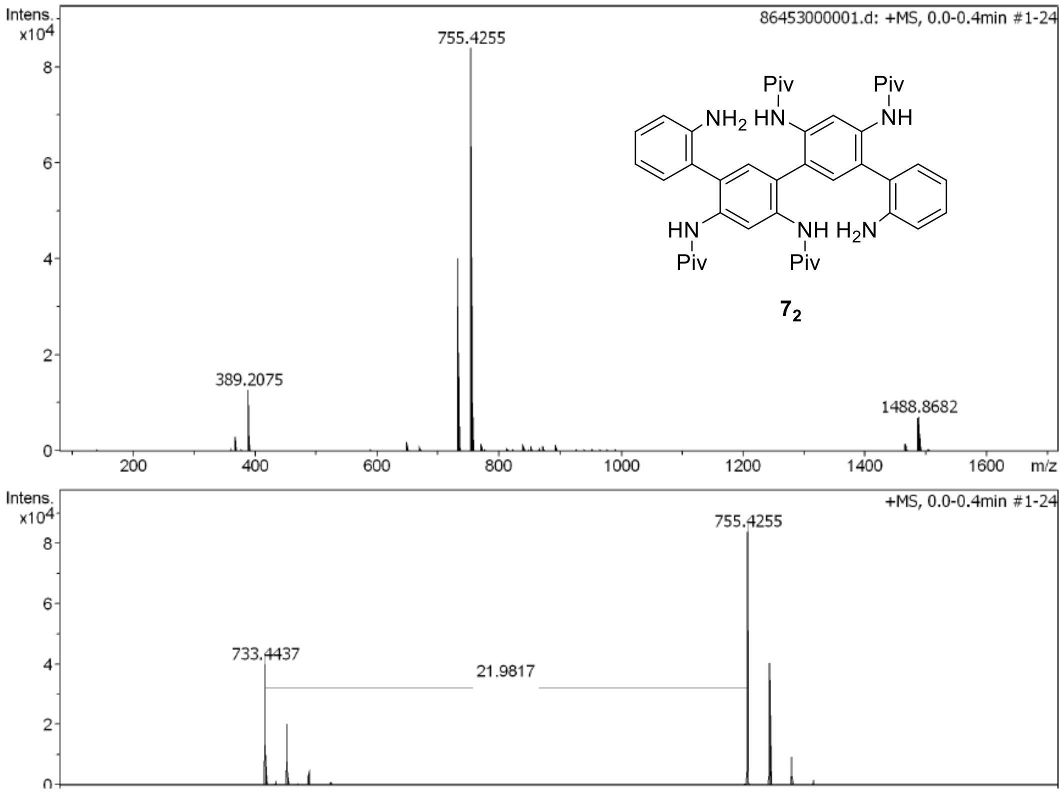

Figure S82. HRMS (ESI) spectrum of $\mathbf{7_2}$.

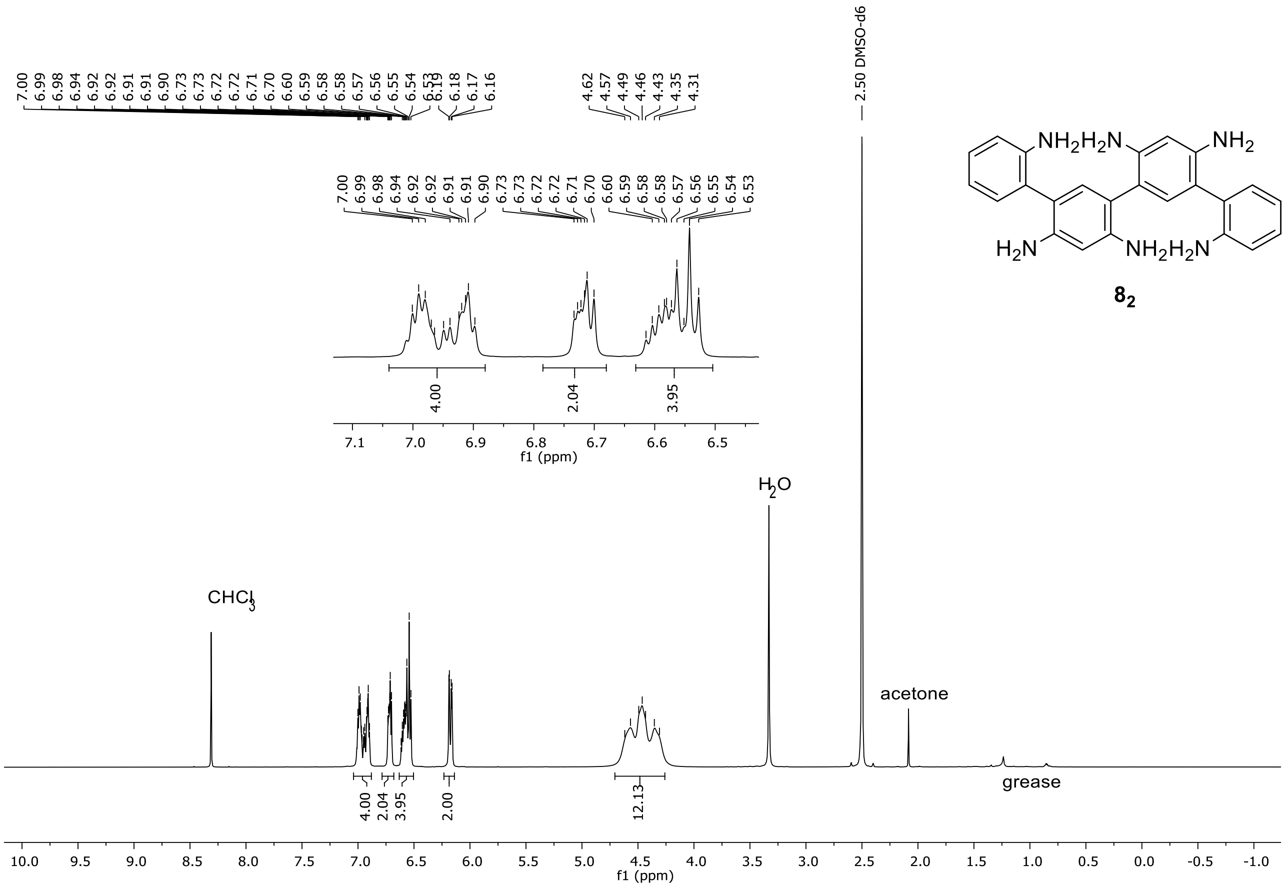

Figure S83. ^{1}H NMR (700 MHz, DMSO-d_6) spectrum of $\mathbf{8_2}$.

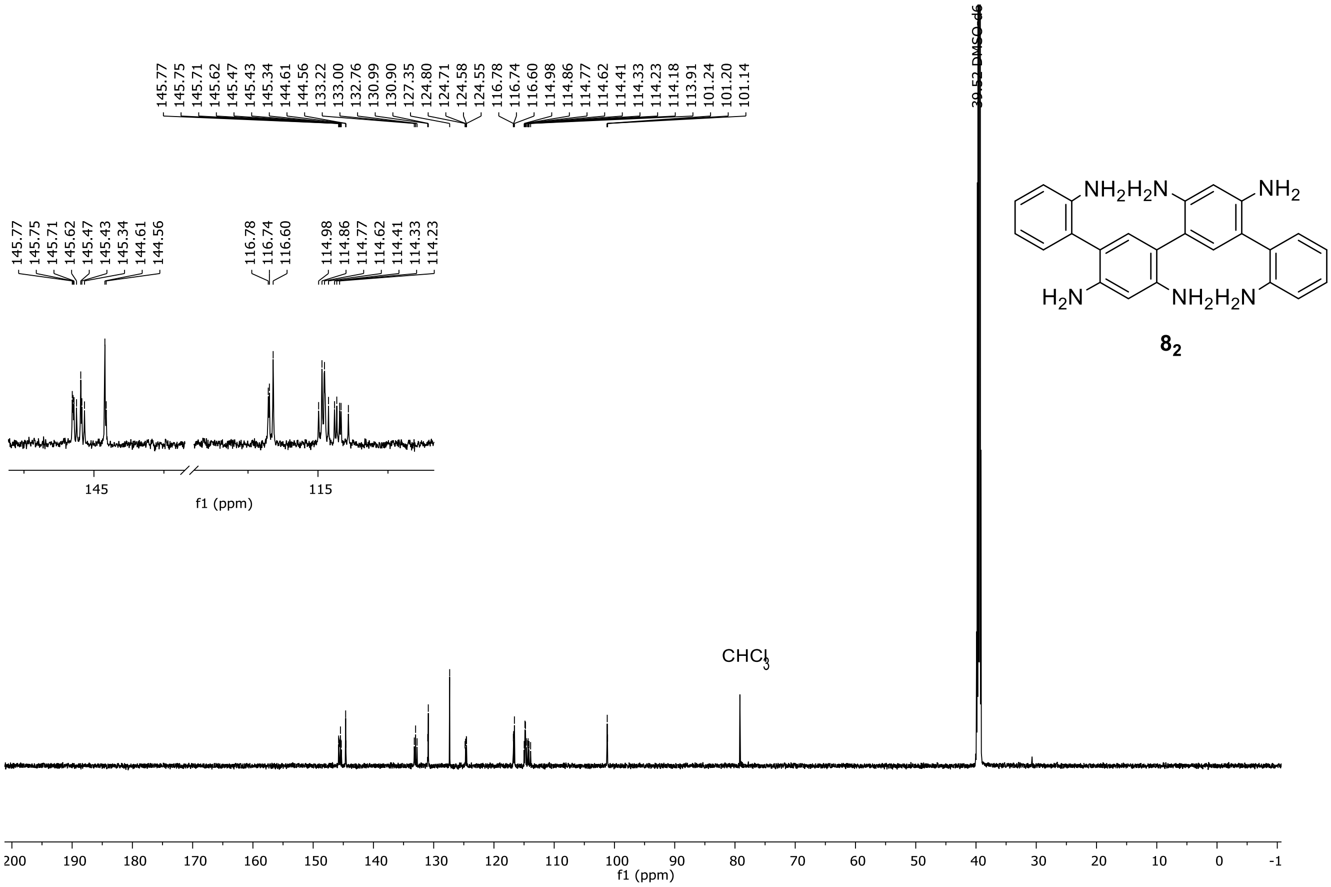

Figure S84. ^{13}C NMR (176 MHz, DMSO-d_6) spectrum of **8_2**.

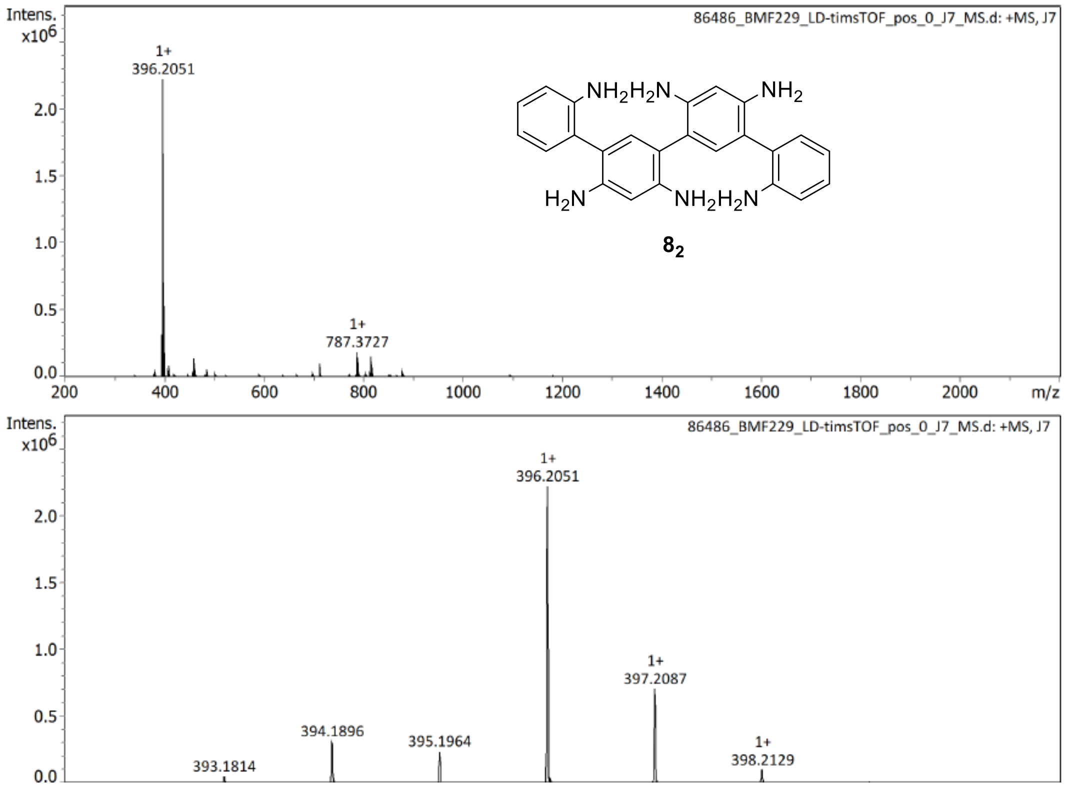

Figure S85. HRMS (LD-timsTOF) spectrum of **8_2**.

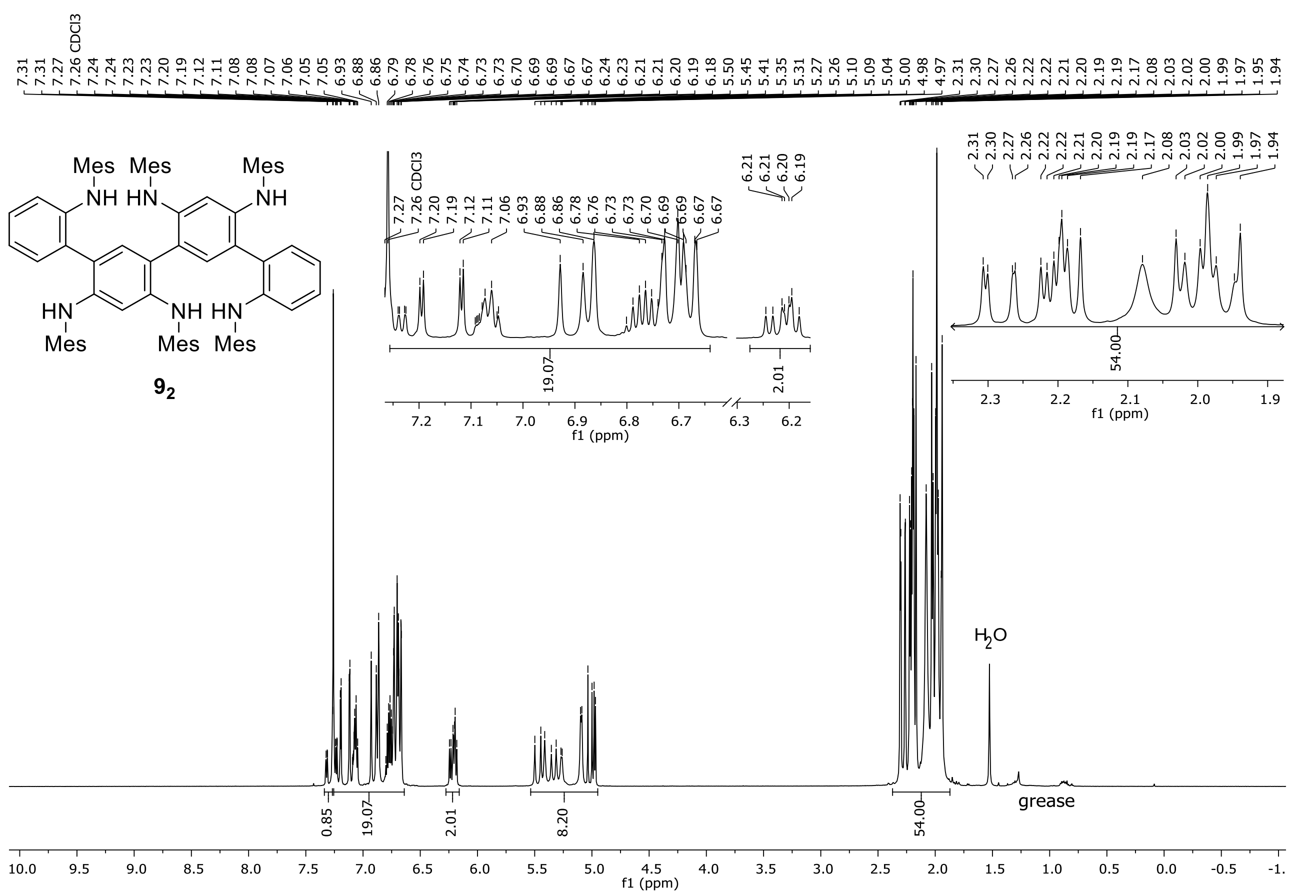

Figure S86. ^{1}H NMR (600 MHz, $CDCl_3$) spectrum of **9$_2$**.

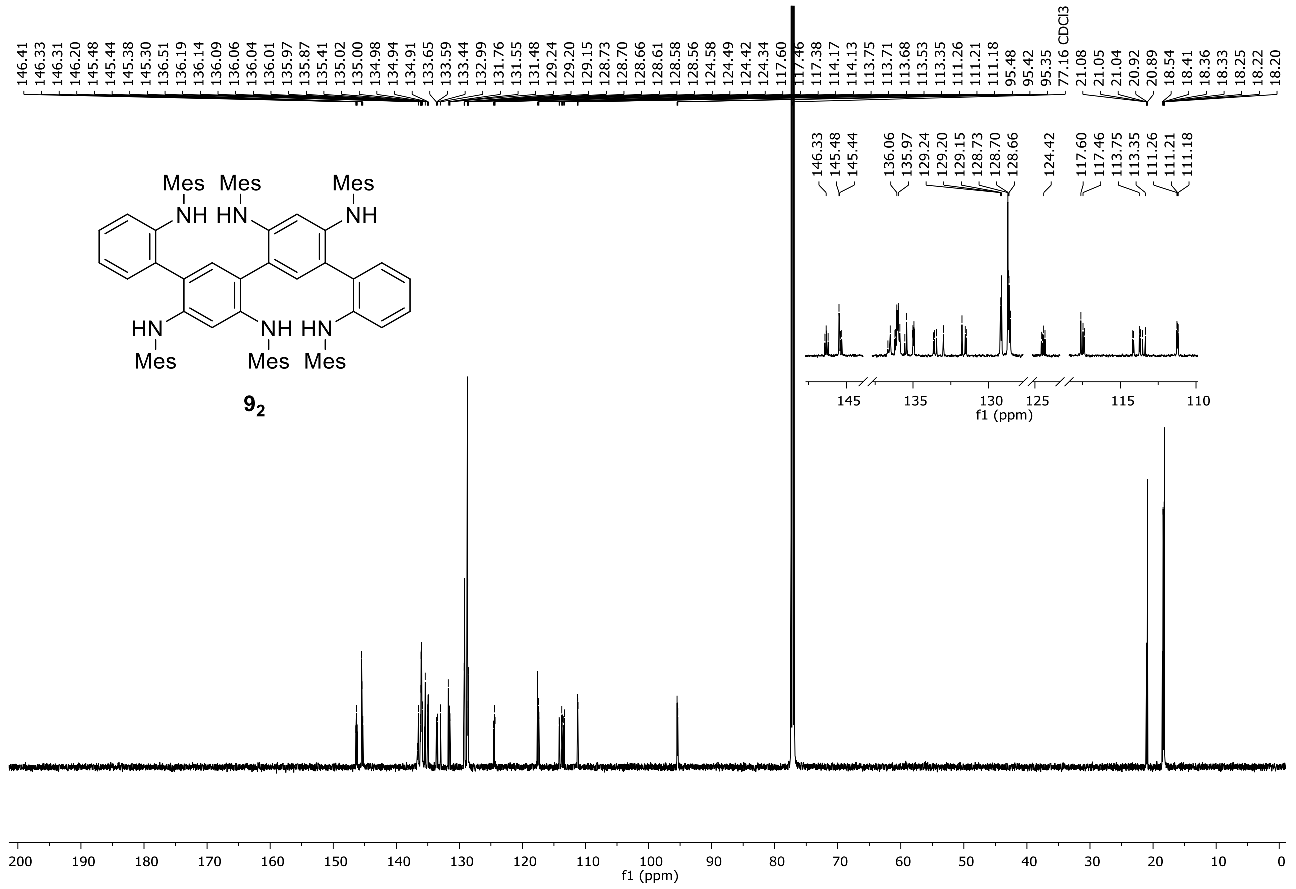

Figure S87. ^{13}C NMR (151 MHz, $CDCl_3$) spectrum of **9$_2$**.

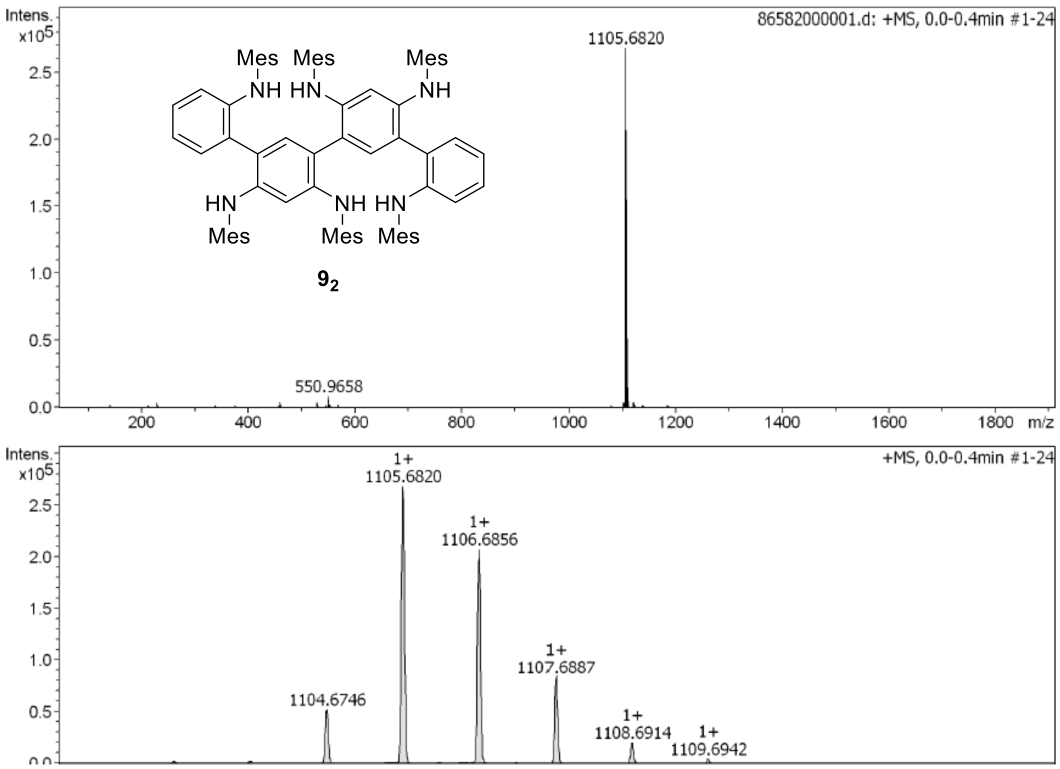

Figure S88. HRMS (ESI) spectrum of $\mathbf{9_2}$.

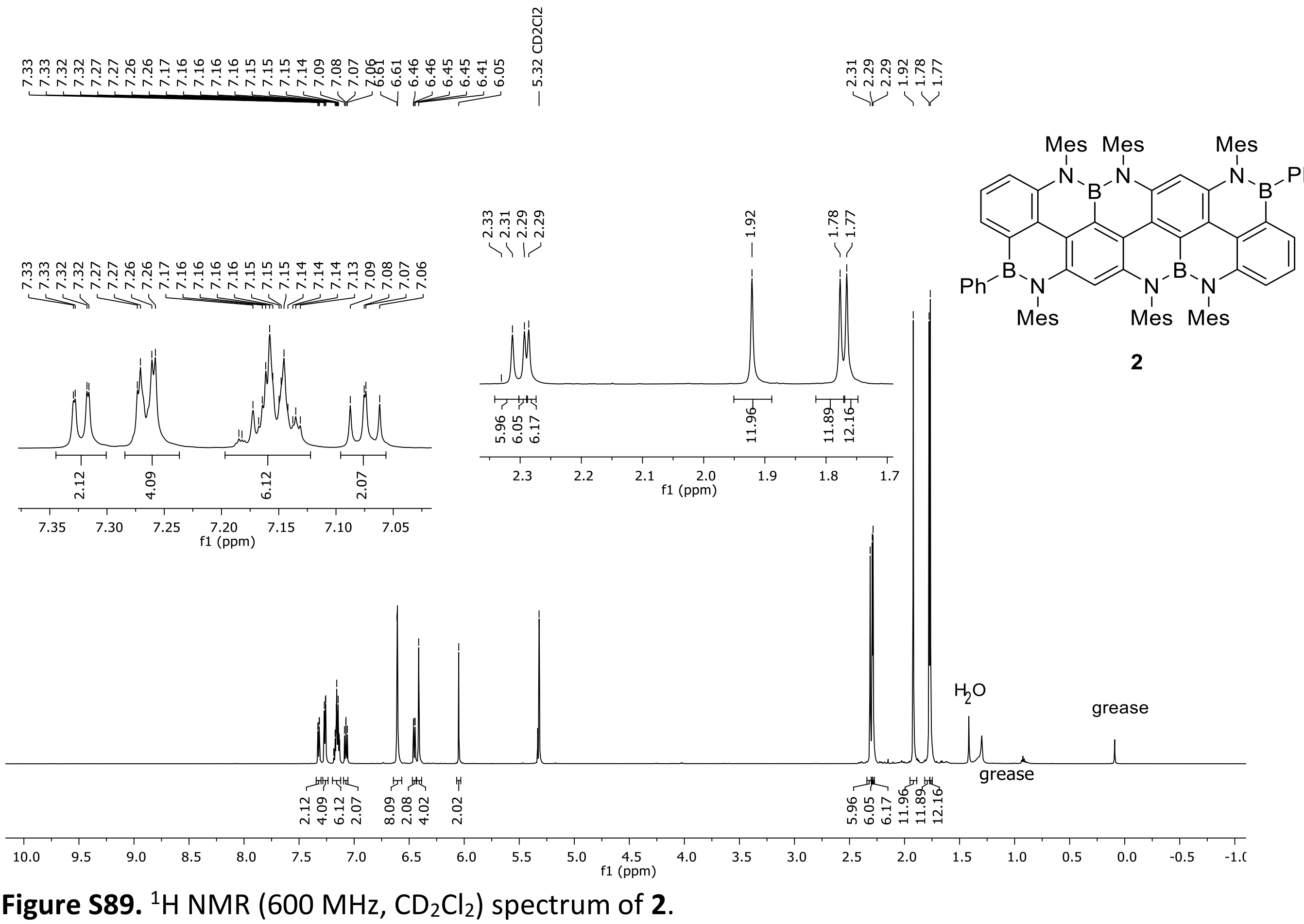

Figure S89. ^{1}H NMR (600 MHz, CD_2Cl_2) spectrum of **2**.

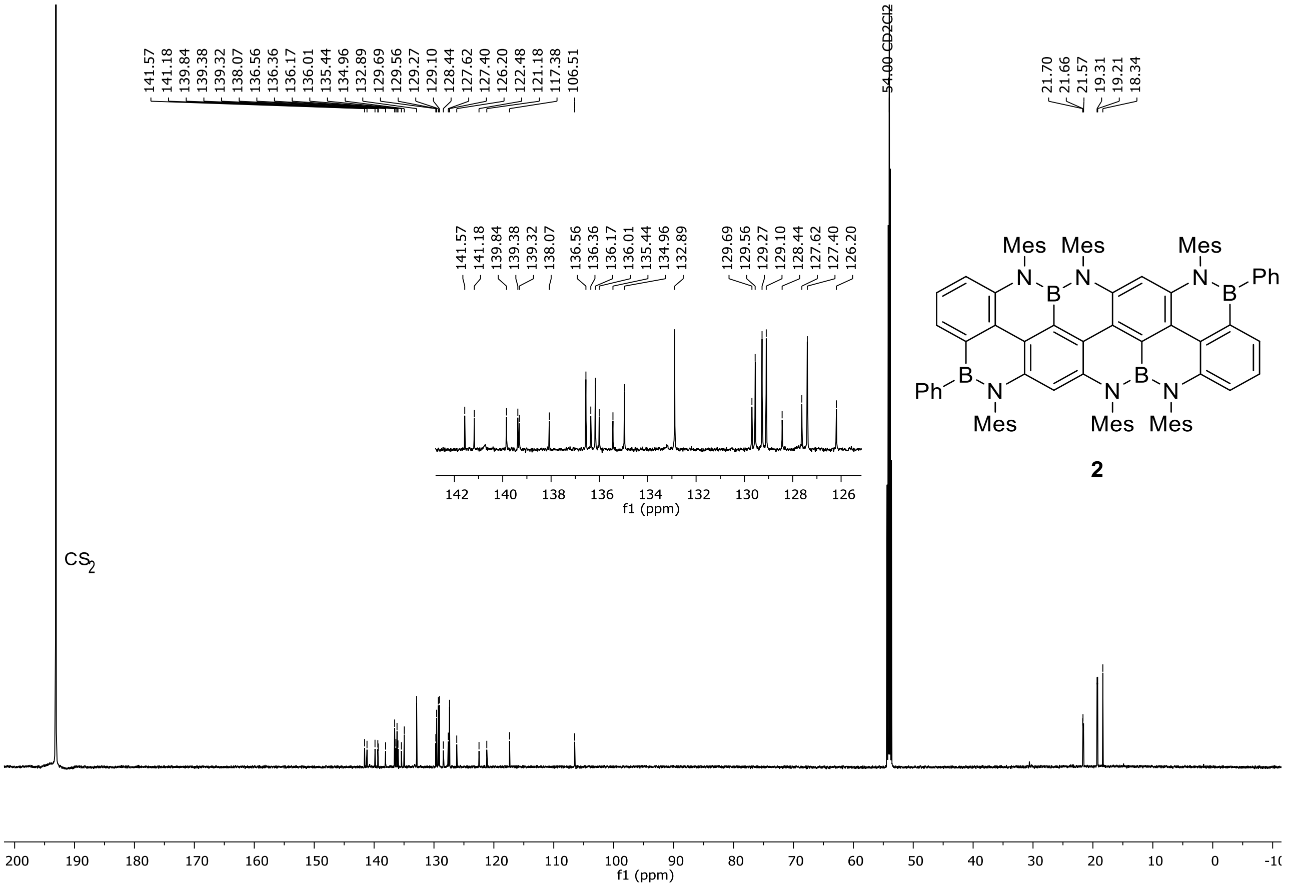

Figure S90. ^{13}C NMR (151 MHz, CD_2Cl_2) spectrum of **2**.

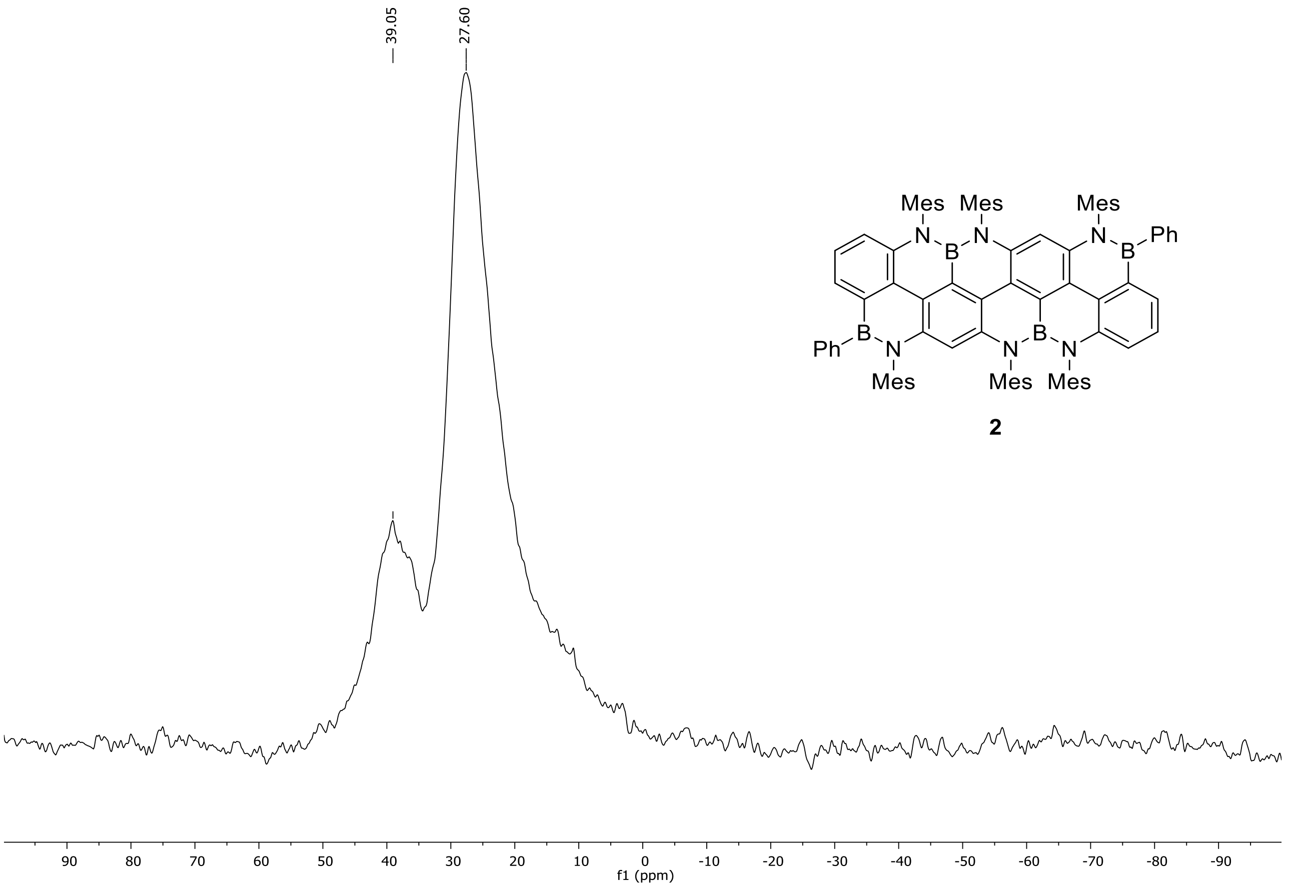

Figure S91. ^{11}B NMR (193 MHz, $CDCl_3$) spectrum of **2**.

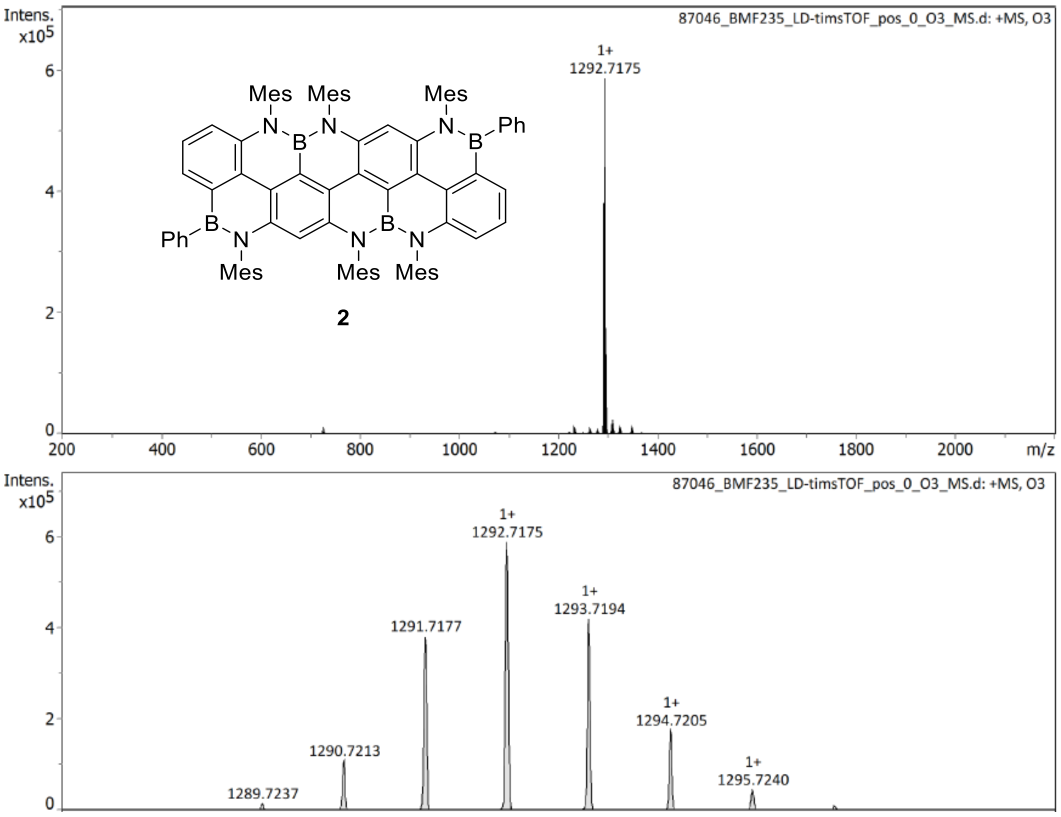

Figure S92. HRMS (LD-timsTOF) spectrum of **2**.

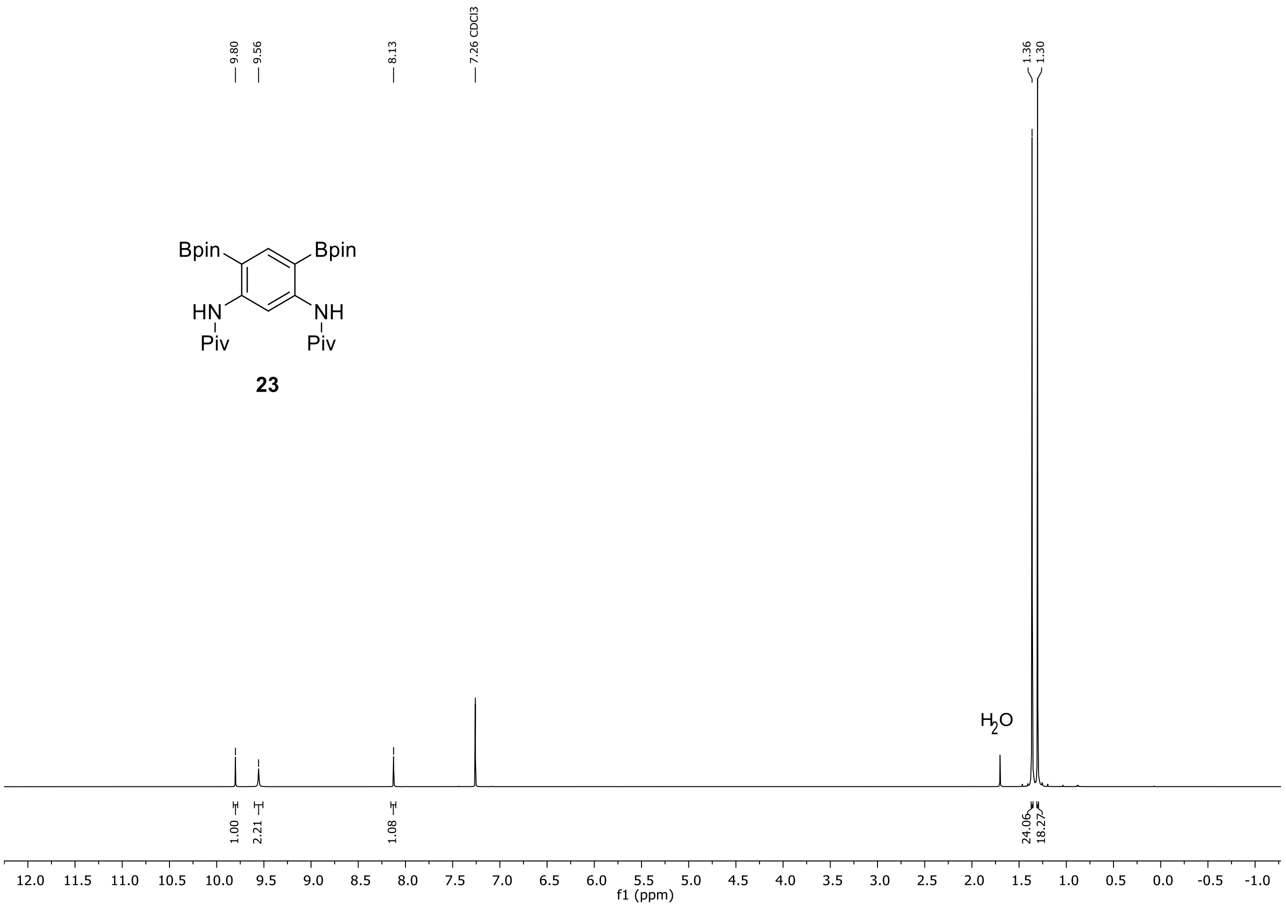

Figure S93. ^{1}H NMR (600 MHz, $CDCl_3$) spectrum of **23**.

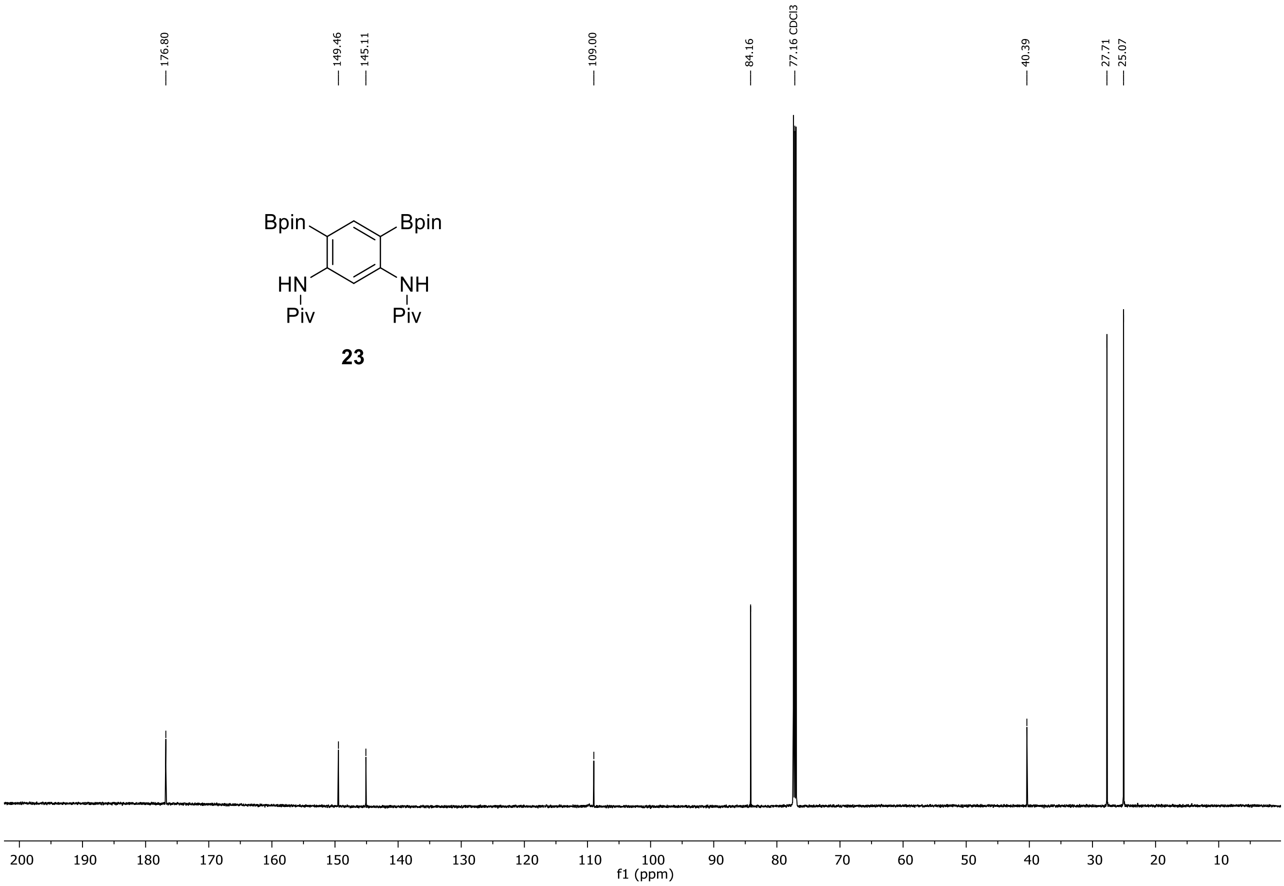

Figure S94. ^{13}C NMR (151 MHz, $CDCl_3$) spectrum of **23**.

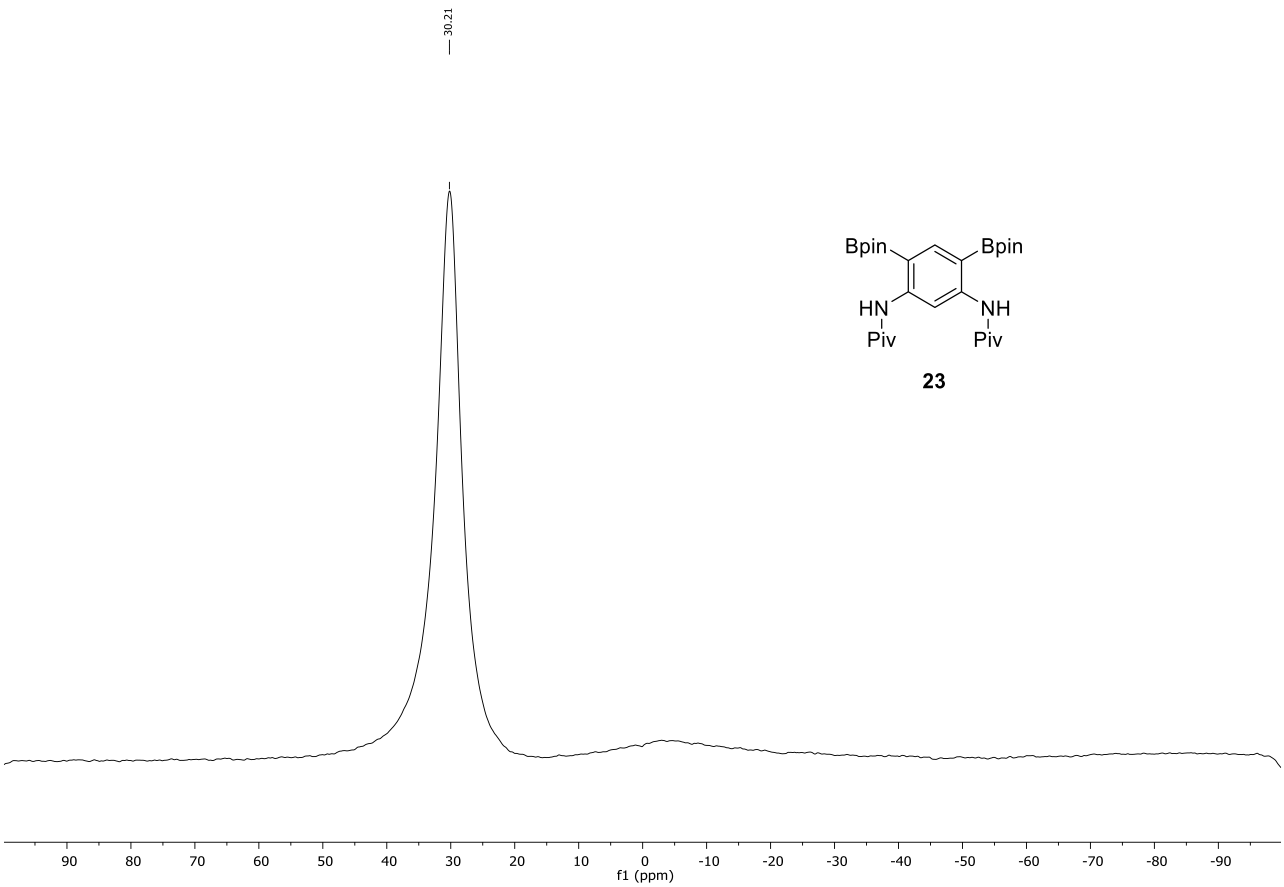

Figure S95. ^{11}B NMR (193 MHz, $CDCl_3$) spectrum of **23**.

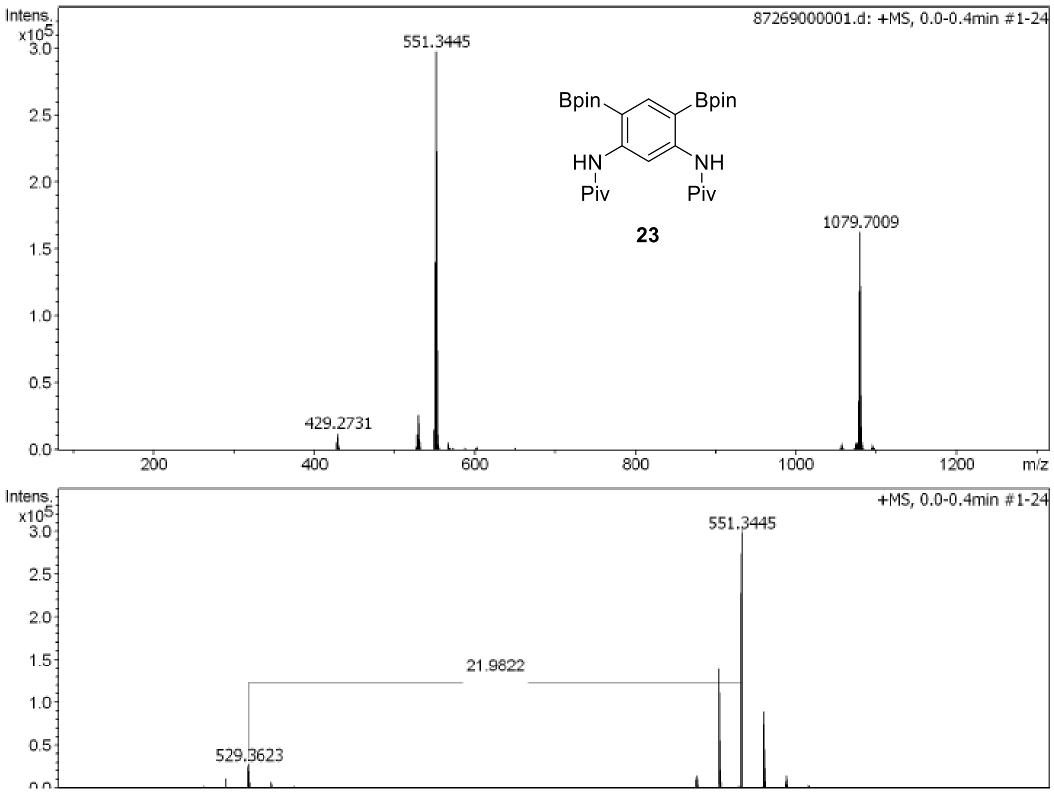

Figure S96. HRMS (ESI) spectrum of **23**.

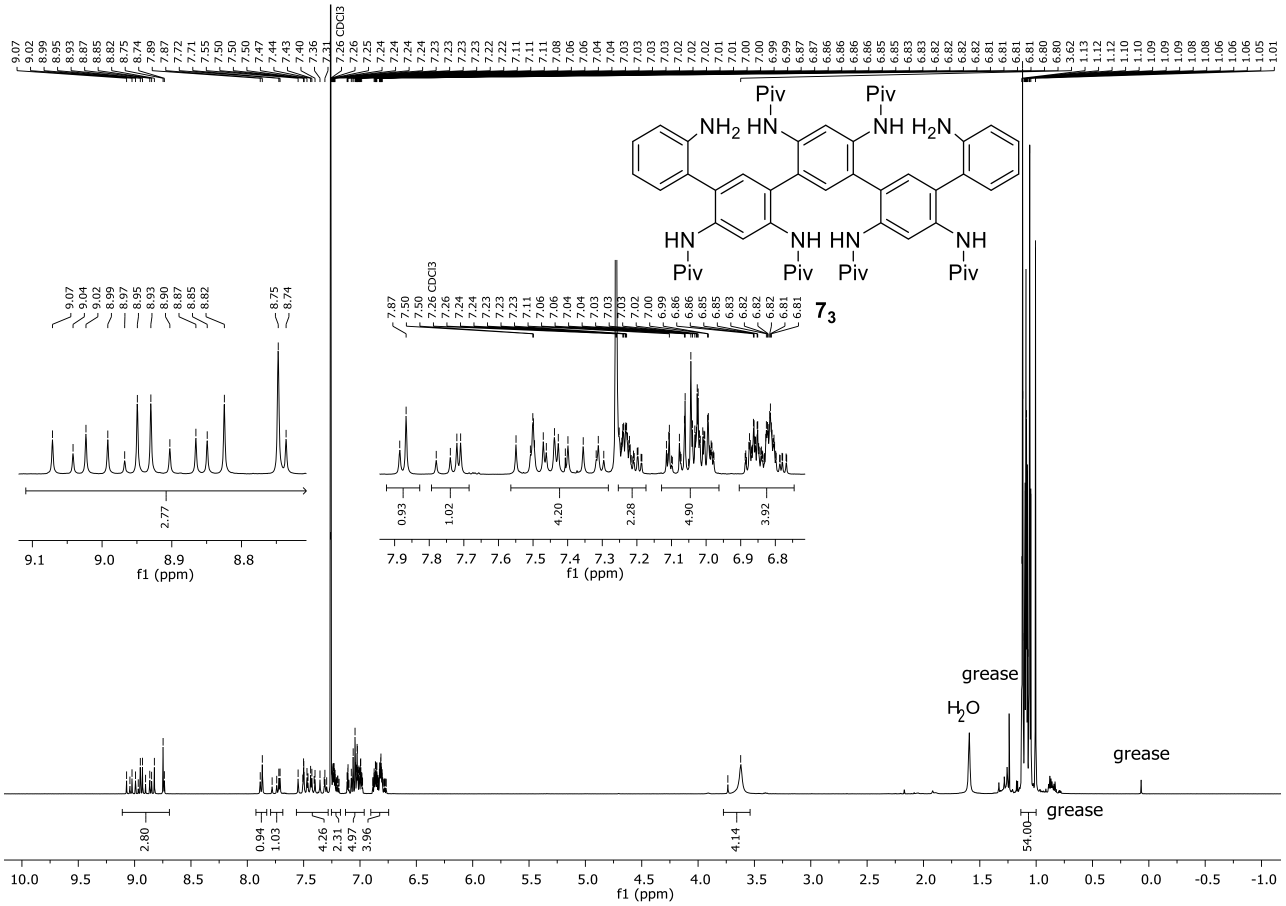

Figure S97. ^{1}H NMR (700 MHz, $CDCl_3$) spectrum of **7_3**.

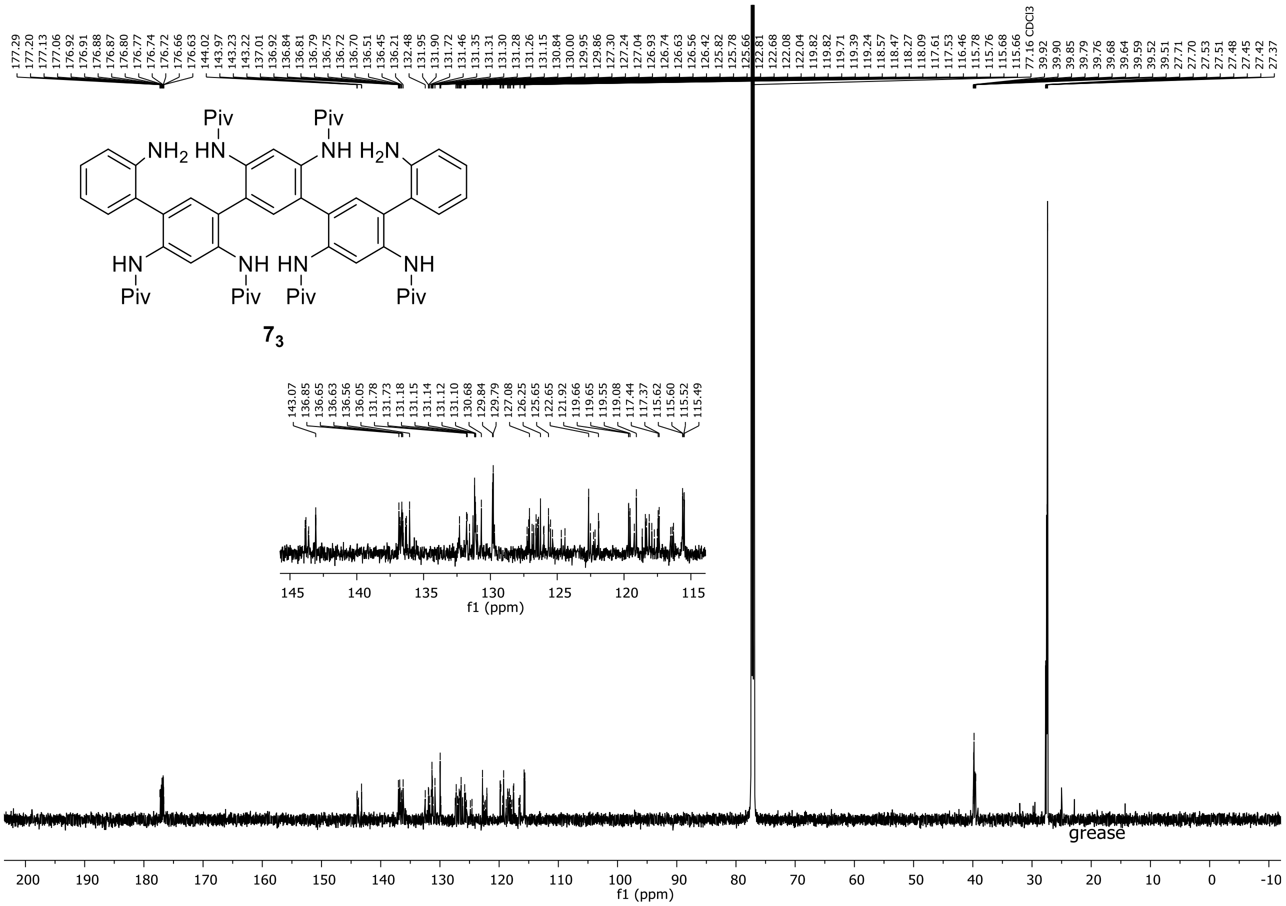

Figure S98. ^{13}C NMR (176 MHz, $CDCl_3$) spectrum of **7_3**.

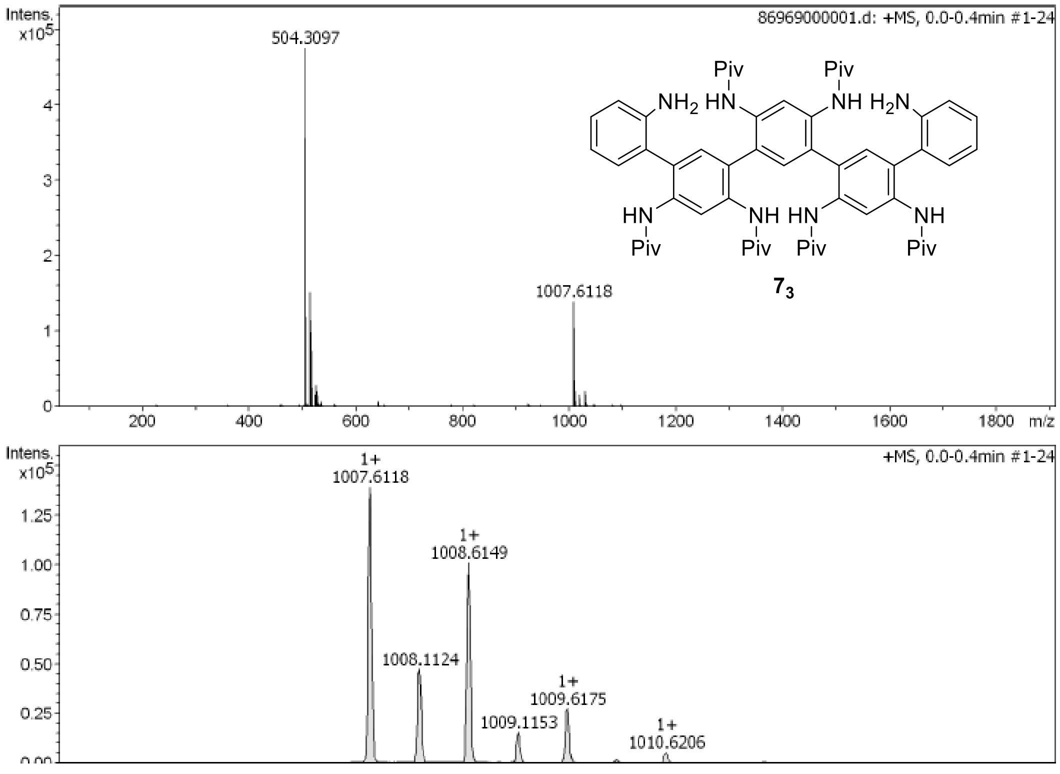

Figure S99. HRMS (ESI) spectrum of **7_3**.

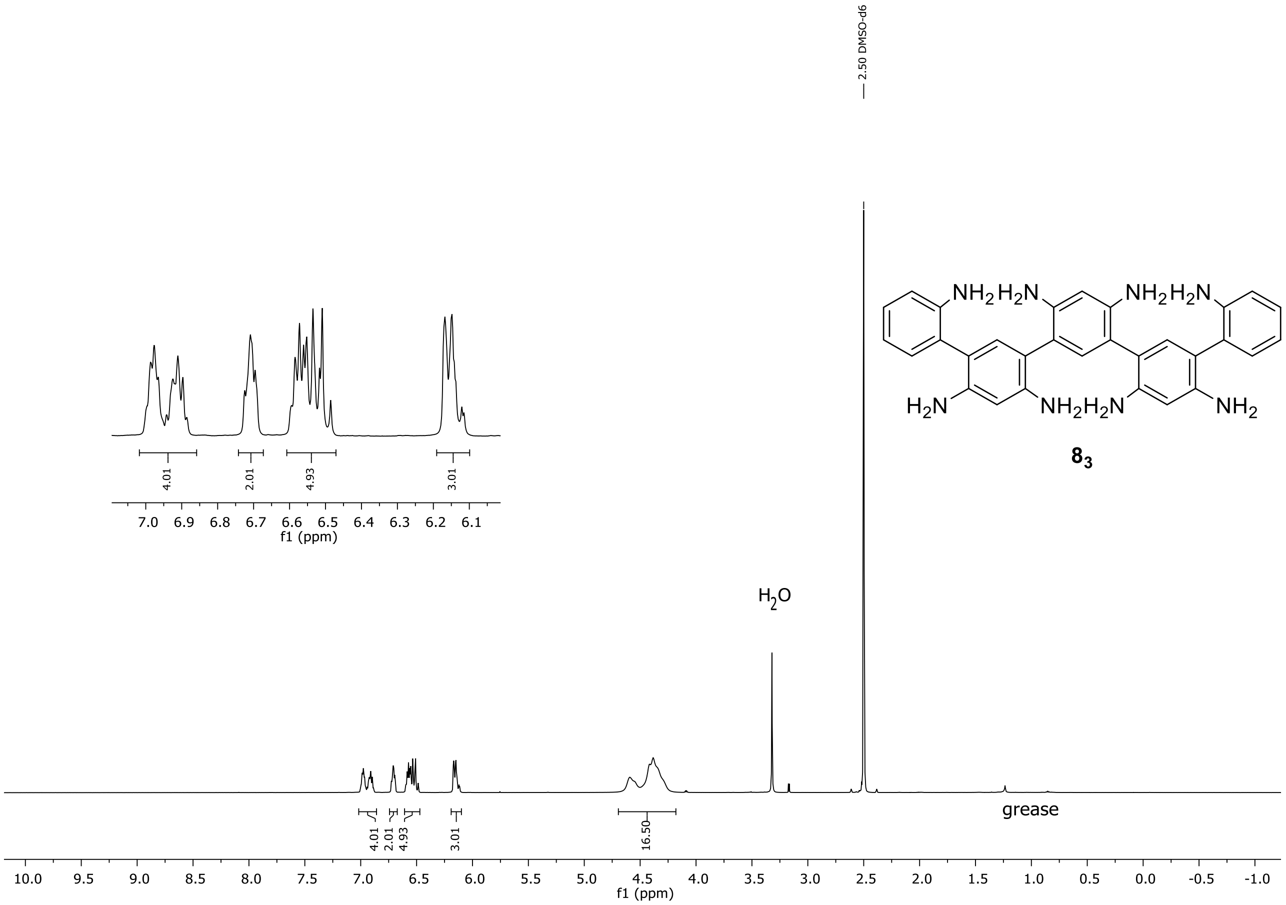

Figure S100. ^{1}H NMR (600 MHz, DMSO-d_6) spectrum of **8$_3$**.

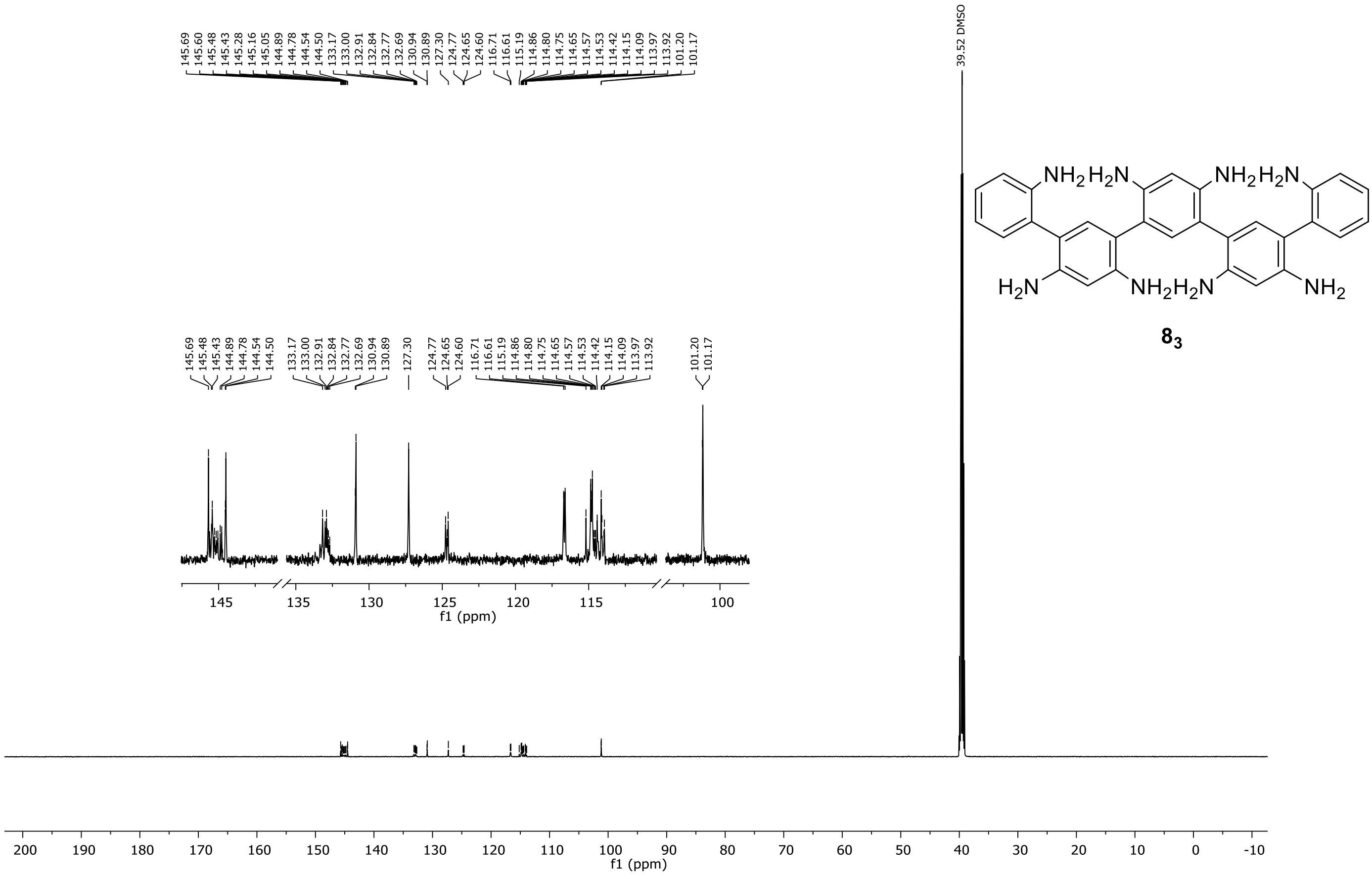

Figure S101. ^{13}C NMR (151 MHz, DMSO-d_6) spectrum of **8$_3$**.

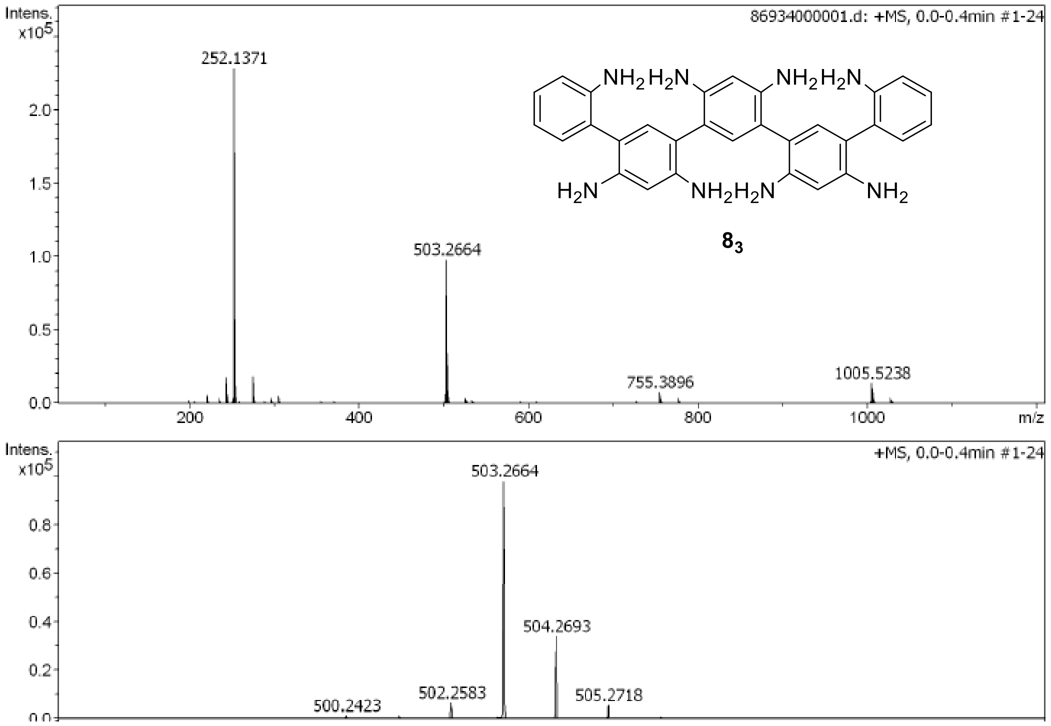

Figure S102. HRMS (ESI) spectrum of **8_3**.

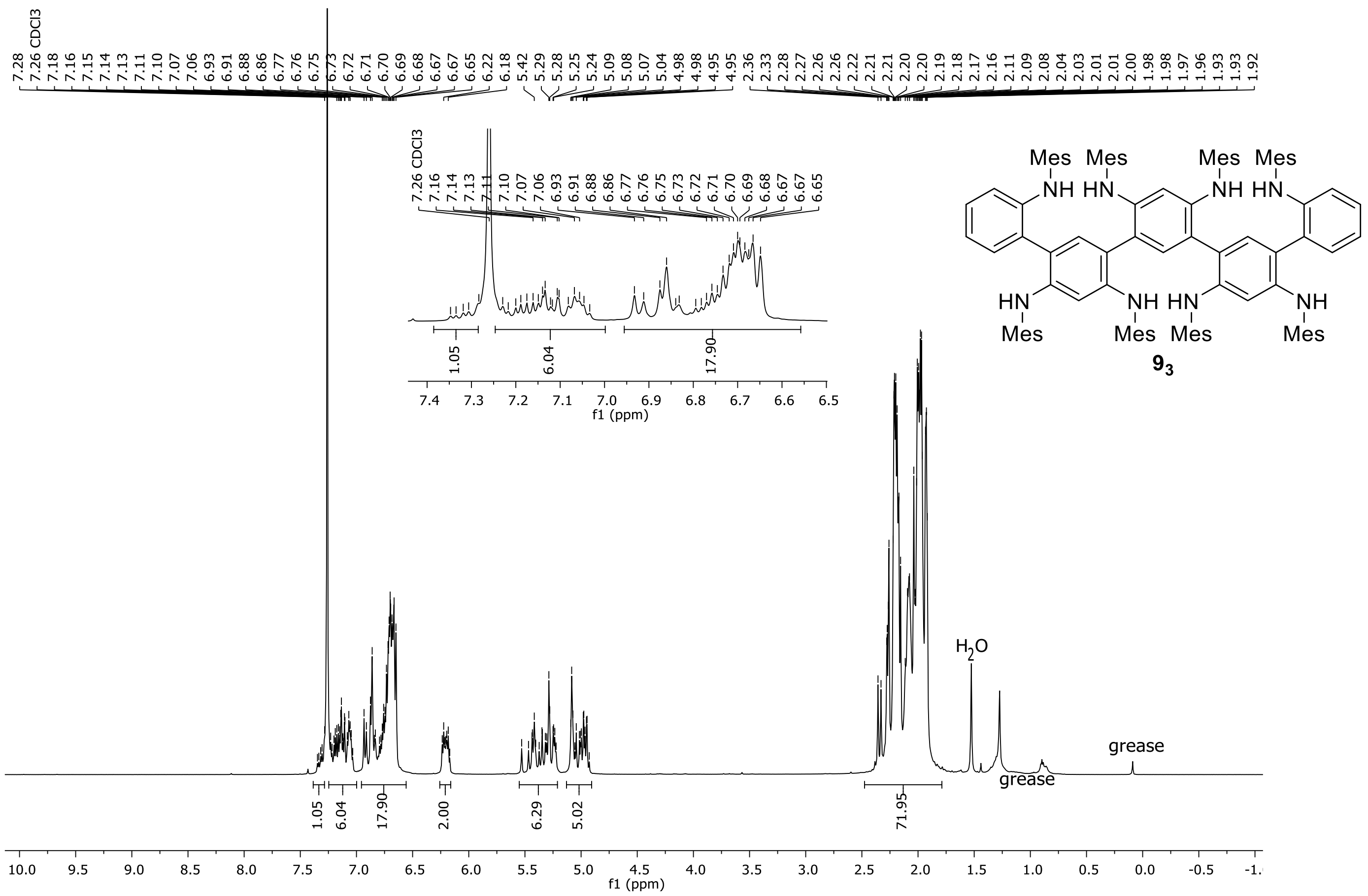

Figure S103. ^{1}H NMR (600 MHz, $CDCl_3$) spectrum of **9_3**.

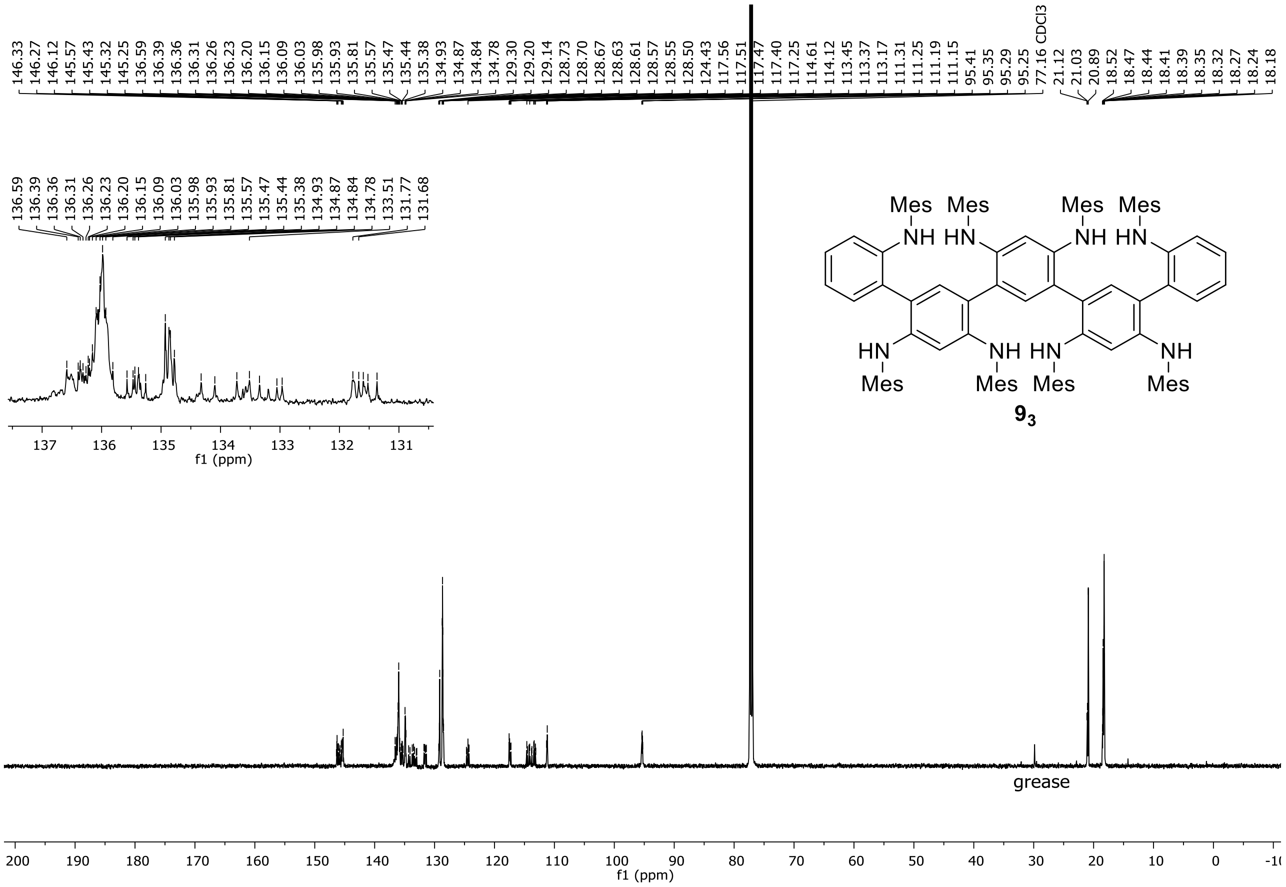

Figure S104. ^{13}C NMR (151 MHz, $CDCl_3$) spectrum of **9_3**.

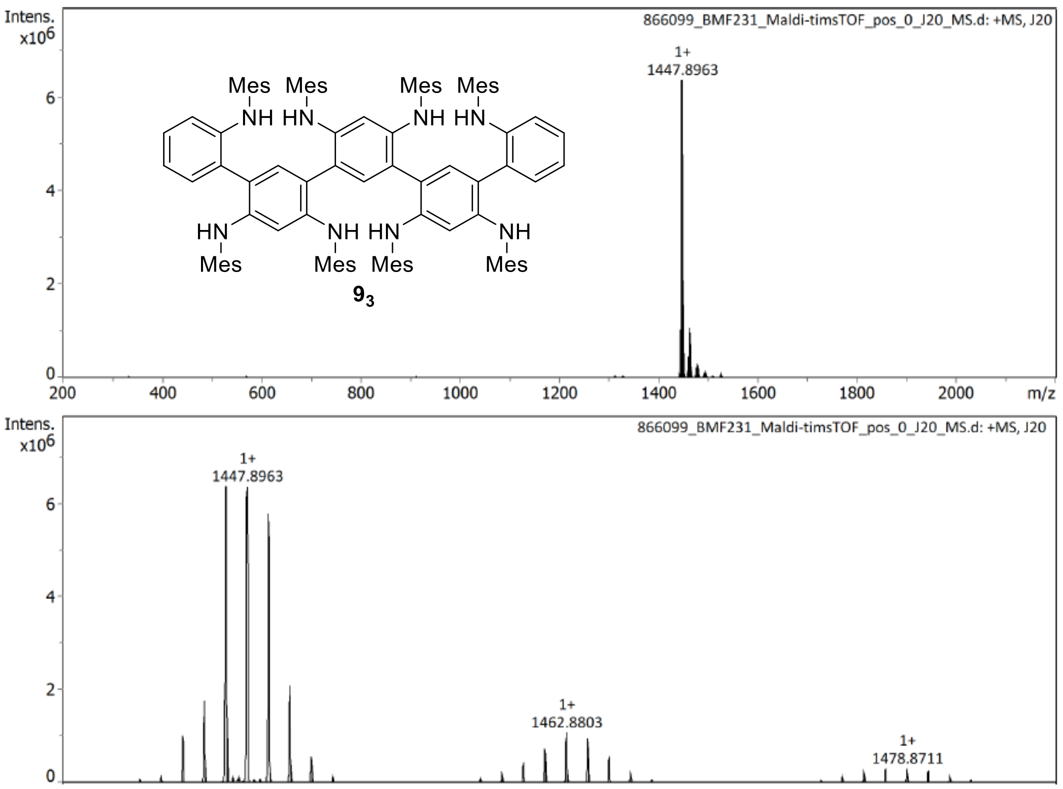

Figure S105. HRMS (MALDI-timsTOF, matrix: DCTB) spectrum of **9_3**.

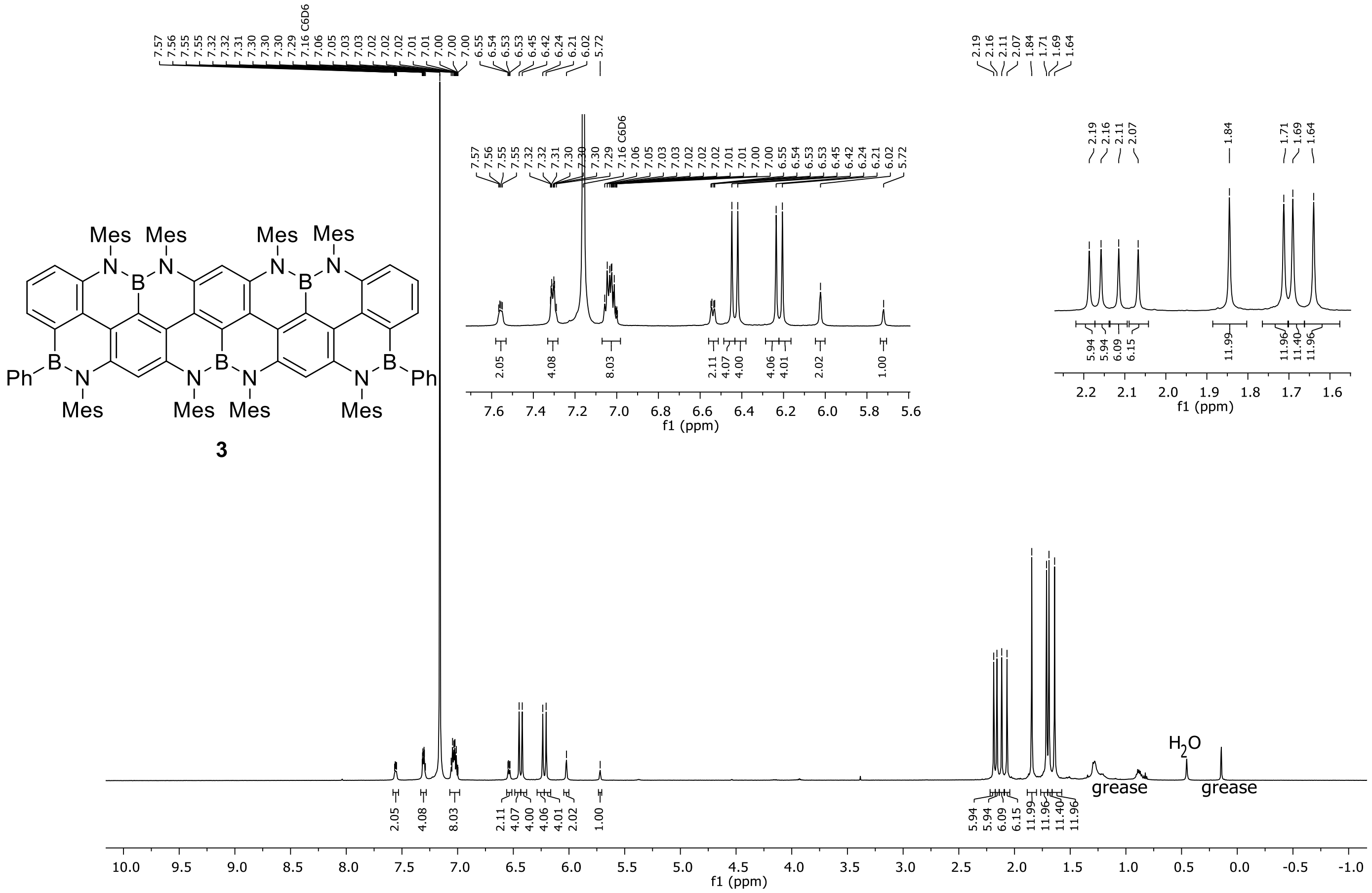

Figure S106. ^{1}H NMR (600 MHz, C_6D_6/CS_2 1:1) spectrum of **3**.

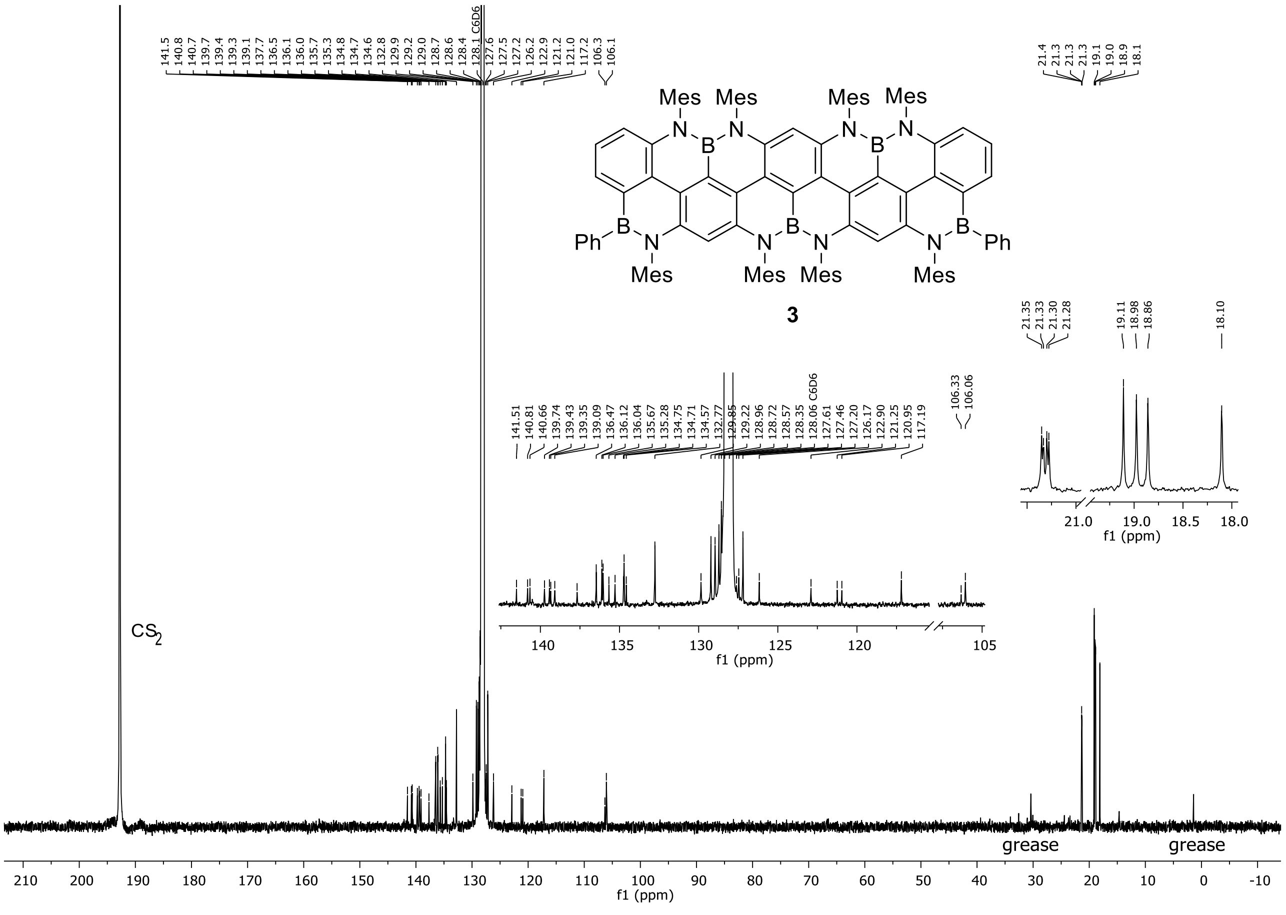

Figure S107. ^{13}C NMR (151 MHz, C_6D_6/CS_2 1:1) spectrum of **3**.

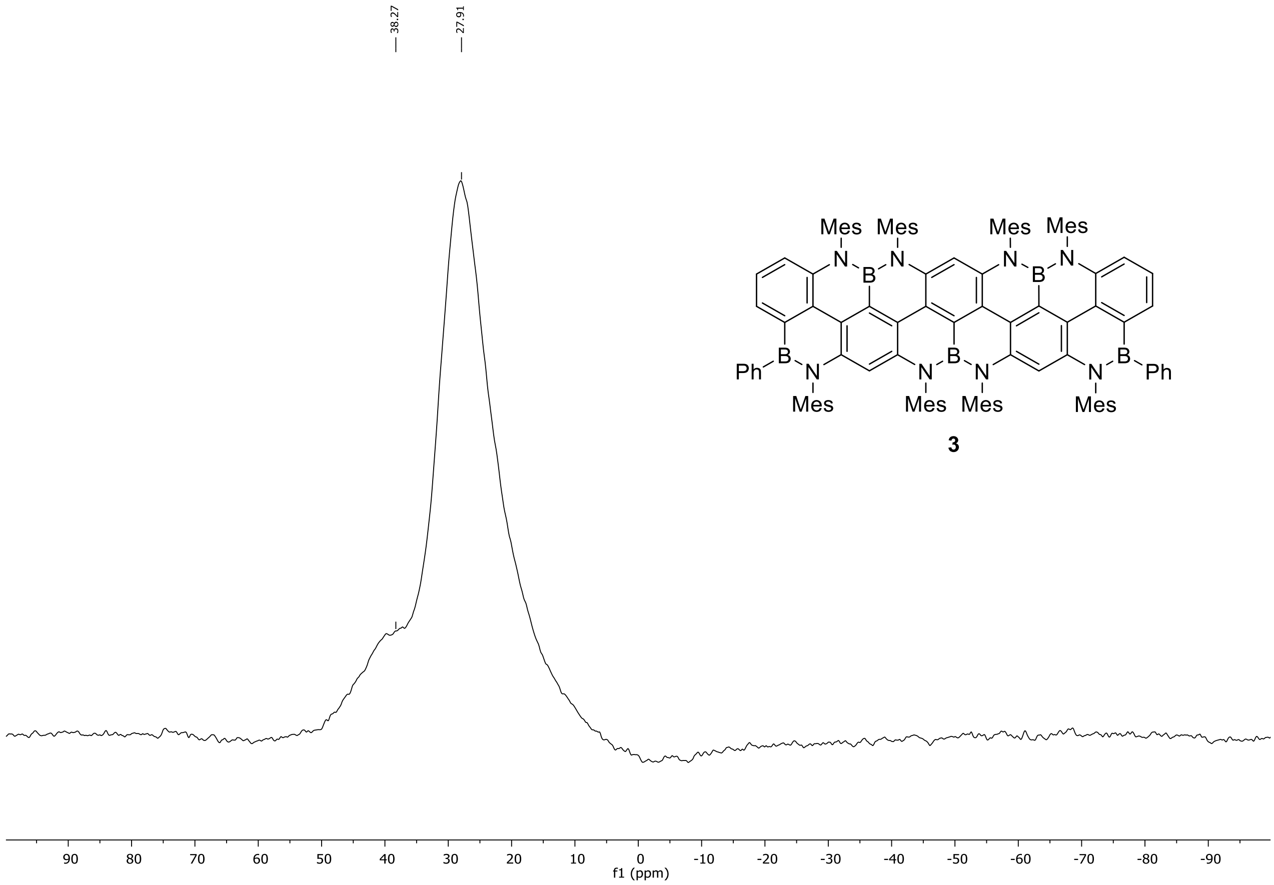

Figure S108. ^{11}B NMR (193 MHz, C_6D_6/CS_2 1:1) spectrum of **3**.

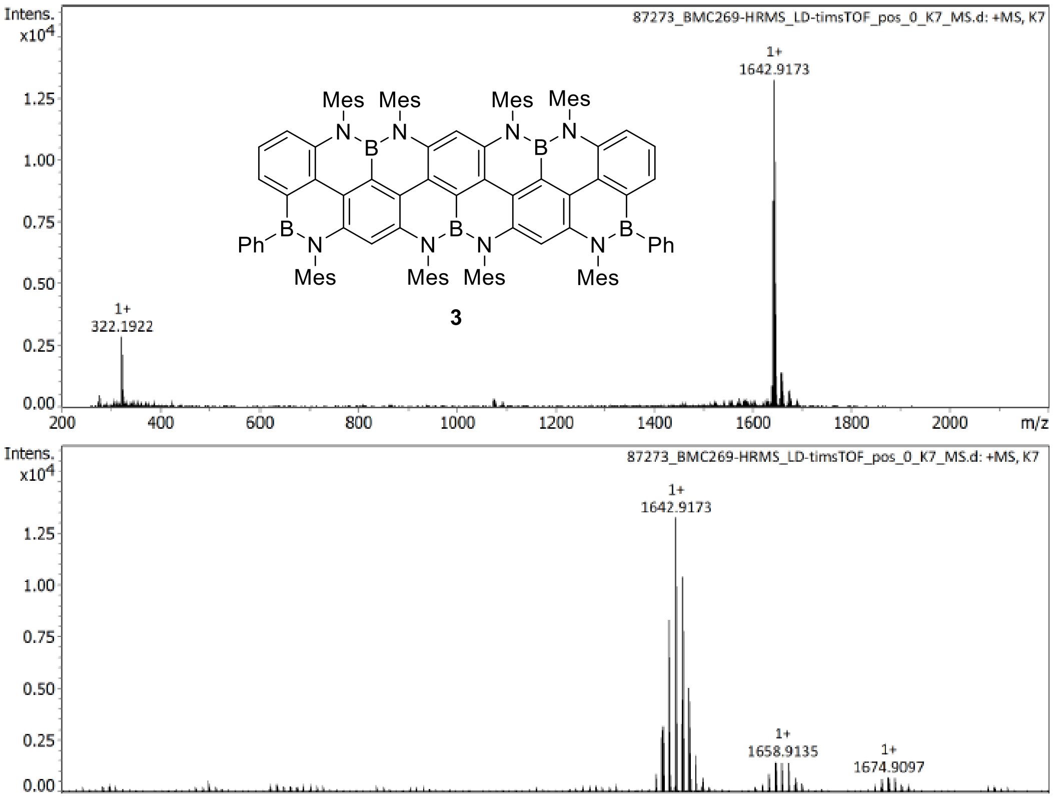

Figure S109. HRMS (LD-timsTOF) spectrum of **3**.

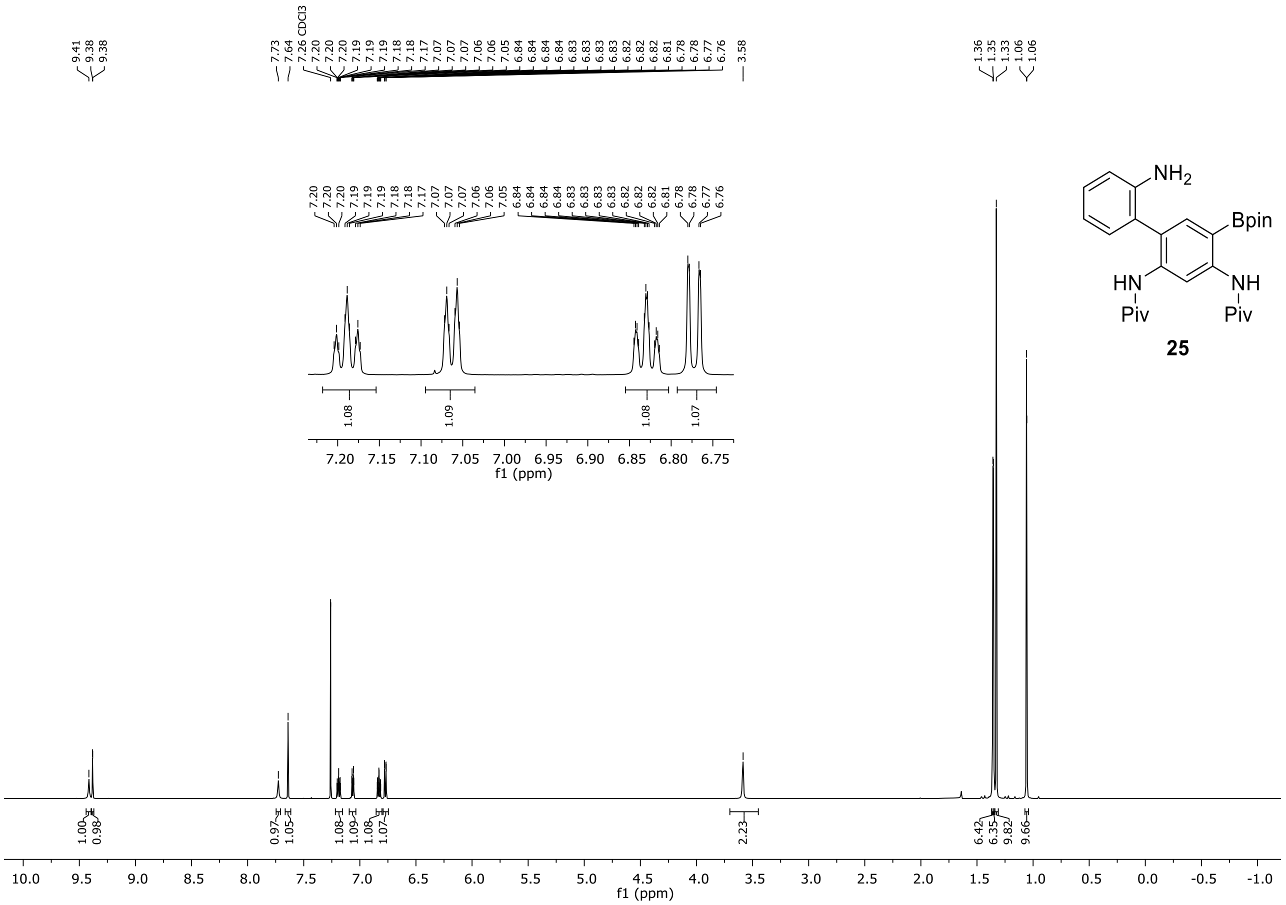

Figure S110. ^{1}H NMR (600 MHz, $CDCl_3$) spectrum of **25**.

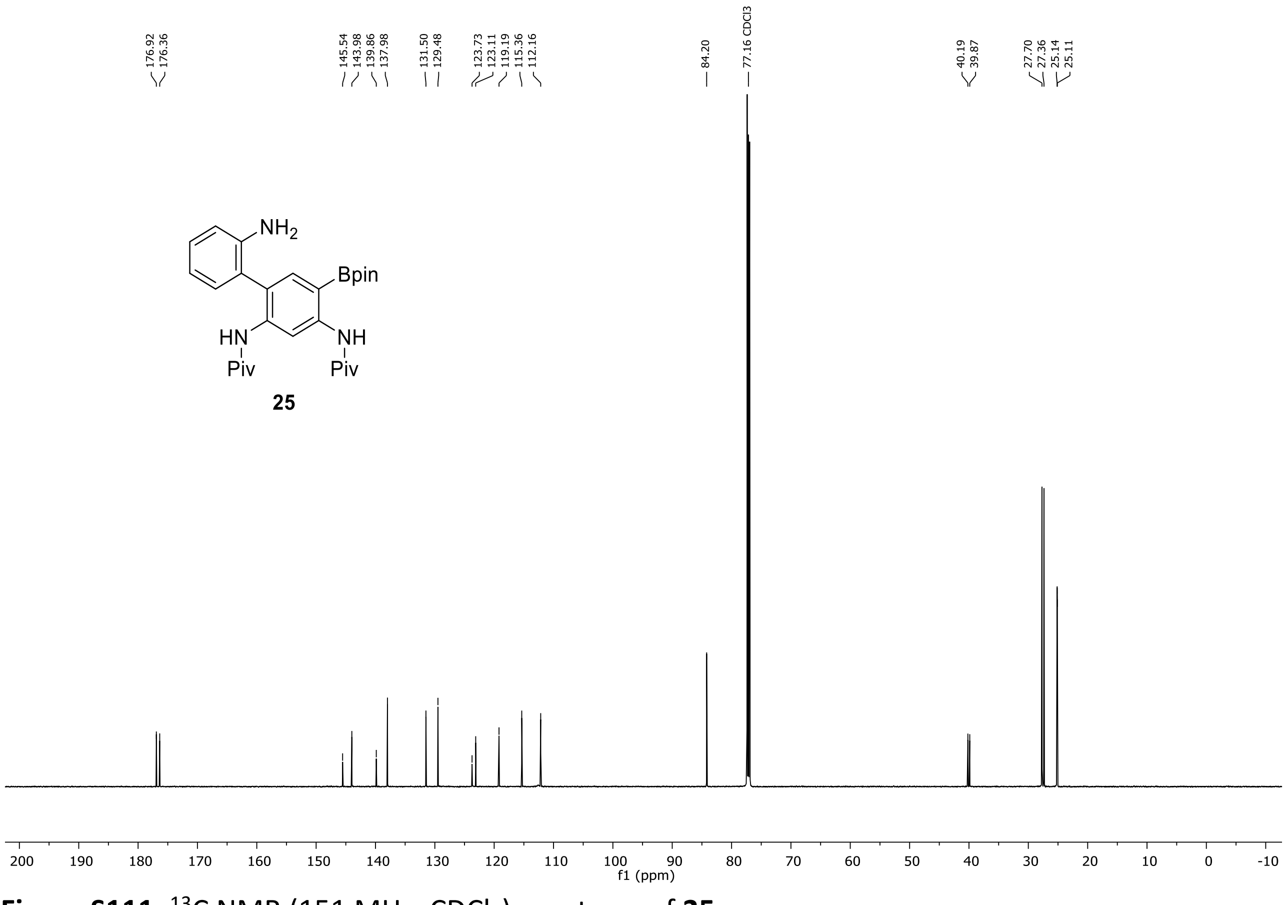

Figure S111. ^{13}C NMR (151 MHz, $CDCl_3$) spectrum of **25**.

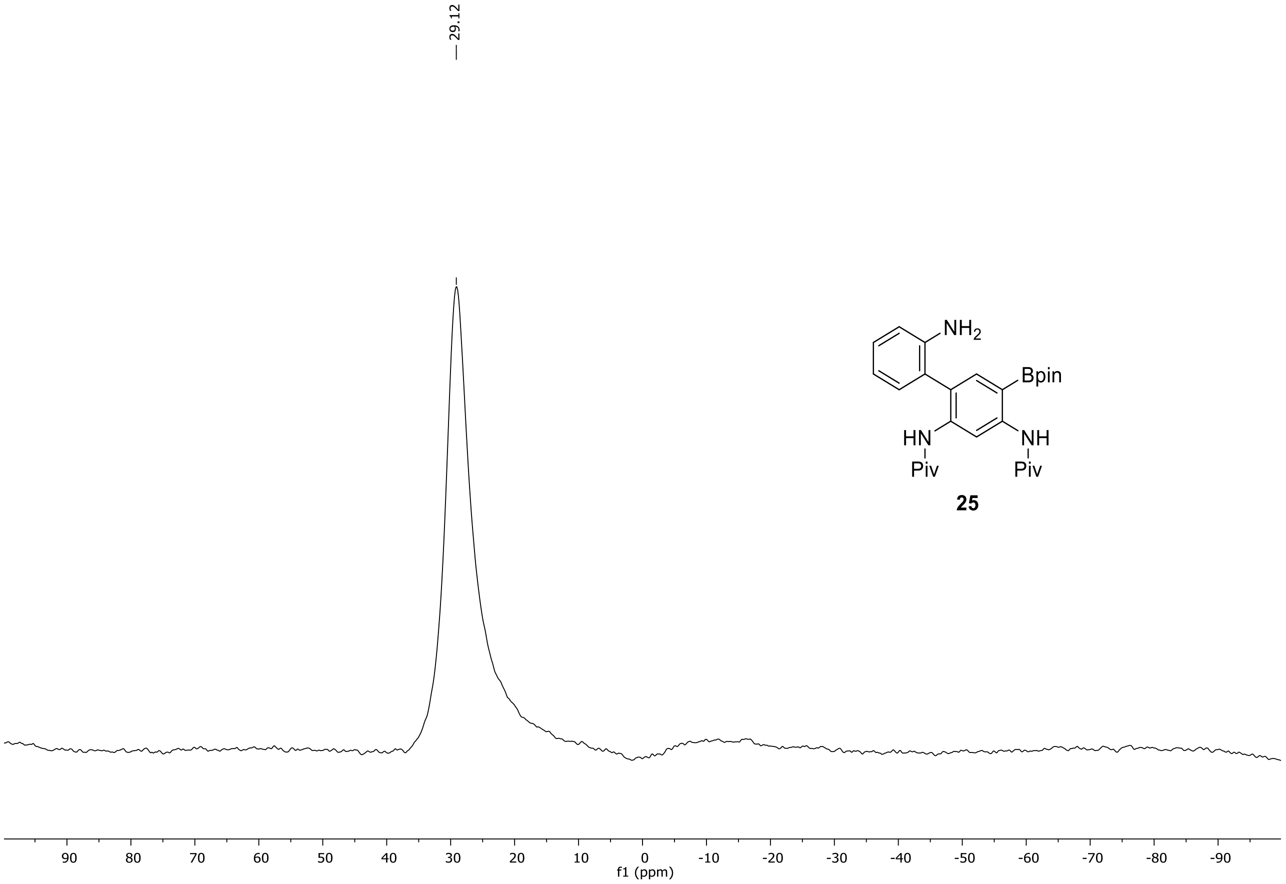

Figure S112. ^{11}B NMR (193 MHz, $CDCl_3$) spectrum of **25**.

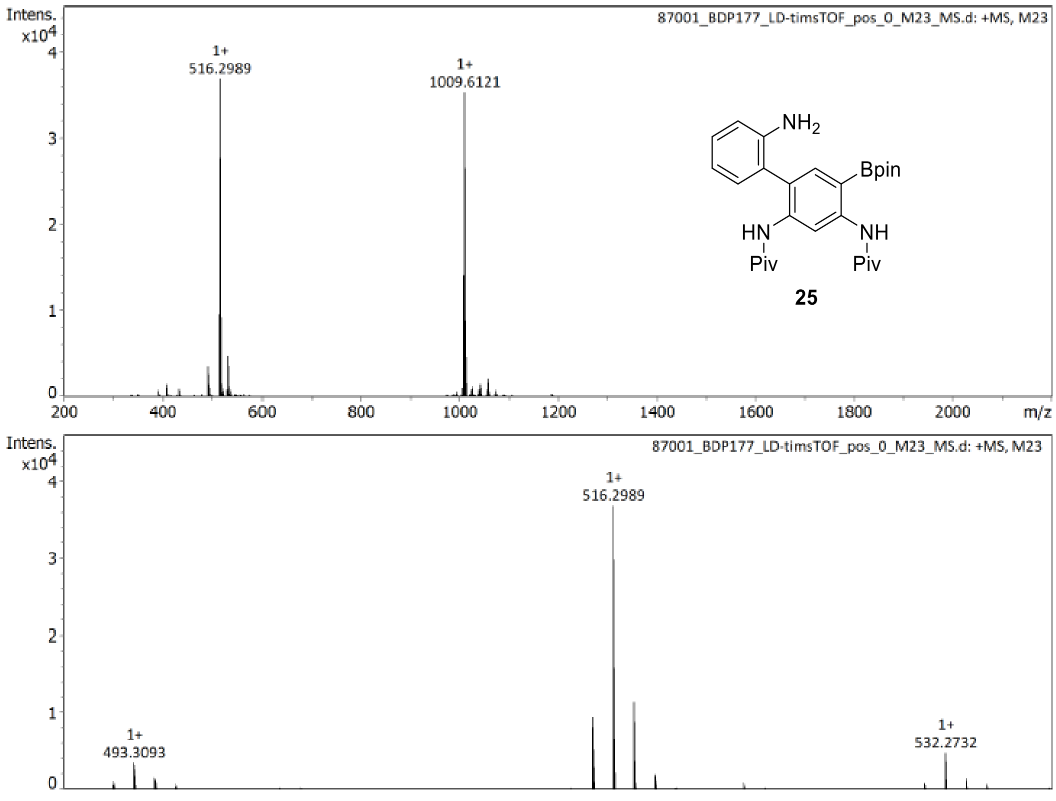

Figure S113. HRMS (LD-timsTOF) spectrum of **25**.

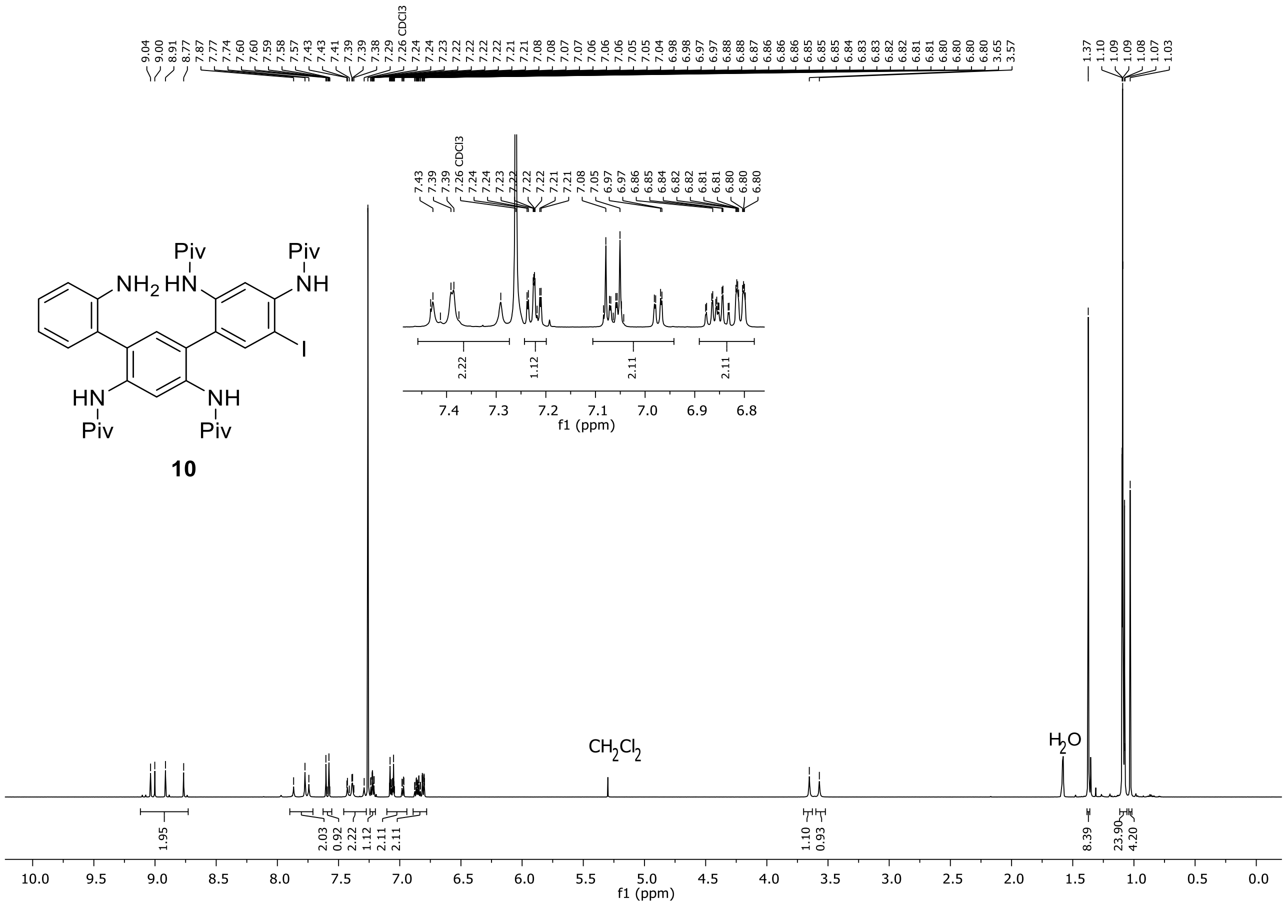

Figure S114. ^{1}H NMR (600 MHz, $CDCl_3$) spectrum of **10**.

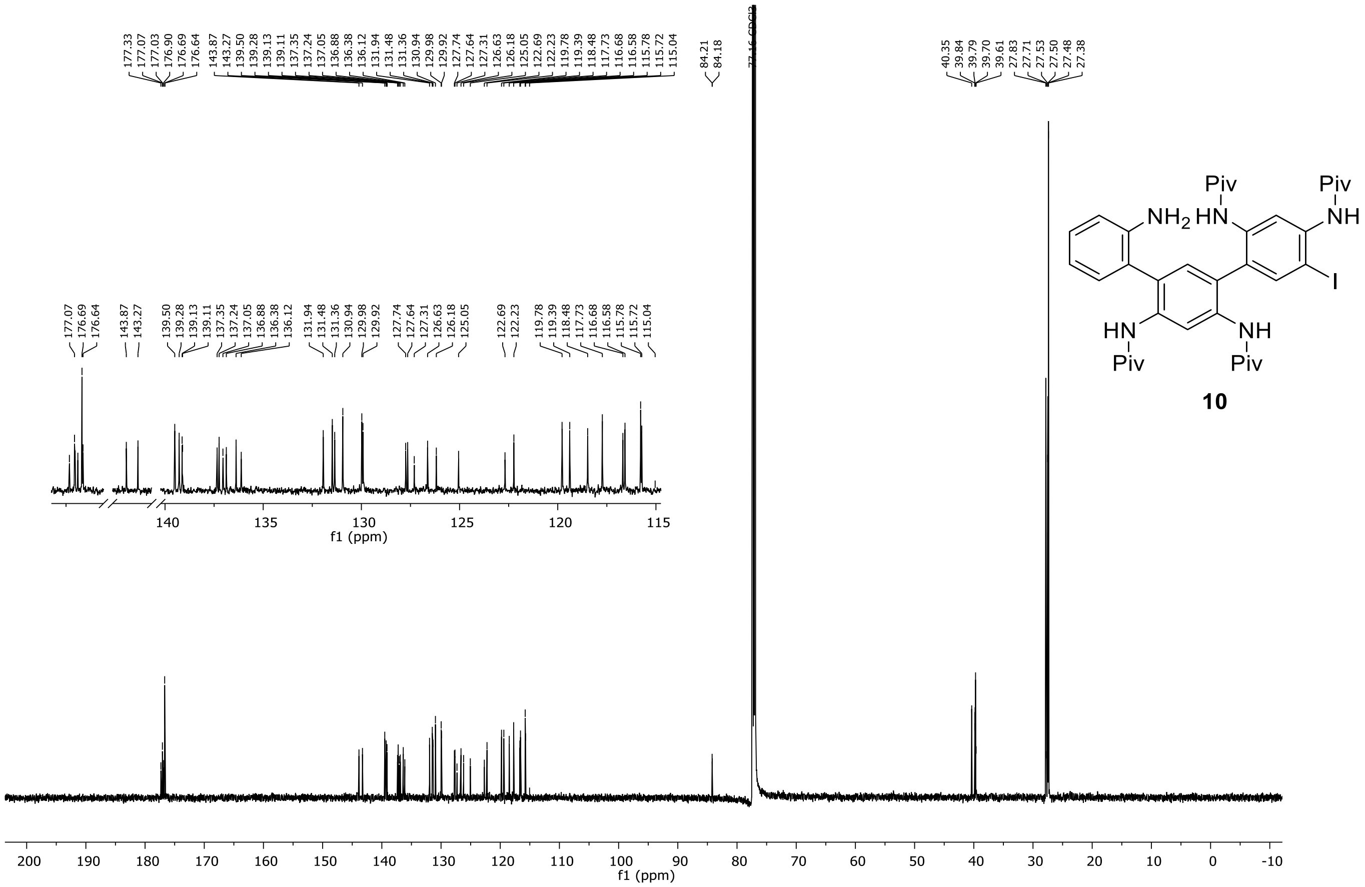

Figure S115. ^{13}C NMR (151 MHz, $CDCl_3$) spectrum of **10**.

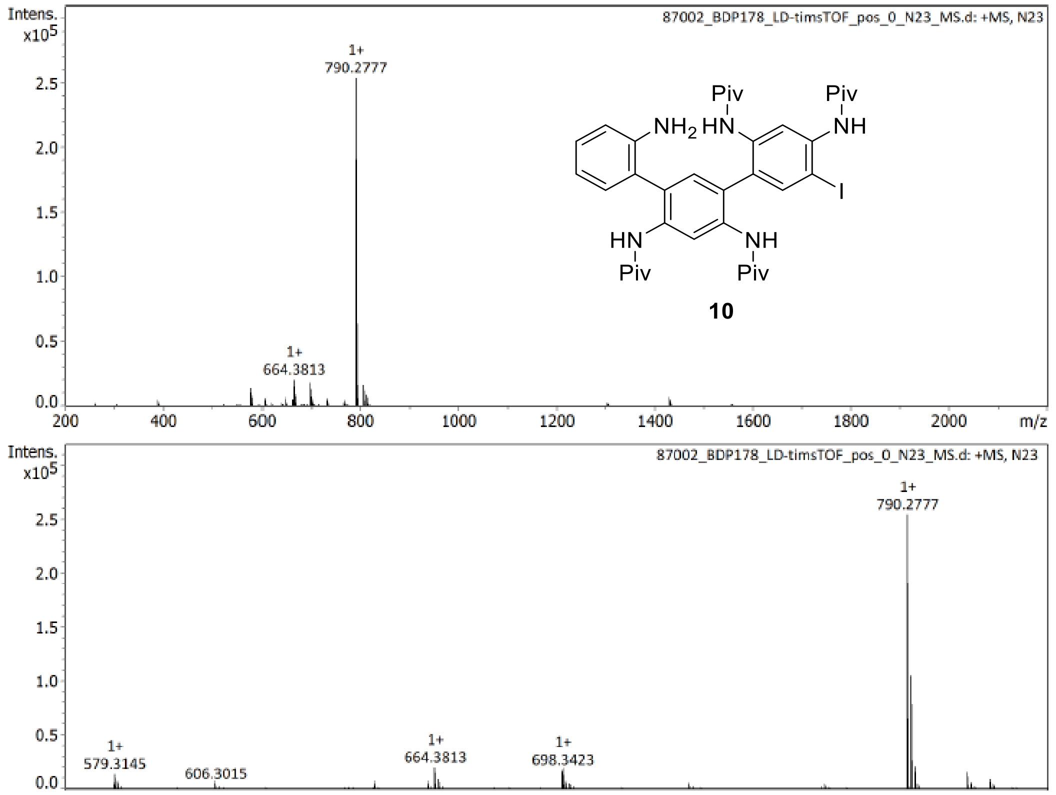

Figure S116. HRMS (LD-timsTOF) spectrum of **10**.

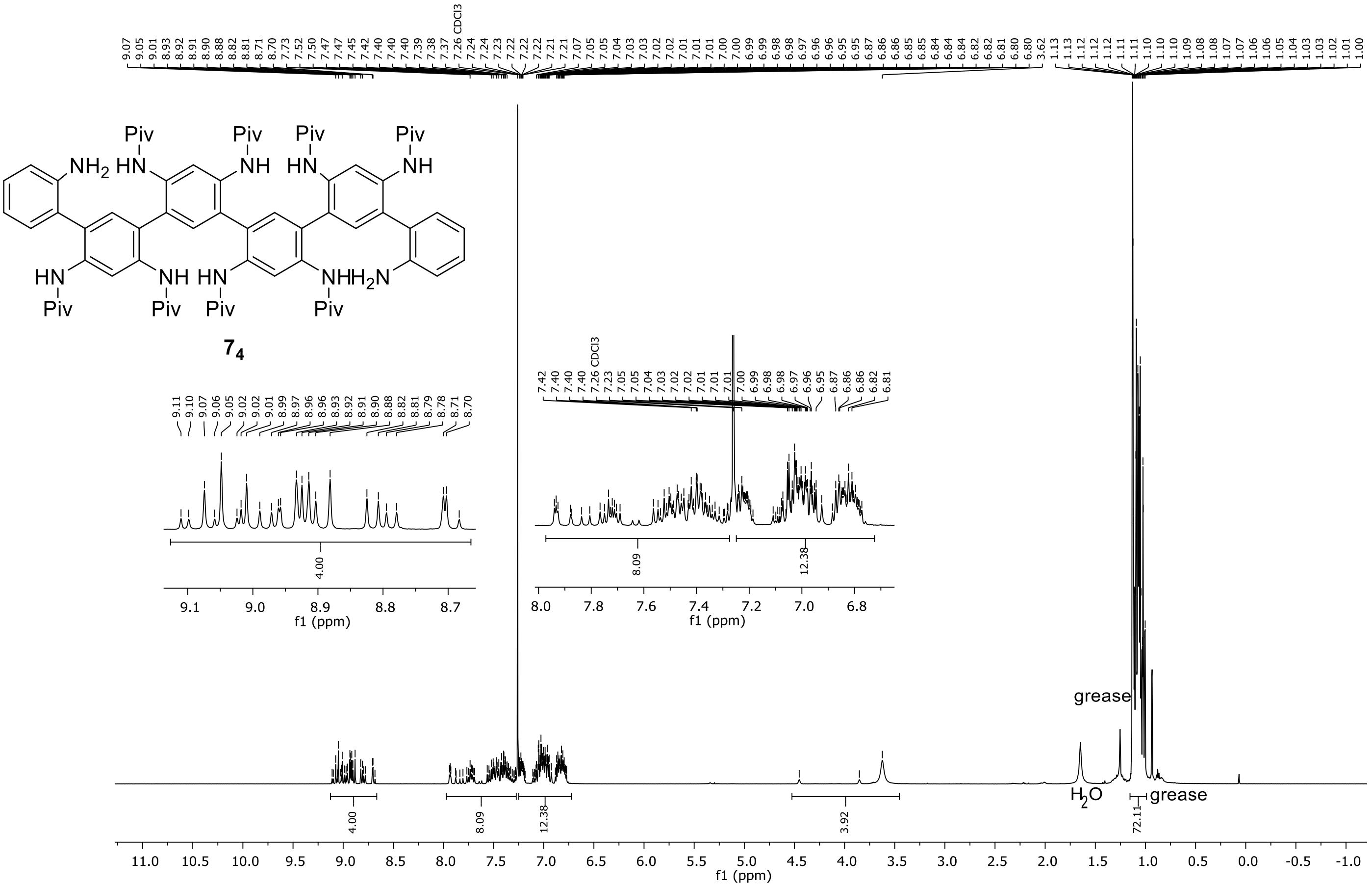

Figure S117. ^{1}H NMR (600 MHz, $CDCl_3$) spectrum of **7_4**.

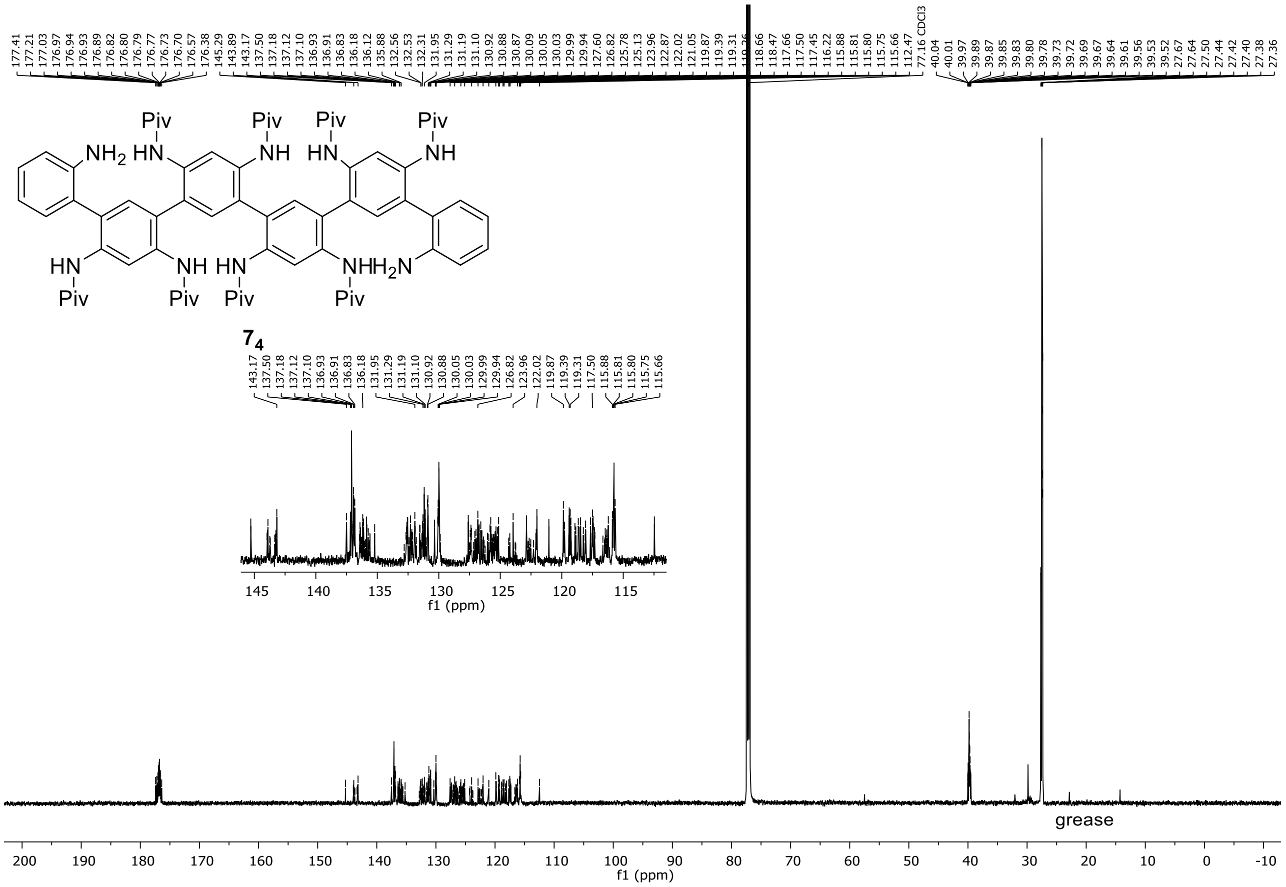

Figure S118. ^{13}C NMR (151 MHz, $CDCl_3$) spectrum of **7_4**.

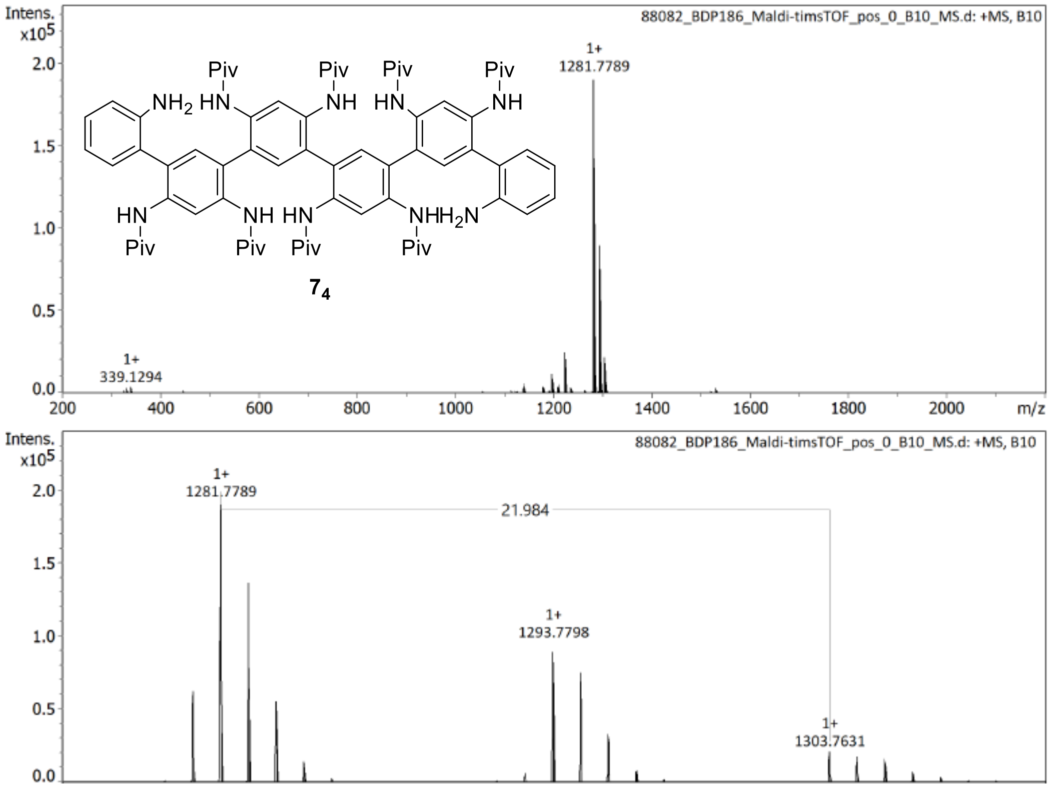

Figure S119. HRMS (MALDI-timsTOF, matrix: DCTB) spectrum of **7_4**.

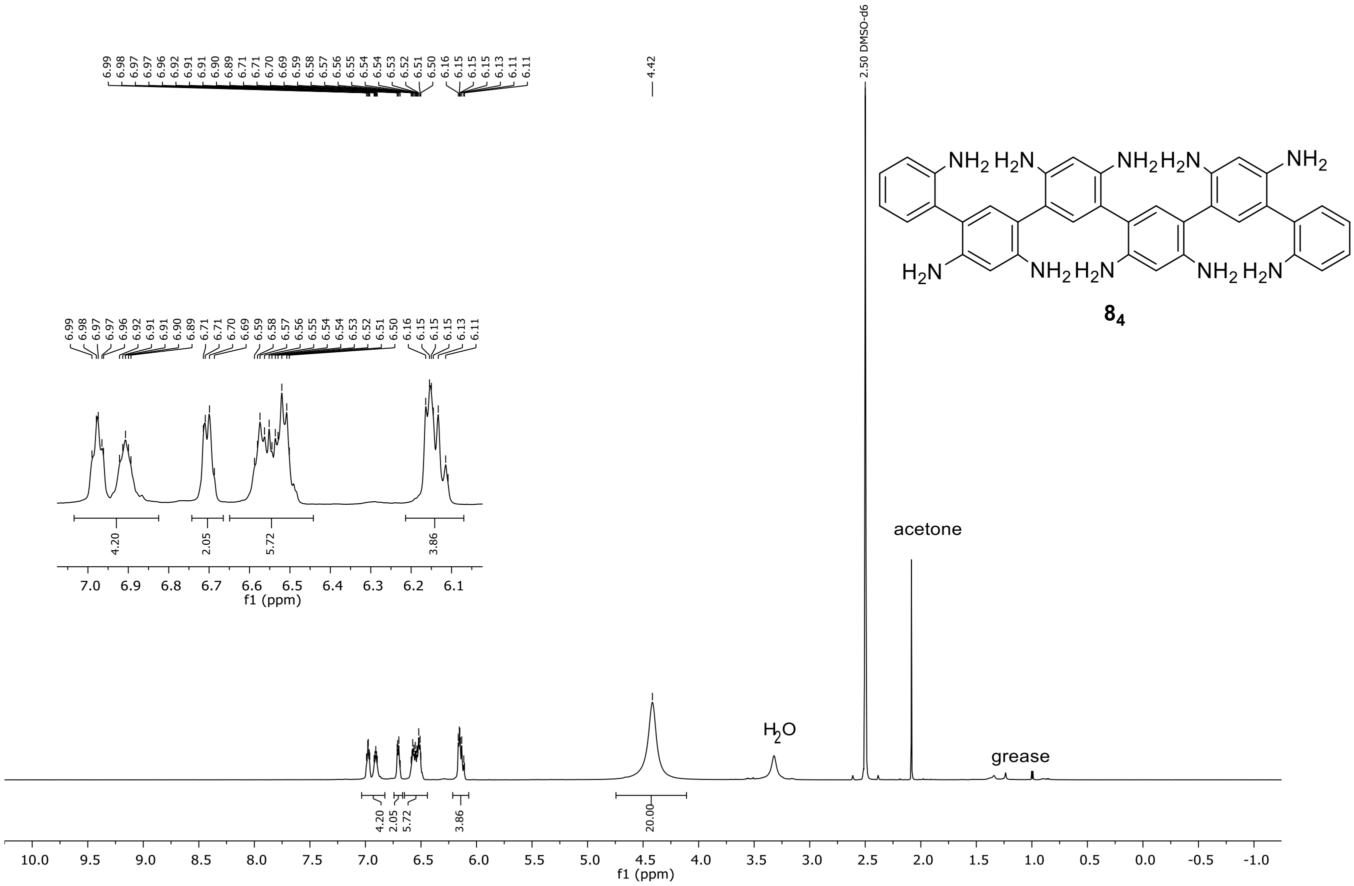

Figure S120. ^{1}H NMR (600 MHz, DMSO-d_6) spectrum of **8$_4$**.

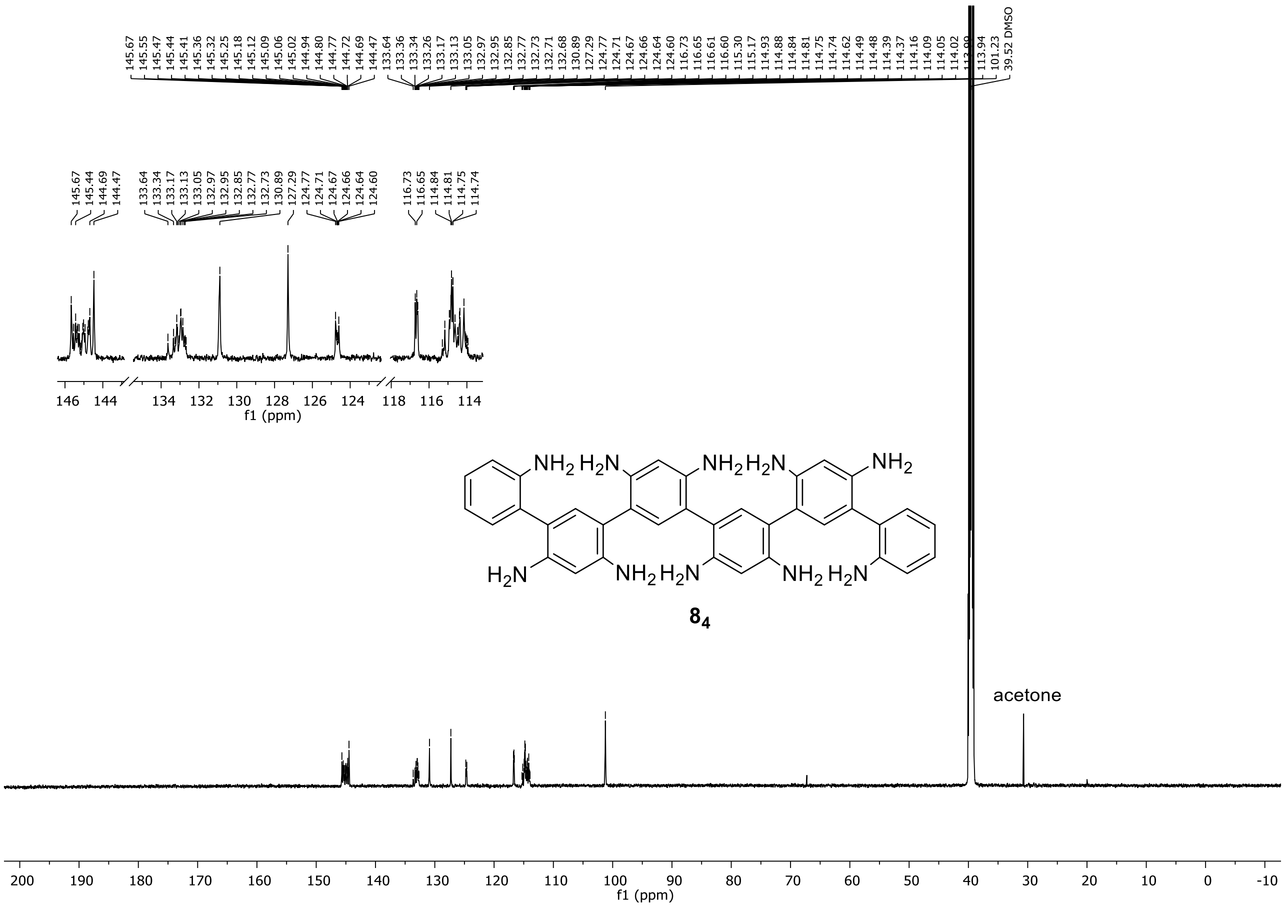

Figure S121. ^{13}C NMR (151 MHz, DMSO-d_6) spectrum of **8$_4$**.

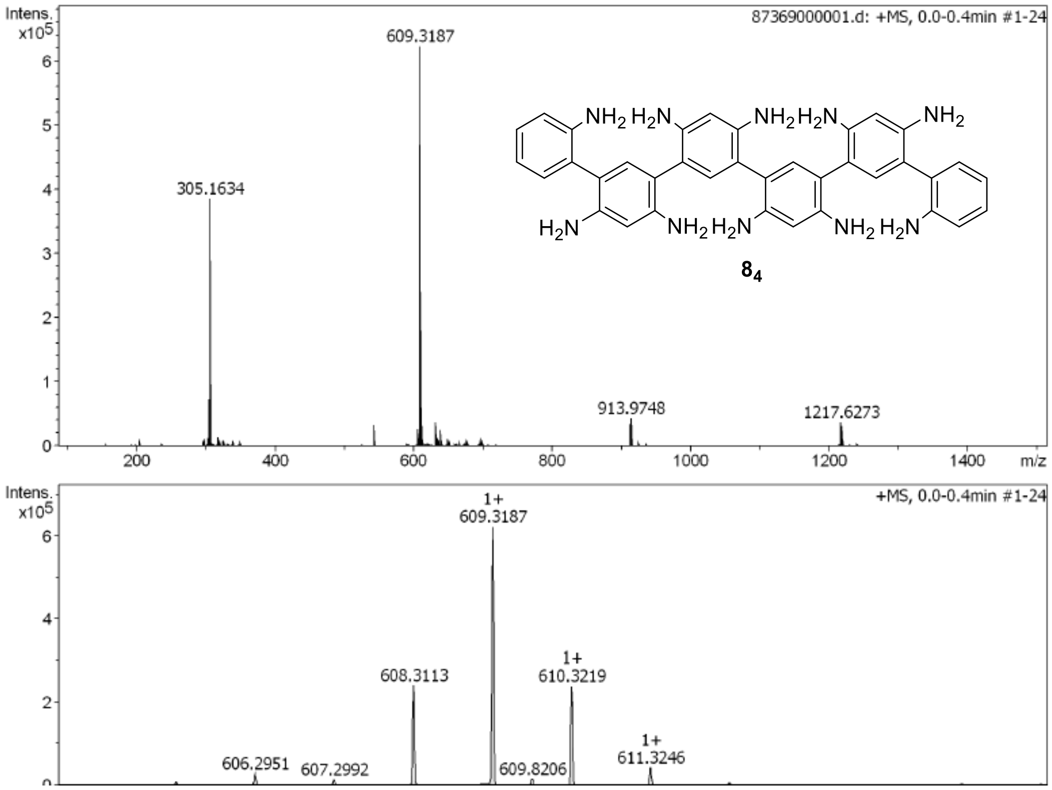

Figure S122. HRMS (ESI) spectrum of **8_4**.

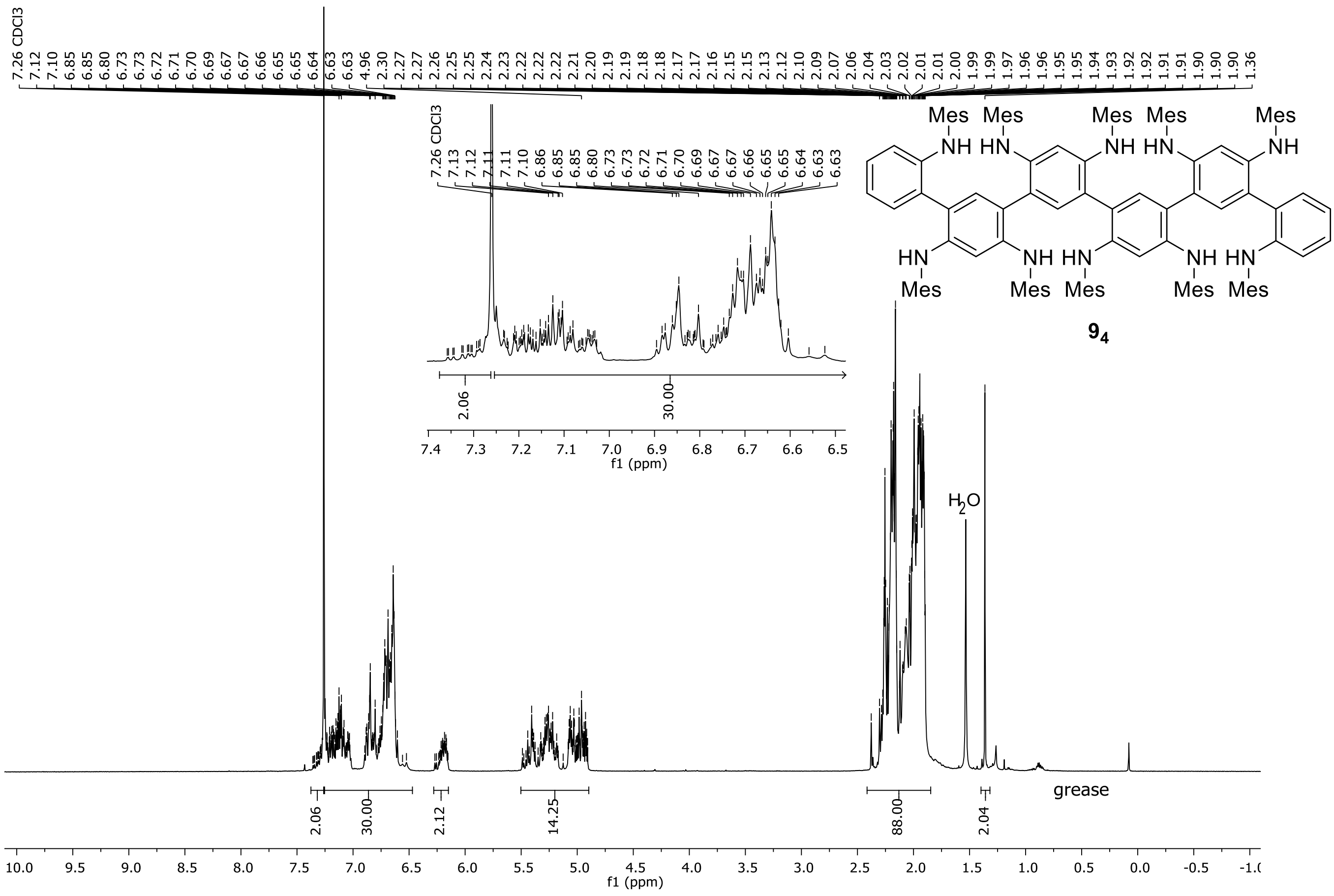

Figure S123. ^{1}H NMR (600 MHz, $CDCl_3$) spectrum of **9_4**.

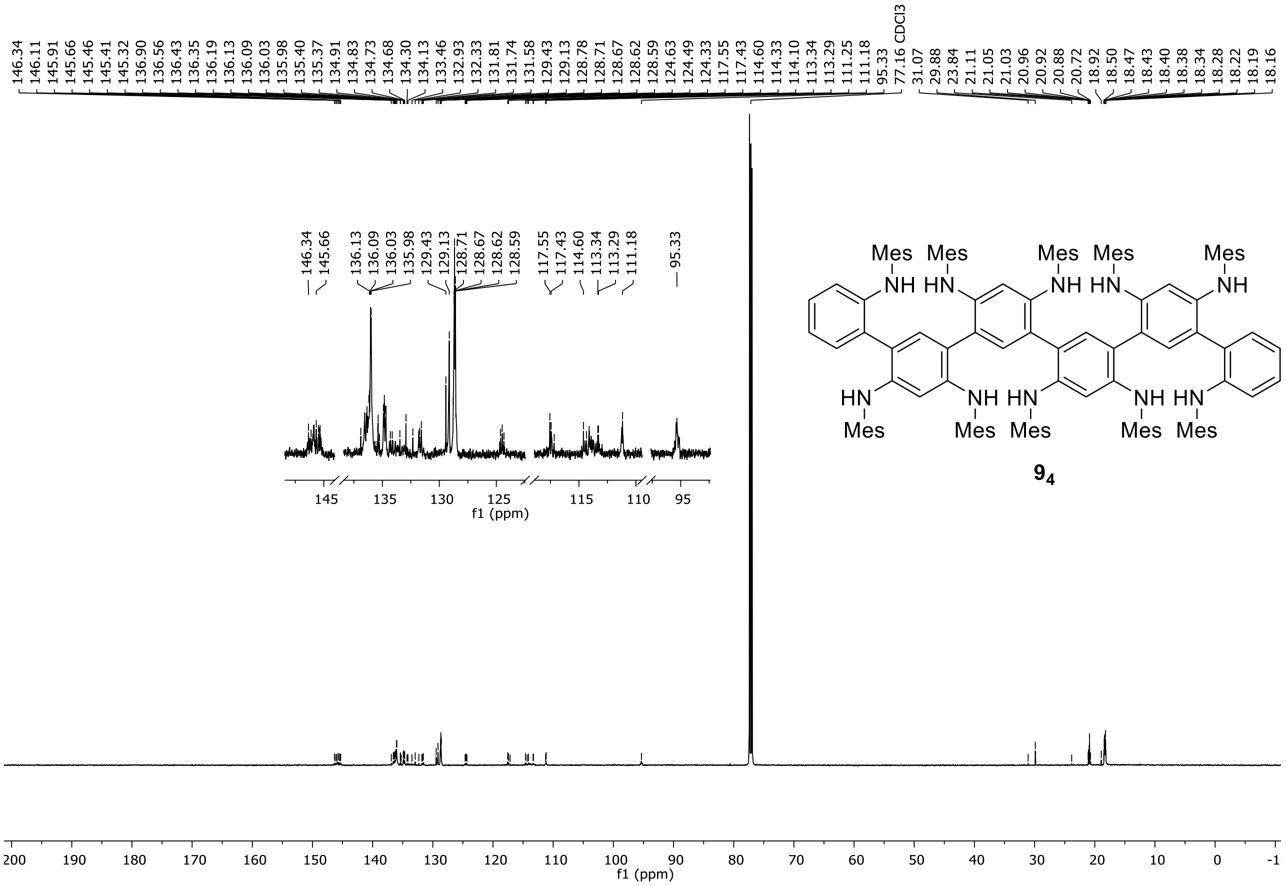

Figure S124. ^{13}C NMR (151 MHz, $CDCl_3$) spectrum of **9_4**.

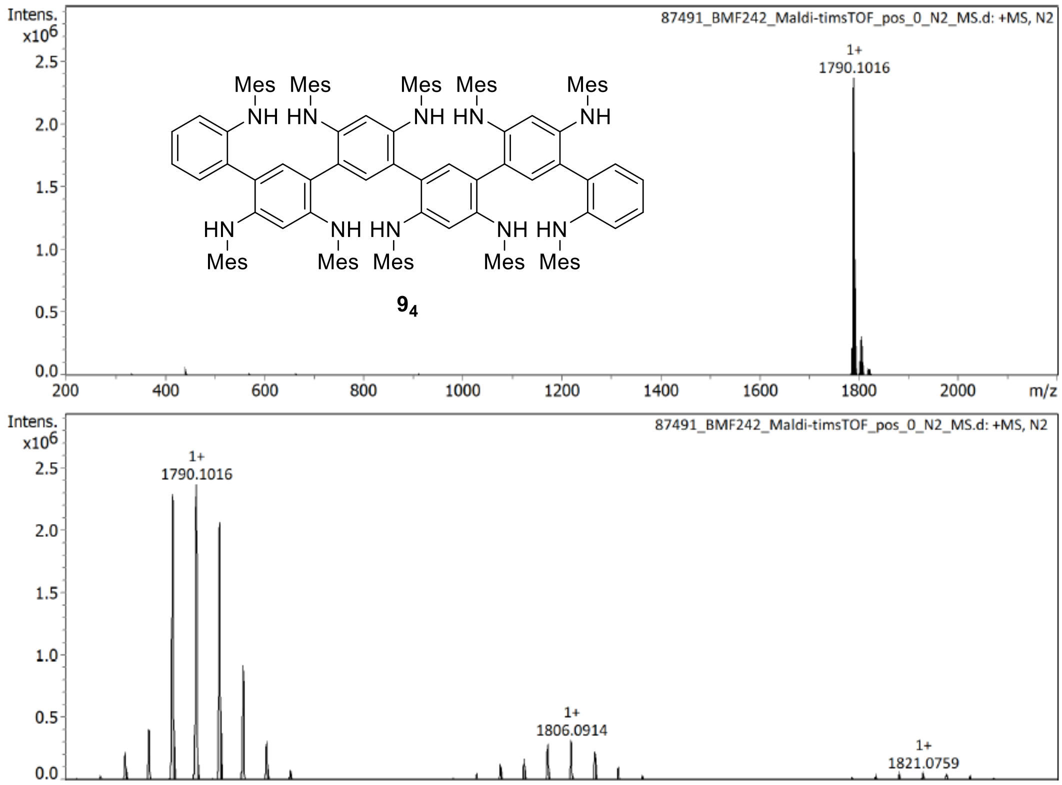

Figure S125. HRMS (MALDI-timsTOF, matrix: DCTB) spectrum of **9_4**.

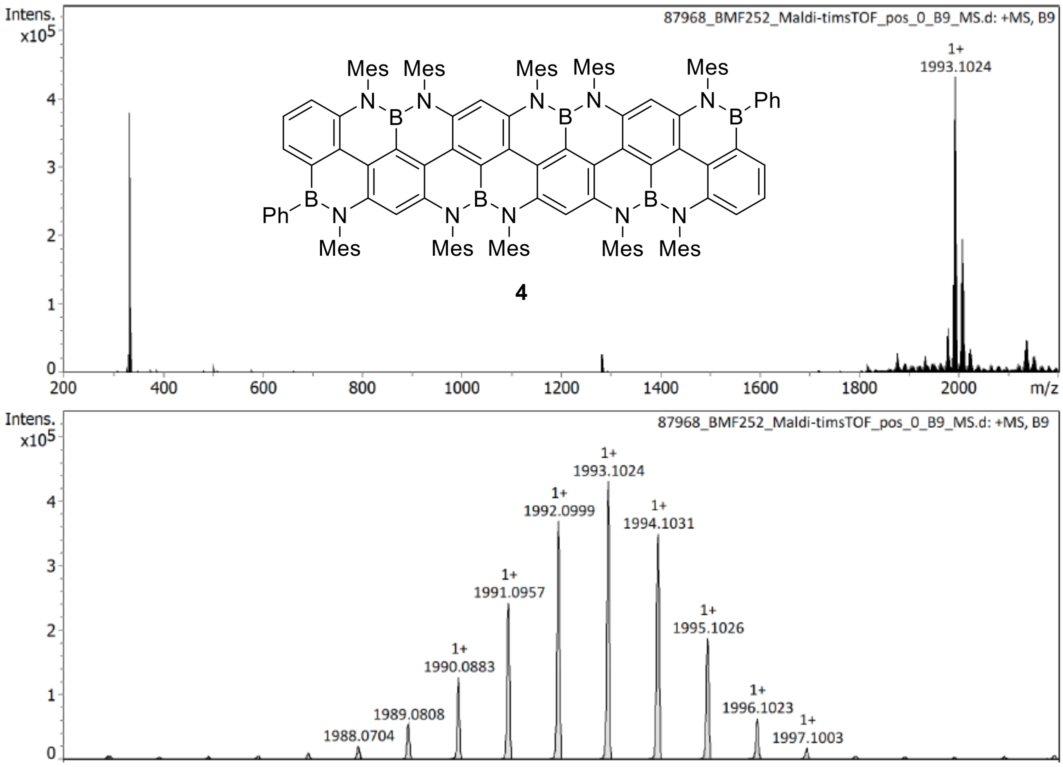

Figure S126. HRMS (MALDI-timsTOF, matrix: DCTB) spectrum of **4**.

3. Optoelectronic characterization

3.1. Photophysical properties

Photophysical characterization of 5^{Ph}

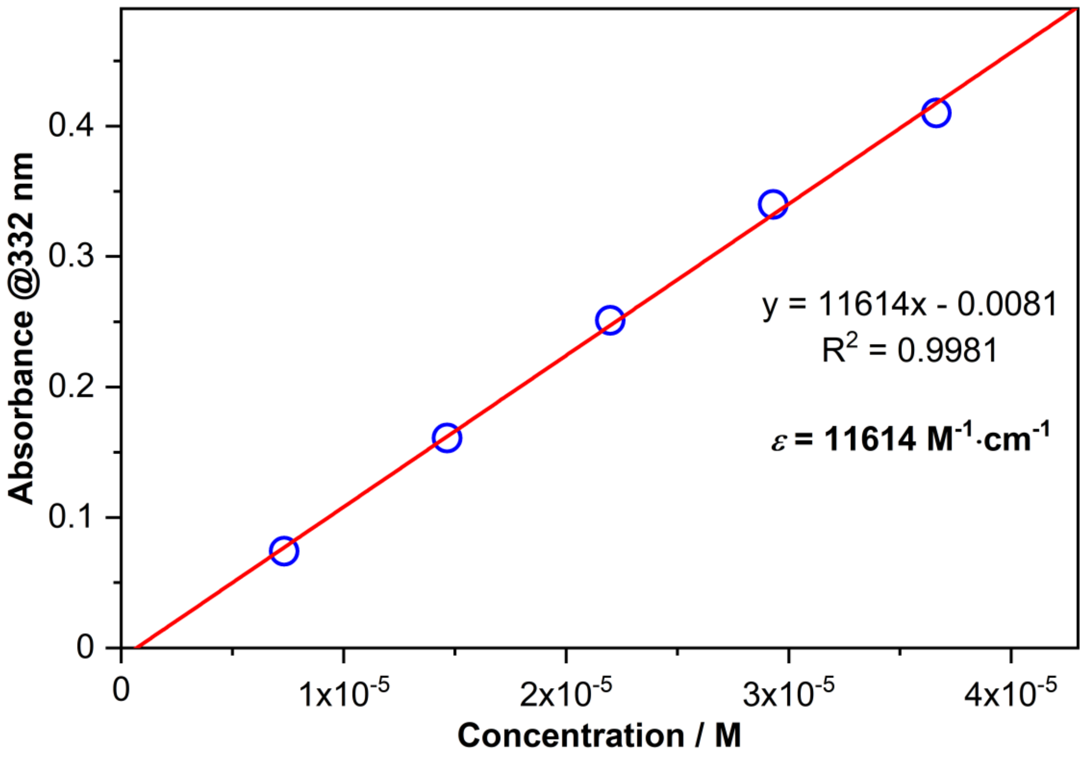

Figure S127. Determination of the molar attenuation coefficient (ε) of 5^{Ph} in 2-MeTHF.

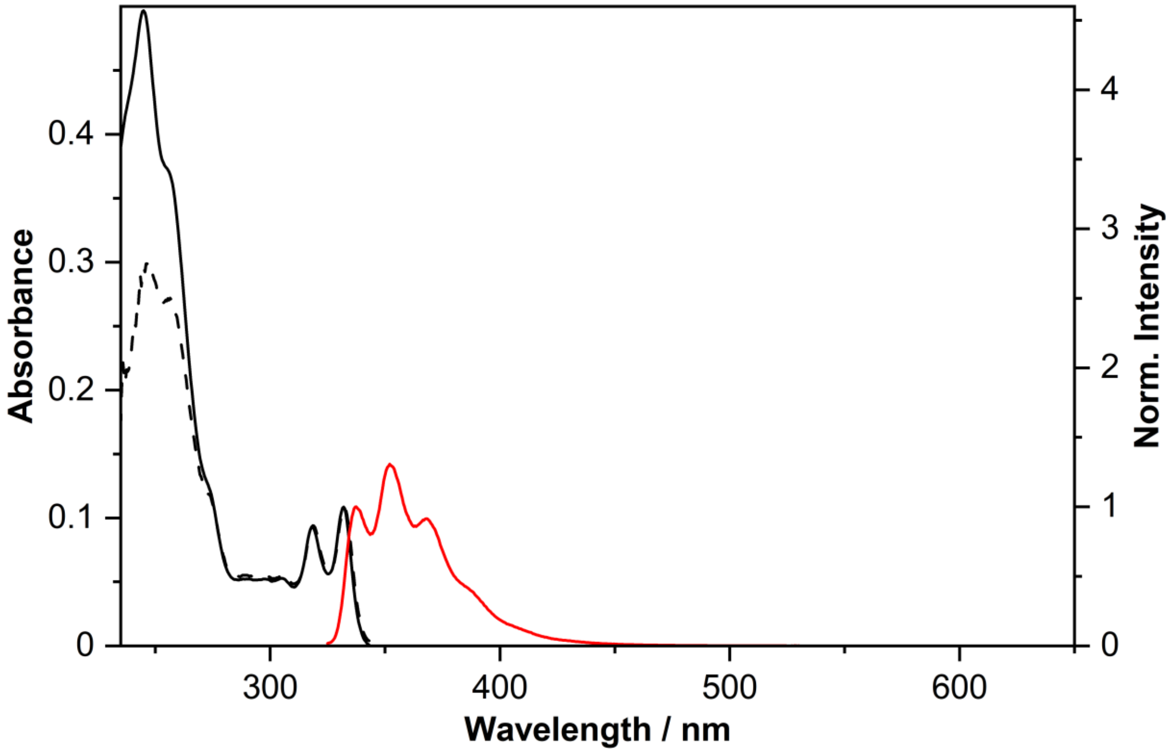

Figure S128. UV-Vis absorption (black), normalized steady-state excitation (dashed, λ_{em} = 353 nm) and emission spectra (red, λ_{ex} = 318 nm) of 5^{Ph} (9.4×10^{-6} M) at 293.15 K in 2-MeTHF.

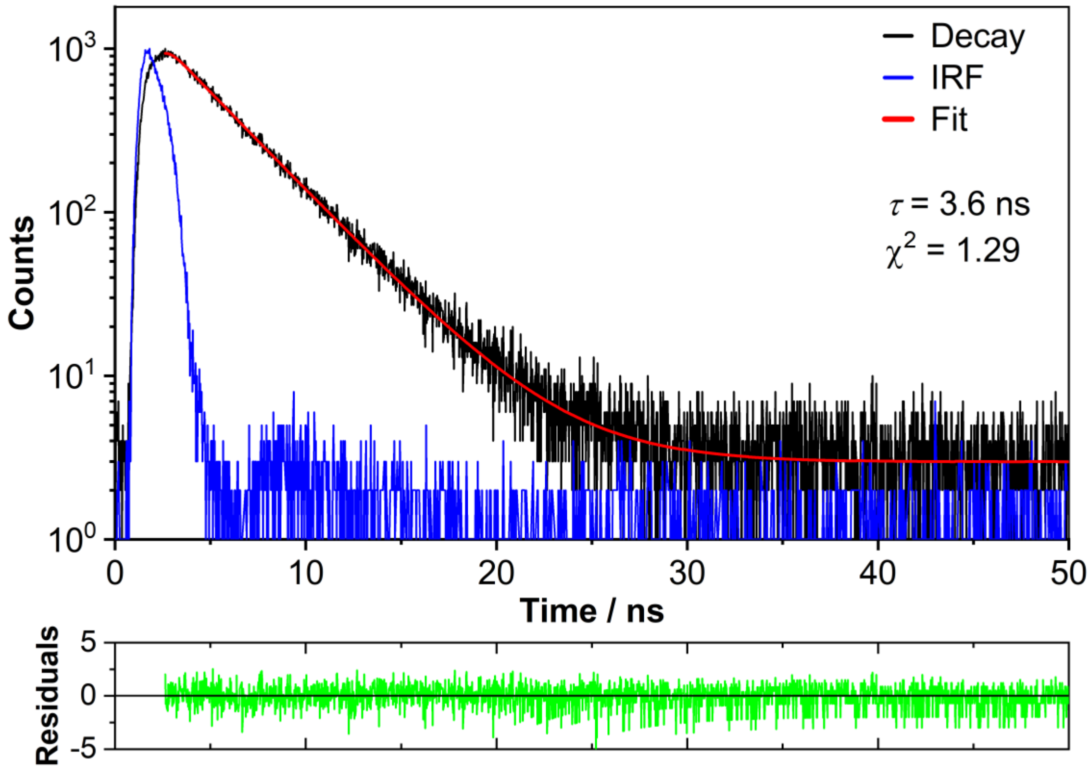

Figure S129. Time-resolved emission decay (λ_{ex} = 294.4 nm, λ_{em} = 358 nm) of $\mathbf{5^{Ph}}$ (9.4×10^{-6} M) at 293.15 K in 2-MeTHF.

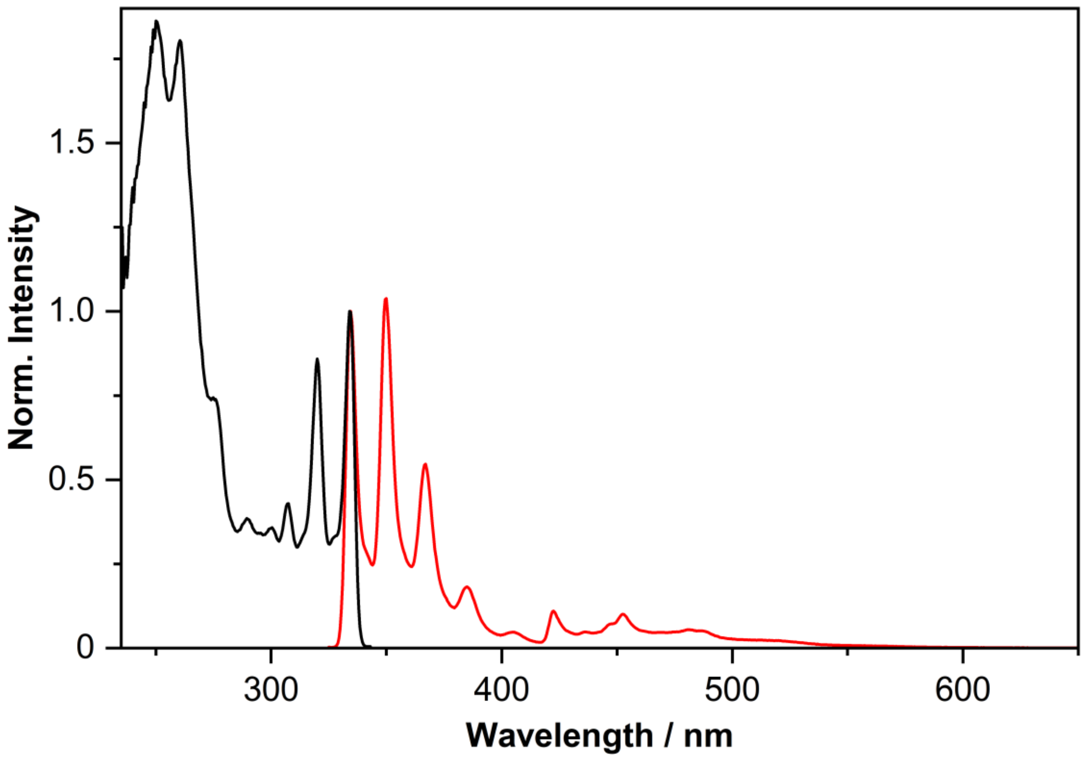

Figure S130. Normalized steady-state excitation (black, λ_{em} = 353 nm) and emission (red, λ_{ex} = 318 nm) spectra of $\mathbf{5^{Ph}}$ (9.4×10^{-6} M) at 77 K in 2-MeTHF.

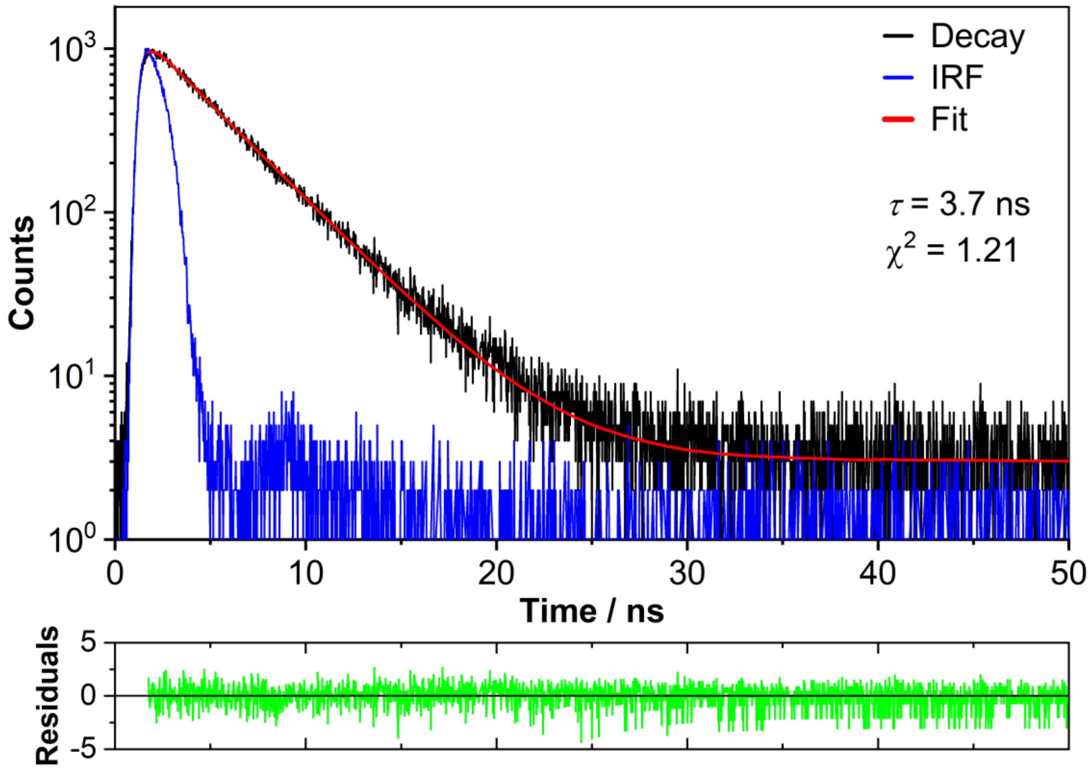

Figure S131. Time-resolved emission decay (λ_{ex} = 294.4 nm, λ_{em} = 358 nm) of **5^{Ph}** (9.4×10^{-6} M) at 77 K in 2-MeTHF.

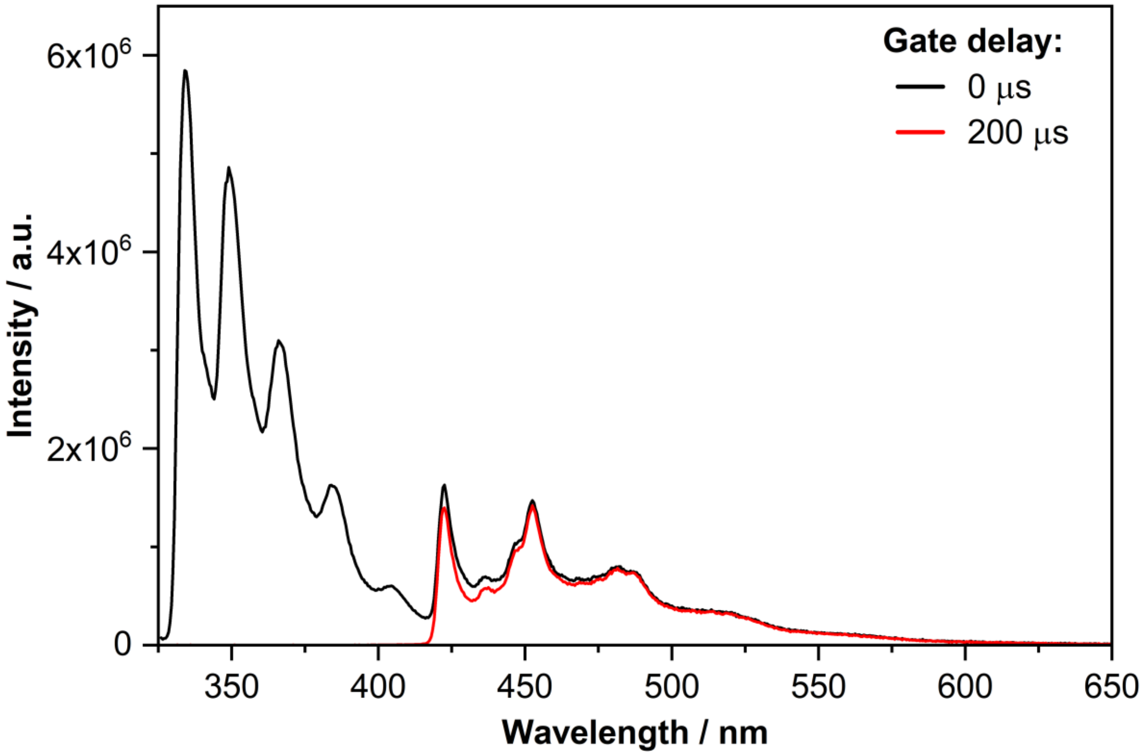

Figure S132. Time-resolved emission spectra (λ_{ex} = 318 nm) of **5^{Ph}** (9.4×10^{-6} M) at 77 K in 2-MeTHF, collected at different delay times.

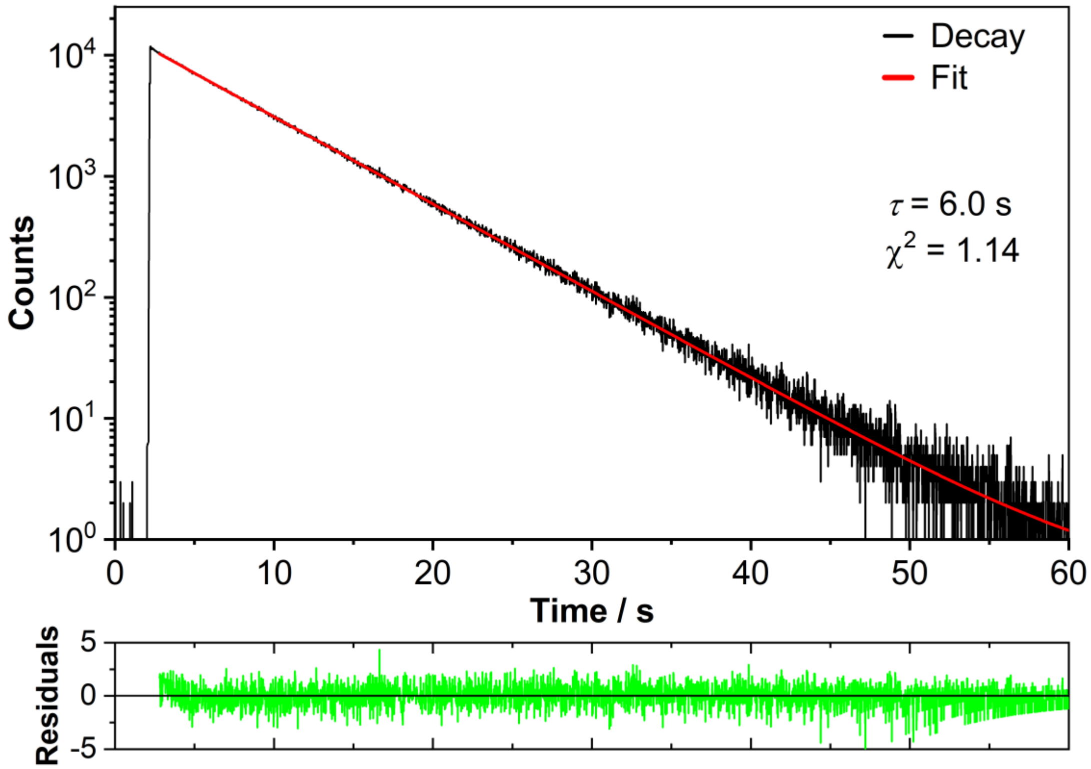

Figure S133. Time-resolved emission decay (λ_{ex} = 318 nm, λ_{em} = 423 nm) of **5^{Ph}** (9.4×10^{-6} M) at 77 K in 2-MeTHF.

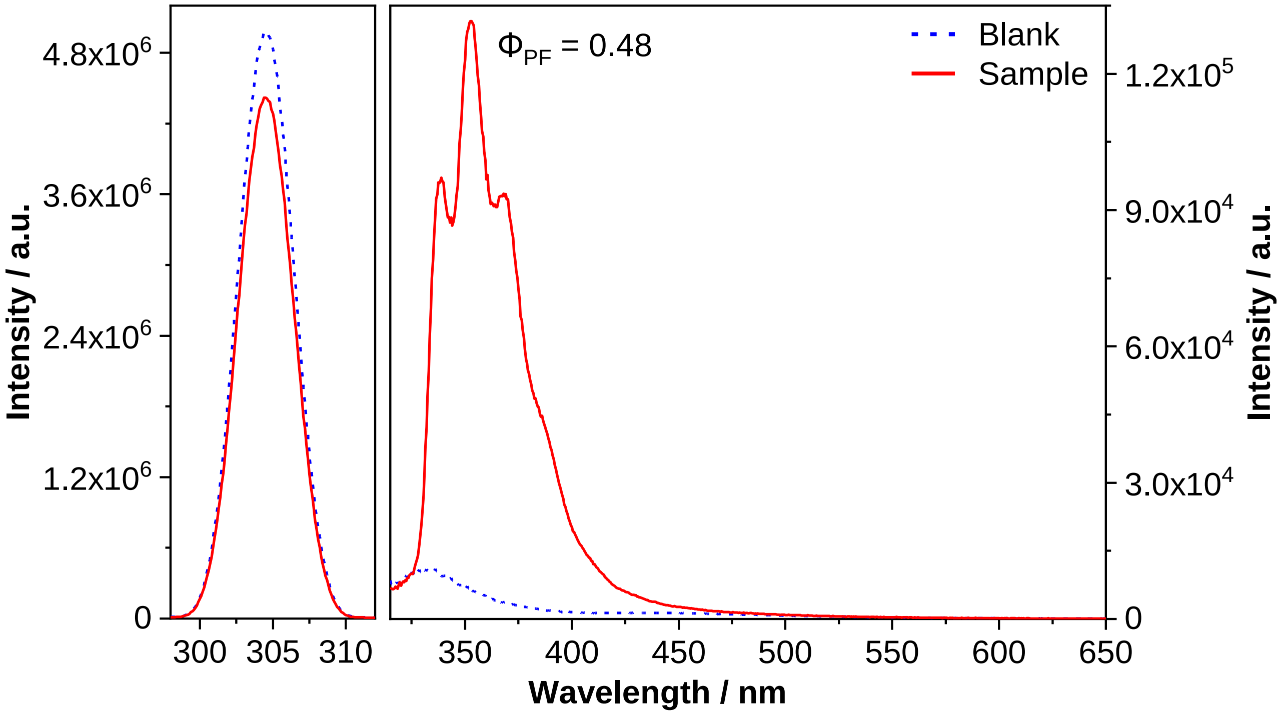

Figure S134. Excitation scatter region (left) and emission spectra (right, λ_{ex} = 305 nm) used to calculate the absolute quantum yield of **5^{Ph}** (9.4×10^{-6} M) in 2-MeTHF.

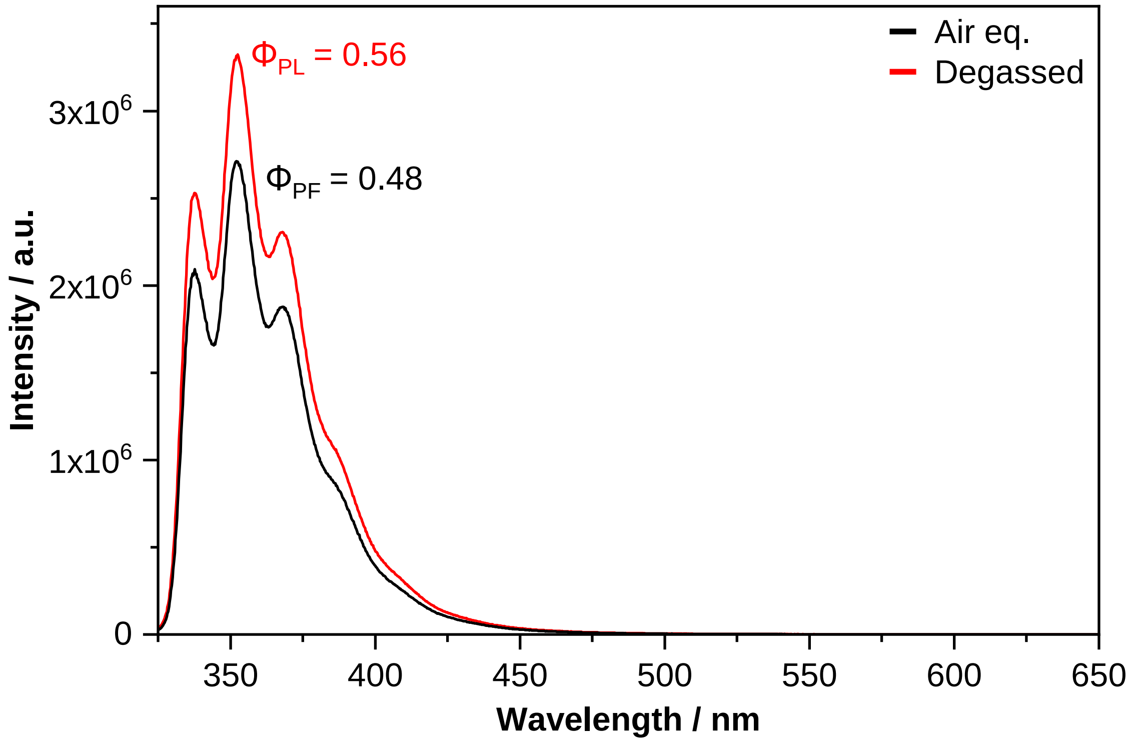

Figure S135. Steady-state emission spectra (λ_{ex} = 318 nm) of $\mathbf{5^{Ph}}$ (1.2×10^{-5} M) at 293.15 K in 2-MeTHF collected before (black) and after degassing (red).

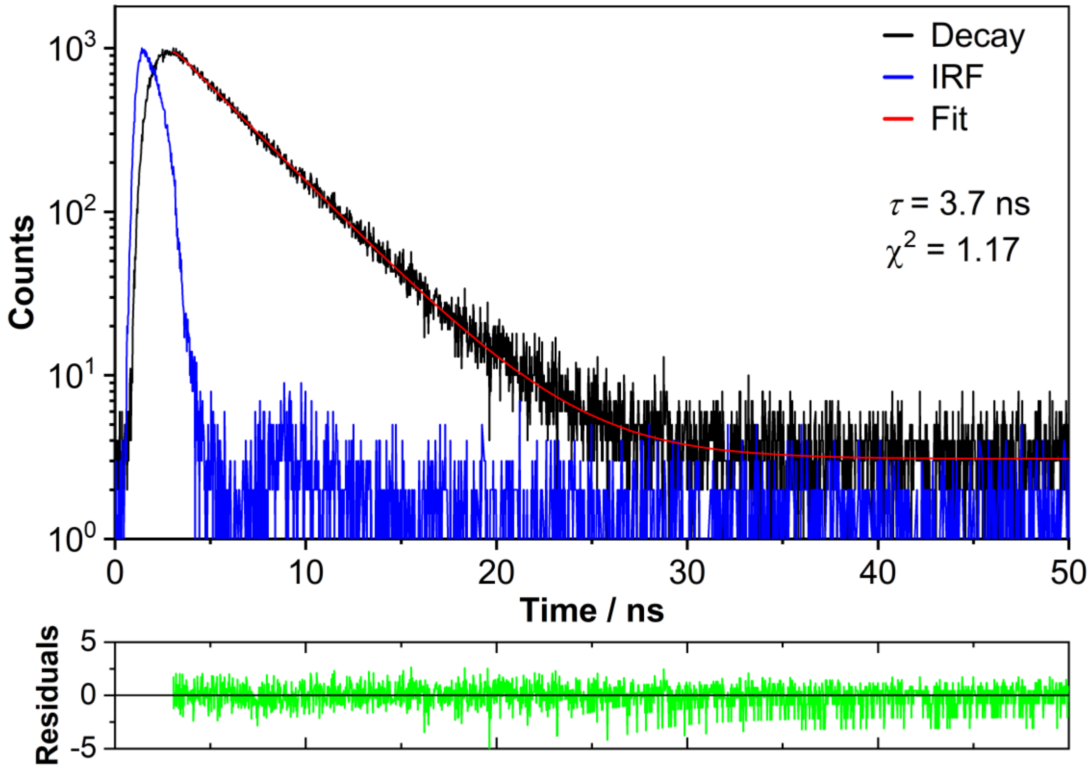

Figure S136. Time-resolved emission decay (λ_{ex} = 294.4 nm, λ_{em} = 353 nm) of $\mathbf{5^{Ph}}$ (1.2×10^{-5} M) at 293.15 K in degassed 2-MeTHF.

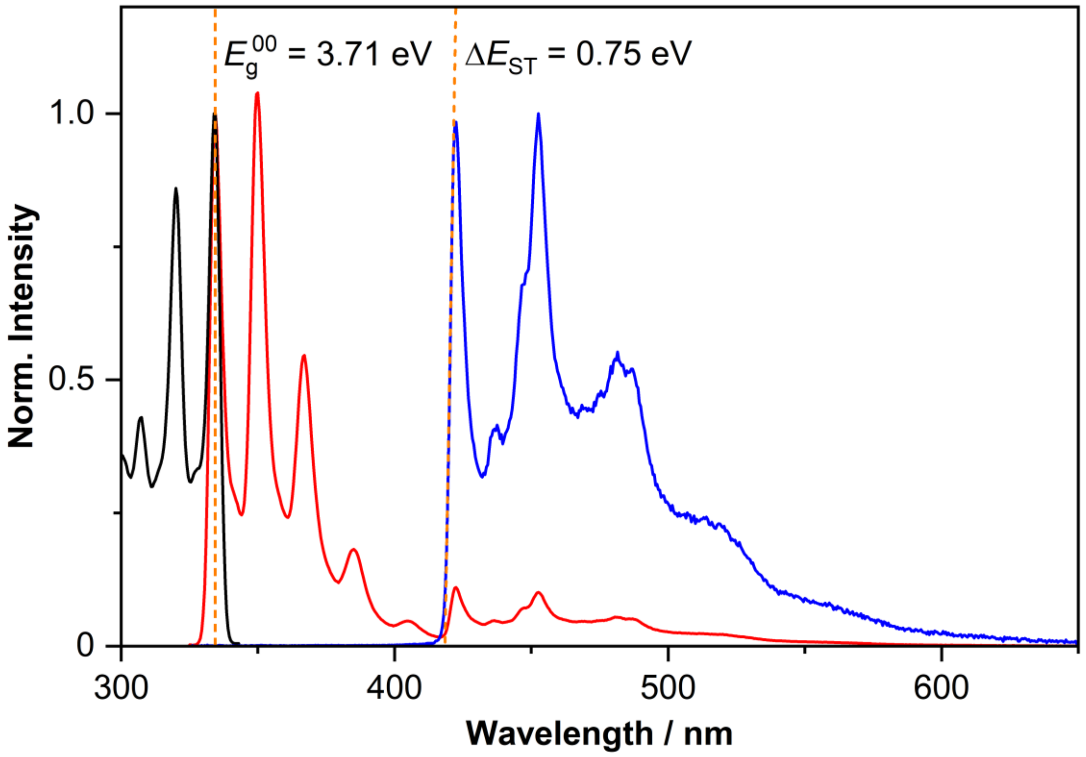

Figure S137. Determination of the optical bandgap (E_g^{00}) and the singlet-triplet gap (ΔE_{ST}) of $\mathbf{5^{Ph}}$ (9.4×10^{-6} M) at 77 K in 2-MeTHF, using the intercept of the normalized steady-state excitation (black, λ_{em} = 353 nm) and emission (red, λ_{ex} = 318 nm) spectra, and the time-resolved emission spectra (blue, λ_{ex} = 318 nm, 200 µs gate delay).

Photophysical characterization of **6**

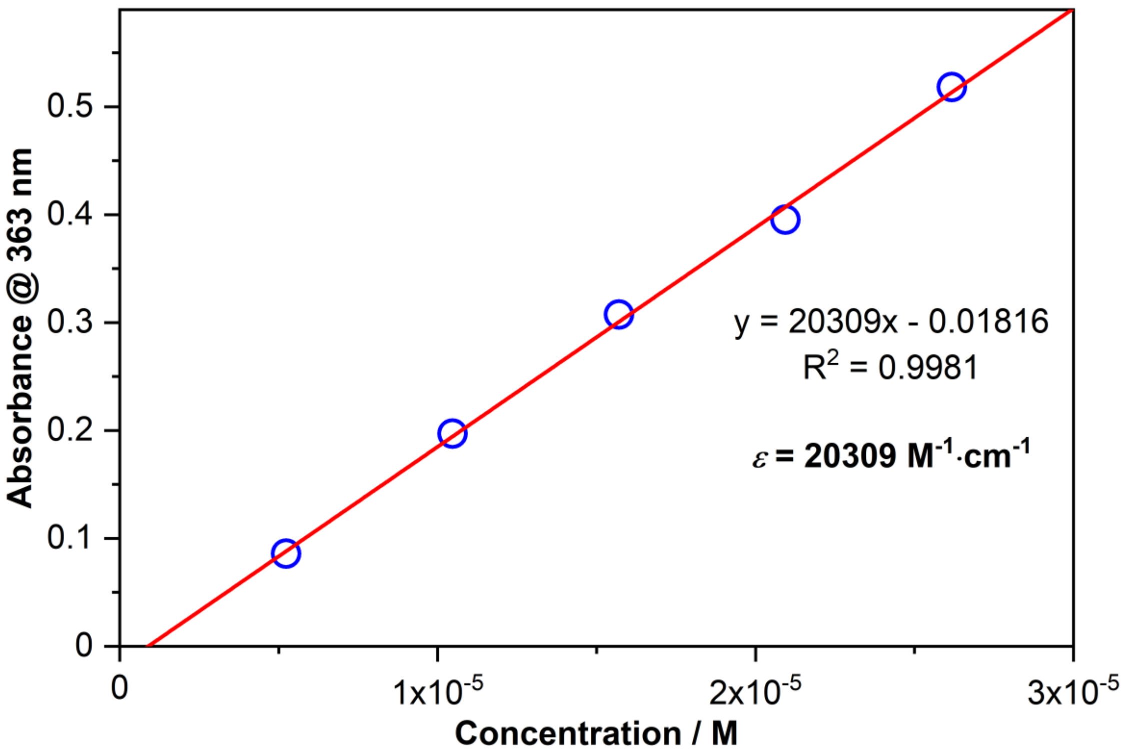

Figure S138. Determination of the molar attenuation coefficient (ε) of **6** in 2-MeTHF.

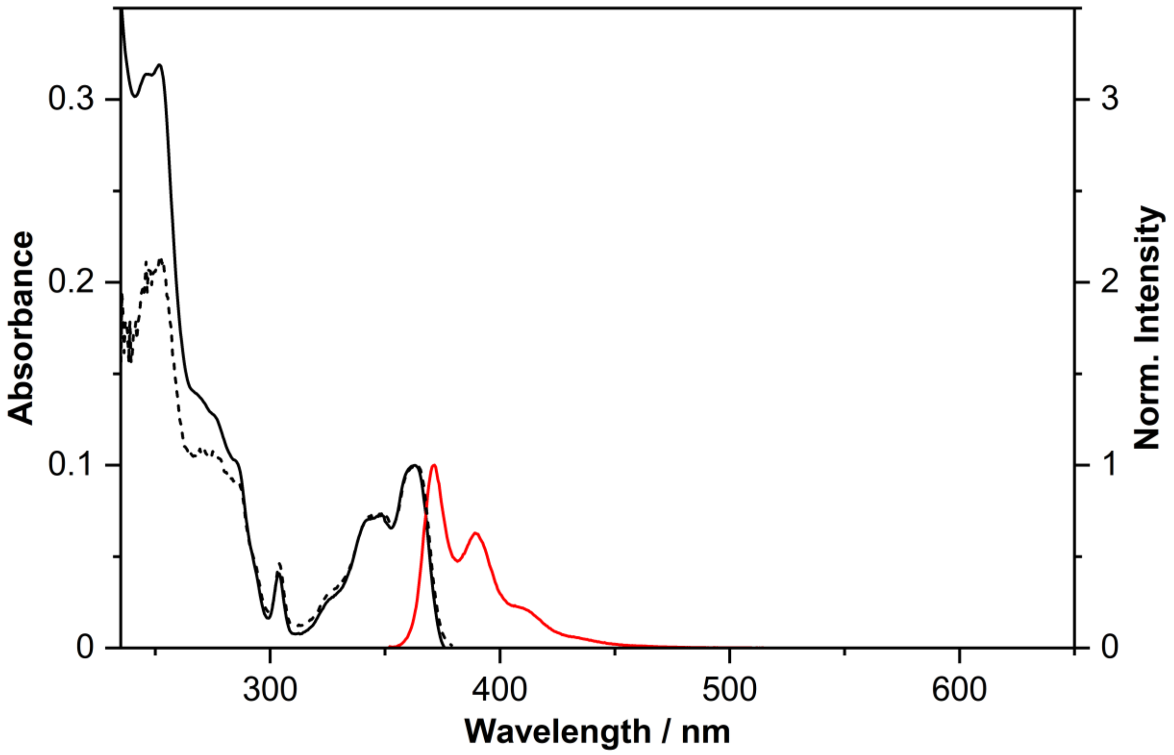

Figure S139. UV-Vis absorption spectra (black), normalized steady-state excitation (dashed, λ_{em} = 389 nm) and emission spectra (red, λ_{ex} = 345 nm) of **6** (4.9×10^{-6} M) at 293.15 K in 2-MeTHF.

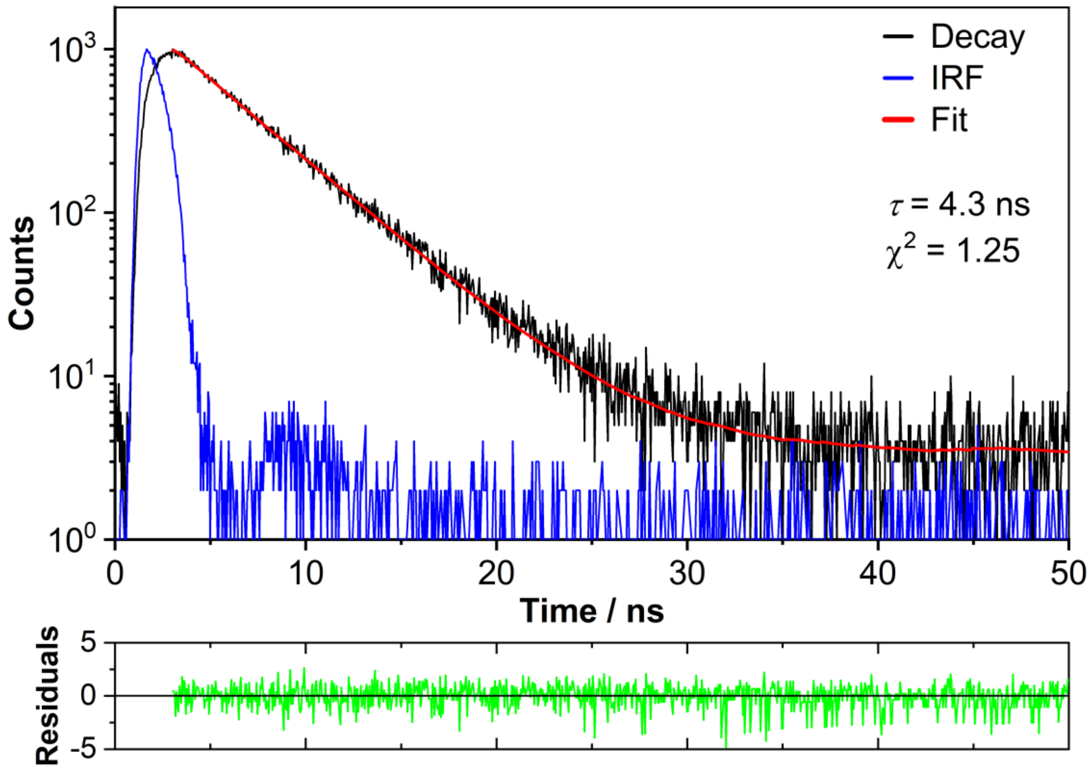

Figure S140. Time-resolved emission decay (λ_{ex} = 294.4 nm, λ_{em} = 389 nm) of **6** (4.9×10^{-6} M) at 293.15 K in 2-MeTHF.

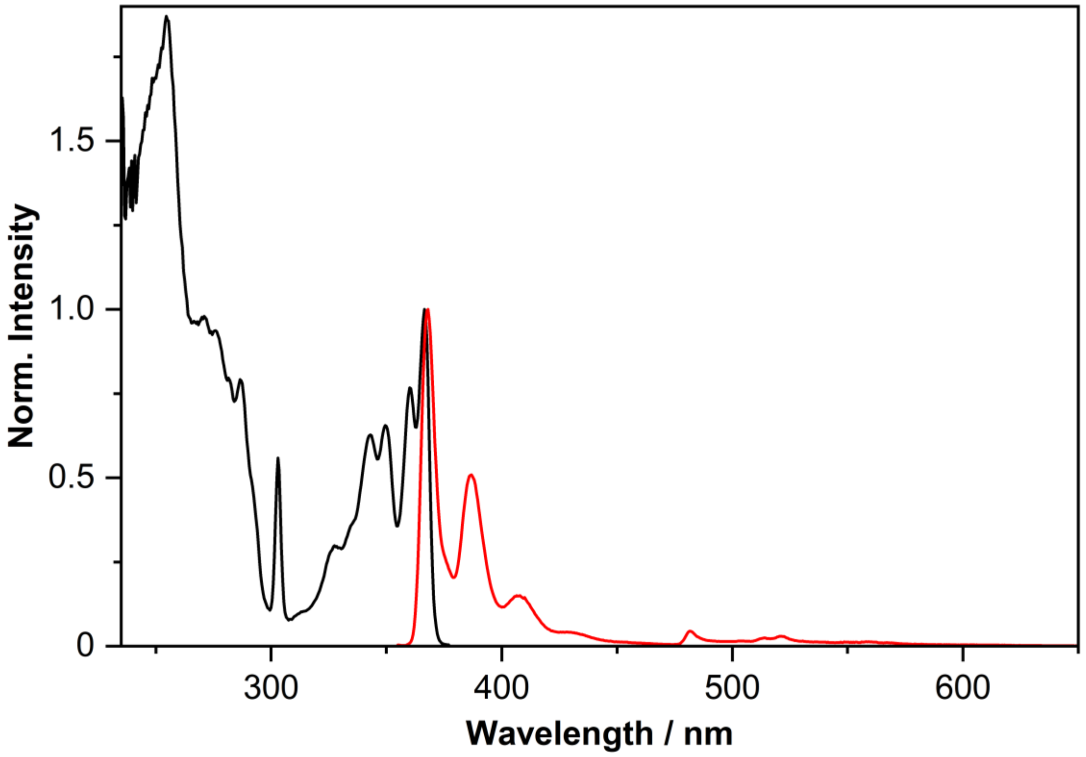

Figure S141. Normalized steady-state excitation (black, λ_{em} = 387 nm) and emission (red, λ_{ex} = 345 nm) spectra of **6** (4.9×10^{-6} M) at 77 K in 2-MeTHF.

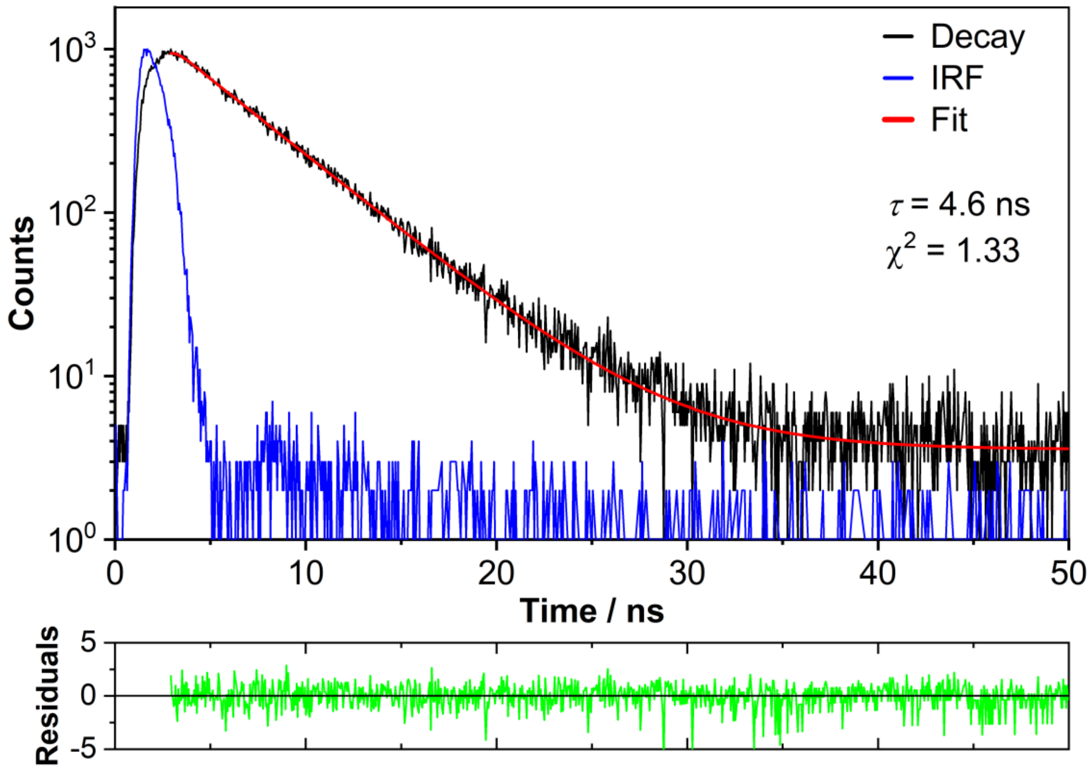

Figure S142. Time-resolved emission decay (λ_{ex} = 294.4 nm, λ_{em} = 387 nm) of **6** (4.9×10^{-6} M) at 77 K in 2-MeTHF.

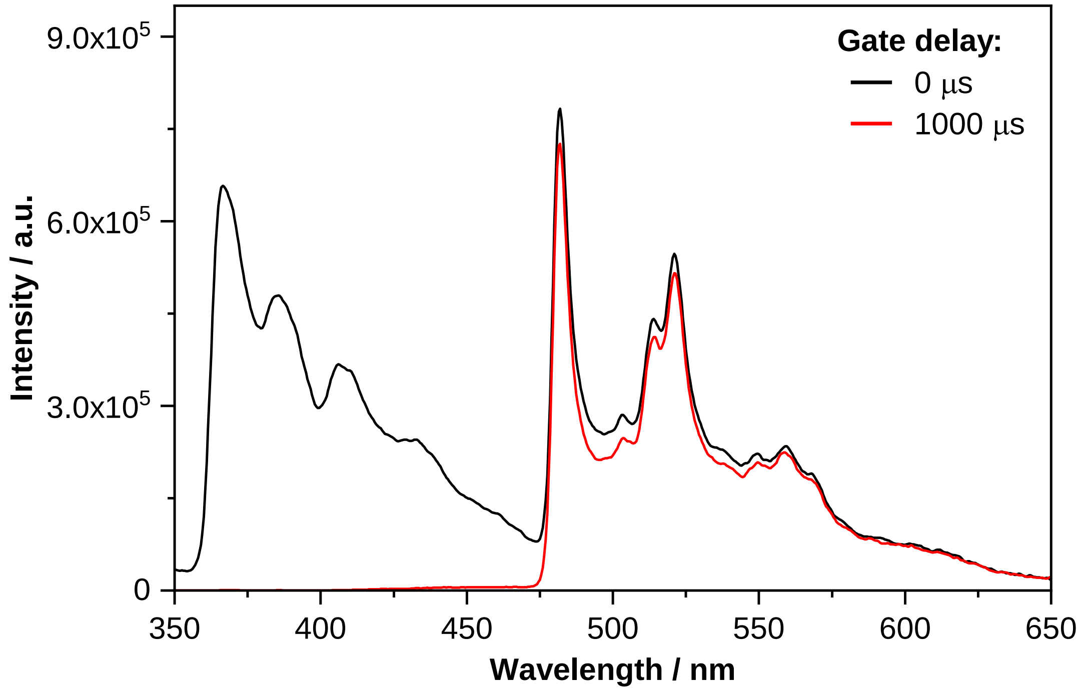

Figure S143. Time-resolved emission spectra (λ_{ex} = 340 nm) of **6** (4.9×10^{-6} M) at 77 K in 2-MeTHF, collected at different delay times.

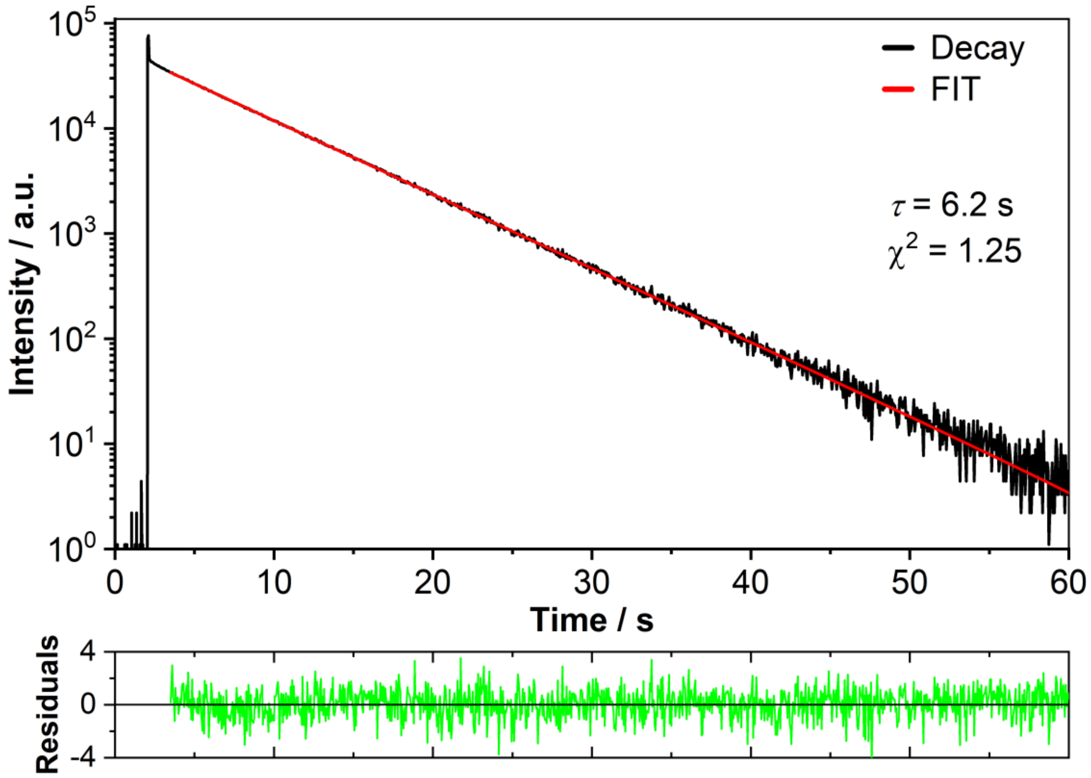

Figure S144. Time-resolved emission decay (λ_{ex} = 340 nm, λ_{em} = 482 nm) of **6** (4.9×10^{-6} M) at 77 K in 2-MeTHF.

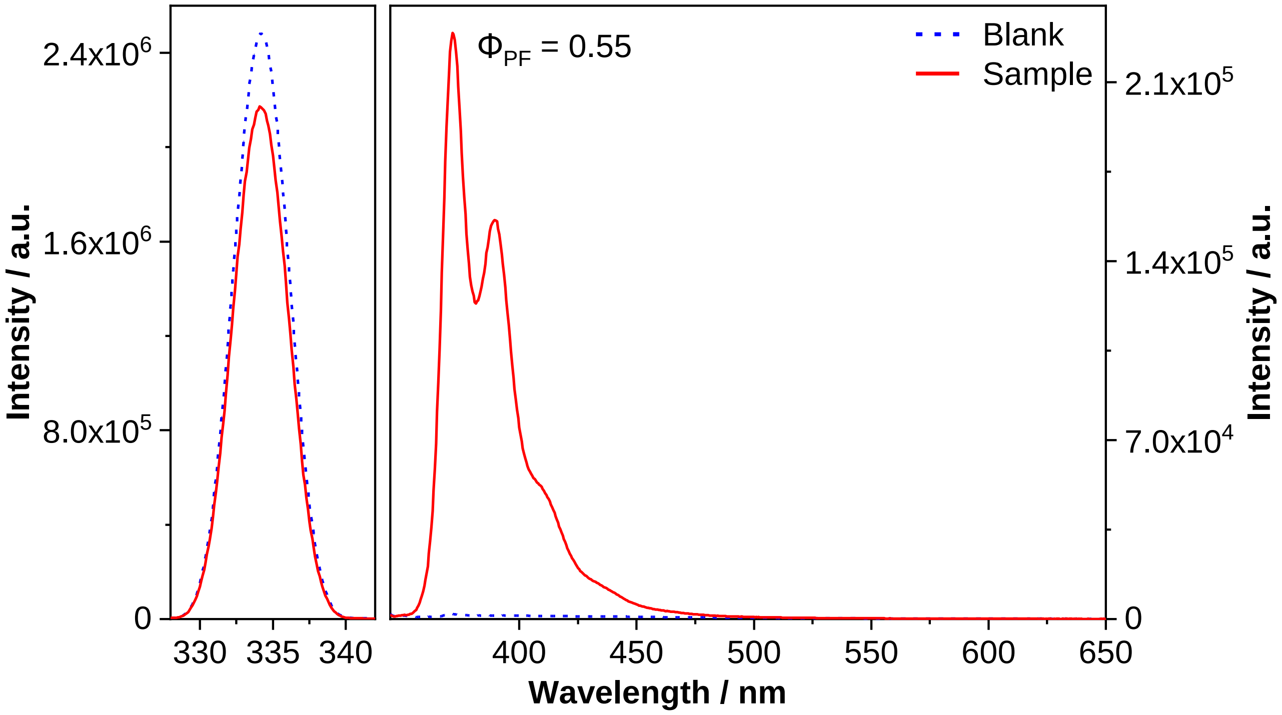

Figure S145. Excitation scatter region (left) and emission spectra (right, λ_{ex} = 335 nm) used to calculate the absolute quantum yield of **6** (4.9×10^{-6} M) in 2-MeTHF.

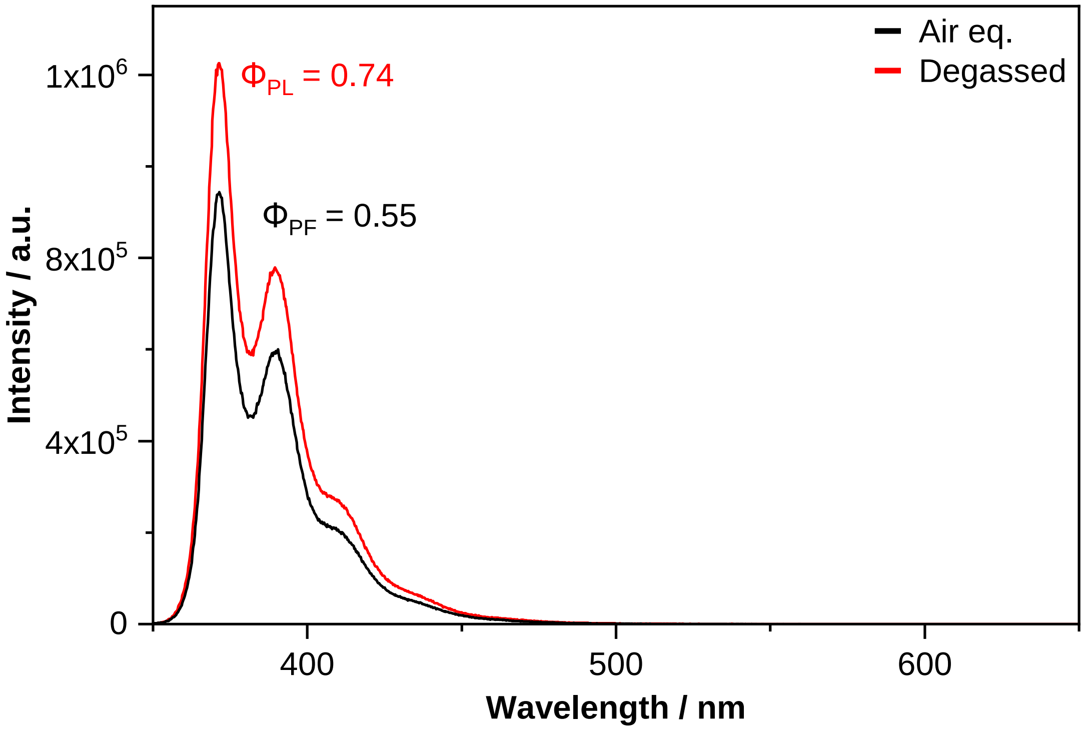

Figure S146. Steady-state emission spectra (λ_{ex} = 345 nm) of **6** (5.8×10^{-6} M) at 293.15 K in 2-MeTHF collected before (black) and after (red) degassing.

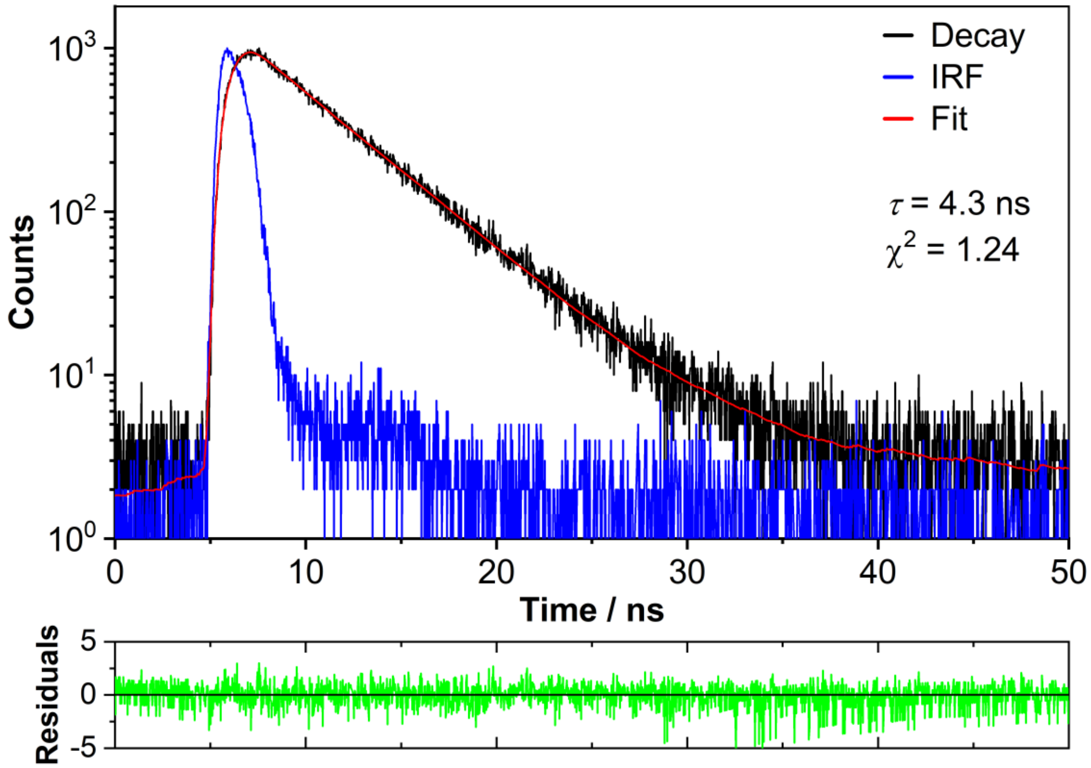

Figure S147. Time-resolved emission decay (λ_{ex} = 294.4 nm, λ_{em} = 371.5 nm) of **6** (5.8×10^{-6} M) at 293.15 K in degassed 2-MeTHF.

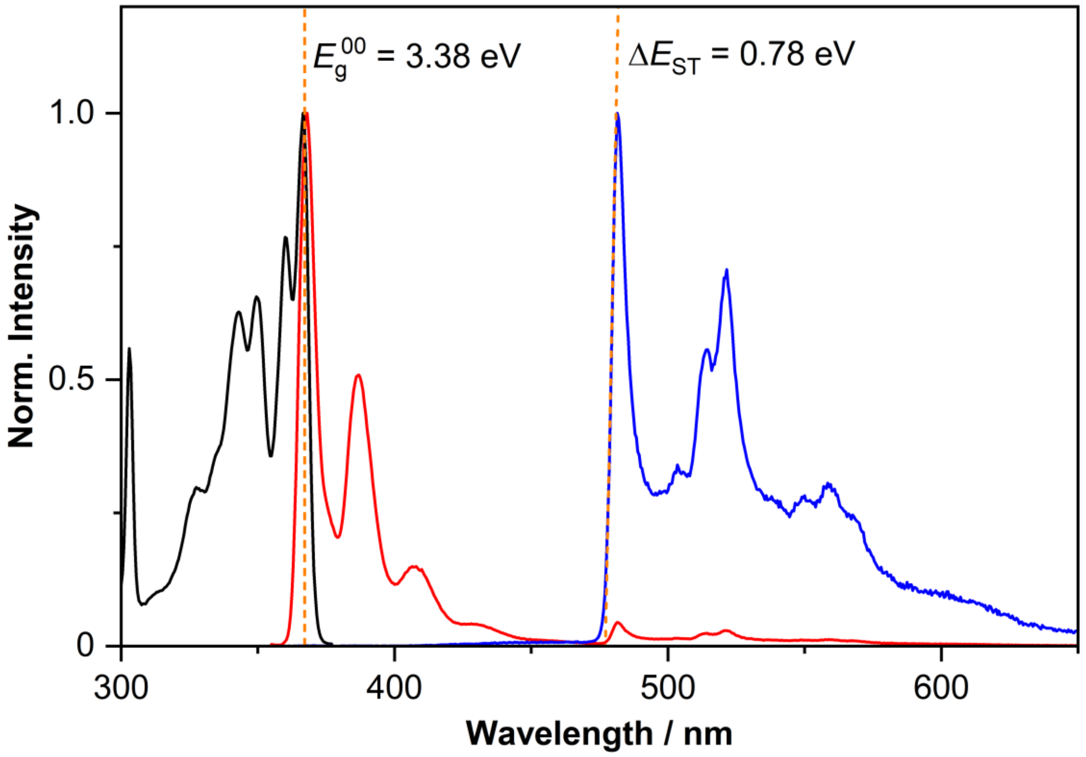

Figure S148. Determination of the optical bandgap (E_g^{00}) and the singlet-triplet gap (ΔE_{ST}) of **6** (4.9×10^{-6} M) at 77 K in 2-MeTHF, using the intercept of the normalized steady-state excitation (black, λ_{em} = 387 nm) and emission (red, λ_{ex} = 345 nm) spectra, and the time-resolved emission spectra (blue, λ_{ex} = 340 nm, 1000 µs gate delay).

Photophysical characterization of $\mathbf{1^{Ph}}$

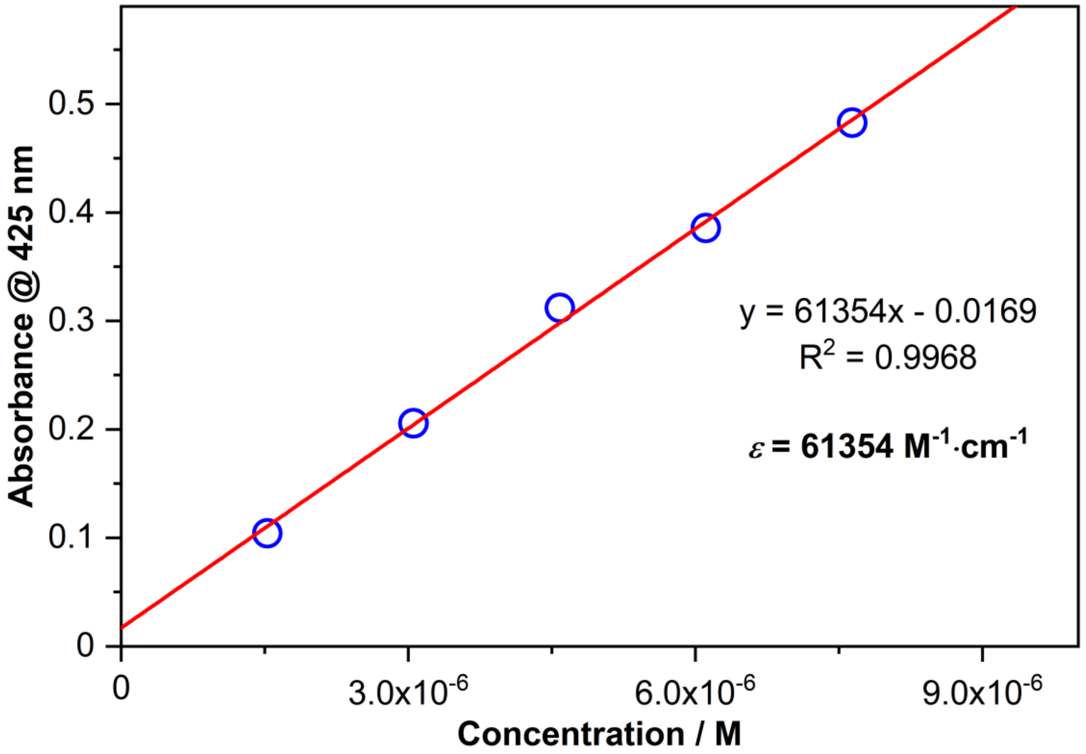

Figure S149. Determination of the molar attenuation coefficient (ε) of $\mathbf{1^{Ph}}$ in 2-MeTHF.

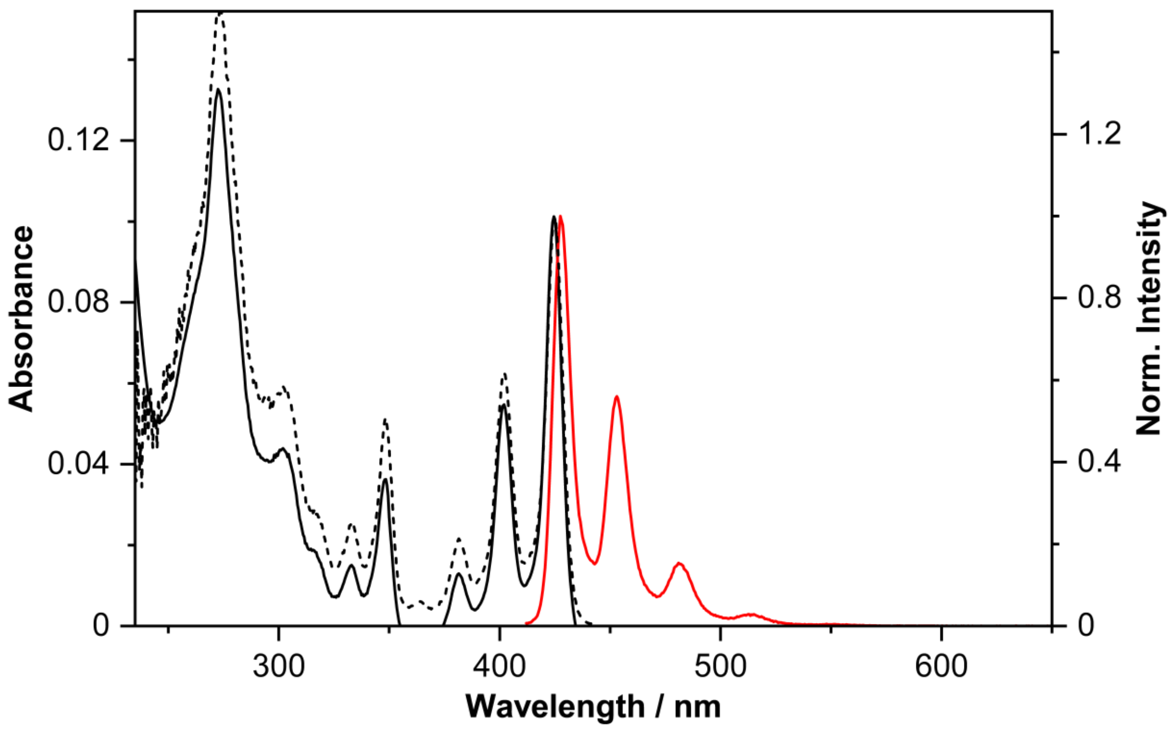

Figure S150. UV-Vis absorption spectra (black), normalized steady-state excitation (dashed, λ_{em} = 453 nm) and emission spectra (red, λ_{ex} = 402 nm) of $\mathbf{1}^{\mathbf{Ph}}$ (1.6×10^{-6} M) at 293.15 K in 2-MeTHF.

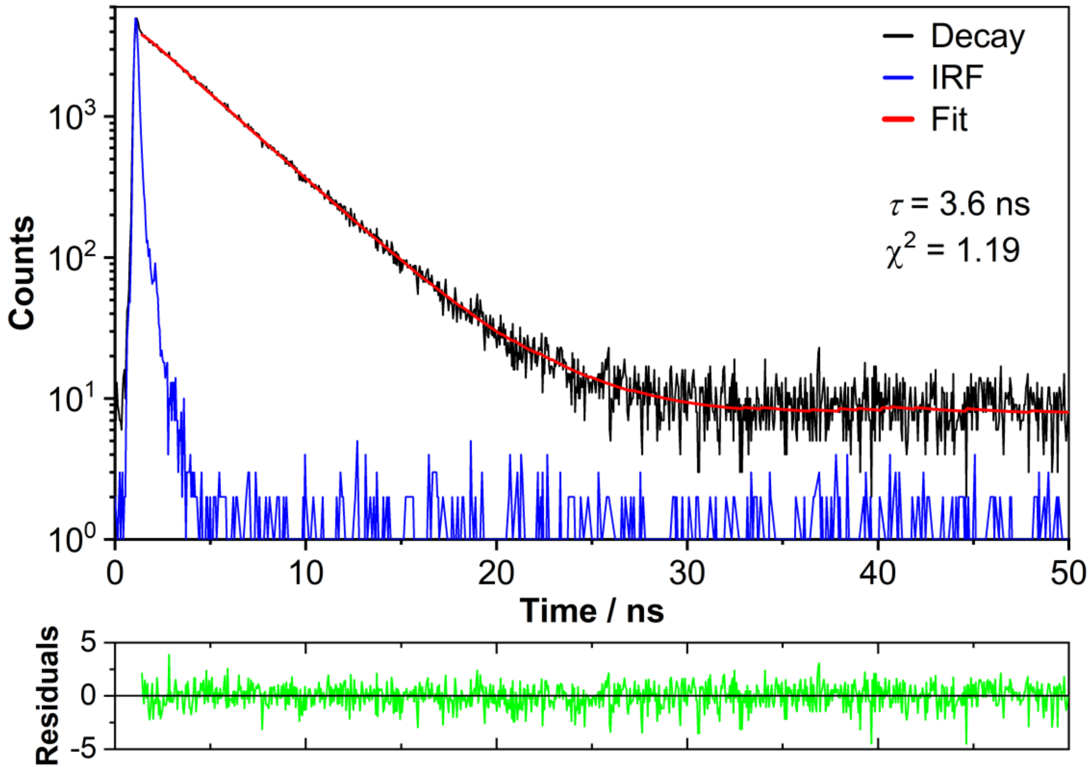

Figure S151. Time-resolved emission decay (λ_{ex} = 405.6 nm, λ_{em} = 428 nm) of $\mathbf{1}^{\mathbf{Ph}}$ (1.6×10^{-6} M) at 293.15 K in 2-MeTHF.

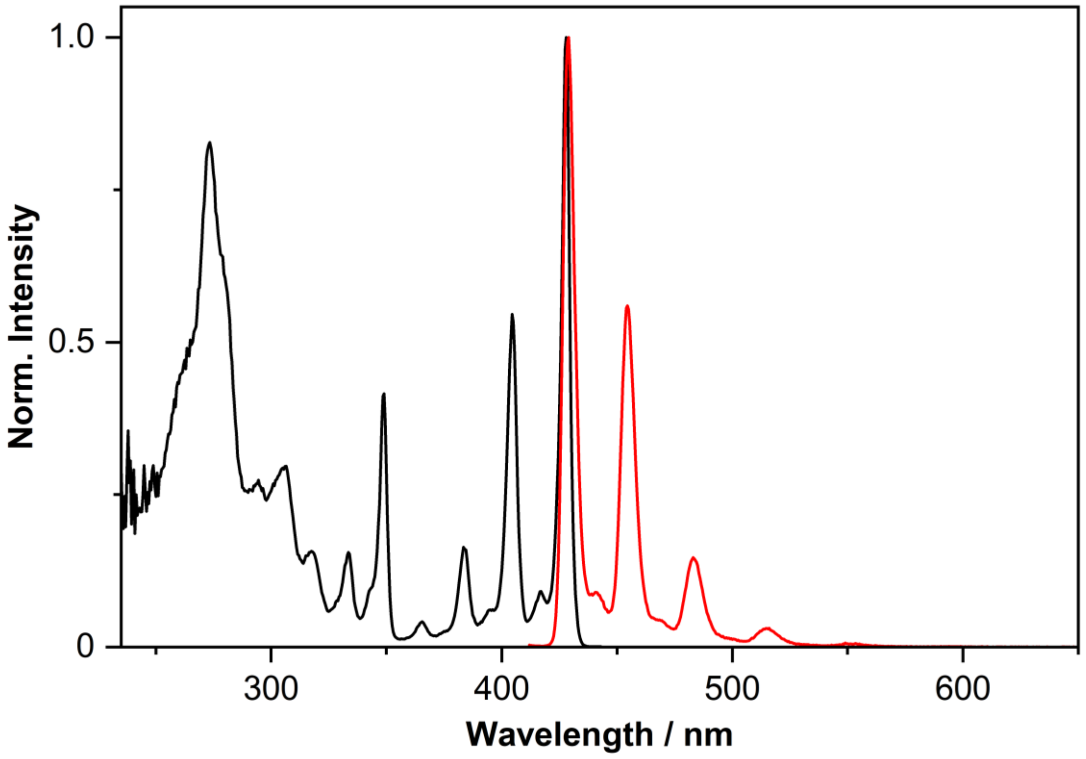

Figure S152. Normalized steady-state excitation (black, λ_{em} = 453 nm) and emission (red, λ_{ex} = 402 nm) spectra of **1**^{Ph} (1.6×10^{-6} M) at 77 K in 2-MeTHF.

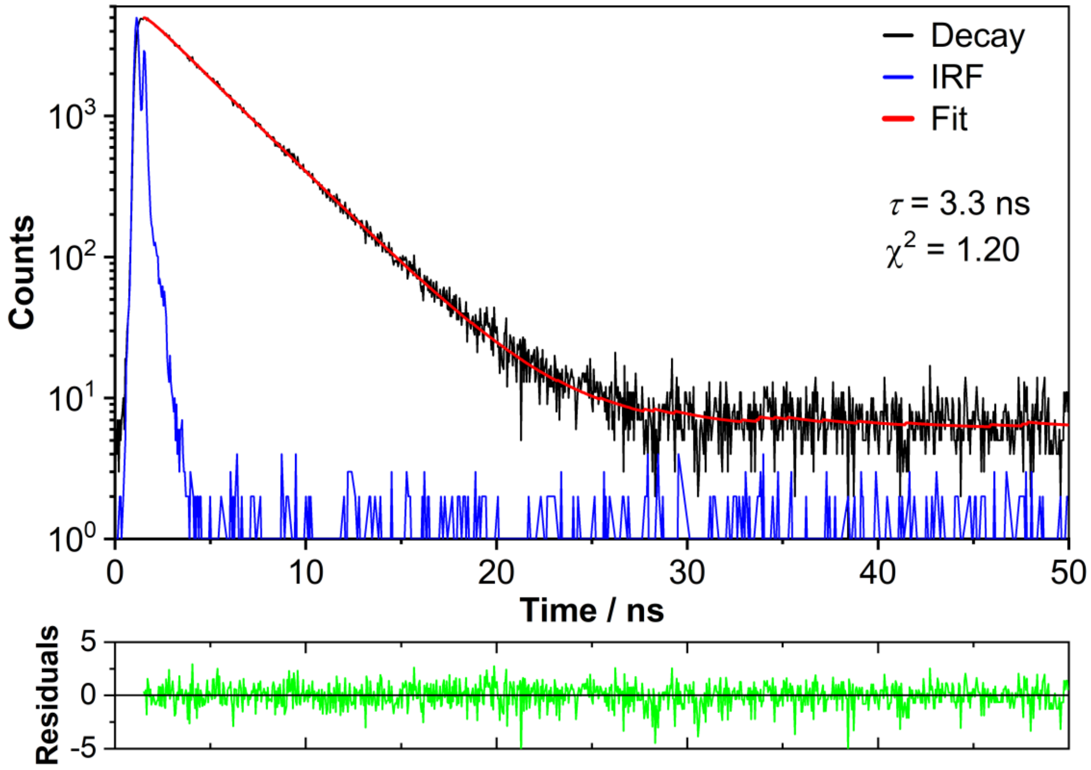

Figure S153. Time-resolved emission decay (λ_{ex} = 405.6 nm, λ_{em} = 428 nm) of **1**^{Ph} (1.6×10^{-6} M) at 77 K in 2-MeTHF.

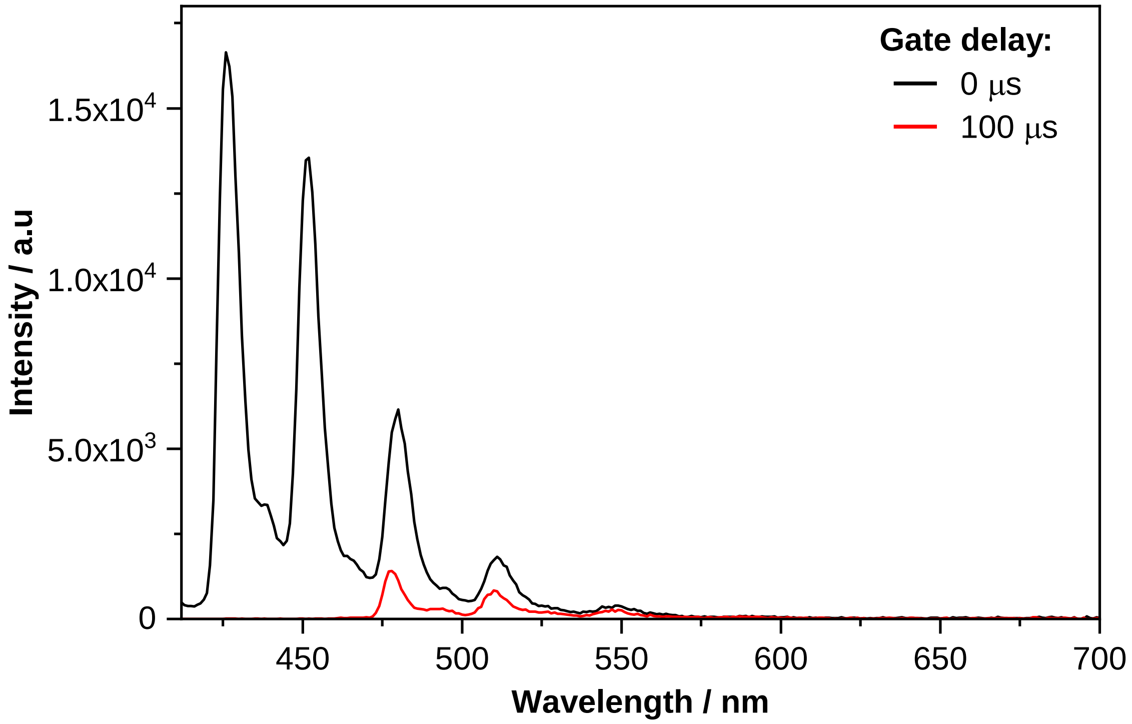

Figure S154. Time-resolved emission spectra (λ_{ex} = 402 nm) of **1^{Ph}** (1.6×10^{-6} M) at 77 K in 2-MeTHF, collected at different delay times.

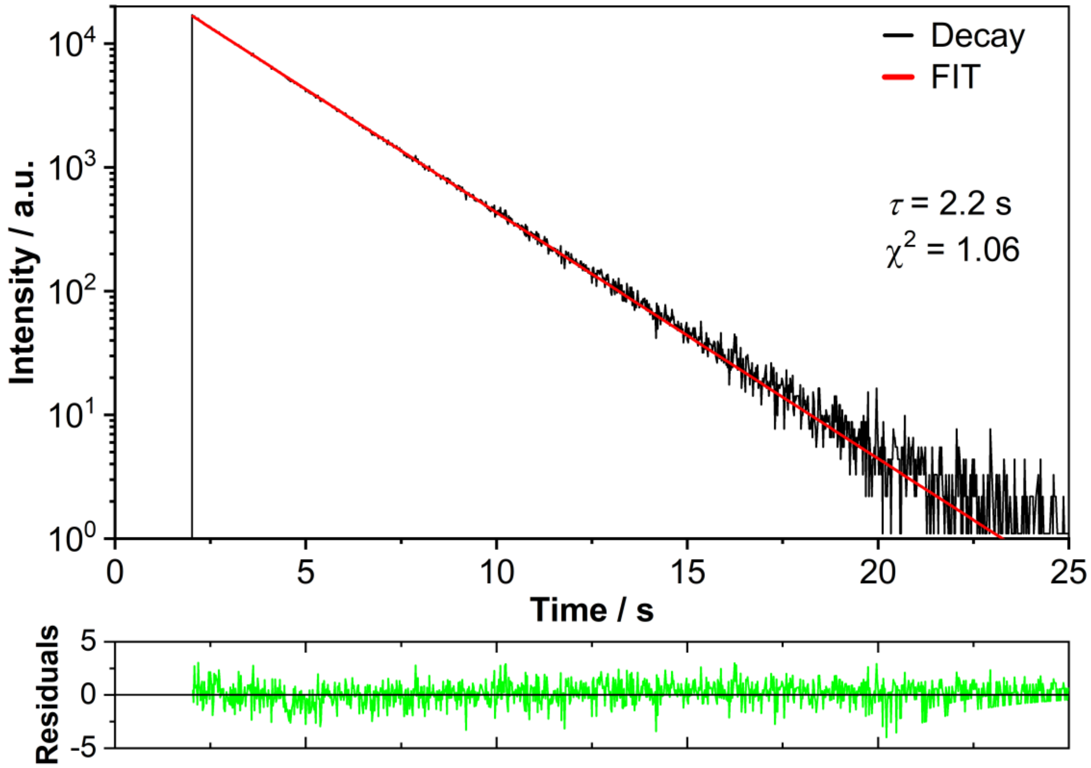

Figure S155. Time-resolved emission decay (λ_{ex} = 425 nm, λ_{em} = 480 nm) of **1^{Ph}** (1.6×10^{-6} M) at 77 K in 2-MeTHF.

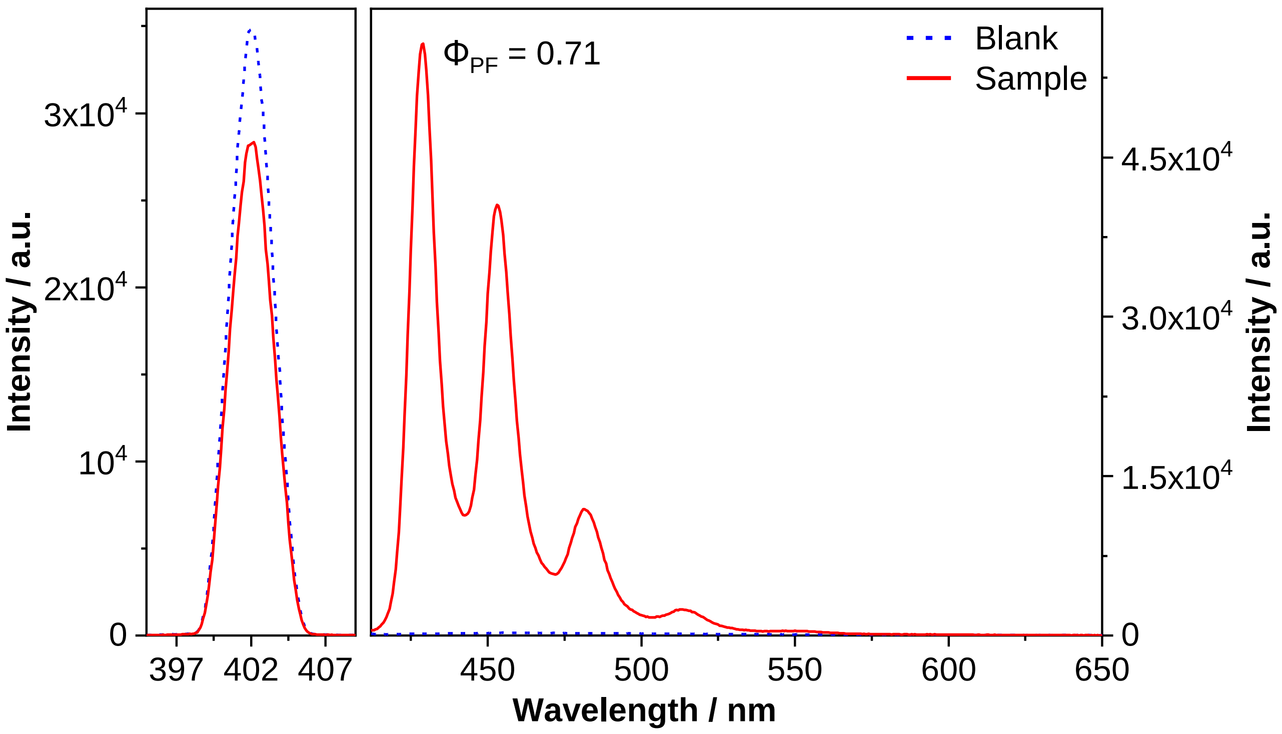

Figure S156. Excitation scatter region (left) and emission spectra (right, λ_{ex} = 402 nm) used to calculate the absolute quantum yield of **1^{Ph}** (1.6×10^{-6} M) in 2-MeTHF.

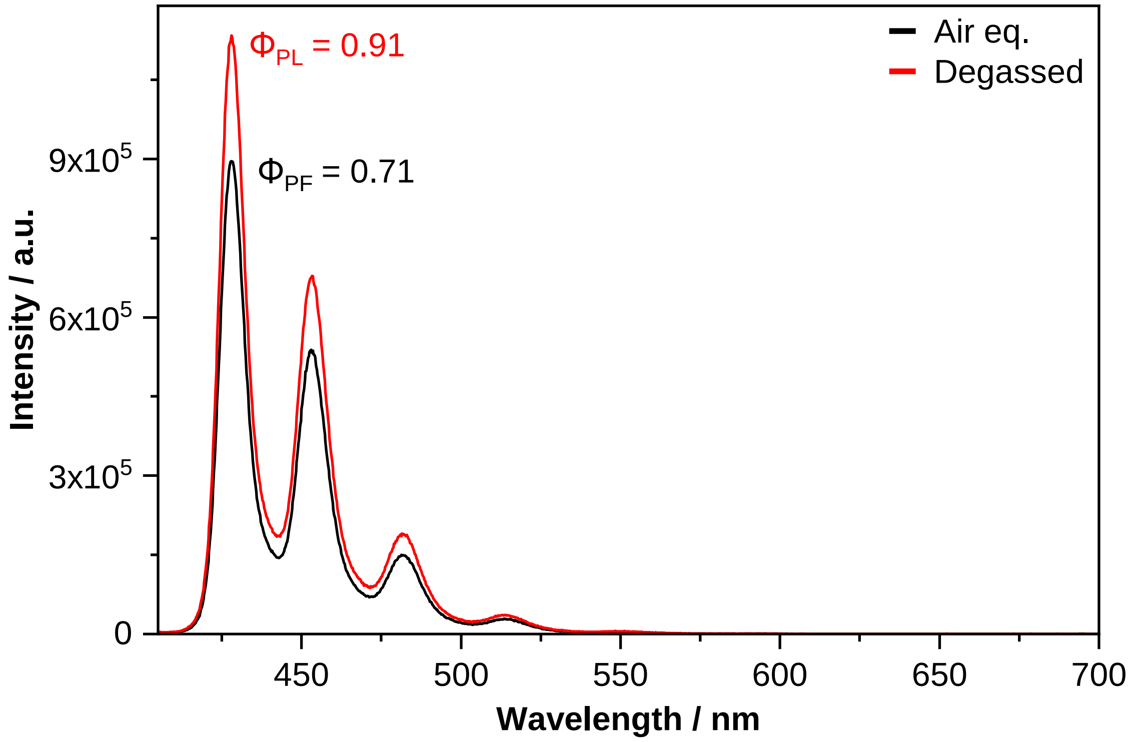

Figure S157. Steady-state emission spectra (λ_{ex} = 400 nm) of **1^{Ph}** (1.6×10^{-6} M) at 293.15 K in 2-MeTHF collected before (black) and after (red) degassing.

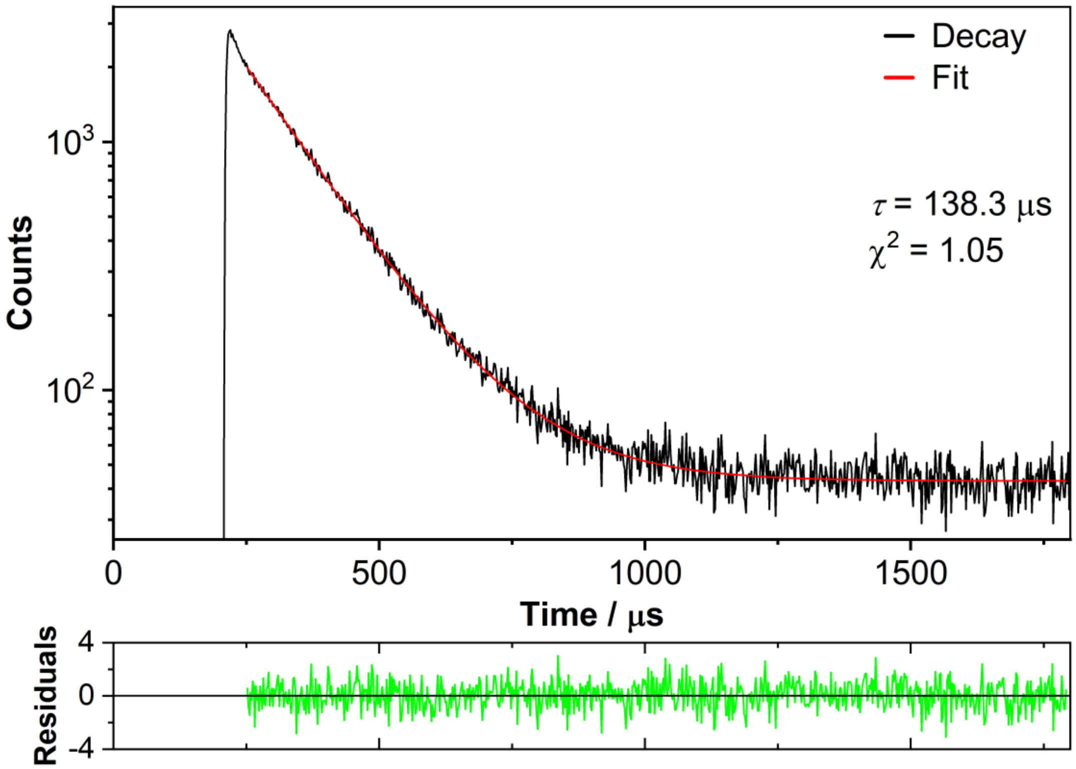

Figure S158. Time-resolved emission decay (λ_{ex} = 402 nm, λ_{em} = 428 nm, gate delay: 200 μs) of **1^{Ph}** (1.8×10^{-6} M) at 293.15 K in degassed 2-MeTHF.

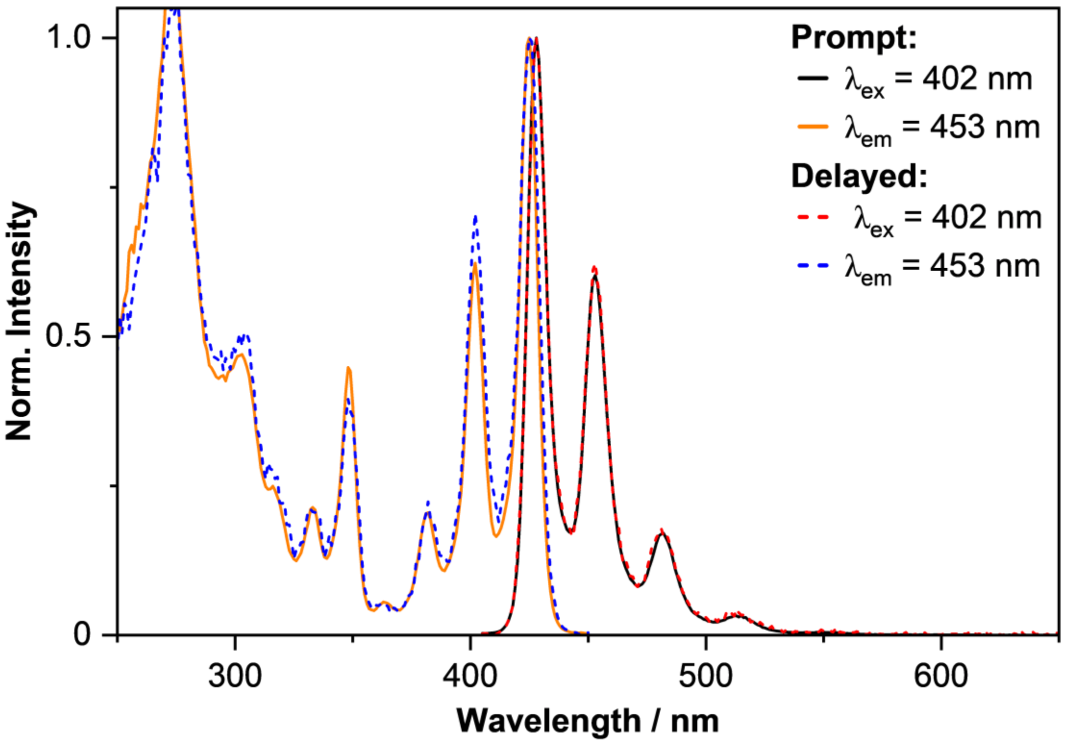

Figure S159. Comparison of the steady-state emission (black) and excitation (orange) spectra of **1^{Ph}** (1.8×10^{-6} M) at 293.15 K in degassed 2-MeTHF, with the gated (200 μs) delayed fluorescence emission (red, dashed) and excitation (blue, dashed).

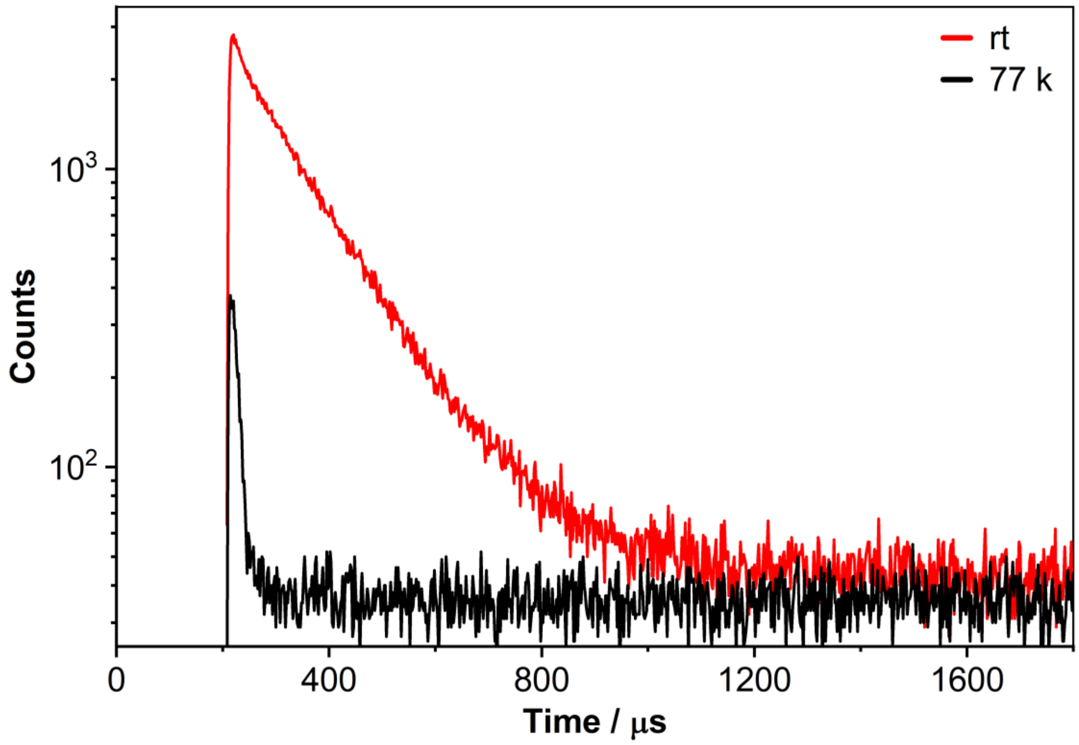

Figure S160. Time-resolved emission decay (λ_{ex} = 402 nm, λ_{em} = 428 nm, gate delay: 200 μs) of **1^{Ph}** (1.8×10^{-6} M) at 293.15 K (red) and at 77 K (black) in degassed 2-MeTHF.

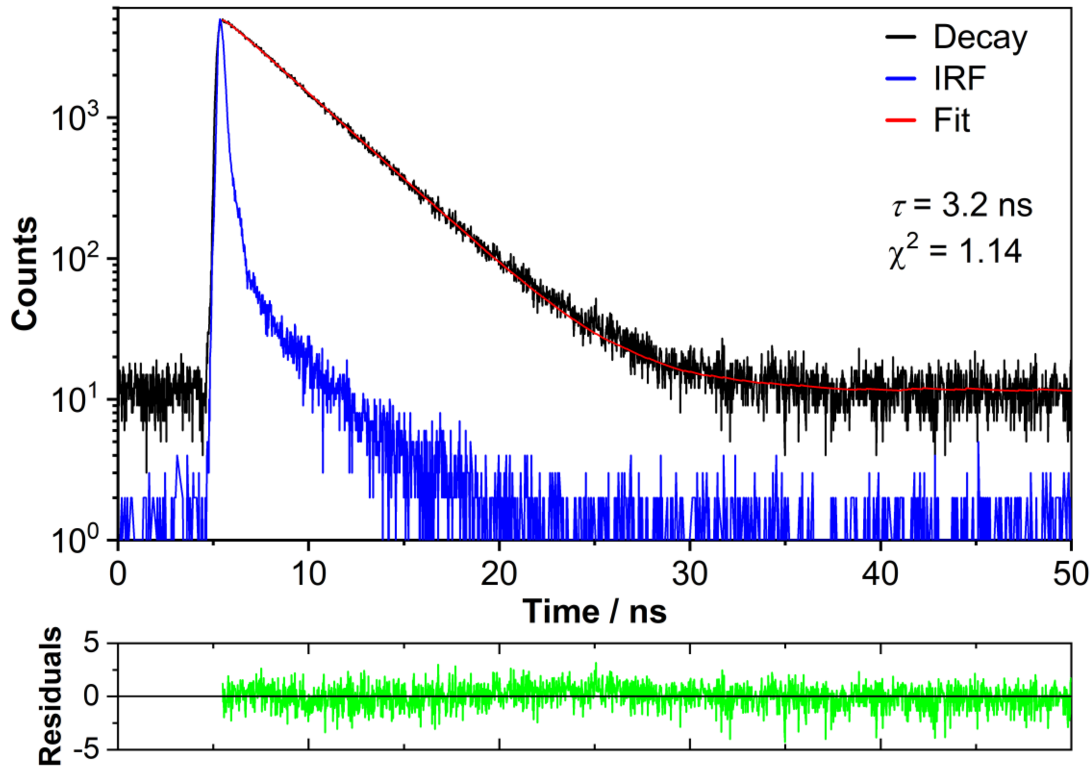

Figure S161. Time-resolved emission decay (λ_{ex} = 405.6 nm, λ_{em} = 428 nm) of **1^{Ph}** (1.8×10^{-6} M) at 293.15 K in degassed 2-MeTHF.

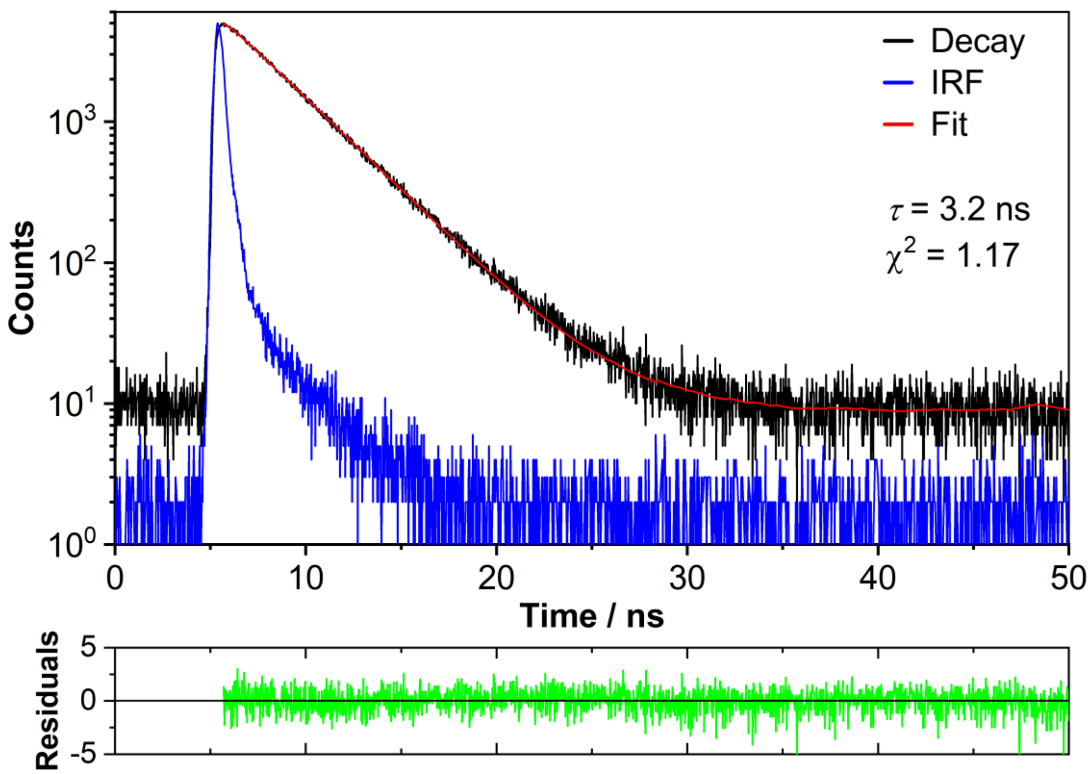

Figure S162. Time-resolved emission decay (λ_{ex} = 405.6 nm, λ_{em} = 430.5 nm) of $\mathbf{1^{Ph}}$ (1.8×10^{-6} M) at 77 K in degassed 2-MeTHF.

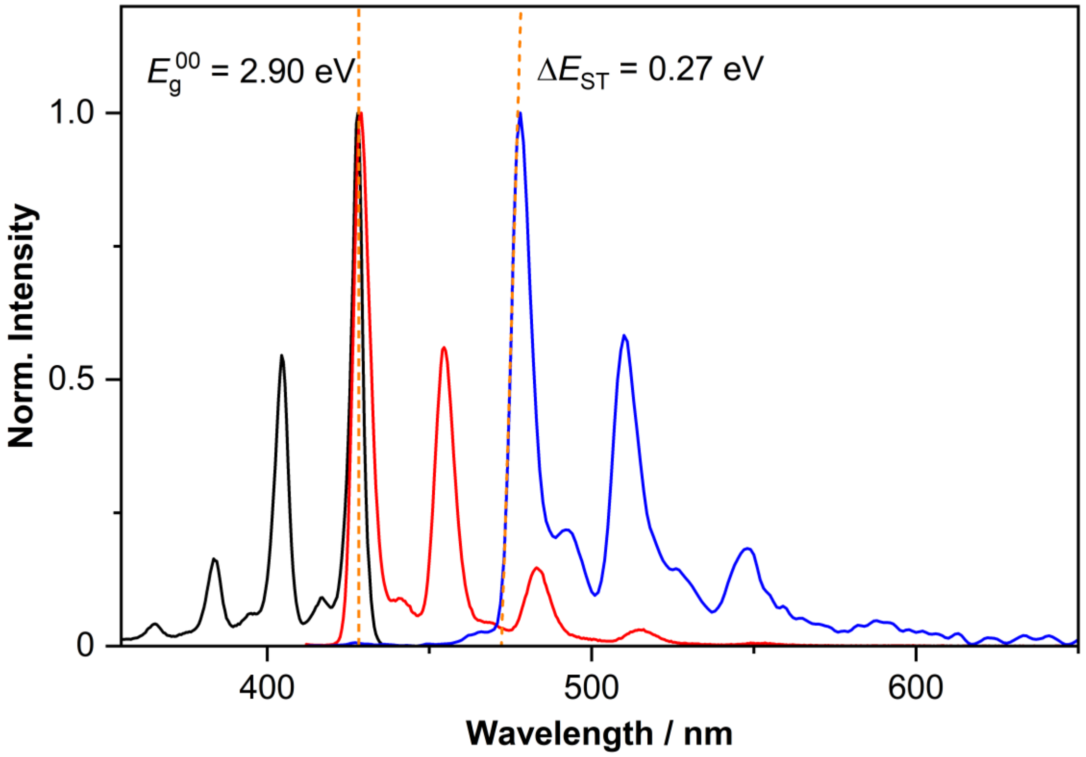

Figure S163. Determination of the optical bandgap (E_g^{00}) and the singlet-triplet gap (ΔE_{ST}) of $\mathbf{1^{Ph}}$ (1.6×10^{-6} M) at 77 K in 2-MeTHF, using the intercept of the normalized steady-state excitation (black, λ_{em} = 453 nm) and emission (red, λ_{ex} = 402 nm) spectra, and the time-resolved emission spectra (blue, λ_{ex} = 402 nm, 100 µs gate delay).

Photophysical characterization of **2**

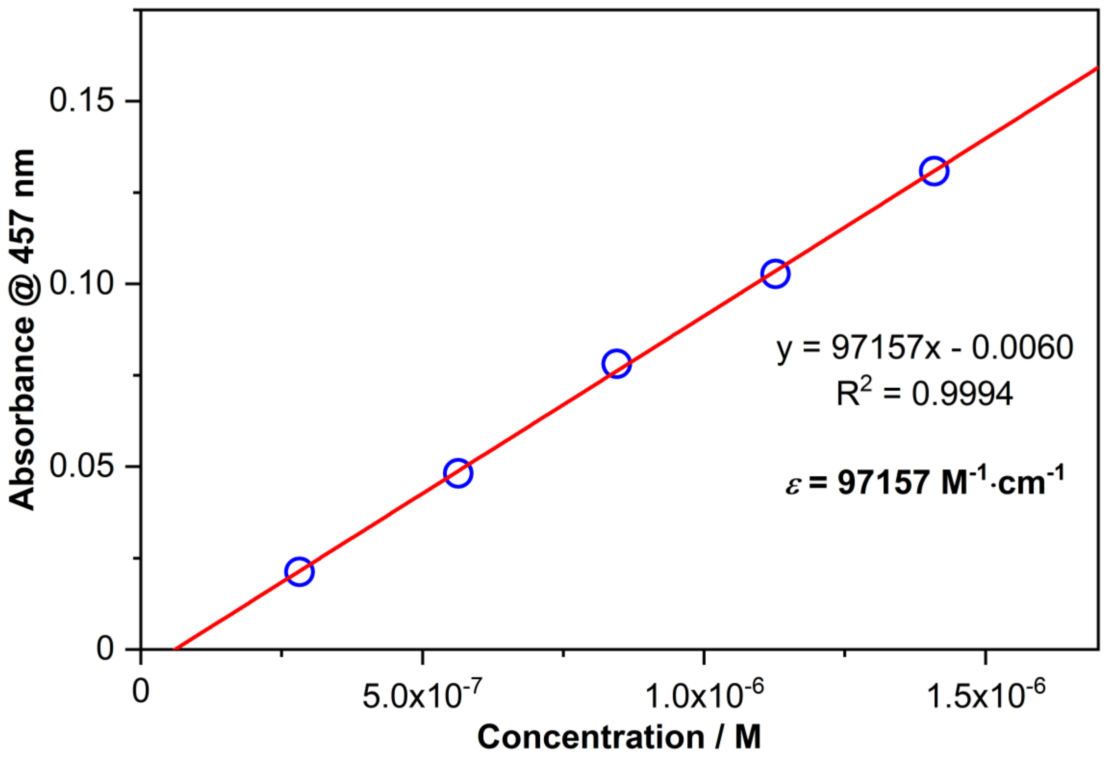

Figure S164. Determination of the molar attenuation coefficient (ε) of **2** in 2-MeTHF.

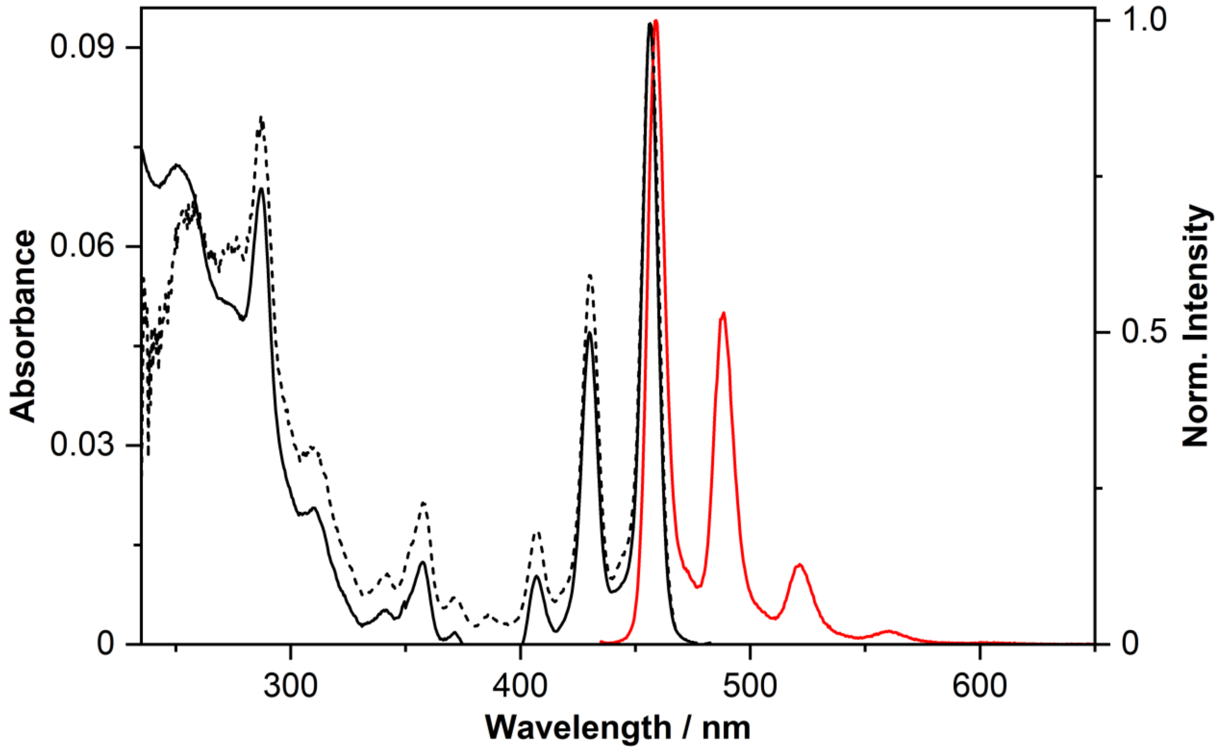

Figure S165. UV-Vis absorption (black), normalized steady-state excitation (dashed, λ_{em} = 489 nm) and emission spectra (red, λ_{ex} = 430 nm) of **2** (1.1×10^{-6} M) at 293.15 K in 2-MeTHF.

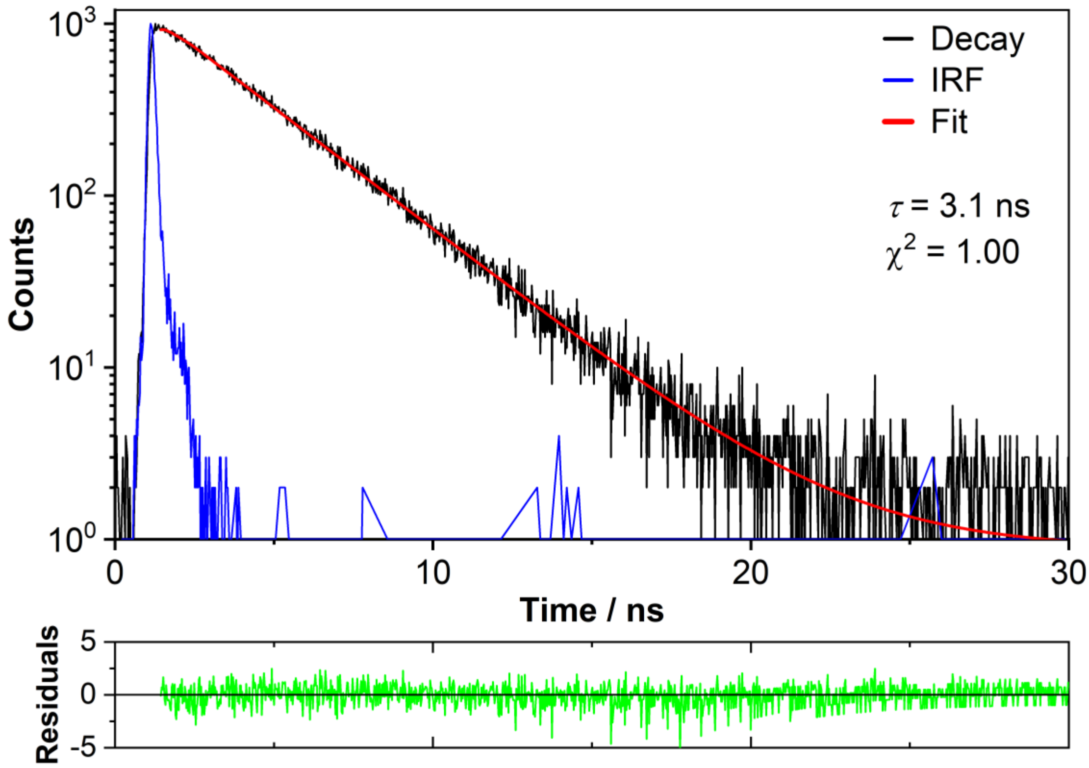

Figure S166. Time-resolved emission decay (λ_{ex} = 405.6 nm, λ_{em} = 428 nm) of **2** (1.1×10^{-6} M) at 293.15 K in 2-MeTHF.

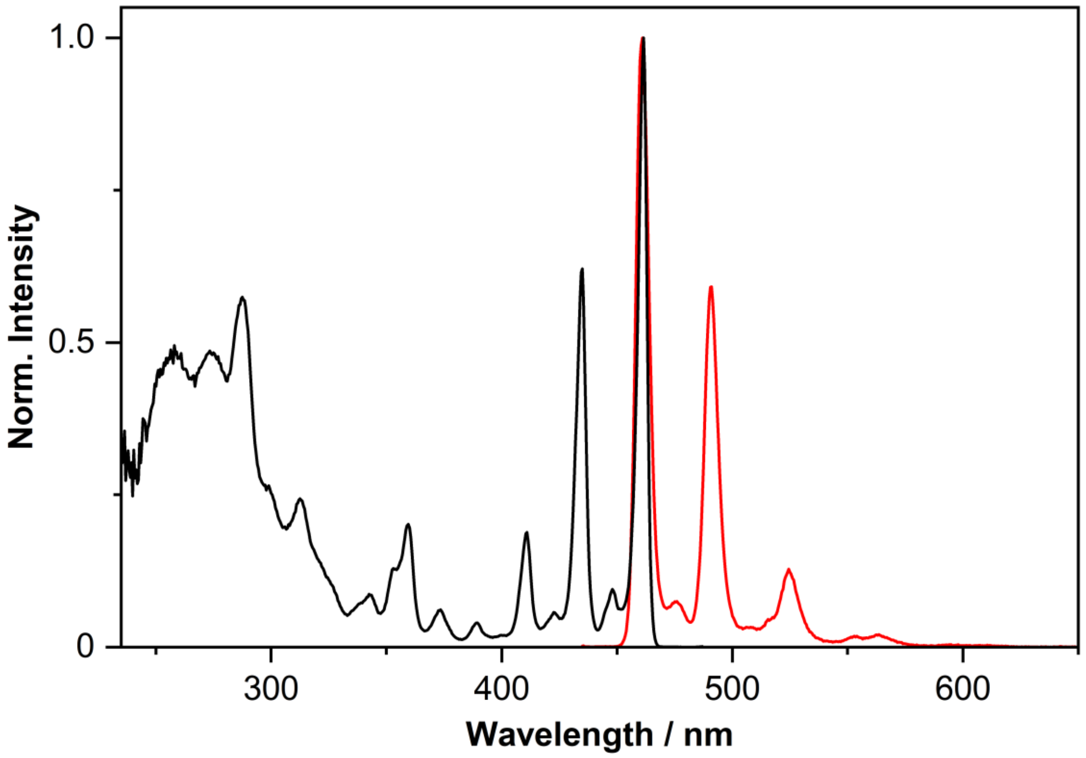

Figure S167. Normalized Steady-state excitation (dashed, λ_{em} = 492 nm) and emission (red, λ_{ex} = 430 nm) spectra of **2** (1.1×10^{-6} M) at 77 K in 2-MeTHF.

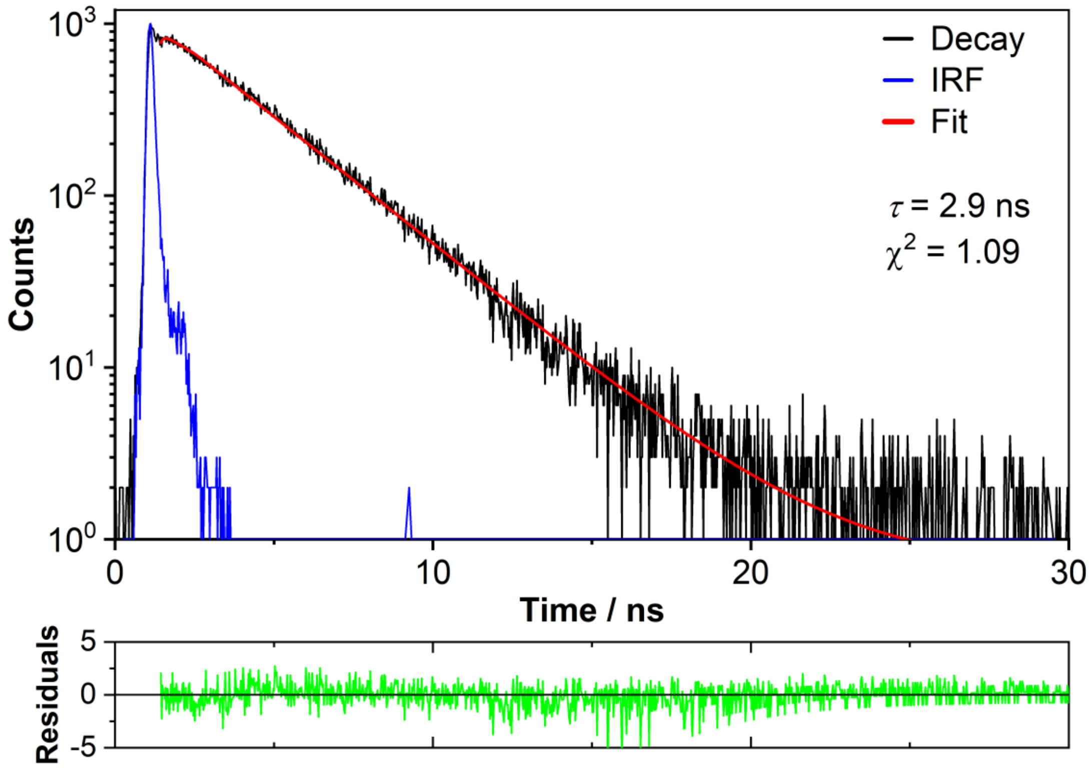

Figure S168. Time-resolved emission decay (λ_{ex} = 405.6 nm, λ_{em} = 428 nm) of **2** (1.1×10^{-6} M) at 77 K in 2-MeTHF.

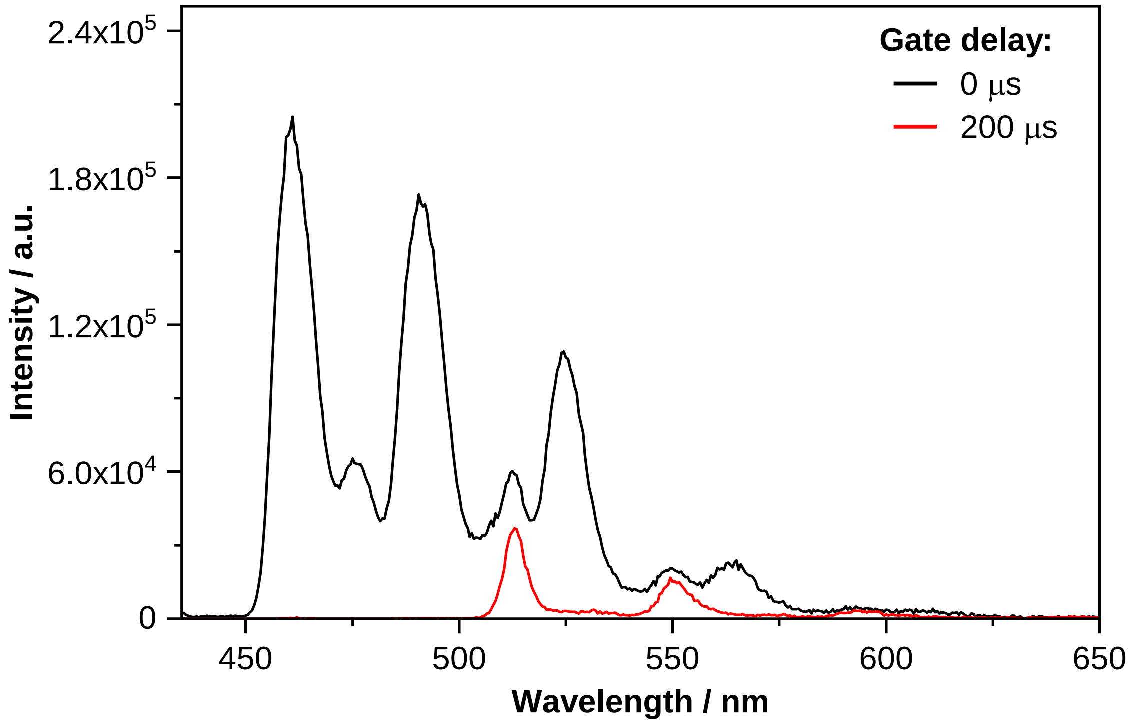

Figure S169. Time-resolved emission spectra (λ_{ex} = 430 nm) of **2** (1.1×10^{-6} M) at 77 K in 2-MeTHF, collected at different delay times.

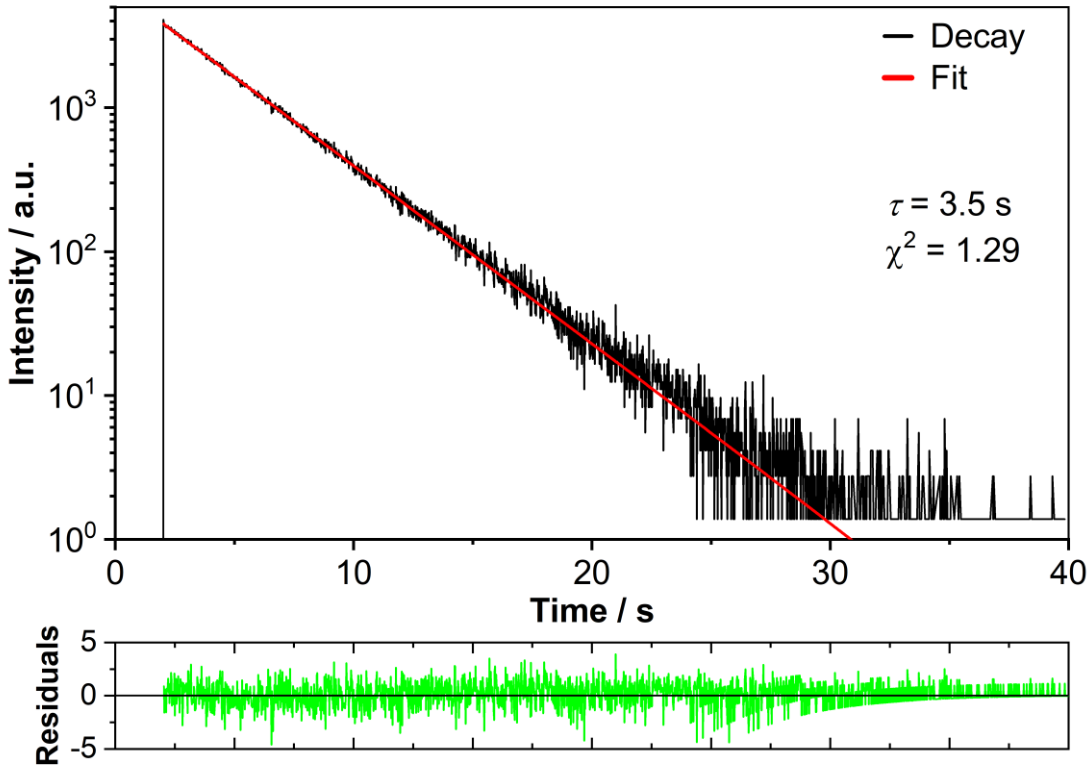

Figure S170. Time-resolved emission decay (λ_{ex} = 430 nm, λ_{em} = 513 nm) of **2** (1.1×10^{-6} M) at 77 K in 2-MeTHF.

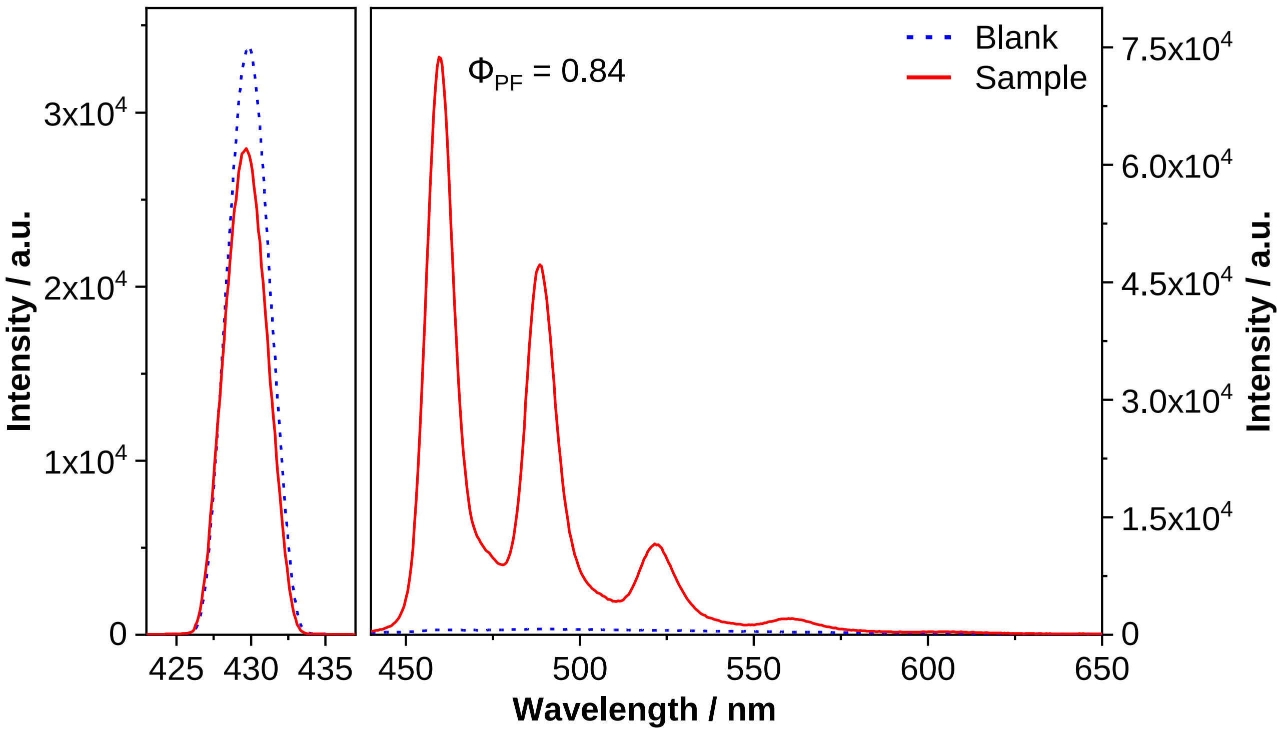

Figure S171. Excitation scatter region (left) and emission spectra (right, λ_{ex} = 430 nm) used to calculate the absolute quantum yield of **2** (1.1×10^{-6} M) in 2-MeTHF.

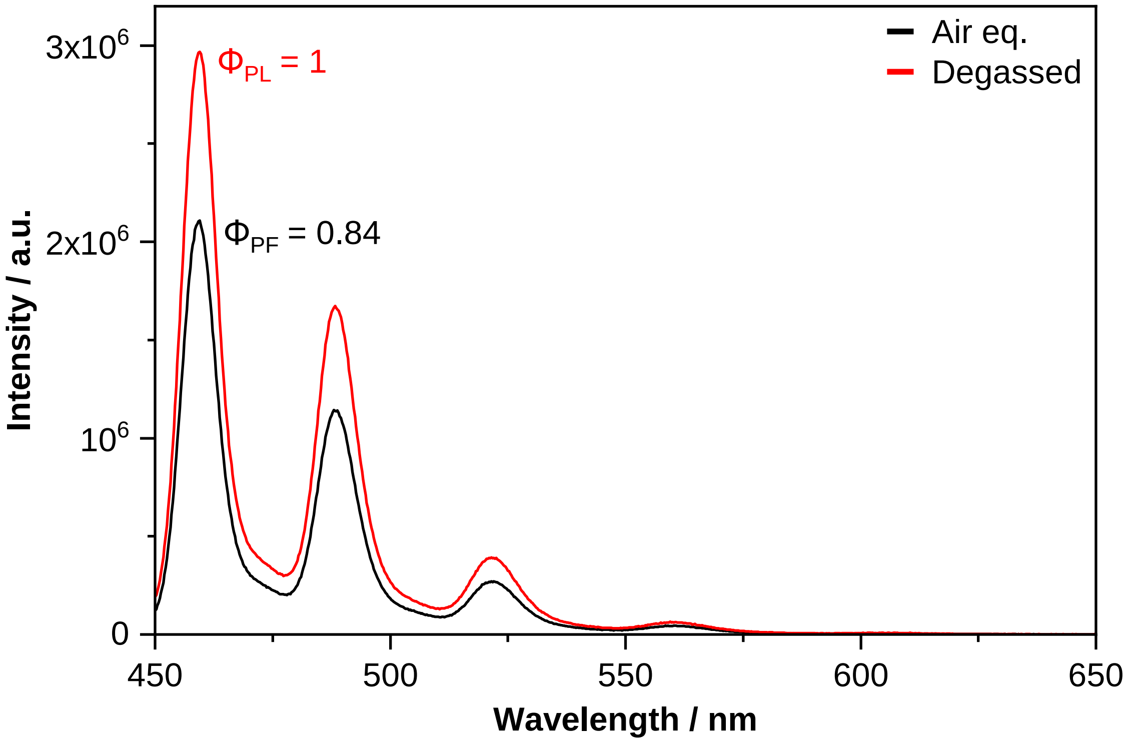

Figure S172. Steady-state emission spectra (λ_{ex} = 430 nm) of **2** (2.4×10^{-6} M) at 293.15 K in 2-MeTHF collected before (black) and after (red) degassing.

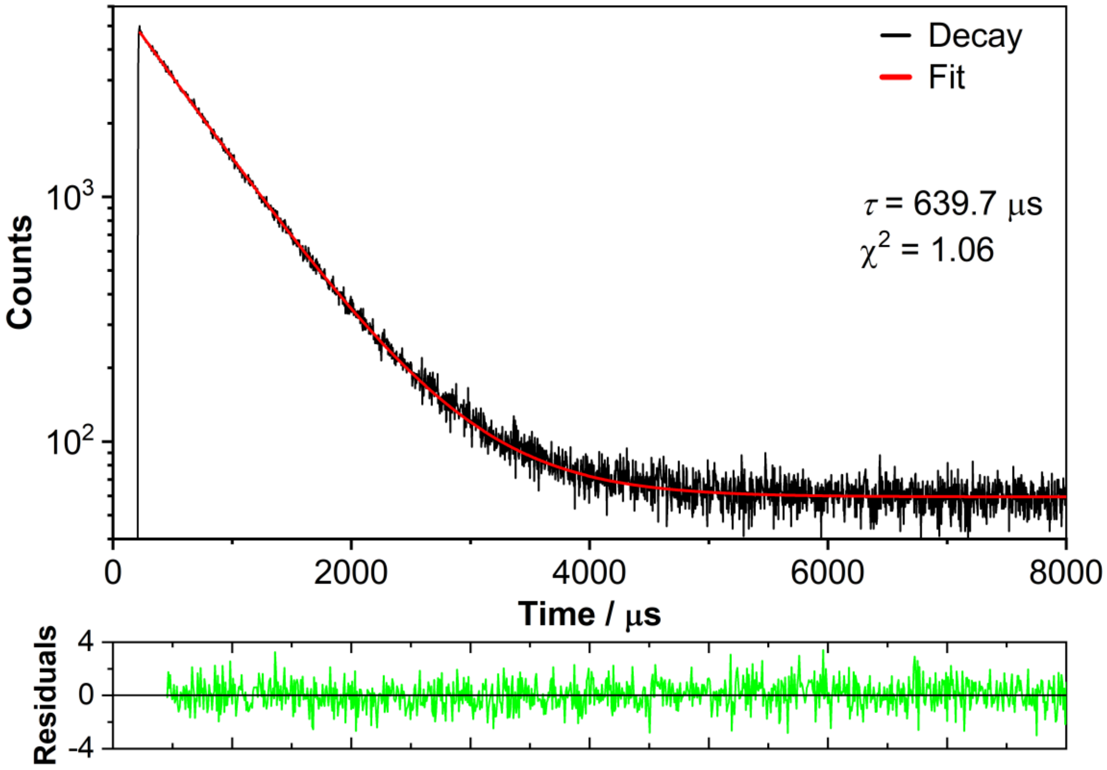

Figure S173. Time-resolved emission decay (λ_{ex} = 430.5 nm, λ_{em} = 459 nm, gate delay: 200 μs) of **2** (1.3×10^{-6} M) at 293.15 K in degassed 2-MeTHF.

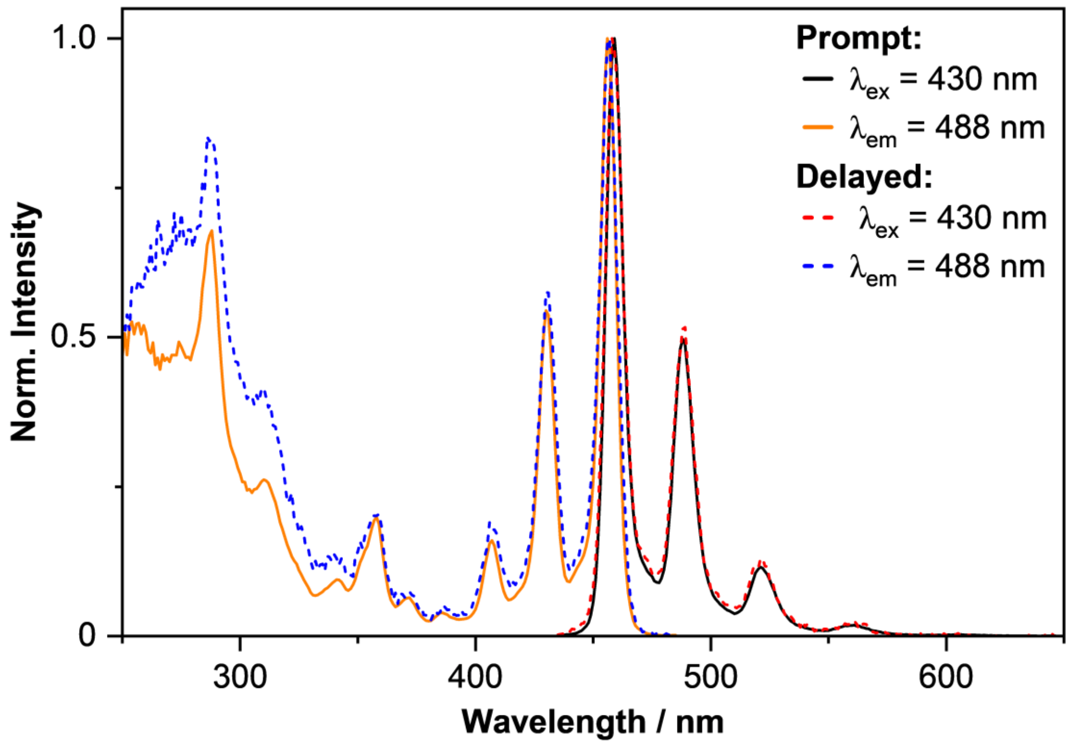

Figure S174. Comparison of the steady-state emission (black) and excitation (blue) spectra of **2** (1.3×10^{-6} M) at 293.15 K in degassed 2-MeTHF, with the gated (200 µs) delayed fluorescence emission (red, dashed) and excitation (blue, dashed).

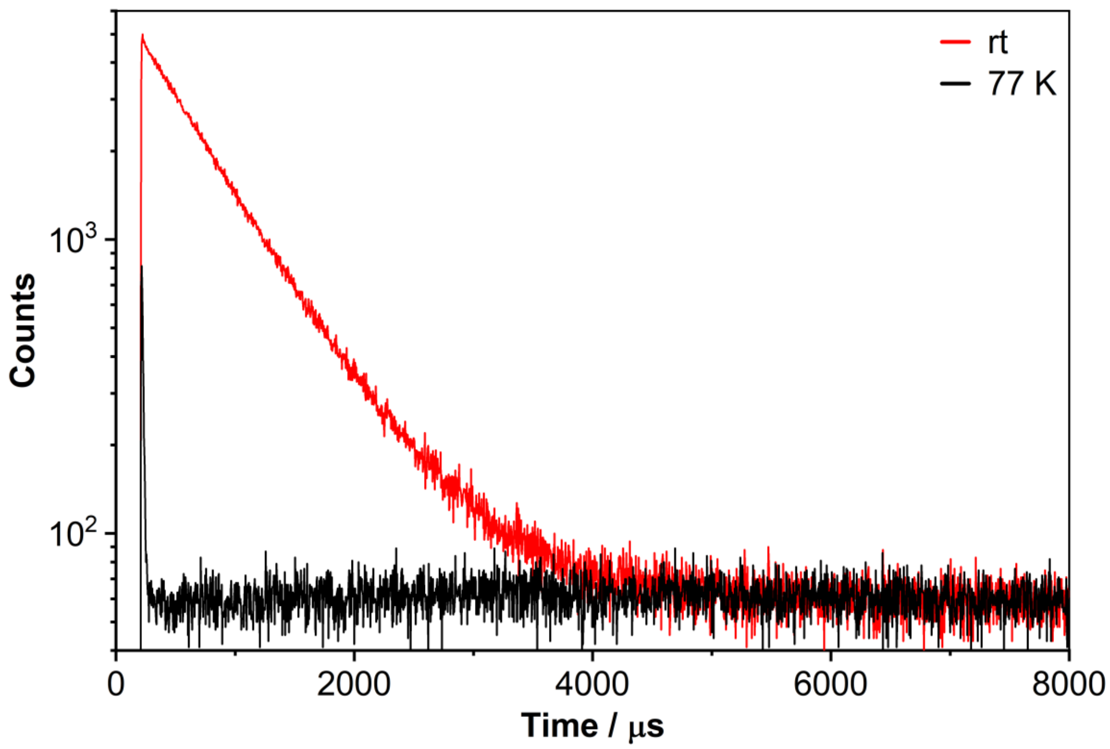

Figure S175. Time-resolved emission decay (λ_{ex} = 430.5 nm, λ_{em} = 459 nm, gate delay: 200 µs) of **2** (1.3×10^{-6} M) at 293.15 K (red) and at 77 K (black) in degassed 2-MeTHF.

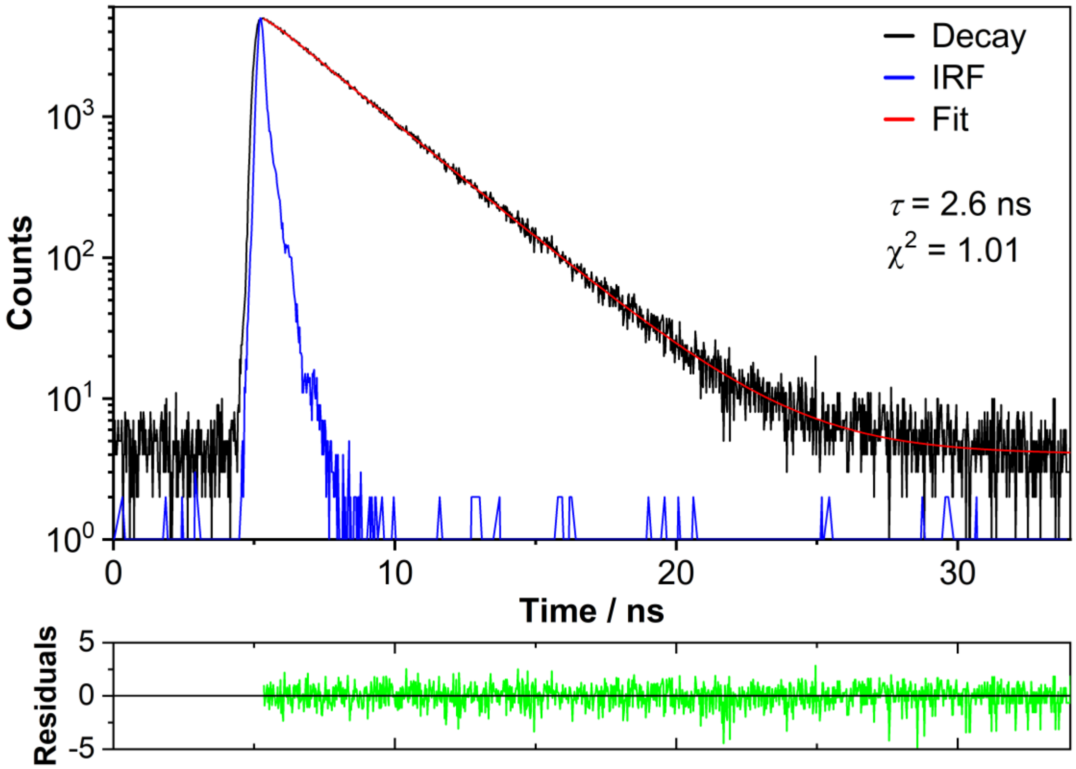

Figure S176. Time-resolved emission decay (λ_{ex} = 405.6 nm, λ_{em} = 459 nm) of **2** (1.3×10^{-6} M) at 293.15 K in degassed 2-MeTHF.

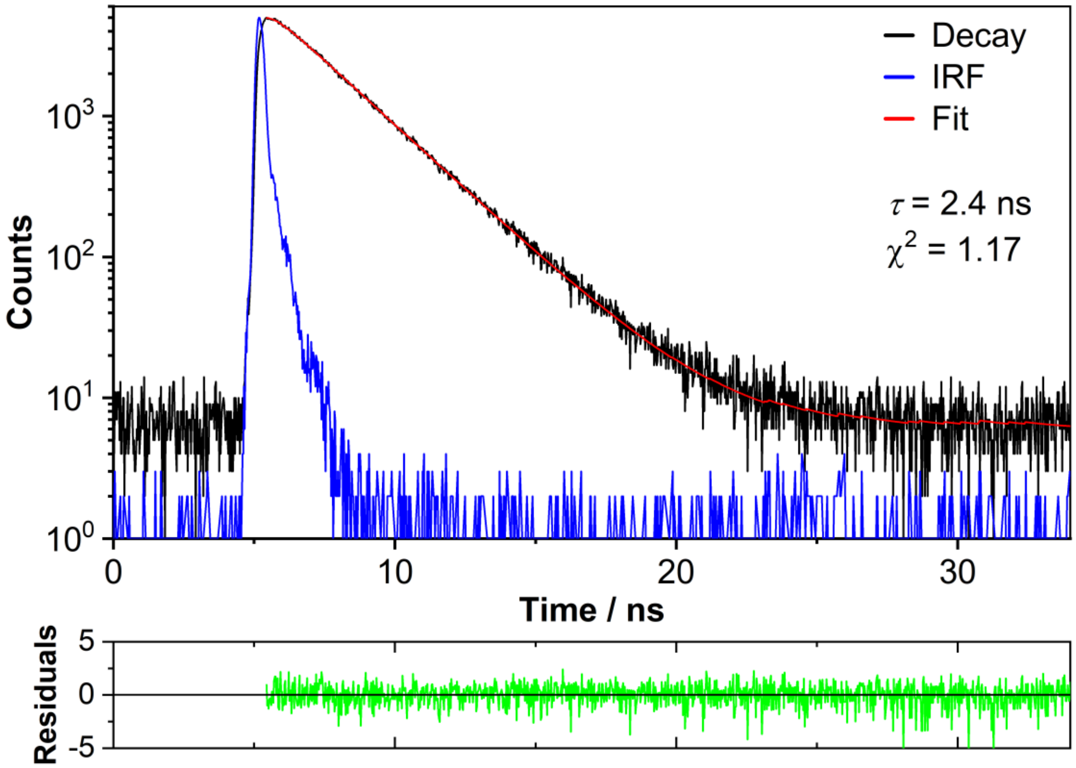

Figure S177. Time-resolved emission decay (λ_{ex} = 405.6 nm, λ_{em} = 459 nm) of **2** (1.3×10^{-6} M) at 77 K in degassed 2-MeTHF.

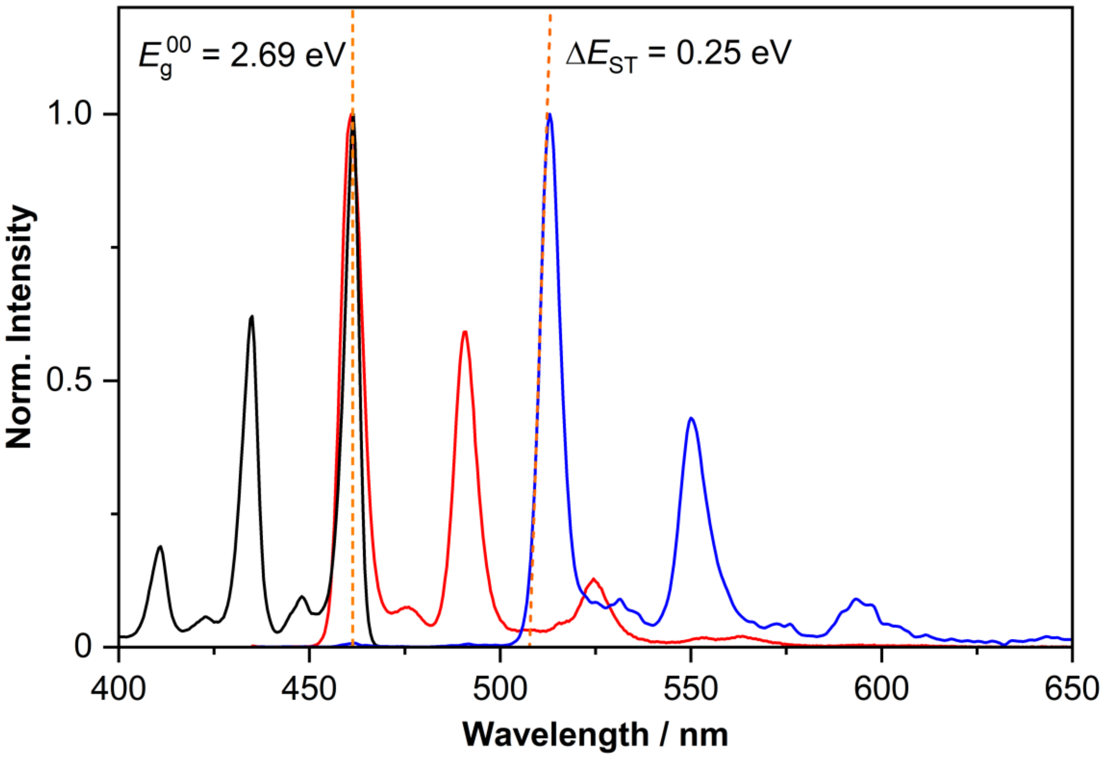

Figure S178. Determination of the optical bandgap (E_g^{00}) and the singlet-triplet gap (ΔE_{ST}) of **2** (1.1×10^{-6} M) at 77 K in 2-MeTHF, using the intercept of the normalized steady-state excitation (black, λ_{em} = 492 nm) and emission (red, λ_{ex} = 430 nm) spectra, and the time-resolved emission spectra (blue, λ_{ex} = 430 nm, 200 µs gate delay).

Photophysical characterization of **3**

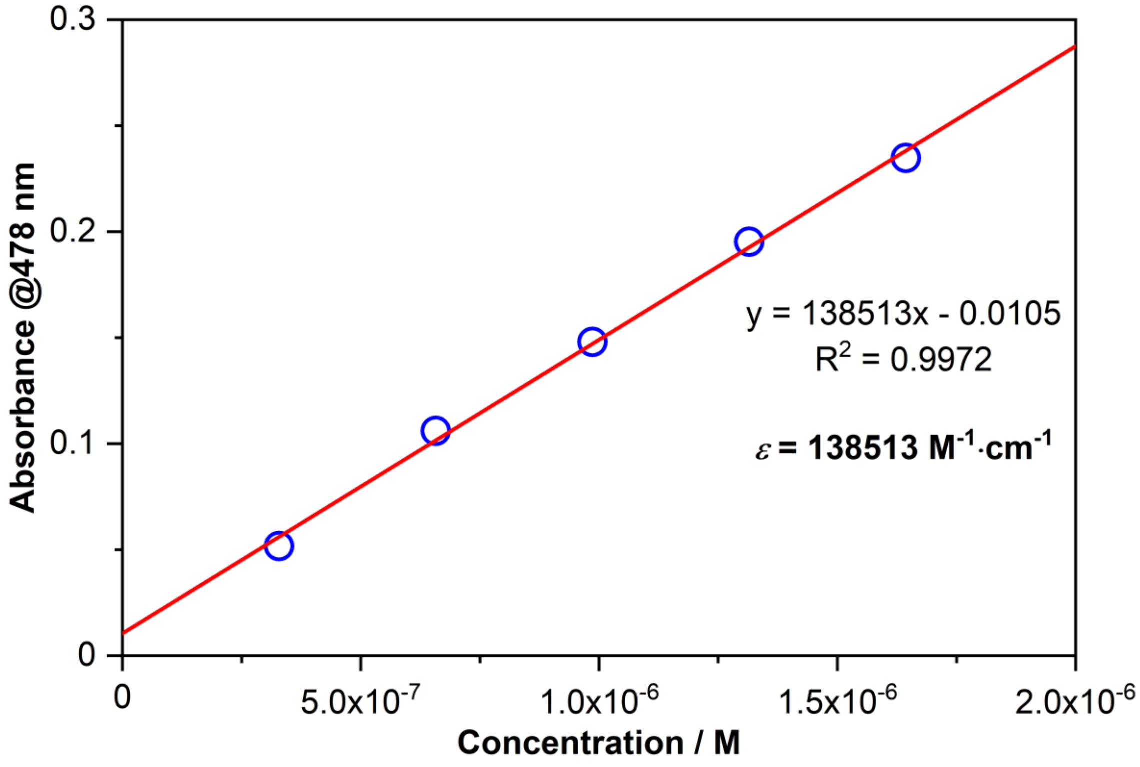

Figure S179. Determination of the molar attenuation coefficient (ε) of **3** in 2-MeTHF.

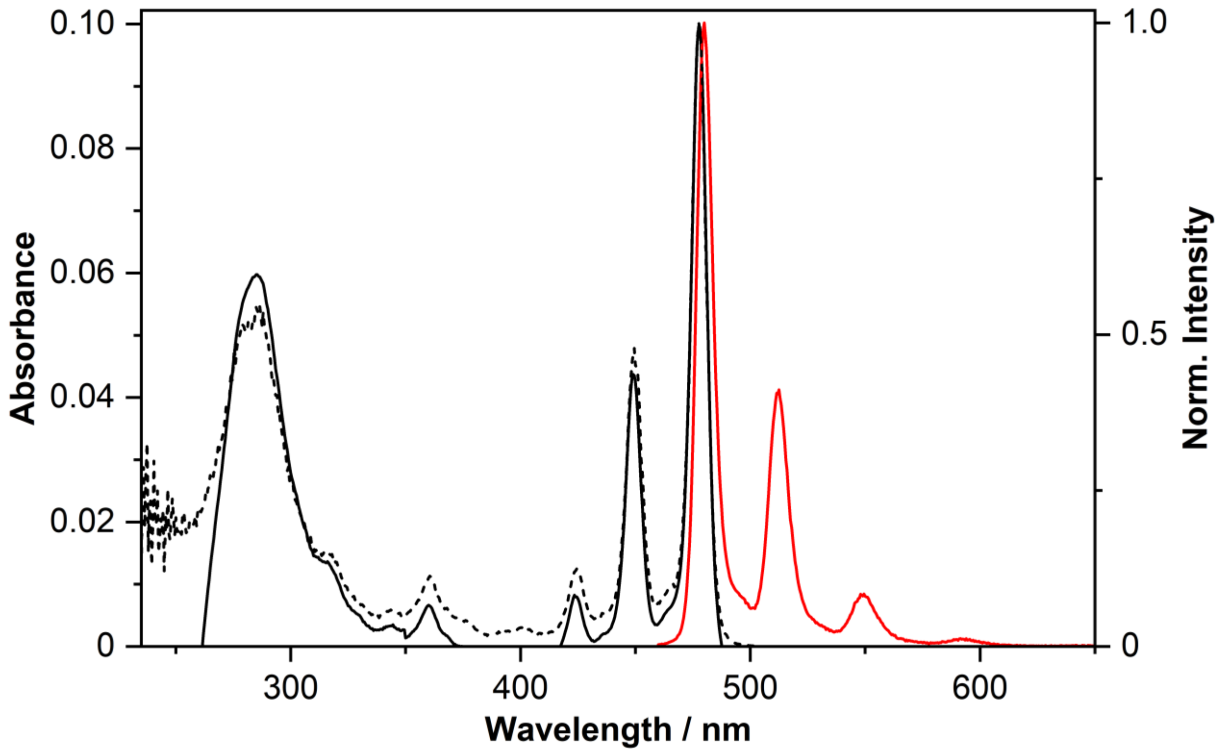

Figure S180. UV-Vis absorption spectra (black), normalized steady-state excitation (dashed, λ_{em} = 512 nm) and emission (red, λ_{ex} = 450 nm) spectra of **3** (7.3×10^{-7} M) at 293.15 K in 2-MeTHF.

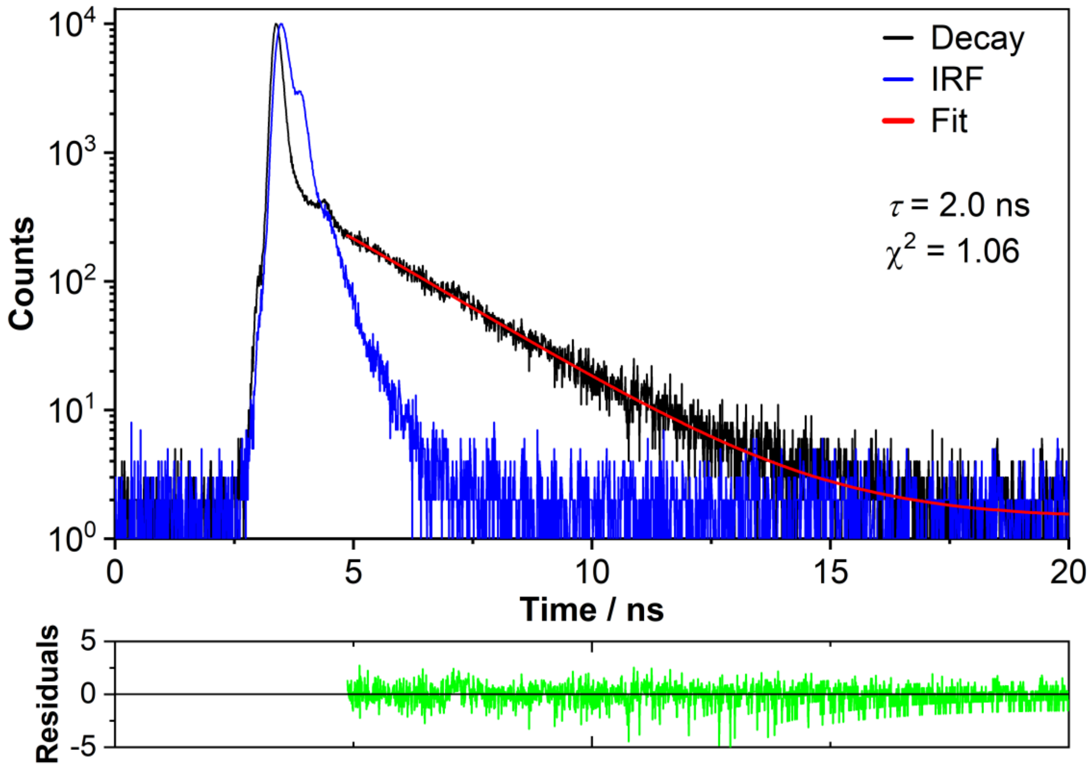

Figure S181. Time-resolved emission decay (λ_{ex} = 405.6 nm, λ_{em} = 480 nm) of **3** (7.3×10^{-7} M) at 293.15 K in 2-MeTHF.

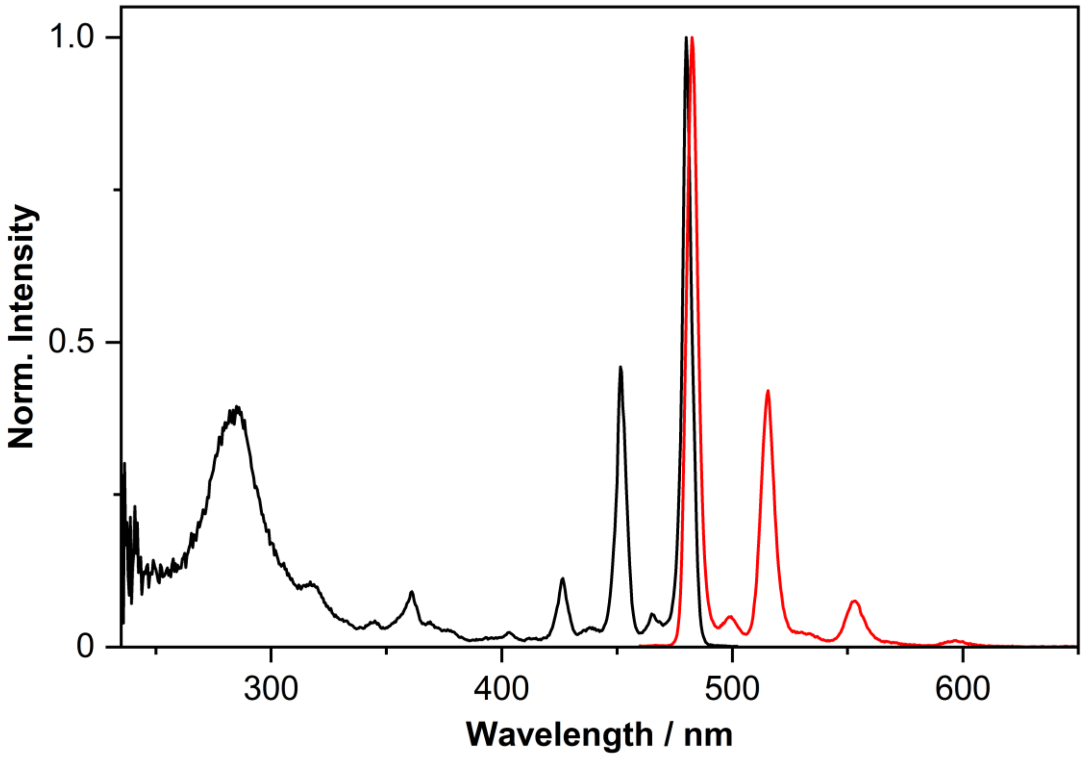

Figure S182. Normalized steady-state excitation (black, λ_{em} = 512 nm) and emission (red, λ_{ex} = 450 nm) spectra of **3** (7.3×10^{-7} M) at 77 K in 2-MeTHF.

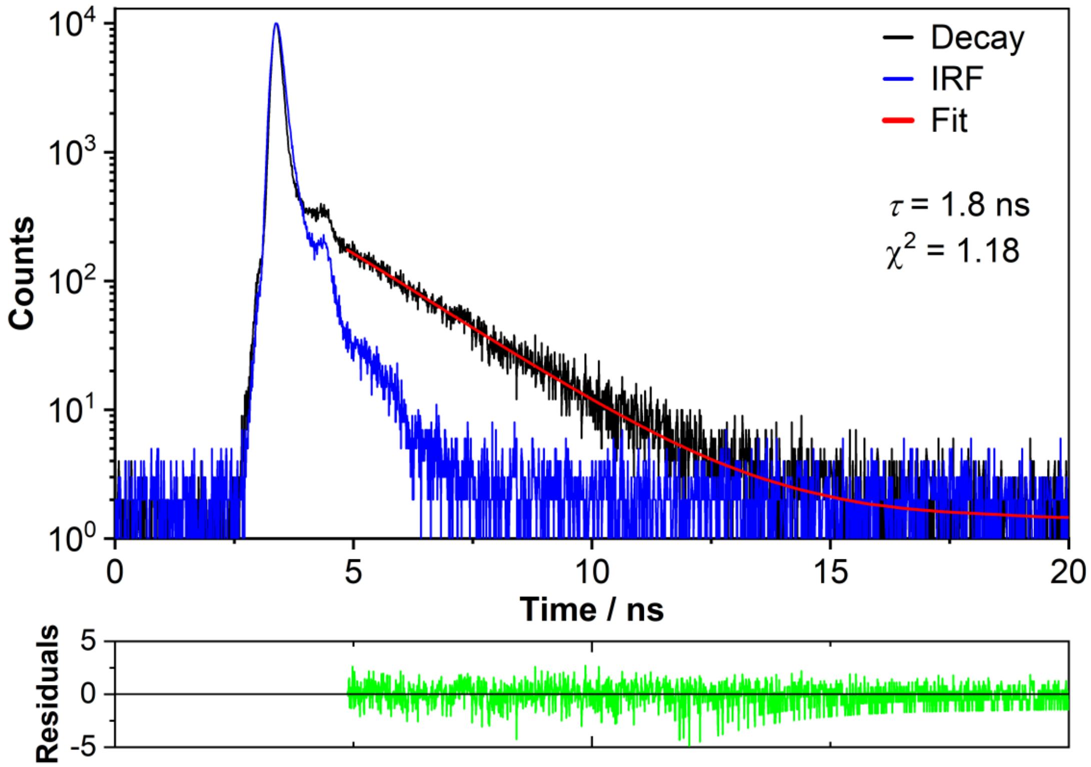

Figure S183. Time-resolved emission decay (λ_{ex} = 405.6 nm, λ_{em} = 480 nm) of **3** (7.3×10^{-7} M) at 77 K in 2-MeTHF.

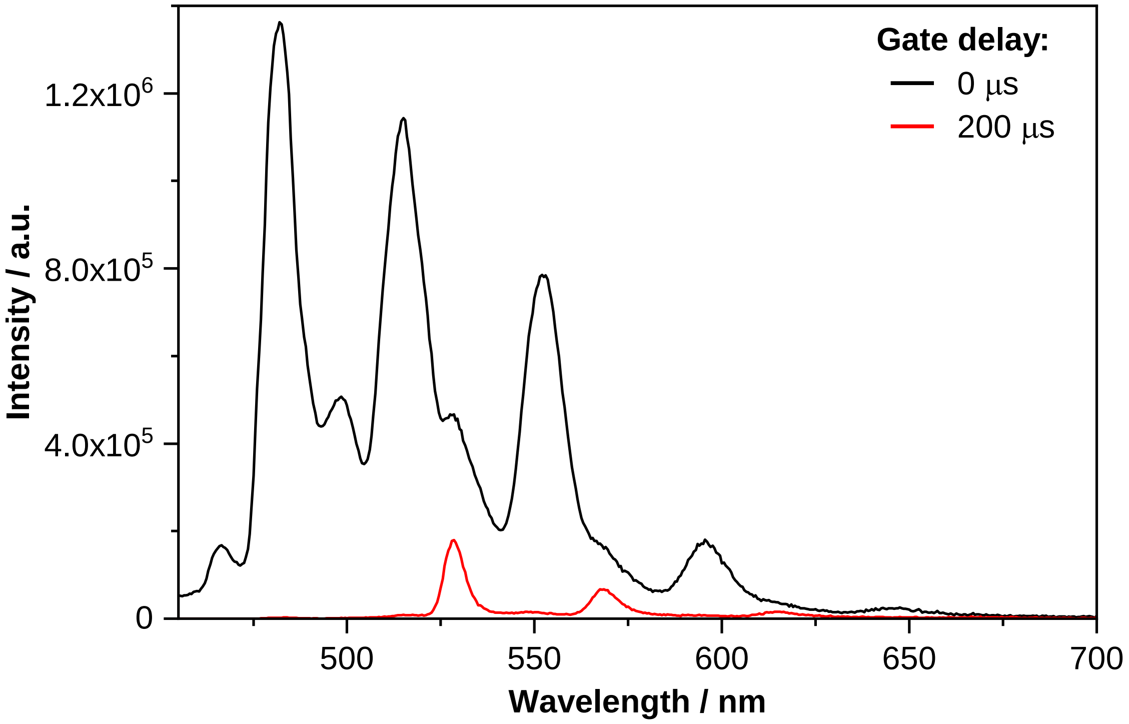

Figure S184. Time-resolved emission spectra (λ_{ex} = 450 nm) of **3** (7.3×10^{-7} M) at 77 K in 2-MeTHF, collected at different delay times.

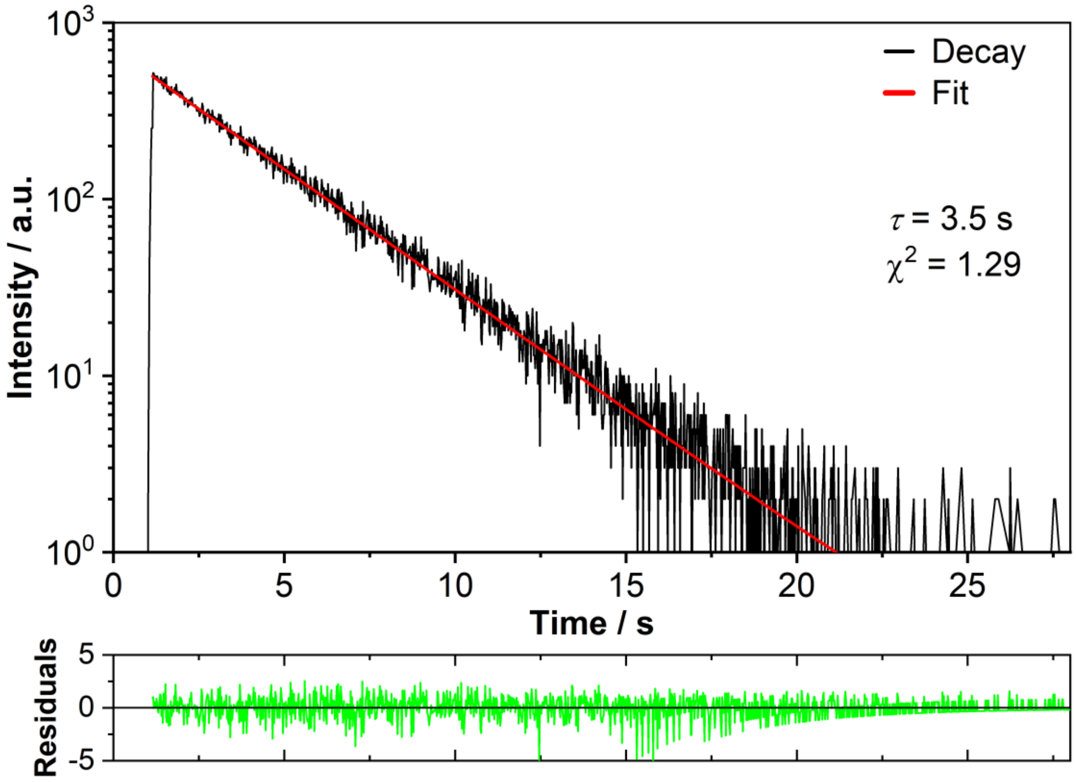

Figure S185. Time-resolved emission decay (λ_{ex} = 450 nm, λ_{em} = 529 nm) of **3** (7.3×10^{-7} M) at 77 K in 2-MeTHF.

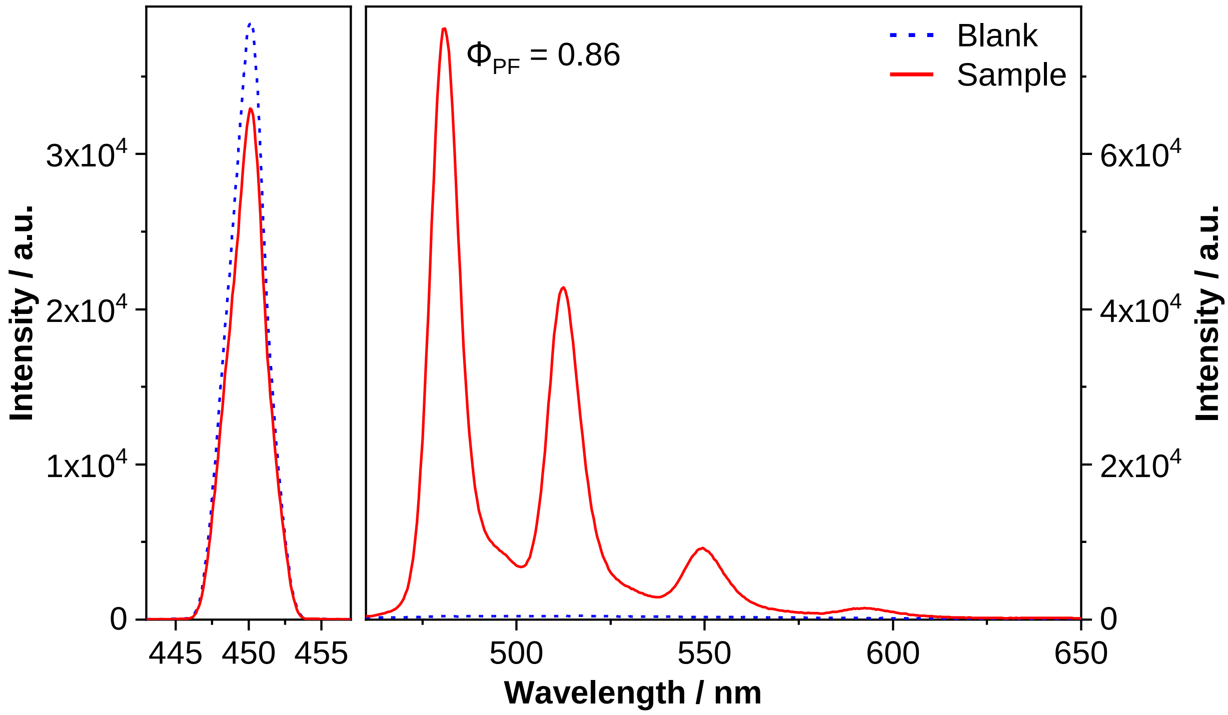

Figure S186. Excitation scatter region (left) and emission spectra (right, λ_{ex} = 450 nm) used to calculate the absolute quantum yield of **3** (7.3×10^{-7} M) in 2-MeTHF.

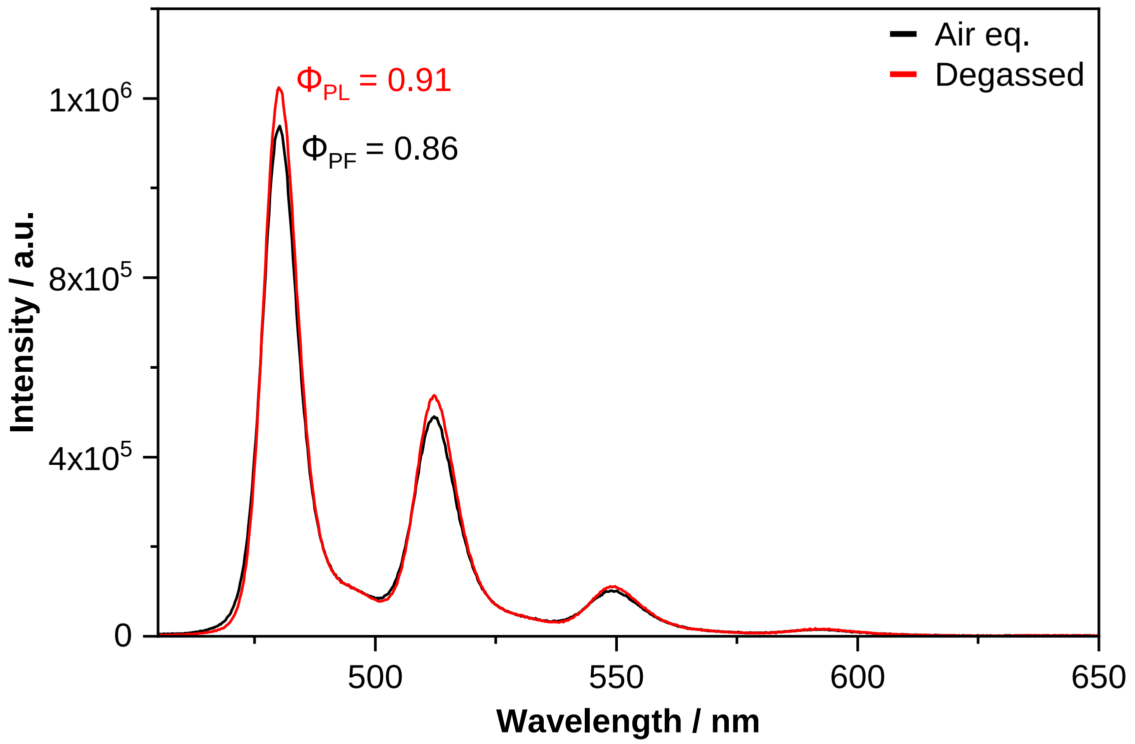

Figure S187. Steady-state emission spectra (λ_{ex} = 318 nm) of **3** (6.1×10^{-7} M) at 293.15 K in 2-MeTHF collected before (black) and after (red) degassing.

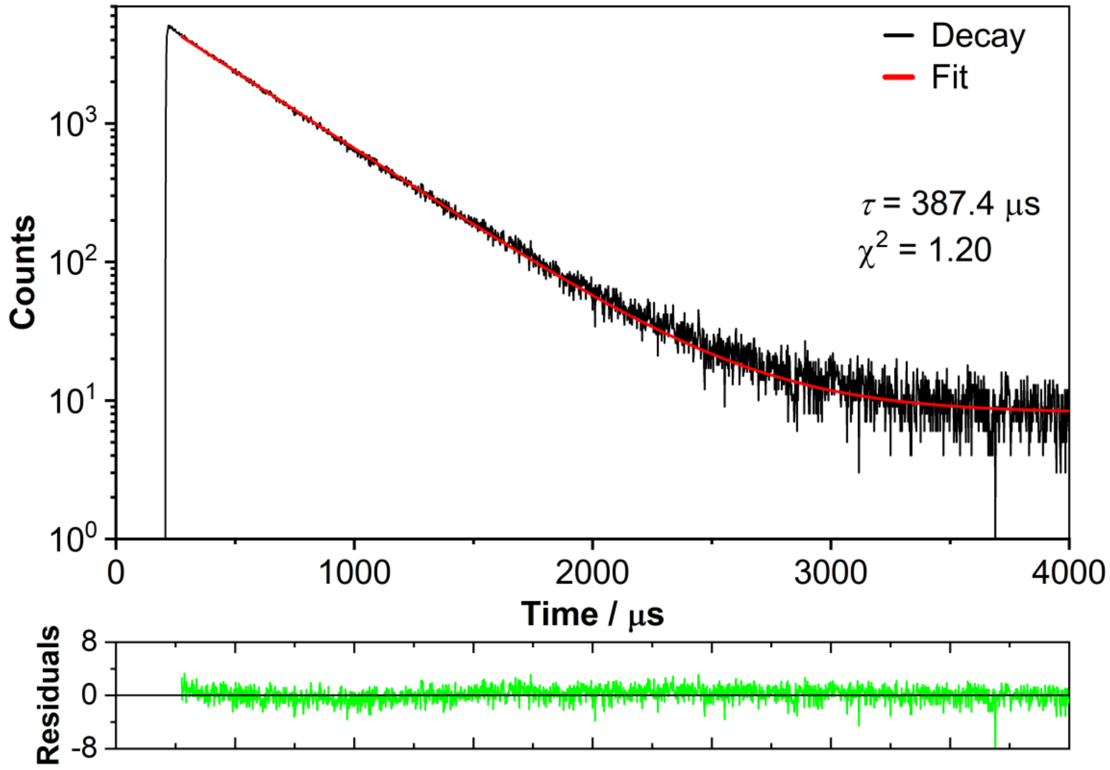

Figure S188. Time-resolved emission decay (λ_{ex} = 450 nm, λ_{em} = 480 nm, gate delay: 200 μs) of **3** (7.6×10^{-7} M) at 293.15 K in degassed 2-MeTHF.

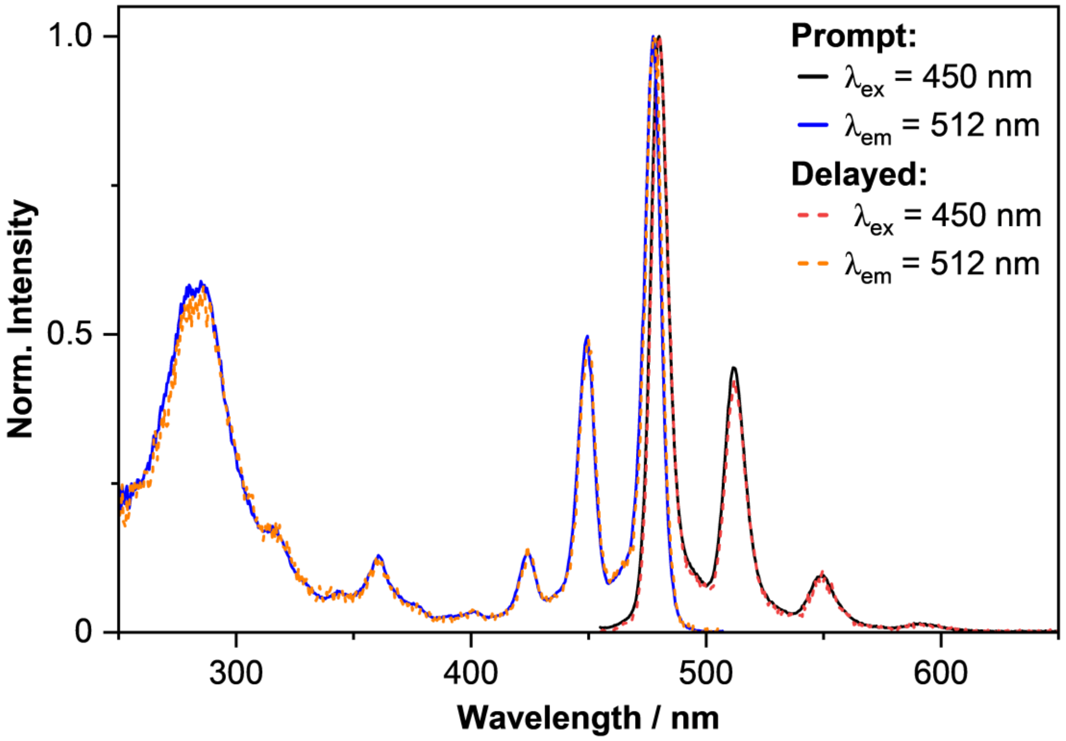

Figure S189. Comparison of the steady-state emission (black) and excitation (orange) spectra of **3** (7.6×10^{-7} M) at 293.15 K in degassed 2-MeTHF, with the gated (200 µs) delayed fluorescence emission (red, dashed) and excitation (blue, dashed).

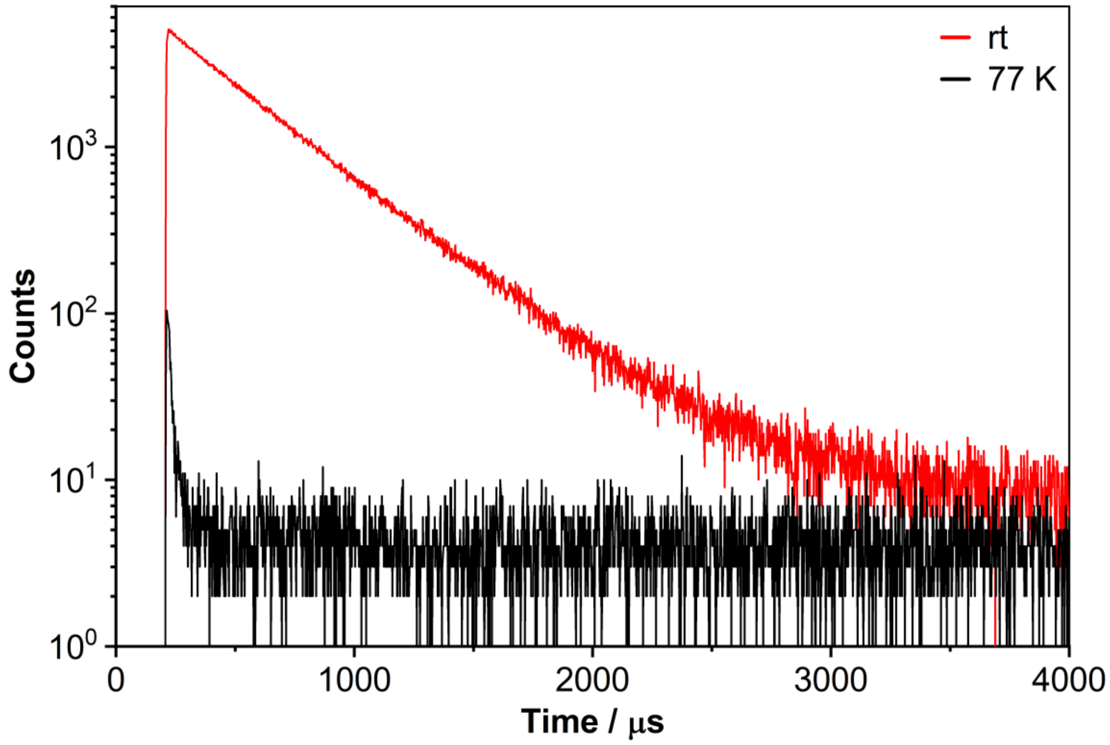

Figure S190. Time-resolved emission decay (λ_{ex} = 450 nm, λ_{em} = 480 nm, gate delay: 200 µs) of **3** (7.6×10^{-7} M) at 293.15 K (red) and at 77 K (black) in degassed 2-MeTHF.

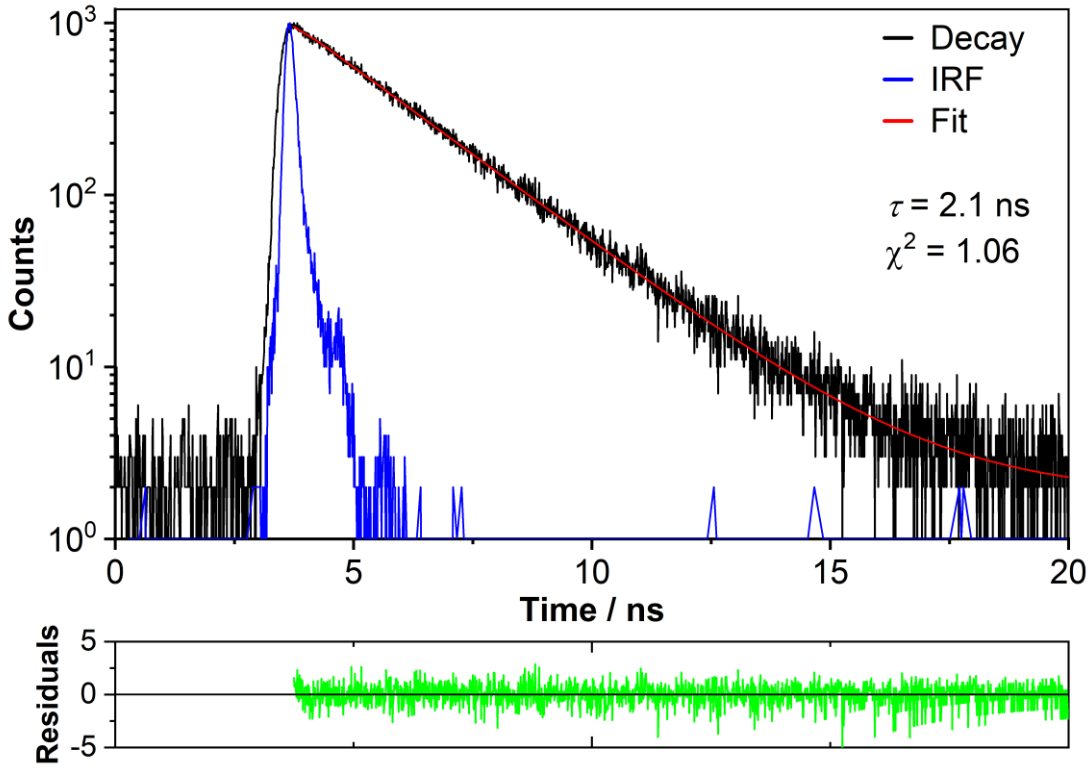

Figure S191. Time-resolved emission decay (λ_{ex} = 405.6 nm, λ_{em} = 480 nm) of **3** (7.6×10^{-7} M) at 293.15 K in degassed 2-MeTHF.

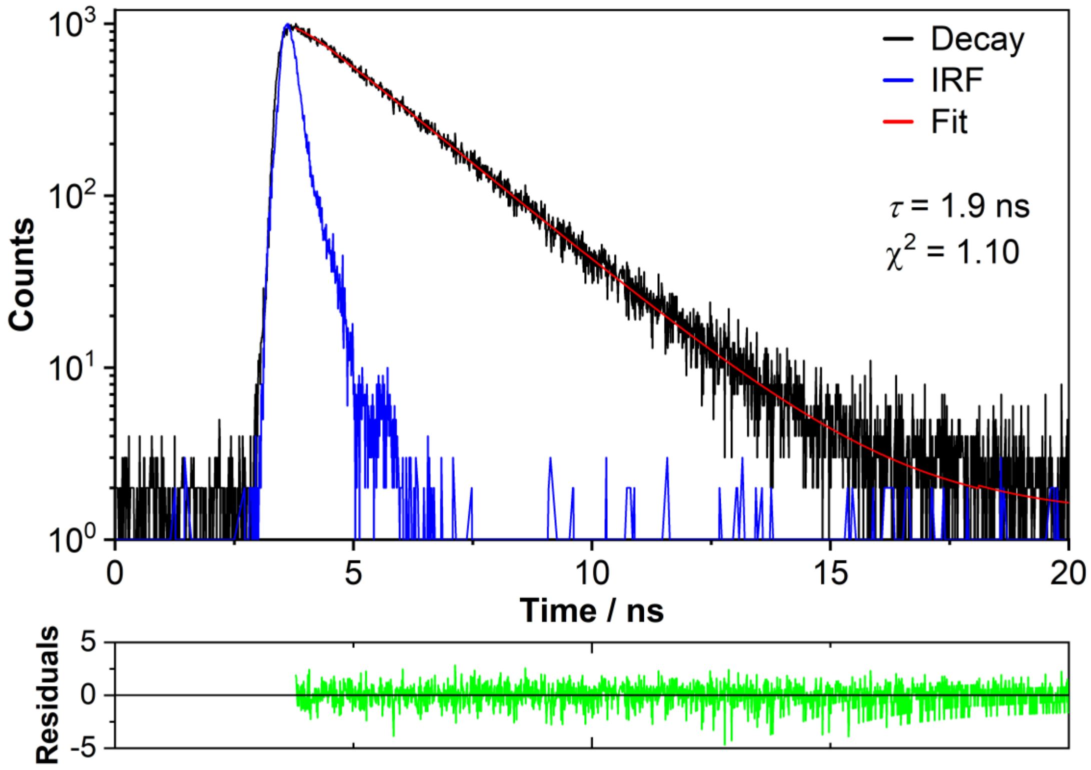

Figure S192. Time-resolved emission decay (λ_{ex} = 405.6 nm, λ_{em} = 480 nm) of **3** (7.6×10^{-7} M) at 77 K in degassed 2-MeTHF.

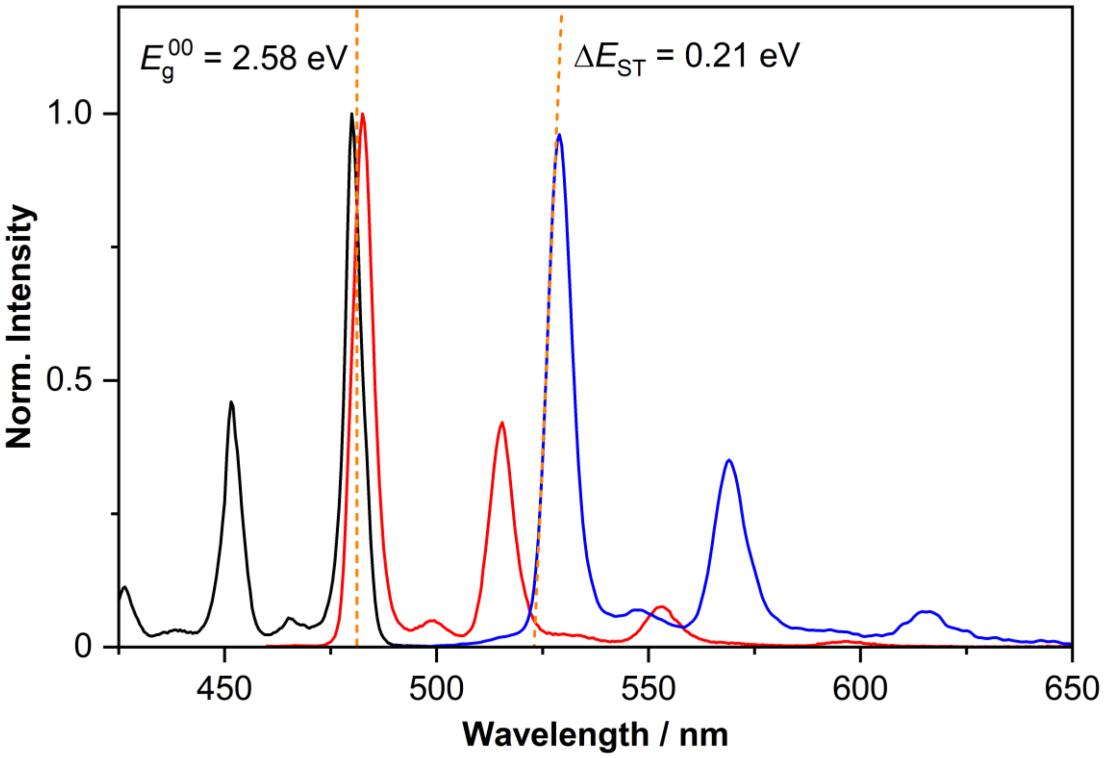

Figure S193. Determination of the optical bandgap (E_g^{00}) and the singlet-triplet gap (ΔE_{ST}) of **3** (7.3×10^{-7} M) at 77 K in 2-MeTHF, using the intercept of the normalized steady-state excitation (black, λ_{em} = 512 nm) and emission (red, λ_{ex} = 450 nm) spectra, and the time-resolved emission spectra (blue, λ_{ex} = 450 nm, 200 µs gate delay).

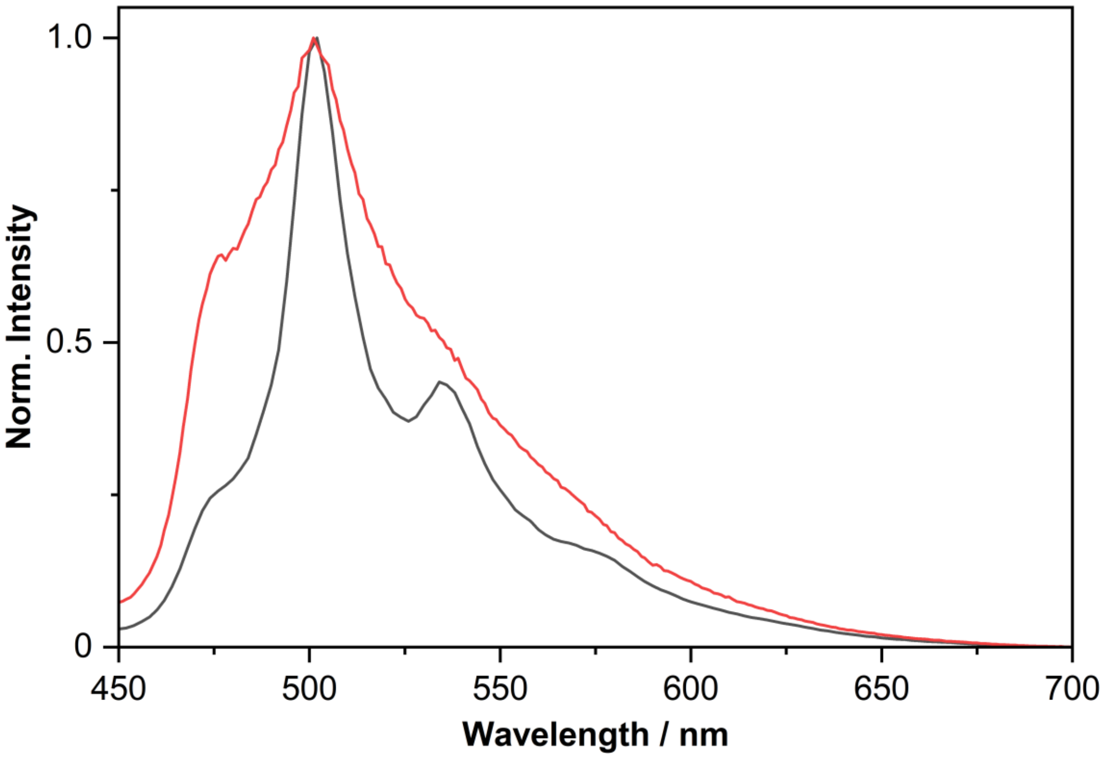

Figure S194. Normalized steady-state emission spectra of **4** after purification by column chromatography (black) and further purification by trituration with CH_3CN (red) at 293.15 K in CH_2Cl_2.

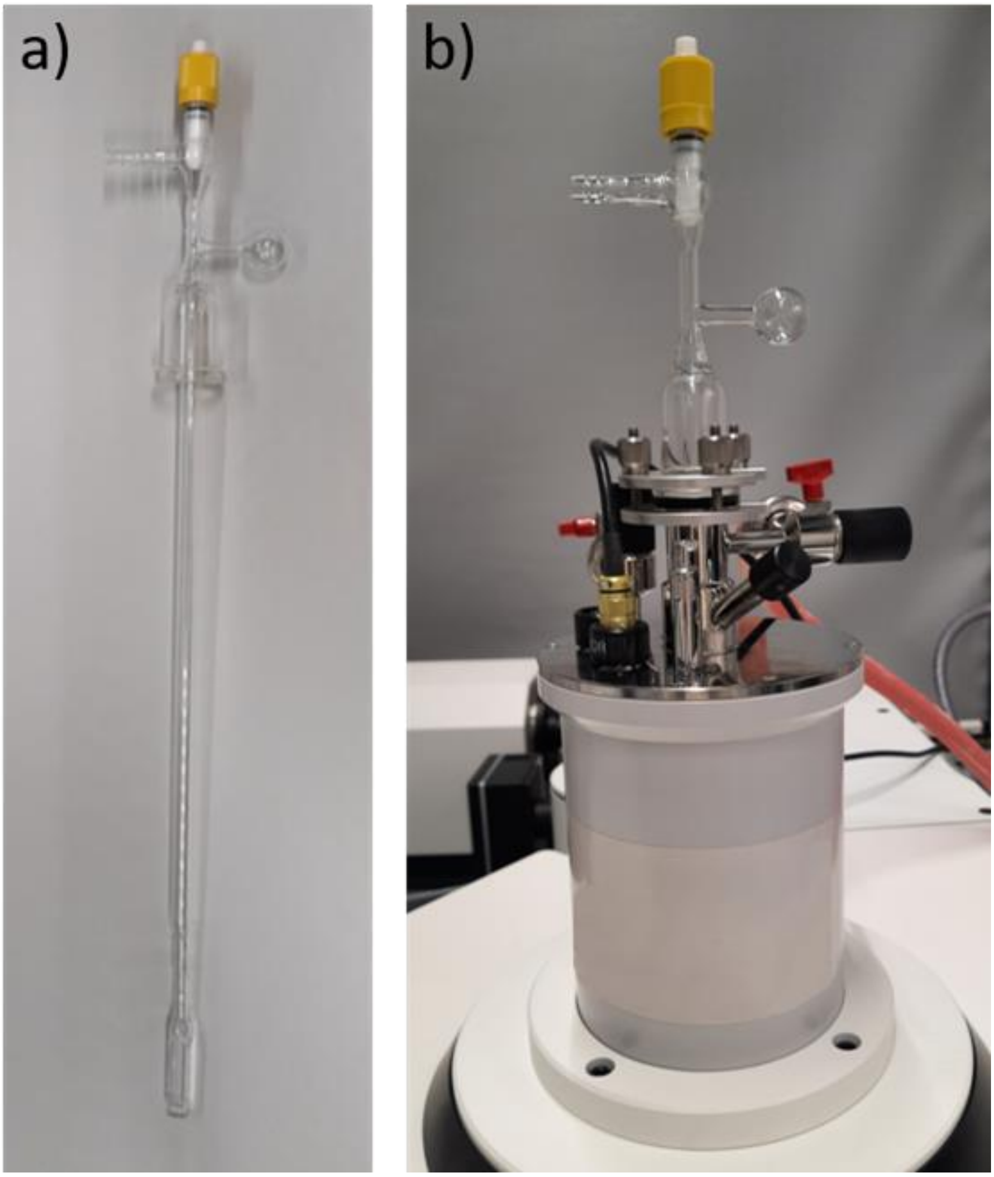

Figure S195. Custom-built cryogenic quartz cuvette (a) used for low-temperature measurements and (b) experimental setup.

While repeating the measurements with these compounds, we noticed that solutions prepared with solids of compounds $\mathbf{1^{Ph}}$, **2**, and **3** (kept in an air-tight vial at 5 °C) aged for 3 to 4 months lacked the delayed emission, whereas UV-Vis absorbance, steady-state emission, and excitation remained unaltered. Changing the solvent from 2-MeTHF to THF (degassed) allowed us to observe the delayed emission again, although with a significantly decreased fluorescence lifetime (*e.g.*, 60 µs vs 138 µs, for $\mathbf{1^{Ph}}$**,** Figure S196). This observation led us to hypothesize that some form of aggregation occurs in the solid state and remains in solution, quenching the delayed emission. To test this hypothesis, we performed temperature-dependent time-resolved fluorescence emission measurements of $\mathbf{1^{Ph}}$ in degassed THF, and measured the delayed emission lifetime as a function of temperature (Figure S196). As hypothesized, increasing the temperature from 25 °C to 60 °C, the delayed emission lifetime also increased from 60 µs to 231 µs, with an additional increase to 378 µs as the temperature

was lowered back to 25 °C, most likely due to reduced thermal deactivation of the triplet state. Going back to 2-MeTHF, and applying the same thermal annealing procedure, allowed us to recover the delayed emission component for all compounds. Noticeably, for nanoribbon **1^Ph^**, after thermal annealing to 80 °C, we observed a delayed fluorescence lifetime longer (204 µs) than previously measured (138 µs) in 2-MeTHF, suggesting that a partial aggregation and consequent quenching of delayed emission could be observed (Figure S197). For nanoribbon **2**, the most insoluble derivative of the series, the annealing process increased the delayed fluorescence lifetime from 22 µs to 204 µs, although the value of 640 µs previously measured could not be achieved in our experimental conditions (Figure S198). Unexpectedly, the thermal annealing of molecule **3** showed a decrease in the delayed emission lifetime during the heating ramp. Nevertheless, once cooled back to room temperature, a lifetime of 333 µs was measured, comparable to the 387 µs previously measured (Figure S199). Steady-state measurements of this sample revealed a new set of peaks with a hypsochromic shift of 0.06 eV (11–16 nm) compared to pristine nanoribbon **3** and an isosbestic point at 444 nm (Figure S200). This observation suggests a transformation of the molecule, likely due to hydrolysis of the terminal phenyl groups attached to boron. Heating the sample further to 80 °C until complete conversion to the hydrolyzed product, followed by mass analysis, revealed a main peak with a m/z of 1522.8376, consistent with the borinic acid-type derivative (Figure S201). The hydrolytic cleavage of B-Ph groups, in non-dry solvents, has already been reported.[29]

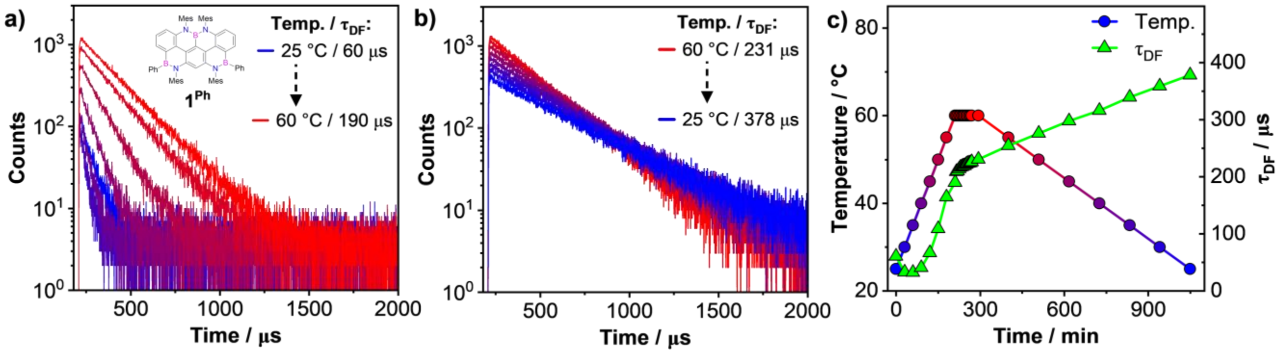

Figure S196. Gated (200 µs) delayed fluorescence decay during heating (a) and cooling (b) of **1^Ph^** in THF. c) delayed fluorescence lifetime as a function of temperature and time.

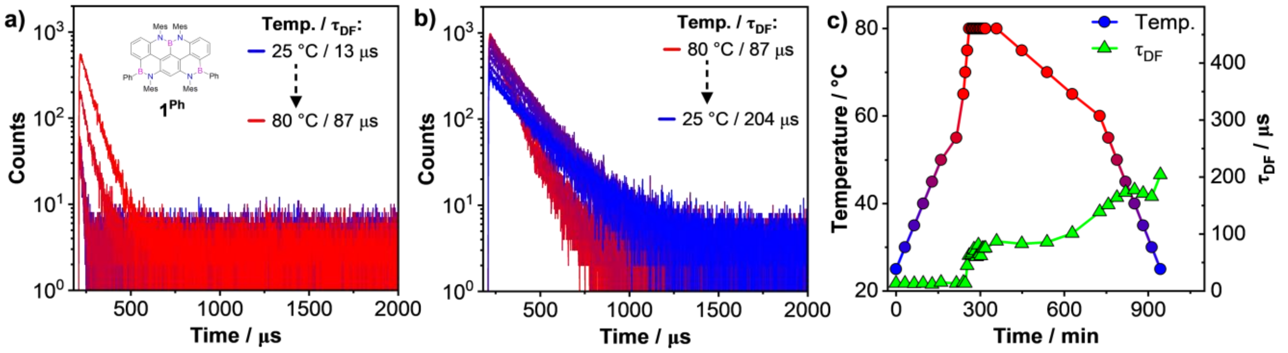

Figure S197. Gated (200 µs) delayed fluorescence decay during heating (a) and cooling (b) of **1^Ph^** in 2-MeTHF. c) delayed fluorescence lifetime as a function of temperature and time.

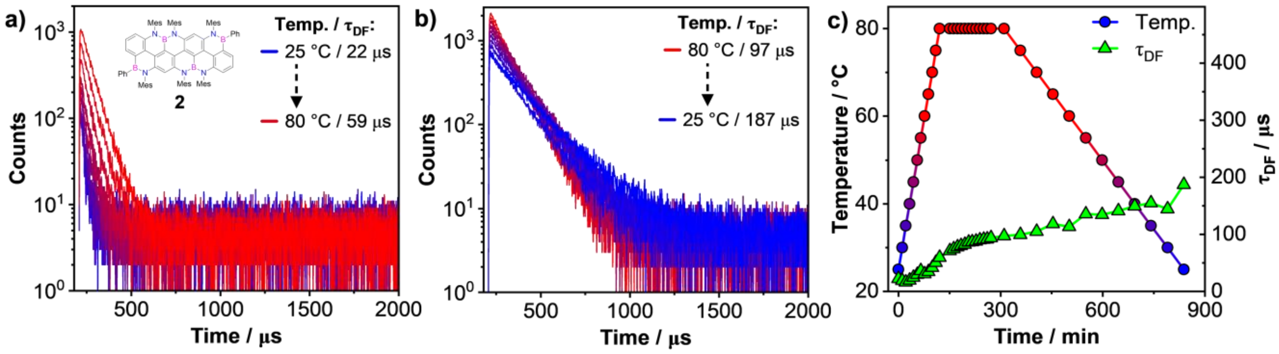

Figure S198. Gated (200 µs) delayed fluorescence decay during heating (a) and cooling (b) of **2** in 2-MeTHF. c) delayed fluorescence lifetime as a function of temperature and time.

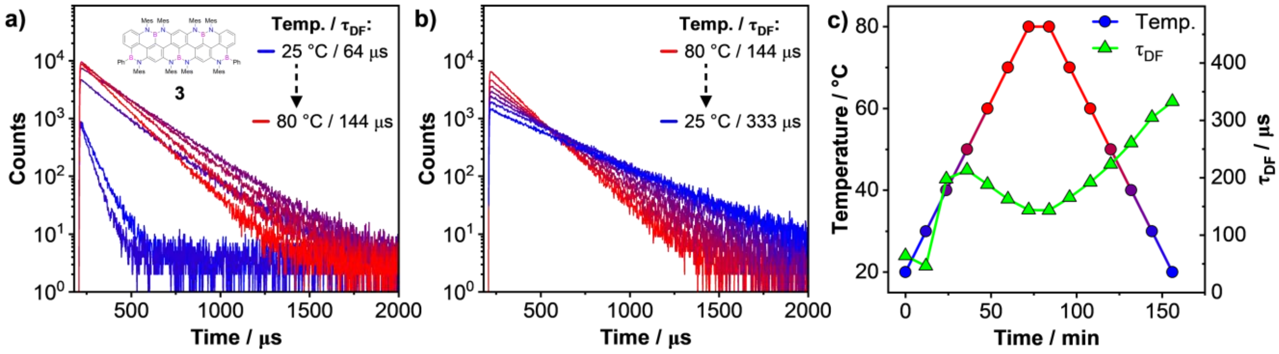

Figure S199. Gated (200 µs) delayed fluorescence decay during heating (a) and cooling (b) of **3** in 2-MeTHF. c) delayed fluorescence lifetime as a function of temperature and time.

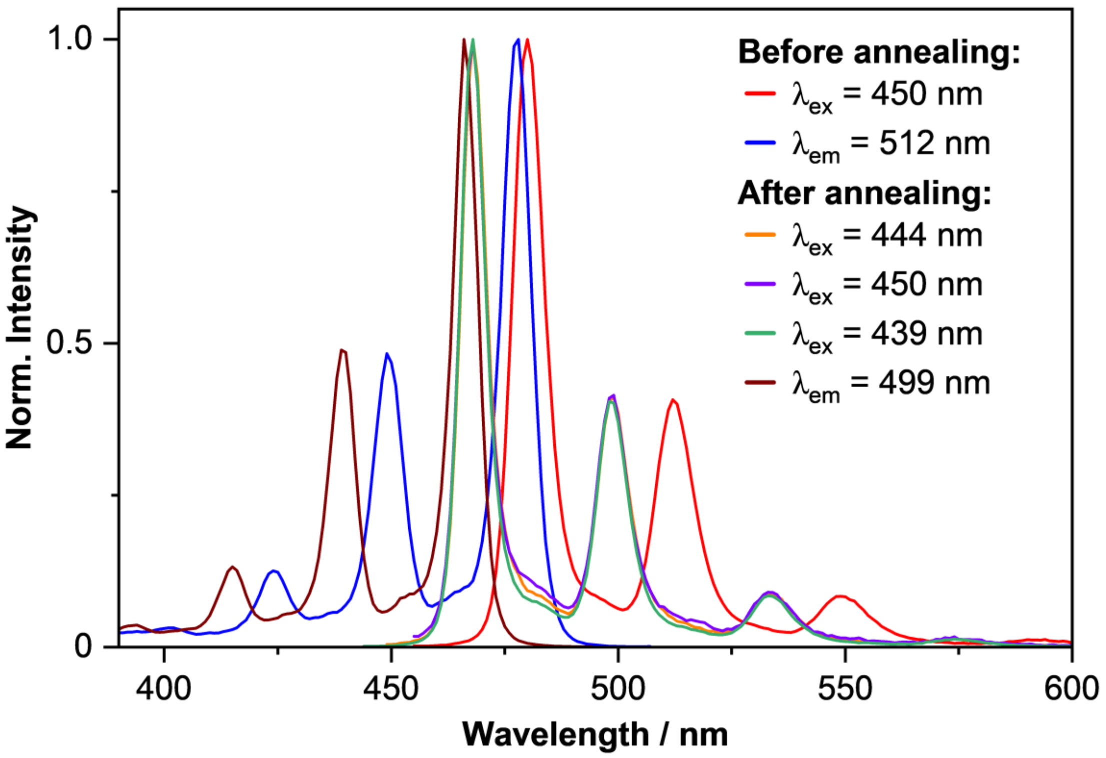

Figure S200. Steady-state excitation and emission of **3** before (red, blue) and after thermal annealing (orange, purple, green, brown).

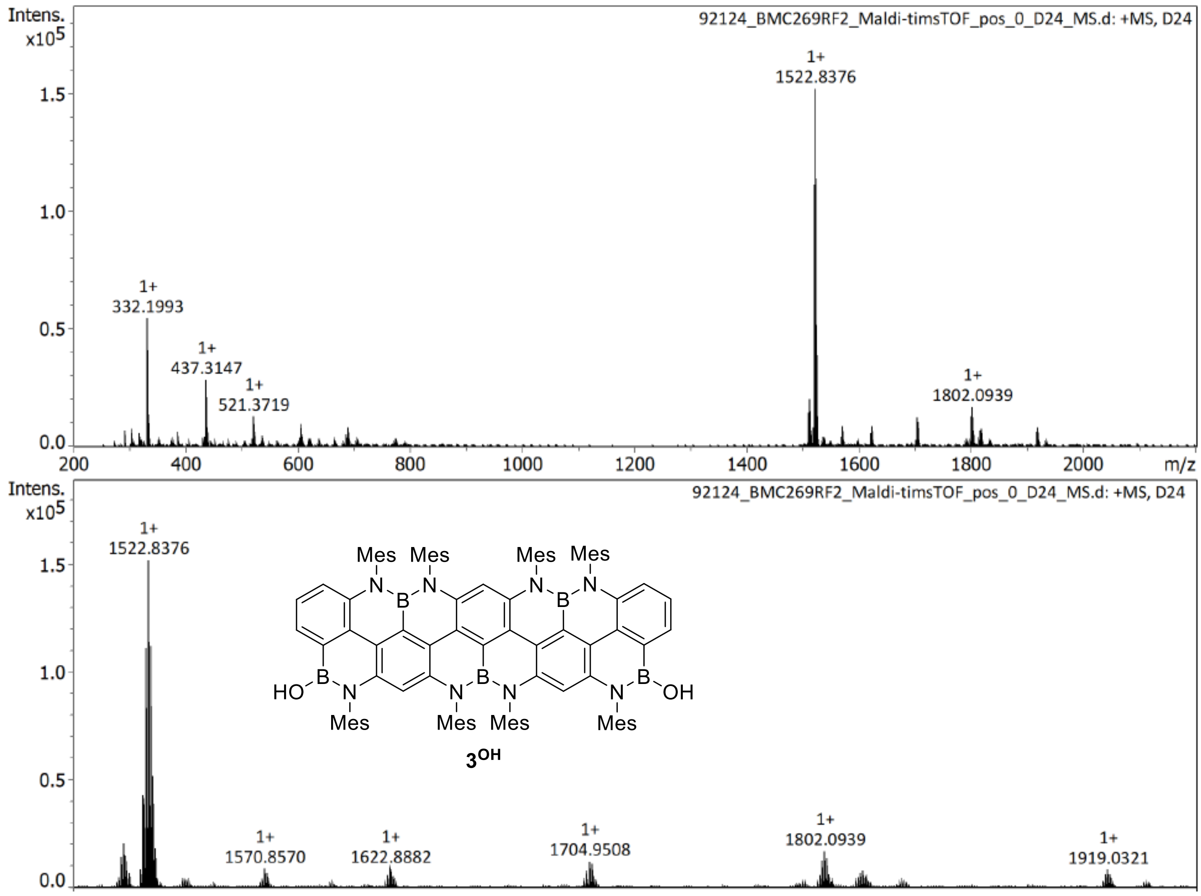

Figure S201. HRMS (MALDI-timsTOF, matrix: DCTB) spectrum of decomposed nanoribbon **3**.

3.2. Electrochemical properties

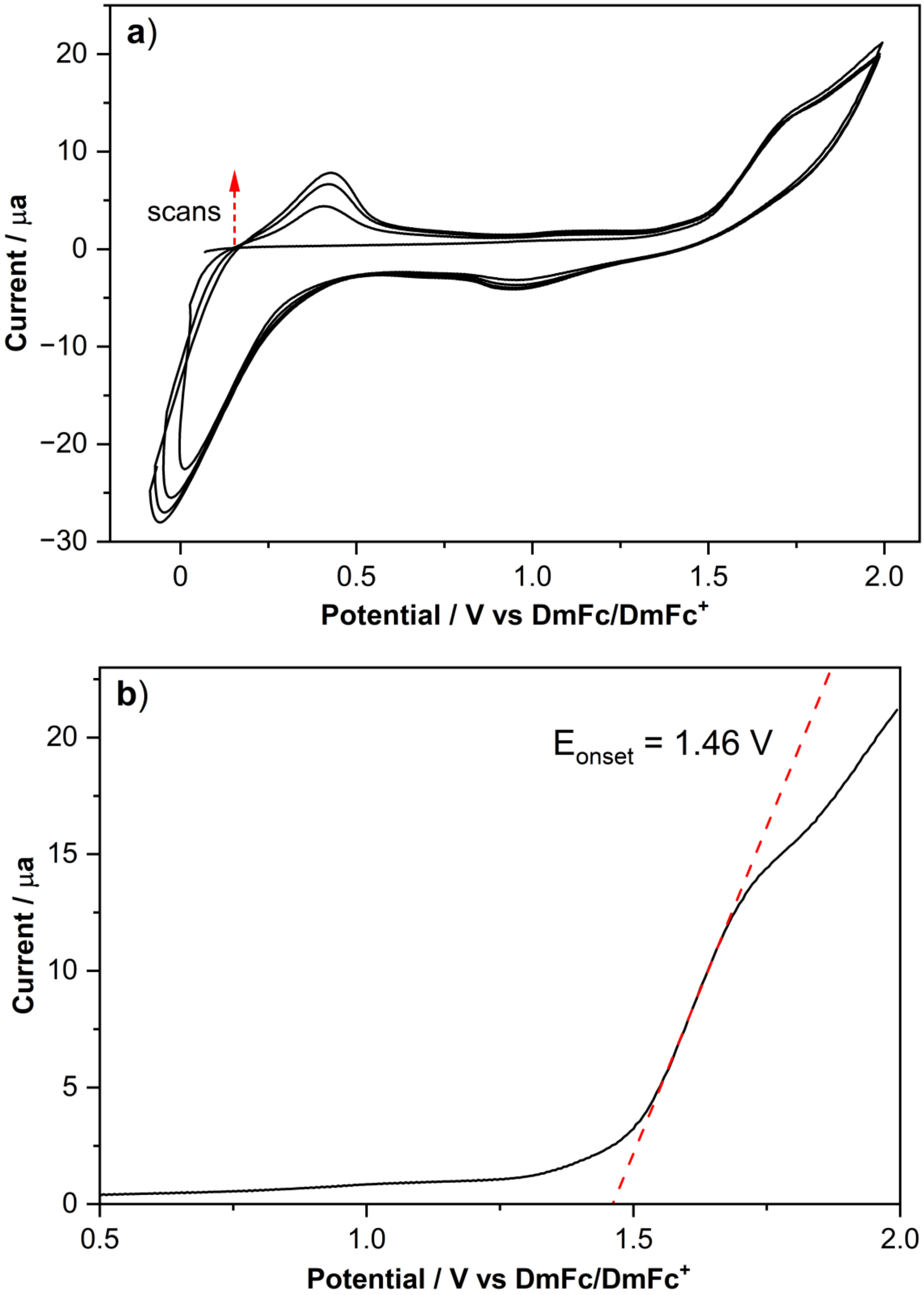

Figure S202. Photophysical analysis of compound $\mathbf{5^{Ph}}$ (0.2 mM) in TCE: a) Cyclic voltammogram at different scan rates (10 - 60 mV/s); b) Linear dependence between peak current and scan rate$^{1/2}$; c) CV of **3** mixed with Decamethylferrocene as an internal reference, at a scan rate of 60 mV/s.

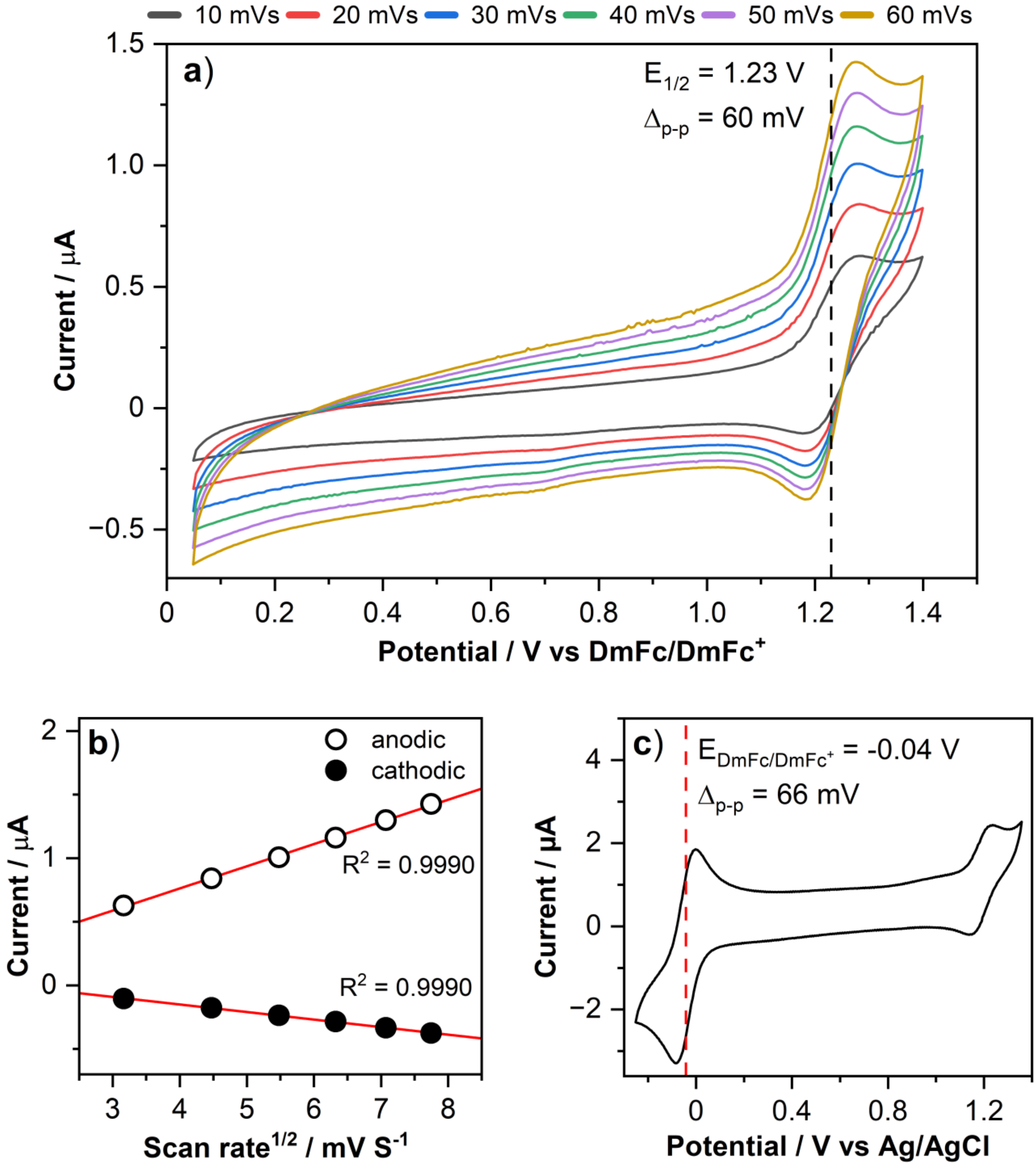

Figure S203. Photophysical analysis of compound **6** (0.2 mM) in *o*-DCB/CH_3CN (4:1): a) Cyclic voltammogram at different scan rates (10 - 60 mV/s); b) Linear dependence between peak current and scan rate$^{1/2}$; c) CV of **6** mixed with Decamethylferrocene as an internal reference, at a scan rate of 100 mV/s.

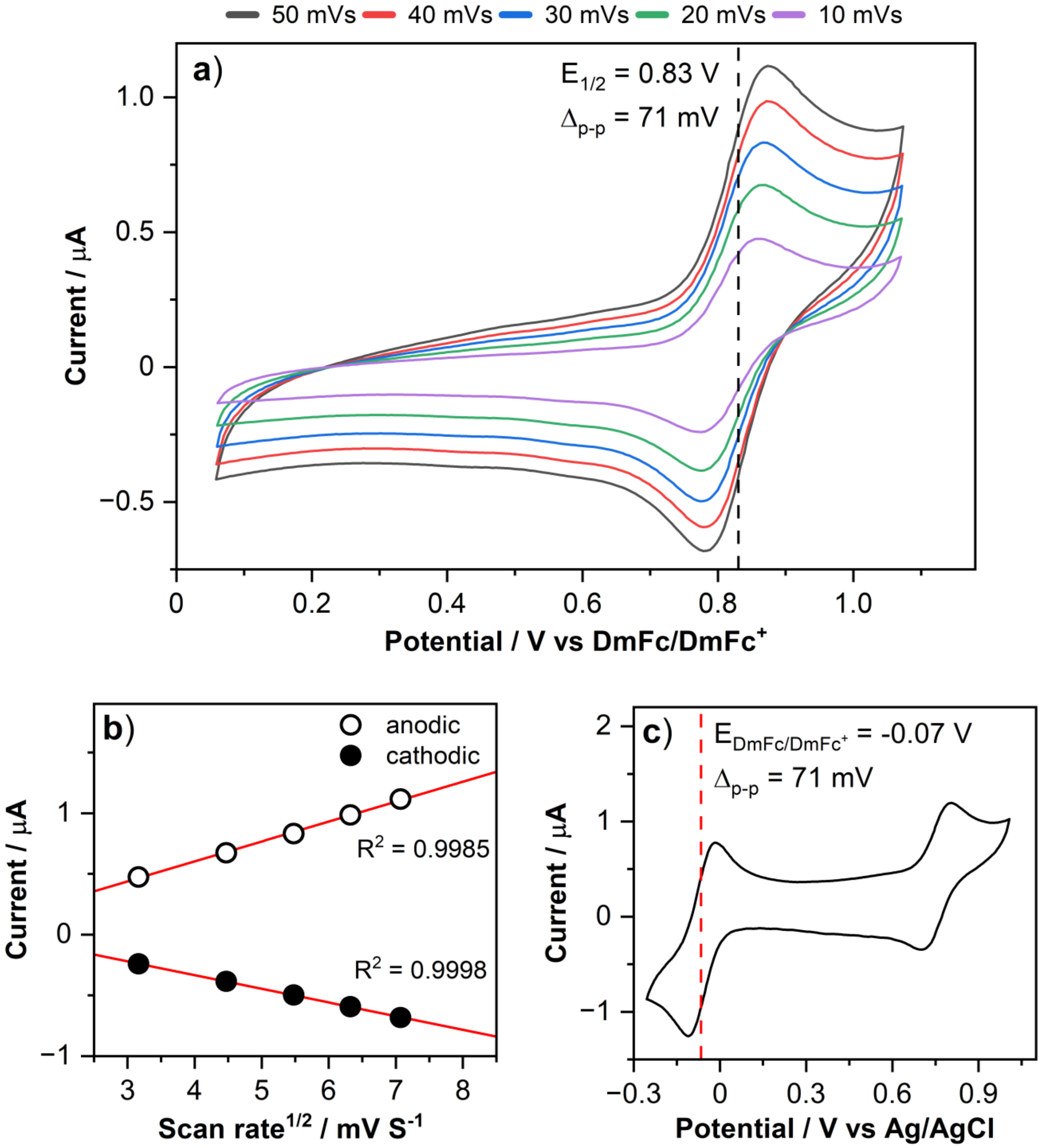

Figure S204. Photophysical analysis of compound **1^{Ph}** (0.2 mM) in TCE: a) Cyclic voltammogram at different scan rates (10 - 60 mV/s); b) Linear dependence between peak current and scan rate$^{1/2}$; c) CV of **1^{Ph}** mixed with Decamethylferrocene as an internal reference, at a scan rate of 60 mV/s.

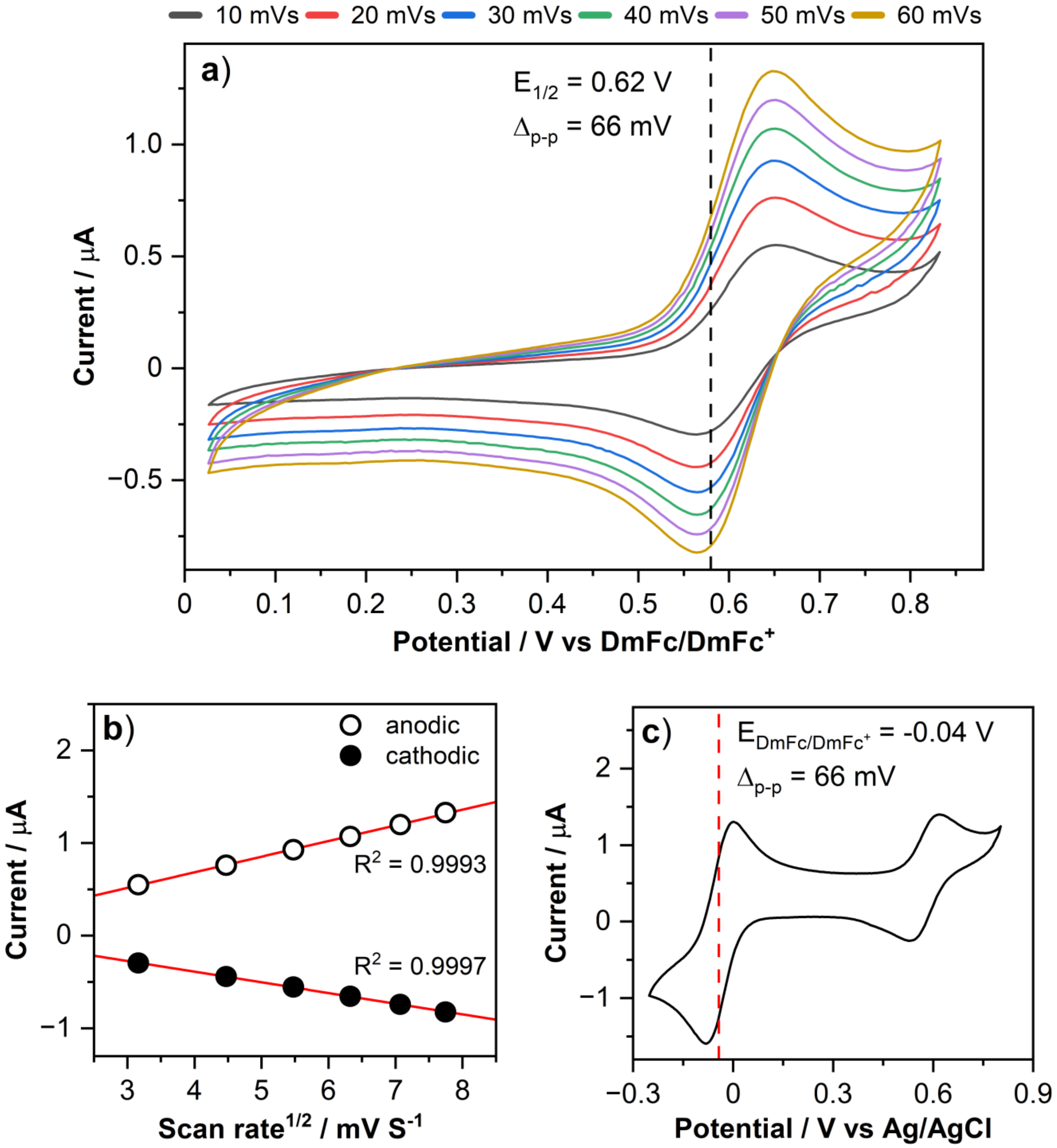

Figure S205. Photophysical analysis of compound **2** (0.2 mM) in *o*-DCB/CH_3CN (4:1): a) Cyclic voltammogram at different scan rates (10 - 60 mV/s); b) Linear dependence between peak current and scan rate$^{1/2}$; c) CV of **2** mixed with Decamethylferrocene as an internal reference, at a scan rate of 60 mV/s.

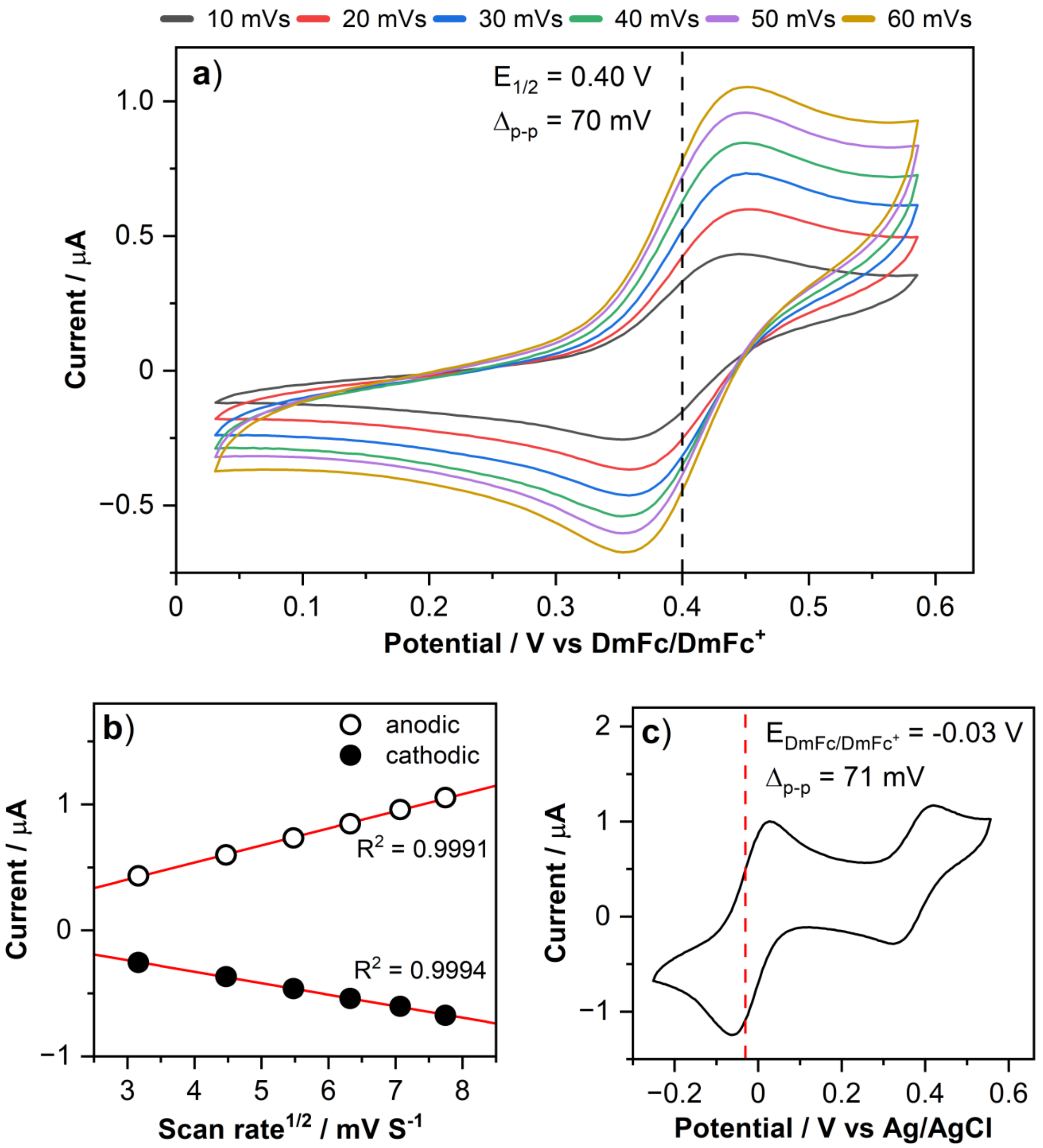

Figure S206. Photophysical analysis of compound **3** (0.2 mM) in *o*-DCB/CH_3CN (4:1): a) Cyclic voltammogram at different scan rates (10 - 60 mV/s); b) Linear dependence between peak current and scan rate$^{1/2}$; c) CV of **3** mixed with Decamethylferrocene as an internal reference, at a scan rate of 60 mV/s.

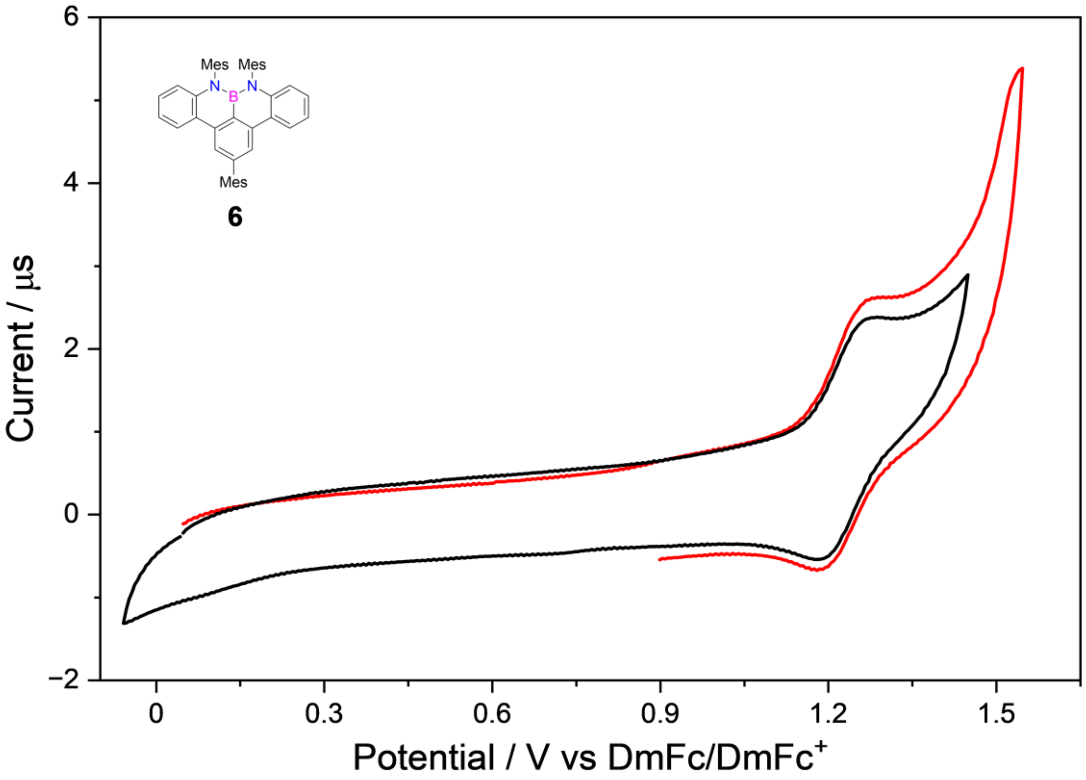

Figure S207. Cyclic voltammogram of compound **6** (0.2 mM) in *o*-DCB/CH_3CN (4:1), scanning only the first oxidation event (0 → 1.4 V, black) and both first and second oxidation (0 → 1.5 V, red). Scan rate: 100 mV/s.

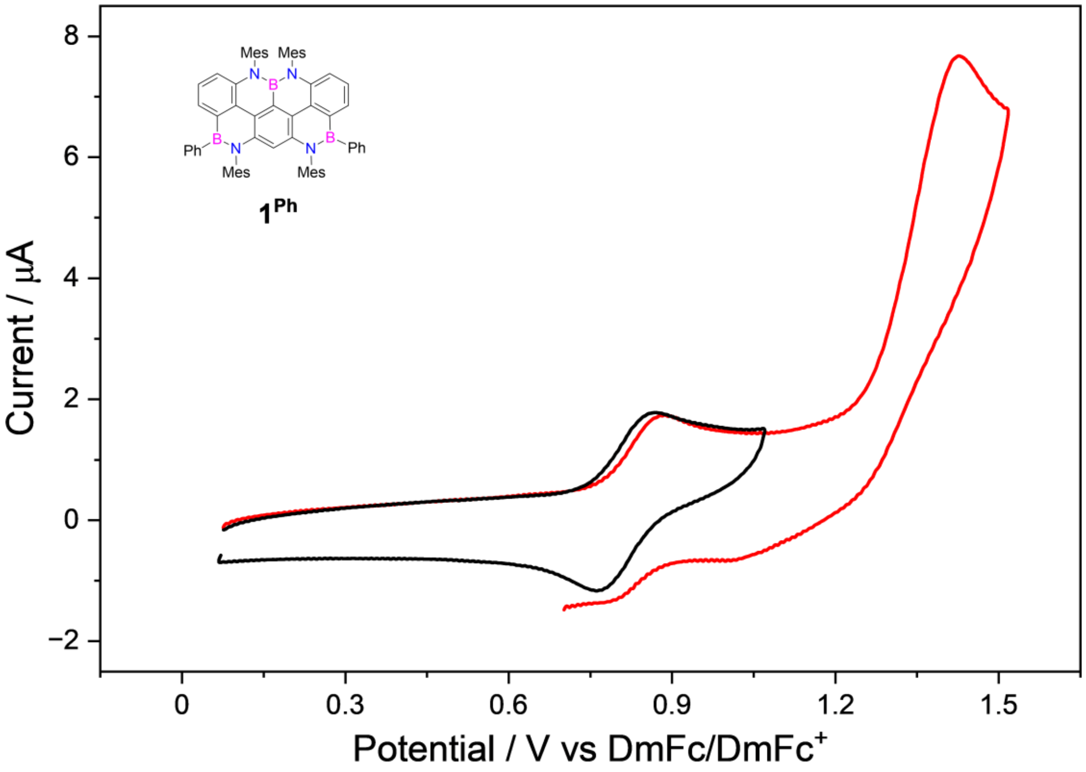

Figure S208. Cyclic voltammogram of compound **2** (0.2 mM) in TEC, scanning only the first oxidation event (0 → 1.0 V, black) and both first and second oxidation (0 → 1.5 V, red). Scan rate: 100 mV/s.

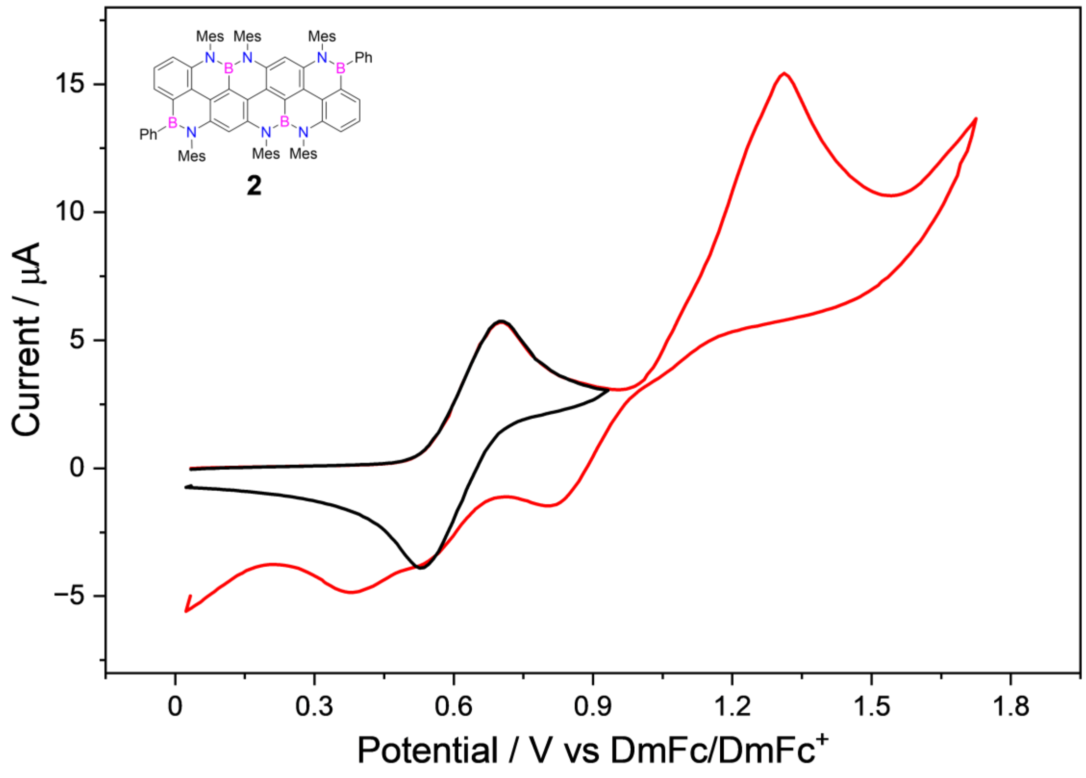

Figure S209. Cyclic voltammogram of compound **2** (0.2 mM) in *o*-DCB/CH_3CN (4:1), scanning only the first oxidation event (0 → 0.9 V, black) and both first and second oxidation (0 → 1.7 V, red). Scan rate: 100 mV/s.

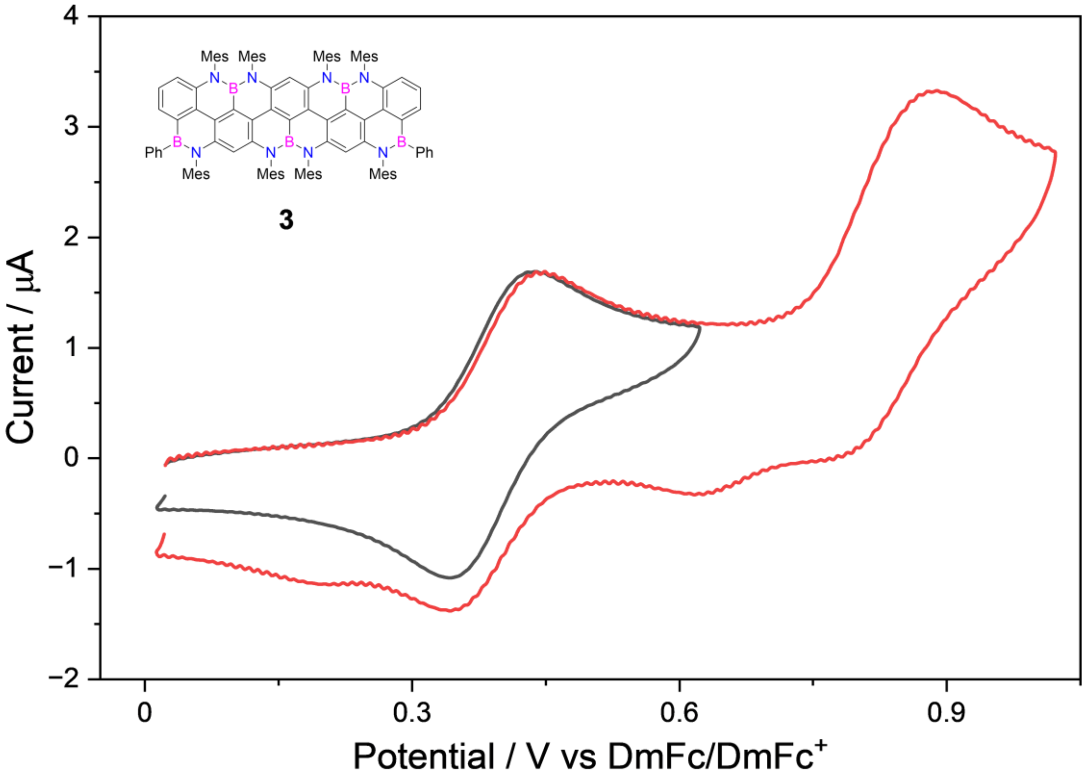

Figure S210. Cyclic voltammogram of compound **3** (0.2 mM) in *o*-DCB/CH_3CN (4:1), scanning only the first oxidation event (0 → 0.6 V, black) and both first and second oxidation (0 → 1 V, red). Scan rate: 100 mV/s.

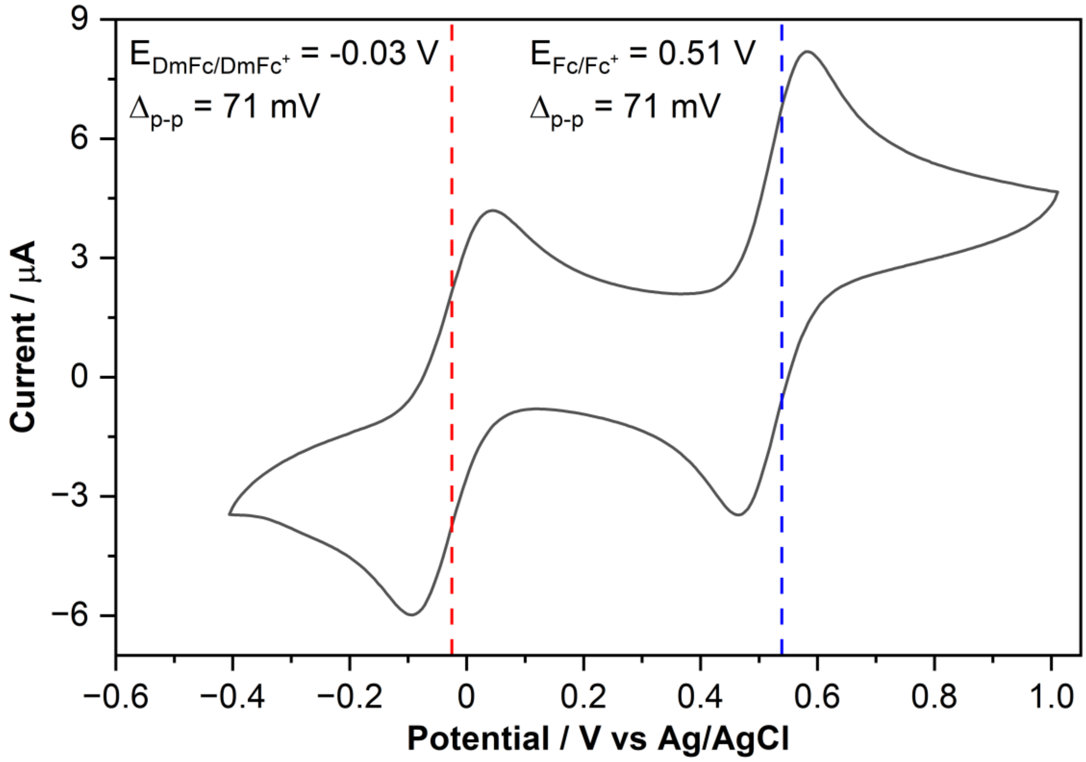

Figure S211. Cyclic voltammogram of Ferrocene and Decamethylferrocene in *o*-DCB/CH_3CN (4:1), at a scan rate of 60 mV/s.

4. Crystallographic data

Table S1. Crystal data and structure refinement for **5^{Me}** (2165184).

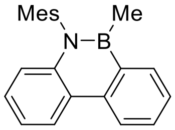

Empirical formula	$C_{22}H_{22}BN$	
Formula weight	311.21	
Crystal system	Orthorhombic	
Space group	*Pna*2_1	
Unit cell dimensions	a = 9.2494(6) Å	α = 90°
	b = 19.0716(8) Å	β = 90°
	c = 9.8649(12) Å	γ = 90°
Volume	1740.2(3) Å^3	
Z	4	
Density (calculated)	1.188 mg/m^3	
Absorption coefficient	0.507 mm^{-1}	
F(000)	664	
Crystal size	0.160 × 0.090 × 0.050 mm^3	
	**Data collection**	
Temperature	100(2) K	
Wavelength	1.54186 Å	
Theta range for data collection	4.637 to 70.736°	
Index ranges	-4 ≤ h ≤ 11, -23 ≤ k ≤ 21, -8 ≤ l ≤ 11	
Reflections collected	9727	
Independent reflections	2727 [R(int) = 0.0223]	
Completeness to theta = 67.679°	99.5%	
	**Refinement**	
Absorption correction	multi-scan	
Refinement method	Full-matrix least-squares on F^2	
Data / restraints / parameters	2727 / 1 / 221	
Goodness-of-fit on F^2	1.040	
Final R indices [I>2sigma(I)]	R1 = 0.0330, wR2 = 0.0788	
R indices (all data)	R1 = 0.0400, wR2 = 0.0818	
Extinction coefficient	n/a	
Largest diff. peak and hole	0.174 and -0.209 e·Å^{-3}	

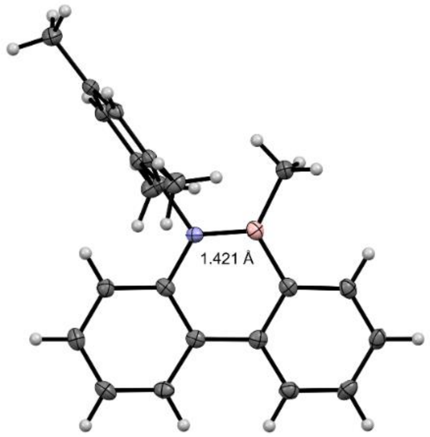

Figure S212. ORTEP representation (50% probability ellipsoids) of the X-ray crystal structure for **5^{Me}**. B-N bond length reported in Å. Atom colors: gray C, pink B, blue N.

Table S2. Crystal data and structure refinement for **5^Ph** (2181763).

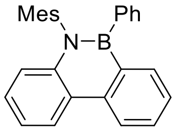

Empirical formula	$C_{27}H_{24}BN$	
Formula weight	373.30	
Crystal system	Monoclinic	
Space group	$P2_1$	
Unit cell dimensions	a = 16.6341(9) Å	α = 90°
	b = 7.3167(3) Å	β = 94.858(4)°
	c = 33.8064(18) Å	γ = 90°
Volume	4099.7(4) $Å^3$	
Z	8	
Density (calculated)	1.210 mg/m^3	
Absorption coefficient	0.069 mm^{-1}	
F(000)	1584	
Crystal size	0.320 × 0.140 × 0.040 mm^3	
	**Data collection**	
Temperature	100(2) K	
Wavelength	0.71073 Å	
Theta range for data collection	1.65 to 28.91°	
Index ranges	-22 ≤ h ≤ 22, -9 ≤ k ≤ 9, -45 ≤ l ≤ 31	
Reflections collected	87622	
Independent reflections	17794 [R(int) = 0.0751]	
Completeness to theta = 25.242°	99.8%	
	**Refinement**	
Absorption correction	multi-scan	
Refinement method	Full-matrix least-squares on F^2	
Data / restraints / parameters	17794 / 1 / 1057	
Goodness-of-fit on F^2	0.838	
Final R indices [I>2sigma(I)]	R1 = 0.0491, wR2 = 0.0900	
R indices (all data)	R1 = 0.1222, wR2 = 0.0963	
Extinction coefficient	n/a	
Largest diff. peak and hole	0.228 and -0.291 $e^{\cdot}Å^{-3}$	

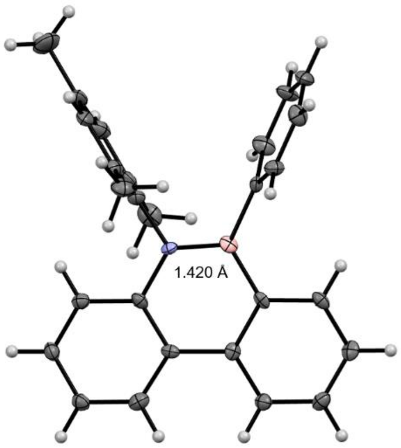

Figure S213. ORTEP representation (50% probability ellipsoids) of the X-ray crystal structure for **5^Ph**. A single, crystallographically independent, molecule representative of the crystal is shown. B-N bond length reported in Å. Atom colors: gray C, pink B, blue N.

Table S3. Crystal data and structure refinement for **5^{Mes}** (2165204).

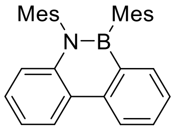

Empirical formula	$C_{30}H_{30}BN$	
Formula weight	415.36	
Crystal system	Monoclinic	
Space group	$P2_1/c$	
Unit cell dimensions	a = 33.993(3) Å	α = 90°
	b = 7.7634(6) Å	β = 99.253(7)°
	c = 17.9014(15) Å	γ = 90°
Volume	4662.8(6) $Å^3$	
Z	8	
Density (calculated)	1.183 mg/m^3	
Absorption coefficient	0.067 mm^{-1}	
F(000)	1776	
Crystal size	0.090 × 0.067 × 0.030 mm^3	
	**Data collection**	
Temperature	100(2) K	
Wavelength	0.71073 Å	
Theta range for data collection	2.288 to 26.371°	
Index ranges	-42 ≤ h ≤ 18, -9 ≤ k ≤ 9, -18 ≤ l ≤ 22	
Reflections collected	94149	
Independent reflections	9491 [R(int) = 0.0907]	
Completeness to theta = 25.242°	99.5%	
	**Refinement**	
Absorption correction	multi-scan	
Refinement method	Full-matrix least-squares on F^2	
Data / restraints / parameters	9491 / 872 / 515	
Goodness-of-fit on F^2	0.750	
Final R indices [I>2sigma(I)]	R1 = 0.0443, wR2 = 0.0874	
R indices (all data)	R1 = 0.1317, wR2 = 0.0953	
Extinction coefficient	n/a	
Largest diff. peak and hole	0.231 and -0.231 $e^{\cdot}Å^{-3}$	

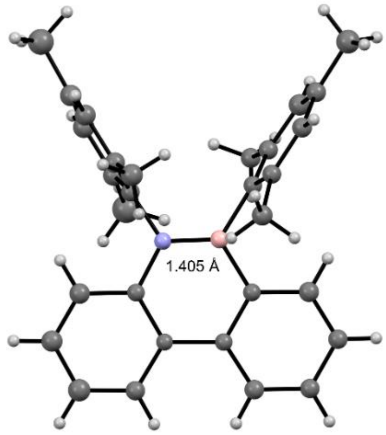

Figure S214. ORTEP representation (50% probability ellipsoids) of the X-ray crystal structure for **5^{Mes}**. A single, crystallographically independent, molecule representative of the crystal is shown. B-N bond length reported in Å. Atom colors: gray C, pink B, blue N.

Table S4. Crystal data and structure refinement for **6** (2165185).

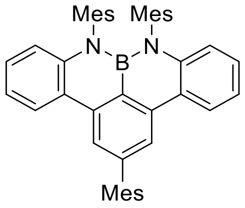

Empirical formula	$C_{45}H_{43}BN_2 \cdot CHCl_3$	
Formula weight	741.99	
Crystal system	Monoclinic	
Space group	$P2_1/n$	
Unit cell dimensions	a = 15.6666(8) Å	α = 90°
	b = 16.4576(8) Å	β = 111.356(3)°
	c = 15.9235(7) Å	γ = 90°
Volume	3823.7(3) Å^3	
Z	4	
Density (calculated)	1.289 mg/m^3	
Absorption coefficient	2.434 mm^{-1}	
F(000)	1560	
Crystal size	0.300 × 0.220 × 0.060 mm^3	
	**Data collection**	
Temperature	100(2) K	
Wavelength	1.54186 Å	
Theta range for data collection	3.388 to 72.170°	
Index ranges	-13 ≤ h ≤ 19, -20 ≤ k ≤ 15, -17 ≤ l ≤ 19	
Reflections collected	25980	
Independent reflections	7223 [R(int) = 0.0275]	
Completeness to theta = 67.679°	98.0%	
	**Refinement**	
Absorption correction	multi-scan	
Refinement method	Full-matrix least-squares on F^2	
Data / restraints / parameters	7223 / 0 / 478	
Goodness-of-fit on F^2	1.061	
Final R indices [I>2sigma(I)]	R1 = 0.0437, wR2 = 0.1212	
R indices (all data)	R1 = 0.0584, wR2 = 0.1295	
Extinction coefficient	n/a	
Largest diff. peak and hole	0.685 and -0.474 e·Å^{-3}	

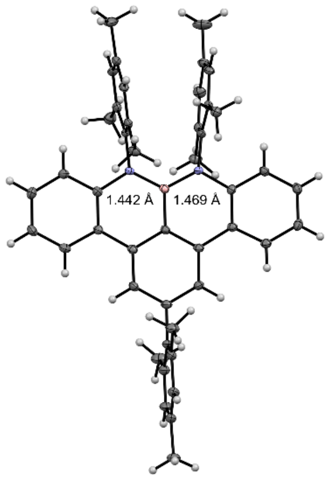

Figure S215. ORTEP representation (50% probability ellipsoids) of the X-ray crystal structure for **6**. B-N bond lengths reported in Å. Solvent molecules omitted for clarity. Atom colors: gray C, pink B, blue N.

Table S5. Crystal data and structure refinement for $\mathbf{1^{Me}}$ (2165187).

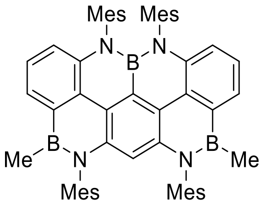

Empirical formula	$C_{56}H_{57}B_3N_4$	
Formula weight	818.51	
Crystal system	Monoclinic	
Space group	*Pc*	
Unit cell dimensions	a = 13.4738(10) Å	α = 90°
	b = 8.0120(5) Å	β = 102.646(6)°
	c = 21.5409(14) Å	γ = 90°
Volume	2269.0(3) $Å^3$	
Z	2	
Density (calculated)	1.198 mg/m^3	
Absorption coefficient	0.519 mm^{-1}	
F(000)	872	
Crystal size	0.350 × 0.090 × 0.050 mm^3	
	**Data collection**	
Temperature	100(2) K	
Wavelength	1.54186 Å	
Theta range for data collection	3.362 to 71.456°	
Index ranges	-16 ≤ h ≤ 13, -5 ≤ k ≤ 9, -26 ≤ l ≤ 24	
Reflections collected	46233	
Independent reflections	6951 [R(int) = 0.0380]	
Completeness to theta = 67.679°	99.5%	
	**Refinement**	
Absorption correction	multi-scan	
Refinement method	Full-matrix least-squares on F^2	
Data / restraints / parameters	6951 / 5624 / 1164	
Goodness-of-fit on F^2	1.030	
Final R indices [I>2sigma(I)]	R1 = 0.0402, wR2 = 0.1007	
R indices (all data)	R1 = 0.0563, wR2 = 0.1093	
Extinction coefficient	n/a	
Largest diff. peak and hole	0.195 and -0.154 $e^{\cdot}Å^{-3}$	

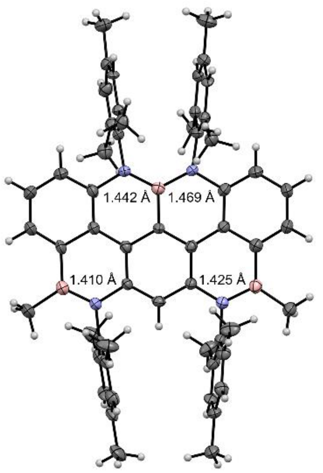

Figure S216. ORTEP representation (50% probability ellipsoids) of the X-ray crystal structure for $\mathbf{1^{Me}}$. A single, crystallographically independent, molecule representative of the crystal is shown. B-N bond lengths reported in Å. Atom colors: gray C, pink B, blue N.

Table S6. Crystal data and structure refinement for **1^Ph^** (2165201).

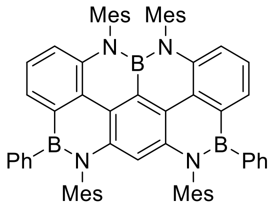

Empirical formula	$(C_{66}H_{61}B_3N_4)_2\cdot(C_7H_8)_3$	
Formula weight	2161.63	
Crystal system	Triclinic	
Space group	$P\bar{1}$	
Unit cell dimensions	a = 15.3759(10) Å	α = 86.551(5)°
	b = 15.6200(9) Å	β = 80.384(5)°
	c = 25.5897(16) Å	γ = 79.257(5)°
Volume	5950.7(7) Å^3	
Z	2	
Density (calculated)	1.206 mg/m^3	
Absorption coefficient	0.069 mm^{-1}	
F(000)	2300	
Crystal size	0.420 × 0.193 × 0.060 mm^3	
	**Data collection**	
Temperature	100(2) K	
Wavelength	0.71073 Å	
Theta range for data collection	1.725 to 26.373°	
Index ranges	-19 ≤ h ≤ 19, -19 ≤ k ≤ 19, -31 ≤ l ≤ 29	
Reflections collected	240755	
Independent reflections	24322 [R(int) = 0.0911]	
Completeness to theta = 25.242°	99.9%	
	**Refinement**	
Absorption correction	multi-scan	
Refinement method	Full-matrix least-squares on F^2	
Data / restraints / parameters	24322 / 0 / 1504	
Goodness-of-fit on F^2	0.910	
Final R indices [I>2sigma(I)]	R1 = 0.0604, wR2 = 0.1803	
R indices (all data)	R1 = 0.1384, wR2 = 0.1994	
Extinction coefficient	n/a	
Largest diff. peak and hole	0.473 and -0.480 e·Å^{-3}	

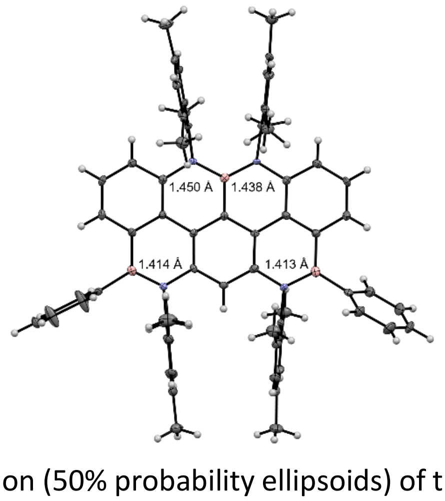

Figure S217. ORTEP representation (50% probability ellipsoids) of the X-ray crystal structure for **1^Ph^**. A single, crystallographically independent, molecule representative of the crystal is shown. B-N bond lengths reported in Å. Solvent molecules omitted for clarity. Atom colors: gray C, pink B, blue N.

Table S7. Crystal data and structure refinement for **2** (2165203).

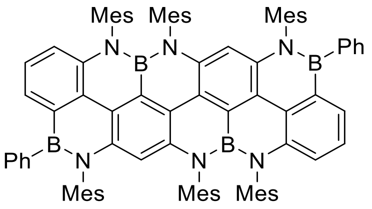

Empirical formula	$C_{90}H_{84}B_4N_6{\cdot}(CH_2Cl_2)_2$	
Formula weight	1458.69	
Crystal system	Triclinic	
Space group	$P\bar{1}$	
Unit cell dimensions	a = 11.0861(13) Å	α = 70.946(8)°
	b = 13.7773(13) Å	β = 74.605(9)°
	c = 14.2346(16) Å	γ = 82.408(9)°
Volume	1978.8(4) Å^3	
Z	1	
Density (calculated)	1.224 mg/m^3	
Absorption coefficient	0.200 mm^{-1}	
F(000)	766	
Crystal size	0.130 × 0.093 × 0.020 mm^3	
	**Data collection**	
Temperature	100(2) K	
Wavelength	0.71073 Å	
Theta range for data collection	2.307 to 25.680°	
Index ranges	-13 ≤ h ≤ 12, -16 ≤ k ≤ 11, -17 ≤ l ≤ 16	
Reflections collected	81468	
Independent reflections	7503 [R(int) = 0.1088]	
Completeness to theta = 25.242°	99.8%	
	**Refinement**	
Absorption correction	multi-scan	
Refinement method	Full-matrix least-squares on F^2	
Data / restraints / parameters	7503 / 22 / 479	
Goodness-of-fit on F^2	0.990	
Final R indices [I>2sigma(I)]	R1 = 0.0913, wR2 = 0.2602	
R indices (all data)	R1 = 0.1716, wR2 = 0.3002	
Extinction coefficient	0.015(3)	
Largest diff. peak and hole	0.897 and -1.138 e·Å^{-3}	

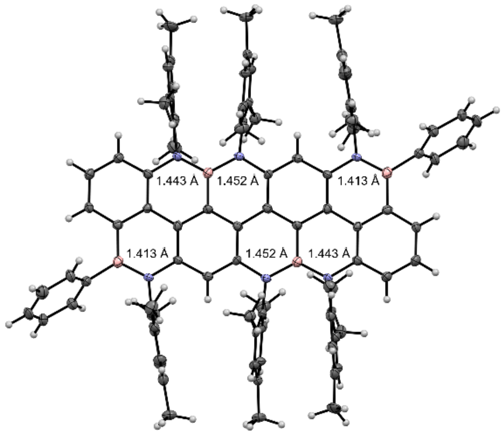

Figure S218. ORTEP representation (50% probability ellipsoids) of the X-ray crystal structure for **2**. B-N bond lengths reported in Å. Solvent molecules omitted for clarity. Atom colors: gray C, pink B, blue N.

Table S8. Crystal data and structure refinement for **3** (2173120).

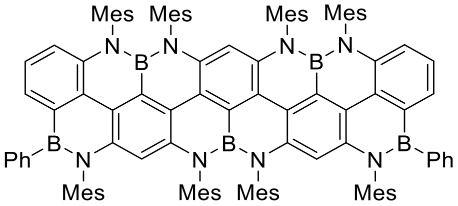

Empirical formula	$C_{114}H_{107}B_5N_8{\cdot}C_7H_8$	
Formula weight	1735.25	
Crystal system	Triclinic	
Space group	$P\bar{1}$	
Unit cell dimensions	a = 7.870(2) Å	α = 78.86(3)°
	b = 15.505(3) Å	β = 88.41(3)°
	c = 39.437(8) Å	γ = 78.46(3)°
Volume	4625.9(17) Å^3	
Z	2	
Density (calculated)	1.246 mg/m^3	
Absorption coefficient	0.055 mm^{-1}	
F(000)	1844	
Crystal size	0.080 × 0.050 × 0.010 mm^3	
	**Data collection**	
Temperature	100(2) K	
Wavelength	0.620 Å	
Theta range for data collection	0.918 to 22.213°	
Index ranges	-12 ≤ h ≤ 12, -23 ≤ k ≤ 24, -60 ≤ l ≤ 61	
Reflections collected	181110	
Independent reflections	17489 [R(int) = 0.0972]	
Completeness to theta = 21.839°	99.6%	
	**Refinement**	
Absorption correction	multi-scan	
Refinement method	Full-matrix least-squares on F^2	
Data / restraints / parameters	17489 / 3 / 1185	
Goodness-of-fit on F^2	1.055	
Final R indices [I>2sigma(I)]	R1 = 0.1541, wR2 = 0.3025	
R indices (all data)	R1 = 0.2139, wR2 = 0.3336	
Extinction coefficient	n/a	
Largest diff. peak and hole	0.562 and -0.440 e·Å^{-3}	

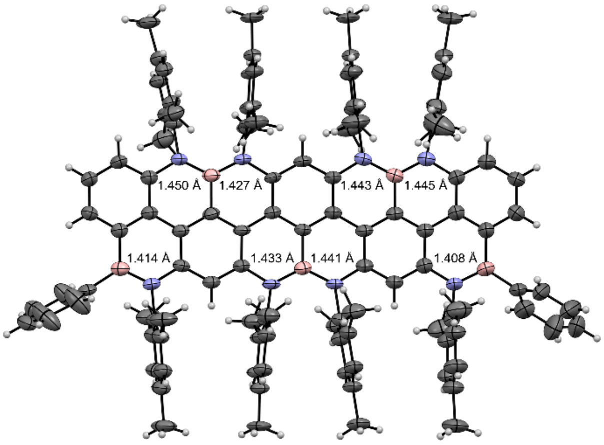

Figure S219. ORTEP representation (50% probability ellipsoids) of the X-ray crystal structure for **3**. B-N bond lengths reported in Å. Solvent molecules omitted for clarity. Atom colors: gray C, pink B, blue N.

5. Computational studies

Optimized S_0 geometries in CH_2Cl_2:

Table S9. Cartesian coordinates for S_0 optimized geometry of **5^H** in solution (CH_2Cl_2).

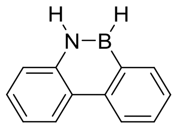

Atom	x	y	z
C	-3.5971848026	-0.2756460657	0.0000008564
C	-2.9182914662	-1.5014171784	-0.0000024200
C	-1.5324441188	-1.5428316769	-0.0000030336
C	-0.7691645810	-0.3562109027	-0.0000003236
C	-1.4544453827	0.8903249470	0.0000017085
C	-2.8639216566	0.8999801503	0.0000024899
C	0.7034447882	-0.3745030676	0.0000002120
C	1.4498676241	-1.5711993059	0.0000026265
C	1.4331043803	0.8422176745	-0.0000011417
C	2.8383555869	0.8304636010	-0.0000018413
C	3.5363381445	-0.3645208365	-0.0000003463
C	2.8353206974	-1.5767158937	0.0000023440
H	-3.3784888696	1.8557249260	0.0000042464
H	-4.6810534242	-0.2519259693	0.0000016173
H	-3.4787746341	-2.4299856988	-0.0000047160
H	3.3704285376	1.7765764148	-0.0000033756
N	0.7588910235	2.0613317613	-0.0000014671
B	-0.6441888156	2.1939797390	0.0000008666
H	1.3593636262	2.8752345181	-0.0000027851
H	-1.1247940862	3.2843190231	0.0000015842
H	0.9335830300	-2.5220595908	0.0000054921
H	-1.0491128394	-2.5110796876	-0.0000063866
H	4.6202284412	-0.3572651147	-0.0000009426
H	3.3714518287	-2.5184085432	0.0000044165

Table S10. Cartesian coordinates for S_0 optimized geometry of **17^H** in solution (CH_2Cl_2).

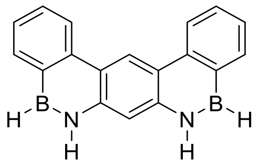

Atom	x	y	z
C	-5.0120682357	-1.8230094076	-0.0000174389
C	-3.8114170253	-2.5444529679	-0.0000470158
C	-2.5898040209	-1.8876968710	-0.0000410410
C	-2.5208502198	-0.4789663850	-0.0000056442
C	-3.7378710455	0.2573738772	0.0000198768
C	-4.9634347177	-0.4378522264	0.0000145576
C	-1.2376793166	0.2457087128	0.0000019182
C	-0.0000000000	-0.4072308776	-0.0000003685
C	-1.2160697515	1.6682504536	0.0000117340
C	0.0000000000	2.3489759020	-0.0000008419
C	1.2160697779	1.6682505171	-0.0000131503
C	1.2376792954	0.2457087017	-0.0000031054
H	-5.8864391513	0.1333708882	0.0000354058
H	-5.9627533311	-2.3439951750	-0.0000215213
H	-3.8320430405	-3.6288925453	-0.0000762099
H	-0.0000000691	3.4351075711	-0.0000008896
C	2.5208502304	-0.4789664178	0.0000046094
C	3.7378710879	0.2573738492	-0.0000178723
C	4.9634347230	-0.4378522296	-0.0000108979
C	5.0120681563	-1.8230094500	0.0000198261
C	3.8114170306	-2.5444529891	0.0000459760
C	2.5898039627	-1.8876968498	0.0000383774
H	5.9627532782	-2.3439951486	0.0000254285
H	3.8320429135	-3.6288925770	0.0000734981
H	5.8864392042	0.1333707316	-0.0000295066
B	3.6895006662	1.7918070927	-0.0000418876
N	2.4093781254	2.3840771047	-0.0000339337
H	4.6520425591	2.4939542696	-0.0000625584
H	2.3008755777	3.3895652471	-0.0000460709
N	-2.4093780883	2.3840771153	0.0000329645
B	-3.6895006080	1.7918071139	0.0000435353
H	-2.3008755142	3.3895652471	0.0000441838
H	-4.6520425485	2.4939541849	0.0000651035
H	1.6886682058	-2.4872876210	0.0000627026
H	0.0000000215	-1.4864200582	0.0000003433
H	-1.6886682058	-2.4872876369	-0.0000683580

Table S11. Cartesian coordinates for S_0 optimized geometry of $\mathbf{6^H}$ in solution (CH_2Cl_2).

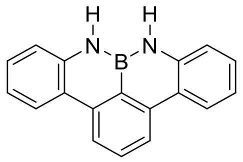

Atom	x	y	z
C	-4.8970426052	-1.2040579210	0.0000008299
C	-3.6728897619	-1.8520164045	0.0000001385
C	-2.4683169779	-1.1265581831	-0.0000003130
C	-2.4953279131	0.2947573958	-0.0000002141
C	-3.7588595260	0.9163108024	0.0000006777
C	-4.9430864570	0.1939392801	0.0000011736
C	-1.2376439094	1.0713560360	-0.0000004253
C	0.0000000380	0.3793076992	-0.0000005370
C	-1.2144939887	2.4751215724	-0.0000003522
C	0.0000000748	3.1544902370	-0.0000002369
C	1.2144940152	2.4751216571	-0.0000002131
C	1.2376438459	1.0713559778	-0.0000004233
H	-5.8943914147	0.7126838042	0.0000018714
H	-5.8130483052	-1.7839889334	0.0000012193
H	-3.6296422465	-2.9367117491	-0.0000000582
H	-0.0000001087	4.2392505328	-0.0000001429
C	2.4953278866	0.2947574048	-0.0000002772
C	3.7588595577	0.9163108024	0.0000002503
C	4.9430864358	0.1939392790	0.0000008607
C	4.8970425629	-1.2040579845	0.0000009889
C	3.6728897724	-1.8520164204	0.0000005617
C	2.4683169779	-1.1265581619	-0.0000001053
H	5.8130483052	-1.7839889122	0.0000015522
H	3.6296420930	-2.9367117702	0.0000008083
H	5.8943914147	0.7126837301	0.0000012497
N	1.2593822127	-1.8127421897	-0.0000007408
B	0.0000000000	-1.1462263664	-0.0000016010
N	-1.2593822339	-1.8127421791	-0.0000010353
H	1.3414819981	-2.8204929880	-0.0000004736
H	-1.3414818764	-2.8204929827	-0.0000007820
H	-3.8181424813	1.9969052088	0.0000011134
H	-2.1297119290	3.0522314723	-0.0000004014
H	2.1297120613	3.0522313718	-0.0000000290
H	3.8181424866	1.9969052141	0.0000002074

Table S12. Cartesian coordinates for S_0 optimized geometry of $\mathbf{1^H}$ in solution (CH_2Cl_2).

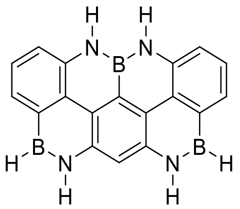

Atom	x	y	z
C	-4.9052356178	-1.5967054251	-0.0000044564
C	-3.6908550529	-2.2821324384	-0.0000059425
C	-2.4737668576	-1.5926568332	-0.0000032338
C	-2.4816694577	-0.1679408467	0.0000005400
C	-3.7234397110	0.5243806942	0.0000027017
C	-4.9234781448	-0.2061699322	-0.0000000229
B	-3.6960921663	2.0621966432	0.0000085385
C	-1.2324000596	0.5743931570	0.0000012167
C	0.0000000381	-0.1074070387	-0.0000003891
C	-1.2183215698	1.9931655029	0.0000027440
C	0.0000000256	2.6758677140	0.0000002483
C	1.2183215751	1.9931655293	-0.0000028008
C	1.2324000596	0.5743931623	-0.0000020477
N	-2.4272195307	2.6847707395	0.0000069574
H	-5.8697699105	0.3240769935	0.0000021293
H	-5.8328010616	-2.1585521980	-0.0000067075
H	-3.6810677617	-3.3679568726	-0.0000091825
H	0.0000000000	3.7624260269	0.0000006387
C	2.4816694365	-0.1679408626	-0.0000010025
C	3.7234397428	0.5243806514	-0.0000026984
C	4.9234781448	-0.2061699349	0.0000006812
C	4.9052355861	-1.5967054992	0.0000055331
C	3.6908550317	-2.2821324226	0.0000065123
C	2.4737668152	-1.5926568014	0.0000029931
H	5.8328010616	-2.1585522298	0.0000083711
H	3.6810676823	-3.3679568831	0.0000100382
H	5.8697699105	0.3240769104	-0.0000014474
B	3.6960921716	2.0621966168	-0.0000101655
N	2.4272195837	2.6847707342	-0.0000066078
N	1.2667165136	-2.2896387113	0.0000024990
B	-0.0000000280	-1.6331012016	-0.0000002542
N	-1.2667165506	-2.2896387272	-0.0000031346
H	-4.6710589125	2.7477182047	0.0000087230
H	-2.3443272072	3.6928030812	0.0000094404
H	4.6710589495	2.7477181517	-0.0000056815
H	2.3443272813	3.6928030600	-0.0000076634
H	1.3566563980	-3.2968415201	0.0000074208
H	-1.3566563451	-3.2968415466	-0.0000081282

Table S13. Cartesian coordinates for S_0 optimized geometry of $\mathbf{2^H}$ in solution (CH_2Cl_2).

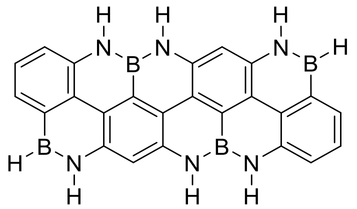

Atom	x	y	z
C	-2.9270535242	2.0145181101	-0.0000020396
C	-1.6597554078	2.6063865772	-0.0000025382
C	-0.4906904733	1.8413062970	-0.0000029925
C	-0.5921663197	0.4218976066	-0.0000023048
C	-1.8677051699	-0.1667670147	-0.0000018415
C	-3.0488645453	0.6032516985	-0.0000018289
B	-1.9777332191	-1.6896306363	-0.0000025334
C	0.5921663144	-0.4218975939	-0.0000022318
C	1.8677051223	0.1667670359	-0.0000015295
C	0.4906904315	-1.8413063499	-0.0000029346
C	1.6597553602	-2.6063865878	-0.0000026547
C	2.9270535083	-2.0145180361	-0.0000019478
C	3.0488645453	-0.6032516720	-0.0000013035
N	-0.7635120159	-2.4460865927	-0.0000034291
H	-1.5841080812	3.6899066404	-0.0000029406
H	1.5841081341	-3.6899066562	-0.0000034112
C	4.3477110426	0.0450717677	0.0000003916
C	5.5354861809	-0.7365458756	0.0000014790
C	6.7863397610	-0.0948891465	0.0000044866
C	6.8697566102	1.2925629731	0.0000065769
C	5.7074508345	2.0650752826	0.0000054163
C	4.4439482099	1.4671461543	0.0000017915
H	7.8356810197	1.7856206089	0.0000093938
H	5.7770140890	3.1485084337	0.0000075284
H	7.6914317578	-0.6929343393	0.0000051314
B	5.3934344332	-2.2676613045	-0.0000007406
N	4.0816201467	-2.7947822836	-0.0000016661
N	3.2892646074	2.2510349588	-0.0000007234
B	1.9777332402	1.6896306998	-0.0000020400
N	0.7635119947	2.4460866191	-0.0000030430
H	-0.7560737899	-3.4573742580	-0.0000029158
H	6.3150242703	-3.0240587604	0.0000003550
H	3.9242971650	-3.7941827767	-0.0000023591
H	3.4518681956	3.2493948249	-0.0000011476
H	0.7560735941	3.4573742844	-0.0000025133
C	-4.3477110320	-0.0450717799	-0.0000000875
C	-4.4439481411	-1.4671462019	0.0000013958
C	-5.7074507816	-2.0650752773	0.0000058157
C	-6.8697566102	-1.2925629837	0.0000073361

C	-6.7863397081	0.0948891391	0.0000049081
C	-5.5354861280	0.7365458174	0.0000013411
H	-7.8356809139	-1.7856206725	0.0000106880
H	-7.6914316520	0.6929344186	0.0000057652
H	-5.7770140890	-3.1485084072	0.0000083234
N	-3.2892645704	-2.2510349535	-0.0000011947
H	-3.4518681162	-3.2493948249	-0.0000014780
N	-4.0816202102	2.7947823048	-0.0000017982
B	-5.3934344861	2.2676612622	-0.0000017292
H	-3.9242972761	3.7941828032	-0.0000019217
H	-6.3150243233	3.0240586281	0.0000014776

Table S14. Cartesian coordinates for S_0 optimized geometry of **3^H** in solution (CH_2Cl_2).

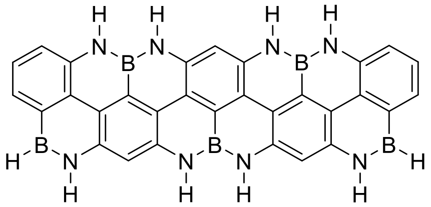

Atom	x	y	z
C	4.9179840206	1.9093802405	0.0000162386
C	3.6967938870	2.5933838624	-0.0000343636
C	2.4754450849	1.9154965394	-0.0000931121
C	2.4726131719	0.4922472126	-0.0001118884
C	3.7011878835	-0.1883068555	-0.0000661815
C	4.9359872960	0.4936712697	0.0000030534
B	3.6975175263	-1.7148830679	-0.0000860395
C	1.2305775098	-0.2619159550	-0.0001547164
C	0.0000000000	0.4186139652	-0.0001495034
C	1.2236984495	-1.6823275992	-0.0001794410
C	-0.0000000485	-2.3615253977	-0.0001997036
C	-1.2236985130	-1.6823275886	-0.0001791130
C	-1.2305775256	-0.2619158814	-0.0001546158
N	2.4305479972	-2.3791535235	-0.0001699751
H	3.7004717956	3.6801804056	-0.0000207609
H	-0.0000000559	-3.4484268821	-0.0002157197
C	-2.4726131983	0.4922472396	-0.0001119321
C	-3.7011878835	-0.1883068317	-0.0000655495
C	-4.9359873066	0.4936712861	0.0000037553
C	-4.9179840259	1.9093802140	0.0000156212
C	-3.6967938976	2.5933838835	-0.0000360204
C	-2.4754450796	1.9154966135	-0.0000943351
H	-3.7004718379	3.6801804162	-0.0000232909
B	-3.6975175474	-1.7148830361	-0.0000846952

N	-2.4305480342	-2.3791534494	-0.0001690343
N	-1.2672543746	2.6107009008	-0.0001270665
B	0.0000000100	1.9468475638	-0.0001367752
N	1.2672543799	2.6107009166	-0.0001255403
H	2.3463924271	-3.3867901624	-0.0002087120
H	-2.3463925065	-3.3867900936	-0.0002077036
H	-1.3515877376	3.6183426779	-0.0001170018
H	1.3515878858	3.6183426938	-0.0001131018
C	6.1835872353	-0.2485204573	0.0000729083
C	6.1744223094	-1.6741320730	0.0000738299
C	7.3903438436	-2.3639243750	0.0001624372
C	8.6066431045	-1.6791883507	0.0002432116
C	8.6261157145	-0.2894423341	0.0002357353
C	7.4259976872	0.4431666446	0.0001522625
H	9.5335565173	-2.2422368321	0.0003110153
H	9.5730352538	0.2399517792	0.0002958963
H	7.3800090657	-3.4498554224	0.0001714918
N	4.9653989653	-2.3708945596	-0.0000078453
H	5.0560854711	-3.3779946816	0.0000160735
N	6.1273671312	2.6022896767	0.0000868532
B	7.3968301274	1.9805707837	0.0001499803
H	6.0438729692	3.6102503466	0.0000973451
H	8.3715815303	2.6674171297	0.0002267972
C	-6.1835871824	-0.2485205023	0.0000738874
C	-6.1744223094	-1.6741320360	0.0000747595
C	-7.3903438436	-2.3639243856	0.0001624953
C	-8.6066430516	-1.6791884300	0.0002425323
C	-8.6261156087	-0.2894423394	0.0002352364
C	-7.4259977401	0.4431666478	0.0001526945
H	-7.3800089069	-3.4498554489	0.0001713696
H	-9.5335565702	-2.2422367897	0.0003098166
H	-9.5730352538	0.2399516522	0.0002952228
N	-4.9653989494	-2.3708945543	-0.0000067335
H	-5.0560854393	-3.3779946921	0.0000172598
N	-6.1273671312	2.6022897243	0.0000857751
B	-7.3968301274	1.9805708101	0.0001523237
H	-6.0438729692	3.6102503942	0.0000936967
H	-8.3715815832	2.6674169868	0.0002202067

Table S15. Cartesian coordinates for S_0 optimized geometry of $\mathbf{4^H}$ in solution (CH_2Cl_2).

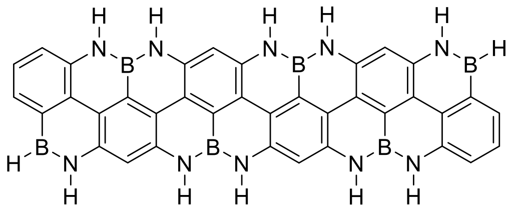

Atom	x	y	z
C	3.1244354163	1.7094380461	-0.0471023364
C	1.9196936238	2.4230076030	-0.0576502680
C	0.6777109746	1.7788175385	-0.0649966817
C	0.6310660516	0.3592285638	-0.0653383037
C	1.8413615061	-0.3556089626	-0.0584273705
C	3.0910433926	0.2903781327	-0.0475197690
B	1.7964567717	-1.8831140641	-0.0596193512
C	-0.6310657288	-0.3592299216	-0.0653211800
C	-1.8413610510	0.3556077454	-0.0584078698
C	-0.6777106730	-1.7788188932	-0.0649490240
C	-1.9196932269	-2.4230088571	-0.0575754624
C	-3.1244348131	-1.7094390356	-0.0470239995
C	-3.0910426888	-0.2903791704	-0.0474658461
N	0.5108027992	-2.5099899900	-0.0682083344
H	1.9502923116	3.5092706403	-0.0556749675
H	-1.9502918988	-3.5092718680	-0.0555810094
C	-4.3107109934	0.4993307591	-0.0309186408
C	-5.5579141398	-0.1457575921	-0.0088446002
C	-6.7726539154	0.5713209711	0.0155449752
C	-6.7139872663	1.9856515993	0.0148410944
C	-5.4738513239	2.6344801665	-0.0107657677
C	-4.2722701798	1.9225358028	-0.0315348508
H	-5.4472372029	3.7207212060	-0.0128181084
B	-5.5979089827	-1.6720481996	-0.0087112407
N	-4.3512745855	-2.3724963367	-0.0328684585
N	-3.0450370912	2.5834691188	-0.0500209226
B	-1.7964564066	1.8831127993	-0.0596379137
N	-0.5108025690	2.5099885718	-0.0682571494
H	0.3977331368	-3.5147888103	-0.0697065287
H	-4.2966030391	-3.3822324385	-0.0347181215
H	-3.1000294619	3.5932583447	-0.0471792188
H	-0.3977329855	3.5147873815	-0.0697810342
C	4.3107120570	-0.4993314592	-0.0309750097
C	4.2722709524	-1.9225366125	-0.0315369625
C	5.4738518531	-2.6344805105	-0.0107413776
C	6.7139888539	-1.9856511653	0.0148023267
C	6.7726561380	-0.5713205531	0.0154392204
C	5.5579155156	0.1457575032	-0.0089347216
H	5.4472372558	-3.7207216135	-0.0127401689

N	3.0450374034	-2.5834702671	-0.0499830636
H	3.1000295518	-3.5932594296	-0.0471027211
N	4.3512751252	2.3724958022	-0.0329639211
B	5.5979099882	1.6720482155	-0.0088084834
H	4.2966029967	3.3822319252	-0.0348078257
C	-8.0408187870	-0.1345162690	0.0439675706
C	-8.0726241365	-1.5599759283	0.0459803987
C	-9.3072601814	-2.2145211864	0.0787227844
C	-10.5033989019	-1.4952624771	0.1070879221
C	-10.4830071641	-0.1057600434	0.1034300503
C	-9.2626242956	0.5923424828	0.0725533777
H	-9.3278296699	-3.3001727595	0.0835503797
H	-11.4456569191	-2.0316395011	0.1322486971
H	-11.4140244519	0.4507537408	0.1253674222
N	-6.8844025420	-2.2912575385	0.0173245694
H	-7.0035301213	-3.2953751225	0.0196589168
N	-7.9026594552	2.7132157660	0.0416977831
B	-9.1892555161	2.1281563068	0.0710250335
H	-7.7903886895	3.7184652336	0.0426266205
H	-10.1427950093	2.8438454618	0.0933066728
C	8.0408196337	0.1345174808	0.0439049284
C	8.0726224431	1.5599769919	0.0460115275
C	9.3072549955	2.2145224987	0.0788753250
C	10.5033931868	1.4952640540	0.1072721583
C	10.4830043065	0.1057619241	0.1035131067
C	9.2626248247	-0.5923406730	0.0724991974
H	11.4456488227	2.0316410040	0.1325236596
H	11.4140219648	-0.4507515051	0.1254431876
H	9.3278221027	3.3001737808	0.0837764236
N	6.8844012190	2.2912580465	0.0173233594
H	7.0035271050	3.2953756728	0.0197173066
N	7.9026610427	-2.7132147182	0.0416699415
B	9.1892630304	-2.1281548886	0.0707230596
H	7.7903874195	-3.7184637625	0.0427615538
H	10.1427865953	-2.8438409850	0.0937811749

TD-DFT calculations (in CH_2Cl_2):

5^{H}

Excited State 1: Singlet-A 4.1167 eV 301.17 nm f=0.2126 <S**2>=0.000
46 -> 49 -0.23939
47 -> 48 0.63744
This state for optimization and/or second-order correction.
Total Energy, E(TD-HF/TD-DFT) = -542.969240741

17^{H}

Excited State 1: Singlet-A 3.5696 eV 347.34 nm f=0.5027 <S**2>=0.000
72 -> 75 0.16256
73 -> 74 0.67888
This state for optimization and/or second-order correction.
Total Energy, E(TD-HF/TD-DFT) = -853.795351499

29

Excited State 1: Singlet-A1 3.4624 eV 358.09 nm f=0.1468 <S**2>=0.000
70 -> 71 0.69578
This state for optimization and/or second-order correction.
Total Energy, E(TD-HF/TD-DFT) = -828.332572563

1^{H}

Excited State 1: Singlet-A 3.0529 eV 406.11 nm f=0.5254 <S**2>=0.000
82 -> 83 0.69586
This state for optimization and/or second-order correction.
Total Energy, E(TD-HF/TD-DFT) = -987.740306979

2^{H}

Excited State 1: Singlet-A 2.8018 eV 442.52 nm f=0.9376 <S**2>=0.000
111 ->112 0.69912
This state for optimization and/or second-order correction.
Total Energy, E(TD-HF/TD-DFT) = -1352.78522970

3^{H}

Excited State 1: Singlet-A 2.6404 eV 469.56 nm f=1.3430 <S**2>=0.000
140 -> 141 0.69938
This state for optimization and/or second-order correction.
Total Energy, E(TD-HF/TD-DFT) = -1717.82616235

4^H

Excited State 1: Singlet-A 2.5204 eV 491.92 nm f=1.6516 <S**2>=0.000
169 -> 170 0.69772
This state for optimization and/or second-order correction.
Total Energy, E(TD-HF/TD-DFT) = -2082.86551350

S_0, S_1 and T_1 optimized geometries in 2-MeTHF:

Table S16. Cartesian coordinates for S_0 optimized geometry of **5^H** in solution (2-MeTHF).

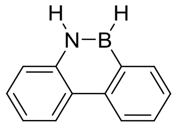

Atom	x	y	z
C	-3.59817366632515	-0.27512331653269	0.00000019874657
C	-2.91956604176521	-1.50168386305382	-0.00000037784993
C	-1.53396912535878	-1.54408288818485	-0.00000021784530
C	-0.76941185438693	-0.35720402695104	-0.00000020159164
C	-1.45486026912559	0.89006874707036	0.00000024190591
C	-2.86476097759464	0.90015414153681	0.00000023049778
C	0.70331851600947	-0.37526577600943	-0.00000003167064
C	1.45092101817345	-1.57215676337691	0.00000017053150
C	1.43311858391172	0.84278676678273	-0.00000024197010
C	2.83897866761505	0.83107573648490	-0.00000015667507
C	3.53710411127699	-0.36396099614603	0.00000002640175
C	2.83632234421720	-1.57661970097143	0.00000017894805
H	-3.38066444649357	1.85669543929044	0.00000060827398
H	-4.68356424202162	-0.25096329697661	0.00000016596368
H	-3.48133105941280	-2.43113945928333	-0.00000045127207
H	3.37045767033178	1.77891979647277	-0.00000032941655
N	0.75956496975963	2.06181343811999	-0.00000032481739
B	-0.64229394258634	2.19211480160497	0.00000008895178
H	1.35879367010534	2.87740755479880	-0.00000047805915
H	-1.12227605082839	3.28719367233029	0.00000027721249
H	0.93561662108496	-2.52496973499156	0.00000064437644
H	-1.05152909426308	-2.51424482048759	-0.00000066477831
H	4.62250710745582	-0.35642663263611	0.00000021152853
H	3.37369749022070	-2.51938881889066	0.00000043260770

Table S17. Cartesian coordinates for S_0 optimized geometry of $\mathbf{17^H}$ in solution (2-MeTHF).

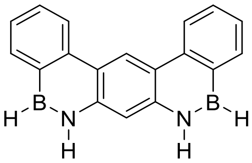

Atom	x	y	z
C	-5.01754725290397	-1.82035029282627	-0.00000018558838
C	-3.81774752345369	-2.54403024470683	-0.00000169468583
C	-2.59478750258974	-1.88996435128984	-0.00000244327073
C	-2.52286587394427	-0.48057993722397	-0.00000077840594
C	-3.73956188823656	0.25786660656460	0.00000058892625
C	-4.96665641488737	-0.43552015711281	0.00000093287411
C	-1.23854939370411	0.24289875681133	-0.00000071713058
C	-0.00000000111677	-0.41013441644105	0.00000014878395
C	-1.21683890937068	1.66663394724720	0.00000018095322
C	-0.00000006145205	2.34630497655218	-0.00000016828777
C	1.21683889977874	1.66663393759060	-0.00000040037262
C	1.23854928878164	0.24289879195175	-0.00000011621615
H	-5.89026741975538	0.13721252977105	0.00000173062076
H	-5.97034627276625	-2.34054185675170	0.00000023933460
H	-3.84057229087698	-3.62984317410366	-0.00000245352653
H	-0.00000001634035	3.43370466409990	0.00000025916846
C	2.52286564578230	-0.48058029849319	0.00000044357776
C	3.73956149366181	0.25786625257974	-0.00000048635588
C	4.96665635023428	-0.43552002175725	-0.00000041581967
C	5.01754747830748	-1.82035012625890	0.00000075102686
C	3.81774780443708	-2.54403033533829	0.00000173841672
C	2.59478753832368	-1.88996477003297	0.00000187914176
H	5.97034648198114	-2.34054161032839	0.00000073939427
H	3.84057272757660	-3.62984320356337	0.00000261025315
H	5.89026705738577	0.13721319014153	-0.00000118101569
B	3.68727471433167	1.79204162706186	-0.00000110304445
N	2.40864155167324	2.38405980918551	-0.00000077896646
H	4.65037392093065	2.50005126484944	-0.00000203449389
H	2.30112952531093	3.39049607745509	-0.00000127658339
N	-2.40864153182007	2.38405995298383	0.00000082034501
B	-3.68727474909746	1.79204197859627	0.00000118415621
H	-2.30112958689458	3.39049625869649	0.00000104170219
H	-4.65037363288790	2.50005196720117	0.00000215292500
H	1.69419740076248	-2.49270745241061	0.00000234416764
H	-0.00000010692527	-1.49032320868304	0.00000027715010
H	-1.69419745023604	-2.49270713201739	-0.00000382915405

Table S18. Cartesian coordinates for S_0 optimized geometry of $\mathbf{6^H}$ in solution (2-MeTHF).

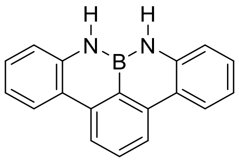

Atom	x	y	z
C	-4.89645571112343	-1.20760939695645	0.00000000932964
C	-3.67116823421437	-1.85365282493324	-0.00000028122168
C	-2.46640229032360	-1.12671977483891	-0.00000004385522
C	-2.49609458151662	0.29603184473173	-0.00000017635948
C	-3.76170556621818	0.91509782945190	-0.00000012740002
C	-4.94462577069828	0.19043753602205	-0.00000089504954
C	-1.23861362005770	1.07434057989537	0.00000015528171
C	-0.00000000668454	0.38297089187202	0.00000100761264
C	-1.21486723525961	2.47859781438432	0.00000029410955
C	-0.00000022788886	3.15763628277353	0.00000031060938
C	1.21486683966538	2.47859802132109	0.00000025101478
C	1.23861355811847	1.07434069860457	0.00000040420169
H	-5.89807184458826	0.70855898356361	-0.00000066053647
H	-5.81284003631353	-1.78984707240160	-0.00000043453561
H	-3.62435151920594	-2.93970217330059	0.00000065725113
H	-0.00000031953166	4.24383837715261	0.00000020406756
C	2.49609483137707	0.29603192250044	0.00000035260419
C	3.76170575842446	0.91509775478865	0.00000020678472
C	4.94462587352490	0.19043736986289	-0.00000093119514
C	4.89645572081099	-1.20760953571976	-0.00000043627522
C	3.67116820485170	-1.85365299633872	-0.00000027466897
C	2.46640241854891	-1.12671978875712	0.00000050935296
H	5.81284001586418	-1.78984724464156	-0.00000125442814
H	3.62435162921921	-2.93970236847697	0.00000053579488
H	5.89807195102330	0.70855885690965	-0.00000071946390
N	1.25772113105245	-1.81115679598650	0.00000063343773
B	0.00000008311355	-1.14269594482354	0.00000069273453
N	-1.25772100151691	-1.81115686692785	0.00000020260963
H	1.33402899205245	-2.82035464289783	0.00000034162287
H	-1.33402911112538	-2.82035469694421	0.00000008130755
H	-3.82521063361699	1.99688407521705	-0.00000127953325
H	-2.13076455232045	3.05721943644483	0.00000063158684
H	3.82521117920936	1.99688402046254	-0.00000048014793

Table S19. Cartesian coordinates for S_0 optimized geometry of $\mathbf{1^H}$ in solution (2-MeTHF).

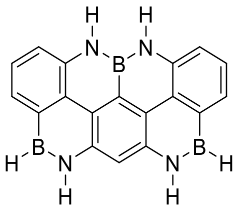

Atom	x	y	z
C	-4.90559591389280	-1.59675274832260	-0.00000072588625
C	-3.69099276940459	-2.28264417336401	-0.00000108366218
C	-2.47242882459415	-1.59437647051627	0.00000012231883
C	-2.48164249470310	-0.16834951228830	-0.00000015166368
C	-3.72402584012121	0.52426615731229	0.00000037932182
C	-4.92429149361607	-0.20650880198523	-0.00000020224679
B	-3.69385894418651	2.06203279553193	0.00000075314184
C	-1.23281951548526	0.57424942877838	0.00000139024492
C	0.00000004671488	-0.10731997346501	0.00000030931992
C	-1.21904936900683	1.99386419901378	0.00000071535966
C	-0.00000001136639	2.67573904403865	-0.00000003417470
C	1.21904914660755	1.99386387512834	-0.00000052903936
C	1.23281999115330	0.57424890947286	-0.00000015853913
N	-2.42702281713865	2.68581573714090	0.00000081802819
H	-5.87233432239688	0.32349273944583	0.00000042554684
H	-5.83431263431625	-2.15958466256925	-0.00000142376855
H	-3.68045482791082	-3.36980916739822	-0.00000182983445
H	-0.00000002523777	3.76347377502223	-0.00000085491890
C	2.48164294914606	-0.16835022474626	-0.00000043884079
C	3.72402575241907	0.52426618276788	-0.00000057190644
C	4.92429133174946	-0.20650869442540	-0.00000053192018
C	4.90559610239064	-1.59675263534393	0.00000045640401
C	3.69099315658965	-2.28264461286555	0.00000035196347
C	2.47242902771437	-1.59437723030883	0.00000090553176
H	5.83431307173440	-2.15958442601014	0.00000023111781
H	3.68045557130895	-3.36980968021822	0.00000115458246
H	5.87233395473555	0.32349322780857	-0.00000018947271
B	3.69385880737734	2.06203281344561	-0.00000120083980
N	2.42702247615376	2.68581543178856	-0.00000106932559
N	1.26608889628571	-2.29088909745813	0.00000106211150
B	-0.00000006129853	-1.63346588744003	0.00000011521186
N	-1.26608914890071	-2.29088900134112	-0.00000010006152
H	-4.67015256525493	2.75266730739570	0.00000096991615
H	-2.34524592792677	3.69485469857517	0.00000110058435
H	4.67015249955319	2.75266716824824	-0.00000093115880
H	2.34524549262632	3.69485435289124	-0.00000013221534
H	1.35238821495355	-3.29954050178475	0.00000093916109
H	-1.35238898245554	-3.29954034195488	-0.00000004039130

Table S20. Cartesian coordinates for S_0 optimized geometry of $\mathbf{2^H}$ in solution (2-MeTHF).

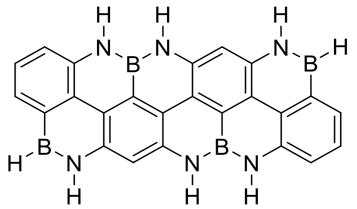

Atom	x	y	z
C	-2.92740586935444	2.01604582391296	-0.00000082560885
C	-1.65942546996059	2.60808053056008	-0.0000000426073
C	-0.48933337893997	1.84292816663398	-0.00000111962511
C	-0.59191469441073	0.42227810663212	-0.00000161589108
C	-1.86754514594810	-0.16623394546480	-0.00000090040255
C	-3.04915102556942	0.60379676046653	-0.00000084096715
B	-1.97743259898765	-1.68995695529215	-0.00000069606328
C	0.59191479859515	-0.42227799050517	-0.00000082997612
C	1.86754492180473	0.16623403448156	-0.00000133359760
C	0.48933327703656	-1.84292802423692	-0.00000099537496
C	1.65942555688646	-2.60808031747686	-0.00000004663213
C	2.92740581310547	-2.01604532296080	-0.00000048807239
C	3.04915110619750	-0.60379616090104	-0.00000062798546
N	-0.76369783760215	-2.44711105627987	-0.00000111076430
H	-1.58275255340175	3.69313405785884	0.00000016540580
H	1.58275276456405	-3.69313379660253	-0.00000005911882
C	4.34782958466096	0.04460522440567	-0.00000023679876
C	5.53660218110839	-0.73676844111572	0.00000003951403
C	6.78732912272105	-0.09391837420010	0.00000132665467
C	6.86959149601495	1.29324482873990	0.00000148473013
C	5.70670143747033	2.06588149004953	0.00000132334526
C	4.44245400187656	1.46812369551602	-0.00000012708660
H	7.83654352363780	1.78761870715448	0.00000270711853
H	5.77572086508877	3.15091904354896	0.00000118605886
H	7.69453468191382	-0.69144182977460	0.00000139459515
B	5.39148964812969	-2.26770701897023	-0.00000017526931
N	4.08142761865079	-2.79575863167767	-0.00000043147065
N	3.28849787323396	2.25132647660815	-0.00000073222571
B	1.97743228987390	1.68995690732929	-0.00000118776416
N	0.76369786239373	2.44711123722342	-0.00000101197821
H	-0.75820867575968	-3.45930253550229	-0.00000005327654
H	6.31381896598999	-3.02967471878250	-0.00000010116438
H	3.92446889493290	-3.79559043778030	-0.00000077522448
H	3.44986673293779	3.25038397322273	0.00000016199821
H	0.75820934376925	3.45930262804102	-0.00000068766006
C	-4.34782907088788	-0.04460556867082	0.00000012081654
C	-4.44245418055487	-1.46812396210684	-0.00000009832850
C	-5.70670166329971	-2.06588167883299	0.00000087707851
C	-6.86959164980319	-1.29324488910999	0.00000133204352

C	-6.78732883493833	0.09391839582489	0.00000121897398
C	-5.53660173739166	0.73676807497811	0.00000037862290
H	-7.83654364357101	-1.78761844175382	0.00000199791809
H	-7.69453432026257	0.69144197212177	0.00000178771757
H	-5.77572092494117	-3.15091926342180	0.00000098457309
N	-3.28849805016828	-2.25132672774254	-0.00000050787158
H	-3.44986664487496	-3.25038427631705	0.00000022219705
N	-4.08142787461781	2.79575874025004	-0.00000021847001
B	-5.39148972758271	2.26770677034182	0.00000010111195
H	-3.92446962515118	3.79559050391425	-0.00000132298941
H	-6.31381916461475	3.02967421566329	0.00000035144506

Table S21. Cartesian coordinates for S_0 optimized geometry of $\mathbf{3^H}$ in solution (2-MeTHF).

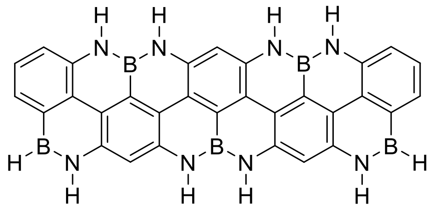

Atom	x	y	z
C	4.91764077223220	1.91033227165326	0.00000190582268
C	3.69538439245002	2.59333538356766	0.00000034980415
C	2.47316822885282	1.91630277421075	0.00000080955281
C	2.47203851066590	0.49168481894313	-0.00000062234346
C	3.70094339555972	-0.18833011751452	0.00000032278870
C	4.93606535410128	0.49373280314738	-0.00000112150939
B	3.69766256757508	-1.71557372087118	-0.00000084615206
C	1.23082635020877	-0.26357201194240	-0.00000001122907
C	0.00000022948515	0.41648616663070	-0.00000061625051
C	1.22518374063747	-1.68527155967649	-0.00000042237119
C	-0.00000026044675	-2.36340686512886	0.00000097467230
C	-1.22518391357513	-1.68527077687377	0.00000115875063
C	-1.23082602675021	-0.26357141911659	0.00000030676507
N	2.43126583325839	-2.38121995581751	-0.00000076457446
H	3.69889707272519	3.68130982484529	0.00000131092668
H	0.00000019160843	-3.45154467220427	0.00000149389119
C	-2.47203875949161	0.49168457636142	-0.00000001778198
C	-3.70094351481350	-0.18833049222459	-0.00000024549188
C	-4.93606532699628	0.49373267962456	0.00000093822230
C	-4.91764072214731	1.91033212871456	-0.00000083591190
C	-3.69538436368542	2.59333549701768	-0.00000174064460
C	-2.47316850101944	1.91630227878525	-0.00000081253407
H	-3.69889621530766	3.68131000265620	-0.00000297101284

B	-3.69766232768291	-1.71557389188043	0.00000186561202
N	-2.43126563513193	-2.38122017817428	0.00000228893543
N	-1.26601843610176	2.61075111216938	0.00000030004046
B	-0.00000008368627	1.94547129877294	-0.00000012860923
N	1.26601811064612	2.61075159449910	0.00000078199601
H	2.35055781278440	-3.39031573180595	-0.00000067954392
H	-2.35055784573566	-3.39031584884338	0.00000238650890
H	-1.34666056467010	3.61993691533751	-0.00000010106997
H	1.34666028496181	3.61993711605652	0.00000179023576
C	6.18341093978235	-0.24825263676872	-0.00000007861229
C	6.17336878345354	-1.67534316739714	-0.00000180539500
C	7.39084962077653	-2.36352218229952	-0.00000211196226
C	8.60722659107453	-1.67798020187415	-0.00000247111574
C	8.62681262150309	-0.28869907521812	-0.00000076425004
C	7.42625028459113	0.44406081824842	-0.00000062233056
H	9.53538710130436	-2.24195008723458	-0.00000268390166
H	9.57538821234612	0.24076210432749	-0.00000136105996
H	7.38024908658551	-3.45084070285674	-0.00000292941857
N	4.96507341783849	-2.37203073517182	-0.00000167526666
H	5.05243293329105	-3.38056210995780	-0.00000281861634
N	6.12593248359672	2.60383940524350	0.00000257604879
B	7.39360044456565	1.98130371174836	0.00000165428042
H	6.04346556683173	3.61277782993095	0.00000723713315
H	8.36944455918350	2.67383074528134	0.00000327051754
C	-6.18341109386458	-0.24825279442217	0.00000013754813
C	-6.17336864500625	-1.67534315525502	0.00000160978543
C	-7.39084957721780	-2.36352205534391	0.00000128698469
C	-8.60722655794322	-1.67798002275814	0.00000026166770
C	-8.62681259630148	-0.28869893812530	-0.00000096752781
C	-7.42625019030281	0.44406080229605	-0.00000088633076
H	-7.38024916913136	-3.45084056132402	0.00000257925577
H	-9.53538707069759	-2.24194991324964	0.00000005561991
H	-9.57538816894820	0.24076232193335	-0.00000164452997
N	-4.96507327619609	-2.37203076343752	0.00000314631311
H	-5.05243316906610	-3.38056211539166	0.00000337687098
N	-6.12593235578257	2.60383936684785	-0.00000226889110
B	-7.39360032672185	1.98130367123058	-0.00000231125956
H	-6.04346626911496	3.61277789911419	-0.00000360365477
H	-8.36944453094021	2.67383054096484	-0.00000323539717

Table S22. Cartesian coordinates for S_0 optimized geometry of $\mathbf{4^H}$ in solution (2-MeTHF).

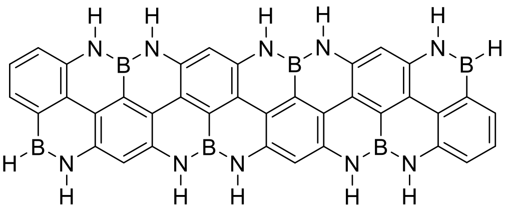

Atom	x	y	z
C	3.12551191089330	1.71154362983197	0.00701183096078
C	1.91929409500321	2.42439864530018	0.00720795315582
C	0.67602959837069	1.78097199027538	0.00746610235135
C	0.63059449122032	0.35989857950087	0.00767009291987
C	1.84128558421213	-0.35452452365045	0.00767170052574
C	3.09135103585281	0.29127213685814	0.00745530597424
B	1.79645609388596	-1.88272602092688	0.00784356768871
C	-0.63059554571507	-0.35989903698745	0.00764951496875
C	-1.84128601508886	0.35452472112080	0.00762584825848
C	-0.67602957652135	-1.78097269973921	0.00745524748503
C	-1.91929438680310	-2.42439878714711	0.00715700099722
C	-3.12551191176451	-1.71154297888487	0.00693032663358
C	-3.09135114556428	-0.29127153126233	0.00733750101316
N	0.51161509928148	-2.51099978542483	0.00786162016083
H	1.95073576959266	3.51206272830264	0.00723580416073
H	-1.95073630901258	-3.51206269069607	0.00718700254387
C	-4.31032301441418	0.49908050852366	0.00727405351470
C	-5.55806694086178	-0.14566666809118	0.00672326282621
C	-6.77350428653846	0.57137192448556	0.00666467068934
C	-6.71476750511048	1.98664270724296	0.00726423412614
C	-5.47343760282587	2.63475068075876	0.00776459441742
C	-4.27064464420778	1.92347953258110	0.00767364626906
H	-5.44559278916462	3.72232999064158	0.00843846592656
B	-5.59796579671523	-1.67257086940853	0.00617655789274
N	-4.35141618640878	-2.37375098227648	0.00667357761628
N	-3.04438868399179	2.58369717312192	0.00809810025033
B	-1.79645580874060	1.88272598760038	0.00780136393678
N	-0.51161487507597	2.51099974031937	0.00784669951695
H	0.40137548372011	-3.51717729023397	0.00776172838409
H	-4.29938261418886	-3.38471635415848	0.00638566199447
H	-3.09670305900399	3.59469147334305	0.00859726057887
H	-0.40137504790780	3.51717718643611	0.00775336959344
C	4.31032301828952	-0.49907910950601	0.00736267311761
C	4.27064521481282	-1.92347784905208	0.00774591798840
C	5.47343787996256	-2.63474942020724	0.00784740888181
C	6.71476839278407	-1.98664192620373	0.00735674940900
C	6.77350471601850	-0.57137109728339	0.00680765224990
C	5.55806716621588	0.14566804736405	0.00682874722510
H	5.44559163481212	-3.72232867981002	0.00851056699609

N	3.04438940413585	-2.58369624085467	0.00815227317497
H	3.09670441609816	-3.59469070031531	0.00864533225816
N	4.35141598934680	2.37375163003195	0.00675645749370
B	5.59796583430956	1.67257236389659	0.00626835904314
H	4.29938196027912	3.38471712248380	0.00644205671565
C	-8.04153869044076	-0.13478991115975	0.00594118652620
C	-8.07193965650854	-1.56175388229822	0.00519933076473
C	-9.30849527568895	-2.21501052384073	0.00432148202422
C	-10.50509510570748	-1.49514065213184	0.00433776045894
C	-10.48527406598250	-0.10593281591846	0.00515108197176
C	-9.26426396549251	0.59254261446558	0.00588977406232
H	-9.32930813584359	-3.30214902823211	0.00362599975268
H	-11.44877040340039	-2.03267064709124	0.00372378418235
H	-11.41825816810411	0.45049588020180	0.00509247741674
N	-6.88406256855569	-2.29251618268817	0.00548869069053
H	-7.00048007015167	-3.29797016970484	0.00508692931856
N	-7.90289372864710	2.71431147424140	0.00734544755633
B	-9.18789336453885	2.12822104335036	0.00677593437032
H	-7.79133186270282	3.72034344448741	0.00778907910503
H	-10.14336089576369	2.84852285733535	0.00665280270962
C	8.04153915422378	0.13479048544869	0.00602477550017
C	8.07193991269098	1.56175516912934	0.00527422087743
C	9.30849575912595	2.21501011095261	0.00437565809164
C	10.50509484998260	1.49513773663758	0.00439009748368
C	10.48527301382789	0.10593071337588	0.00518201255728
C	9.26426274198835	-0.59254261238186	0.00597040100431
H	11.44877078976105	2.03266545182997	0.00372171226299
H	11.41825461948419	-0.45050338379641	0.00522731562900
H	9.32931177651069	3.30214851246690	0.00369274183524
N	6.88406277817607	2.29251723739484	0.00555345391041
H	7.00048060128621	3.29797113894459	0.00513588973297
N	7.90289374172088	-2.71431156669896	0.00741257056549
B	9.18789404894831	-2.12822139260203	0.00681809218159
H	7.79133002728374	-3.72034310198721	0.00784541230426
H	10.14336109904630	-2.84852125763102	0.00656602326819

Table S23. Cartesian coordinates for S_1 optimized geometry of $\mathbf{5^H}$ in solution (2-MeTHF).

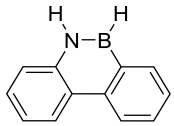

Atom	x	y	z
C	-3.61098007971049	-0.28280494594660	-0.00000003294545
C	-2.92694723257069	-1.51859943659386	-0.00000078818036
C	-1.53891799255809	-1.54928872797511	-0.00000020567579
C	-0.75992900148120	-0.35287650202297	0.00000025739996
C	-1.46832094645806	0.92526236670573	0.00000073381665
C	-2.87437283912808	0.90595795161793	0.00000076672094
C	0.67549195274331	-0.38712770532153	0.00000022927013
C	1.44244817884242	-1.58727408613846	0.00000128989340
C	1.44446259905258	0.86199205396668	0.00000063804052
C	2.85752445080878	0.84486165453942	-0.00000041989223
C	3.55439031999379	-0.36275155842254	-0.00000084950479
C	2.83733950821915	-1.57435503917409	0.00000049490807
H	-3.40645988213128	1.85427730510548	0.00000074115558
H	-4.69579285610950	-0.25638910699151	-0.00000071351699
H	-3.48355074119081	-2.45024059304792	-0.00000163086295
H	3.38650456131488	1.79321306867596	-0.00000168300463
N	0.78087146120105	2.05176067899373	0.00000049616267
B	-0.66725640460303	2.20141791859471	-0.00000021114761
H	1.37577114888106	2.87185203710466	0.00000076436003
H	-1.10570797099105	3.31198342540560	-0.00000028723447
H	0.93821987797406	-2.54447570308775	0.00000322691300
H	-1.05150196302697	-2.51719912353113	-0.00000147588034
H	4.63888719900821	-0.36758513237854	-0.00000169594810
H	3.37582665191997	-2.51661080007790	0.00000035515276

Table S24. Cartesian coordinates for S_1 optimized geometry of **17^H^** in solution (2-MeTHF).

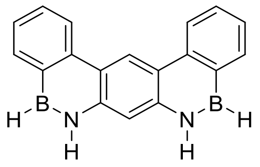

Atom	x	y	z
C	-5.01767453556810	-1.82270558417191	0.00000545546588
C	-3.81343066701302	-2.55059314900562	-0.00000550108083
C	-2.59563507927875	-1.89210551066207	-0.00001226555692
C	-2.51912735150170	-0.47042714633654	-0.00000741149331
C	-3.75594389595113	0.27541197179385	0.00000025985552
C	-4.97029591581006	-0.43115834413729	0.00000705224234
C	-1.25016912482669	0.22320507537406	-0.00000745942491
C	-0.00000021814521	-0.43727474523648	-0.00000772349419
C	-1.22264461836343	1.67690998929059	-0.00000436044599
C	0.00000046013459	2.35764024196807	-0.00000365194923
C	1.22264503174503	1.67691058750166	-0.00000542124061
C	1.25016919045557	0.22320566682120	-0.00000668843751
H	-5.90030516466648	0.13170348469135	0.00001586092147
H	-5.97093572394164	-2.34160263148333	0.00001226503059
H	-3.83478552010695	-3.63601359193146	-0.00000688542575
H	0.00000031412902	3.44420004946333	-0.00000010357479
C	2.51912670998949	-0.47042694597198	-0.00000393702061
C	3.75594362593471	0.27541221481595	-0.00000111573202
C	4.97029551752839	-0.43115799303707	0.00000392038850
C	5.01767432788147	-1.82270529184277	0.00000689716965
C	3.81343031893535	-2.55059305299725	0.00000662213925
C	2.59563475520560	-1.89210576775460	0.00000064901979
H	5.97093541644439	-2.34160241309216	0.00001042761611
H	3.83478550383465	-3.63601351842326	0.00001008165763
H	5.90030457150806	0.13170416764379	0.00000520882190
B	3.70590547838749	1.79330610980914	-0.00000225318575
N	2.39512015312103	2.37981926224136	-0.00000334444184
H	4.65123954638388	2.52260839250495	0.00000127299770
H	2.28732352955544	3.38695263616271	-0.00000232559875
N	-2.39511908524099	2.37981859508417	0.00000089276052
B	-3.70590535876686	1.79330560366480	0.00000642106505
H	-2.28732281450201	3.38695199112825	0.00000284923409
H	-4.65123868736129	2.52260872408199	0.00001083938905
H	1.69374109101990	-2.49247393704072	0.00000132202774
H	-0.00000009956781	-1.51524114365104	-0.00000990104402
H	-1.69374168158195	-2.49247399726566	-0.00001794865575

Table S25. Cartesian coordinates for S_1 optimized geometry of $\mathbf{6^H}$ in solution (2-MeTHF).

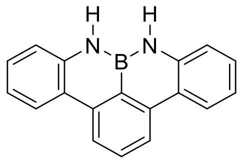

Atom	x	y	z
C	-4.89019867619600	-1.23314756298901	0.00000066655434
C	-3.65556472963681	-1.87624588973931	0.00000311694697
C	-2.46193602959489	-1.13493419095972	0.00000215606286
C	-2.47529780993059	0.30909535553569	-0.00000007370803
C	-3.75653911079413	0.91770649657754	-0.00000375425464
C	-4.92898332720072	0.16835382589182	-0.00000278509388
C	-1.24290689023991	1.07486405609026	0.00000162369737
C	0.00000017925759	0.39856482782034	0.00000000926657
C	-1.22288734385754	2.51173132389021	0.00000083754853
C	-0.00000077740441	3.17825078091176	0.00000154273908
C	1.22288640356232	2.51173177304550	0.00000106099086
C	1.24290688580691	1.07486453495743	0.00000027098293
H	-5.88633032047334	0.68062036389291	-0.00000547563116
H	-5.80766985893960	-1.81147242187449	0.00000099732151
H	-3.59523947951702	-2.96122674470491	0.00000456028750
H	-0.00000131124568	4.26592302809540	0.00000190742501
C	2.47529822210225	0.30909560766120	-0.00000059459087
C	3.75653958745903	0.91770623140732	0.00000058743555
C	4.92898367988424	0.16835325743093	-0.00000036131968
C	4.89019886253034	-1.23314809221431	-0.00000157732903
C	3.65556474078282	-1.87624624048193	-0.00000219908328
C	2.46193637520109	-1.13493396888455	-0.00000098629959
H	5.80767001150022	-1.81147291802835	-0.00000199030705
H	3.59523930469609	-2.96122707469012	-0.00000306735550
H	5.88633088400158	0.68061949446794	0.00000034042691
N	1.25308975519371	-1.79856166297933	-0.00000092240036
B	-0.00000000619216	-1.11058242973581	0.00000017886613
N	-1.25308956837629	-1.79856190540576	0.00000253764771
H	1.31347694439078	-2.81126421723090	-0.00000102197910
H	-1.31347646636172	-2.81126443178485	0.00000362089692
H	-3.83326529963165	1.99737896473399	-0.00000709258164
H	-2.13826006115568	3.09002506792966	0.00000200252535
H	2.13825879850802	3.09002608398835	0.00000251762573
H	3.83326643187113	1.99737867737509	0.00000136668601

Table S26. Cartesian coordinates for S_1 optimized geometry of $\mathbf{1^H}$ in solution (2-MeTHF).

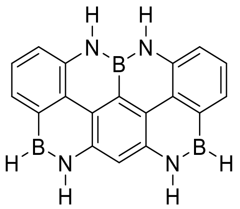

Atom	x	y	z
C	-4.89752762425708	-1.61352700125604	-0.00000133371046
C	-3.68192523841521	-2.29958340667825	0.00000041111690
C	-2.46482999174606	-1.59521925391770	0.00000188085944
C	-2.47251395853177	-0.15327758967106	0.00000246115660
C	-3.73546477626200	0.54023125161098	-0.00000008105583
C	-4.92086649467904	-0.21855383630607	-0.00000173167225
B	-3.71473189516256	2.06118370767469	-0.00000154973081
C	-1.24418409402037	0.57533592209602	0.00000362942045
C	0.00000027377411	-0.10753764517596	0.00000487602788
C	-1.22809810434443	2.00953405427012	0.00000094944143
C	0.00000005580950	2.69333681193943	0.00000113236497
C	1.22809798510407	2.00953447657408	0.00000157316383
C	1.24418436540532	0.57533620929005	0.00000308853018
N	-2.42390423357091	2.68781332132743	-0.00000122528726
H	-5.87724935746796	0.29740837447686	-0.00000269852593
H	-5.82706340353158	-2.17452515326110	-0.00000242464215
H	-3.66166137163381	-3.38600783664859	-0.00000060430061
H	-0.00000040752415	3.78038068786672	-0.00000041350198
C	2.47251385326576	-0.15327815867290	0.00000175673517
C	3.73546427227727	0.54023114721897	0.00000004802202
C	4.92086614821815	-0.21855367494744	-0.00000227174522
C	4.89752770849804	-1.61352683652441	-0.00000263124114
C	3.68192540911014	-2.29958389821252	-0.00000147385604
C	2.46483027374365	-1.59521963429111	0.00000061426802
H	5.82706366905985	-2.17452445550092	-0.00000302293075
H	3.66166172659034	-3.38600832503366	-0.00000227867979
H	5.87724873308081	0.29740909796223	-0.00000289791758
B	3.71473185660413	2.06118355358418	0.00000142569672
N	2.42390438621593	2.68781350747891	0.00000130722118
N	1.26745891025998	-2.28318899860314	0.00000108596194
B	0.00000009523659	-1.61009346564558	0.00000359979878
N	-1.26745906800218	-2.28318895591935	0.00000241953245
H	-4.68293237350862	2.76265490152831	-0.00000310841809
H	-2.34493243139065	3.69737668276346	-0.00000261440420
H	4.68293284843530	2.76265401139427	-0.00000073066620
H	2.34493274242366	3.69737667159281	0.00000042765479
H	1.34703780284095	-3.29369816049922	-0.00000084391509
H	-1.34703829190520	-3.29369810388452	0.00000124922863

Table S27. Cartesian coordinates for S_1 optimized geometry of $\mathbf{2^H}$ in solution (2-MeTHF).

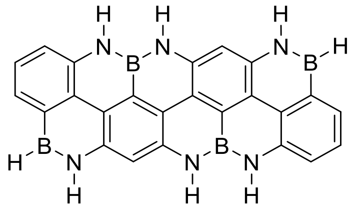

Atom	x	y	z
C	-2.92946724758764	2.02688250815619	-0.00000213822414
C	-1.65634421743833	2.61818919137981	-0.00000375525981
C	-0.48262075937379	1.84814360276210	-0.00000591100864
C	-0.58013232025192	0.41341732755645	-0.00000789884417
C	-1.87372916682538	-0.17560881460708	-0.00000743264734
C	-3.05403045584234	0.59992053433874	-0.00000324898030
B	-1.98088640755763	-1.67781202096056	-0.00000840507834
C	0.58013238132927	-0.41341699096574	-0.00000827694238
C	1.87372909241350	0.17560911711404	-0.00000704029893
C	0.48262036650781	-1.84814329577490	-0.00000726675556
C	1.65634405555246	-2.61818866311450	-0.00000474723087
C	2.92946700630383	-2.02688177056432	-0.00000130529116
C	3.05403047266282	-0.59991998286829	-0.00000370834751
N	-0.75944791348457	-2.44411531435120	-0.00000871781708
H	-1.57689427496780	3.70240979409206	-0.00000328194391
H	1.57689416420978	-3.70240921542905	-0.00000312900540
C	4.34343088219972	0.03808020907429	-0.00000171730467
C	5.54339854091867	-0.74964489518336	0.00000369359544
C	6.78694019894107	-0.09063830953246	0.00000780566023
C	6.86670753797964	1.29995269488068	0.00000729640667
C	5.70737494895963	2.07616673753743	0.00000279065270
C	4.43971084376784	1.46817374954850	-0.00000191504141
H	7.83536442360337	1.79117741857749	0.00001162857891
H	5.77208005223531	3.16086944000190	0.00000262508689
H	7.69971194094258	-0.68007844787083	0.00001231446302
B	5.40218226621294	-2.26942421710484	0.00000669389992
N	4.07430584550277	-2.79524091458621	0.00000279938659
N	3.29396674903024	2.24796257184443	-0.00000515218028
B	1.98088611859884	1.67781226828771	-0.00000679322559
N	0.75944767645101	2.44411550210529	-0.00000733481474
H	-0.75750264040065	-3.45720977871245	-0.00000759869831
H	6.31610048012750	-3.04111076265893	0.00001085294278
H	3.91883413316530	-3.79554002362296	0.00000462033885
H	3.45442257311729	3.24788749464979	-0.00000466285844
H	0.75750307919651	3.45720992779499	-0.00000720563790
C	-4.34343037492389	-0.03808067036440	-0.00000109395528
C	-4.43971063645112	-1.46817416901991	-0.00000218251410
C	-5.70737477976011	-2.07616705719012	0.00000214294704

C	-6.86670740697066	-1.29995298647203	0.00000821969526
C	-6.78693973494842	0.09063803215426	0.00001024249468
C	-5.54339811522533	0.74964452368967	0.00000537814038
H	-7.83536423649351	-1.79117758099487	0.00001210156471
H	-7.69971145307436	0.68007826176874	0.00001526280446
H	-5.77207988428161	-3.16086976123677	0.00000126694989
N	-3.29396656337628	-2.24796289796273	-0.00000680635224
H	-3.45442200514007	-3.24788789250268	-0.00000618103208
N	-4.07430629959356	2.79524121064276	0.00000096404048
B	-5.40218265998240	2.26942391483649	0.00000663496695
H	-3.91883514371153	3.79554033599809	-0.00000009974333
H	-6.31610113226677	3.04111006485931	0.00000967241807

Table S28. Cartesian coordinates for S_1 optimized geometry of **3^H^** in solution (2-MeTHF).

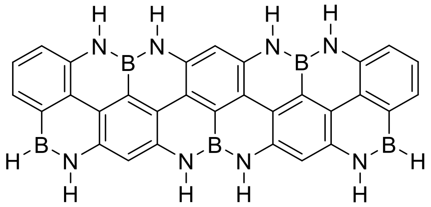

Atom	x	y	z
C	4.92085017293765	1.91799057715739	-0.00000335897520
C	3.69829315726487	2.60308041737831	-0.00000419504055
C	2.46870441372550	1.92331599147647	-0.00000019353201
C	2.46285940905170	0.49144644068855	0.00000027891934
C	3.70477123221688	-0.19422255370649	0.00000196998900
C	4.93968479420057	0.48870248050304	-0.00000172267255
B	3.69910516055326	-1.70426408700146	0.00000367682402
C	1.23808993949670	-0.24993065209318	0.00000359356044
C	0.00000027810963	0.43508188854862	0.00000348455472
C	1.22768840138622	-1.68727351533428	0.00000250394718
C	-0.00000004394575	-2.36681153794026	0.00000223137542
C	-1.22768825094883	-1.68727280987640	0.00000139062277
C	-1.23808923038136	-0.24992999296881	0.00000251002571
N	2.42287377633318	-2.37455994230800	0.00000428247795
H	3.70163476813362	3.69049579722577	-0.00000500023055
H	0.00000037363404	-3.45416968137831	0.00000235504442
C	-2.46285952046273	0.49144687509369	0.00000357889740
C	-3.70477093099466	-0.19422188316837	-0.00000028093817
C	-4.93968479670768	0.48870300073000	0.00000059837991
C	-4.92085051948354	1.91799102326453	0.00000350331034
C	-3.69829362690515	2.60308135418092	0.00000475648188
C	-2.46870473127431	1.92331637159673	0.00000403150329
H	-3.70163469532115	3.69049666270923	0.00000585410035
B	-3.69910487314849	-1.70426344622761	-0.00000075409725

N	-2.42287370194428	-2.37455954345808	0.00000051012999
N	-1.27267693364463	2.61374719509057	0.00000500850288
B	-0.00000004677281	1.94299761983262	0.00000369795956
N	1.27267682381933	2.61374705039767	0.00000213408699
H	2.34480169960138	-3.38460982846383	0.00000472022515
H	-2.34480266974286	-3.38460937678356	0.00000152661021
H	-1.35276373389485	3.62367367115809	0.00000625502711
H	1.35276439039677	3.62367340212673	0.00000089170455
C	6.18071532870695	-0.24642627279813	-0.00000153303713
C	6.16983546945209	-1.67734681353555	0.00000042908727
C	7.38691534604673	-2.37508440896331	0.00000148553923
C	8.60427534719926	-1.68794081582775	-0.00000006592571
C	8.62830202284254	-0.29830785377333	-0.00000248305756
C	7.43347777792019	0.44863660832826	-0.00000454459081
H	9.53250581280071	-2.25199943287272	0.00000167116591
H	9.58097735212260	0.22422933704089	-0.00000398991088
H	7.37157067408524	-3.46194727475539	0.00000335421256
N	4.96418380875490	-2.36957538348473	0.00000335481058
H	5.04811837683376	-3.37883072796536	0.00000471881054
N	6.12302955634582	2.60095030495237	-0.00000578526872
B	7.40386906474958	1.97552766656227	-0.00000675464648
H	6.04353422295053	3.61019005615401	-0.00000510524161
H	8.37412365671477	2.67578072504501	-0.00000876153459
C	-6.18071516591550	-0.24642619553435	-0.00000267528532
C	-6.16983531170962	-1.67734665579936	-0.00000333196617
C	-7.38691505793482	-2.37508443861181	-0.00000519903900
C	-8.60427524960279	-1.68794089502257	-0.00000566311727
C	-8.62830200537783	-0.29830804385552	-0.00000532051686
C	-7.43347768856696	0.44863650023321	-0.00000307608855
H	-7.37157011774131	-3.46194727126681	-0.00000517709542
H	-9.53250555781967	-2.25199981350663	-0.00000685868124
H	-9.58097742495009	0.22422901730223	-0.00000559537641
N	-4.96418333663236	-2.36957499242438	-0.00000178030445
H	-5.04811783934971	-3.37883035546017	-0.00000203928114
N	-6.12303007299251	2.60095034414213	0.00000411669519
B	-7.40386958347206	1.97552754525782	0.00000064803224
H	-6.04353543102247	3.61019036624083	0.00000616156892
H	-8.37412445972616	2.67578020574856	-0.00000003873143

Table S29. Cartesian coordinates for S_1 optimized geometry of $\mathbf{4^H}$ in solution (2-MeTHF).

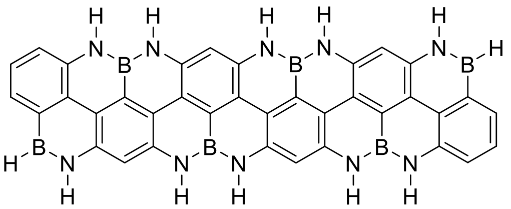

Atom	x	y	z
C	3.12684070824823	1.71611548419713	0.00697922703328
C	1.92143709293491	2.43049937667489	0.00702235366014
C	0.67294741162258	1.78616615061414	0.00711233770261
C	0.62314905667411	0.35341700534244	0.00709491857602
C	1.84096129967586	-0.36716217465487	0.00704088388183
C	3.09503103546627	0.27979552373924	0.00697277305040
B	1.79605726368219	-1.87768076284192	0.00709675415123
C	-0.62315010947994	-0.35341640722954	0.00709209061115
C	-1.84096197835591	0.36716337155262	0.00702710590942
C	-0.67294706661298	-1.78616536788101	0.00709803203827
C	-1.92143668476291	-2.43049857623620	0.00699542462970
C	-3.12684072381766	-1.71611504985352	0.00694569974165
C	-3.09503129019379	-0.27979520193788	0.00694783184161
N	0.50270682726433	-2.50949205974144	0.00719844257439
H	1.95334453347976	3.51744517880346	0.00701674472848
H	-1.95334400471809	-3.51744431651546	0.00698406097530
C	-4.30172244122216	0.49930874648594	0.00684801864950
C	-5.56054538249996	-0.14929218705697	0.00667592746957
C	-6.77569451365317	0.56911456983576	0.00655754986382
C	-6.71551057388915	1.99356409231437	0.00659505161005
C	-5.47315796324119	2.64269738276273	0.00674865475010
C	-4.26505422829361	1.92746015847025	0.00689122155149
H	-5.44441727776544	3.72985982569046	0.00682390685846
B	-5.59927538410427	-1.66172687926914	0.00667696064826
N	-4.34402389147067	-2.36871761434410	0.00688910058748
N	-3.04591618539673	2.58378733496622	0.00707260666230
B	-1.79605748751798	1.87768194740641	0.00709115562002
N	-0.50270667609797	2.50949302389431	0.00720850421980
H	0.39344037888712	-3.51650054605926	0.00726705087606
H	-4.29559120977194	-3.38044860869188	0.00692108836348
H	-3.09667771451768	3.59549285863233	0.00708165743488
H	-0.39344038378271	3.51650148053307	0.00728204960437
C	4.30172262947175	-0.49930775819745	0.00688459827229
C	4.26505377635308	-1.92745926791597	0.00692047090893
C	5.47315713531469	-2.64269714472055	0.00678425872018
C	6.71550991181014	-1.99356438306352	0.00665019374336
C	6.77569450204808	-0.56911489905057	0.00661939504802
C	5.56054567702123	0.14929249278580	0.00672845744222
H	5.44441561723791	-3.72985949572889	0.00685221843685

N	3.04591601596035	-2.58378628743616	0.00708454860591
H	3.09667779466396	-3.59549198457633	0.00708965907754
N	4.34402407808601	2.36871772870313	0.00693789673883
B	5.59927584165039	1.66172735380742	0.00673285880601
H	4.29559118693997	3.38044872856804	0.00697334506664
C	-8.03926566586801	-0.13100219857993	0.00636863719596
C	-8.06987570315013	-1.56070899371799	0.00630019424453
C	-9.30545240007601	-2.22123139544886	0.00602164459566
C	-10.50376771169480	-1.49961241330794	0.00587490597497
C	-10.48721951106588	-0.11097905983805	0.00598686597095
C	-9.26986664720739	0.59936620532511	0.00622548009904
H	-9.32248689577543	-3.30814797298152	0.00591701119211
H	-11.44747706508107	-2.03742092680414	0.00566975762781
H	-11.42363422012941	0.44022766364335	0.00590146081587
N	-6.88262029700428	-2.28837761613887	0.00649743083929
H	-6.99654972784418	-3.29444221892467	0.00647065606555
N	-7.89905844422960	2.71325735590933	0.00648517951800
B	-9.19506070018199	2.12606747440814	0.00632886419275
H	-7.78941663476106	3.71952268695615	0.00646205612072
H	-10.14542077250237	2.85354784309201	0.00618906904198
C	8.03926561745073	0.13100147243488	0.00644271852218
C	8.06987630455666	1.56070805009450	0.00636341193348
C	9.30545317960030	2.22123026929021	0.00607798870925
C	10.50376855027428	1.49961143858346	0.00594295356675
C	10.48721992754699	0.11097793829849	0.00607116851980
C	9.26986686219074	-0.59936708331106	0.00631298548346
H	11.44747770699962	2.03742030269188	0.00573037949889
H	11.42363427064237	-0.44022943395367	0.00600094003568
H	9.32248820975881	3.30814686888738	0.00596238575013
N	6.88262110706815	2.28837732251585	0.00655615980748
H	6.99655088233981	3.29444185955618	0.00652743957693
N	7.89905768441912	-2.71325791978328	0.00655432869340
B	9.19506007052287	-2.12606839823028	0.00643699655081
H	7.78941516378681	-3.71952304683458	0.00652232045069
H	10.14542025608734	-2.85354891660962	0.00628552266401

Table S30. Cartesian coordinates for T_1 optimized geometry of $\mathbf{5^H}$ in solution (2-MeTHF).

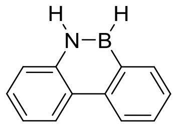

Atom	x	y	z
C	-3.59434048379449	-0.29608797130024	0.00000516225126
C	-2.91025338547991	-1.52512283978976	0.00000077274154
C	-1.53405334670319	-1.57623240831264	-0.00000420930812
C	-0.73432044695394	-0.34853374343887	-0.00000480116544
C	-1.46086696023024	0.92031109210510	-0.00000216386710
C	-2.83926048646907	0.91703378497437	0.00000248995827
C	0.66402270867200	-0.36646883481840	-0.00000498660332
C	1.44590175448468	-1.60413146964047	-0.00000349265505
C	1.44724465301029	0.87980114835870	-0.00000315921782
C	2.82518039299473	0.84928321342051	0.00000192475084
C	3.53446556393003	-0.37639117780503	0.00000445030545
C	2.81619601538210	-1.59407037297059	0.00000144741329
H	-3.37191542192361	1.86434653350806	0.00000532603218
H	-4.67813276089812	-0.26768402017449	0.00000918813927
H	-3.47647903558257	-2.45208802666439	0.00000126517973
H	3.37150321874286	1.78843892224292	0.00000456372650
N	0.76213463991981	2.09501729712325	-0.00000433339712
B	-0.64226270151794	2.22363897012414	-0.00000364471730
H	1.35855289857117	2.91282326726770	-0.00000362334642
H	-1.12197504874621	3.31802362009516	-0.00000287188160
H	0.92868582294687	-2.55381098081505	-0.00000425544300
H	-1.04879487616729	-2.54330685870440	-0.00000719662877
H	4.61817530800560	-0.37500485061349	0.00000837814471
H	3.35859197780644	-2.53478429417207	0.00000376958802

Table S31. Cartesian coordinates for T_1 optimized geometry of **17^{H}** in solution (2-MeTHF).

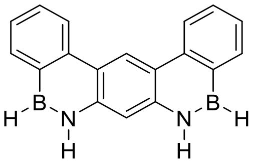

Atom	x	y	z
C	-5.04347844761289	-1.81757413497268	-0.00004765327164
C	-3.84547351375118	-2.55012465956794	-0.00013198384625
C	-2.62049323905733	-1.90253851706701	-0.00012250278363
C	-2.53813003393965	-0.49247439578346	-0.00003570064431
C	-3.75484688242101	0.25748277177311	0.00005317682742
C	-4.98589075858760	-0.43506544834253	0.00004574205578
C	-1.24618078274161	0.20992541979991	-0.00003124406888
C	-0.01211462223069	-0.44695357243719	-0.00010155490707
C	-1.22387076511838	1.63102111612502	0.00002989103145
C	0.03171063396128	2.34154869790188	-0.00003182084747
C	1.23928143156570	1.68673709529390	-0.00007400196318
C	1.28155541422084	0.24048609091066	-0.00004566730872
H	-5.90717132486422	0.14149081340162	0.00011407241618
H	-5.99895743513089	-2.33274488505208	-0.00005050228709
H	-3.87491450741851	-3.63567952746919	-0.00020380421415
H	0.00788736972213	3.42807954769263	-0.00003902354023
C	2.51192129802390	-0.44834934978354	0.00003859908410
C	3.77031293247794	0.29255113691002	-0.00003801384471
C	4.97103248362320	-0.40828313882790	-0.00002915509273
C	5.01366749687782	-1.81238649504383	0.00007806602842
C	3.80235033922281	-2.54034979247063	0.00021177921020
C	2.58896830230025	-1.89505758236438	0.00019369489935
H	5.96482436940023	-2.33398461061541	0.00007509001644
H	3.83007905077963	-3.62596861154892	0.00033668329844
H	5.90468061466692	0.14822137485732	-0.00009766324585
B	3.71732437086725	1.82215411106969	-0.00012667044025
N	2.43122096831304	2.40647407223606	-0.00012744302692
H	4.67554461708492	2.53573440730174	-0.00019182384251
H	2.31812216359367	3.41223957427108	-0.00016248750829
N	-2.38197429245495	2.35171520873032	0.00011585603545
B	-3.69381991452410	1.77924835339649	0.00014311078888
H	-2.26461973846887	3.35820365955255	0.00014912459279
H	-4.63327184832836	2.51550166223034	0.00022506781516
H	1.68699037139290	-2.49321330273021	0.00034499042524
H	0.00001937359599	-1.52419953931837	-0.00028694588018
H	-1.72228549504018	-2.50886755005903	-0.00017928196123

Table S32. Cartesian coordinates for T_1 optimized geometry of $\mathbf{6^H}$ in solution (2-MeTHF).

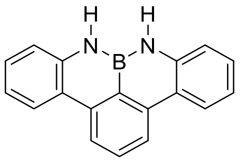

Atom	x	y	z
C	-4.89127140160460	-1.20747373296632	0.00000724210387
C	-3.66729124585918	-1.86509411605296	0.00000057011982
C	-2.46238968504075	-1.15024240324467	-0.00000400785751
C	-2.46561941179511	0.29882060584475	-0.00000374668347
C	-3.75025413667681	0.92665244453703	0.00000411476511
C	-4.92267632452987	0.19965186568439	0.00000852542404
C	-1.24112035298837	1.04191780364471	-0.00000637775838
C	0.00000013432948	0.36466059568511	-0.00000854528028
C	-1.21717551527063	2.50623744827161	-0.00000537657263
C	-0.00000049859482	3.17577980076037	-0.00000486845397
C	1.21717489865961	2.50623771497041	-0.00000487594436
C	1.24112013719412	1.04191801340351	-0.00000648378680
H	-5.87613383114350	0.71907327753798	0.00001397851586
H	-5.81394670255170	-1.77780659142662	0.00001081481198
H	-3.62774237467792	-2.95129978732375	-0.00000003362283
H	-0.00000078269938	4.26264077081911	-0.00000252014227
C	2.46561943911508	0.29882068338421	-0.00000355821891
C	3.75025422041882	0.92665201930075	0.00000309586507
C	4.92267640593371	0.19965140332410	0.00000838935574
C	4.89127168083669	-1.20747423356530	0.00000795470831
C	3.66729142711700	-1.86509439210139	0.00000093424719
C	2.46238990371451	-1.15024241448657	-0.00000375450250
H	5.81394708251497	-1.77780676835590	0.00001211460409
H	3.62774237085706	-2.95130003282227	-0.00000009518609
H	5.87613387583823	0.71907298596956	0.00001352458821
N	1.25815147986675	-1.82661394385805	-0.00000837349405
B	0.00000008378329	-1.15213050304172	-0.00001003052541
N	-1.25815116461664	-1.82661395921940	-0.00000821057094
H	1.32971086977665	-2.83762461034313	-0.00000781199254
H	-1.32971050600702	-2.83762459751587	-0.00000779988233
H	-3.81163627573034	2.00715510313558	0.00000467771538
H	-2.13374285006091	3.08017216074720	-0.00000107472790
H	2.13374205423497	3.08017271275612	-0.00000182202229
H	3.81163699565661	2.00715467654743	0.00000343040081

Table S33. Cartesian coordinates for T_1 optimized geometry of $\mathbf{1^H}$ in solution (2-MeTHF).

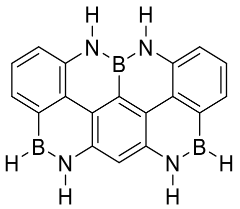

Atom	x	y	z
C	-4.90007741373691	-1.61049260120598	-0.00000141057450
C	-3.68525062432724	-2.29850926825384	0.00000024706782
C	-2.46483904667481	-1.59545301246006	0.00000148332811
C	-2.47284981495871	-0.15299489311734	0.00000315143387
C	-3.73602215298595	0.54123466964429	0.00000099295979
C	-4.92347271110232	-0.21725518019919	-0.00000093285613
B	-3.71321809594583	2.05785749132084	0.00000022062790
C	-1.24485510981471	0.57229721525896	0.00000319006849
C	0.00000019983615	-0.11310257864089	0.00000309211500
C	-1.22717658299555	2.00861291757890	0.00000083968826
C	0.00000023075439	2.69244253072431	0.00000054697558
C	1.22717646781250	2.00861315427420	-0.00000050242950
C	1.24485567329849	0.57229738754316	0.00000048140921
N	-2.42050239921181	2.68541189037988	0.00000040243948
H	-5.87916814117679	0.29996787964830	-0.00000213099300
H	-5.82962565906111	-2.17140313820090	-0.00000271160869
H	-3.66611594640561	-3.38459408790467	-0.00000115810965
H	0.00000011249903	3.77914578669006	0.00000024354218
C	2.47284970639168	-0.15299579373636	0.00000037843446
C	3.73602148583837	0.54123439697438	-0.00000020655394
C	4.92347213403919	-0.21725473957387	-0.00000033747487
C	4.90007745264019	-1.61049214171968	0.00000067088775
C	3.68525084483887	-2.29850952168905	-0.00000063222175
C	2.46483921158925	-1.59545382502725	-0.00000036018804
H	5.82962596078025	-2.17140243014258	0.00000159000234
H	3.66611669858593	-3.38459436498869	-0.00000077774411
H	5.87916711072962	0.29996920657956	-0.00000042884535
B	3.71321797933979	2.05785741815579	-0.00000189645681
N	2.42050277887863	2.68541195646987	-0.00000090593850
N	1.27143940689966	-2.28230056955429	-0.00000063388594
B	-0.00000002834146	-1.60831781984849	0.00000133345133
N	-1.27143970484760	-2.28230018356258	0.00000136330682
H	-4.67878483688307	2.76234361359571	-0.00000028629767
H	-2.34176706657714	3.69511041168590	-0.00000040989523
H	4.67878532480641	2.76234266528645	-0.00000186426916
H	2.34176740535406	3.69511015216346	-0.00000076689396
H	1.35212906601345	-3.29291749399082	-0.00000233462057
H	-1.35212991587931	-3.29291710015747	0.0000046011898

Table S34. Cartesian coordinates for T_1 optimized geometry of $\mathbf{2^H}$ in solution (2-MeTHF).

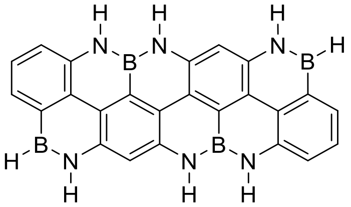

Atom	x	y	z
C	-2.92903859826316	2.02269520338492	-0.00000391601334
C	-1.65301430925347	2.61754260675270	-0.0000126199698
C	-0.48136238122890	1.85193386557448	0.00000276859315
C	-0.57781530683196	0.41148964187236	0.00000267423423
C	-1.87547618632104	-0.17923419897811	0.00000163247430
C	-3.05388703034126	0.59690487746652	0.00000002984022
B	-1.98266588958349	-1.67809240924039	0.00000276785110
C	0.57781527011242	-0.41148888166290	0.00000134567277
C	1.87547606457895	0.17923473280294	0.00000079681762
C	0.48136196156734	-1.85193312725904	0.00000046166381
C	1.65301409195620	-2.61754177407436	-0.00000412474024
C	2.92903819641260	-2.02269427922500	-0.00000397240630
C	3.05388697097516	-0.59690420552809	-0.00000153118576
N	-0.76016323334954	-2.44635761761256	0.00000170610295
H	-1.57669494510740	3.70179722976983	-0.00000344820350
H	1.57669471775312	-3.70179635218725	-0.00000498849608
C	4.34485561927514	0.03944606078688	-0.00000190141513
C	5.54302904734377	-0.74737442485181	-0.00000350681831
C	6.78854211732558	-0.09051938265408	0.00000029934393
C	6.86891457104353	1.29924576296579	0.00000572405043
C	5.71021901387563	2.07553758220810	0.00000629542019
C	4.44123033143230	1.46939471807127	0.00000209013691
H	7.83750038865760	1.79050395133513	0.00000994186000
H	5.77583427626288	3.16006405716214	0.00000998216976
H	7.70009191924946	-0.68160943579329	-0.00000036648099
B	5.40010502759574	-2.26614454964041	-0.00000713016740
N	4.07160083732167	-2.79045848686564	-0.00000809972967
N	3.29677989951000	2.24865369827197	0.00000378214541
B	1.98266574981480	1.67809293735261	0.00000239071144
N	0.76016312491495	2.44635809274722	0.00000294815654
H	-0.75838171794317	-3.45951082085650	0.00000157541942
H	6.31208245509937	-3.03931348885816	-0.00001090630958
H	3.91589933716591	-3.79088416990444	-0.00001048070897
H	3.45742504081210	3.24867416763733	0.00000574853823
H	0.75838238209391	3.45951124799572	0.00000225920688
C	-4.34485512048883	-0.03944655512385	0.00000011502526
C	-4.44123005435405	-1.46939520469769	0.00000343984155
C	-5.71021874623465	-2.07553818585653	0.00000689887039
C	-6.86891435043653	-1.29924639322565	0.00000591475924

C	-6.78854166852740	0.09051876801744	0.00000131524574
C	-5.54302868542530	0.74737392723214	-0.00000226087949
H	-7.83750006305509	-1.79050460027632	0.00000882042233
H	-7.70009156854353	0.68160873251891	-0.00000006586719
H	-5.77583379707699	-3.16006466684803	0.00000954216194
N	-3.29677951974707	-2.24865388938108	0.00000365423896
H	-3.45742404212782	-3.24867448244571	0.00000552616510
N	-4.07160146056477	2.79045873029951	-0.00000940569771
B	-5.40010562128602	2.26614405784051	-0.00000786996629
H	-3.91590075540438	3.79088447915359	-0.00001390931216
H	-6.31208336065431	3.03931245382689	-0.00001330074470

Table S35. Cartesian coordinates for T_1 optimized geometry of $\mathbf{3^H}$ in solution (2-MeTHF).

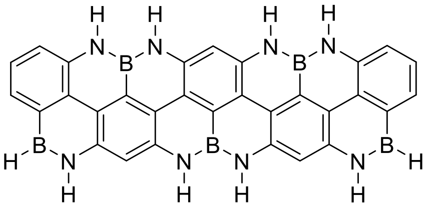

Atom	x	y	z
C	4.92181859149513	1.91586781917543	-0.00000546746811
C	3.69818909306538	2.60312207723078	-0.00000648048013
C	2.46959681401799	1.92573789244995	-0.00000158037340
C	2.46314230821274	0.49044559476067	0.00000026959209
C	3.70555634202198	-0.19526781633104	0.00000115323254
C	4.94070838710082	0.48896711085221	-0.00000253472401
B	3.69981563336109	-1.70341864393925	0.00000337222895
C	1.23971036853976	-0.24851716700211	0.00000255404668
C	0.00000032769256	0.43865272497345	0.00000233846521
C	1.22838488516411	-1.68652592172974	0.00000209688368
C	-0.00000006975618	-2.36709327207151	0.00000098769537
C	-1.22838477217930	-1.68652515061753	0.00000045821590
C	-1.23970958104772	-0.24851660868426	0.00000160849494
N	2.42221553869635	-2.37332448392981	0.00000344257313
H	3.70326194374312	3.69040996548273	-0.00000894083959
H	0.00000036770807	-3.45431464403331	0.00000095093632
C	-2.46314232901950	0.49044579954189	0.00000323609806
C	-3.70555619147782	-0.19526739709299	0.00000050453220
C	-4.94070847514551	0.48896755425080	0.00000307858711
C	-4.92181870799829	1.91586809272875	0.00000649923247
C	-3.69818933374966	2.60312274345864	0.00000622895832
C	-2.46959704632338	1.92573782572435	0.00000367156554
H	-3.70326136777225	3.69041054503150	0.00000733125149
B	-3.69981544373954	-1.70341821819329	-0.00000132295850
N	-2.42221551050451	-2.37332407383275	-0.00000008574823

N	-1.27396612145610	2.61485450390781	0.00000356680558
B	-0.00000008512861	1.94172695695732	0.00000265270736
N	1.27396585083300	2.61485457484524	0.00000022823065
H	2.34361448045562	-3.38353290032624	0.00000426201446
H	-2.34361502926343	-3.38353236871478	-0.00000027649723
H	-1.35377887263644	3.62487737561731	0.00000394840914
H	1.35377891491446	3.62487719762779	-0.00000249869225
C	6.18153137245635	-0.24650006209864	-0.00000066952445
C	6.17009651994323	-1.67803291685756	0.00000263331934
C	7.38785097994153	-2.37399460193574	0.00000555160294
C	8.60489131595173	-1.68764896715536	0.00000333734857
C	8.62869902722318	-0.29824357228811	-0.00000090257437
C	7.43299086620941	0.44676204302226	-0.00000356104281
H	9.53276378518115	-2.25203865049058	0.00000558876034
H	9.58064414924751	0.22530812911719	-0.00000310436533
H	7.37308402839219	-3.46075544215409	0.00000826094340
N	4.96532594952474	-2.37019351669904	0.00000395554643
H	5.04920781335105	-3.37946838410103	0.00000572088223
N	6.12377104602994	2.59906350272826	-0.00000864027543
B	7.40319084119410	1.97432448883900	-0.00000748234723
H	6.04431769324731	3.60837910601383	-0.00000942062154
H	8.37345595298563	2.67385534108506	-0.00000991947899
C	-6.18153115137115	-0.24650022733362	-0.00000091650364
C	-6.17009651209910	-1.67803296246061	-0.00000358142895
C	-7.38785097032506	-2.37399466039833	-0.00000746377527
C	-8.60489135372407	-1.68764891042699	-0.00000788150374
C	-8.62869888931100	-0.29824361040779	-0.00000571792046
C	-7.43299055095703	0.44676182388479	-0.00000138488656
H	-7.37308398617515	-3.46075548218213	-0.00000903275186
H	-9.53276377704074	-2.25203871069162	-0.00001070551854
H	-9.58064395740395	0.22530822253679	-0.00000628389667
N	-4.96532568614625	-2.37019327578065	-0.00000257739339
H	-5.04920745375544	-3.37946815676384	-0.00000346319804
N	-6.12377143794920	2.59906348806871	0.00000926765053
B	-7.40319108409083	1.97432435255482	0.00000471304495
H	-6.04431891538709	3.60837916034011	0.00001239501442
H	-8.37345652496691	2.67385476391690	0.00000603191838

Table S36. Cartesian coordinates for T_1 optimized geometry of 4^H in solution (2-MeTHF).

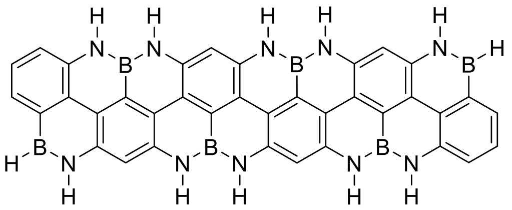

Atom	x	y	z
C	3.12803697389201	1.71628692168054	0.00687122321623
C	1.92249658427566	2.43141055793633	0.00689657339211
C	0.67310486862362	1.78611064307049	0.00696216968648
C	0.62327607303497	0.35271890797620	0.00694614986064
C	1.84114568256845	-0.36866389747354	0.00691269342284
C	3.09725244599973	0.28055306765894	0.00686869467801
B	1.79632366094542	-1.87586215186500	0.00695703963196
C	-0.62327679935589	-0.35271783996936	0.00694220521253
C	-1.84114580600992	0.36866595456737	0.00690100598760
C	-0.67310427495548	-1.78610924085226	0.00694439044222
C	-1.92249606925255	-2.43140925977456	0.00686725089813
C	-3.12803664224685	-1.71628585149983	0.00683782139722
C	-3.09725274583047	-0.28055165604335	0.00684504071168
N	0.50195944060037	-2.50848261929803	0.00701841593142
H	1.95428193488150	3.51830253455777	0.00689044083754
H	-1.95428147882169	-3.51830114991128	0.00685373612320
C	-4.30273195738723	0.49734170961976	0.00678244299234
C	-5.56144300122456	-0.15038184904353	0.00665991928903
C	-6.77666540772928	0.56928515623983	0.00659282577345
C	-6.71641093060813	1.99136921061345	0.00663084669397
C	-5.47315814096570	2.64174356582653	0.00673539192998
C	-4.26610235091523	1.92863789721438	0.00682405373910
H	-5.44560633486826	3.72883428468839	0.00680255873038
B	-5.60044166737109	-1.66231124900442	0.00664982267784
N	-4.34507814056717	-2.36898480490421	0.00679353140544
N	-3.04730019044222	2.58420639745068	0.00695151974714
B	-1.79632352173448	1.87586362846482	0.00695551273659
N	-0.50195898677537	2.50848439002380	0.00703363744601
H	0.39272791050051	-3.51566845438993	0.00707882024228
H	-4.29634633701975	-3.38083093035912	0.00681509278507
H	-3.09787827208616	3.59592890313498	0.00697609420837
H	-0.39272748892365	3.51567015386824	0.00709978579038
C	4.30273235744803	-0.49734057776468	0.00681633286299
C	4.26610179688043	-1.92863655683293	0.00685147792582
C	5.47315705594547	-2.64174295326042	0.00677151304007
C	6.71640969026506	-1.99136925902636	0.00668888780276
C	6.77666483713214	-0.56928552088069	0.00665547246664
C	5.56144293734261	0.15038209982348	0.00671123278719
H	5.44560435355304	-3.72883360790948	0.00683290474881

N	3.04729982026382	-2.58420514854341	0.00696039615055
H	3.09787791994295	-3.59592782009102	0.00698169617080
N	4.34507898927323	2.36898563305891	0.00684235031468
B	5.60044225571340	1.66231238969507	0.00670485668202
H	4.29634724791304	3.38083171770894	0.00686858169569
C	-8.04021962261115	-0.13132703098448	0.00645261786334
C	-8.07071704746423	-1.56137903464386	0.00638125985157
C	-9.30709707176501	-2.21979321285048	0.00616186708528
C	-10.50461228727026	-1.49868059284431	0.00606451777643
C	-10.48731296038629	-0.10991988443342	0.00617203973674
C	-9.26923110582933	0.59769360840454	0.00636188683172
H	-9.32506932884921	-3.30659708805945	0.00606439986060
H	-11.44824819095668	-2.03632511039440	0.00590234089624
H	-11.42281769876123	0.44250501755110	0.00612414866475
N	-6.88444773026179	-2.28948764451692	0.00651596277970
H	-6.99867418503649	-3.29550080018047	0.00649362559107
N	-7.89988472919036	2.71208489279677	0.00657242740599
B	-9.19402333758466	2.12576281874499	0.00647588079809
H	-7.78993764985060	3.71834872472433	0.00655299268066
H	-10.14466013124440	2.85213828174558	0.00636636835310
C	8.04021945168334	0.13132624887262	0.00652759465250
C	8.07071794145199	1.56137776744826	0.00644377803265
C	9.30709833674627	2.21979116730093	0.00621729838549
C	10.50461329430956	1.49867835893842	0.00613026131486
C	10.48731304872609	0.10991745530493	0.00625405018621
C	9.26923057196711	-0.59769484550510	0.00645050303315
H	11.44824918018401	2.03632302883162	0.00595995462122
H	11.42281710775351	-0.44250860460932	0.00621856740760
H	9.32507160277929	3.30659505671516	0.00610825408984
N	6.88444882482154	2.28948733029630	0.00657399449861
H	6.99867583738300	3.29550039792208	0.00654968158118
N	7.89988349321637	-2.71208595833157	0.00664703334292
B	9.19402172367362	-2.12576448058082	0.00658845197669
H	7.78993544465166	-3.71834955408002	0.00661867603765
H	10.14465892581000	-2.85213963976444	0.00646715439898

Excitation energies at S_0, S_1 and T_1 optimized geometries (in 2-MeTHF):

Table S37. S_1 and T_1 excitation energies, $\Delta \boldsymbol{E}_{\boldsymbol{S_1-T_1}}$ energy gaps, and oscillator strengths $\boldsymbol{f_{osc_{S_1}}}$ for the B3LYP/6-311G*/CPCM(2-MeTHF) optimized S_0 geometries.

Molecule	E_{S_1} (eV)	E_{T_1} (eV)	$\Delta E_{S_1-T_1}$ (eV)	$f_{osc_{S_1}}$
**5^H**	4.21	3.38	0.83	0.187
**17^H**	3.67	3.12	0.54	0.517
**6^H**	3.60	2.83	0.77	0.179
**1^H**	3.14	2.58	0.56	0.625
**2^H**	2.87	2.39	0.48	1.126
**3^H**	2.70	2.29	0.41	1.600
**4^H**	2.57	2.22	0.45	1.934

Table S38. S_1 and T_1 excitation energies, $\Delta \boldsymbol{E}_{\boldsymbol{S_1-T_1}}$ energy gap, and oscillator strength $\boldsymbol{f_{osc_{S_1}}}$ for the B3LYP/6-311G*/CPCM(2-MeTHF) optimized S_1 geometries.

Molecule	E_{S_1} (eV)	E_{T_1} (eV)	$\Delta E_{S_1-T_1}$ (eV)	$f_{osc_{S_1}}$
**5^H**	3.92	3.08	0.84	0.252
**17^H**	3.42	2.85	0.57	0.146
**6^H**	3.38	2.53	0.85	0.206
**1^H**	2.94	2.34	0.60	0.685
**2^H**	2.69	2.18	0.51	1.213
**3^H**	2.53	2.11	0.42	1.701
**4^H**	2.41	2.05	0.36	2.027

Table S39. S_1 and T_1 excitation energies, $\Delta \boldsymbol{E}_{\boldsymbol{S_1-T_1}}$ energy gap, and oscillator strength $\boldsymbol{f_{osc_{S_1}}}$ for the B3LYP/6-311G*/CPCM (2-MeTHF) optimized T_1 geometries.

Molecule	E_{S_1} (eV)	E_{T_1} (eV)	$\Delta E_{S_1-T_1}$ (eV)	$f_{osc_{S_1}}$
**5^H**	3.91	2.71	1.21	0.377
**17^H**	3.41	2.57	0.83	0.665
**6^H**	3.33	2.36	0.97	0.234
**1^H**	2.93	2.32	0.61	0.703
**2^H**	2.69	2.16	0.53	1.123
**3^H**	2.53	2.10	0.44	1.721
**4^H**	2.41	2.04	0.37	2.054

Spin-orbit couplings at T_1 optimized geometries (in 2-MeTHF):

Table S40. Spin-orbit coupling (SOC) matrix elements in cm^{-1} of **5^H** at the B3LYP/6-311G*/CPCM (2-MeTHF) optimized T_1 geometry.

SOC	S_0	S_1	S_2	S_3	S_4	S_5	S_6	S_7	S_8	S_9	S_{10}
T_1	0.00	0.02	0.03	0.03	0.02	0.04	0.01	0.00	1.26	0.00	0.00
T_2	0.01	0.01	0.01	0.01	0.05	0.00	0.00	0.01	1.88	0.02	0.00
T_3	0.01	0.04	0.03	0.01	0.05	0.04	0.02	0.02	0.31	0.03	0.01
T_4	0.02	0.01	0.01	0.02	0.02	0.01	0.02	0.01	0.89	0.01	0.00
T_5	0.02	0.02	0.01	0.03	0.01	0.05	0.00	0.01	0.17	0.01	0.00
T_6	0.02	0.00	0.01	0.03	0.00	0.01	0.00	0.01	0.45	0.02	0.05
T_7	0.01	0.02	0.01	0.03	0.01	0.02	0.00	0.01	0.78	0.03	0.02
T_8	0.02	0.00	0.01	0.03	0.00	0.00	0.04	0.01	0.19	0.00	0.01
T_9	0.05	0.00	0.01	0.02	0.00	0.02	0.01	0.01	0.69	0.01	0.01
T_{10}	0.02	0.00	0.00	0.01	0.00	0.00	0.02	0.02	0.85	0.00	0.00

Table S41. Spin-orbit coupling (SOC) matrix elements in cm^{-1} of **17^H** at the B3LYP/6-311G*/CPCM (2-MeTHF) optimized T_1 geometry.

SOC	S_0	S_1	S_2	S_3	S_4	S_5	S_6	S_7	S_8	S_9	S_{10}
T_1	0.01	0.00	0.00	0.04	0.01	0.04	0.03	0.00	0.01	0.01	0.00
T_2	0.00	0.02	0.01	0.01	0.02	0.03	0.02	0.05	0.01	0.01	0.01
T_3	0.00	0.05	0.03	0.00	0.02	0.02	0.03	0.06	0.01	0.01	0.00
T_4	0.01	0.01	0.01	0.02	0.00	0.02	0.01	0.01	0.01	0.01	0.02
T_5	0.01	0.00	0.00	0.00	0.04	0.00	0.01	0.00	0.01	0.02	0.02
T_6	0.02	0.00	0.02	0.01	0.00	0.05	0.01	0.02	0.00	0.02	0.03
T_7	0.01	0.00	0.01	0.02	0.03	0.03	0.02	0.00	0.02	0.01	0.01
T_8	0.01	0.01	0.01	0.00	0.01	0.01	0.01	0.02	0.00	0.03	0.01
T_9	0.01	0.01	0.01	0.02	0.00	0.02	0.01	0.01	0.00	0.02	0.01
T_{10}	0.01	0.01	0.01	0.01	0.01	0.01	0.02	0.01	0.01	0.02	0.01

Table S42. Spin-orbit coupling (SOC) matrix elements in cm^{-1} of **6^H** at the B3LYP/6-311G*/CPCM (2-MeTHF) optimized T_1 geometry.

SOC	S_0	S_1	S_2	S_3	S_4	S_5	S_6	S_7	S_8	S_9	S_{10}
T_1	0.00	0.00	0.03	0.01	0.02	0.00	0.04	0.03	0.00	0.05	0.00
T_2	0.05	0.03	0.00	0.00	0.00	0.01	0.00	0.00	0.02	0.00	0.00
T_3	0.00	0.00	0.01	0.01	0.00	0.00	0.03	0.02	0.00	0.00	0.00
T_4	0.03	0.00	0.00	0.00	0.00	0.02	0.00	0.00	0.01	0.00	0.02
T_5	0.03	0.04	0.00	0.00	0.00	0.03	0.00	0.00	0.02	0.00	0.01
T_6	0.01	0.04	0.00	0.00	0.00	0.00	0.00	0.00	0.01	0.00	0.02
T_7	0.00	0.00	0.01	0.00	0.01	0.00	0.01	0.01	0.00	0.02	0.00
T_8	0.02	0.00	0.00	0.00	0.00	0.01	0.00	0.00	0.00	0.00	0.03
T_9	0.00	0.00	0.04	0.04	0.01	0.00	0.01	0.01	0.00	0.01	0.00
T_{10}	0.04	0.02	0.00	0.00	0.00	0.02	0.00	0.00	0.00	0.00	0.00

Table S43. Spin-orbit coupling (SOC) matrix elements in cm^{-1} of $\mathbf{1^H}$ at the B3LYP/6-311G*/CPCM (2-MeTHF) optimized T_1 geometry.

SOC	S_0	S_1	S_2	S_3	S_4	S_5	S_6	S_7	S_8	S_9	S_{10}
T_1	0.04	0.00	0.02	0.00	0.00	0.06	0.00	0.02	0.00	0.00	0.00
T_2	0.00	0.02	0.00	0.00	0.01	0.00	0.01	0.00	0.01	0.01	0.01
T_3	0.00	0.02	0.00	0.00	0.03	0.00	0.00	0.00	0.00	0.02	0.04
T_4	0.00	0.05	0.00	0.00	0.00	0.00	0.00	0.00	0.03	0.03	0.06
T_5	0.03	0.00	0.01	0.00	0.00	0.01	0.00	0.00	0.00	0.00	0.00
T_6	0.05	0.00	0.00	0.01	0.00	0.01	0.00	0.00	0.00	0.00	0.00
T_7	0.08	0.00	0.00	0.01	0.00	0.00	0.00	0.01	0.00	0.00	0.00
T_8	0.00	0.00	0.00	0.00	0.01	0.00	0.00	0.00	0.00	0.02	0.02
T_9	0.00	0.00	0.00	0.00	0.01	0.00	0.02	0.00	0.01	0.00	0.01
T_{10}	0.01	0.00	0.01	0.02	0.00	0.05	0.00	0.02	0.00	0.00	0.00

Table S44. Spin-orbit coupling (SOC) matrix elements in cm^{-1} of $\mathbf{2^H}$ at the B3LYP/6-311G*/CPCM (2-MeTHF) optimized T_1 geometry.

SOC	S_0	S_1	S_2	S_3	S_4	S_5	S_6	S_7	S_8	S_9	S_{10}
T_1	0.00	0.00	0.00	0.01	0.00	0.01	0.00	0.07	0.00	0.00	0.01
T_2	0.04	0.00	0.00	0.00	0.03	0.00	0.00	0.00	0.01	0.00	0.00
T_3	0.00	0.02	0.00	0.00	0.00	0.02	0.00	0.01	0.00	0.00	0.00
T_4	0.01	0.00	0.02	0.00	0.00	0.00	0.00	0.00	0.01	0.01	0.00
T_5	0.00	0.05	0.00	0.01	0.00	0.00	0.00	0.01	0.00	0.00	0.00
T_6	0.00	0.01	0.00	0.00	0.00	0.00	0.00	0.01	0.00	0.00	0.01
T_7	0.07	0.00	0.02	0.00	0.00	0.00	0.01	0.00	0.00	0.00	0.00
T_8	0.06	0.00	0.01	0.00	0.01	0.00	0.01	0.00	0.00	0.01	0.00
T_9	0.06	0.00	0.01	0.00	0.00	0.00	0.00	0.00	0.00	0.01	0.00
T_{10}	0.00	0.00	0.00	0.00	0.00	0.01	0.00	0.02	0.00	0.00	0.00

Table S45. Spin-orbit coupling (SOC) matrix elements in cm^{-1} of $\mathbf{3^H}$ at the B3LYP/6-311G*/CPCM (2-MeTHF) optimized T_1 geometry.

SOC	S_0	S_1	S_2	S_3	S_4	S_5	S_6	S_7	S_8	S_9	S_{10}
T_1	0.02	0.00	0.00	0.00	0.00	0.00	0.01	0.02	0.00	0.06	0.00
T_2	0.00	0.00	0.00	0.03	0.00	0.01	0.00	0.00	0.01	0.00	0.01
T_3	0.02	0.00	0.02	0.00	0.01	0.00	0.01	0.02	0.00	0.00	0.00
T_4	0.00	0.02	0.00	0.00	0.00	0.02	0.00	0.00	0.00	0.00	0.01
T_5	0.02	0.00	0.01	0.00	0.01	0.00	0.00	0.01	0.00	0.01	0.00
T_6	0.00	0.02	0.00	0.00	0.00	0.00	0.00	0.00	0.00	0.00	0.00
T_7	0.00	0.07	0.00	0.01	0.00	0.00	0.00	0.00	0.00	0.00	0.00
T_8	0.00	0.02	0.00	0.02	0.00	0.01	0.00	0.00	0.00	0.00	0.02
T_9	0.06	0.00	0.03	0.00	0.00	0.00	0.03	0.00	0.00	0.00	0.00
T_{10}	0.08	0.00	0.00	0.00	0.00	0.00	0.00	0.01	0.00	0.00	0.00

Table S46. Spin-orbit coupling (SOC) matrix elements in cm^{-1} of $\mathbf{4^H}$ at the B3LYP/6-311G*/CPCM (2-MeTHF) optimized T_1 geometry.

SOC	S_0	S_1	S_2	S_3	S_4	S_5	S_6	S_7	S_8	S_9	S_{10}
T_1	0.00	0.00	0.00	0.01	0.00	0.00	0.02	0.01	0.00	0.00	0.00
T_2	0.02	0.00	0.00	0.00	0.03	0.00	0.00	0.00	0.01	0.00	0.02
T_3	0.00	0.00	0.00	0.01	0.00	0.00	0.01	0.00	0.00	0.00	0.00
T_4	0.02	0.00	0.02	0.00	0.00	0.00	0.00	0.00	0.02	0.00	0.00
T_5	0.00	0.02	0.00	0.00	0.00	0.00	0.02	0.00	0.00	0.00	0.00
T_6	0.00	0.02	0.00	0.01	0.00	0.00	0.00	0.01	0.00	0.00	0.00
T_7	0.02	0.00	0.00	0.00	0.01	0.00	0.00	0.00	0.01	0.00	0.01
T_8	0.01	0.00	0.01	0.00	0.01	0.00	0.00	0.00	0.00	0.00	0.00
T_9	0.00	0.07	0.00	0.01	0.00	0.00	0.00	0.00	0.00	0.01	0.00
T_{10}	0.05	0.00	0.03	0.00	0.00	0.03	0.00	0.00	0.00	0.00	0.00

6. References

(1) Koziskova, J.; Hahn, F.; Richter, J.; Kožíšek, J. Comparison of Different Absorption Corrections on the Model Structure of Tetrakis(μ_2-Acetato)-Diaqua-Di-Copper(II). *Acta Chim. Slovaca* **2016**, *9*, 136–140.

(2) Sheldrick, G. M. *SHELXT* – Integrated Space-Group and Crystal-Structure Determination. *Acta Crystallogr. A Found. Adv.* **2015**, *71*, 3–8.

(3) Sheldrick, G. M. Crystal Structure Refinement with *SHELXL*. *Acta Crystallogr. C Struct. Chem.* **2015**, *71*, 3–8.

(4) Hübschle, C. B.; Sheldrick, G. M.; Dittrich, B. *ShelXle*: A Qt Graphical User Interface for *SHELXL*. *J. Appl. Crystallogr.* **2011**, *44*, 1281–1284.

(5) Dolomanov, O. V.; Bourhis, L. J.; Gildea, R. J.; Howard, J. A. K.; Puschmann, H. *OLEX2*: A Complete Structure Solution, Refinement and Analysis Program. *J. Appl. Crystallogr.* **2009**, *42*, 339–341.

(6) Lausi, A.; Polentarutti, M.; Onesti, S.; Plaisier, J. R.; Busetto, E.; Bais, G.; Barba, L.; Cassetta, A.; Campi, G.; Lamba, D.; Pifferi, A.; Mande, S. C.; Sarma, D. D.; Sharma, S. M.; Paolucci, G. Status of the Crystallography Beamlines at Elettra. *Eur. Phys. J. Plus* **2015**, *130*, 43.

(7) Kabsch, W. *XDS*. *Acta Crystallogr. D Biol. Crystallogr.* **2010**, *66*, 125–132.

(8) Winn, M. D.; Ballard, C. C.; Cowtan, K. D.; Dodson, E. J.; Emsley, P.; Evans, P. R.; Keegan, R. M.; Krissinel, E. B.; Leslie, A. G. W.; McCoy, A.; McNicholas, S. J.; Murshudov, G. N.; Pannu, N. S.; Potterton, E. A.; Powell, H. R.; Read, R. J.; Vagin, A.; Wilson, K. S. Overview of the CCP4 Suite and Current Developments. *Acta Crystallogr. D Biol. Crystallogr.* **2011**, *67*, 235–242.

(9) Evans, P. R.; Murshudov, G. N. How Good Are My Data and What Is The Resolution? *Acta Crystallogr. D Biol. Crystallogr.* **2013**, *69*, 1204–1214.

(10) Sheldrick, G. M. *SADABS*; University of Göttingen, Germany, 2014.

(11) Emsley, P.; Lohkamp, B.; Scott, W. G.; Cowtan, K. Features and Development of *Coot*. *Acta Crystallogr. D Biol. Crystallogr.* **2010**, *66*, 486–501.

(12) Spek, A. L. *PLATON* SQUEEZE: A Tool for the Calculation of the Disordered Solvent Contribution to the Calculated Structure Factors. *Acta Crystallogr. C Struct. Chem.* **2015**, *71*, 9–18.

(13) Gaussian 16, Revision A.03, Frisch, M. J.; Trucks, G. W.; Schlegel, H. B.; Scuseria, G. E.; Robb, M. A.; Cheeseman, J. R.; Scalmani, G.; Barone, V.; Petersson, G. A.; Nakatsuji, H.; Li, X.; Caricato, M.; Marenich, A. V.; Bloino, J.; Janesko, B. G.; Gomperts, R.; Mennucci, B.; Hratchian, H. P.; Ortiz, J. V.; Izmaylov, A. F.; Sonnenberg, J. L.; Williams-Young, D.; Ding, F.; Lipparini, F.; Egidi, F.; Goings, J.; Peng, B.; Petrone, A.; Henderson, T.; Ranasinghe, D.; Zakrzewski, V. G.; Gao, J.; Rega, N.; Zheng, G.; Liang, W.; Hada, M.; Ehara, M.; Toyota, K.; Fukuda, R.; Hasegawa, J.; Ishida, M.; Nakajima, T.; Honda, Y.; Kitao, O.; Nakai, H.; Vreven, T.; Throssell, K.; Montgomery, J. A., Jr.; Peralta, J. E.; Ogliaro, F.; Bearpark, M. J.; Heyd, J. J.; Brothers, E. N.; Kudin, K. N.; Staroverov, V. N.; Keith, T. A.; Kobayashi, R.; Normand, J.; Raghavachari, K.; Rendell, A. P.; Burant, J. C.; Iyengar, S. S.; Tomasi, J.; Cossi, M.; Millam, J. M.; Klene, M.; Adamo, C.; Cammi, R.; Ochterski, J. W.; Martin, R. L.; Morokuma, K.; Farkas, O.; Foresman, J. B.; Fox, D. J. Gaussian, Inc., Wallingford CT, **2016**.

(14) Becke, A. D. Density-Functional Thermochemistry. III. The Role of Exact Exchange. *J. Chem. Phys.* **1993**, *98*, 5648–5652.

(15) Stephens, P. J.; Devlin, F. J.; Chabalowski, C. F.; Frisch, M. J. Ab Initio Calculation of Vibrational Absorption and Circular Dichroism Spectra Using Density Functional Force Fields. *J. Phys. Chem.* **1994**, *98*, 11623–11627.

(16) Vosko, S. H.; Wilk, L.; Nusair, M. Accurate Spin-Dependent Electron Liquid Correlation Energies for Local Spin Density Calculations: A Critical Analysis. *Can. J. Phys.* **1980**, *58*, 1200–1211.

(17) Krishnan, R.; Binkley, J. S.; Seeger, R.; Pople, J. A. Self-Consistent Molecular Orbital Methods. XX. A Basis Set for Correlated Wave Functions. *J. Chem. Phys.* **1980**, *72*, 650–654.

(18) Neese, F. The ORCA Program System. *WIREs Comput. Mol. Sci.* **2012**, *2*, 73–78.

(19) Neese, F.; Wennmohs, F.; Hansen, A.; Becker, U. Efficient, Approximate and Parallel Hartree–Fock and Hybrid DFT Calculations. A 'Chain-of-Spheres' Algorithm for the Hartree–Fock Exchange. *Chem. Phys.* **2009**, *356*, 98–109.

(20) Weigend, F. Accurate Coulomb-Fitting Basis Sets for H to Rn. *Phys. Chem. Chem. Phys.* **2006**, *8*, 1057.

(21) Hirata, S.; Head-Gordon, M. Time-Dependent Density Functional Theory Within the Tamm–Dancoff Approximation. *Chem. Phys. Lett.* **1999**, *314*, 291–299.

(22) MacNeil, S. L.; Wilson, B. J.; Snieckus, V. Anionic *N*-Fries Rearrangement of *N*-Carbamoyl Diarylamines to Anthranilamides. Methodology and Application to Acridone and Pyranoacridone Alkaloids. *Org. Lett.* **2006**, *8*, 1133–1136.

(23) Roscales, S.; Csákÿ, A. G. Synthesis of Di(Hetero)Arylamines from Nitrosoarenes and Boronic Acids: A General, Mild, and Transition-Metal-Free Coupling. *Org. Lett.* **2018**, *20*, 1667–1671.

(24) Zhang, W.; Li, G.; Xu, L.; Zhuo, Y.; Wan, W.; Yan, N.; He, G. 9,10-Azaboraphenanthrene-Containing Small Molecules and Conjugated Polymers: Synthesis and Their Application in Chemodosimeters for the Ratiometric Detection of Fluoride Ions. *Chem. Sci.* **2018**, *9*, 4444–4450.

(25) McDonald, K. P.; Ramabhadran, R. O.; Lee, S.; Raghavachari, K.; Flood, A. H. Polarized Naphthalimide CH Donors Enhance Cl^- Binding within an Aryl-Triazole Receptor. *Org. Lett.* **2011**, *13*, 6260–6263.

(26) Zhang, W.; Zhang, F.; Tang, R.; Fu, Y.; Wang, X.; Zhuang, X.; He, G.; Feng, X. Angular BN-Heteroacenes with *Syn*-Structure-Induced Promising Properties as Host Materials of Blue Organic Light-Emitting Diodes. *Org. Lett.* **2016**, *18*, 3618–3621.

(27) Kelly, T. R.; Bridger, G. J.; Zhao, C. Bisubstrate Reaction Templates. Examination of the Consequences of Identical Versus Different Binding Sites. *J. Am. Chem. Soc.* **1990**, *112*, 8024–8034.

(28) Fan, W.; Han, Y.; Wang, X.; Hou, X.; Wu, J. Expanded Kekulenes. *J. Am. Chem. Soc.* **2021**, *143*, 13908–13916.

(29) Li, G.; Xiong, W.-W.; Gu, P.-Y.; Cao, J.; Zhu, J.; Ganguly, R.; Li, Y.; Grimsdale, A. C.; Zhang, Q. 1,5,9-Triaza-2,6,10-Triphenylboracoronene: BN-Embedded Analogue of Coronene. *Org. Lett.* **2015**, *17*, 560–563.
